# Supplementary material for: Modular assembly of indole alkaloids enabled by multicomponent reaction
Source: Nat Commun. 2023 Aug 9;14:4806. doi: 10.1038/s41467-023-40598-y (PMC10412628; doi:10.1038/s41467-023-40598-y)
Supplement: Supplementary file 1 — Supplementary Information [file 41467_2023_40598_MOESM1_ESM.pdf]

Supplementary Information

Modular Assembly of Indole Alkaloids Enabled by Multicomponent Reaction

Jiaming Li[1], Zhencheng Lai[1], Weiwei Zhang[1], Linwei Zeng[1], and Sunliang Cui[1,*]

[1]Institute of Drug Discovery and Design, National Key Laboratory of Advanced Drug Delivery and Release Systems, College of Pharmaceutical Sciences, Zhejiang University, Hangzhou 310058, China

*Correspondence to: slcui@zju.edu.cn

Table of Content

1. General Considerations 3

2. Starting Materials 4

3. Optimization of the Reaction Conditions 26

4. Procedures of the Indole Alkaloids Synthesis and Characterization 31

5. Procedures of Synthetic Application 71

6. Mechanistic Investigation 82

7. X-ray Crystallographic Data 102

8. Copies of NMR Spectra 107

9. References 266

1. General Considerations

^{1}H NMR and ^{13}C NMR spectra were recorded on a Bruker AV-600 spectrometer (College of Life Sciences, Zhejiang University), a Bruker AV-500 spectrometer (College of Pharmaceutical Science, Zhejiang University) or a WNMR-I-400 spectrometer (Department of Chemistry, Zhejiang University) in chloroform-*d* ($CDCl_3$, contain internal TMS) or DMSO-d_6, or CD_3OD. For $CDCl_3$ as solvent, chemical shifts of ^{1}H NMR spectra were reported in ppm with the internal TMS signal at 0 ppm as a standard, and chemical shifts of ^{13}C NMR spectra were reported in ppm with the chloroform signal at 77.16 ppm as a standard. With respect to DMSO-d_6 as solvent, chemical shifts of ^{1}H NMR and ^{13}C NMR spectra were reported in ppm with the DMSO-d_6 signal at 2.50 ppm and 39.52 ppm as the standard respectively. With respect to CD_3OD as solvent, chemical shifts of ^{1}H NMR and ^{13}C NMR spectra were reported in ppm with the CD_3OD signal at 3.31 ppm and 49,00 ppm as the standard respectively[1]. ^{19}F NMR spectra were recorded on a Bruker AV-600 spectrometer. The data is being reported as (s = singlet, d = doublet, t = triplet, q = quartet, quint = quintet, hept = heptet, dd = double doublet, dt = double of triplet, m = multiplet or unresolved, br = broad singlet, coupling constant(s) in Hz, integration).

HRMS were performed on Agilent Technologies 6546-LC/Q-TOF mass spectrometer (ESI-TOF) (College of Pharmaceutical Sciences, Zhejiang University).

X-Ray crystallographic analyses were performed collected on a ‘Bruker D8 Venture’ diffractometer (CuK\a radiation, radiation wavelength = 1.54178) or ‘Xcalibur, Atlas, Gemini ultra’ diffractometer (MoK\a radiation, radiation wavelength = 0.71073), (Department of Chemistry, Zhejiang University).

All reagents and solvents, such as ethyl acetate (EA), petroleum ether (PE), methanol (MeOH), toluene (PhMe), 1,4-dioxane (dioxane), *N,N*-Dimethylformamide (DMF), tetrahydrofuran (THF), dichloromethane (DCM), acetonitrile (MeCN), 1,2-dichloroethane (DCE), triethylamine (TEA) were commercially available and used directly without further purification unless stated otherwise.

Reactions were conducted at Heidolph thermostatic magnetic stirrer, and monitored by thin layer chromatography (TLC) using silicycle pre-coated silica gel plates. The products were purified by column chromatography performed over silica gel (200–300 mesh), or recrystallized from suitable solvents.

2. Starting Materials

2.1 Synthesis of starting indoles and pyrroles

The used indoles in this work were listed in Supplementary Figure 1, and were prepared according to the reported methods or obtained commercially available. Indoles **7a-7d, 7g, 7j-7n** were purchased from Shanghai Bide Pharmatech Ltd. Indoles **7o** and **7p** were prepared according to the **Method E**. Indoles **1a-1d**, **1g**, **1j-1m** were prepared according to the reported **Method A**; Indole **1f** was synthesized from $Zn(CN)_2$ according to **Method B**; Indoles **1h** and **1i** were synthesized from corresponding pinacol boric ester according to the **Method C**; Indoles **1n**, **1o**, **1q**, **1s**, **5a**, **5d-5f**, **12a** were prepared according to the reported **Method D** and **Method E**; Indoles **1p**, **1r**, **10a**, **11a** were prepared according to the **Method F**; Indoles **5b** and **5c** were synthesized according to the **Method G**; Indole **1e** was synthesized according to the **Method H**. Pyrroles **1t** and **1u** were prepared according to the **Method I**. Indoles **5g** and **5h** were prepared according to the reported **Method J**. Indole [D]-**1a** was synthesized according to the **Method K**, and indole [D_3]-**1a** was prepared according to the **Method L**. Indole [D]-**5a** was synthesized according to the **Method M**, and indole [D_3]-**5a** was synthesized according to the **Method N**.

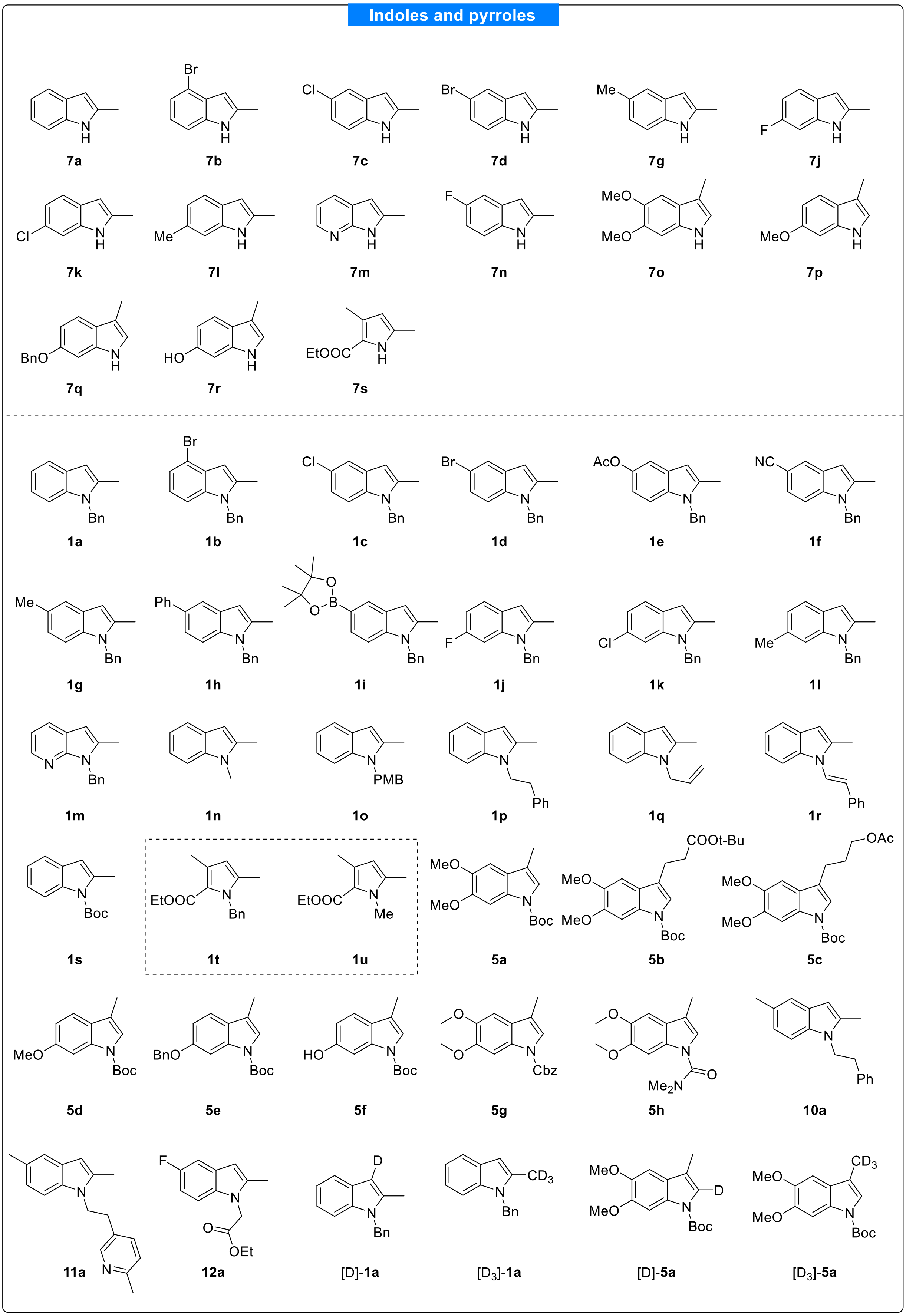

Supplementary Figure 1. Starting indoles. All used indoles and pyrroles in this protocol are listed.

2.1.1 **Method A** for the preparation of indoles[2]

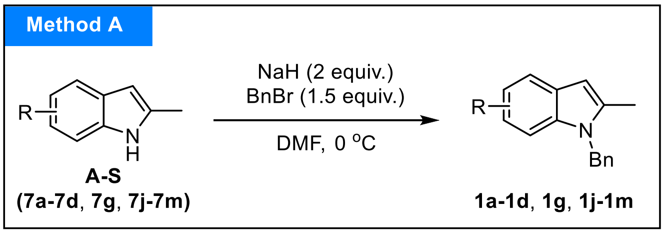

A flask equipped with a magnetic stir bar was charged with indoles **A-S** (1 mmol). DMF (5 mL) was added as solvent. NaH (2 mmol, 2 equiv.) was added slowly and the reaction was stirred at 0 °C for 30 minutes. Then the benzyl bromide (1.5 mmol, 1.5 equiv.) was dissolved in DMF and added dropwise. The mixture was warmed to room temperature and stirred for 1 hour. Water was added to quench the reaction and the mixture was extracted with EtOAc. The organic layer was washed with brine, dried over anhydrous Na_2SO_4 and concentrated under vacuum to obtain the residue, which was purified by silica gel column chromatography to afford the corresponding starting indoles **1a-1d**, **1g**, **1j-1m**.

2.1.2 **Method B** for the preparation of indoles[3]

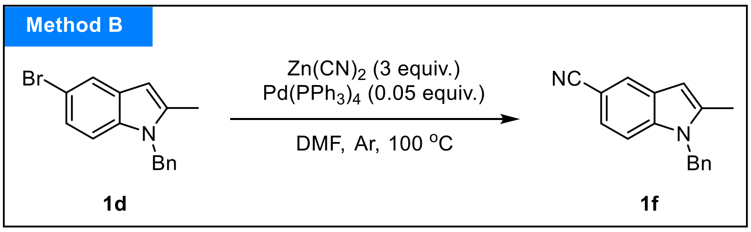

A two-neck flask equipped with a magnetic stir bar was charged with indoles **1d** (2 mmol), $Zn(CN)_2$ (6 mmol, 3 equiv.), $Pd(PPh_3)_4$ (5 mol%) and then purged with argon three times. DMF (8 mL) was added as solvent. The mixture was stirred at 100 °C for 2 hours. Water was added to quench the reaction and the mixture was extracted with EtOAc. The organic layer was washed with brine, dried over anhydrous Na_2SO_4 and concentrated under vacuum to obtain the residue, which was purified by silica gel column chromatography to afford the corresponding indole **1f**.

2.1.3 **Method C** for the preparation of indoles[4]

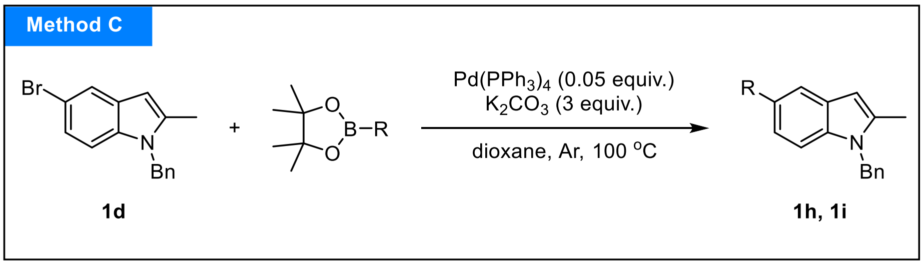

A two-neck flask equipped with a magnetic stir bar was charged with indoles **1d** (2 mmol), $Pd(PPh_3)_4$ (0.1 mmol, 0.05 equiv.), K_2CO_3 (6 mmol, 3 equiv.), corresponding pinacol boric ester (3 mmol, 1.5 equiv.) and then purged with argon three times. Dioxane (8 mL) was added as solvent. The reaction was stirred at 100 °C overnight. After completion, water was added to quench the reaction and the mixture was extracted with EtOAc. The organic layer was washed with brine, dried over anhydrous Na_2SO_4 and concentrated under vacuum to obtain the residue, which was purified by silica gel column chromatography to afford the corresponding starting indoles **1h** and **1i**.

2.1.4 **Method D** for the preparation of indoles[5-6]

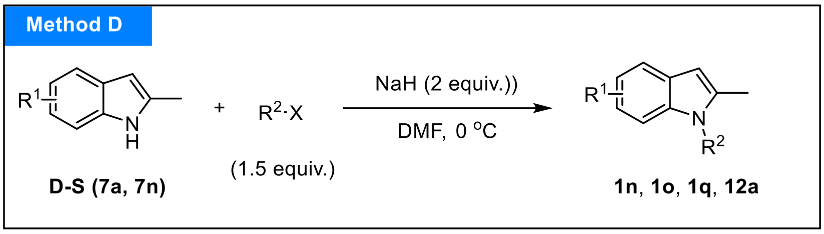

A flask equipped with a magnetic stir bar was charged with indoles **D-S** (1 mmol). DMF (5 mL) was added as solvent. NaH (2 mmol, 2 equiv.) was added slowly and the reaction was stirred at 0 °C for 30 minutes. Then the corresponding halogenated compounds (1.5 mmol, 1.5 equiv.) was dissolved in DMF and added dropwise. The mixture was warmed to room temperature and stirred for 1 hour. Water was added to quench the reaction and the mixture was extracted with EtOAc. The organic layer was washed with brine, dried over anhydrous Na_2SO_4 and concentrated under vacuum to obtain the residue, which was purified by silica gel column chromatography to afford the corresponding starting indoles **1n**, **1o**, **1q** and **12a**.

2.1.5 **Method E** for the preparation of indoles[7-8]

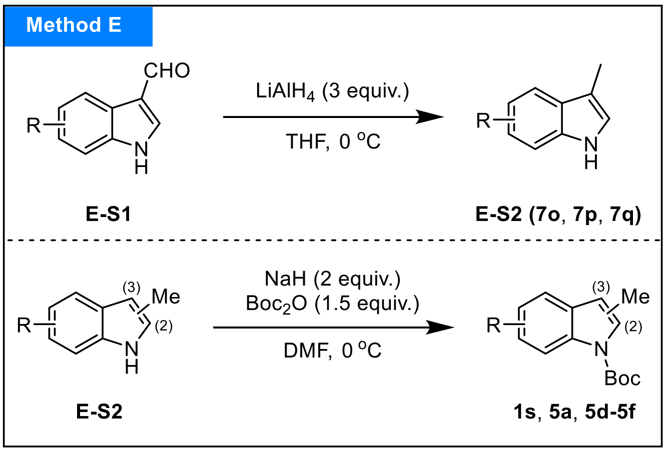

A flask equipped with a magnetic stir bar was charged with 3-formaldehyde indole **E-S1** (1 mmol). THF (5 mL) was added as solvent. $LiAlH_4$ (3 mmol, 3 equiv.) was added slowly, and the reaction was stirred at 0 °C for 1 hour. Water was added to quench the reaction and the mixture was extracted with EtOAc. The organic layer was washed with brine, dried over anhydrous Na_2SO_4 and concentrated under vacuum. The residue was purified by silica gel column chromatography to afford the corresponding methyl indole intermediates **E-S2 (7o, 7p** and **7q)**.

A flask equipped with a magnetic stir bar was charged with methyl indoles **E-S2** (1 mmol). DMF (5 mL) was added as solvent. NaH (2 mmol, 2 equiv.) was added and the reaction was stirred at 0 °C for 30 minutes. Then the Boc_2O (1.5 mmol, 1.5 equiv.) was dissolved in DMF and added dropwise. The mixture was warmed to room temperature and stirred for 1 hour. Water was added to quench the reaction and the mixture was extracted with EtOAc. The organic layer was washed with brine, dried over anhydrous Na_2SO_4 and concentrated under vacuum. The residue was purified by silica gel column chromatography to afford indoles **1s**, **5a**, **5d-5f**.

2.1.6 Method F for the preparation of indoles[9]

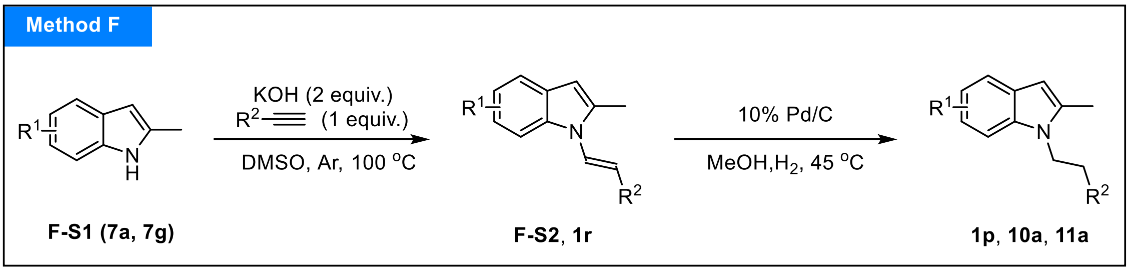

A two-neck flask equipped with a magnetic stir bar was charged with indoles **F-S1** (2 mmol), KOH (4 mmol, 2 equiv.), and then purged with argon three times. Corresponding alkynes (2 mmol, 1 equiv.) and DMSO (8 mL) was added. The reaction was stirred at 100 °C for 2 hours. After completion, water was added to quench the reaction and the mixture was extracted with EtOAc. The organic layer was washed with brine, dried over anhydrous Na_2SO_4 and concentrated under vacuum to obtain the residue, which was purified by silica gel column chromatography to afford the corresponding alkene indoles **F-S2** and **1r**.

To a solution of **F-S2** or **1r** (1 mmol) in MeOH (5 mL) was added 10% Pd/C (10% wt.). Then the mixture was purged with hydrogen three times. The resulting reaction mixture was kept at 45 °C for 2 hours. After completion, the resulting mixture was concentrated in vacuo; and purified by silica gel column chromatography to afford the starting indoles **1p**, **10a**, **11a**.

2.1.7 **Method G** for the preparation of indoles[10]

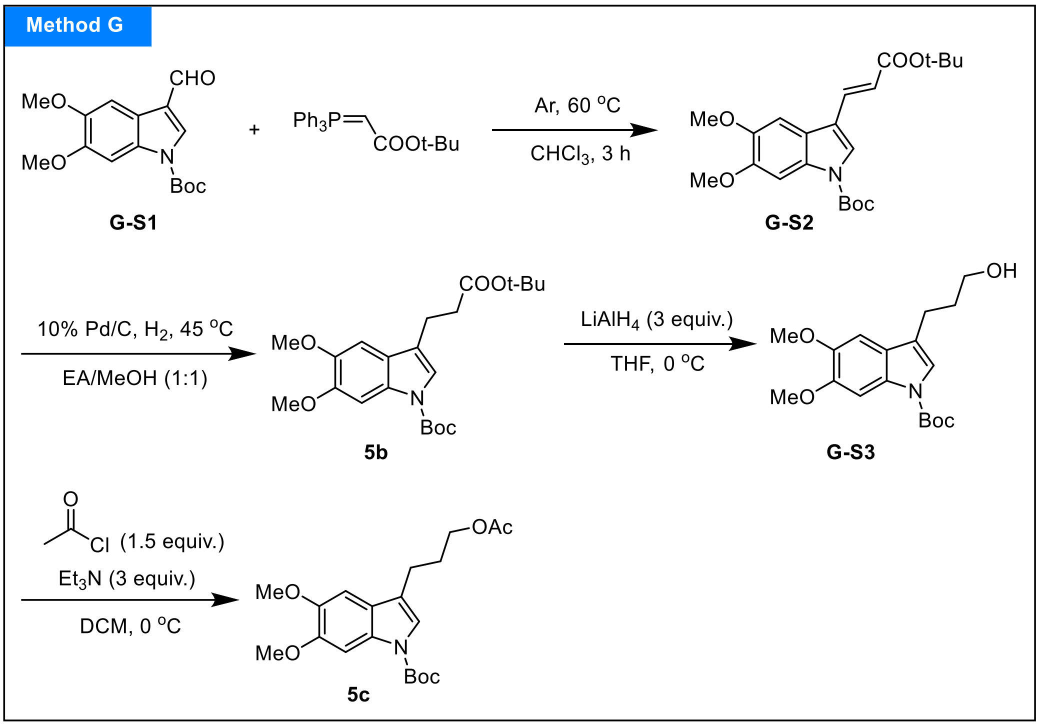

A two-neck flask equipped with a magnetic stir bar was charged with 3-formaldehyde indole **G-S1** (2 mmol), *tert*-butyl(triphenylphosphoranylidene)acetate (2.4 mmol, 1.2 equiv.), and then purged with argon three times. $CHCl_3$ (10 mL) was added as solvent. The reaction was stirred at 60 °C for 3 hours. After completion, water was added to quench the reaction and the mixture was extracted with EtOAc. The organic layer was washed with brine, dried over anhydrous Na_2SO_4 and concentrated under vacuum to obtain the residue, which was purified by silica gel column chromatography to afford the corresponding intermediate **G-S2**.

To a solution of **G-S2** (1 mmol) in MeOH (3 mL) and EA (3 mL) was added 10% Pd/C (10% wt.). Then the mixture was purged with hydrogen three times. The resulting reaction mixture was kept at 45 °C for 2 hours. After completion, the resulting mixture was concentrated in vacuo; and purified by silica gel column chromatography to afford the starting indoles **5b**.

A flask equipped with a magnetic stir bar was charged with **5b** (1 mmol). THF (5 mL) was added as solvent. $LiAlH_4$ (3 mmol, 3 equiv.) was added slowly and the reaction was stirred at 0 °C for 1 hour. Water was added to quench the reaction and the mixture was extracted with EtOAc. The organic layer was washed with brine, dried over anhydrous Na_2SO_4 and concentrated under vacuum to obtain the residue, which was purified by silica gel column chromatography to afford the corresponding intermediate **G-S3**.

A two-neck flask equipped with a magnetic stir bar was charged with **G-S3** (0.5 mmol) and then purged with argon three times. Triethylamine (1.5 mmol, 3 equiv.) and DCM (3 mL) was added. The reaction was stirred at 0 °C for 10 minutes. Then acetyl chloride (0.75 mmol, 1.5 equiv.) was added dropwise. The mixture was stirred at room temperature for 1 hour. After completion, water was added to quench the reaction and the mixture was extracted with EtOAc. The organic layer was washed with brine, dried over anhydrous Na_2SO_4 and concentrated under vacuum to obtain the residue, which was purified by silica gel column chromatography to afford the corresponding indoles **5c**.

2.1.8 Method H for the preparation of indoles

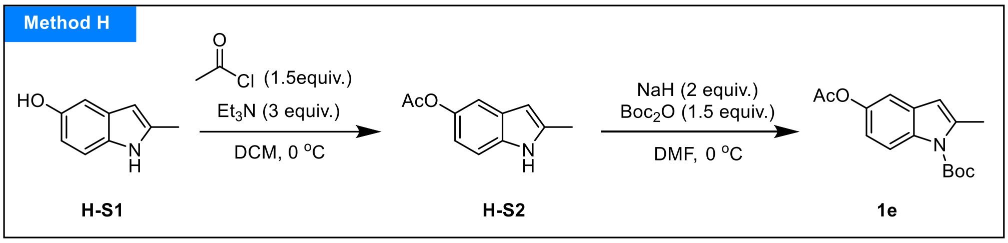

A two-neck flask equipped with a magnetic stir bar was charged with **H-S1** (2 mmol) and then purged with argon three times. Triethylamine (6 mmol, 3 equiv.) and DCM (10 mL) was added. The reaction was stirred at 0 °C for 10 minutes. Then acetyl chloride (3 mmol, 1.5 equiv.) was added dropwise. The mixture was stirred at room temperature for 1 hour. After completion, water was added to quench the reaction and the mixture was extracted with EtOAc. The organic layer was washed with brine, dried over anhydrous Na_2SO_4 and concentrated under vacuum to obtain the residue, which was purified by silica gel column chromatography to afford the intermediate **H-S2**.

A flask equipped with a magnetic stir bar was charged with indoles **H-S2** (1 mmol). DMF (5 mL) was added as solvent. NaH (2 mmol, 2 equiv.) was added slowly and the reaction was stirred at 0 °C for 30 minutes. Then the Boc_2O (1.5 mmol, 1.5 equiv.) was dissolved in DMF and added dropwise. The mixture was warmed to room temperature and stirred for 1 hour. Water was added to quench the reaction and the mixture was extracted with EtOAc. The organic layer was washed with brine, dried over anhydrous Na_2SO_4 and concentrated under vacuum to obtain the residue, which was purified by silica gel column chromatography to afford the corresponding starting indole **1e**.

2.1.9 Method I for the preparation of pyrroles

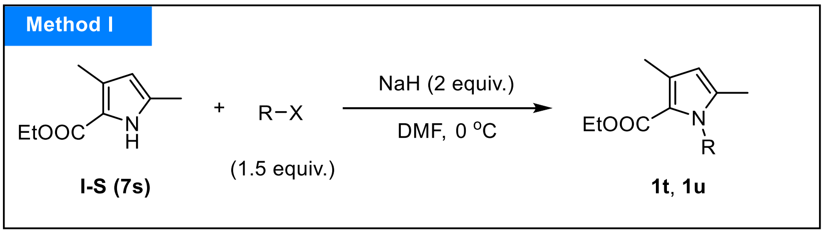

A flask equipped with a magnetic stir bar was charged with pyrroles **I-S** (1 mmol). DMF (5 mL) was added as solvent. NaH (2 mmol, 2 equiv.) was added slowly and the reaction was stirred at 0 °C for 30 minutes. Then the corresponding halogenated compounds (1.5 mmol, 1.5 equiv.) was dissolved in DMF and added dropwise. The mixture was warmed to room temperature and stirred for 1 hour. Water was added to quench the reaction and the mixture was extracted with EtOAc. The organic layer was washed with brine, dried over anhydrous Na_2SO_4 and concentrated under vacuum to obtain the residue, which was purified by silica gel column chromatography to afford the corresponding pyrroles **1t** and **1u**.

2.1.10 Method J for the preparation of indoles

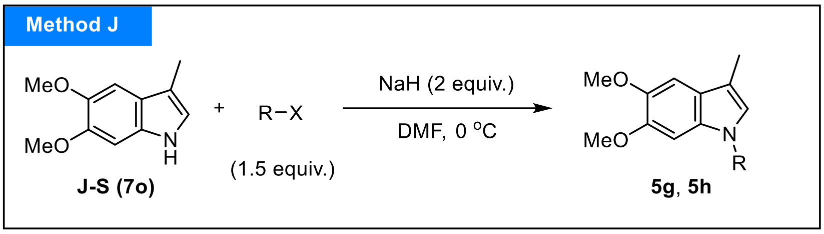

A flask equipped with a magnetic stir bar was charged with indoles **J-S** (1 mmol). DMF (5 mL) was added as solvent. NaH (2 mmol, 2 equiv.) was added slowly and the reaction was stirred at 0 °C for 30 minutes. Then the corresponding halogenated compounds (1.5 mmol, 1.5 equiv.) was dissolved in DMF and added dropwise. The mixture was warmed to room temperature and stirred for 1 hour. Water was added to quench the reaction and the mixture was extracted with EtOAc. The organic layer was washed with brine, dried over anhydrous Na_2SO_4 and concentrated under vacuum to obtain the residue, which was purified by silica gel column chromatography to afford the corresponding indoles **5g** and **5h**.

2.1.11 Method K for the preparation of indoles

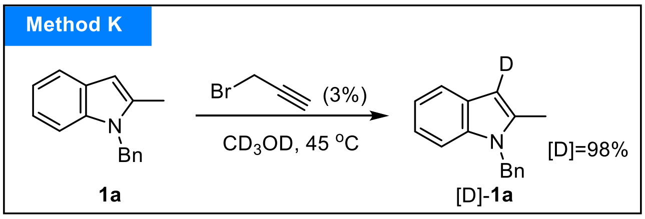

A flask equipped with a magnetic stir bar was charged with indoles **1a** (1 mmol). CD_3OD (2.5 mL) was added as solvent. 3-bromopropyne (3%) was added and the reaction was stirred at 45 °C for 8 hours. The mixture was concentrated under vacuum to obtain the the corresponding indole [D]-**1a**.

2.1.12 Method L for the preparation of indoles

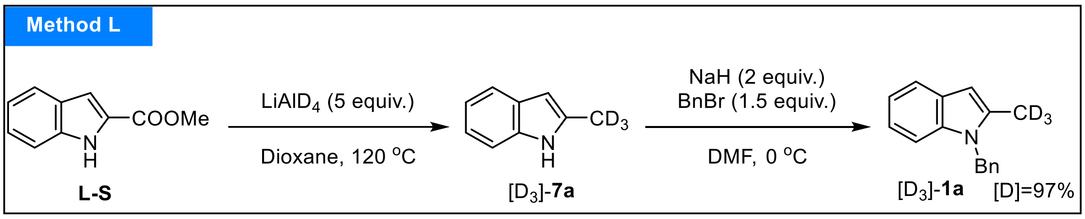

A flask equipped with a magnetic stir bar was charged with indoles **L-S** (1 mmol). Dioxane (5 mL) was added as solvent. $LiAlD_4$ (deuterated rate ≥ 98%, 5 mmol, 5 equiv.) was added slowly and the reaction was stirred at 120 °C overnight. Then the mixture was cooled to room temperature. Water was added to quench the reaction and the mixture was extracted with EtOAc. The organic layer was washed with brine, dried over anhydrous Na_2SO_4 and concentrated under vacuum to obtain the residue, which was purified by silica gel column chromatography to afford the corresponding indole [D_3]-**7a**.

A flask equipped with a magnetic stir bar was charged with [D_3]-**7a** (1 mmol). DMF (5 mL) was added as solvent. NaH (2 mmol, 2 equiv.) was added slowly and the reaction was stirred at 0 °C for 30 minutes. Then the benzyl bromide (1.5 mmol, 1.5 equiv.) was dissolved in DMF and added dropwise. The mixture was warmed to room temperature and stirred for 1 hour. Water was added to quench the reaction and the mixture was extracted with EtOAc. The organic layer was washed with brine, dried over anhydrous Na_2SO_4 and concentrated under vacuum to obtain the residue, which was purified by silica gel column chromatography to afford the corresponding starting indole [D_3]-**1a**.

2.1.13 **Method M** for the preparation of indoles

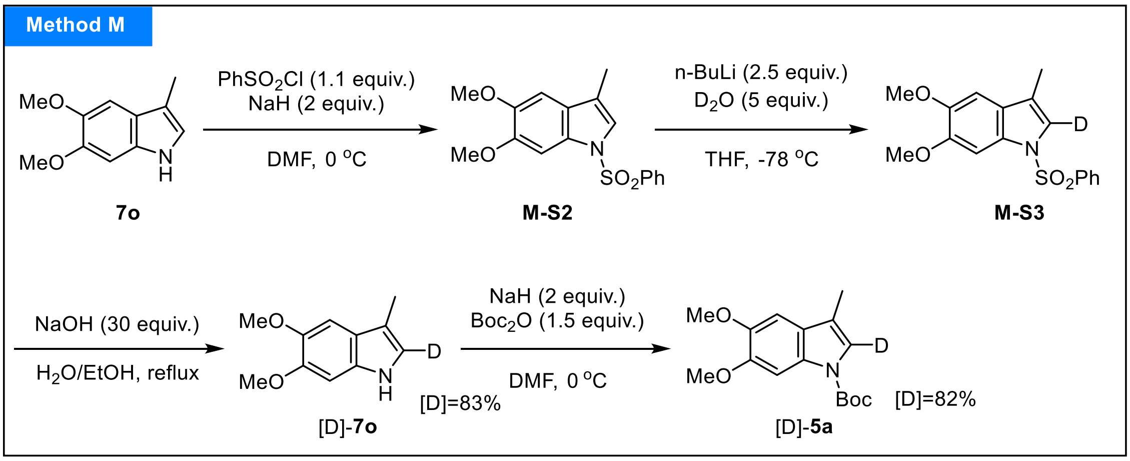

A flask equipped with a magnetic stir bar was charged with **7o** (1 mmol). DMF (5 mL) was added as solvent. NaH (2 mmol, 2 equiv.) was added slowly and the reaction was stirred at 0 °C for 30 minutes. Then the benzenesulfonyl chloride (1.1 mmol, 1.1 equiv.) was dissolved in DMF and added dropwise. The mixture was warmed to room temperature and stirred for 1 hour. Water was added to quench the reaction and the mixture was extracted with EtOAc. The organic layer was washed with brine, dried over anhydrous Na_2SO_4 and concentrated under vacuum to obtain the residue, which was purified by silica gel column chromatography to afford the intermediate **M-S2**.

A two-neck flask equipped with a magnetic stir bar was charged with **M-S2** (0.5 mmol) and then purged with argon three times. anhydrous THF (4 mL) was added as solvent. The mixture was stirred at -78 °C for 30 minutes. n-BuLi (2.5 equiv.) was added and the reaction was kept at -78 °C for 1 hour. Then D_2O (5 equiv.) was added and the mixture was extracted with EtOAc. The organic layer was washed with brine, dried over anhydrous Na_2SO_4 and concentrated under vacuum to obtain the crude **M-S3** for the further steps without purification.

To a solution of **M-S3** (0.5 mmol) in EtOH (10 mL) was added NaOH aqueous solution (15 mmol in 10 mL water.).The resulting reaction mixture was refluxed for 3 hours. After completion, the resulting mixture was extracted with EtOAc. The organic layer was washed with brine, dried over anhydrous Na_2SO_4 and concentrated under vacuum to obtain obtain the residue, which was purified by silica gel column chromatography to afford the intermediate [D]-**7o**.

A flask equipped with a magnetic stir bar was charged with [D]-**7o** (1 mmol). DMF (5 mL) was added as solvent. NaH (2 mmol, 2 equiv.) was added and the reaction was stirred at 0 °C for

30 minutes. Then the Boc_2O (1.5 mmol, 1.5 equiv.) was dissolved in DMF and added dropwise. The mixture was warmed to room temperature and stirred for 1 hour. Water was added to quench the reaction and the mixture was extracted with EtOAc. The organic layer was washed with brine, dried over anhydrous Na_2SO_4 and concentrated under vacuum. The residue was purified by silica gel column chromatography to afford indoles [D]-**5a**.

2.1.14 Method N for the preparation of indoles

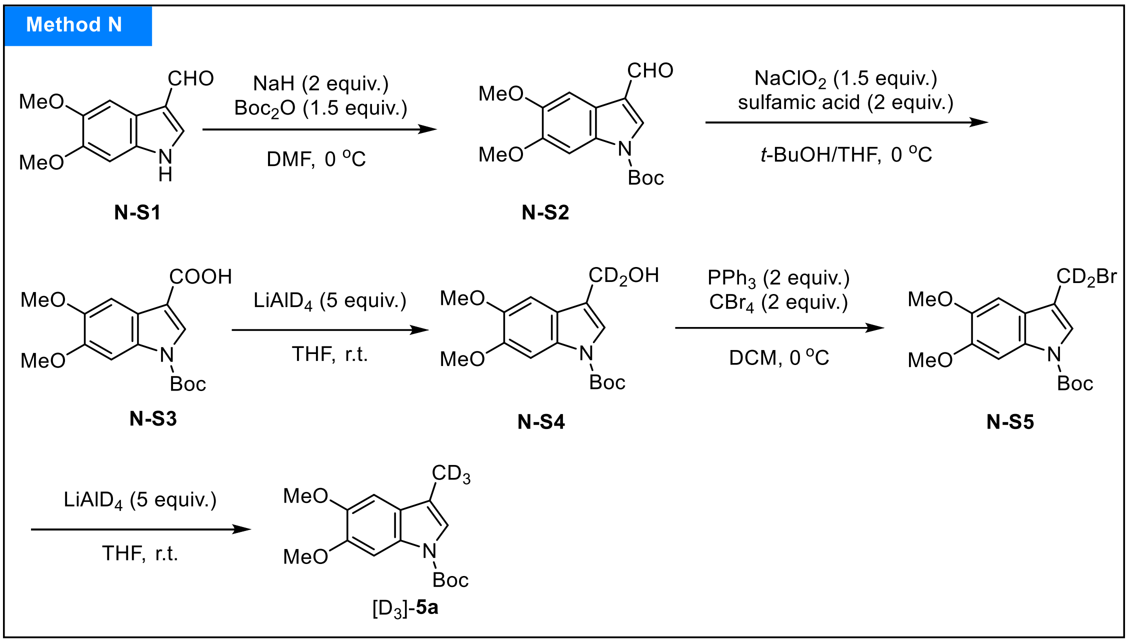

A flask equipped with a magnetic stir bar was charged with **N-S1** (1 mmol). DMF (5 mL) was added as solvent. NaH (2 mmol, 2 equiv.) was added and the reaction was stirred at 0 °C for 30 minutes. Then the Boc_2O (1.5 mmol, 1.5 equiv.) was dissolved in DMF and added dropwise. The mixture was warmed to room temperature and stirred for 1 hour. Water was added to quench the reaction and the mixture was extracted with EtOAc. The organic layer was washed with brine, dried over anhydrous Na_2SO_4 and concentrated under vacuum. The residue was purified by silica gel column chromatography to afford **N-S2** as white solid.

To a solution (*t*-BuOH and THF, 20 mL, 1:1) of **N-S2** (1 mmol, 1equiv.) and sulfamic acid (2 mmol, 2 equiv.) at 0 °C was added aqueous sodium chlorite solution (1.5 mmol, 1.5 equiv., in 5 mL water). The resulting mixture was kept at 0 °C for 2 hours. The reaction was diluted with water and extracted with ethyl acetate. The combined organic layer was dried over Na_2SO_4 and concentrated. The crude product (**N-S3**) was used for the further steps directly.

A schlenk tube equipped with a magnetic stirrer bar was charged with acid **N-S3** (0.5 mmol), THF (3 mL) was added as solvent. Then $LiAlD_4$ (deuterated rate ≥ 98%, 2.5 mmol, 5 equiv.) was added and the reaction was stirred at room temperature for 2 minutes. After completion, the mixture was quenched by water. The aqueous layer was extracted with ethyl acetate. Afterwards, the combined organic layer was dried over Na_2SO_4 and concentrated. The residue was purified by flash column chromatography on silica gel to give the intermediate **N-S4**.

A reaction tube equipped with a magnetic stir bar was charged with **N-S4** (1 mmol). DCM (5 mL) was added as solvent. the mixture was stirred at 0 ^{o}C for 10 minutes. Then the triphenylphosphine (2 mmol, 2 equiv.) and carbon tetrabromide (2 mmol, 2 equiv.) were added. The reaction was kept at 0 ^{o}C and stirred for 1 hour. Water was added to quench the reaction and the mixture was extracted with EtOAc. The organic layer was washed with brine, dried over anhydrous Na_2SO_4 and concentrated under vacuum to obtain the crude **N-S5** for the further steps without purification.

A schlenk tube equipped with a magnetic stirrer bar was charged with the acid **N-S5** (0.5 mmol), THF (3 mL) was added as solvent. Then $LiAlD_4$ (deuterated rate ≥ 98%, 2.5 mmol, 5 equiv.) was added and the reaction was stirred at room temperature for 2 minutes. After completion, the mixture was quenched by water. The aqueous layer was extracted with ethyl acetate. Afterwards, the combined organic layer was dried over Na_2SO_4 and concentrated. The residue was purified by flash column chromatography on silica gel to give the intermediate [D_3]-**5a**.

2.2 Characterization of new starting materials

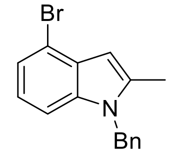

1b

1-benzyl-4-bromo-2-methyl-1*H*-indole (1b): off-white solid, m.p. = 75 – 77 °C, 90% yield. **^{1}H NMR (400 MHz, $CDCl_3$)** δ 7.33 – 7.25 (m, 4H), 7.18 (d, *J* = 8.0 Hz, 1H), 7.03 – 6.96 (m, 3H), 6.43 (t, *J* = 1.2 Hz, 1H), 5.33 (s, 2H), 2.42 (s, 3H). **^{13}C NMR (101 MHz, $CDCl_3$)** δ 138.8, 136.7, 128.9, 128.8, 127.6, 126.9, 125.9, 121.7, 119.1, 118.8, 110.9, 109.5, 49.8, 9.7. **HRMS (ESI-TOF)** calcd for $C_{16}H_{14}NBr$ $(M+H^{+})$: 300.0382; Found: 300.0374.

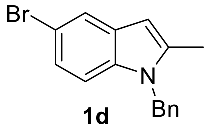

1d

1-benzyl-5-bromo-2-methyl-1*H*-indole (1d): white solid, m.p. = 85 – 88 °C, 85% yield. **^{1}H NMR (400 MHz, CDCl$_3$)** δ 7.71 (d, *J* = 2.0 Hz, 1H), 7.32 – 7.27 (m, 3H), 7.21 (dd, J_1 = 8.8 Hz , J_2 = 2.0 Hz, 1H), 7.09 (d, *J* = 8.8 Hz, 1H), 6.99 – 6.95 (m, 2H), 6.32 (s, 1H), 5.32 (s, 2H), 2.40 (s, 3H). **^{13}C NMR (101 MHz, CDCl$_3$)** δ 138.2, 137.4, 135.9, 129.9, 128.9, 127.5, 125.9, 123.6, 122.3, 112.8, 110.7, 100.2, 46.6, 12.8. **HRMS (ESI-TOF)** calcd for $C_{16}H_{14}NBr$ (M+H$^+$): 300.0382; Found: 300.0375.

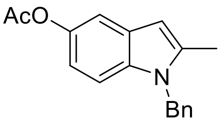

1e

1-benzyl-2-methyl-1*H*-indol-5-yl acetate (1e) : off-white solid, m.p. = 94 – 96 °C, 59% yield (for 3 steps). **^{1}H NMR (400 MHz, CDCl$_3$)** δ 7.32 – 7.27 (m, 4H), 7.18 (d, *J* = 8.8 Hz, 1H), 7.03 – 6.99 (m, 2H), 6.84 (dd, J_1 = 8.8 Hz, J_2 = 2.0 Hz, 1H), 6.34 (s, 1H), 5.32 (s, 2H), 2.40 (s, 3H), 2.35 (s, 3H). **^{13}C NMR (101 MHz, CDCl$_3$)** δ 170.5, 144.4, 138.1, 137.6, 135.1, 128.8, 128.4, 127.4, 126.0, 114.6, 111.8, 109.5, 100.8, 46.7, 21.3, 12.9. **HRMS (ESI-TOF)** calcd for $C_{18}H_{17}NO_2$ (M+H$^+$): 280.1332; Found: 280.1338.

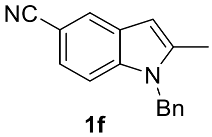

1f

1-benzyl-2-methyl-1*H*-indole-5-carbonitrile (1f): off-white solid, m.p. = 67 – 69 °C, 76 mg, 76% yield. **^{1}H NMR (400 MHz, CDCl$_3$)** δ 7.92 (s, 1H), 7.39 – 7.29 (m, 4H), 7.27 (d, *J* = 8.4 Hz, 1H), 7.01 – 6.93 (m, 2H), 6.45 (d, *J* = 1.2 Hz, 1H), 5.37 (s, 2H), 2.44 (s, 3H). **^{13}C NMR (101 MHz, CDCl$_3$)** δ 139.5, 138.8, 136.8, 129.0, 128.0, 127.8, 125.9, 125.2, 124.1, 121.1, 110.0, 102.6, 101.5, 46.8, 12.9.

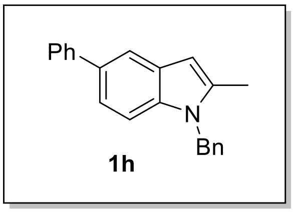

1-benzyl-2-methyl-5-phenyl-1*H*-indole (1h): off-white solid, m.p. = 149 – 151 °C, 75% yield. **^{1}H NMR (400 MHz, $CDCl_3$)** δ 7.83 (d, J = 1.2 Hz, 1H), 7.71 – 7.65 (m, 2H), 7.47 (t, J = 7.6 Hz, 2H), 7.41 (dd, J_1 = 8.4 Hz, J_2 = 1.6 Hz, 1H), 7.36 – 7.31 (m, 3H), 7.31 – 7.26 (m, 2H), 7.06 (d, J = 6.8 Hz, 2H), 6.43 (s, 1H), 5.38 (s, 2H), 2.44 (s, 3H). **^{13}C NMR (101 MHz, $CDCl_3$)** δ 142.7, 137.9, 137.5, 136.8, 133.1, 128.9, 128.7, 128.6, 127.4, 126.2, 126.1, 120.6, 118.4, 109.5, 100.9, 46.7, 12.9. **HRMS (ESI-TOF)** calcd for $C_{22}H_{19}N$ (M+H$^+$): 298.1590; Found: 298.1590.

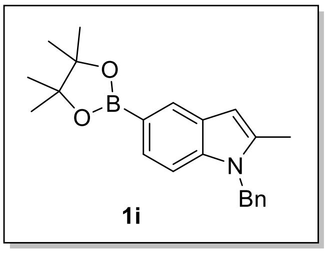

1-benzyl-2-methyl-5-(4,4,5,5-tetramethyl-1,3,2-dioxaborolan-2-yl)-1*H*-indole (1i): off-white solid, m.p. = 84 – 86 °C, 80% yield. **^{1}H NMR (400 MHz, $CDCl_3$)** δ 8.14 (s, 1H), 7.61 (dd, J_1 = 8.4 Hz, J_2 =12.0 Hz, 1H), 7.27 (m, 4H), 7.01 – 6.95 (m, 2H), 6.39 (s, 1H), 5.36 (s, 2H), 2.39 (s, 3H), 1.40 (s, 12H). **^{13}C NMR (101 MHz, $CDCl_3$)** δ 139.3, 137.8, 136.9, 128.8, 128.0, 127.6, 127.3, 127.1, 126.0, 108.7, 101.2, 83.4, 46.5, 25.0, 12.8. **HRMS (ESI-TOF)** calcd for $C_{22}H_{26}NO_2B$ (M+H$^+$): 348.2129; Found: 348.2120.

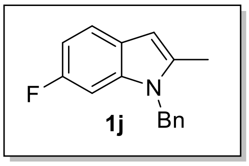

1-benzyl-6-fluoro-2-methyl-1*H*-indole (1j): off-white solid, m.p. = 65 – 67 °C, 87% yield. **^{1}H NMR (400 MHz, $CDCl_3$)** δ 7.54 – 7.40 (m, 1H), 7.38 – 7.19 (m, 3H), 6.98 (d, J = 6.4 Hz, 2H), 6.87 (m, 2H), 6.32 (s, 1H), 5.25 (s, 2H), 2.37 (s, 3H). **^{13}C NMR (101 MHz, $CDCl_3$)** δ 160.6, 158.2, 137.4, 137.2, 128.9, 127.5, 126.0, 124.6, 120.3, 120.2, 108.1, 107.9, 100.5, 96.1, 95.8, 46.7, 12.8. **^{19}F NMR (565 MHz, $CDCl_3$)** δ -122.23. **HRMS (ESI-TOF)** calcd for $C_{16}H_{14}NF$ (M+H$^+$): 240.1183; Found: 240.1188.

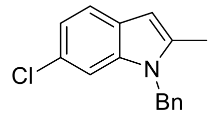

1k

1-benzyl-6-chloro-2-methyl-1*H*-indole (1k): off-white solid, m.p. = 63 – 65 °C, 85% yield. **^{1}H NMR (400 MHz, CDCl$_3$)** δ 7.72 (d, J = 2.0 Hz, 1H), 7.35 – 7.28 (m, 3H), 7.22 (dd, J_1 = 8.8 Hz, J_2 = 2.0 Hz, 1H), 7.10 (d, J = 8.8 Hz, 1H), 7.01 – 6.94 (m, 2H), 6.32 (s, 1H), 5.32 (s, 2H), 2.41 (s, 3H). **^{13}C NMR (101 MHz, CDCl$_3$)** δ 138.0, 136.7, 128.8, 127.6, 126.9, 125.9, 121.6, 119.1, 118.8, 110.9, 109.5, 49.8, 9.7. **HRMS (ESI-TOF)** calcd for $C_{16}H_{14}NCl$ (M+H$^+$): 256.0888; Found: 256.0885.

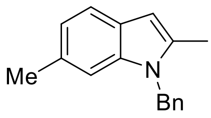

1l

1-benzyl-2,6-dimethyl-1*H*-indole (1l): off-white solid, m.p. = 57 – 59 °C, 90% yield. **^{1}H NMR (400 MHz, CDCl$_3$)** δ 7.34 (s, 1H), 7.28 – 7.19 (m, 3H), 7.08 (d, J = 8.4 Hz, 1H), 6.98 – 6.89 (m, 3H), 6.23 (s, 1H), 5.27 (s, 2H), 2.42 (s, 3H), 2.34 (s, 3H). **^{13}C NMR (101 MHz, CDCl$_3$)** δ 138.1, 136.8, 135.6, 128.8, 128.7, 128.5, 127.2, 126.0, 122.3, 119.6, 108.9, 100.0, 46.5, 21.5, 12.8. **HRMS (ESI-TOF)** calcd for $C_{17}H_{17}N$(M+H$^+$): 236.1434; Found: 236.1434.

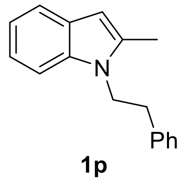

1p

2-methyl-1-phenethyl-1*H*-indole (1p): light yellow gel, 80% yield (for 2 steps). **^{1}H NMR (400 MHz, CDCl$_3$)** δ 7.66 (d, J = 7.6 Hz, 1H), 7.42 (d, J = 8.0 Hz, 1H), 7.36 (m, 3H), 7.30 – 7.25 (m, 1H), 7.21 (d, J = 7.6 Hz, 1H), 7.18 – 7.14 (m, 2H), 6.31 (s, 1H), 4.37 (t, J = 7.6 Hz, 2H), 3.12 (t, J = 7.6 Hz, 2H), 2.24 (s, 3H). **^{13}C NMR (101 MHz, CDCl$_3$)** δ 138.8, 136.7, 136.4, 129.0, 128.7, 128,4, 126.8, 120.6, 119.9, 119.5, 109.0, 100.0, 45.1, 36.5, 12.6. **RMS (ESI-TOF)** calcd for $C_{17}H_{17}N$ (M+H$^+$): 236.1434; Found: 236.1433.

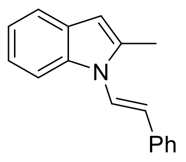

1r

(*E*)-2-methyl-1-styryl-1*H*-indole (1r): light yellow solid, m.p. = 65 – 67°C, 76 mg, 91% yield. **^{1}H NMR (400 MHz, $CDCl_3$)** δ 7.59 (d, *J* = 7.6 Hz, 1H), 7.30 – 7.06 (m, 6H), 7.00 – 6.92 (m, 2H), 6.80 (d, *J* = 8.8 Hz, 1H), 6.68 (d, *J* = 8.8 Hz, 1H), 6.42 (s, 1H), 2.27 (s, 3H). **^{13}C NMR (101 MHz, $CDCl_3$)** δ 136.4, 135.8, 134.6, 129.1, 128.8, 128.6, 128.5, 128.1, 122.8, 121.1, 120.2, 119.6, 110.7, 102.0, 13.1. **HRMS (ESI-TOF)** calcd for $C_{17}H_{15}N$ ($M+H^+$): 234.1277; Found: 234.1277.

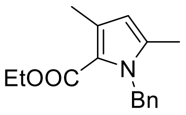

1t

ethyl 1-benzyl-3,5-dimethyl-1*H*-pyrrole-2-carboxylate (1t): off-white solid, m.p. = 43 – 45 °C, 85% yield. **^{1}H NMR (400 MHz, $CDCl_3$)** δ 7.25 (m, 2H), 7.20 – 7.13 (m, 1H), 6.95 – 6.85 (m, 2H), 5.85 (d, *J* = 1.6 Hz, 1H), 5.54 (d, *J* = 1.6 Hz, 2H), 4.18 (qd, J_1 = 7.2 Hz, J_2 = 2.8 Hz, 2H), 2.33 (d, *J* = 3.6 Hz, 3H), 2.12 (d, *J* = 1.6 Hz, 3H), 1.25 (td, J_1 = 7.2 Hz, J_2 = 2.8 Hz, 3H). **^{13}C NMR (101 MHz, $CDCl_3$)** δ 162.0, 138.9, 135.9, 130.1, 128.6, 126.8, 125.8, 118.9, 111.3, 59.4, 48.5, 14.6, 14.4, 12.4. **HRMS (ESI-TOF)** calcd for $C_{16}H_{19}NO_2$ ($M+H^+$): 258.1489; Found: 258.1491.

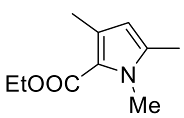

1u

ethyl 1,3,5-trimethyl-1*H*-pyrrole-2-carboxylate (1u): white solid, m.p. = 38 – 40 °C, 89% yield. **^{1}H NMR (400 MHz, $CDCl_3$)** δ 5.76 (s, 1H), 4.28 (q, *J* = 7.2 Hz, 2H), 3.76 (s, 3H), 2.29 (s, 3H), 2.19 (s, 3H), 1.35 (t, *J* = 7.2 Hz, 3H). **^{13}C NMR (101 MHz, $CDCl_3$)** δ 162.3, 135.5, 129.4, 119.1, 110.5, 59.3, 32.8, 14.6, 14.4, 12.5. **HRMS (ESI-TOF)** calcd for $C_{10}H_{15}NO_2$ ($M+H^+$): 182.1176; Found: 182.1175.

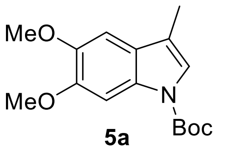

5a

***tert*-butyl 5,6-dimethoxy-3-methyl-1*H*-indole-1-carboxylate (5a):** light yellow gel, 89% yield. **^{1}H NMR (400 MHz, CDCl$_3$)** δ 7.76 (s, 1H), 7.22 (s, 1H), 6.91 (s, 1H), 3.95 (d, *J* = 3.2 Hz, 6H), 2.23 (d, *J* = 0.8 Hz, 3H), 1.65 (s, 9H). **^{13}C NMR (101 MHz, CDCl$_3$)** δ 149.9, 147.7, 146.2, 129.8, 124.0, 121.2, 116.2, 100.7, 99.0, 82.9, 56.2, 56.1, 28.3, 9.8. **HRMS (ESI-TOF)** calcd for $C_{16}H_{21}NO_4$ (M+H$^+$): 292.1543; Found: 292.1540.

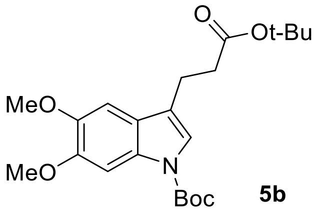

5b

***tert*-butyl 3-(3-(*tert*-butoxy)-3-oxopropyl)-5,6-dimethoxy-1*H*-indole-1-carboxylate (5b):** white solid, m.p. = 90 – 92 °C, 75% yield (for 2 steps). **^{1}H NMR (400 MHz, CDCl$_3$)** δ 7.80 (s, 1H), 7.26 (s, 1H), 6.98 (s, 1H), 3.99 (t, *J* = 6.8 Hz, 6H), 2.97 (t, *J* = 7.6 Hz, 2H), 2.79 – 2.48 (m, 2H), 1.68 (s, 9H), 1.49 (s, 9H). **^{13}C NMR (101 MHz, CDCl$_3$)** δ 172.5, 149.8, 147.8, 146.2, 129.8, 122.9, 120.7, 119.7, 100.6, 99.0, 83.1, 80.6, 56.3, 56.1, 35.0, 28.3, 28.2, 20.5. **HRMS (ESI-TOF)** calcd for $C_{22}H_{31}NO_6$ (M+H$^+$): 406.2224; Found: 406.2223.

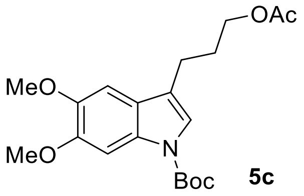

5c

***tert*-butyl 3-(3-acetoxypropyl)-5,6-dimethoxy-1*H*-indole-1-carboxylate (5c):** off-white solid, m.p. = 72 – 74 °C, 50% yield (for 4 steps). **^{1}H NMR (400 MHz, CDCl$_3$)** δ 7.76 (s, 1H), 7.23 (s, 1H), 6.91 (s, 1H), 4.16 (t, *J* = 6.4 Hz, 2H), 3.95 (d, *J* = 4.0 Hz, 6H), 2.72 (t, *J* = 7.6 Hz, 2H), 2.15 – 1.97 (m, 5H), 1.66 (s, 9H). **^{13}C NMR (101 MHz, CDCl$_3$)** δ 171.2, 149.8, 147.8, 146.2, 129.9, 123.0, 120.8, 119.8, 100.6, 99.1, 83.2, 64.1, 56.3, 56.1, 28.3, 28.1, 21.5, 21.1. **HRMS (ESI-TOF)** calcd for $C_{20}H_{27}NO_6$ (M+H$^+$): 378.1911; Found: 378.1906.

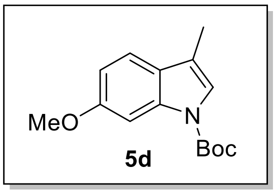

***tert*-butyl 6-methoxy-3-methyl-1*H*-indole-1-carboxylate (5d):** light yellow gel, 70% yield (for 2 steps). **^{1}H NMR (400 MHz, CDCl$_3$)** δ 7.77 (s, 1H), 7.38 (dd, J_1 = 8.4 Hz, J_2 =1.6 Hz, 1H), 7.27 (s, 1H), 6.91 (dt, J = 8.4, 2.0 Hz, 1H), 3.91 (s, 3H), 2.27 (s, 3H), 1.70 (s, 9H). **^{13}C NMR (101 MHz, CDCl$_3$)** δ 157.9, 149.9, 146.8, 125.3, 121.5, 119.4, 116.3, 111.6, 99.4, 85.2, 83.1, 55.7, 28.3, 27.5, 9.7. **HRMS (ESI-TOF)** calcd for $C_{15}H_{19}NO_3$ (M+H$^+$): 262.1438; Found: 262.1442.

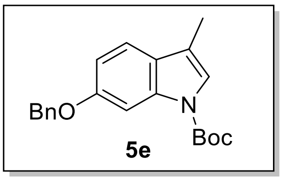

***tert*-butyl 6-(benzyloxy)-3-methyl-1*H*-indole-1-carboxylate (5e):** light yellow gel, 90% yield. **^{1}H NMR (400 MHz, CDCl$_3$)** δ 7.89 (s, 1H), 7.53 (d, J = 6.8 Hz, 2H), 7.42 (m, 4H), 7.30 (s, 1H), 7.01 (dd, J_1 = 8.4 Hz, J_2 = 2.4 Hz, 1H), 5.19 (s, 2H), 2.29 (s, 3H), 1.70 (s, 9H). **^{13}C NMR (101 MHz, CDCl$_3$)** δ 157.1, 149.9, 137.3, 128.6, 127.9, 127.6, 125.6, 121.7, 119.4, 116.4, 112.3, 100.8, 83.1, 70.5, 28.3, 9.7. **HRMS (ESI-TOF)** calcd for $C_{21}H_{23}NO_3$ (M+H$^+$): 338.1751; Found: 338.1750.

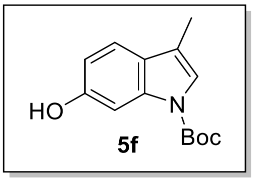

***tert*-butyl 6-hydroxy-3-methyl-1*H*-indole-1-carboxylate (5f):** colorless oil, 50% yield. **^{1}H NMR (400 MHz, CDCl$_3$)** δ 8.00 (s, 1H), 7.48 (d, J = 8.4 Hz, 1H), 7.38 (s, 1H), 7.09 (dd, J_1 = 8.4 Hz, J_2 = 2.0 Hz, 1H), 2.28 (s, 3H), 1.68 (s, 9H). **^{13}C NMR (101 MHz, CDCl$_3$)** δ 152.5, 149.6, 148.5, 129.3, 123.4, 119.2, 116.2,108.7, 83.4, 28.2, 9.6. **HRMS (ESI-TOF)** calcd for $C_{14}H_{17}NO_3$ (M+H$^+$): 248.1281; Found: 248.1277.

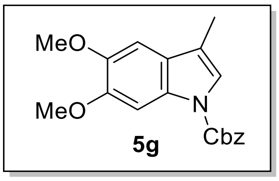

benzyl 5,6-dimethoxy-3-methyl-1*H*-indole-1-carboxylate (5g): white solid, m.p. = 87 – 89 °C, 70% yield. **^{1}H NMR (400 MHz, CDCl$_3$)** δ 7.54 – 7.49 (m, 2H), 7.48 – 7.39 (m, 4H), 7.31 (s, 1H), 6.95 (s, 1H), 5.44 (s, 2H), 3.98 (s, 6H), 2.26 (s, 3H). **^{13}C NMR (101 MHz, CDCl$_3$)** δ 150.9, 147.9, 146.5, 135.4, 128.8, 128.7, 128.4, 127.0, 124.1, 120.8, 117.2, 100.8, 99.1, 68.4, 56.3, 56.2, 9.9. **HRMS (ESI-TOF)** calcd for $C_{19}H_{19}NO_4$ (M+H$^+$): 326.1387; Found: 326.1386.

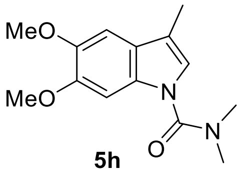

5h

5,6-dimethoxy-*N*,*N*,3-trimethyl-1*H*-indole-1-carboxamide (5h): white solid, m.p. = 95 – 97 °C, 86% yield. **^{1}H NMR (400 MHz, CDCl$_3$)** δ 7.38 (s, 1H), 6.97 (d, J = 1.2 Hz, 1H), 6.95 (s, 1H), 3.97 (d, J = 5.6 Hz, 6H), 3.13 (s, 6H), 2.29 (d, J = 1.2 Hz, 3H). **^{13}C NMR (101 MHz, CDCl$_3$)** δ 155.7, 147.7, 146.0, 130.5, 122.9, 121.7, 114.8, 100.6, 97.8, 56.3, 56.3, 38.6, 9.8. **HRMS (ESI-TOF)** calcd for $C_{14}H_{18}N_2O_3$ (M+H$^+$): 263.1390; Found: 263.1384.

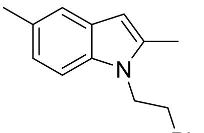

10a

2,5-dimethyl-1-phenethyl-1*H*-indole (10a): off-white solid, m.p. = 64 – 66 °C, 85% yield (for 2 steps). **^{1}H NMR (400 MHz, CDCl$_3$)** δ 7.41 (s, 1H), 7.38 – 7.28 (m, 4H), 7.16 – 7.11 (m, 2H), 7.07 (dd, J_1 = 8.4 Hz, J_2 = 1.6 Hz, 1H), 6.19 (s, 1H), 4.32 (t, J = 7.2 Hz, 2H), 3.09 (t, J = 7.2 Hz, 2H), 2.54 (s, 3H), 2.19 (s, 3H). **^{13}C NMR (101 MHz, CDCl$_3$)** δ 138.8, 136.7, 134.7, 129.0, 128.7, 128.6, 128.6, 126.7, 122.0, 119.7, 108.7, 99.4, 45.1, 36.5, 21.6, 12.5. **HRMS (ESI-TOF)** calcd for $C_{18}H_{19}N$ (M+H$^+$): 250.1590; Found: 250.1590.

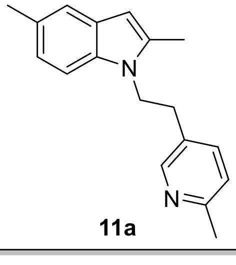

11a

2,5-dimethyl-1-(2-(6-methylpyridin-3-yl)ethyl)-1*H*-indole (11a): off-white solid, m.p. = 81 – 83 °C, 81% yield (for 2 steps). **^{1}H NMR (400 MHz, CDCl$_3$)** δ 8.31 (d, *J* = 2.4 Hz, 1H), 7.38 (s, 1H), 7.21 (d, *J* = 8.4 Hz, 1H), 7.09 – 6.97 (m, 3H), 6.16 (t, *J* = 1.2 Hz, 1H), 4.26 (t, *J* = 7.2 Hz, 2H), 3.02 (t, *J* = 7.2 Hz, 2H), 2.58 (s, 3H), 2.50 (s, 3H), 2.14 (s, 3H). **^{13}C NMR (101 MHz, CDCl$_3$)** δ 156.8, 149.3, 136.9, 136.4, 134.6, 131.0, 128.7, 128.6, 123.0, 122.1, 119.8, 108.6, 99.8, 44.6, 33.0, 24.1, 21.5, 12.6. **HRMS (ESI-TOF)** calcd for $C_{18}H_{20}N_2$ (M+H$^+$): 265.1699; Found: 265.1698.

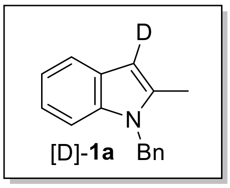

1-benzyl-2-methyl-1*H*-indole-3-*d* ([D]-**1a**)**:** brown solid, m.p. = 55 – 57 °C, 99% yield. **^{1}H NMR (400 MHz, CDCl$_3$)** δ 7.77 – 7.71 (m, 1H), 7.43 – 7.32 (m, 4H), 7.29 – 7.21 (m, 2H), 7.16 – 7.03 (m, 2H), 5.40 (s, 2H), 2.50 (s, 3H). **^{13}C NMR (101 MHz, CDCl$_3$)** δ 138.1, 137.4, 136.8, 128.9, 128.3, 127.4, 126.2, 120.9, 119.9, 119.7, 109.4, 46.6, 12.9. **HRMS (ESI-TOF)** calcd for $C_{16}H_{14}DN$ (M+H$^+$): 223.1340; Found: 223.1336.

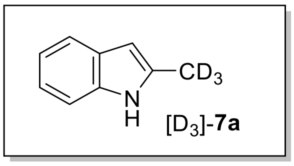

2-(methyl-*d*3)-1*H*-indole ([D$_3$]**-7a):** off-white solid, m.p. = 90 – 92 °C, 50% yield. **^{1}H NMR (400 MHz, CDCl$_3$)** δ 7.85 (s, 1H), 7.60 – 7.54 (m, 1H), 7.32 (dd, J_1 = 8.0 Hz, J_2 = 1.2 Hz, 1H), 7.19 – 7.09 (m, 2H), 6.27 (dd, J_1 = 2.0 Hz, J_2 = 0.8 Hz, 1H). **^{13}C NMR (101 MHz, CDCl$_3$)** δ 136.2, 135.3, 129.2, 121.0, 119.8, 110.5, 100.4.

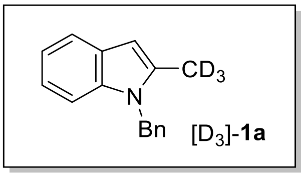

1-benzyl-2-(methyl-*d*3)-1*H*-indole ([D$_3$]**-1a):** light yellow solid, m.p. = 78 – 80 °C, 92% yield. **^{1}H NMR (400 MHz, CDCl$_3$)** δ 7.68 – 7.64 (m, 1H), 7.38 – 7.30 (m, 4H), 7.22 – 7.16 (m, 2H), 7.08 – 7.03 (m, 2H), 6.43 (s, 1H), 5.38 (s, 2H). **^{13}C NMR (101 MHz, CDCl$_3$)** δ 138.0, 137.3,

128.9, 128.4, 128.3, 127.4, 126.1, 120.9, 119.8, 119.6, 109.3, 100.6, 46.5. **HRMS (ESI-TOF)** calcd for $C_{16}H_{12}D_3N$ (M+H$^+$): 225.1466; Found: 225.1467.

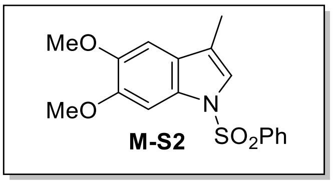

5,6-dimethoxy-3-methyl-1-(phenylsulfonyl)-1*H*-indole (M-S2): white solid, m.p. = 147 – 149 °C, 95% yield. **^{1}H NMR (400 MHz, CDCl$_3$)** δ 7.87 – 7.79 (m, 2H), 7.60 (s, 1H), 7.57 – 7.52 (m, 1H), 7.44 (dd, J_1= 8.4 Hz, J_2 = 7.2 Hz, 2H), 7.20 (d, J = 1.2 Hz, 1H), 6.87 (s, 1H), 4.01 (s, 3H), 3.94 (s, 3H), 2.23 (s, 3H). **^{13}C NMR (101 MHz, CDCl$_3$)** δ 148.2, 147.1, 138.3, 133.6, 129.6, 129.2, 126.6, 124.8, 121.8, 119.2, 101.0, 97.7, 56.5, 56.2, 9.9. **HRMS (ESI-TOF)** calcd for $C_{17}H_{17}NO_4S$ (M+H$^+$): 332.0951; Found: 332.0951.

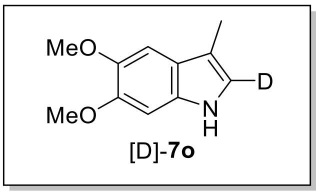

5,6-dimethoxy-3-methyl-1*H*-indole-2-*d* ([D]-7o): light yellow solid, m.p. = 138 – 140 °C, 70% yield (for 2 steps). **^{1}H NMR (400 MHz, CDCl$_3$)** δ 7.91 (s, 1H), 7.06 (s, 1H), 6.83 (d, J = 5.6 Hz, 1H), 4.00 (s, 3H), 3.91 (d, J = 2.4 Hz, 3H), 2.35 (s, 3H). **^{13}C NMR (101 MHz, CDCl$_3$)** δ 147.1, 144.8, 130.5, 121.1, 120.2, 111.4, 100.7, 94.6, 56.4, 56.3, 9.9. **HRMS (ESI-TOF)** calcd for $C_{11}H_{12}DNO_2$ (M+H$^+$): 193.1082; Found: 193.1085.

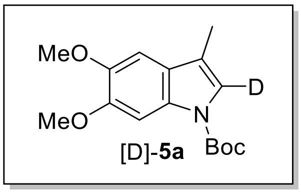

***tert*-butyl 5,6-dimethoxy-3-methyl-1*H*-indole-1-carboxylate-2-*d* ([D]-5a):** white solid, m.p. = 90 – 92 °C, 46 mg, 59% yield. **^{1}H NMR (400 MHz, CDCl$_3$)** δ 7.89 – 7.72 (m, 1H), 7.25 (s, 0.16H), 6.94 (s, 1H), 3.99 (d, J = 4.0 Hz, 6H), 2.26 (s, 3H), 1.68 (s, 9H). **^{13}C NMR (101 MHz, CDCl$_3$)** δ 149.9, 147.7, 146.2, 124.0, 121.3, 116.2, 116.1, 100.7, 99.1, 82.9, 56.2, 56.2, 28.3, 9.8. **HRMS (ESI-TOF)** calcd for $C_{16}H_{20}DNO_4$ (M+H$^+$): 293.1606; Found: 293.1601.

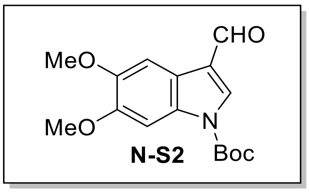

***tert*-butyl 3-formyl-5,6-dimethoxy-1*H*-indole-1-carboxylate (N-S2):** white solid, m.p. = 159 – 161 °C, 89% yield. **^{1}H NMR (400 MHz, CDCl$_3$)** δ 10.03 (s, 1H), 8.08 (s, 1H), 7.73 (m, 2H), 3.97 (m, 6H), 1.70 (s, 9H). **^{13}C NMR (101 MHz, CDCl$_3$)** δ 186.1, 148.9, 148.8, 147.8, 134.9, 130.3, 121.6, 118.8, 103.1, 98.4, 85.4, 56.2, 56.1, 28.1. **HRMS (ESI-TOF)** calcd for $C_{16}H_{19}NO_5$ (M+H$^+$): 306.1336; Found: 306.1334.

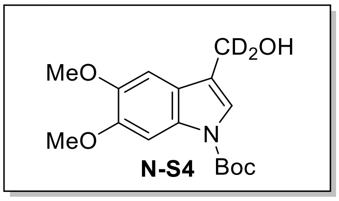

***tert*-butyl 3-(hydroxymethyl-*d*$_2$)-5,6-dimethoxy-1*H*-indole-1-carboxylate (N-S4):** colorless gel, 30% yield (for 2 steps). **^{1}H NMR (400 MHz, CDCl$_3$)** δ 7.76 (s, 1H), 7.43 (s, 1H), 7.08 (s, 1H), 3.94 (m, 6H), 1.65 (s, 9H). **^{13}C NMR (101 MHz, CDCl$_3$)** δ 149.8, 148.0, 146.5, 130.1, 122.2, 121.8, 120.2, 100.9, 99.0, 83.5, 56.2, 56.1, 28.2. **HRMS (ESI-TOF)** calcd for $C_{16}H_{19}D_2NO_5$ (M+H$^+$): 310.1618; Found: 310.1613.

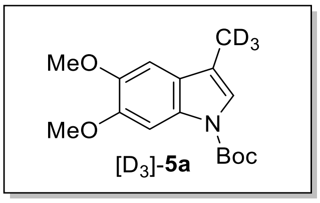

***tert*-butyl 5,6-dimethoxy-3-(methyl-*d*$_3$)-1*H*-indole-1-carboxylate (**[D$_3$]-**5a):** white solid, m.p. = 90 – 92 °C, 60% yield (for 2 steps). **^{1}H NMR (400 MHz, CDCl$_3$)** δ 7.76 (s, 1H), 7.22 (s, 1H), 6.91 (s, 1H), 3.95 (m, 6H), 1.65 (s, 9H). **^{13}C NMR (101 MHz, CDCl$_3$)** δ 149.9, 147.7, 146.2, 129.8, 124.0, 121.3, 116.1, 100.7, 99.0, 82.9, 56.2, 56.1, 28.3. **HRMS (ESI-TOF)** calcd for $C_{16}H_{18}D_3NO_4$ (M+H$^+$): 295.1732; Found:295.1729.

3. Optimization of the Reaction Conditions

3.1 Reaction optimization for the multicomponent synthesis of γ-tetrahydrocarboline

Supplementary Table 1. Reaction Optimization for the multicomponent synthesis of γ-tetrahydrocarboline[a]

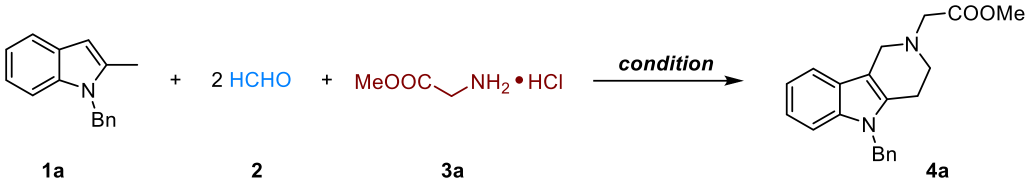

Entry	2 (equiv.)	3a (equiv.)	Solvent	Temp.	Time	Yield (%)[b]
**1**	5	2	DMSO (1 mL)	r. t.	12 hr	84
**2**	5	2	DMF (1 mL)	r. t.	12 hr	92
**3**	5	2	THF (1 mL)	r. t.	12 hr	87
**4**	5	2	MeCN (1 mL)	r. t.	1.5 hr	25
**5**	5[c]	2	MeCN (1 mL)	45 °C	6 hr	trace
**6**	5	2	MeOH (1 mL)	r. t.	12 hr	12
**7**	5	2	DMF (1 mL)	r. t. to 60 °C	2 hr	90
**8**	5	2	DMF/MeCN (1 mL, 1:1)	r. t. to 45 °C	2 hr	90

[a]The reaction was conducted with **1a** (0.15 mmol, 1 equiv.), formaldehyde **2** (37% in water, 0.75 mmol, 60 μL, 5 equiv.), **3a** (0.3 mmol, 2 equiv.).

[b]Yield refers to isolated product by column chromatography on silica gel eluted with petroleum ether/ethyl acetate (v/v, 4:1). [c]Polyformaldehyde was used as formaldehyde source.

To screen the *N*-substitution groups, we chosen the model substrates of indole derivatives **1**, formaldehyde **2**, and methyl glycine ester hydrochloride **3a**.

Supplementary Table 2. Screen the *N*-substitutions for the multicomponent synthesis of γ-tetrahydrocarboline*

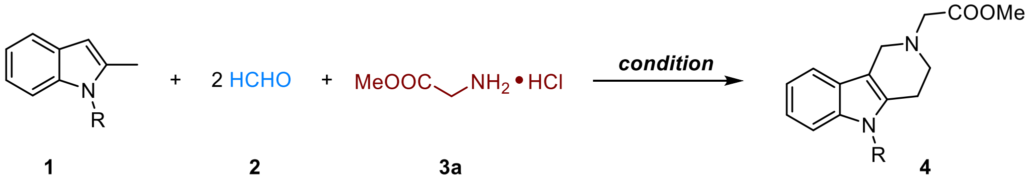

Entry	R	Solvent	Temp.	Time	Yield (%)
**1**	Bn	DMF (1 mL)	r. t. to 60 °C	2 hr	90
**2**	Boc	DMF (1 mL)	r. t. to 60 °C	12 hr	trace
**3**	Boc	THF (1 mL)	r. t. to 60 °C	36 hr	30
**4**	Boc	MeCN (1 mL)	r. t. to 60 °C	18 hr	36

**5**	Ts	MeCN (1 mL)	r. t. to 60 ºC	12 hr	trace
**6**	Cbz	MeCN (1 mL)	r. t. to 60 ºC	36 hr	25
**7**	Ac	MeCN (1 mL)	r. t. to 60 ºC	12 hr	trace
**8**	Me	DMF (1 mL)	r. t. to 60 ºC	2 hr	87
**9**	H	MeCN (1 mL)	r. t. to 60 ºC	2 hr	N/A
**10**	H	DMF (1 mL)	r. t. to 60 ºC	2 hr	N/A

*The reactions conducted with **1** (0.15 mmol, 1 equiv.), formaldehyde **2** (37% in water, 0.75 mmol, 60 μL, 5 equiv.), **3a** (0.3 mmol, 2 equiv.) and yield refers to isolated product by column chromatography on silica gel eluted with petroleum ether/ethyl acetate (v/v, 4:1); "N/A" = Not Available.

3.2 Real-time monitoring test of γ-tetrahydrocarboline

To detect the content change of reaction components, A mixture of **1** (0.2 mmol), formaldehyde **2** (37% in water, 0.08 mL, 5 equiv.) and **3a** (0.4 mmol, 2 equiv.) in DMF (1.5 mL) was stirred at room temperature for 15 minutes. Then the reaction was heated to 60 ºC until the reaction was completed. The content of intermediates and product was detected at 15 min, 30 min, 45 min, 60 min, 90 min, 120 min, 150 min and 180 min.

Supplementary Table 3. Real-time monitoring test for the multicomponent synthesis of **4a**

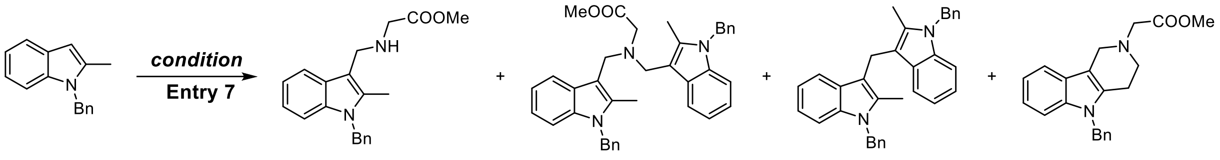

Time (min)	Temp.	18 (yield, %)	19 (yield, %)	21 (yield, %)	4a (yield, %)
**15**	r. t.	60	10	5	0
**30**	60 ºC	35	5	10	32
**45**	60 ºC	31	trace	trace	64
**60**	60 ºC	24	trace	trace	70
**90**	60 ºC	11	-	-	82
**120**	60 ºC	trace	-	-	87
**150**	60 ºC	-	-	-	90
**180**	60 ºC	-	-	-	92

The intermediates **18**, **19** and **21** were detected in the progress[13]. Guided by the previous studies[11-12], The byproduct **21** was formed from coupling of indoles and formaldehyde, which was catalysed by acid.

3.3 Reaction optimization for the multicomponent synthesis of β-tetrahydrocarboline

Supplementary Table 4. Reaction Optimization for the multicomponent synthesis of dimethoxy β-tetrahydrocarboline*

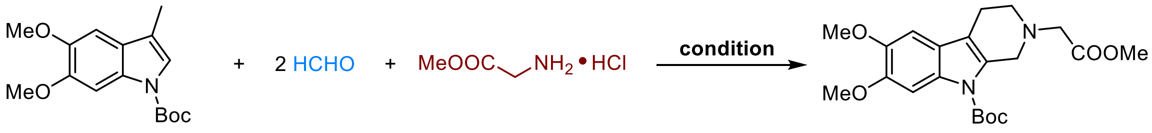

Entry	2 (equiv.)	3a (equiv.)	Solvent	Temp.	Time	Yield (%)
**1**	5	2	MeCN (1 mL)	r. t. to 60 ºC	12 hr	72
**2**	5	2	DMF (1 mL)	r. t. to 60 ºC	12 hr	73
**3**	5	2	DMF/MeCN (1 mL, 1:1)	r. t. to 60 ºC	12 hr	80
**4**	5	2	MeCN (1 mL)	r. t. to 80 ºC	6 hr	80
**5**	5[a]	2	MeCN (1 mL)	r. t. to 60 ºC	12 hr	46

*The reactions conducted with **5a** (0.15 mmol, 1 equiv.), formaldehyde **2** (37% in water, 0.75 mmol, 60 µL, 5 equiv.), **3a** (0.3 mmol, 2 equiv.) and yield refers to isolated product by column chromatography on silica gel eluted with petroleum ether/ethyl acetate (v/v, 2:1); ([a]): paraformaldehyde was used as formaldehyde source.

Supplementary Table 5. Reaction optimization for the multicomponent synthesis of methoxy β-tetrahydrocarboline*

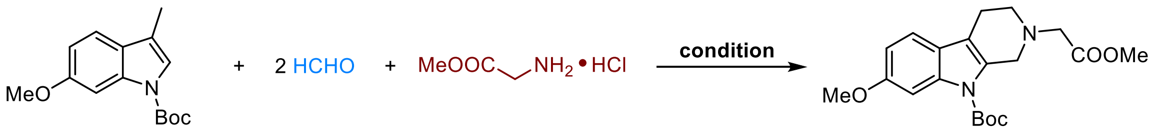

Entry	2 (equiv.)	3a (equiv.)	*p*-TSA (equiv.)	Solvent	Temp.	Time	Yield (%)
**1**	5	2	0	MeCN (1 mL)	r. t. to 80 ºC	8 hr	40
**2**	5	2	0.2	MeCN (1 mL)	r. t. to 80 ºC	16 hr	50
**3**	5	2	0.5	MeCN (1 mL)	r. t. to 60 ºC	8 hr	56
**4**	5	2	1	MeCN (1 mL)	r. t. to 60 ºC	8 hr	52

**5**	5	2	0.5	MeCN (1 mL)	r. t. to 80 °C	8 hr	45
**6**	5	2	0.5	MeCN (1 mL)	r. t. to 50 °C	16 hr	50

*The reactions conducted with **5a** (0.15 mmol, 1 equiv.), formaldehyde **2** (37% in water, 0.75 mmol, 60 μL, 5 equiv.), **3a** (0.3 mmol, 2 equiv.), *p*-toluenesulfonic acid (*p*-TSA) and yield refers to isolated product by column chromatography on silica gel eluted with petroleum ether/ethyl acetate (v/v, 2:1).

To screen the substituted groups, we used the model substrates of indole derivatives **4**, formaldehyde **2**, and methyl glycine ester hydrochloride **3a**.

Supplementary Table 6. Screen the substituents for the multicomponent synthesis of β-tetrahydrocarboline*

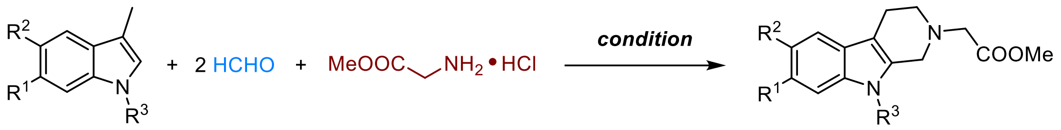

4 2 3a 6

Entry	R^1	R^2	R^3	Solvent	Temp.	Time	Yield (%)
**1**	OMe	OMe	Bn	MeCN (1 mL)	r. t. to 60 °C	2 hr	N/A
**2**	OMe	OMe	Me	MeCN (1 mL)	r. t. to 60 °C	2 hr	N/A
**3**	OMe	OMe	Boc	MeCN (1 mL)	r. t. to 80 °C	6 hr	80
**4**	OMe	OMe	Cbz	MeCN (1 mL)	r. t. to 80 °C	6 hr	65
**5**	OMe	OMe	H	MeCN (1 mL)	r. t. to 60 °C	2 hr	N/A
**6**	OMe	H	Boc	MeCN (1 mL)	r. t. to 80 °C	8 hr	40
**7**	OMe	H	Bn	DMF (1 mL)	r. t. to 60 °C	2 hr	trace
**8**[a]	OMe	H	Boc	MeCN (1 mL)	r. t. to 60 °C	8 hr	56
**9**	H	OMe	Bn	DMF (1 mL)	r. t. to 60 °C	2 hr	N/A
**10**	H	H	Me	MeCN (1 mL)	r. t. to 60 °C	8 hr	N/A

*The reactions conducted with **4** (0.15 mmol, 1 equiv.), formaldehyde **2** (37% in water, 0.75 mmol, 60 μL, 5 equiv.), **3a** (0.3 mmol, 2 equiv.) and yield refers to isolated product by column chromatography on silica gel eluted with petroleum ether/ethyl acetate (v/v, 2:1); [a]TsOH (0.075 mmol, 0.5 equiv.) was added as an additive; "N/A" = Not Available.

According to the results, electron-rich groups like methoxy were important for 3-substituted indole. Indoles with dimethoxy substituents could give the desired product in high yield. In addition, the *N*-substituents affect the reaction significantly. The electron-donating groups, such as methyl and benzyl, increased the instability of indole. On the contrary, the electron-

withdrawing groups, especially weak electron-withdrawing groups, not only improved the stability, but also contributed to the formation of β-tetrahydrocarboline.

3.4 Real-time monitoring test

To detect the content change of reaction components, a mixture of **5a** (0.2 mmol), formaldehyde **2** (37% in water, 0.08 mL, 5 equiv.) and **3a** (0.4 mmol, 2 equiv.) in MeCN (1.5 mL) was stirred at room temperature for 15 minutes. Then the reaction was heated to 80 °C until the reaction was completed. The content of intermediates and product was detected at 15 min, 30 min, 60 min, 90 min, 120 min, 180 min, 240 min and 360 min.

Supplementary Table 7. Real-time monitoring test for the multicomponent synthesis of **4a**

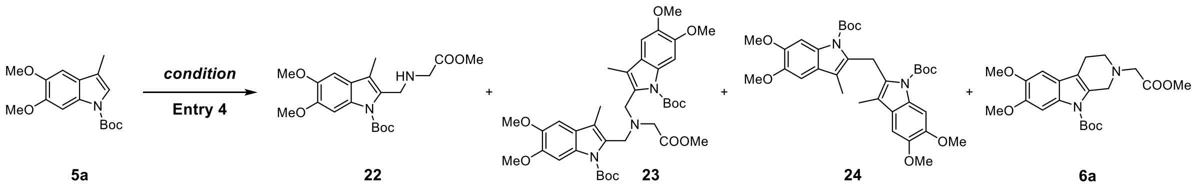

Time (min)	Temp.	22 (yield, %)	23 (yield, %)	24 (yield, %)	6a (yield, %)
**15**	r. t.	95	-	trace	0
**30**	80 °C	65	10	10	trace
**60**	80 °C	45	7	trace	42
**90**	80 °C	25	trace	-	60
**120**	80 °C	12	-	-	72
**180**	80 °C	trace	-	-	75
**240**	80 °C	trace	-	-	78
**360**	80 °C	trace	-	-	78

The intermediates **22**, **23** and **24** were detected in the progress[13]. Guided by the previous studies[11-12], The byproduct **24** was formed from coupling of indoles and formaldehyde, which was catalysed by acid.

4. Procedures of the Indole Alkaloids Synthesis and Characterization

4.1 General procedure for the synthesis of 4a-4aac

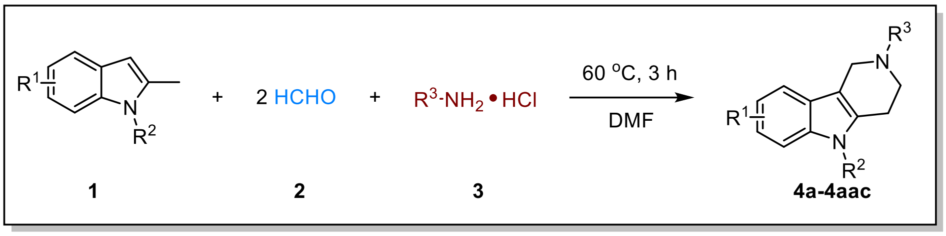

A mixture of **1** (0.2 mmol), formaldehyde **2** (37% in water, 0.08 mL, 5 equiv.) and corresponding primary amine hydrochloride **3** (0.4 mmol, 2 equiv.) in DMF (1.5 mL) was stirred at 60 °C until the reaction was completed. The reaction was quenched by saturated aqueous $NaHCO_3$. The aqueous layer was extracted with ethyl acetate (three times), and the combined organic layer was dried over Na_2SO_4 and concentrated. Purification by silica gel column chromatography to give corresponding indole alkaloids products **4a-4aac**.

4.2 General procedure for the synthesis of 4aad and 4aae

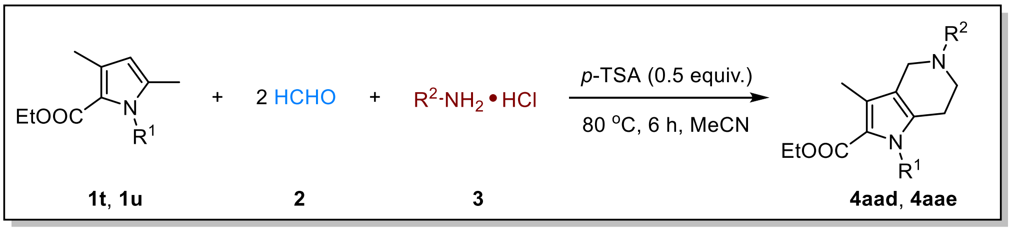

A mixture of **1** (0.2 mmol), formaldehyde **2** (37% in water, 0.08 mL, 5 equiv.), TsOH (0.1 mmol, 0.5 equiv.) and corresponding primary amine hydrochloride **3** (0.4 mmol, 2 equiv.) in MeCN (1.5 mL) was stirred at 80 °C until the reaction was completed. The reaction was quenched by saturated aqueous $NaHCO_3$. The aqueous layer was extracted with ethyl acetate (three times), and the combined organic layer was dried over Na_2SO_4 and concentrated. Purification by silica gel column chromatography to give corresponding indole alkaloids products **4aad** and **4aae**.

4.3 General procedure for the synthesis of 6a-6p

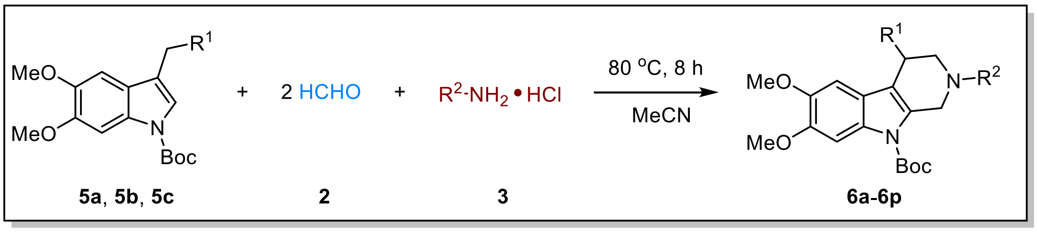

A mixture of **5** (0.2 mmol), formaldehyde **2** (37% in water, 0.08 mL, 5 equiv.) and corresponding primary amine hydrochloride **3** (0.4 mmol, 2 equiv.) in MeCN (1.5 mL) was stirred at 80 °C until the reaction was completed. The reaction was quenched by saturated aqueous $NaHCO_3$. The aqueous layer was extracted with ethyl acetate (three times), and the combined organic layer was dried over Na_2SO_4 and concentrated. Purification by silica gel column chromatography to give corresponding indole alkaloids products **6a-6p**.

4.4 General procedure for the synthesis of 6q-6ah

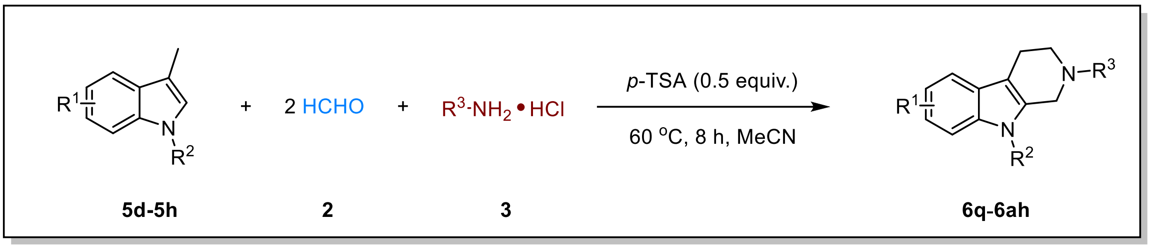

A mixture of **5** (0.2 mmol), formaldehyde **2** (37% in water, 0.08 mL, 5 equiv.), TsOH (0.1 mmol, 0.5 equiv.) and corresponding primary amine hydrochloride **3** (0.4 mmol, 2 equiv.) in MeCN (1.5 mL) was stirred at 60 °C until the reaction was completed. The reaction was quenched by saturated aqueous $NaHCO_3$. The aqueous layer was extracted with ethyl acetate (three times), and the combined organic layer was dried over Na_2SO_4 and concentrated. Purification by silica gel column chromatography to give corresponding indole alkaloids products **6q-6ah**.

4.4 Characterization of products 4 and 6

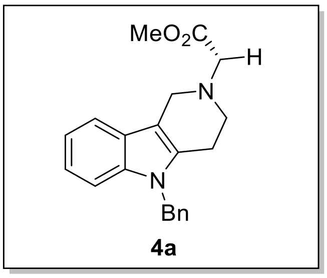

methyl 2-(5-benzyl-1,3,4,5-tetrahydro-2*H*-pyrido[4,3-*b*]indol-2-yl)acetate (4a) : According to the general procedure for 3 h. yellow gel, 61 mg, 92% yield. **^{1}H NMR (400 MHz, $CDCl_3$)** δ 7.48 (d, *J* = 7.2 Hz, 1H), 7.36 – 7.22 (m, 4H), 7.21 – 7.09 (m, 2H), 7.05 (d, *J* = 6.8 Hz, 2H), 5.29 (s, 2H), 3.98 (s, 2H), 3.81 (s, 3H), 3.58 (s, 2H), 3.08 (t, *J* = 5.6 Hz, 2H), 2.84 (t, *J* = 5.6 Hz, 2H). **^{13}C NMR (101 MHz, $CDCl_3$)** δ 171.2, 137.9, 136.8, 133.3, 128.8, 127.4, 126.2, 125.8, 121.1, 119.2, 117.7, 109.3, 107.9, 58.6, 51.8, 50.3, 49.5, 46.4, 22.6. **HRMS (ESI-TOF)** calcd for $C_{21}H_{22}N_2O_2$ (M+H$^+$): 335.1754; Found: 335.1757.

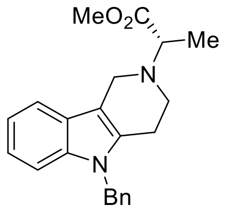

4b

methyl (*S*)-2-(5-benzyl-1,3,4,5-tetrahydro-2*H*-pyrido[4,3-*b*]indol-2-yl)propanoate (4b): According to the general procedure for 3 h, light yellow gel, 56 mg, 81% yield. **^{1}H NMR (400 MHz, CDCl$_3$)** δ 7.44 (d, *J* = 7.2 Hz, 1H), 7.28 – 7.16 (m, 4H), 7.14 – 7.04 (m, 2H), 7.01 (d, *J* = 7.2 Hz, 2H), 5.22 (s, 2H), 3.94 (s, 2H), 3.73 (s, 3H), 3.64 (q, *J* = 7.2 Hz, 1H), 3.08 (dt, *J* = 11.6, 5.6 Hz, 1H), 2.94 (dt, *J* = 11.6, 5.6 Hz, 1H), 2.76 (s, 2H), 1.45 (d, *J* = 7.2 Hz, 3H). **^{13}C NMR (101 MHz, CDCl$_3$)** δ 173.8, 137.9, 136.9, 133.7, 128.8, 127.3, 126.3, 125.9, 121.0, 119.1, 117.7, 109.3, 108.3, 62.2, 51.6, 47.0, 46.5, 46.1, 23.6, 15.3. **HRMS (ESI-TOF)** calcd for $C_{22}H_{24}N_2O_2$ (M+H$^+$): 349.1911; Found:349.1911.

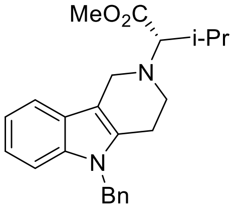

4c

methyl (*S*)-2-(5-benzyl-1,3,4,5-tetrahydro-2*H*-pyrido[4,3-*b*]indol-2-yl)-3-methylbutanoate (4c): According to the general procedure for 3 h, yellow gel, 64 mg, 85% yield. **^{1}H NMR (400 MHz, CDCl$_3$)** δ 7.47 – 7.38 (m, 1H), 7.22 (m, 4H), 7.13 – 7.03 (m, 2H), 7.00 (d, *J* = 7.2 Hz, 2H), 5.21 (s, 2H), 3.87 (s, 2H), 3.71 (s, 3H), 3.19 – 3.00 (m, 2H), 2.75 (m, 3H), 2.22 (m, 1H), 1.01 (d, *J* = 6.4 Hz, 3H), 0.93 (d, *J* = 6.4 Hz, 3H). **^{13}C NMR (101 MHz, CDCl$_3$)** δ 172.6, 138.0, 136.8, 134.1, 128.8, 127.3, 126.3, 125.8, 121.0, 119.2, 117.7, 109.3, 108.8, 74.3, 50.9, 46.7, 46.5, 46.3, 27.4, 23.9, 20.0, 19.5. **HRMS (ESI-TOF)** calcd for $C_{24}H_{28}N_2O_2$ (M+H$^+$):377.2224; Found:377.2229.

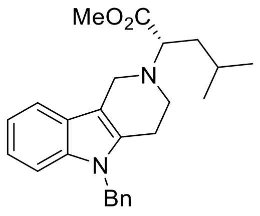

4d

methyl (*S*)-2-(5-benzyl-1,3,4,5-tetrahydro-2*H*-pyrido[4,3-*b*]indol-2-yl)-4-methylpentanoate (4d): According to the general procedure for 3 h, yellow gel, 62 mg, 79% yield. **^{1}H NMR (400 MHz, $CDCl_3$)** δ 7.44 (d, *J* = 7.2 Hz, 1H), 7.31 – 7.16 (m, 4H), 7.15 – 7.03 (m, 2H), 7.00 (d, *J* = 7.2 Hz, 2H), 5.21 (s, 2H), 3.93 (s, 2H), 3.71 (s, 3H), 3.65 – 3.52 (m, 1H), 3.19 – 3.05 (m, 1H), 2.99 – 2.85 (m, 1H), 2.72 (d, *J* = 4.8 Hz, 2H), 1.84 – 1.73 (m, 1H), 1.73 – 1.61 (m, 2H), 0.94 (dd, J_1 = 9.6 Hz, J_2 = 6.0 Hz, 6H). **^{13}C NMR (101 MHz, $CDCl_3$)** δ 173.3, 138.0, 136.8, 133.9, 128.8, 127.3, 126.3, 125.8, 121.0, 119.2, 117.7, 109.3, 108.6, 65.4, 51.2, 46.8, 46.5, 46.3, 38.7, 25.2, 23.9, 22.8, 22.7. **HRMS (ESI-TOF)** calcd for $C_{25}H_{30}N_2O_2$ (M+H$^+$): 391.2380; Found: 391.2375.

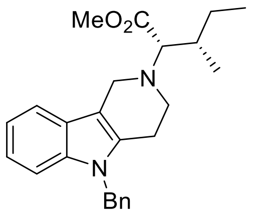

4e

methyl (*2S,3S*)-2-(5-benzyl-1,3,4,5-tetrahydro-2*H*-pyrido[4,3-*b*]indol-2-yl)-3-methylpentanoate (4e): According to the general procedure for 3 h, light yellow gel, 60 mg, 77% yield. **^{1}H NMR (400 MHz, $CDCl_3$)** δ 7.51 (d, *J* = 7.6 Hz, 1H), 7.29 (m, 4H), 7.20 – 7.10 (m, 2H), 7.07 (d, *J* = 7.2 Hz, 2H), 5.29 (s, 2H), 3.99 – 3.86 (m, 2H), 3.78 (s, 3H), 3.24 (d, *J* = 10.4 Hz, 1H), 3.21 – 3.11 (m, 1H), 2.90 – 2.82 (m, 1H), 2.78 (t, *J* = 4.8 Hz, 2H), 2.12 (m, 1H), 1.81 (m, 1H), 1.22 (m, 1H), 0.95 (m, 6H). **^{13}C NMR (101 MHz, $CDCl_3$)** δ 172.5, 138.0, 136.8, 134.1, 128.8, 127.3, 126.2, 125.8, 121.0, 119.2, 117.6, 109.3, 108.8, 72.5, 50.8, 46.6, 46.6, 46.5, 33.1, 25.1, 23.8, 16.1, 10.6. **HRMS (ESI-TOF)** calcd for $C_{25}H_{30}N_2O_2$ (M+H$^+$): 391.2380; Found: 391.2377.

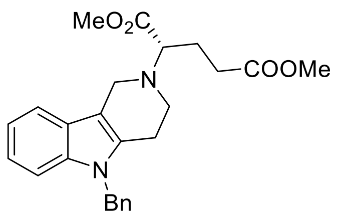

4f

dimethyl (*S*)-2-(5-benzyl-1,3,4,5-tetrahydro-2*H*-pyrido[4,3-*b*]indol-2-yl)pentanedioate (4f): According to the general procedure for 3 h, light yellow solid, m.p. = 78 – 80 °C, 76 mg, 91% yield. **^{1}H NMR (400 MHz, $CDCl_3$)** δ 7.55 – 7.43 (m, 1H), 7.29 (m, 4H), 7.15 (m, 2H), 7.06 (d, J = 6.8 Hz, 2H), 5.28 (s, 2H), 3.97 (dd, J_1 = 70.4 Hz, J_2 = 13.2 Hz, 2H), 3.78 (s, 3H), 3.65 – 3.55 (m, 4H), 3.27 – 3.14 (m, 1H), 2.92 (m, 1H), 2.77 (s, 2H), 2.50 (t, J = 7.2 Hz, 2H), 2.34 – 2.07 (m, 2H). **^{13}C NMR (101 MHz, $CDCl_3$)** δ 173.7, 172.5, 138.0, 136.8, 133.8, 128.8, 127.4, 126.3, 125.7, 121.0, 119.2, 117.6, 109.3, 108.5, 66.2, 51.6, 51.4, 46.9, 46.4, 46.2, 30.8, 24.5, 23.7. **HRMS (ESI-TOF)** calcd for $C_{25}H_{28}N_2O_4$ (M+H$^+$): 421.2122; Found: 421.2119.

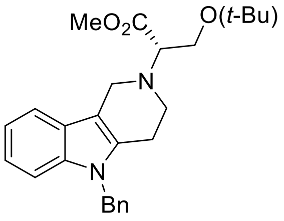

4g

methyl (*S*)-2-(5-benzyl-1,3,4,5-tetrahydro-2*H*-pyrido[4,3-*b*]indol-2-yl)-3-(*tert*-butoxy)propanoate (4g): According to the general procedure for 3 h, yellow gel, 71 mg, 84% yield. **^{1}H NMR (400 MHz, $CDCl_3$)** δ 7.44 (d, J = 7.2 Hz, 1H), 7.22 (m, 4H), 7.14 – 7.04 (m, 2H), 7.01 (d, J = 7.2 Hz, 2H), 5.22 (s, 2H), 3.97 (q, J = 13.6 Hz, 2H), 3.88 (t, J = 8.0 Hz, 1H), 3.77 – 3.64 (m, 5H), 3.16 – 2.97 (m, 2H), 2.75 (s, 2H), 1.19 (s, 9H). **^{13}C NMR (101 MHz, $CDCl_3$)** δ 171.8, 137.9, 136.8, 133.7, 128.8, 127.3, 126.3, 125.8, 121.0, 119.1, 117.7, 109.3, 108.4, 73.4, 67.8, 61.2, 51.4, 48.0, 47.1, 46.4, 27.5, 23.6. **HRMS (ESI-TOF)** calcd for $C_{26}H_{32}N_2O_3$ (M+H$^+$): 421.2486; Found: 421.2486.

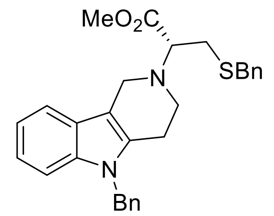

4h

methyl (*R*)-2-(5-benzyl-1,3,4,5-tetrahydro-2*H*-pyrido[4,3-*b*]indol-2-yl)-3-(benzylthio)propanoate (4h): According to the general procedure for 3 h, light yellow gel, 70 mg, 75% yield. **^{1}H NMR (400 MHz, CDCl$_3$)** δ 7.54 – 7.45 (m, 1H), 7.41 – 7.23 (m, 9H), 7.21 – 7.10 (m, 2H), 7.06 (d, J = 6.8 Hz, 2H), 5.27 (s, 2H), 3.94 (dd, J_1 = 42.0 Hz, J_2 = 13.2 Hz, 2H), 3.81 (d, J = 6.4 Hz, 5H), 3.70 (m, 1H), 3.11 (m, 1H), 3.02 (m, 1H), 2.97 – 2.86 (m, 2H), 2.78 (d, J = 4.4 Hz, 2H). **^{13}C NMR (101 MHz, CDCl$_3$)** δ 171.5, 138.3, 137.9, 136.8, 133.7, 129.0, 128.8, 128.6, 127.4, 127.2, 126.3, 125.7, 121.1, 119.3, 117.7, 109.3, 108.2, 67.6, 51.6, 47.3, 46.5, 46.3, 36.9, 30.7, 23.7. **HRMS (ESI-TOF)** calcd for $C_{29}H_{30}N_2O_2S$ (M+H$^+$): 471.2101; Found: 471.2096.

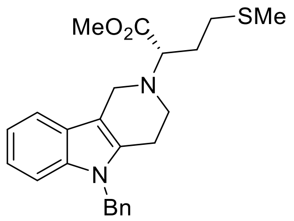

4i

methyl (*S*)-2-(5-benzyl-1,3,4,5-tetrahydro-2*H*-pyrido[4,3-*b*]indol-2-yl)-4-(methylthio)butanoate (4i): According to the general procedure for 3 h, light yellow gel, 59 mg, 72% yield. **^{1}H NMR (400 MHz, CDCl$_3$)** δ 7.44 (d, J = 7.2 Hz, 1H), 7.23 (m, 4H), 7.14 – 7.04 (m, 2H), 7.00 (d, J = 7.2 Hz, 2H), 5.22 (s, 2H), 3.93 (m, 2H), 3.77 – 3.62 (m, 4H), 3.21 – 3.06 (m, 1H), 2.91 (dt, J_1 = 11.6 Hz, J_2 =5.6 Hz, 1H), 2.81 – 2.66 (m, 2H), 2.59 (t, J = 7.2 Hz, 2H), 2.22 – 1.99 (m, 5H). **^{13}C NMR (101 MHz, CDCl$_3$)** δ 172.7, 137.9, 136.8, 133.8, 128.8, 127.4, 126.3, 125.8, 121.1, 119.2, 117.6, 109.3, 108.5, 65.7, 51.4, 47.0, 46.5, 46.1, 31.0, 29.0, 23.9, 15.6. **HRMS (ESI-TOF)** calcd for $C_{24}H_{28}N_2O_2S$ (M+H$^+$): 409.1944; Found: 409.1942.

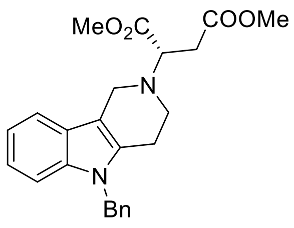

4j

dimethyl (*S*)-2-(5-benzyl-1,3,4,5-tetrahydro-2*H*-pyrido[4,3-*b*]indol-2-yl)succinate (4j): According to the general procedure for 3 h, light yellow gel, 56 mg, 69% yield. **^{1}H NMR (400 MHz, CDCl$_3$)** δ 7.49 (d, *J* = 6.8 Hz, 1H), 7.36 – 7.23 (m, 4H), 7.21 – 7.09 (m, 2H), 7.05 (d, *J* = 6.8 Hz, 2H), 5.27 (s, 2H), 4.21 – 4.04 (m, 2H), 3.88 (d, *J* = 13.2 Hz, 1H), 3.81 (s, 3H), 3.72 (s, 3H), 3.13 (m, 2H), 2.96 (m, 1H), 2.89 – 2.67 (m, 3H). **^{13}C NMR (101 MHz, CDCl$_3$)** δ 171.9, 171.6, 137.9, 136.8, 133.6, 128.8, 127.4, 126.3, 125.7, 121.1, 119.3, 117.6, 109.3, 108.3, 63.5, 51.9, 51.8, 47.4, 46.5, 46.3, 34.6, 23.9. **HRMS (ESI-TOF)** calcd for $C_{24}H_{26}N_2O_4$ (M+H$^+$): 407.1965; Found: 407.1966.

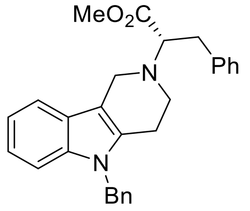

4k

methyl (*S*)-2-(5-benzyl-1,3,4,5-tetrahydro-2*H*-pyrido[4,3-*b*]indol-2-yl)-3-phenylpropanoate (4k): According to the general procedure for 3 h, light yellow gel, 84 mg, 99% yield. **^{1}H NMR (400 MHz, CDCl$_3$)** δ 7.45 (d, *J* = 7.2 Hz, 1H), 7.32 – 7.15 (m, 9H), 7.14 – 7.04 (m, 2H), 7.00 (d, *J* = 7.2 Hz, 2H), 5.22 (s, 2H), 3.99 (q, *J* = 13.2 Hz, 2H), 3.76 (dd, J_1 = 9.6 Hz, J_1 = 5.6 Hz, 1H), 3.61 (s, 3H), 3.31 – 3.04 (m, 3H), 2.96 (m, 1H), 2.75 (d, *J* = 5.2 Hz, 2H). **^{13}C NMR (101 MHz, CDCl$_3$)** δ 172.1, 138.2, 137.9, 136.9, 133.7, 129.3, 128.8, 128.5, 127.4, 126.6, 126.3, 125.8, 121.1, 119.2, 117.7, 109.3, 108.4, 69.5, 51.3, 47.2, 46.6, 46.5, 36.2, 23.8. **HRMS (ESI-TOF)** calcd for $C_{28}H_{28}N_2O_2$ (M+H$^+$): 425.2224; Found: 425.2224.

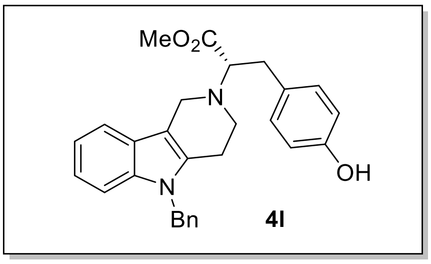

methyl (*S*)-2-(5-benzyl-1,3,4,5-tetrahydro-2*H*-pyrido[4,3-*b*]indol-2-yl)-3-(4-hydroxyphenyl)propanoate (4l): According to the general procedure for 3 h, light yellow gel, 78 mg, 89% yield. **^{1}H NMR (400 MHz, CDCl$_3$)** δ 7.56 – 7.49 (m, 1H), 7.36 – 7.22 (m, 4H), 7.22 – 7.12 (m, 2H), 7.06 (t, *J* = 9.2 Hz, 4H), 6.70 (d, *J* = 8.0 Hz, 2H), 5.26 (s, 2H), 4.14 – 4.02 (m, 2H), 3.78 (m, 1H), 3.66 (s, 3H), 3.28 – 3.16 (m, 2H), 3.12 – 3.00 (m, 2H), 2.92 – 2.72 (m, 2H). **^{13}C NMR (101 MHz, CDCl$_3$)** δ 172.4, 154.7, 137.9, 136.9, 133.7, 130.3, 129.4, 128.8, 127.4, 126.2, 125.8, 121.2, 119.3, 117.7, 115.5, 109.4, 108.1, 69.8, 51.4, 47.4, 46.5, 35.3, 23.6. **HRMS (ESI-TOF)** calcd for $C_{28}H_{28}N_2O_3$ (M+H$^+$): 441.2173; Found: 441.2173.

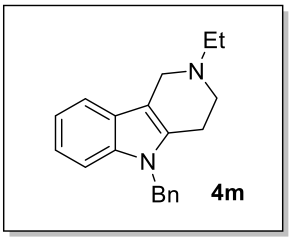

5-benzyl-2-ethyl-2,3,4,5-tetrahydro-1*H*-pyrido[4,3-*b*]indole (4m): According to the general procedure for 3 h, light yellow gel, 47mg, 81% yield. **^{1}H NMR (400 MHz, CDCl$_3$)** δ 7.51 (d, *J* = 7.6 Hz, 1H), 7.35 – 7.21 (m, 4H), 7.15 (dd, J_1 = 13.6 Hz, J_2 = 7.2 Hz, 2H), 7.07 (d, *J* = 7.6 Hz, 2H), 5.29 (s, 2H), 3.83 (s, 2H), 2.94 (t, *J* = 5.6 Hz, 2H), 2.84 (t, *J* = 5.2 Hz, 2H), 2.77 (q, *J* = 7.2 Hz, 2H), 1.29 (t, *J* = 7.2 Hz, 3H). **^{13}C NMR (101 MHz, CDCl$_3$)** δ 137.9, 136.9, 133.8, 128.8, 127.3, 126.3, 125.9, 121.0, 119.1, 117.7, 109.3, 108.2, 51.9, 50.4, 49.3, 46.5, 22.9, 12.7. **HRMS (ESI-TOF)** calcd for $C_{20}H_{22}N_2$ (M+H$^+$): 291.1856; Found: 291.1853.

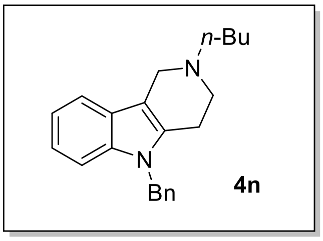

5-benzyl-2-butyl-2,3,4,5-tetrahydro-1*H*-pyrido[4,3-*b*]indole (4n): According to the general procedure for 6 h, yellow gel, 51 mg, 80% yield. **^{1}H NMR (400 MHz, CDCl$_3$)** δ 7.51 (d, *J* = 7.2

Hz, 1H), 7.34 – 7.22 (m, 4H), 7.21 – 7.11 (m, 2H), 7.07 (d, *J* = 7.6 Hz, 2H), 5.29 (s, 2H), 3.83 (s, 2H), 2.93 (t, *J* = 5.6 Hz, 2H), 2.83 (t, *J* = 5.2 Hz, 2H), 2.73 – 2.61 (m, 2H), 1.75 – 1.63 (m, 2H), 1.45 (m, 2H), 1.02 (t, *J* = 7.2 Hz, 3H). **^{13}C NMR (101 MHz, $CDCl_3$)** δ 138.0, 136.9, 133.8, 128.8, 127.3, 126.3, 126.0, 121.0, 119.1, 117.7, 109.3, 108.3, 58.0, 50.8, 49.8, 46.5, 29.8, 22.9, 20.9, 14.2. **HRMS (ESI-TOF)** calcd for $C_{22}H_{26}N_2$ (M+H$^+$): 319.2169; Found: 319.2165.

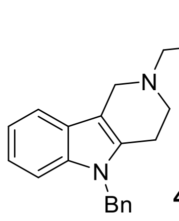

4o

5-benzyl-2-phenethyl-2,3,4,5-tetrahydro-1*H*-pyrido[4,3-*b*]indole (4o): According to the general procedure for 3 h, light yellow gel, 47 mg, 64% yield. **^{1}H NMR (400 MHz, $CDCl_3$)** δ 7.54 (d, *J* = 7.2 Hz, 1H), 7.42 – 7.25 (m, 9H), 7.21 – 7.12 (m, 2H), 7.09 (d, *J* = 7.2 Hz, 2H), 5.31 (s, 2H), 3.93 (s, 2H), 3.09 – 2.91 (m, 6H), 2.87 (t, *J* = 5.2 Hz, 2H). **^{13}C NMR (101 MHz, $CDCl_3$)** δ 140.4, 137.9, 136.9, 133.7, 128.8, 128.8, 128.5, 127.4, 126.3, 126.2, 125.9, 121.1, 119.2, 117.7, 109.3, 108.2, 60.1, 50.8, 49.7, 46.5, 34.4, 23.0. **HRMS (ESI-TOF)** calcd for $C_{26}H_{26}N_2$ (M+H$^+$): 367.2169; Found: 367.2172.

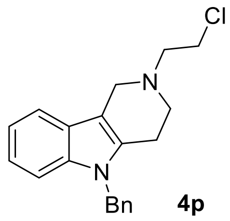

4p

5-benzyl-2-(2-chloroethyl)-2,3,4,5-tetrahydro-1*H*-pyrido[4,3-*b*]indole (4p): According to the general procedure for 3 h, light yellow gel, 56 mg, 86% yield. **^{1}H NMR (400 MHz, $CDCl_3$)** δ 7.51 (d, *J* = 7.6 Hz, 1H), 7.37 – 7.24 (m, 4H), 7.23 – 7.12 (m, 2H), 7.08 (d, *J* = 6.8 Hz, 2H), 5.29 (s, 2H), 3.93 (s, 2H), 3.78 (t, *J* = 7.2 Hz, 2H), 3.07 (t, *J* = 7.2 Hz, 2H), 3.03 (t, *J* = 5.6 Hz, 2H), 2.83 (t, *J* = 5.6 Hz, 2H). **^{13}C NMR (101 MHz, $CDCl_3$)** δ 137.9, 136.9, 133.5, 128.8, 127.4, 126.3, 125.8, 121.2, 119.3, 117.7, 109.4, 107.9, 59.1, 50.7, 49.7, 46.5, 41.6, 22.7. **HRMS (ESI-TOF)** calcd for $C_{20}H_{21}N_2Cl$ (M+H$^+$): 325.1466; Found: 325.1463.

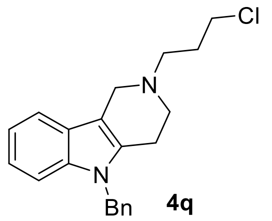

4q

5-benzyl-2-(3-chloropropyl)-2,3,4,5-tetrahydro-1*H*-pyrido[4,3-*b*]indole (4q): According to the general procedure for 3 h, light yellow gel, 53 mg, 79% yield. **^{1}H NMR (400 MHz, $CDCl_3$)** δ 7.50 (dd, J_1 = 7.2 Hz, J_2 = 1.2 Hz, 1H), 7.35 – 7.22 (m, 4H), 7.21 – 7.10 (m, 2H), 7.07 (d, J = 7.2 Hz, 2H), 5.29 (s, 2H), 3.82 (s, 2H), 3.71 (t, J = 6.4 Hz, 2H), 2.93 (t, J = 5.6 Hz, 2H), 2.83 (m, 4H), 2.15 (m, 2H). **^{13}C NMR (101 MHz, $CDCl_3$)** δ 137.9, 136.9, 133.8, 128.8, 127.4, 126.3, 125.9, 121.1, 119.2, 117.6, 109.3, 108.2, 55.0, 50.9, 49.8, 46.5, 43.4, 30.6, 22.9. **HRMS (ESI-TOF)** calcd for $C_{21}H_{23}N_2Cl$ (M+H$^+$): 339.1623; Found: 339.1619.

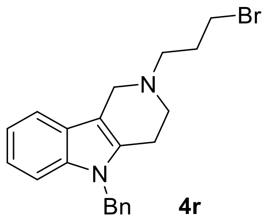

4r

5-benzyl-2-(3-bromopropyl)-2,3,4,5-tetrahydro-1*H*-pyrido[4,3-*b*]indole (4r): According to the general procedure for 3 h, white solid, m.p. = 99 – 101 °C, 46 mg, 60% yield. **^{1}H NMR (400 MHz, DMSO)** δ 7.46 (t, J = 7.2 Hz, 2H), 7.36 – 6.99 (m, 7H), 5.41 (s, 2H), 4.88 (s, 2H), 4.39 (dd, J_1 = 17.6 Hz, J_2 = 9.2 Hz, 2H), 4.21 (dd, J_1 = 17.2 Hz, J_2 = 9.6 Hz, 2H), 3.93 (t, J = 5.6 Hz, 2H), 3.17 (s, 2H), 2.72 – 2.52 (m, 2H). **^{13}C NMR (101 MHz, DMSO)** δ 138.2, 137.3, 131.2, 129.2, 127.8, 127.0, 125.3, 122.4, 120.1, 118.3, 110.8, 101.4, 62.4, 57.8, 56.3, 46.5, 18.5, 14.3. **HRMS (ESI-TOF)** calcd for $C_{21}H_{23}N_2Br$ (M+H$^+$): 383.1117; Found: 383.1107.

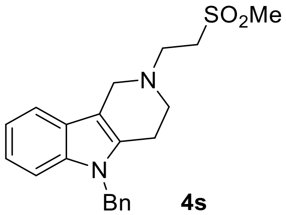

4s

5-benzyl-2-(2-(methylsulfonyl)ethyl)-2,3,4,5-tetrahydro-1*H*-pyrido[4,3-*b*]indole (4s): According to the general procedure for 3 h, white solid, m.p. = 150 – 152 °C, 57 mg, 78% yield.

^{1}H NMR (400 MHz, CDCl$_3$) δ 7.53 – 7.46 (m, 1H), 7.35 – 7.24 (m, 4H), 7.22 – 7.12 (m, 2H), 7.04 (d, *J* = 6.8 Hz, 2H), 5.29 (s, 2H), 3.87 (s, 2H), 3.31 (t, *J* = 6.4 Hz, 2H), 3.21 (t, *J* = 6.4 Hz, 2H), 3.05 (s, 3H), 2.99 (t, *J* = 5.6 Hz, 2H), 2.81 (t, *J* = 5.6 Hz, 2H). **^{13}C NMR (101 MHz, CDCl$_3$)** δ 137.8, 136.9, 133.5, 128.8, 127.5, 126.2, 125.6, 121.3, 119.4, 117.6, 109.4, 107.6, 52.9, 51.2, 50.7, 49.5, 46.5, 42.5, 22.8. **HRMS (ESI-TOF)** calcd for $C_{21}H_{24}N_2O_2S$ (M+H$^+$): 369.1631; Found: 369.1627.

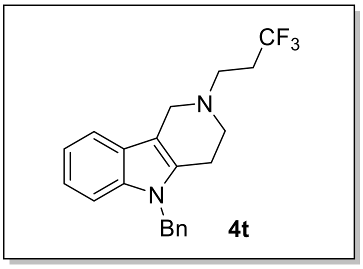

5-benzyl-2-(3,3,3-trifluoropropyl)-2,3,4,5-tetrahydro-1*H*-pyrido[4,3-*b*]indole (4t): According to the general procedure for 3 h, light yellow gel, 46 mg, 65% yield. **^{1}H NMR (400 MHz, CDCl$_3$)** δ 7.55 – 7.48 (m, 1H), 7.38 – 7.24 (m, 4H), 7.17 (m, 2H), 7.07 (d, *J* = 6.8 Hz, 2H), 5.30 (s, 2H), 3.85 (s, 2H), 2.96 (m, 4H), 2.84 (t, *J* = 5.6 Hz, 2H), 2.62 – 2.44 (m, 2H). **^{13}C NMR (101 MHz, CDCl$_3$)** δ 137.8, 136.9, 133.5, 130.8, 128.8, 128.1, 127.4, 125.8, 125.3, 122.7, 121.2, 119.3, 117.6, 109.4, 107.8, 50.7, 50.4, 50.4, 50.3, 50.3, 49.7, 46.5, 33.0, 32.7, 32.4, 32.1, 22.9. **^{19}F NMR (565 MHz, CDCl$_3$)** δ -65.24. **HRMS (ESI-TOF)** calcd for $C_{21}H_{21}N_2F_3$ (M+H$^+$): 359.1730; Found: 359.1727.

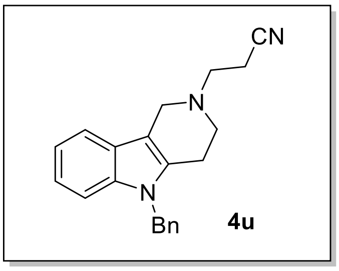

3-(5-benzyl-1,3,4,5-tetrahydro-2*H*-pyrido[4,3-*b*]indol-2-yl)propanenitrile (4u): According to the general procedure for 3 h, light yellow gel, 60 mg, 95% yield. **^{1}H NMR (400 MHz, CDCl$_3$)** δ 7.49 (d, *J* = 7.2 Hz, 1H), 7.36 – 7.24 (m, 4H), 7.22 – 7.11 (m, 2H), 7.06 (d, *J* = 6.8 Hz, 2H), 5.29 (s, 2H), 3.89 (s, 2H), 3.10 – 2.95 (m, 4H), 2.82 (t, *J* = 5.6 Hz, 2H), 2.68 (t, *J* = 7.2 Hz, 2H). **^{13}C NMR (101 MHz, CDCl$_3$)** δ 137.8, 136.9, 133.4, 128.8, 127.4, 126.2, 125.7, 121.3, 119.3, 118.9, 117.6, 109.4, 107.6, 52.9, 50.4, 49.3, 46.5, 22.7, 16.6. **HRMS (ESI-TOF)** calcd for $C_{21}H_{21}N_3$ (M+H$^+$): 316.1808; Found: 316.1807.

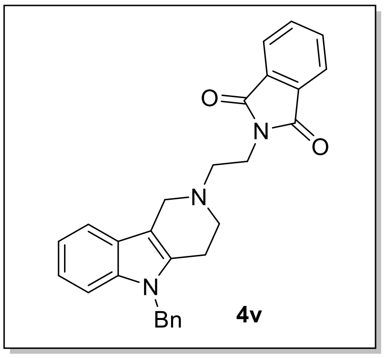

2-(2-(5-benzyl-1,3,4,5-tetrahydro-2*H*-pyrido[4,3-*b*]indol-2-yl)ethyl)isoindoline-1,3-dione (4v): According to the general procedure for 3 h, light yellow solid, m.p. = 49 – 51 °C, 73 mg, 84% yield. **^{1}H NMR (400 MHz, CDCl$_3$)** δ 7.88 – 7.75 (m, 2H), 7.71 – 7.61 (m, 2H), 7.50 – 7.37 (m, 1H), 7.29 – 7.14 (m, 4H), 7.12 – 7.02 (m, 2H), 6.99 (d, *J* = 6.4 Hz, 2H), 5.21 (s, 2H), 3.94 (t, *J* = 6.8 Hz, 2H), 3.83 (s, 2H), 3.05 – 2.86 (m, 4H), 2.73 (t, *J* = 5.6 Hz, 2H). **^{13}C NMR (101 MHz, CDCl$_3$)** δ 168.5, 138.0, 136.7, 133.9, 133.9, 132.3, 128.8, 127.3, 126.2, 125.9, 123.2, 121.0, 119.2, 117.7, 109.3, 108.2, 54.9, 50.2, 49.9, 46.4, 35.9, 22.8. **HRMS (ESI-TOF)** calcd for $C_{28}H_{25}N_3O_2$ (M+H$^+$): 436.2020; Found: 436.2014.

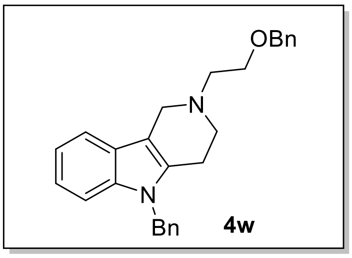

5-benzyl-2-(2-(benzyloxy)ethyl)-2,3,4,5-tetrahydro-1*H*-pyrido[4,3-*b*]indole (4w): According to the general procedure for 3 h, light yellow gel, 70 mg, 89% yield. **^{1}H NMR (400 MHz, CDCl$_3$)** δ 7.52 – 7.46 (m, 1H), 7.45 – 7.24 (m, 9H), 7.20 – 7.03 (m, 4H), 5.29 (s, 2H), 4.63 (s, 2H), 3.88 (s, 2H), 3.78 (t, *J* = 5.6 Hz, 2H), 3.05 – 2.91 (m, 4H), 2.82 (t, *J* = 5.6 Hz, 2H). **^{13}C NMR (101 MHz, CDCl$_3$)** δ 138.4, 138.0, 136.8, 133.7, 128.8, 128.5, 127.8, 127.7, 127.3, 126.3, 126.0, 121.0, 119.1, 117.7, 109.3, 108.3, 73.3, 68.5, 57.2, 51.1, 50.1, 46.4, 22.8. **HRMS (ESI-TOF)** calcd for $C_{27}H_{28}N_2O$ (M+H$^+$): 397.2274; Found: 397.2273.

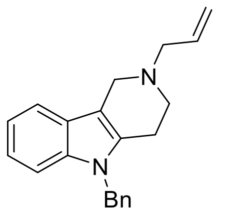

4x

2-allyl-5-benzyl-2,3,4,5-tetrahydro-1*H*-pyrido[4,3-*b*]indole (4x): According to the general procedure for 3 h, light yellow gel, 56 mg, 93% yield. **^{1}H NMR (400 MHz, $CDCl_3$)** δ 7.51 (d, *J* = 7.2 Hz, 1H), 7.36 – 7.23 (m, 4H), 7.22 – 7.10 (m, 2H), 7.07 (d, *J* = 6.8 Hz, 2H), 6.07 (m, 1H), 5.39 – 5.21 (m, 4H), 3.83 (s, 2H), 3.35 (d, *J* = 6.0 Hz, 2H), 2.94 (t, *J* = 5.6 Hz, 2H), 2.83 (t, *J* = 5.6 Hz, 2H). **^{13}C NMR (101 MHz, $CDCl_3$)** δ 137.9, 136.9, 135.6, 133.8, 128.8, 127.3, 126.3, 125.9, 121.0, 119.1, 118.0, 117.7, 109.3, 108.4, 61.2, 50.2, 49.8, 46.5, 22.9. **HRMS (ESI-TOF)** calcd for $C_{21}H_{22}N_2$ (M+H$^+$): 303.1856; Found: 303.1853.

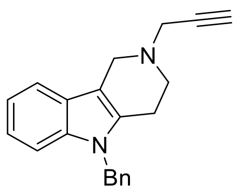

4y

5-benzyl-2-(prop-2-yn-1-yl)-2,3,4,5-tetrahydro-1*H*-pyrido[4,3-*b*]indole (4y): According to the general procedure for 3 h, light yellow gel, 53 mg, 89% yield. **^{1}H NMR (400 MHz, $CDCl_3$)** δ 7.56 – 7.48 (m, 1H), 7.36 – 7.23 (m, 4H), 7.21 – 7.12 (m, 2H), 7.06 (d, *J* = 7.2 Hz, 2H), 5.30 (s, 2H), 3.97 (s, 2H), 3.67 (d, *J* = 2.4 Hz, 2H), 3.04 (t, *J* = 5.6 Hz, 2H), 2.86 (t, *J* = 5.6 Hz, 2H), 2.36 (t, *J* = 2.4 Hz, 1H). **^{13}C NMR (101 MHz, $CDCl_3$)** δ 137.9, 136.9, 133.3, 128.8, 127.4, 126.2, 125.8, 121.1, 119.2, 117.7, 109.3, 108.2, 79.1, 73.4, 49.5, 48.5, 46.6, 46.4, 23.0. **HRMS (ESI-TOF)** calcd for $C_{21}H_{20}N_2$ (M+H$^+$): 301.1699; Found: 301.1699.

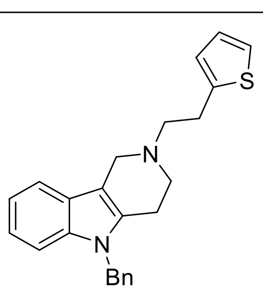

4z

5-benzyl-2-(2-(thiophen-2-yl)ethyl)-2,3,4,5-tetrahydro-1*H*-pyrido[4,3-*b*]indole (4z): According to the general procedure for 3 h, light yellow gel, 51 mg, 69% yield. **^{1}H NMR (400 MHz, CDCl$_3$)** δ 7.50 – 7.42 (m, 1H), 7.29 – 7.19 (m, 4H), 7.17 – 7.06 (m, 3H), 7.02 (d, J = 6.8 Hz, 2H), 6.93 (dd, J_1 = 5.2 Hz, J_2 = 3.6 Hz, 1H), 6.87 (d, J = 3.2 Hz, 1H), 5.25 (s, 2H), 3.86 (s, 2H), 3.23 – 3.12 (m, 2H), 3.01 – 2.91 (m, 4H), 2.80 (t, J = 5.6 Hz, 2H). **^{13}C NMR (101 MHz, CDCl$_3$)** δ 142.8, 137.9, 136.9, 133.8, 128.8, 127.3, 126.7, 126.3, 125.9, 124.7, 123.5, 121.0, 119.2, 117.7, 109.3, 108.2, 59.6, 50.7, 49.7, 46.5, 28.5, 22.9. **HRMS (ESI-TOF)** calcd for $C_{24}H_{24}N_2S$ (M+H$^+$): 373.1733; Found: 373.1732.

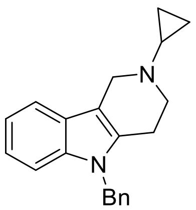

4aa

5-benzyl-2-cyclopropyl-2,3,4,5-tetrahydro-1*H*-pyrido[4,3-*b*]indole (4aa): According to the general procedure for 3 h, light yellow solid, m.p. = 92 – 94 °C, 55 mg, 91% yield. **^{1}H NMR (400 MHz, CDCl$_3$)** δ 7.56 – 7.48 (m, 1H), 7.35 – 7.25 (m, 4H), 7.21 – 7.12 (m, 2H), 7.09 (d, J = 6.8 Hz, 2H), 5.28 (s, 2H), 3.99 (s, 2H), 3.12 (t, J = 5.6 Hz, 2H), 2.82 (t, J = 5.6 Hz, 2H), 2.05 – 1.99 (m, 1H), 0.63 (d, J = 4.0 Hz, 4H). **^{13}C NMR (101 MHz, CDCl$_3$)** δ 138.0, 136.9, 133.7, 128.8, 127.3, 126.4, 125.9, 121.0, 119.1, 117.7, 109.2, 108.5, 50.8, 49.8, 46.5, 37.9, 22.8, 6.5. **HRMS (ESI-TOF)** calcd for $C_{21}H_{22}N_2$ (M+H$^+$): 303.1856; Found: 303.1855.

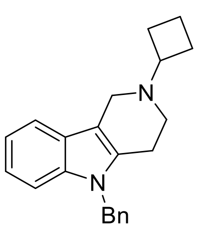

4ab

5-benzyl-2-cyclobutyl-2,3,4,5-tetrahydro-1*H*-pyrido[4,3-*b*]indole (4ab): According to the general procedure for 3 h, yellow solid, m.p. = 73 – 75 °C, 50 mg, 80% yield. **^{1}H NMR (400 MHz, CDCl$_3$)** δ 7.53 – 7.46 (m, 1H), 7.27 (m, 4H), 7.19 – 7.04 (m, 4H), 5.28 (s, 2H), 3.69 (s, 2H), 3.10 (t, J = 7.6 Hz, 1H), 2.79 (m, 4H), 2.29 – 2.16 (m, 2H), 2.13 – 1.98 (m, 2H), 1.88 – 1.67

(m, 2H). **^{13}C NMR (101 MHz, $CDCl_3$)** δ 137.9, 136.9, 133.7, 128.7, 127.3, 126.3, 126.0, 121.0, 119.1, 117.7, 109.2, 108.0, 59.7, 46.5, 46.5, 46.2, 27.8, 22.7, 14.5. **HRMS (ESI-TOF)** calcd for $C_{22}H_{24}N_2$ (M+H$^+$): 317.2012; Found: 317.2019.

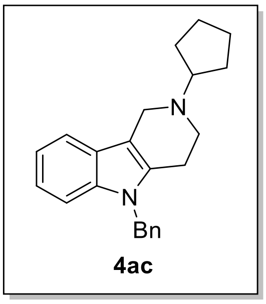

5-benzyl-2-cyclopentyl-2,3,4,5-tetrahydro-1*H*-pyrido[4,3-*b*]indole (4ac): According to the general procedure for 3 h, light yellow gel, 48 mg, 73% yield. **^{1}H NMR (400 MHz, $CDCl_3$)** δ 7.53 – 7.41 (m, 1H), 7.24 (m, 4H), 7.15 – 6.94 (m, 4H), 5.24 (s, 2H), 3.87 (s, 2H), 2.96 (t, J = 5.6 Hz, 2H), 2.91 – 2.84 (m, 1H), 2.81 (t, J = 5.6 Hz, 2H), 2.04 (m, 2H), 1.77 (s, 2H), 1.61 (m, 4H). **^{13}C NMR (101 MHz, $CDCl_3$)** δ 137.9, 136.9, 133.7, 128.8, 127.3, 126.3, 126.0, 121.0, 119.1, 117.7, 109.3, 108.0, 66.6, 49.3, 48.8, 46.5, 30.9, 24.2, 22.8. **HRMS (ESI-TOF)** calcd for $C_{23}H_{26}N_2$ (M+H$^+$): 331.2169; Found: 331.2161.

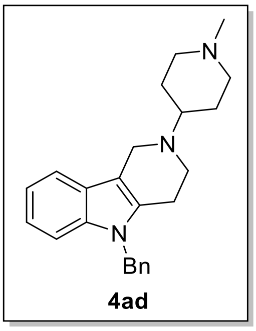

5-benzyl-2-(1-methylpiperidin-4-yl)-2,3,4,5-tetrahydro-1*H*-pyrido[4,3-*b*]indole (4ad): According to the general procedure for 3 h, light yellow gel, 42 mg, 58% yield. **^{1}H NMR (400 MHz, $CDCl_3$)** δ 7.47 (d, J = 7.2 Hz, 1H), 7.37 – 7.20 (m, 4H), 7.19 – 6.97 (m, 4H), 5.27 (s, 2H), 3.94 (s, 2H), 3.06 – 2.93 (m, 4H), 2.79 (t, J = 5.2 Hz, 2H), 2.70 – 2.55 (m, 1H), 2.33 (s, 3H), 2.11 – 1.93 (m, 4H), 1.88 – 1.73 (m, 2H). **^{13}C NMR (101 MHz, $CDCl_3$)** δ 138.0, 136.9, 134.0, 128.7, 127.3, 126.3, 126.0, 120.9, 119.1, 117.6, 109.2, 108.7, 61.1, 55.6, 46.5, 46.4, 46.2, 45.6, 28.4, 23.6. **HRMS (ESI-TOF)** calcd for $C_{24}H_{29}N_3$ (M+H$^+$): 360.2434; Found: 360.2436.

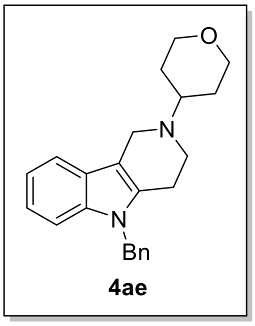

5-benzyl-2-(tetrahydro-2*H*-pyran-4-yl)-2,3,4,5-tetrahydro-1*H*-pyrido[4,3-*b*]indole (4ae): According to the general procedure for 3 h, light yellow gel, 54 mg, 79% yield. **^{1}H NMR (400 MHz, CDCl$_3$)** δ 7.52 – 7.46 (m, 1H), 7.28 (m, 4H), 7.20 – 7.02 (m, 4H), 5.28 (s, 2H), 4.12 (dd, J_1 = 11.2 Hz, J_2 = 4.0 Hz, 2H), 3.95 (s, 2H), 3.48 (t, J = 11.2 Hz, 2H), 3.02 (t, J = 5.6 Hz, 2H), 2.88 – 2.73 (m, 3H), 1.99 – 1.91 (m, 2H), 1.87 – 1.74 (m, 2H). **^{13}C NMR (101 MHz, CDCl$_3$)** δ 137.9, 136.9, 133.9, 128.8, 127.3, 126.3, 126.0, 121.0, 119.1, 117.6, 109.3, 108.4, 67.7, 60.4, 46.5, 46.4, 45.5, 29.9, 23.4. **HRMS (ESI-TOF)** calcd for $C_{23}H_{26}N_2O$ (M+H$^+$): 347.2118; Found: 347.2119.

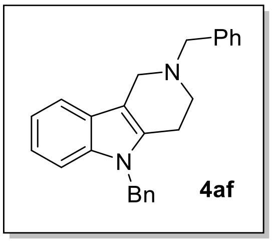

2,5-dibenzyl-2,3,4,5-tetrahydro-1*H*-pyrido[4,3-*b*]indole (4af): According to the general procedure for 3 h, light yellow solid, m.p. = 129 – 131 °C, 66 mg, 93% yield. **^{1}H NMR (400 MHz, CDCl$_3$)** δ 7.48 (d, J = 7.6 Hz, 3H), 7.40 (t, J = 7.2 Hz, 2H), 7.37 – 7.27 (m, 5H), 7.20 – 7.02 (m, 4H), 5.29 (s, 2H), 3.86 (d, J = 6.8 Hz, 4H), 2.94 (t, J = 5.6 Hz, 2H), 2.81 (t, J = 5.6 Hz, 2H). **^{13}C NMR (101 MHz, CDCl$_3$)** δ 138.7, 138.0, 136.9, 133.9, 129.2, 128.8, 128.4, 127.3, 127.2, 126.3, 125.9, 121.0, 119.1, 117.7, 109.2, 108.5, 62.4, 50.2, 50.0, 46.5, 22.9. **HRMS (ESI-TOF)** calcd for $C_{25}H_{24}N_2$ (M+H$^+$): 353.2012; Found: 353.2009.

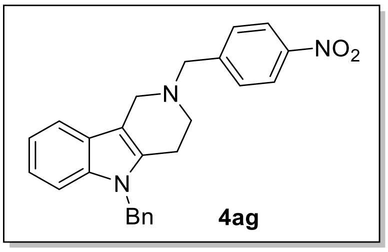

5-benzyl-2-(4-nitrobenzyl)-2,3,4,5-tetrahydro-1*H*-pyrido[4,3-*b*]indole (4ag): According to the general procedure for 3 h, light yellow solid, m.p. = 108 – 110 °C, 61 mg, 77% yield. **^{1}H NMR (400 MHz, CDCl$_3$)** δ 8.18 (d, *J* = 8.4 Hz, 2H), 7.58 (d, *J* = 8.4 Hz, 2H), 7.39 (d, *J* = 7.6 Hz, 1H), 7.31 – 7.18 (m, 4H), 7.08 (m, 4H), 5.24 (s, 2H), 3.87 (s, 2H), 3.78 (s, 2H), 2.88 (t, *J* = 5.6 Hz, 2H), 2.77 (t, *J* = 5.6 Hz, 2H). **^{13}C NMR (101 MHz, CDCl$_3$)** δ 147.3, 146.9, 137.9, 136.9, 133.6, 129.5, 128.8, 127.4, 126.3, 125.7, 123.7, 121.2, 119.3, 117.7, 109.4, 108.0, 61.5, 50.5, 50.1, 46.5, 22.9. **HRMS (ESI-TOF)** calcd for $C_{25}H_{23}N_3O_2$ (M+H$^+$): 398.1863; Found: 398.1862.

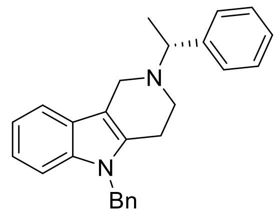

4ah

(*R*)-5-benzyl-2-(1-phenylethyl)-2,3,4,5-tetrahydro-1*H*-pyrido[4,3-*b*]indole (4ah): According to the general procedure for 3 h, light yellow gel, 64 mg, 87% yield. **^{1}H NMR (400 MHz, CDCl$_3$)** δ 7.46 – 7.28 (m, 5H), 7.28 – 7.15 (m, 5H), 7.14 – 6.96 (m, 4H), 5.20 (s, 2H), 4.01 (d, *J* = 13.6 Hz, 1H), 3.77 – 3.58 (m, 2H), 3.01 – 2.87 (m, 1H), 2.82 – 2.54 (m, 3H), 1.52 (d, *J* = 6.8 Hz, 3H). **^{13}C NMR (101 MHz, CDCl$_3$)** δ 144.6, 138.0, 136.9, 134.1, 128.8, 128.4, 127.6, 127.3, 127.0, 126.4, 126.1, 120.9, 119.1, 117.7, 109.2, 108.6, 64.3, 48.0, 47.2, 46.5, 23.2, 20.6. **HRMS (ESI-TOF)** calcd for $C_{26}H_{26}N_2$ (M+H$^+$): 367.2169; Found: 367.2167.

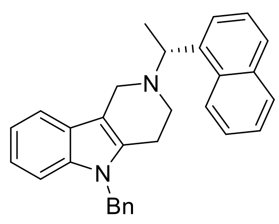

4ai

(*R*)-5-benzyl-2-(1-(naphthalen-1-yl)ethyl)-2,3,4,5-tetrahydro-1*H*-pyrido[4,3-*b*]indole (4ai): According to the general procedure for 3 h, light yellow gel, 73 mg, 88% yield. **^{1}H NMR (400 MHz, CDCl$_3$)** δ 8.55 (d, *J* = 6.0 Hz, 1H), 7.99 – 7.91 (m, 1H), 7.83 (m, 2H), 7.55 (m, 4H), 7.32 (m, 4H), 7.24 – 7.14 (m, 2H), 7.10 (d, *J* = 7.2 Hz, 2H), 5.28 (s, 2H), 4.53 (q, *J* = 6.4 Hz, 1H), 4.30 (d, *J* = 13.6 Hz, 1H), 3.84 (d, *J* = 13.6 Hz, 1H), 3.17 – 3.00 (m, 1H), 2.87 – 2.75 (m, 2H), 2.64 (m, 1H), 1.73 (d, *J* = 6.4 Hz, 3H). **^{13}C NMR (101 MHz, CDCl$_3$)** δ 141.4, 138.1, 136.9, 134.4, 134.2, 131.7, 128.9, 128.8, 127.4, 127.4, 126.4, 126.1, 125.7, 125.4, 124.6, 124.1, 121.0,

119.2, 117.7, 109.3, 108.7, 60.9, 48.5, 47.6, 46.5, 23.4, 20.1. **HRMS (ESI-TOF)** calcd for $C_{30}H_{28}N_2$ (M+H$^+$): 417.2325; Found: 417.2324.

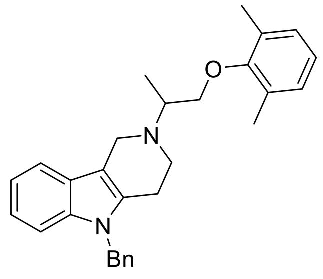

4aj

5-benzyl-2-(1-(2,6-dimethylphenoxy)propan-2-yl)-2,3,4,5-tetrahydro-1*H*-pyrido[4,3-*b*] indole (4aj): According to the general procedure for 3 h, light yellow gel, 70 mg, 82% yield. **^{1}H NMR (400 MHz, CDCl$_3$)** δ 7.54 (d, *J* = 7.6 Hz, 1H), 7.38 – 7.26 (m, 4H), 7.24 – 7.13 (m, 2H), 7.13 – 7.04 (m, 4H), 7.03 – 6.93 (m, 1H), 5.31 (s, 2H), 4.09 (s, 3H), 3.96 – 3.83 (m, 1H), 3.55 – 3.38 (m, 1H), 3.25 – 3.07 (m, 2H), 2.85 (t, *J* = 5.2 Hz, 2H), 2.40 (s, 6H), 1.48 (d, *J* = 6.8 Hz, 3H). **^{13}C NMR (101 MHz, CDCl$_3$)** δ 156.0, 138.0, 136.9, 134.1, 131.0, 129.0, 128.8, 127.3, 126.4, 126.1, 123.9, 121.0, 119.1, 117.7, 109.3, 108.9, 74.2, 59.1, 46.8, 46.5, 45.9, 23.9, 16.6, 13.3. **HRMS (ESI-TOF)** calcd for $C_{29}H_{32}N_2O$ (M+H$^+$): 425.2587; Found: 425.2592.

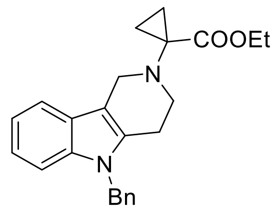

4ak

ethyl 1-(5-benzyl-1,3,4,5-tetrahydro-2*H*-pyrido[4,3-*b*]indol-2-yl)cyclopropane-1-carboxylate (4ak): According to the general procedure for 3 h, light yellow gel, 36 mg, 50% yield. **^{1}H NMR (400 MHz, CDCl$_3$)** δ 7.49 (d, *J* = 7.2 Hz, 1H), 7.39 – 7.21 (m, 4H), 7.20 – 6.99 (m, 4H), 5.27 (s, 2H), 4.22 (q, *J* = 7.2 Hz, 3H), 3.45 (s, 2H), 2.72 (s, 2H), 1.45 (s, 2H), 1.32 (t, *J* = 7.2 Hz, 4H), 1.15 (s, 2H). **^{13}C NMR (101 MHz, CDCl$_3$)** δ 174.5, 138.1, 136.8, 133.8, 128.8, 127.3, 126.3, 125.8, 120.9, 119.1, 117.6, 109.2, 109.1, 60.4, 47.7, 46.4, 45.9, 45.3, 24.3, 19.4, 14.5. **HRMS (ESI-TOF)** calcd for $C_{24}H_{26}N_2O_2$ (M+H$^+$): 375.2067; Found: 375.2060.

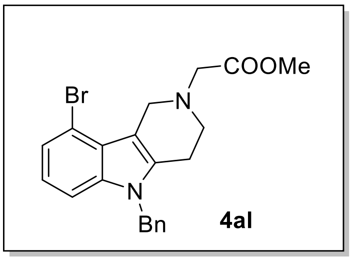

methyl 2-(5-benzyl-9-bromo-1,3,4,5-tetrahydro-2*H*-pyrido[4,3-*b*]indol-2-yl)acetate (4al): According to the general procedure for 3 h, light yellow gel, 64 mg, 78% yield. **^{1}H NMR (400 MHz, CDCl$_3$)** δ 7.36 – 7.20 (m, 4H), 7.17 (d, *J* = 8.0 Hz, 1H), 7.01 (d, *J* = 6.8 Hz, 2H), 6.95 (t, *J* = 8.0 Hz, 1H), 5.26 (s, 2H), 4.32 (s, 2H), 3.80 (s, 3H), 3.60 (s, 2H), 3.05 (t, *J* = 5.6 Hz, 2H), 2.82 (t, *J* = 5.6 Hz, 2H). **^{13}C NMR (101 MHz, CDCl$_3$)** δ 171.2, 137.8, 137.2, 134.6, 128.9, 127.5, 126.1, 125.3, 123.0, 121.9, 113.4, 108.7, 108.4, 58.5, 51.8, 50.6, 49.5, 46.6, 22.9. **HRMS (ESI-TOF)** calcd for $C_{21}H_{21}N_2O_2Br$ (M+H$^+$): 413.0859; Found: 413.0853.

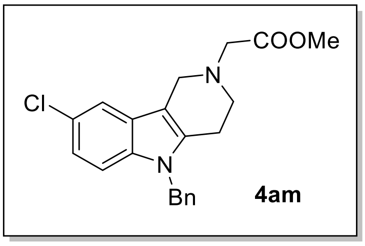

methyl 2-(5-benzyl-8-chloro-1,3,4,5-tetrahydro-2*H*-pyrido[4,3-*b*]indol-2-yl)acetate (4am): According to the general procedure for 3 h, light yellow gel, 44 mg, 60% yield. **^{1}H NMR (400 MHz, CDCl$_3$)** δ 7.38 (d, *J* = 1.6 Hz, 1H), 7.30 – 7.20 (m, 3H), 7.10 (d, *J* = 8.4 Hz, 1H), 7.04 (dd, J_1 = 8.4 Hz, J_2 = 2.0 Hz, 1H), 6.97 (d, *J* = 6.8 Hz, 2H), 5.21 (s, 2H), 3.87 (s, 2H), 3.76 (s, 3H), 3.52 (s, 2H), 3.02 (t, *J* = 5.6 Hz, 2H), 2.79 (t, *J* = 5.6 Hz, 2H). **^{13}C NMR (101 MHz, CDCl$_3$)** δ 171.1, 137.4, 135.2, 134.9, 128.9, 127.5, 126.8, 126.1, 124.9, 121.2, 117.3, 110.3, 107.7, 58.6, 51.9, 50.2, 49.2, 46.6, 22.8. **HRMS (ESI-TOF)** calcd for $C_{21}H_{21}N_2O_2Cl$ (M+H$^+$): 369.1364; Found:369.1358.

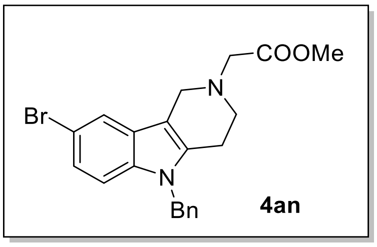

methyl 2-(5-benzyl-8-bromo-1,3,4,5-tetrahydro-2*H*-pyrido[4,3-*b*]indol-2-yl)acetate (4an): According to the general procedure for 3 h, light yellow gel, 60 mg, 73% yield. **^{1}H NMR (400**

MHz, $CDCl_3$) δ 7.58 (d, *J* = 1.6 Hz, 1H), 7.35 – 7.25 (m, 3H), 7.21 (dd, J_1 = 8.4 Hz, J_2 =1.6 Hz, 1H), 7.10 (d, *J* = 8.4 Hz, 1H), 7.01 (d, *J* = 6.8 Hz, 2H), 5.25 (s, 2H), 3.90 (s, 2H), 3.80 (s, 3H), 3.56 (s, 2H), 3.06 (t, *J* = 5.6 Hz, 2H), 2.83 (t, *J* = 5.6 Hz, 2H). **^{13}C NMR (101 MHz, $CDCl_3$)** δ 171.1, 137.3, 135.5, 134.8, 128.9, 127.5, 127.4, 126.1, 123.8, 120.4, 112.5, 110.8, 107.6, 58.6, 51.9, 50.2, 49.2, 46.6, 22.8. **HRMS (ESI-TOF)** calcd for $C_{21}H_{21}N_2O_2Br$ (M+H$^+$): 413.0859; Found:413.0843.

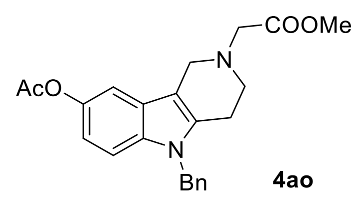

4ao

methyl 2-(8-acetoxy-5-benzyl-1,3,4,5-tetrahydro-2*H*-pyrido[4,3-*b*]indol-2-yl)acetate (4ao): According to the general procedure for 3 h, light yellow gel, 57 mg, 73% yield. **^{1}H NMR (400 MHz, $CDCl_3$)** δ 7.32 – 7.21 (m, 3H), 7.21 – 7.12 (m, 2H), 7.06 – 6.97 (m, 2H), 6.83 (dd, J_1 = 8.8 Hz, J_2 =2.0 Hz, 1H), 5.24 (s, 2H), 3.89 (s, 2H), 3.77 (s, 3H), 3.53 (s, 2H), 3.04 (t, *J* = 5.6 Hz, 2H), 2.80 (t, *J* = 5.6 Hz, 2H), 2.32 (s, 3H). **^{13}C NMR (101 MHz, $CDCl_3$)** δ 171.2, 170.5, 144.1, 137.5, 134.8, 134.7, 128.8, 127.5, 126.2, 126.0, 114.9, 110.0, 109.6, 108.1, 58.5, 51.9, 50.2, 49.3, 46.6, 22.7, 21.3. **HRMS (ESI-TOF)** calcd for $C_{23}H_{24}N_2O_4$ (M+H$^+$): 393.1809; Found: 393.1805.

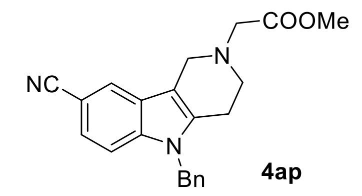

4ap

methyl 2-(5-benzyl-8-cyano-1,3,4,5-tetrahydro-2*H*-pyrido[4,3-*b*]indol-2-yl)acetate (4ap): According to the general procedure for 3 h, light yellow gel, 61 mg, 85% yield. **^{1}H NMR (400 MHz, $CDCl_3$)** δ 7.76 (s, 1H), 7.34 (d, *J* = 8.4 Hz, 1H), 7.30 – 7.26 (m, 3H), 7.24 (s, 1H), 6.97 (d, *J* = 6.4 Hz, 2H), 5.27 (s, 2H), 3.91 (s, 2H), 3.77 (s, 3H), 3.55 (s, 2H), 3.04 (t, *J* = 5.6 Hz, 2H), 2.82 (t, *J* = 5.6 Hz, 2H). **^{13}C NMR (101 MHz, $CDCl_3$)** δ 171.0, 138.4, 136.7, 136.2, 129.0, 127.8, 126.1, 125.6, 124.3, 123.1, 120.9, 110.0, 109.1, 102.2, 58.5, 51.9, 50.0, 48.9, 46.7, 22.9. **HRMS (ESI-TOF)** calcd for $C_{22}H_{21}N_3O_2$ (M+H$^+$): 360.1707; Found: 360.1708.

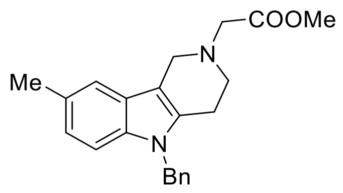

4aq

methyl 2-(5-benzyl-8-methyl-1,3,4,5-tetrahydro-2*H*-pyrido[4,3-*b*]indol-2-yl)acetate (4aq): According to the general procedure for 3 h, yellow gel, 57 mg, 82% yield. **^{1}H NMR (400 MHz, $CDCl_3$)** δ 7.36 – 7.22 (m, 4H), 7.15 (d, *J* = 8.4 Hz, 1H), 7.05 (d, *J* = 6.8 Hz, 2H), 6.99 (d, *J* = 8.4 Hz, 1H), 5.26 (s, 2H), 3.96 (s, 2H), 3.82 (s, 3H), 3.58 (s, 2H), 3.07 (t, *J* = 5.6 Hz, 2H), 2.83 (t, *J* = 5.6 Hz, 2H), 2.49 (s, 3H). **^{13}C NMR (101 MHz, $CDCl_3$)** δ 171.3, 138.0, 135.2, 133.3, 128.8, 128.4, 127.3, 126.2, 126.0, 122.6, 117.6, 109.0, 107.3, 58.6, 51.9, 50.4, 49.5, 46.4, 22.6, 21.5. **HRMS (ESI-TOF)** calcd for $C_{22}H_{24}N_2O_2$ ($M+H^+$): 349.1911; Found: 349.1908.

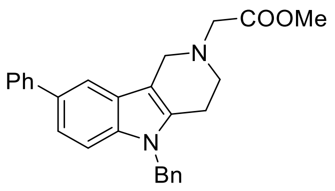

4ar

methyl 2-(5-benzyl-8-phenyl-1,3,4,5-tetrahydro-2*H*-pyrido[4,3-*b*]indol-2-yl)acetate (4ar): According to the general procedure for 3 h, light yellow gel, 76 mg, 93% yield. **^{1}H NMR (400 MHz, $CDCl_3$)** δ 7.69 (m, 3H), 7.51 – 7.40 (m, 3H), 7.38 – 7.25 (m, 5H), 7.09 (d, *J* = 6.8 Hz, 2H), 5.31 (s, 2H), 4.03 (s, 2H), 3.82 (s, 3H), 3.60 (s, 2H), 3.10 (t, *J* = 5.6 Hz, 2H), 2.86 (t, *J* = 5.6 Hz, 2H). **^{13}C NMR (101 MHz, $CDCl_3$)** δ 171.2, 142.6, 137.8, 136.4, 134.0, 132.8, 128.9, 128.7, 127.4, 127.4, 126.3, 126.3, 120.9, 116.3, 109.5, 108.2, 58.5, 51.9, 50.3, 49.4, 46.6, 22.7. **HRMS (ESI-TOF)** calcd for $C_{27}H_{26}N_2O_2$ ($M+H^+$): 411.2067; Found: 411.2064.

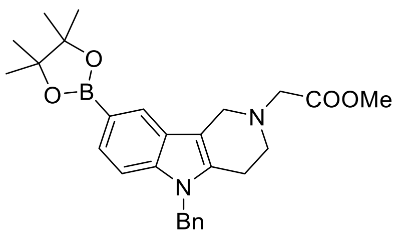

4as

methyl 2-(5-benzyl-8-(4,4,5,5-tetramethyl-1,3,2-dioxaborolan-2-yl)-1,3,4,5-tetrahydro-2*H*-pyrido[4,3-*b*]indol-2-yl)acetate (4as): According to the general procedure for 3 h, light yellow gel, 74 mg, 80% yield. **^{1}H NMR (400 MHz, CDCl$_3$)** δ 8.01 (s, 1H), 7.62 (d, *J* = 8.0 Hz, 1H), 7.34 – 7.19 (m, 4H), 7.01 (d, *J* = 6.4 Hz, 2H), 5.28 (s, 2H), 3.97 (s, 2H), 3.80 (s, 3H), 3.55 (s, 2H), 3.05 (t, *J* = 5.6 Hz, 2H), 2.81 (t, *J* = 5.6 Hz, 2H), 1.39 (s, 12H). **^{13}C NMR (101 MHz, CDCl$_3$)** δ 171.1, 138.9, 137.7, 133.4, 128.8, 127.5, 127.4, 126.2, 125.6, 125.5, 108.7, 108.6, 83.4, 58.6, 51.9, 50.3, 49.5, 46.4, 24.9, 22.5. **HRMS (ESI-TOF)** calcd for $C_{27}H_{33}N_2O_4B$ (M+H$^+$): 461.2606; Found: 461.2601.

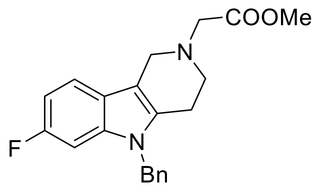

4at

methyl 2-(5-benzyl-7-fluoro-1,3,4,5-tetrahydro-2*H*-pyrido[4,3-*b*]indol-2-yl)acetate (4at): According to the general procedure for 3 h, light yellow gel, 48 mg, 68% yield. **^{1}H NMR (400 MHz, CDCl$_3$)** δ 7.39 – 7.22 (m, 4H), 7.04 (d, *J* = 7.2 Hz, 2H), 6.97 – 6.80 (m, 2H), 5.22 (s, 2H), 3.94 (s, 2H), 3.80 (s, 3H), 3.57 (s, 2H), 3.06 (t, *J* = 5.6 Hz, 2H), 2.83 (t, *J* = 5.6 Hz, 2H). **^{13}C NMR (101 MHz, CDCl$_3$)** δ 171.2, 160.7, 158.4, 137.3, 137.0, 136.8, 133.7, 133.7, 128.9, 127.5, 126.2, 122.4, 118.3, 118.2, 108.0, 107.7, 107.5, 96.2, 96.0, 58.5, 51.9, 50.2, 49.3, 46.6, 22.7. **^{19}F NMR (565 MHz, CDCl$_3$)** δ -121.61. **HRMS (ESI-TOF)** calcd for $C_{21}H_{21}N_2O_2F$ (M+H$^+$): 353.1660; Found: 353.1656.

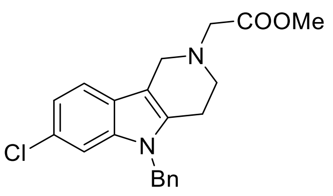

4au

methyl 2-(5-benzyl-7-chloro-1,3,4,5-tetrahydro-2*H*-pyrido[4,3-*b*]indol-2-yl)acetate (4au): According to the general procedure for 3 h, light yellow gel, 33 mg, 45% yield. **^{1}H NMR (400 MHz, CDCl$_3$)** δ 7.32 (m, 4H), 7.23 (s, 1H), 7.08 (dd, J_1 = 8.4 Hz, J_2 =1.6 Hz, 1H), 7.02 (d, *J* = 7.2 Hz, 2H), 5.23 (s, 2H), 3.93 (s, 2H), 3.80 (s, 3H), 3.57 (s, 2H), 3.06 (t, *J* = 5.6 Hz, 2H), 2.82 (t, *J* = 5.6 Hz, 2H). **^{13}C NMR (101 MHz, CDCl$_3$)** δ 171.1, 137.3, 134.2, 128.9, 127.6, 127.0,

126.1, 124.4, 119.8, 118.5, 109.4, 108.1, 58.5, 51.9, 50.1, 49.2, 46.5, 22.7. **HRMS (ESI-TOF)** calcd for $C_{21}H_{21}N_2O_2Cl$ $(M+H^+)$: 369.1364; Found: 369.1359.

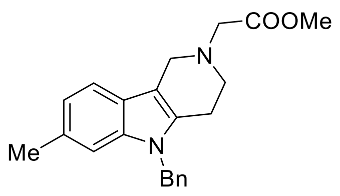

4av

methyl 2-(5-benzyl-7-methyl-1,3,4,5-tetrahydro-2*H*-pyrido[4,3-*b*]indol-2-yl)acetate (4av): According to the general procedure for 3 h, light yellow gel, 38 mg, 54% yield. **^{1}H NMR (400 MHz, $CDCl_3$)** δ 7.40 – 7.23 (m, 4H), 7.05 (d, *J* = 7.2 Hz, 3H), 6.96 (d, *J* = 8.0 Hz, 1H), 5.26 (s, 2H), 3.97 (s, 2H), 3.80 (s, 3H), 3.58 (s, 2H), 3.07 (t, *J* = 5.6 Hz, 2H), 2.81 (t, *J* = 5.6 Hz, 2H), 2.46 (s, 3H). **^{13}C NMR (101 MHz, $CDCl_3$)** δ 171.2, 138.0, 137.3, 132.5, 130.9, 128.8, 127.3, 126.2, 123.7, 120.8, 117.4, 109.3, 107.5, 58.4, 51.8, 50.3, 49.5, 46.3, 22.5, 21.9. **HRMS (ESI-TOF)** calcd for $C_{22}H_{24}N_2O_2$ $(M+H^+)$: 349.1911; Found: 349.1905.

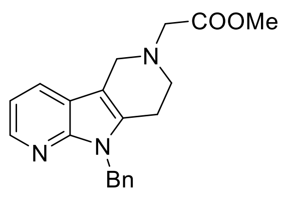

4aw

methyl 2-(9-benzyl-5,7,8,9-tetrahydro-6*H*-pyrrolo[2,3-*b*:4,5-*c*']dipyridin-6-yl)acetate (4aw): According to the general procedure for 3 h, light yellow gel, 50 mg, 75% yield. **^{1}H NMR (400 MHz, $CDCl_3$)** δ 8.29 (d, *J* = 4.4 Hz, 1H), 7.74 (d, *J* = 7.6 Hz, 1H), 7.27 (m, 3H), 7.17 – 6.96 (m, 3H), 5.49 (s, 2H), 3.91 (s, 2H), 3.79 (s, 3H), 3.54 (s, 2H), 3.03 (t, *J* = 5.6 Hz, 2H), 2.81 (t, *J* = 5.2 Hz, 2H). **^{13}C NMR (101 MHz, $CDCl_3$)** δ 171.1, 148.3, 142.0, 138.0, 134.0, 128.7, 127.3, 126.8, 125.4, 118.2, 115.5, 106.2, 58.7, 51.9, 50.1, 49.0, 44.8, 22.9. **HRMS (ESI-TOF)** calcd for $C_{20}H_{21}N_3O_2$ $(M+H^+)$: 336.1707; Found: 336.1708.

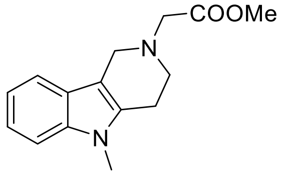

4ax

methyl 2-(5-methyl-1,3,4,5-tetrahydro-2*H*-pyrido[4,3-*b*]indol-2-yl)acetate (4ax): According to the general procedure for 3 h, light yellow gel, 45 mg, 87% yield. **^{1}H NMR (400 MHz, $CDCl_3$)** δ 7.44 (d, *J* = 7.6 Hz, 1H), 7.30 (d, *J* = 8.0 Hz, 1H), 7.21 (t, *J* = 7.6 Hz, 1H), 7.11 (t, *J* = 7.6 Hz, 1H), 3.94 (s, 2H), 3.81 (s, 3H), 3.66 (s, 3H), 3.58 (s, 2H), 3.11 (t, *J* = 5.6 Hz, 2H), 2.92 (t, *J* = 5.6 Hz, 2H). **^{13}C NMR (101 MHz, $CDCl_3$)** δ 171.3, 137.1, 133.4, 125.6, 120.8, 118.9, 117.6, 108.7, 107.2, 58.5, 51.8, 50.3, 49.5, 29.1, 22.5. **HRMS (ESI-TOF)** calcd for $C_{15}H_{18}N_2O_2$ (M+H$^+$): 259.1441; Found: 259.1439.

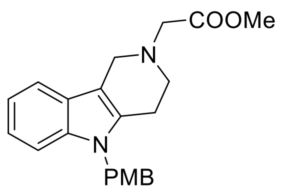

4ay

methyl 2-(5-(4-methoxybenzyl)-1,3,4,5-tetrahydro-2*H*-pyrido[4,3-*b*]indol-2-yl)acetate (4ay): According to the general procedure for 3 h, light yellow gel, 54 mg, 74% yield. **^{1}H NMR (400 MHz, $CDCl_3$)** δ 7.47 (d, *J* = 7.6 Hz, 1H), 7.27 (d, *J* = 8.0 Hz, 1H), 7.14 (m, 2H), 6.99 (d, *J* = 8.4 Hz, 2H), 6.83 (d, *J* = 8.4 Hz, 2H), 5.23 (s, 2H), 3.97 (s, 2H), 3.80 (m, 6H), 3.58 (s, 2H), 3.07 (t, *J* = 5.6 Hz, 2H), 2.85 (t, *J* = 5.6 Hz, 2H). **^{13}C NMR (101 MHz, $CDCl_3$)** δ 171.2, 158.9, 136.8, 133.2, 129.9, 127.5, 125.8, 121.0, 119.1, 117.7, 114.1, 109.3, 107.7, 58.6, 55.3, 51.9, 50.3, 49.5, 45.9, 22.7. **HRMS (ESI-TOF)** calcd for $C_{22}H_{24}N_2O_3$ (M+H$^+$): 365.1860; Found: 365.1855.

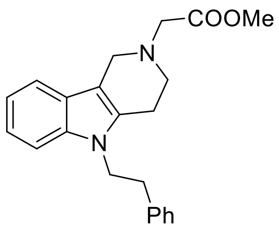

4az

methyl 2-(5-phenethyl-1,3,4,5-tetrahydro-2*H*-pyrido[4,3-*b*]indol-2-yl)acetate (4az): According to the general procedure for 3 h, light yellow gel, 58 mg, 83% yield. **^{1}H NMR (400 MHz, $CDCl_3$)** δ 7.46 (d, *J* = 7.6 Hz, 1H), 7.39 – 7.26 (m, 4H), 7.23 (dd, J_1 = 13.6 Hz, J_2 = 6.4 Hz, 1H), 7.14 (t, *J* = 7.2 Hz, 1H), 7.08 (d, *J* = 6.8 Hz, 2H), 4.27 (t, *J* = 7.2 Hz, 2H), 3.94 (s, 2H), 3.81 (s, 3H), 3.52 (s, 2H), 3.05 (t, *J* = 7.2 Hz, 2H), 2.98 (t, *J* = 5.6 Hz, 2H), 2.55 (t, *J* = 5.6 Hz,

2H). **^{13}C NMR (101 MHz, $CDCl_3$)** δ 171.3, 138.8, 136.0, 133.1, 128.9, 128.7, 126.7, 126.0, 120.8, 119.0, 117.8, 109.0, 107.0, 58.2, 51.8, 50.2, 49.4, 44.9, 36.6, 22.1. **HRMS (ESI-TOF)** calcd for $C_{22}H_{24}N_2O_2$ (M+H$^+$): 349.1911; Found: 349.1904.

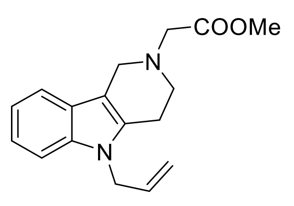

4aaa

methyl 2-(5-allyl-1,3,4,5-tetrahydro-2*H*-pyrido[4,3-*b*]indol-2-yl)acetate (4aaa): According to the general procedure for 3 h, light yellow gel, 37 mg, 65% yield. **^{1}H NMR (400 MHz, $CDCl_3$)** δ 7.44 (d, *J* = 7.6 Hz, 1H), 7.28 (d, *J* = 8.0 Hz, 1H), 7.18 (t, *J* = 7.2 Hz, 1H), 7.11 (dd, J_1 = 11.2 Hz, J_2 = 3.6 Hz, 1H), 5.94 (m, 1H), 5.14 (dd, J_1 = 10.0 Hz, J_2 = 0.8 Hz 1H), 4.93 (d, J_1 = 17.2 Hz, J_2 =0.8 Hz, 1H), 4.72 – 4.61 (m, 2H), 3.95 (s, 2H), 3.81 (s, 3H), 3.58 (s, 2H), 3.10 (t, *J* = 5.6 Hz, 2H), 2.88 (t, *J* = 5.6 Hz, 2H). **^{13}C NMR (101 MHz, $CDCl_3$)** δ 171.3, 136.5, 133.5, 133.1, 125.8, 120.9, 119.0, 117.7, 116.4, 109.1, 107.5, 58.6, 51.8, 50.3, 49.5, 45.2, 22.4. **HRMS (ESI-TOF)** calcd for $C_{17}H_{20}N_2O_2$ (M+H$^+$): 285.1598; Found: 285.1594.

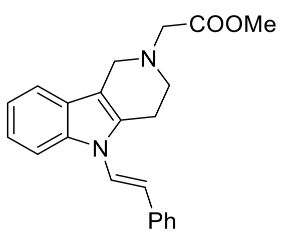

4aab

methyl (*E*)-2-(5-styryl-1,3,4,5-tetrahydro-2*H*-pyrido[4,3-*b*]indol-2-yl)acetate (4aab): According to the general procedure for 3 h, light yellow gel, 55 mg, 79% yield. **^{1}H NMR (400 MHz, $CDCl_3$)** δ 7.44 – 7.38 (m, 1H), 7.15 (m, 3H), 7.12 – 7.05 (m, 3H), 7.02 – 6.94 (m, 2H), 6.73 (d, *J* = 8.8 Hz, 1H), 6.52 (d, *J* = 8.8 Hz, 1H), 3.93 (s, 2H), 3.75 (s, 3H), 3.48 (s, 2H), 2.92 (t, *J* = 5.6 Hz, 2H), 2.58 (t, *J* = 5.6 Hz, 2H). **^{13}C NMR (101 MHz, $CDCl_3$)** δ 171.3, 135.6, 134.7, 133.0, 128.6, 128.5, 128.0, 127.1, 126.5, 122.3, 121.5, 120.0, 117.5, 110.9, 109.8, 58.1, 51.8, 50.2, 49.2, 22.8. **HRMS (ESI-TOF)** calcd for $C_{22}H_{22}N_2O_2$ (M+H$^+$): 347.1754; Found: 347.1748.

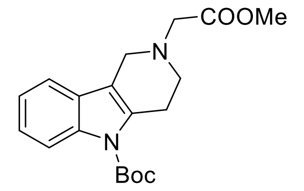

4aac

***tert*-butyl 2-(2-methoxy-2-oxoethyl)-1,2,3,4-tetrahydro-5*H*-pyrido[4,3-*b*]indole-5-carboxylate (4aac):** According to the general procedure, MeCN was replaced as solvent for 12 h, light yellow gel, 25 mg, 36% yield. **^{1}H NMR (400 MHz, CDCl$_3$)** δ 8.14 (d, *J* = 8.0 Hz, 1H), 7.31 (d, *J* = 7.6 Hz, 1H), 7.26 – 7.15 (m, 2H), 3.81 (s, 2H), 3.77 (s, 3H), 3.52 (s, 2H), 3.16 (t, *J* = 5.6 Hz, 2H), 2.99 (t, *J* = 5.6 Hz, 2H), 1.65 (s, 9H). **^{13}C NMR (101 MHz, CDCl$_3$)** δ 171.1, 150.5, 136.0, 133.3, 127.9, 123.7, 122.6, 117.3, 115.6, 114.5, 83.5, 58.5, 51.9, 50.7, 49.0, 28.3, 26.6. **HRMS (ESI-TOF)** calcd for $C_{19}H_{22}N_2O_4$ (M+H$^+$): 345.1809; Found: 345.1803.

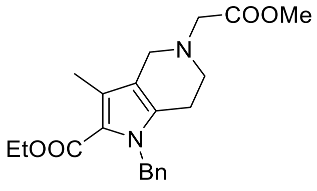

4aad

ethyl 1-benzyl-5-(2-methoxy-2-oxoethyl)-3-methyl-4,5,6,7-tetrahydro-1*H*-pyrrolo[3,2-*c*]pyridine-2-carboxylate (4aad): According to the procedure for 6 h, white solid, m.p. = 83 – 85 °C, 34 mg, 46% yield. **^{1}H NMR (400 MHz, CDCl$_3$)** δ 7.25 (m, 2H), 7.19 (m, 1H), 6.94 (m, 2H), 5.48 (s, 2H), 4.19 (q, *J* = 7.2 Hz, 2H), 3.74 (s, 3H), 3.58 (s, 2H), 3.46 (s, 2H), 2.89 (t, *J* = 5.6 Hz, 2H), 2.60 (t, *J* = 5.6 Hz, 2H), 2.22 (s, 3H), 1.25 (t, *J* = 7.2 Hz, 3H). **^{13}C NMR (101 MHz, CDCl$_3$)** δ 171.0, 162.1, 138.8, 133.0, 128.5, 126.8, 126.0, 125.8, 118.6, 116.6, 59.4, 58.7, 51.8, 50.2, 49.4, 48.3, 22.8, 14.4, 11.5. **HRMS (ESI-TOF)** calcd for $C_{21}H_{26}N_2O_4$ (M+H$^+$): 371.1965; Found: 371.1970.

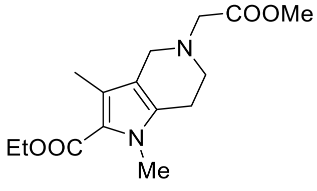

4aae

ethyl 5-(2-methoxy-2-oxoethyl)-1,3-dimethyl-4,5,6,7-tetrahydro-1*H*-pyrrolo[3,2-*c*]pyridine-2-carboxylate (4aae): According to the procedure for 6 h, light yellow gel, 38 mg, 65% yield. **^{1}H NMR (400 MHz, CDCl$_3$)** δ 4.34 – 4.18 (q, *J* = 6.8 Hz, 2H), 3.75 (d, *J* = 1.2 Hz, 3H), 3.70 (s, 3H), 3.56 (s, 2H), 3.47 (s, 2H), 2.95 (t, *J* = 5.2 Hz, 2H), 2.68 (t, *J* = 5.2 Hz, 2H), 2.16 (s, 3H), 1.39 – 1.31 (t, *J* = 6.8 Hz, 3H). **^{13}C NMR (101 MHz, CDCl$_3$)** δ 170.9, 162.4, 132.5, 125.1, 118.9, 115.7, 59.3, 58.5, 51.8, 50.2, 49.4, 32.6, 22.7, 14.5, 11.4. **HRMS (ESI-TOF)** calcd for $C_{15}H_{22}N_2O_4$ (M+H$^+$): 295.1652; Found: 295.1656.

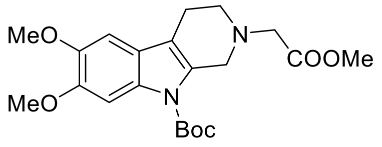

6a

***tert*-butyl 6,7-dimethoxy-2-(2-methoxy-2-oxoethyl)-1,2,3,4-tetrahydro-9*H*-pyrido[3,4-*b*]indole-9-carboxylate (6a):** According to the general procedure for 8 h, light yellow gel, 65 mg, 80% yield. **^{1}H NMR (400 MHz, CDCl$_3$)** δ 7.75 (s, 1H), 6.83 (s, 1H), 4.06 (s, 2H), 3.93 (m, 6H), 3.75 (s, 3H), 3.52 (s, 2H), 2.98 (t, *J* = 5.6 Hz, 2H), 2.74 (t, *J* = 5.6 Hz, 2H), 1.64 (s, 9H). **^{13}C NMR (101 MHz, CDCl$_3$)** δ 171.1, 150.3, 147.0, 146.2, 130.8, 129.7, 121.9, 114.4, 99.8, 99.7, 83.5, 58.0, 56.2, 56.1, 52.2, 51.9, 49.4, 28.3, 21.1. **HRMS (ESI-TOF)** calcd for $C_{21}H_{28}N_2O_6$ (M+H$^+$): 405.2020; Found: 405.2017.

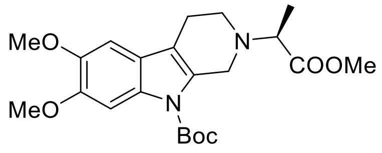

6b

***tert*-butyl (*S*)-6,7-dimethoxy-2-(1-methoxy-1-oxopropan-2-yl)-1,2,3,4-tetrahydro-9*H*-pyrido[3,4-*b*]indole-9-carboxylate (6b):** According to the general procedure for 8 h, light yellow solid, m.p. = 98 – 100 °C, 64 mg, 77% yield. **^{1}H NMR (400 MHz, CDCl$_3$)** δ 7.81 (s, 1H), 6.86 (s, 1H), 4.10 (s, 2H), 3.96 (d, *J* = 5.2 Hz, 6H), 3.77 (s, 3H), 3.67 (q, *J* = 7.2 Hz, 1H), 3.04 (dd, J_1 = 11.6 Hz, J_2 = 5.6 Hz, 1H), 2.92 (dd, J_1 = 11.6 Hz, J_2 = 6.0 Hz, 1H), 2.74 (d, *J* = 3.2 Hz, 2H), 1.69 (s, 9H), 1.48 (d, *J* = 7.2 Hz, 3H). **^{13}C NMR (101 MHz, CDCl$_3$)** δ 173.7, 150.3, 146.9,

146.2, 131.3, 129.9, 122.0, 114.9, 99.7, 99.7, 83.4, 61.9, 56.2, 56.1, 51.6, 49.3, 46.3, 28.3, 22.2, 15.2. **HRMS (ESI-TOF)** calcd for $C_{22}H_{30}N_2O_6$ (M+H$^+$): 419.2177; Found: 419.2175.

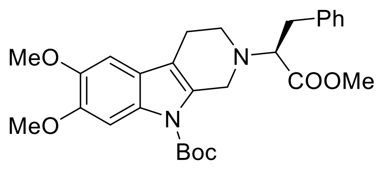

6c

***tert*-butyl (*S*)-6,7-dimethoxy-2-(1-methoxy-1-oxo-3-phenylpropan-2-yl)-1,2,3,4-tetrahydro-9*H*-pyrido[3,4-*b*]indole-9-carboxylate (6c):** According to the general procedure for 8 h, light yellow solid, m.p. = 116 – 118 °C, 62 mg, 63% yield. **^{1}H NMR (400 MHz, CDCl$_3$)** δ 7.83 (s, 1H), 7.36 – 7.19 (m, 5H), 6.87 (s, 1H), 4.15 (t, J = 7.2 Hz, 2H), 3.98 (d, J = 5.2 Hz, 6H), 3.78 (dd, J_1 = 9.2 Hz, J_2 = 5.6 Hz, 1H), 3.66 (s, 3H), 3.34 – 3.21 (m, 1H), 3.21 – 3.06 (m, 2H), 2.93 (m, 1H), 2.74 (d, J = 4.8 Hz, 2H), 1.70 (s, 9H). **^{13}C NMR (101 MHz, CDCl$_3$)** δ 172.0, 150.2, 147.0, 146.2, 138.0, 131.2, 129.9, 129.2, 128.5, 126.6, 121.9, 114.8, 99.7, 99.7, 83.4, 69.1, 56.2, 56.2, 51.3, 49.9, 46.3, 36.1, 28.3, 22.4. **HRMS (ESI-TOF)** calcd for $C_{28}H_{34}N_2O_6$ (M+H$^+$): 495.2490; Found: 495.2483.

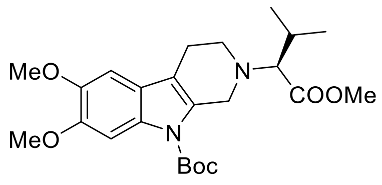

6d

***tert*-butyl (*S*)-6,7-dimethoxy-2-(1-methoxy-3-methyl-1-oxobutan-2-yl)-1,2,3,4-tetrahydro-9*H*-pyrido[3,4-*b*]indole-9-carboxylate (6d):** According to the general procedure for 8 h, light yellow solid, m.p. = 95 – 97 °C, 62 mg, 70% yield. **^{1}H NMR (400 MHz, CDCl$_3$)** δ 7.81 (s, 1H), 6.82 (s, 1H), 4.04 – 3.84 (m, 8H), 3.71 (s, 3H), 3.12 – 2.99 (m, 2H), 2.73 – 2.62 (m, 3H), 2.29 – 2.14 (m, 1H), 1.65 (s, 9H), 1.02 (d, J = 6.8 Hz, 3H), 0.93 (d, J = 6.4 Hz, 3H). **^{13}C NMR (101 MHz, CDCl$_3$)** δ 172.4, 150.2, 146.9, 146.2, 131.6, 129.9, 122.0, 115.0, 99.7, 99.7, 83.3, 74.0, 56.2, 50.8, 50.3, 45.6, 28.3, 27.4, 22.5, 19.9, 19.4. **HRMS (ESI-TOF)** calcd for $C_{24}H_{34}N_2O_6$ (M+H$^+$): 447.2490; Found: 447.2493.

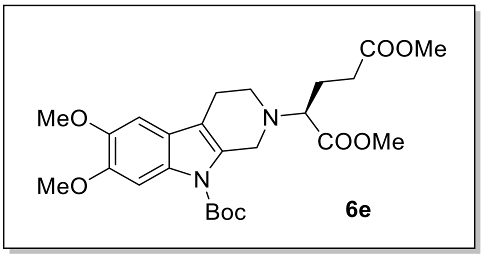

dimethyl (*S*)-2-(9-(*tert*-butoxycarbonyl)-6,7-dimethoxy-1,3,4,9-tetrahydro-2*H*-pyrido[3,4-*b*]indol-2-yl)pentanedioate (6e): According to the general procedure for 8 h, light yellow solid, m.p. = 125 – 127 °C, 52 mg, 53% yield. **^{1}H NMR (400 MHz, CDCl$_3$)** δ 7.78 (s, 1H), 6.82 (s, 1H), 4.10 (d, *J* = 16.0 Hz, 1H), 3.94 (m, 7H), 3.72 (s, 3H), 3.60 (s, 3H), 3.52 (m, 1H), 3.13 – 2.99 (m, 1H), 2.79 (m, 1H), 2.66 (d, *J* = 2.0 Hz, 2H), 2.44 (t, *J* = 7.2 Hz, 2H), 2.14 (m, 2H), 1.66 (s, 9H). **^{13}C NMR (101 MHz, CDCl$_3$)** δ 173.7, 172.4, 150.2, 146.9, 146.2, 131.5, 129.8, 121.9, 114.9, 99.7, 99.7, 83.5, 66.0, 56.2, 56.2, 51.6, 51.4, 49.5, 46.1, 30.7, 28.3, 24.4, 22.4. **HRMS (ESI-TOF)** calcd for $C_{25}H_{34}N_2O_8$ (M+H$^+$): 491.2388; Found: 491.2387.

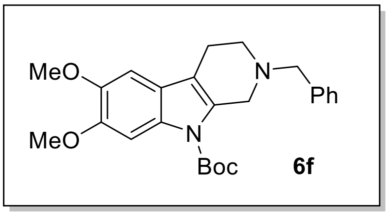

***tert*-butyl 2-benzyl-6,7-dimethoxy-1,2,3,4-tetrahydro-9*H*-pyrido[3,4-*b*]indole-9-carboxylate (6f):** According to the general procedure for 8 h, light yellow gel, 70 mg, 83% yield. **^{1}H NMR (400 MHz, CDCl$_3$)** δ 7.80 (s, 1H), 7.41 (d, *J* = 7.2 Hz, 2H), 7.34 (t, *J* = 7.2 Hz, 2H), 7.29 (d, *J* = 7.2 Hz, 1H), 6.83 (s, 1H), 3.94 (d, *J* = 5.2 Hz, 6H), 3.86 (s, 2H), 3.79 (s, 2H), 2.85 (t, *J* = 5.6 Hz, 2H), 2.72 (t, *J* = 5.6 Hz, 2H), 1.56 (s, 9H). **^{13}C NMR (101 MHz, CDCl$_3$)** δ 150.3, 147.0, 146.2, 138.4, 131.3, 130.0, 129.2, 128.4, 127.2, 122.0, 114.8, 99.8, 99.7, 83.3, 62.0, 56.2, 56.2, 52.8, 49.5, 28.2, 21.4. **HRMS (ESI-TOF)** calcd for $C_{25}H_{30}N_2O_4$ (M+H$^+$): 423.2278; Found: 423.2277.

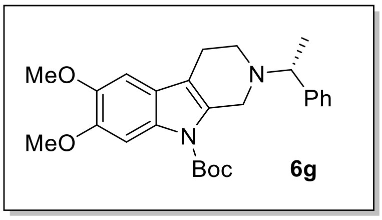

***tert*-butyl (*R*)-6,7-dimethoxy-2-(1-phenylethyl)-1,2,3,4-tetrahydro-9*H*-pyrido[3,4-*b*]indole-9-carboxylate (6g):** According to the general procedure for 8 h, light yellow gel, 56 mg, 64% yield. **^{1}H NMR (400 MHz, CDCl$_3$)** δ 7.82 (s, 1H), 7.43 – 7.30 (m, 4H), 7.26 (t, *J* = 7.2 Hz, 1H), 6.82 (s, 1H), 4.04 (d, *J* = 16.0 Hz, 1H), 3.94 (d, *J* = 8.4 Hz, 6H), 3.82 – 3.63 (m, 2H), 2.90 (m, 1H), 2.77 – 2.54 (m, 3H), 1.56 (s, 9H), 1.51 (d, *J* = 6.4 Hz, 3H). **^{13}C NMR (101 MHz, CDCl$_3$)** δ 150.3, 146.9, 146.2, 144.4, 131.6, 130.1, 128.5, 127.6, 127.1, 121.9, 115.0, 99.7, 99.7, 83.2, 63.8, 56.2, 56.2, 50.6, 47.0, 28.3, 21.6, 20.6. **HRMS (ESI-TOF)** calcd for $C_{26}H_{32}N_2O_4$ (M+H$^+$): 437.2435; Found: 437.2430.

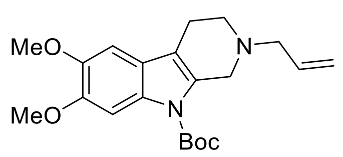

6h

***tert*-butyl 2-allyl-6,7-dimethoxy-1,2,3,4-tetrahydro-9*H*-pyrido[3,4-*b*]indole-9-carboxylate (6h):** According to the general procedure for 8 h, light yellow gel, 68 mg, 91% yield. **^{1}H NMR (400 MHz, CDCl$_3$)** δ 7.83 (s, 1H), 6.87 (s, 1H), 6.04 (m, 1H), 5.37 – 5.23 (m, 2H), 3.97 (d, *J* = 5.2 Hz, 8H), 3.37 (d, *J* = 6.8 Hz, 2H), 2.94 (t, *J* = 5.6 Hz, 2H), 2.79 (t, *J* = 5.6 Hz, 2H), 1.68 (s, 9H). **^{13}C NMR (101 MHz, CDCl$_3$)** δ 150.3, 147.1, 146.3, 137.0, 134.5, 130.5, 130.0, 121.8, 118.9, 114.6, 99.8, 83.5, 60.3, 56.2, 56.2, 52.1, 49.1, 28.3, 20.9. **HRMS (ESI-TOF)** calcd for $C_{21}H_{28}N_2O_4$ (M+H$^+$): 373.2122; Found: 373.2117.

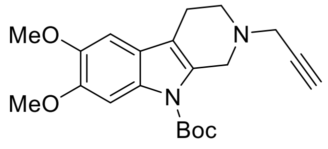

6i

***tert*-butyl 6,7-dimethoxy-2-(prop-2-yn-1-yl)-1,2,3,4-tetrahydro-9*H*-pyrido[3,4-*b*]indole-9-carboxylate (6i):** According to the general procedure for 8 h, light yellow gel, 67 mg, 90% yield. **^{1}H NMR (400 MHz, CDCl$_3$)** δ 7.77 (s, 1H), 6.83 (s, 1H), 4.02 (s, 2H), 3.93 (d, *J* = 4.0 Hz, 6H), 3.58 (d, *J* = 2.0 Hz, 2H), 2.91 (t, *J* = 5.6 Hz, 2H), 2.76 (t, *J* = 5.2 Hz, 2H), 2.29 (s, 1H), 1.66 (s, 9H). **^{13}C NMR (101 MHz, CDCl$_3$)** δ 150.3, 147.0, 146.2, 131.0, 129.9, 121.9, 114.4, 99.8, 99.7,

83.5, 78.8, 73.4, 56.2, 56.2, 51.3, 48.7, 46.3, 28.3, 21.5. **HRMS (ESI-TOF)** calcd for $C_{21}H_{26}N_2O_4$ (M+H$^+$): 371.1965; Found: 371.1963.

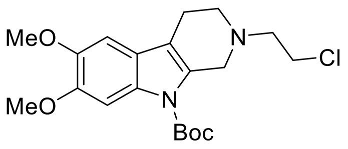

6j

***tert*-butyl 2-(2-chloroethyl)-6,7-dimethoxy-1,2,3,4-tetrahydro-9*H*-pyrido[3,4-*b*]indole-9-carboxylate (6j):** According to the general procedure for 8 h, light yellow solid, m.p. = 105 – 107 °C, 46 mg, 59% yield. **^{1}H NMR (400 MHz, CDCl$_3$)** δ 7.75 (s, 1H), 6.83 (s, 1H), 4.04 (d, *J* = 11.6 Hz, 2H), 3.93 (d, *J* = 3.6 Hz, 6H), 3.72 (t, *J* = 6.8 Hz, 2H), 3.03 (t, *J* = 6.8 Hz, 2H), 2.95 (t, *J* = 5.6 Hz, 2H), 2.74 (s, 2H), 1.66 (s, 9H). **^{13}C NMR (101 MHz, CDCl$_3$)** δ 150.3, 147.0, 146.3, 130.9, 129.7, 121.9, 114.7, 99.8, 99.7, 83.6, 58.5, 56.2, 56.1, 52.5, 49.7, 41.6, 28.3, 21.1. **HRMS (ESI-TOF)** calcd for $C_{20}H_{27}N_2O_4$ (M+H$^+$): 395.1732; Found: 395.1729.

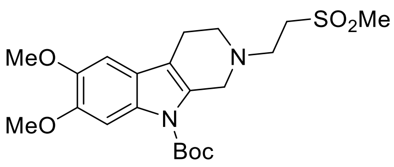

6k

***tert*-butyl 6,7-dimethoxy-2-(2-(methylsulfonyl)ethyl)-1,2,3,4-tetrahydro-9*H*-pyrido[3,4-*b*]indole-9-carboxylate (6k):** According to the general procedure for 8 h, light yellow solid, m.p. = 68 – 70 °C, 57 mg, 65% yield. **^{1}H NMR (400 MHz, CDCl$_3$)** δ 7.73 (s, 1H), 6.83 (s, 1H), 3.95 (t, *J* = 7.6 Hz, 8H), 3.26 (t, *J* = 6.0 Hz, 2H), 3.16 (t, *J* = 6.0 Hz, 2H), 3.04 (s, 3H), 2.90 (t, *J* = 5.6 Hz, 2H), 2.73 (t, *J* = 5.6 Hz, 2H), 1.68 (s, 9H). **^{13}C NMR (101 MHz, CDCl$_3$)** δ 150.3, 147.2, 146.4, 130.6, 129.7, 121.7, 114.6, 99.9, 99.7, 83.8, 56.2, 56.2, 52.8, 52.3, 50.8, 49.7, 42.6, 28.3, 21.2. **HRMS (ESI-TOF)** calcd for $C_{21}H_{30}N_2O_6S$ (M+H$^+$): 439.1897; Found: 439.1893.

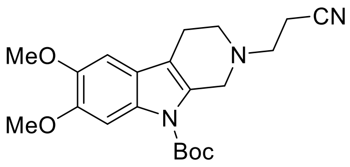

6l

***tert*-butyl 2-(2-cyanoethyl)-6,7-dimethoxy-1,2,3,4-tetrahydro-9*H*-pyrido[3,4-*b*]indole-9-carboxylate (6l):** According to the general procedure for 8 h, light yellow gel, 67 mg, 87% yield. **^{1}H NMR (400 MHz, CDCl$_3$)** δ 7.78 (s, 1H), 6.87 (s, 1H), 4.02 (s, 2H), 3.97 (d, *J* = 2.4 Hz, 6H), 3.01 (t, *J* = 7.2 Hz, 2H), 2.95 (t, *J* = 5.6 Hz, 2H), 2.77 (t, *J* = 5.2 Hz, 2H), 2.67 (t, *J* = 7.2 Hz, 2H), 1.71 (s, 9H). **^{13}C NMR (101 MHz, CDCl$_3$)** δ 150.3, 147.1, 146.3, 130.6, 129.7, 121.8, 118.8, 114.7, 99.8, 99.7, 83.7, 56.2, 56.1, 52.4, 52.1, 49.4, 28.3, 21.1, 16.6. **HRMS (ESI-TOF)** calcd for $C_{21}H_{27}N_3O_4$ (M+H$^+$): 386.2074; Found: 386.2069.

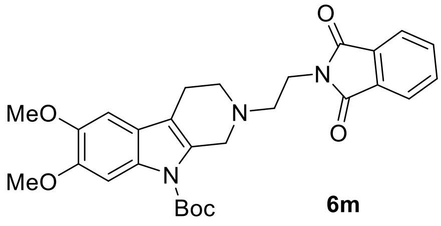

6m

***tert*-butyl 2-(2-(1,3-dioxoisoindolin-2-yl)ethyl)-6,7-dimethoxy-1,2,3,4-tetrahydro-9*H*-pyrido[3,4-*b*]indole-9-carboxylate (6m):** According to the general procedure for 8 h, light yellow gel, 71 mg, 70% yield. **^{1}H NMR (400 MHz, CDCl$_3$)** δ 7.87 – 7.74 (m, 3H), 7.68 (m, 2H), 6.81 (s, 1H), 4.00 – 3.84 (m, 10H), 2.92 (t, *J* = 6.0 Hz, 4H), 2.67 (s, 2H), 1.66 (s, 9H). **^{13}C NMR (101 MHz, CDCl$_3$)** δ 168.4, 150.3, 147.0, 146.2, 133.9, 132.2, 131.1, 129.8, 123.2, 121.9, 114.8, 99.7, 99.7, 83.5, 56.2, 54.4, 52.9, 49.1, 35.7, 28.3, 21.1. **HRMS (ESI-TOF)** calcd for $C_{28}H_{31}N_3O_6$ (M+H$^+$): 506.2286; Found: 506.2286.

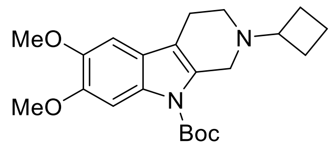

6n

***tert*-butyl 2-cyclobutyl-6,7-dimethoxy-1,2,3,4-tetrahydro-9*H*-pyrido[3,4-*b*]indole-9-carboxylate (6n):** According to the general procedure for 8 h, light yellow gel, 62 mg, 80% yield. **^{1}H NMR (400 MHz, CDCl$_3$)** δ 7.76 (s, 1H), 6.82 (s, 1H), 3.92 (m, 8H), 3.27 – 3.07 (m, 1H), 2.80 (s, 4H), 2.17 (m, 4H), 1.90 – 1.72 (m, 2H), 1.65 (s, 9H). **^{13}C NMR (101 MHz, CDCl$_3$)** δ 150.2, 147.2, 146.3, 136.0, 129.9, 121.7, 114.6, 99.8, 99.7, 83.6, 59.3, 56.2, 56.1, 49.0, 45.7, 28.3, 27.3, 20.6, 14.4. **HRMS (ESI-TOF)** calcd for $C_{22}H_{30}N_2O_4$ (M+H$^+$): 387.2278; Found: 387.2274.

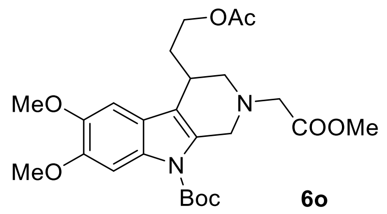

6o

***tert*-butyl 4-(2-acetoxyethyl)-6,7-dimethoxy-2-(2-methoxy-2-oxoethyl)-1,2,3,4-tetrahydro-9*H*-pyrido[3,4-*b*]indole-9-carboxylate (6o):** According to the general procedure for 8 h, light yellow gel, 75 mg, 77% yield. **^{1}H NMR (400 MHz, CDCl$_3$)** δ 7.80 (s, 1H), 6.94 (s, 1H), 4.44 – 4.16 (m, 3H), 3.97 (s, 6H), 3.86 (d, *J* = 16.43 Hz, 1H), 3.78 (s, 3H), 3.55 (q, *J* = 16.4 Hz, 2H), 3.04 (s, 1H), 2.90 (d, *J* = 6.0 Hz, 2H), 2.31 – 2.08 (m, 5H), 1.69 (s, 9H). **^{13}C NMR (101 MHz, CDCl$_3$)** δ 171.2, 171.1, 150.2, 147.0, 146.2, 131.5, 130.0, 121.3, 117.6, 100.1, 100.0, 83.7, 63.2, 58.5, 56.2, 56.2, 53.8, 52.4, 51.7, 32.2, 30.0, 28.3, 21.1. **HRMS (ESI-TOF)** calcd for $C_{25}H_{34}N_2O_8$ (M+H$^+$): 491.2388; Found: 491.2383.

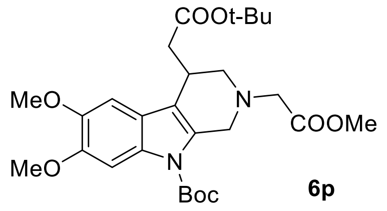

6p

***tert*-butyl 4-(2-(*tert*-butoxy)-2-oxoethyl)-6,7-dimethoxy-2-(2-methoxy-2-oxoethyl)-1,2,3,4-tetrahydro-9*H*-pyrido[3,4-*b*]indole-9-carboxylate (6p):** According to the general procedure for 8 h, light yellow gel, 56 mg, 54% yield. **^{1}H NMR (600 MHz, CDCl$_3$)** δ 7.77 (s, 1H), 6.92 (s, 1H), 4.20 (d, *J* = 16.0 Hz, 1H), 3.94 (s, 6H), 3.83 (d, *J* = 16.0 Hz, 1H), 3.74 (s, 3H), 3.59 – 3.43 (m, 2H), 3.34 (d, *J* = 4.8 Hz, 1H), 2.97 – 2.83 (m, 2H), 2.79 – 2.63 (m, 2H), 1.65 (s, 9H), 1.48 (s, 9H). **^{13}C NMR (151 MHz, CDCl$_3$)** δ 172.5, 171.0, 150.2, 147.0, 146.3, 120.9, 117.3, 100.2, 99.9, 83.6, 80.5, 58.5, 56.4, 56.1, 54.1, 52.3, 51.6, 38.8, 29.9, 28.3, 28.2. **HRMS (ESI-TOF)** calcd for $C_{27}H_{38}N_2O_8$ (M+H$^+$): 519.2701; Found: 519.2695.

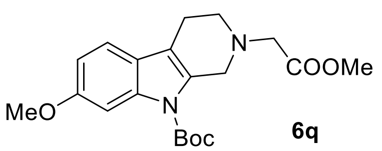

6q

***tert*-butyl 7-methoxy-2-(2-methoxy-2-oxoethyl)-1,2,3,4-tetrahydro-9*H*-pyrido[3,4-*b*]indole-9-carboxylate (6q):** According to the general procedure for 8 h, light yellow gel, 42 mg, 56% yield. **^{1}H NMR (400 MHz, CDCl$_3$)** δ 7.73 (s, 1H), 7.27 (d, *J* = 6.0 Hz, 1H), 6.86 (dd, J_1 = 8.4 Hz, J_2 = 2.0 Hz, 1H), 4.08 (s, 2H), 3.86 (s, 3H), 3.76 (s, 3H), 3.52 (s, 2H), 2.98 (t, *J* = 5.6 Hz, 2H), 2.76 (t, *J* = 5.6 Hz, 2H), 1.66 (s, 9H). **^{13}C NMR (101 MHz, CDCl$_3$)** δ 171.2, 157.4, 150.4, 136.7, 131.1, 123.2, 118.1, 114.5, 111.4, 100.3, 83.7, 58.1, 55.7, 52.1, 51.8, 49.4, 28.3, 21.0. **HRMS (ESI-TOF)** calcd for $C_{20}H_{26}N_2O_5$ (M+H$^+$): 375.1914; Found: 375.1911.

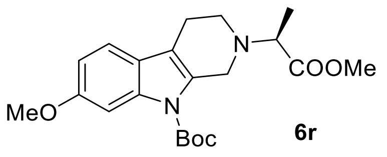

6r

***tert*-butyl (*S*)-7-methoxy-2-(1-methoxy-1-oxopropan-2-yl)-1,2,3,4-tetrahydro-9*H*-pyrido [3,4-*b*]indole-9-carboxylate (6r):** According to the general procedure for 8 h, light yellow gel, 33 mg, 43% yield. **^{1}H NMR (400 MHz, CDCl3)** δ 7.75 (d, *J* = 1.6 Hz, 1H), 7.25 (d, *J* = 8.0 Hz, 1H), 6.85 (dd, J_1 = 8.4 Hz, J_2 = 2.4 Hz, 1H), 4.07 (s, 2H), 3.86 (s, 3H), 3.74 (s, 3H), 3.64 (q, *J* = 7.2 Hz, 1H), 2.93 (m, 2H), 2.71 (m, 2H), 1.66 (s, 9H), 1.44 (d, *J* = 7.2 Hz, 3H). **^{13}C NMR (101 MHz, CDCl3)** δ 173.7, 157.3, 150.3, 136.8, 131.6, 123.2, 118.1, 114.9, 111.4, 100.3, 83.5, 62.0, 55.7, 51.5, 49.3, 46.3, 28.3, 22.1, 15.2. **HRMS (ESI-TOF)** calcd for $C_{21}H_{28}N_2O_5$ (M+H$^+$): 389.2071; Found: 389.2073.

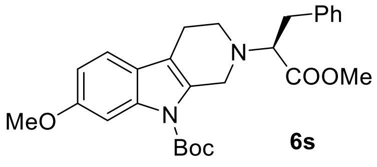

6s

***tert*-butyl (*S*)-7-methoxy-2-(1-methoxy-1-oxo-3-phenylpropan-2-yl)-1,2,3,4-tetrahydro-9*H*-pyrido[3,4-*b*]indole-9-carboxylate (6s):** According to the general procedure for 8 h, light yellow gel, 42 mg, 45% yield. **^{1}H NMR (400 MHz, CDCl$_3$)** δ 7.81 (s, 1H), 7.29 (m, 6H), 6.90 (dd, J_1 = 8.4 Hz, J_2 = 2.0 Hz, 1H), 4.29 – 4.06 (m, 2H), 3.91 (s, 3H), 3.79 (dd, J_1 = 9.2 Hz, J_2 = 5.6 Hz, 1H), 3.66 (d, *J* = 4.4 Hz, 3H), 3.27 (m, 1H), 3.21 – 3.07 (m, 2H), 2.93 (m, 1H), 2.84 – 2.66 (m, 2H), 1.71 (s, 9H). **^{13}C NMR (101 MHz, CDCl$_3$)** δ 172.0, 157.4, 150.3, 138.1, 136.8,

131.5, 129.2, 128.5, 126.6, 123.2, 118.1, 114.9, 111.5, 100.2, 83.6, 69.1, 55.7, 51.3, 49.9, 46.3, 36.1, 28.3, 22.3. **HRMS (ESI-TOF)** calcd for $C_{27}H_{32}N_2O_5$ (M+H$^+$): 465.2384; Found: 465.2389.

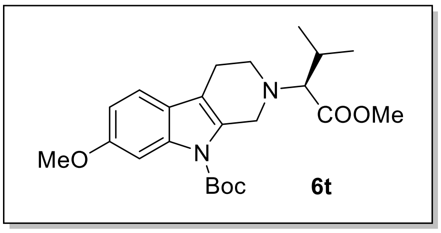

***tert*-butyl (*S*)-7-methoxy-2-(1-methoxy-3-methyl-1-oxobutan-2-yl)-1,2,3,4-tetrahydro-9*H*-pyrido[3,4-*b*]indole-9-carboxylate (6t):** According to the general procedure for 8 h, light yellow gel, 29 mg, 35% yield. **^{1}H NMR (400 MHz, CDCl$_3$)** δ 7.77 (s, 1H), 7.25 (d, *J* = 8.4 Hz, 1H), 6.85 (dd, J_1 = 8.4 Hz, J_2 = 2.0 Hz, 1H), 4.07 – 3.79 (m, 5H), 3.72 (d, *J* = 9.2 Hz, 3H), 3.04 (t, *J* = 10.4 Hz, 2H), 2.68 (d, *J* = 7.6 Hz, 3H), 2.28 – 2.15 (m, 1H), 1.66 (s, 9H), 1.02 (d, *J* = 6.4 Hz, 3H), 0.93 (d, *J* = 6.4 Hz, 3H). **^{13}C NMR (101 MHz, CDCl$_3$)** δ 172.4, 157.3, 150.3, 136.8, 131.9, 123.2, 118.0, 115.0, 111.5, 100.2, 83.5, 74.0, 55.7, 50.8, 50.2, 45.5, 28.3, 27.4, 22.4, 19.9, 19.4. **HRMS (ESI-TOF)** calcd for $C_{23}H_{32}N_2O_5$ (M+H$^+$): 417.2384; Found: 417.2386.

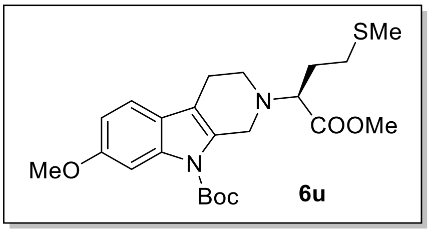

***tert*-butyl (*S*)-7-methoxy-2-(1-methoxy-4-(methylthio)-1-oxobutan-2-yl)-1,2,3,4-tetrahydro-9*H*-pyrido[3,4-*b*]indole-9-carboxylate (6u):** According to the general procedure for 8 h, light yellow gel, 36 mg, 40% yield. **^{1}H NMR (400 MHz, CDCl$_3$)** δ 7.74 (s, 1H), 7.25 (d, *J* = 8.2 Hz, 1H), 6.85 (dd, J_1 = 8.4 Hz, J_2 = 2.0 Hz, 1H), 4.06 (dd, J_1 = 39.2, J_2 = 16.0 Hz, 2H), 3.86 (s, 3H), 3.73 (s, 3H), 3.68 (t, *J* = 7.6 Hz, 1H), 3.10 – 3.01 (m, 1H), 2.87 – 2.78 (m, 1H), 2.68 (d, *J* = 5.2 Hz, 2H), 2.60 (t, *J* = 7.2 Hz, 2H), 2.17 – 2.05 (m, 5H), 1.66 (s, 9H). **^{13}C NMR (101 MHz, CDCl$_3$)** δ 172.6, 157.3, 150.3, 136.7, 131.7, 123.2, 118.1, 114.9, 111.4, 100.3, 83.6, 65.3, 55.7, 51.4, 49.5, 46.0, 30.9, 28.8, 28.3, 22.4, 15.6. **HRMS (ESI-TOF)** calcd for $C_{23}H_{32}N_2O_5S$ (M+H$^+$): 449.2105; Found: 449.2107.

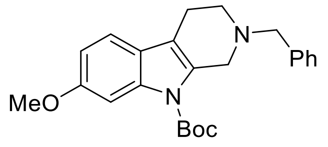

6v

***tert*-butyl 2-benzyl-7-methoxy-1,2,3,4-tetrahydro-9*H*-pyrido[3,4-*b*]indole-9-carboxylate (6v):** According to the general procedure for 8 h, off-white solid, m.p. = 98 – 100 °C, 72 mg, 92% yield. **^{1}H NMR (400 MHz, $CDCl_3$)** δ 7.80 (s, 1H), 7.50 – 7.21 (m, 8H), 6.89 (dd, J_1 = 8.4 Hz, J_2 = 2.0 Hz, 1H), 3.90 (s, 5H), 3.83 (s, 2H), 2.88 (t, J = 5.2 Hz, 2H), 2.76 (t, J = 5.2 Hz, 2H), 1.61 (s, 9H). **^{13}C NMR (101 MHz, $CDCl_3$)** δ 157.4, 150.3, 138.4, 136.9, 131.6, 129.2, 128.4, 127.3, 123.2, 118.1, 114.8, 111.5, 100.2, 83.5, 62.0, 55.7, 52.7, 49.4, 28.2, 21.3. **HRMS (ESI-TOF)** calcd for $C_{24}H_{28}N_2O_3$ (M+H$^+$): 393.2173; Found: 393.2172.

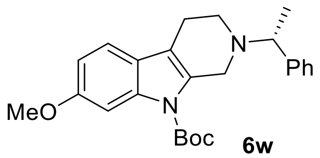

6w

***tert*-butyl (*R*)-7-methoxy-2-(1-phenylethyl)-1,2,3,4-tetrahydro-9*H*-pyrido[3,4-*b*]indole-9-carboxylate (6w):** According to the general procedure for 8 h, light yellow gel, 44 mg, 54% yield. **^{1}H NMR (400 MHz, $CDCl_3$)** δ 7.83 (d, J = 2.0 Hz, 1H), 7.45 – 7.40 (m, 2H), 7.37 (t, J = 7.6 Hz, 2H), 7.32 (m, 1H), 7.29 – 7.25 (m, 1H), 6.88 (dd, J_1 = 8.4 Hz, J_2 = 2.4 Hz, 1H), 4.08 (d, J = 16.4 Hz, 1H), 3.90 (s, 3H), 3.81 (d, J = 16.4 Hz, 1H), 3.74 (q, J = 6.8 Hz, 1H), 3.02 – 2.85 (m, 1H), 2.80 – 2.60 (m, 3H), 1.61 (s, 9H), 1.54 (d, J = 6.8 Hz, 3H). **^{13}C NMR (101 MHz, $CDCl_3$)** δ 157.3, 150.4, 144.5, 137.0, 131.9, 128.4, 127.6, 127.1, 123.2, 118.0, 115.1, 111.5, 100.2, 83.3, 63.8, 55.7, 50.6, 46.9, 28.3, 21.6, 20.5. **HRMS (ESI-TOF)** calcd for $C_{25}H_{30}N_2O_3$ (M+H$^+$): 407.2329; Found: 407.2331.

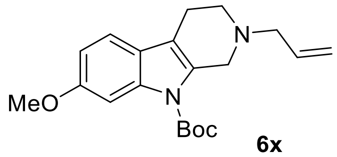

6x

***tert*-butyl 2-allyl-7-methoxy-1,2,3,4-tetrahydro-9*H*-pyrido[3,4-*b*]indole-9-carboxylate (6x):** According to the general procedure for 8 h, light yellow gel, 52 mg, 76% yield. **^{1}H NMR (400 MHz, $CDCl_3$)** δ 7.77 (d, J = 2.0 Hz, 1H), 7.26 (d, J = 8.4 Hz, 1H), 6.85 (dd, J_1 = 8.4 Hz, J_2 =2.4

Hz, 1H), 6.11 – 5.89 (m, 1H), 5.34 – 5.15 (m, 2H), 3.94 – 3.82 (m, 5H), 3.28 (d, J = 6.4 Hz, 2H), 2.82 (t, J = 5.6 Hz, 2H), 2.73 (m, 2H), 1.64 (s, 9H). **^{13}C NMR (101 MHz, CDCl$_3$)** δ 157.4, 150.4, 136.9, 135.5, 131.6, 123.2, 118.1, 118.0, 114.8, 111.4, 100.3, 83.4, 60.8, 55.7, 52.6, 49.4, 28.3, 21.3. **HRMS (ESI-TOF)** calcd for $C_{20}H_{26}N_2O_3$ (M+H$^+$): 343.2016; Found: 343.2020.

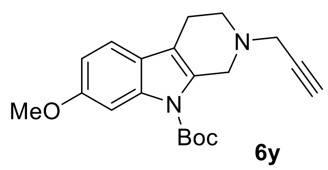

6y

***tert*-butyl 7-methoxy-2-(prop-2-yn-1-yl)-1,2,3,4-tetrahydro-9*H*-pyrido[3,4-*b*]indole-9-carboxylate (6y):** According to the general procedure for 8 h, light yellow gel, 44 mg, 65% yield. **^{1}H NMR (400 MHz, CDCl$_3$)** δ 7.74 (s, 1H), 7.27 (d, J = 7.2 Hz, 1H), 6.86 (dd, J_1 = 8.4 Hz, J_2 = 2.0 Hz, 1H), 4.03 (s, 2H), 3.87 (s, 3H), 3.58 (d, J = 2.0 Hz, 2H), 2.90 (t, J = 5.6 Hz, 2H), 2.76 (t, J = 5.6 Hz, 2H), 2.29 (s, 1H), 1.66 (s, 9H). **^{13}C NMR (101 MHz, CDCl$_3$)** δ 157.4, 150.4, 136.8, 131.4, 123.1, 118.1, 114.4, 111.4, 100.3, 83.6, 78.9, 73.3, 55.7, 51.3, 48.7, 46.3, 28.3, 21.5. **HRMS (ESI-TOF)** calcd for $C_{20}H_{24}N_2O_3$ (M+H$^+$): 341.1860; Found: 341.1859.

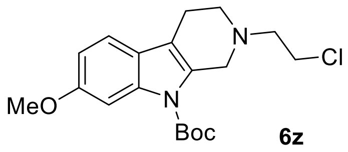

6z

***tert*-butyl 2-(2-chloroethyl)-7-methoxy-1,2,3,4-tetrahydro-9*H*-pyrido[3,4-*b*]indole-9-carboxylate (6z):** According to the general procedure for 8 h, light yellow gel, 33 mg, 46% yield. **^{1}H NMR (400 MHz, CDCl$_3$)** δ 7.72 (d, J = 1.6 Hz, 1H), 7.26 (d, J = 8.4 Hz, 1H), 6.86 (dd, J_1 = 8.4 Hz, J_2 = 2.4 Hz, 1H), 4.01 (s, 2H), 3.86 (s, 3H), 3.70 (t, J = 6.8 Hz, 2H), 3.01 (t, J = 6.8 Hz, 2H), 2.92 (t, J = 5.6 Hz, 2H), 2.74 (t, J = 5.6 Hz, 2H), 1.67 (s, 9H). **^{13}C NMR (101 MHz, CDCl$_3$)** δ 157.4, 150.4, 136.7, 131.2, 123.1, 118.1, 114.8, 111.4, 100.4, 83.7, 58.6, 55.7, 52.5, 49.7, 41.6, 28.3, 21.0. **HRMS (ESI-TOF)** calcd for $C_{19}H_{25}N_2O_3Cl$ (M+H$^+$): 365.1626; Found: 365.1628.

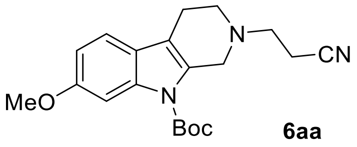

6aa

***tert*-butyl 2-(2-cyanoethyl)-7-methoxy-1,2,3,4-tetrahydro-9*H*-pyrido[3,4-*b*]indole-9-**

carboxylate (6aa): According to the general procedure for 8 h, light yellow gel, 31 mg, 43% yield. **^{1}H NMR (400 MHz, CDCl$_3$)** δ 7.71 (d, J = 1.6 Hz, 1H), 7.27 (d, J = 8.4 Hz, 1H), 6.86 (dd, J_1 = 8.4 Hz, J_2 = 2.4 Hz, 1H), 3.99 (d, J = 1.6 Hz, 2H), 3.86 (s, 3H), 2.98 (t, J = 7.2 Hz, 2H), 2.90 (t, J = 5.6 Hz, 2H), 2.73 (dd, J_1 = 6.4 Hz, J_2 = 4.8 Hz, 2H), 2.63 (t, J = 7.2 Hz, 2H), 1.68 (s, 9H). **^{13}C NMR (101 MHz, CDCl$_3$)** δ 157.5, 150.4, 136.6, 131.0, 123.0, 118.7, 118.2, 114.7, 111.4, 100.5, 83.8, 55.7, 52.4, 52.1, 49.4, 28.3, 21.1, 16.7. **HRMS (ESI-TOF)** calcd for $C_{20}H_{25}N_3O_3$ (M+H$^+$): 356.1969; Found: 356.1969

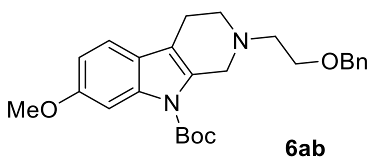

6ab

***tert*-butyl 2-(2-(benzyloxy)ethyl)-7-methoxy-1,2,3,4-tetrahydro-9*H*-pyrido[3,4-*b*]indole-9-carboxylate (6ab):** According to the general procedure for 8 h, light yellow gel, 55 mg, 63% yield. **^{1}H NMR (400 MHz, CDCl$_3$)** δ 7.76 (d, J = 1.6 Hz, 1H), 7.39 – 7.31 (m, 4H), 7.31 – 7.24 (m, 2H), 6.86 (dd, J_1 = 8.4 Hz, J_2 = 2.4 Hz, 1H), 4.58 (s, 2H), 3.98 (s, 2H), 3.87 (s, 3H), 3.71 (t, J = 5.6 Hz, 2H), 2.90 (m, 4H), 2.72 (t, J = 5.6 Hz, 2H), 1.63 (s, 9H). **^{13}C NMR (101 MHz, CDCl$_3$)** δ 157.3, 150.4, 138.4, 136.8, 131.7, 128.4, 127.8, 127.6, 123.3, 118.1, 114.8, 111.4, 100.3, 83.5, 73.3, 68.5, 56.8, 55.7, 53.0, 50.1, 28.3, 21.2. **HRMS (ESI-TOF)** calcd for $C_{26}H_{32}N_2O_4$ (M+H$^+$): 437.2435; Found: 437.2436.

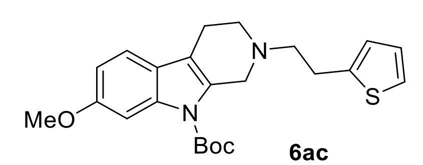

6ac

***tert*-butyl 7-methoxy-2-(2-(thiophen-2-yl)ethyl)-1,2,3,4-tetrahydro-9*H*-pyrido[3,4-*b*]indole-9-carboxylate (6ac):** According to the general procedure for 8 h, light yellow gel, 30 mg, 36% yield. **^{1}H NMR (400 MHz, CDCl$_3$)** δ 7.74 (s, 1H), 7.28 (d, J = 8.4 Hz, 1H), 7.14 (d, J = 5.2 Hz, 1H), 6.94 (dd, J_1 = 5.2 Hz, J_2 = 3.6 Hz, 1H), 6.91 – 6.81 (m, 2H), 3.99 (s, 2H), 3.87 (s, 3H), 3.26 – 3.11 (m, 2H), 3.00 – 2.86 (m, 4H), 2.75 (t, J = 5.2 Hz, 2H), 1.67 (s, 9H). **^{13}C NMR (101 MHz, CDCl$_3$)** δ 157.3, 150.4, 142.7, 136.7, 131.6, 126.7, 124.7, 123.6, 123.2, 118.1, 114.9, 111.4, 100.4, 83.6, 59.1, 55.7, 52.6, 49.7, 28.4, 28.3, 21.4. **HRMS (ESI-TOF)** calcd for $C_{23}H_{28}N_2O_3S$ (M+H$^+$): 413.1893; Found: 413.1896.

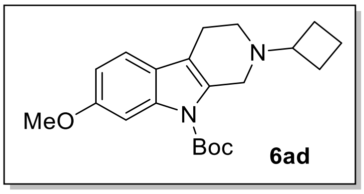

***tert*-butyl 2-cyclobutyl-7-methoxy-1,2,3,4-tetrahydro-9*H*-pyrido[3,4-*b*]indole-9-carboxylate (6ad):** According to the general procedure for 8 h, light yellow gel, 36 mg, 50% yield. **^{1}H NMR (400 MHz, CDCl$_3$)** δ 7.75 (s, 1H), 7.25 (d, J = 8.0 Hz, 1H), 6.84 (dd, J_1 = 8.4 Hz, J_2 = 1.6 Hz, 1H), 3.86 (s, 3H), 3.76 (s, 2H), 3.04 (dd, J_1 = 15.6 Hz, J_2 = 7.6 Hz, 1H), 2.68 (m, 4H), 2.14 (dd, J_1 = 15.6 Hz, J_2 = 8.0 Hz, 2H), 2.05 – 1.93 (m, 2H), 1.75 (m, 2H), 1.64 (s, 9H). **^{13}C NMR (101 MHz, CDCl$_3$)** δ 157.3, 150.4, 136.8, 131.5, 123.3, 118.1, 114.9, 111.4, 100.2, 83.4, 59.4, 55.7, 49.3, 45.6, 28.3, 27.8, 21.3, 14.5. **HRMS (ESI-TOF)** calcd for $C_{21}H_{28}N_2O_3$ (M+H$^+$): 357.2173; Found: 357.2175.

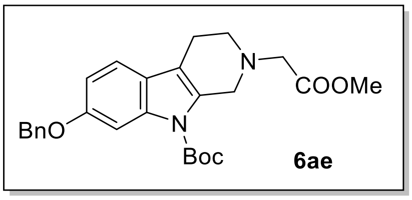

***tert*-butyl 7-(benzyloxy)-2-(2-methoxy-2-oxoethyl)-1,2,3,4-tetrahydro-9*H*-pyrido[3,4-*b*]indole-9-carboxylate (6ae):** According to the general procedure for 8 h, light yellow gel, 31 mg, 35% yield. **^{1}H NMR (400 MHz, CDCl$_3$)** δ 7.83 (s, 1H), 7.46 (m, J = 7.6 Hz, 2H), 7.38 (m, 2H), 7.30 (m, 2H), 6.94 (m, 1H), 5.12 (s, 2H), 4.08 (s, 2H), 3.76 (s, 3H), 3.52 (s, 2H), 2.98 (t, J = 5.6 Hz, 2H), 2.76 (t, J = 5.6 Hz, 2H), 1.64 (s, 9H). **^{13}C NMR (101 MHz, CDCl$_3$)** δ 171.1, 156.6, 150.3, 137.3, 136.7, 131.2, 128.5, 127.9, 127.6, 123.5, 118.1, 114.5, 112.2, 101.7, 83.7, 70.6, 58.1, 52.1, 51.8, 49.4, 28.3, 21.0. **HRMS (ESI-TOF)** calcd for $C_{26}H_{30}N_2O_5$ (M+H$^+$): 451.2227; Found: 451.2225.

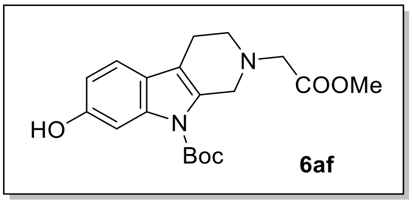

***tert*-butyl 7-hydroxy-2-(2-methoxy-2-oxoethyl)-1,2,3,4-tetrahydro-9*H*-pyrido[3,4-*b*]indole-9-carboxylate (6af):** According to the general procedure for 8 h, light yellow gel, 14 mg, 20% yield. **^{1}H NMR (400 MHz, CDCl$_3$)** δ 7.61 (s, 1H), 7.19 (d, J = 8.0 Hz, 1H), 6.76 (d, J = 8.0 Hz, 1H), 4.07 (s, 2H), 3.76 (s, 3H), 3.53 (s, 2H), 2.99 (m, 2H), 2.75 (m 2H), 1.64 (s, 9H). **^{13}C NMR**

(101 MHz, $CDCl_3$) δ 171.0, 153.2, 150.3, 136.7, 130.9, 123.3, 118.2, 114.5, 111.5, 102.8, 83.8, 58.0, 52.0, 51.9, 49.4, 28.3, 20.9. **HRMS (ESI-TOF)** calcd for $C_{19}H_{24}N_2O_5$ ($M+H^+$): 361.1758; Found: 361.1762.

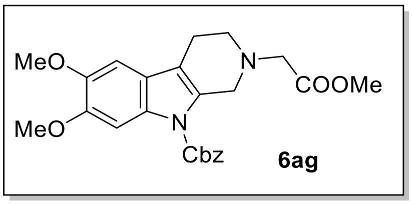

benzyl 6,7-dimethoxy-2-(2-methoxy-2-oxoethyl)-1,2,3,4-tetrahydro-9*H*-pyrido[3,4-*b*]indole-9-carboxylate (6ag): According to the general procedure for 8 h, light yellow solid, m.p. = 108 – 110 °C, 63 mg, 72% yield. **^{1}H NMR (400 MHz, $CDCl_3$)** δ 7.67 (s, 1H), 7.47 (m, 2H), 7.44 – 7.31 (m, 3H), 6.82 (s, 1H), 5.39 (s, 2H), 4.06 (s, 2H), 3.91 (s, 3H), 3.75 (m, 6H), 3.49 (s, 2H), 2.98 (t, J = 5.6 Hz, 2H), 2.74 (t, J = 5.6 Hz, 2H). **^{13}C NMR (101 MHz, $CDCl_3$)** δ 171.0, 151.4, 147.2, 146.5, 134.9, 130.8, 129.7, 128.8, 128.7, 122.1, 115.3, 99.9, 68.8, 57.9, 56.2, 56.0, 51.8, 51.8, 49.3, 21.1. **HRMS (ESI-TOF)** calcd for $C_{24}H_{26}N_2O_6$ ($M+H^+$): 439.1864; Found: 439.1862.

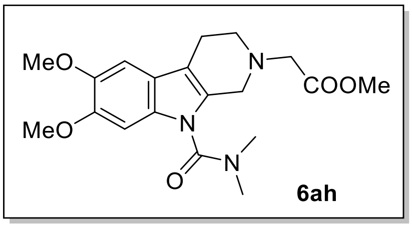

methyl 2-(9-(dimethylcarbamoyl)-6,7-dimethoxy-1,3,4,9-tetrahydro-2*H*-pyrido[3,4-*b*]indol-2-yl)acetate (6ah): According to the general procedure for 8 h, light yellow gel, 20 mg, 27% yield. **^{1}H NMR (400 MHz, $CDCl_3$)** δ 6.88 (s, 1H), 6.82 (s, 1H), 3.91 (m, 8H), 3.75 (s, 3H), 3.50 (s, 2H), 3.19 – 2.93 (m, 8H), 2.80 (d, J = 5.2 Hz, 2H). **^{13}C NMR (101 MHz, $CDCl_3$)** δ 171.0, 154.6, 147.0, 145.9, 131.0, 129.1, 121.1, 112.0, 100.5, 96.7, 58.3, 56.6, 56.3, 51.9, 50.4, 50.3, 38.4, 21.3. **HRMS (ESI-TOF)** calcd for $C_{19}H_{25}N_3O_5$ ($M+H^+$): 376.1867; Found: 376.1868.

5. Procedures of Synthetic Application

5.1 Procedure for the synthesis of 8

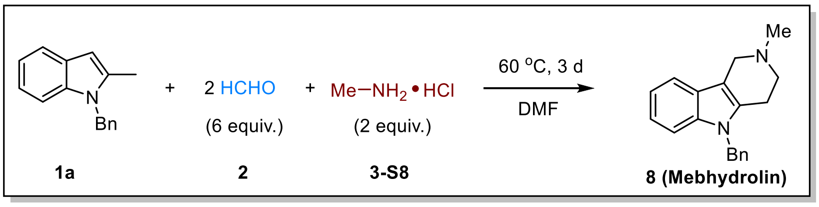

A mixture of **1a** (44 mg, 0.2 mmol), formaldehyde **2** (37% in water, 0.08 mL, 5 equiv.) and methylamine hydrochloride **3-S8** (26 mg, 0.4 mmol) in DMF (1 mL) was stirred at 60 °C until the reaction was completed. The reaction was quenched by saturated aqueous $NaHCO_3$. The aqueous layer was extracted with ethyl acetate (three times), and the combined organic layer was dried over Na_2SO_4 and concentrated. Purification by silica gel column chromatography to give Mebhydrolin **8** (38 mg, 69% yield).

5.2 Gram scale experiment for synthesis of 8

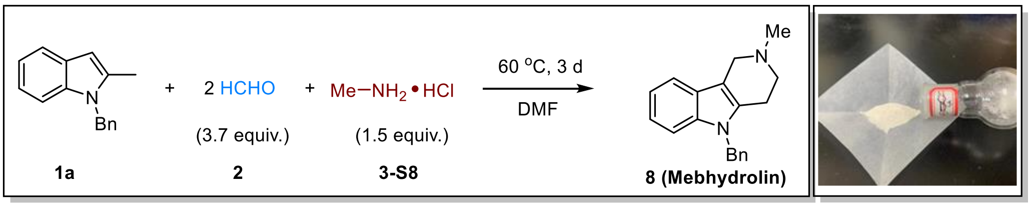

A mixture of **1a** (1.1 g, 5 mmol), formaldehyde **2** (37% in water, 1,5 mL, 3.7 equiv.) and methylamine hydrochloride **3-S8** (507 mg, 7.5 mmol) in DMF (20 mL) was stirred at 60 °C until the reaction was completed. The reaction was quenched by saturated aqueous $NaHCO_3$. The aqueous layer was extracted with ethyl acetate (three times), and the combined organic layer was dried over Na_2SO_4 and concentrated. Purification by silica gel column chromatography to give Mebhydrolin **8** (895 mg, 64.9% yield).

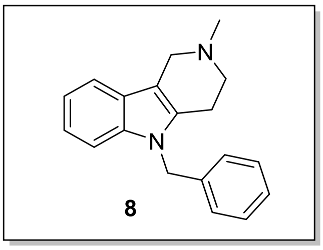

5-benzyl-2-methyl-2,3,4,5-tetrahydro-1*H*-pyrido[4,3-*b*]indole (8): off-white solid, m.p. = 86 – 88 °C, 38 mg, 69% yield. **^{1}H NMR (400 MHz, $CDCl_3$)** δ 7.54 – 7.46 (m, 1H), 7.34 – 7.22 (m, 4H), 7.20 – 7.09 (m, 2H), 7.05 (d, *J* = 7.2 Hz, 2H), 5.30 (s, 2H), 3.78 (s, 2H), 2.97 – 2.76 (m,

4H), 2.62 (s, 3H). **^{13}C NMR (101 MHz, $CDCl_3$)** δ 137.9, 136.9, 133.4, 128.8, 127.3, 126.2, 125.8, 121.0, 119.2, 117.7, 109.3, 108.4, 52.5, 51.9, 46.4, 45.8, 23.0. **HRMS (ESI-TOF)** calcd for $C_{19}H_{20}N_2$ (M+H$^+$): 277.1699; Found: 277.1696.

5.3 Procedure for the synthesis of 9

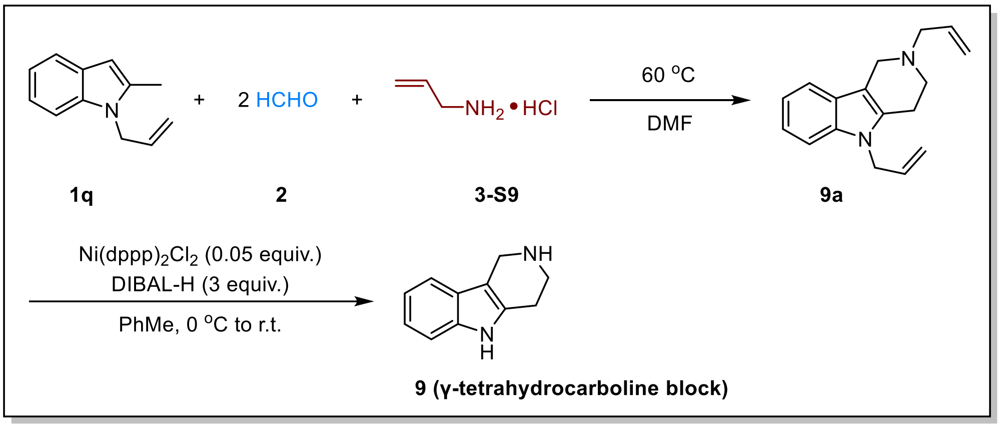

A mixture of **1q** (34 mg, 0.2 mmol), formaldehyde **2** (37% in water, 0.08 mL, 5 equiv.) and allylamine hydrochloride **3-S9** (37 mg, 0.4 mmol) in DMF (1 mL) was stirred at 60 °C until the reaction was completed. The reaction was quenched by saturated aqueous $NaHCO_3$. The aqueous layer was extracted with ethyl acetate (three times), and the combined organic layer was dried over Na_2SO_4 and concentrated. Purification by silica gel column chromatography to give the intermediate **9a** (35 mg, 70% yield).

A schlenk tube equipped with a magnetic stir bar was charged with **9a** (50 mg, 0.2 mmol), Ni(dppp)$_2$Cl$_2$ (5 mg, 5 mol%), and then purged with argon three times. Anhydrous toluene (2 mL) was added. The reaction was stirred at 0 °C for 15 minutes. Then DIBAL-H (0.6 mL, 0.6 mmol, 1M in hexanes) was added dropwise. The resulting mixture was stirred at 0 °C for 1 hour and then slowly warmed to room temperature. After completion, water was added to quench the reaction and the mixture was extracted with EtOAc. The organic layer was washed with brine, dried over anhydrous Na_2SO_4 and concentrated under vacuum to obtain the residue, which was purified by silica gel column chromatography to afford the product **9** (24 mg, 70% yield).

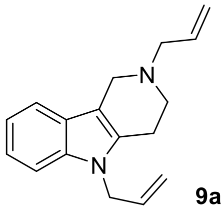

9a

2,5-diallyl-2,3,4,5-tetrahydro-1*H*-pyrido[4,3-*b*]indole (9a): light yellow gel, 35 mg, 70% yield. **^{1}H NMR (400 MHz, CDCl$_3$)** δ 7.42 (d, *J* = 7.6 Hz, 1H), 7.27 – 7.21 (m, 1H), 7.13 (t, *J* = 7.2 Hz, 1H), 7.06 (t, *J* = 7.2 Hz, 1H), 6.01 (m, 1H), 5.90 (m, 1H), 5.28 (d, *J* = 17.2 Hz, 1H), 5.21 (d, *J* = 10.4 Hz, 1H), 5.10 (d, *J* = 10.4 Hz, 1H), 4.89 (d, *J* = 17.2 Hz, 1H), 4.66 – 4.55 (m, 2H), 3.74 (s, 2H), 3.30 (d, *J* = 6.4 Hz, 2H), 2.90 (t, *J* = 5.6 Hz, 2H), 2.82 (t, *J* = 5.2 Hz, 2H). **^{13}C NMR (101 MHz, CDCl$_3$)** δ 136.5, 135.6, 133.5, 133.5, 125.8, 120.8, 119.0, 118.0, 117.7, 116.4, 109.1, 108.4, 61.1, 50.2, 49.8, 45.3, 22.7. **HRMS (ESI-TOF)** calcd for $C_{17}H_{20}N_2$ (M+H$^+$): 253.1699; Found: 253.1696.

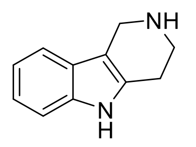

9

2,3,4,5-tetrahydro-1*H*-pyrido[4,3-*b*]indole (9): light yellow gel, m.p. = 78 – 80 °C, 24 mg, 70% yield. **^{1}H NMR (400 MHz, MeOD)** δ 7.35 (d, *J* = 7.6 Hz, 1H), 7.29 (d, *J* = 8.0 Hz, 1H), 7.11 – 7.03 (m, 1H), 7.02 – 6.91 (m, 1H), 4.88 (s, 2H), 4.13 (s, 2H), 3.35 – 3.27 (m, 3H), 2.93 (t, *J* = 6.0 Hz, 2H). **^{13}C NMR (101 MHz, MeOD)** δ 136.3, 131.1, 125.5, 120.9, 118.6, 116.8, 110.5, 104.7, 42.3, 41.2, 21.9. **HRMS (ESI-TOF)** calcd for $C_{11}H_{12}N_2$ (M+H$^+$): 173.1073; Found: 173.1070.

5.4 Procedure for the synthesis of 10

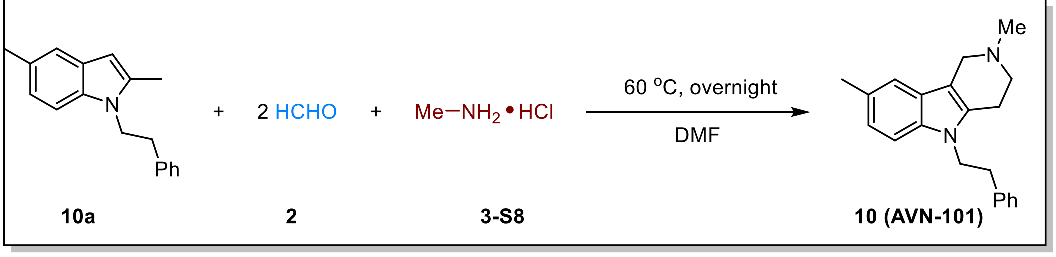

A mixture of **10a** (50 mg, 0.2 mmol), formaldehyde **2** (37% in water, 0.08 mL, 5 equiv.) and methylamine hydrochloride **3-S8** (26 mg, 0.4 mmol) in DMF (1 mL) was stirred at 60 °C until the reaction was completed. The reaction was quenched by saturated aqueous $NaHCO_3$. The aqueous layer was extracted with ethyl acetate (three times), and the combined organic layer was dried over Na_2SO_4 and concentrated. Purification by silica gel column chromatography to give the desired product **10** (53 mg, 87% yield).

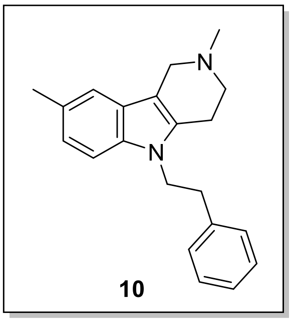

2,8-dimethyl-5-phenethyl-2,3,4,5-tetrahydro-1*H*-pyrido[4,3-*b*]indole (10): light yellow gel, 53 mg, 87% yield. **^{1}H NMR (400 MHz, CDCl$_3$)** δ 7.30 – 7.16 (m, 5H), 7.07 – 6.96 (m, 3H), 4.19 (t, *J* = 7.2 Hz, 2H), 3.70 (s, 2H), 2.98 (t, *J* = 7.2 Hz, 2H), 2.75 (t, *J* = 5.6 Hz, 2H), 2.54 (s, 3H), 2.49 (t, *J* = 5.6 Hz, 2H), 2.45 (s, 3H). **^{13}C NMR (101 MHz, CDCl$_3$)** δ 138.9, 134.5, 132.9, 128.9, 128.6, 128.3, 126.7, 126.0, 122.3, 117.6, 108.7, 106.4, 52.3, 51.7, 45.2, 45.1, 36.6, 22.2, 21.5. **HRMS (ESI-TOF)** calcd for $C_{21}H_{24}N_2$ (M+H$^+$): 305.2012; Found: 305.2011.

5.5 Procedure for the synthesis of 11

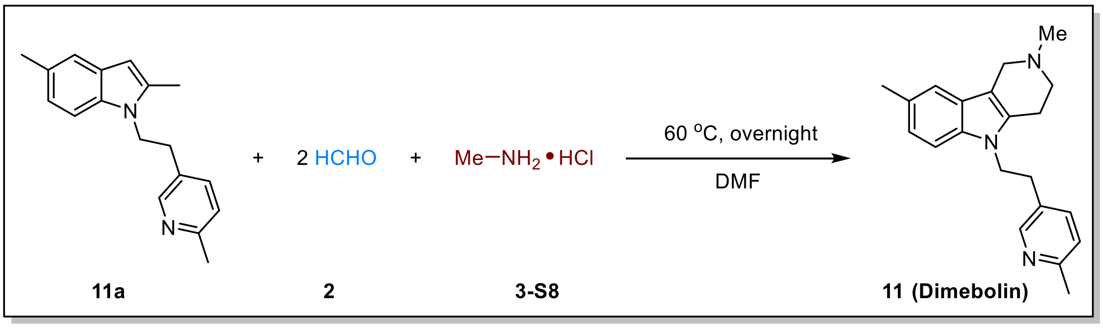

A mixture of **11a** (53 mg, 0.2 mmol), formaldehyde **2** (37% in water, 0.08 mL, 5 equiv.) and methylamine hydrochloride **3-S8** (26 mg, 0.4 mmol) in DMF (1 mL) was stirred at 60 °C until the reaction was completed. The reaction was quenched by saturated aqueous $NaHCO_3$. The aqueous layer was extracted with ethyl acetate (three times), and the combined organic layer was dried over Na_2SO_4 and concentrated. Purification by silica gel column chromatography to give the desired product **11** (51 mg, 80% yield).

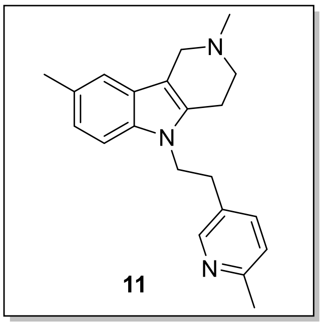

2,8-dimethyl-5-(2-(6-methylpyridin-3-yl)ethyl)-2,3,4,5-tetrahydro-1*H*-pyrido[4,3-*b*]indole (11): light yellow gel, 51 mg, 80% yield. **^{1}H NMR (400 MHz, $CDCl_3$)** δ 8.19 (d, J = 1.2 Hz, 1H), 7.20 (s, 1H), 7.15 (d, J = 8.4 Hz, 1H), 7.06 (dd, J_1 = 8.0 Hz, J_2 = 2.0 Hz, 1H), 6.99 (d, J = 8.0 Hz, 2H), 4.18 (t, J = 7.2 Hz, 2H), 3.75 (s, 2H), 2.96 (t, J = 7.2 Hz, 2H), 2.80 (t, J = 5.6 Hz, 2H), 2.56 (s, 3H), 2.54 – 2.48 (m, 5H), 2.44 (s, 3H). **^{13}C NMR (101 MHz, $CDCl_3$)** δ 156.8, 149.3, 136.9, 134.4, 132.4, 131.0, 128.5, 126.0, 123.0, 122.6, 117.7, 108.7, 106.3, 52.1, 51.6, 44.9, 44.7, 33.2, 24.1, 22.1, 21.5. **HRMS (ESI-TOF)** calcd for $C_{21}H_{25}N_3$ ($M+H^+$): 320.2121; Found: 320.2119.

5.6 Procedure for the synthesis of 12 and 13

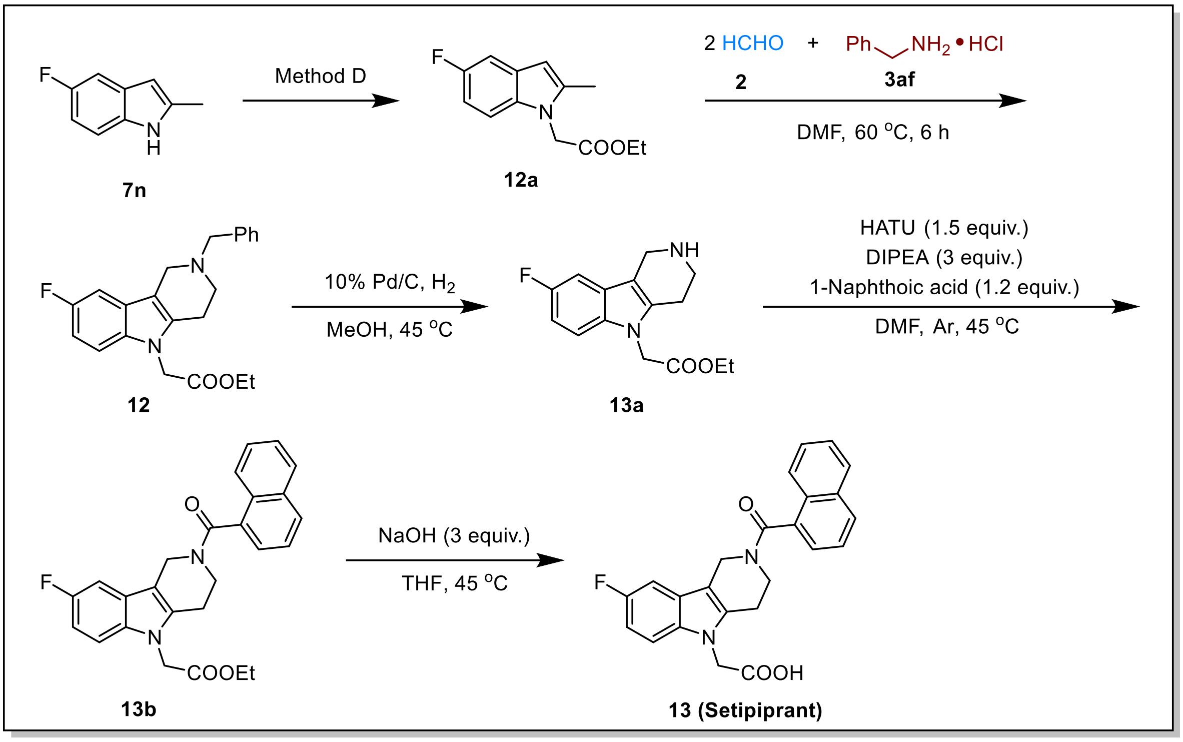

A mixture of **12a** (47 mg, 0.2 mmol), formaldehyde **2** (37% in water, 0.08 mL, 5 equiv.) and benzylamine hydrochloride **3af** (57 mg, 0.4 mmol) in DMF (1 mL) was stirred at 60 °C until the reaction was completed. The reaction was quenched by saturated aqueous $NaHCO_3$. The aqueous layer was extracted with ethyl acetate (three times), and the combined organic layer was dried over Na_2SO_4 and concentrated. Purification by silica gel column chromatography to give the desired product **12** (69 mg, 86% yield).

To a solution of **12** (73 mg, 0.2 mmol) in MeOH (5 mL) was added 10% Pd/C (10% wt.). Then the mixture was purged with hydrogen three times. The resulting reaction mixture was kept at 45 °C for 6 hours. After completion, the resulting mixture was concentrated in vacuo; and

purified by silica gel column chromatography to afford the starting indoles **13a** (76 mg, 91% yield).

A schlenk tube equipped with a magnetic stir bar was charged with **13a** (55 mg, 0.2 mmol), HATU (114 mg, 0.3 mmol), 1-naphthoic acid (41 mg, 0.24 mmol) and then purged with argon three times. Anhydrous DMF (2 mL) was added. The reaction was stirred at 0 ^{o}C for 15 minutes. Then DIPEA (78 mg, 0.6 mmol) was added dropwise. The resulting mixture was stirred at 0 ^{o}C for 1 hour and then slowly warmed to room temperature. After completion, water was added to quench the reaction and the mixture was extracted with EtOAc. The organic layer was washed with brine, dried over anhydrous Na_2SO_4 and concentrated under vacuum to obtain the residue, which was purified by silica gel column chromatography to afford the intermediate **13b**.

A flask equipped with a magnetic stir bar was charged with **13b** (86 mg, 0.2 mmol). THF (5 mL) was added as solvent. NaOH (24 mg, 0.6 mmol) was dissolved in water (1 mL) and added dropwise. The reaction was stirred at 45 ^{o}C for 2 hours. 4N HCl was added to quench the reaction and the mixture was extracted with EtOAc. The organic layer was washed with brine, dried over anhydrous Na_2SO_4 and concentrated under vacuum to obtain the residue, which was purified by silica gel column chromatography to afford the Setipiprant **13** (55 mg, 69% yield for 2 steps).

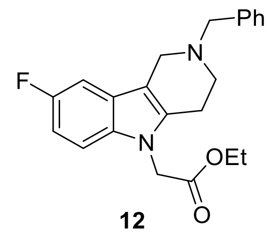

12

ethyl 2-(2-benzyl-8-fluoro-1,2,3,4-tetrahydro-5*H*-pyrido[4,3-*b*]indol-5-yl)acetate (12): light yellow gel, 63 mg, 86% yield. **^{1}H NMR (400 MHz, CDCl$_3$)** δ 7.42 (d, *J* = 7.2 Hz, 2H), 7.35 (t, *J* = 7.2 Hz, 2H), 7.30 (d, *J* = 7.2 Hz, 1H), 7.08 (dd, J_1 = 8.8 Hz, J_2 = 4.0 Hz, 1H), 7.02 (dd, J_1 = 9.6 Hz, J_2 = 2.0 Hz, 1H), 6.88 (m, 1H), 4.69 (s, 2H), 4.20 (m, 2H), 3.80 (s, 2H), 3.69 (s, 2H), 2.92 (t, *J* = 5.6 Hz, 2H), 2.77 (t, *J* = 5.2 Hz, 2H), 1.26 (t, *J* = 7.2 Hz, 3H). **^{13}C NMR (101 MHz, CDCl$_3$)** δ 168.6, 159.1, 156.8, 138.5, 135.6, 133.5, 129.1, 128.4, 127.2, 126.5, 126.4, 109.2, 109.1, 109.0, 103.3, 103.1, 62.2, 61.7, 49.9, 49.6, 44.9, 22.7, 14.2. **^{19}F NMR (565 MHz, CDCl$_3$)** δ -124.85. **HRMS (ESI-TOF)** calcd for $C_{22}H_{23}N_2O_2F$ (M+H$^+$): 367.1816; Found: 367.1816.

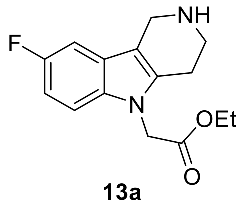

13a

ethyl 2-(8-fluoro-1,2,3,4-tetrahydro-5*H*-pyrido[4,3-*b*]indol-5-yl)acetate (13a): off-white solid, m.p. = 122 – 124 °C, 76 mg, 91% yield. **^{1}H NMR (400 MHz, MeOD)** δ 7.21 (dd, J_1 = 8.8 Hz, J_2 = 4.0 Hz, 1H), 7.05 (dd, J_1 = 9.6 Hz, J_2 = 2.4 Hz, 1H), 6.86 (td, J_1 = 9.2 Hz, J_2 = 2.4 Hz, 1H), 4.90 – 4.87 (m, 2H), 4.17 (q, J = 7.2 Hz, 2H), 3.98 (s, 2H), 3.21 (t, J = 5.6 Hz, 2H), 2.76 (t, J = 5.6 Hz, 2H), 1.24 (t, J = 7.2 Hz, 3H). **^{13}C NMR (101 MHz, MeOD)** δ 169.2, 159.1, 156.8, 135.5, 133.4, 126.1, 126.0, 109.3, 109.2, 108.7, 108.4, 107.7, 102.3, 102.1, 61.3, 43.8, 42.3, 41.0, 21.6, 13.1. **^{19}F NMR (565 MHz, MeOD)** δ -127.12. **HRMS (ESI-TOF)** calcd for $C_{15}H_{17}N_2O_2F$ (M+H$^+$): 277.1347; Found: 277.1347.

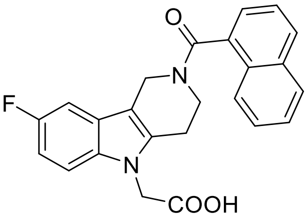

13

2-(2-(1-naphthoyl)-8-fluoro-1,2,3,4-tetrahydro-5*H*-pyrido[4,3-*b*]indol-5-yl)acetic acid (13): light yellow solid, m.p. = 210 – 212 °C, 55 mg, 69% yield (for 2 steps). Mixture of two rotamers: **^{1}H NMR (400 MHz, CDCl$_3$)** δ 9.41 (s, 1H), 8.03 – 7.61 (m, 3H), 7.40 (m, 4H), 7.19 – 6.56 (m, 3H), 5.14 – 4.93 (m, 1H), 4.52 (m, 2H), 4.39 – 4.00 (m, 2H), 3.45 (s, 1H), 2.98 – 2.17 (m, 2H). **^{13}C NMR (101 MHz, CDCl$_3$)** δ 171.1, 159.2, 159.0, 156.9, 156.7, 135.4, 134.2, 133.5, 133.4, 133.3, 133.2, 129.8, 129.6, 129.5, 129.3, 128.5, 127.3, 126.7, 126.6, 125.8, 125.7, 125.2, 125.2, 125.1, 124.6, 124.5, 124.2, 123.9, 110.1, 109.9, 109.9, 109.7, 109.5, 109.4, 109.4, 109.3, 107.1, 106.8, 103.6, 103.3, 103.1, 102.9, 44.9, 44.6, 44.4, 44.3, 40.0, 39.8, 22.9, 22.2. **^{19}F NMR (565 MHz, CDCl$_3$)** δ -123.76, -124.12. **HRMS (ESI-TOF)** calcd for $C_{24}H_{19}N_2O_3F$ (M+H$^+$): 403.1452; Found: 403.1451.

5.7 Procedure for the synthesis of 14

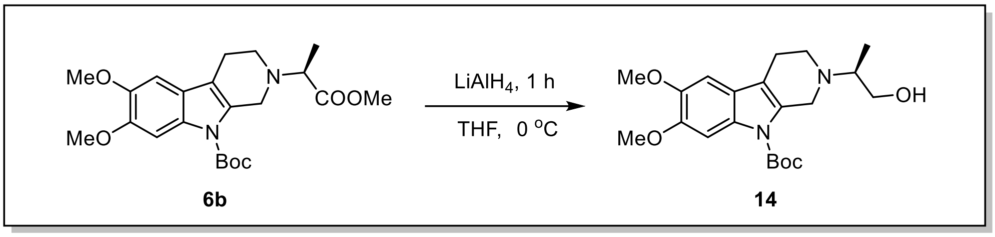

A flask equipped with a magnetic stir bar was charged with **6b** (84 mg, 0.2 mmol). THF (5 mL) was added as solvent. $LiAlH_4$ (23 mg, 0.6 mmol) was added slowly, and the reaction was stirred at 0 °C for 1 hour. Water was added to quench the reaction and the mixture was extracted with EtOAc. The organic layer was washed with brine, dried over anhydrous Na_2SO_4 and concentrated under vacuum to obtain the residue, which was purified by silica gel column chromatography to afford the product **14** (69 mg, 89% yield).

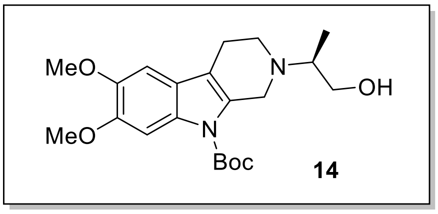

***tert*-butyl (*S*)-2-(1-hydroxypropan-2-yl)-6,7-dimethoxy-1,2,3,4-tetrahydro-9*H*-pyrido[3,4-*b*]indole-9-carboxylate (14)**: light yellow gel, 69 mg, 89% yield. **^{1}H NMR (400 MHz, $CDCl_3$)** δ 7.76 (s, 1H), 6.84 (s, 1H), 4.12 – 3.83 (m, 8H), 3.58 – 3.39 (m, 2H), 3.18 – 2.96 (m, 2H), 2.76 – 2.61 (m, 3H), 1.67 (s, 9H), 1.05 (d, J = 6.4 Hz, 3H). **^{13}C NMR (101 MHz, $CDCl_3$)** δ 150.3, 147.0, 146.3, 131.8, 129.7, 121.9, 115.0, 99.9, 99.7, 83.5, 62.7, 60.0, 56.2, 56.2, 48.3, 44.3, 28.3, 22.4, 9.8. **HRMS (ESI-TOF)** calcd for $C_{21}H_{30}N_2O_5$ ($M+H^+$): 391.2227; Found: 391.2223.

5.8 Procedure for the synthesis of 15

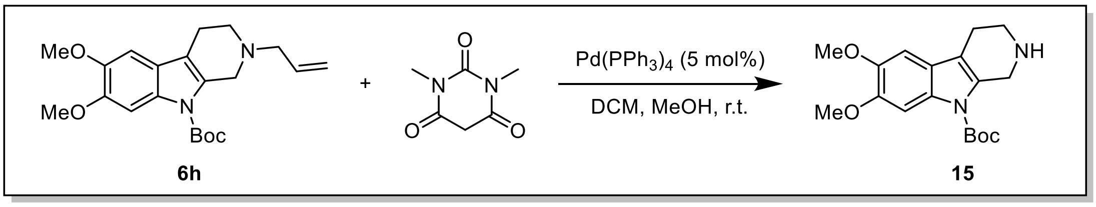

A flask equipped with a magnetic stir bar was charged with **6h** (84 mg, 0.2 mmol). 1,3-dimethylbarbituric acid (47 mg, 0.3 mmol) and $Pd(PPh_3)_4$ (11 mg, 5 mol%). DCM (4 mL) and MeOH (1 mL) was added as solvent, and the reaction was stirred at room temperature for 2 hours. Saturated aqueous $NaHCO_3$ was added to quench the reaction and the mixture was extracted with

DCM. The organic layer was washed with brine, dried over anhydrous Na_2SO_4 and concentrated under vacuum to obtain the residue, which was purified by silica gel column chromatography to afford the product **15** (60 mg, 90% yield).

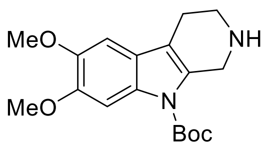

15

***tert*-butyl 6,7-dimethoxy-1,2,3,4-tetrahydro-9*H*-pyrido[3,4-*b*]indole-9-carboxylate (15):** light yellow gel, 60 mg, 90% yield. **^{1}H NMR (400 MHz, $CDCl_3$)** δ 7.79 (s, 1H), 6.83 (s, 1H), 4.30 (s, 2H), 3.94 (d, *J* = 6.4 Hz, 6H), 3.23 (t, *J* = 5.6 Hz, 2H), 2.75 (t, *J* = 5.2 Hz, 2H), 1.65 (s, 9H). **^{13}C NMR (101 MHz, $CDCl_3$)** δ 150.2, 147.3, 146.3, 134.0, 130.6, 129.6, 121.9, 114.7, 99.7, 83.8, 56.2, 56.2, 44.9, 42.4, 28.3, 21.8. **HRMS (ESI-TOF)** calcd for $C_{18}H_{24}N_2O_4$ (M+H$^+$): 333.1809; Found: 333.1810.

5.9 Procedure for the synthesis of 16

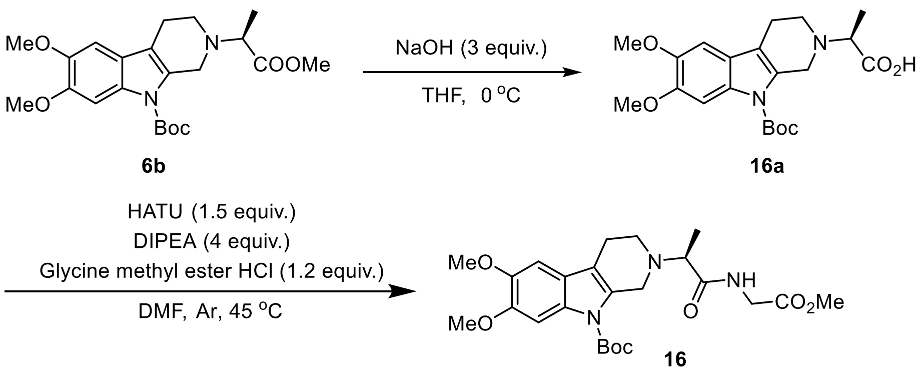

A flask equipped with a magnetic stir bar was charged with **6b** (84 mg, 0.2 mmol). THF (5 mL) was added as solvent. NaOH (24 mg, 0.6 mmol) was dissolved in water (1 mL) and added dropwise. The reaction was stirred at 45 °C for 2 hours. 4N HCl was added to water layer pH to 6 and the mixture was extracted with DCM. The organic layer was washed with brine, dried over anhydrous Na_2SO_4 and concentrated under vacuum to obtain the intermediate acid **16a,** which was used in next step without purification**.**

A schlenk tube equipped with a magnetic stir bar was charged with **16a**, HATU (114 mg, 0.3 mmol), glycine methyl ester hydrochloride (30 mg, 0.24 mmol) and then purged with argon

three times. Anhydrous DMF (2 mL) was added. The reaction was stirred at 0 °C for 15 minutes. Then DIPEA (104 mg, 0.8 mmol) was added dropwise. The resulting mixture was stirred at 45°C for 3 hours. After completion, water was added to quench the reaction and the mixture was extracted with EtOAc. The organic layer was washed with brine, dried over anhydrous Na_2SO_4 and concentrated under vacuum to obtain the residue, which was purified by silica gel column chromatography to afford the intermediate **16** (62 mg, 65% yield for 2 steps).

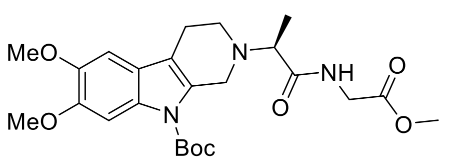

16

***tert*-butyl (*S*)-6,7-dimethoxy-2-(1-((2-methoxy-2-oxoethyl)amino)-1-oxopropan-2-yl)-1,2,3,4-tetrahydro-9*H*-pyrido[3,4-*b*]indole-9-carboxylate (16):** light yellow gel, 62 mg, 65% yield (for 2 steps). **^{1}H NMR (400 MHz, $CDCl_3$)** δ 7.99 – 7.58 (m, 2H), 6.84 (s, 1H), 4.19 – 3.99 (m, 3H), 3.99 – 3.87 (m, 7H), 3.73 (s, 3H), 3.43 (q, *J* = 6.8 Hz, 1H), 2.82 (d, *J* = 4.0 Hz, 2H), 2.78 – 2.71 (m, 2H), 1.65 (s, 9H), 1.37 (d, *J* = 6.8 Hz, 3H). **^{13}C NMR (101 MHz, $CDCl_3$)** δ 174.4, 170.6, 150.3, 147.1, 146.3, 131.3, 129.8, 121.8, 114.9, 99.8, 99.8, 83.6, 63.3, 56.2, 56.1, 52.3, 49.8, 46.8, 40.7, 28.3, 22.2, 11.5. **HRMS (ESI-TOF)** calcd for $C_{24}H_{33}N_3O_7$ (M+H$^+$): 476.2391; Found: 476.2385.

5.10 Procedure for the synthesis of 17

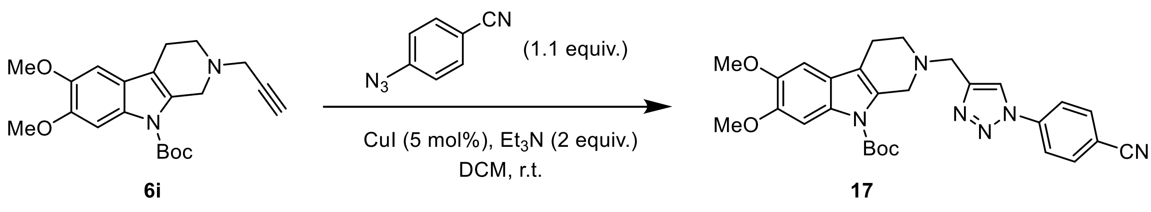

A flask equipped with a magnetic stir bar was charged with **6i** (74 mg, 0.2 mmol), 4-azidobenzonitrile (32 mg, 0.22 mmol), triethylamine (40 mg, 0.4 mmol) and CuI (2 mg, 5 mol%). DCM (2 mL) was added as solvent, and the reaction was stirred at room temperature for 2 hours. The mixture was concentrated under vacuum to obtain the residue, which was purified by silica gel column chromatography to afford the product **17** (93 mg, 90% yield).

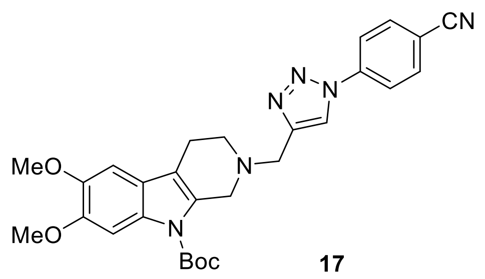

17

***tert*-butyl 2-((1-(4-cyanophenyl)-1*H*-1,2,3-triazol-4-yl)methyl)-6,7-dimethoxy-1,2,3,4-tetrahydro-9*H*-pyrido[3,4-*b*]indole-9-carboxylate (17):** light yellow gel, 93 mg, 90% yield. **^{1}H NMR (400 MHz, $CDCl_3$)** δ 8.11 (s, 1H), 7.90 (d, *J* = 7.2 Hz, 2H), 7.81 (d, *J* = 7.2 Hz, 2H), 7.74 (s, 1H), 6.82 (s, 1H), 4.04 (s, 4H), 3.91 (s, 6H), 2.96 (s, 2H), 2.74 (s, 2H), 1.61 (s, 9H). **^{13}C NMR (101 MHz, $CDCl_3$)** δ 150.3, 147.1, 146.8, 146.3, 139.9, 134.0, 130.8, 129.8, 128.9, 121.8, 120.5, 117.7, 114.6, 112.4, 99.8, 83.6, 56.2, 56.1, 52.8, 52.1, 49.6, 28.3, 21.2. **HRMS (ESI-TOF)** calcd for $C_{28}H_{30}N_6O_4$ $(M+H^+)$: 515.2401; Found: 515.2397.

6. Mechanistic Investigation

6.1 Procedure for the synthesis of 18 and 19[13]

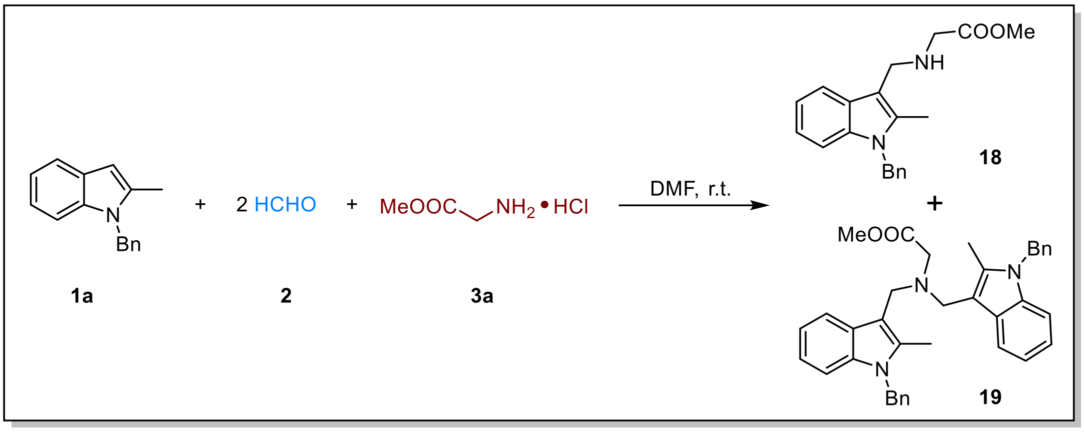

A mixture of **1a** (44 mg, 0.2 mmol), formaldehyde **2** (37% in water, 0.08 mL, 5 equiv.) and **3a** (50 mg, 0.4 mmol) in DMF (1 mL) was stirred at room temperature for 15 minutes. The reaction was quenched by saturated aqueous $NaHCO_3$. The aqueous layer was extracted with ethyl acetate (three times), and the combined organic layer was dried over Na_2SO_4 and concentrated. Purification by silica gel column chromatography to give the intermediates **18** and **19**.

6.2 Procedure for the synthesis of 20

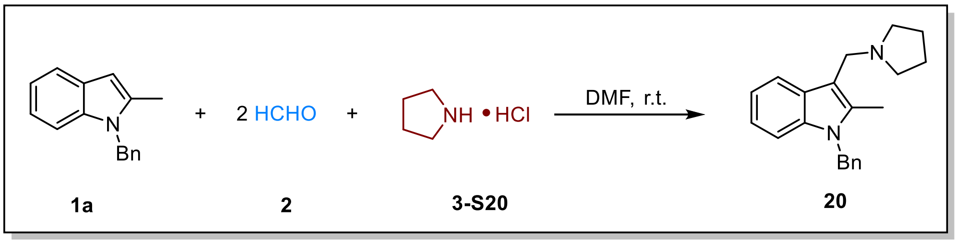

A mixture of **1a** (44 mg, 0.2 mmol), formaldehyde **2** (37% in water, 0.08 mL, 5 equiv.) and methylamine hydrochloride **3-S20** (43 mg, 0.4 mmol) in DMF (1 mL) was stirred at room temperature for 12 hours. The reaction was quenched by saturated aqueous $NaHCO_3$. The aqueous layer was extracted with ethyl acetate (three times), and the combined organic layer was dried over Na_2SO_4 and concentrated. Purification by silica gel column chromatography to give the intermediate **20**.

6.3 Deuterium-labelling and stepwise control reaction of γ-tetrahydrocarboline

To gain some insights into the reaction mechanism, several deuterium labeling experiments were conducted. We used **1a**, intermediate **18**, intermediate **19** as the starting materials respectively.

CD_2O (20% in D_2O), **3a** and DMF were added and the mixture was stirred at 60 °C until the reaction was completed. The deuterium-labelling results of the product compounds were determined by ^1H NMR spectrums.

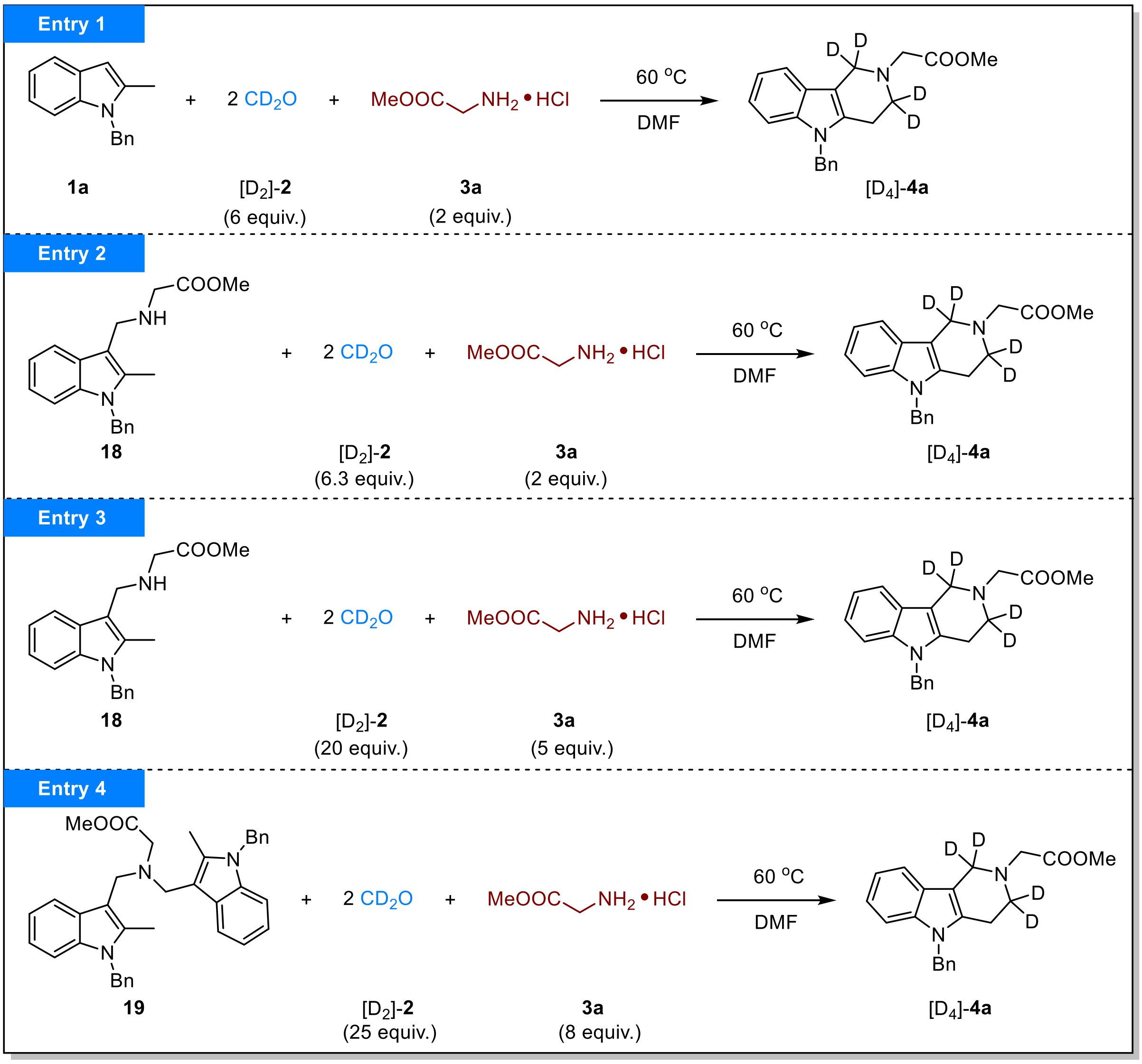

Entry 1: A mixture of **1a** (44 mg, 0.2 mmol), CD_2O **2** (20% in D_2O, 0.2 mL, 6 equiv.) and **3a** (50 mg, 0.4 mmol) in DMF (1 mL) was stirred at 60 °C until the reaction was completed. The reaction was quenched by saturated aqueous $NaHCO_3$. The aqueous layer was extracted with ethyl acetate (three times), and the combined organic layer was dried over Na_2SO_4 and concentrated. Purification by silica gel column chromatography to give the product [D_4]-**4a**.

Entry 2: A mixture of **18** (32 mg, 0.1 mmol), CD_2O **2** (20% in D_2O, 0.1 mL, 6.3 equiv.) and **3a** (25 mg, 0.2 mmol) in DMF (1 mL) was stirred at 60 °C until the reaction was completed. The reaction was quenched by saturated aqueous $NaHCO_3$. The aqueous layer was extracted with

ethyl acetate (three times), and the combined organic layer was dried over Na_2SO_4 and concentrated. Purification by silica gel column chromatography to give the product [D_4]-**4a**.

Entry 3: A mixture of **18** (32 mg, 0.1 mmol), CD_2O **2** (20% in D_2O, 0.3 mL, 20 equiv.) and **3a** (62.5 mg, 0.5 mmol) in DMF (1 mL) was stirred at 60 °C until the reaction was completed. The reaction was quenched by saturated aqueous $NaHCO_3$. The aqueous layer was extracted with ethyl acetate (three times), and the combined organic layer was dried over Na_2SO_4 and concentrated. Purification by silica gel column chromatography to give the product [D_4]-**4a**.

Entry 4: A mixture of **18** (28 mg, 0.05 mmol), CD_2O **2** (20% in D_2O, 0.2 mL, 25 equiv.) and **3a** (60 mg, 0.4 mmol) in DMF (1 mL) was stirred at 60 °C until the reaction was completed. The reaction was quenched by saturated aqueous $NaHCO_3$. The aqueous layer was extracted with ethyl acetate (three times), and the combined organic layer was dried over Na_2SO_4 and concentrated. Purification by silica gel column chromatography to give the product [D_4]-**4a**.

For deuterium-labelling and stepwise control reaction, the product **4a** could be obtained by all entries. As expected, the position-10 is almost deuterated methylene (Supplementary Figure 2). However, in the **Entry 2-4**, the methylene at position-11 was almost deuterized, which is hydrogenic in the starting materials **18** and **19**. And deuterization rate at both positions were improved through increasing the equivalent of CD_2O and **3a**.

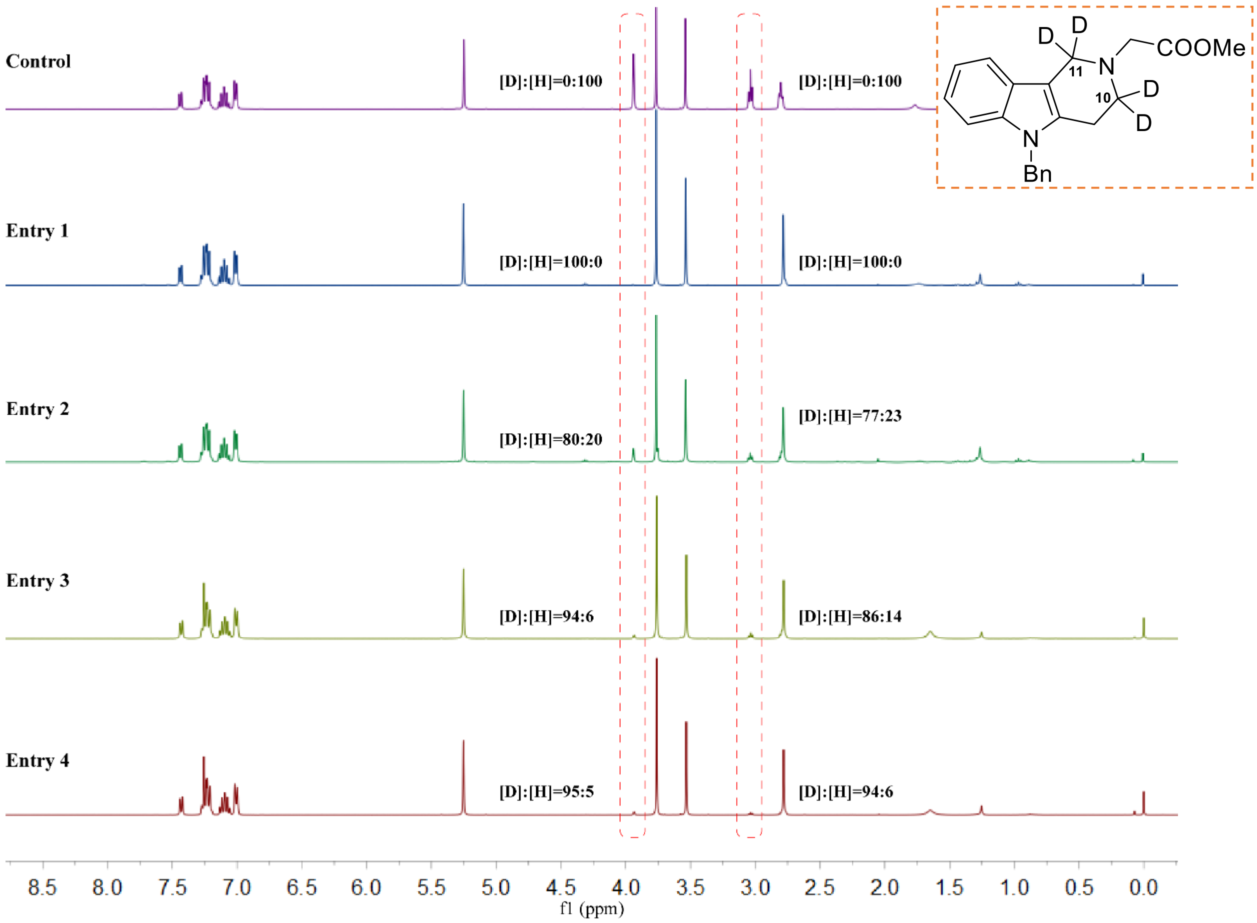

Supplementary Figure 2. ^{1}H NMR comparison of deuterium-labelling experiments for [D_4]-**4a**.

Based on these observations, a plausible reaction mechanism is proposed (Supplementary Figure 3). Intermediate **18** was formed via the Mannich alkylamination. At this stage, there are two reaction pathways to the final compound. **Path a** involves dearomatized alkenyl intermediate **Int-a2**. **Int-a2** undergoes the retro-Mannich reaction and cyclization to furnish the **4a**. However, due to the cyclization competition of alkenyl groups, there should have been nearly half of products are hydrogen at position-11, which was inconsistent with the result. In contrast, as described in **path b**, after forming **19** via the secondary Mannich reaction**,** a further alkylamination induced the inactivated carbon center to undergo dehydrogenation along with the formation of **Int-5**. The subsequent iminium formation of **Int-6** engages in cyclization, and retro-Mannich reaction to deliver the desired product [D_4]-**4a** with high deuterium rate.

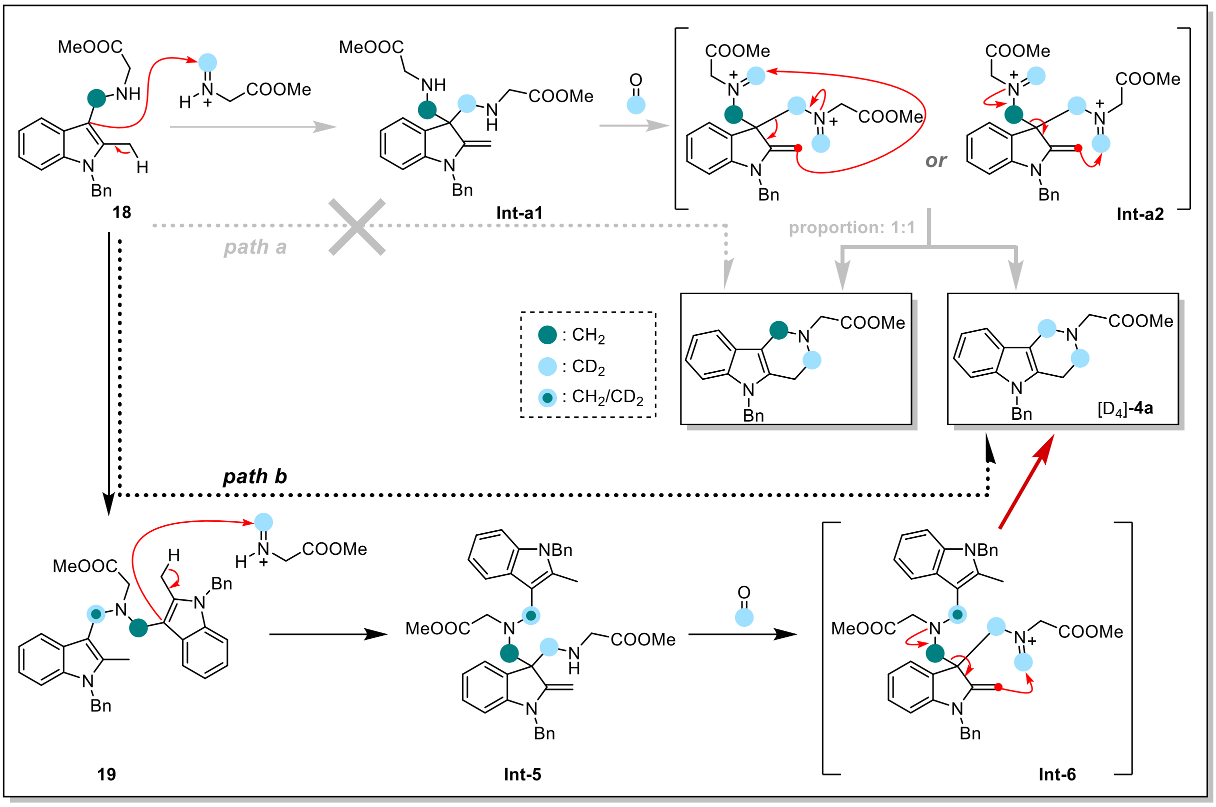

Supplementary Figure 3. Proposed reaction pathways.

To gain a deeper understanding of the reaction mechanism, a series of control experiments were carried out to detect reaction process.

A mixture of intermediate **18** or **19** (0.1 mmol), formaldehyde **2** (37% in water, 0.08 mL, 5 equiv.) in DMF (1.5 mL) was stirred at 60 ^{o}C for 3 hours. The reaction was detected every 20 minutes. TLC analysis showed that **4a** was not observed in both reactions. Interestingly, when we added 1drop aqueous HCl solution (1 N) as an additive to the reaction solution of **18** and formaldehyde, TLC analyses showed that **19** and **4a** were formed.

At this stage, we hypothesized that an alkylamination would occur from two molecules of **18** and formaldehyde to generate the intermediate **19** and release an imino group. Subsequently, cyclization, and retro-Mannich reaction would achieve the construction of **4a**.

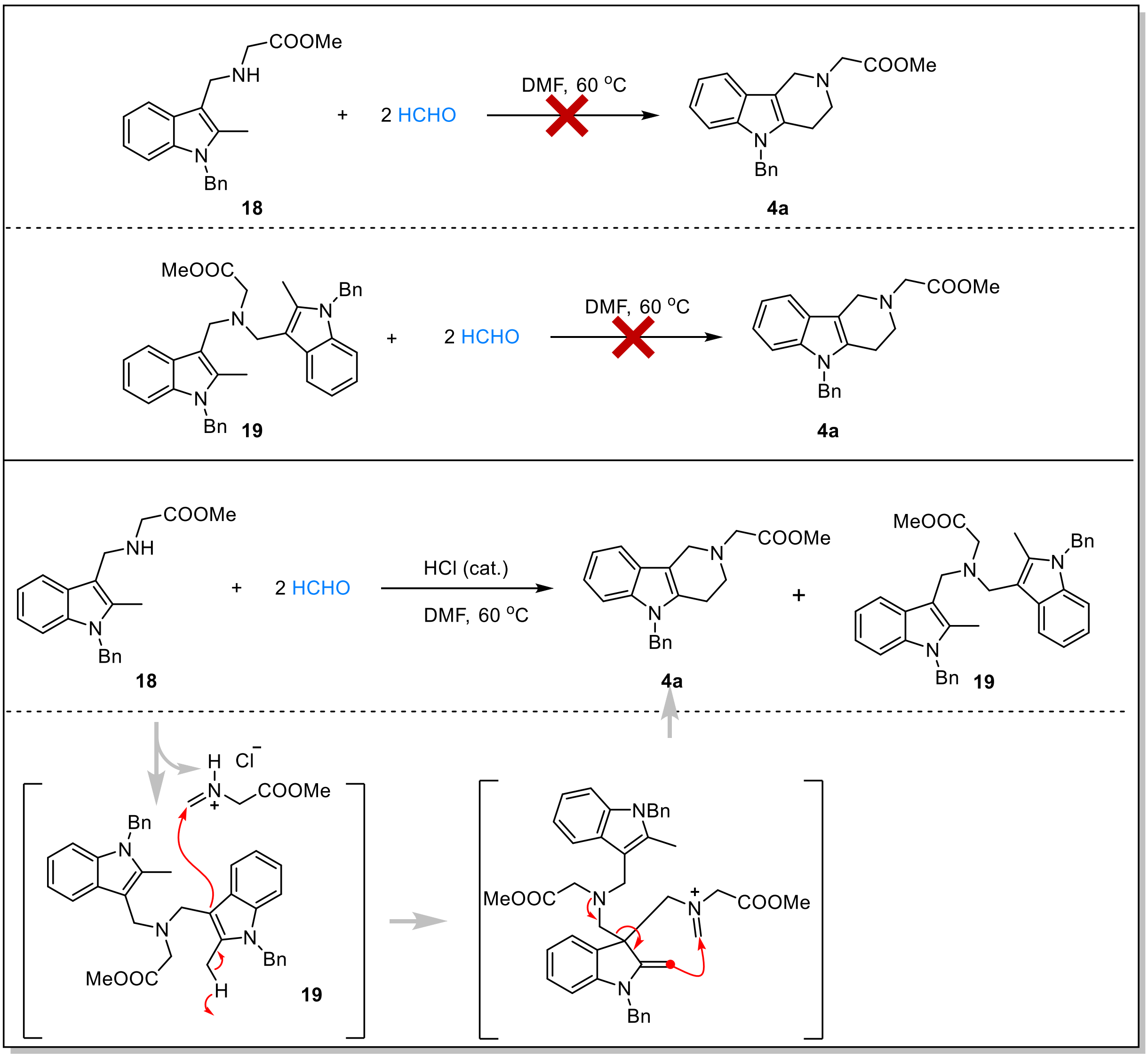

6.4 Cross-over reaction of γ-tetrahydrocarboline

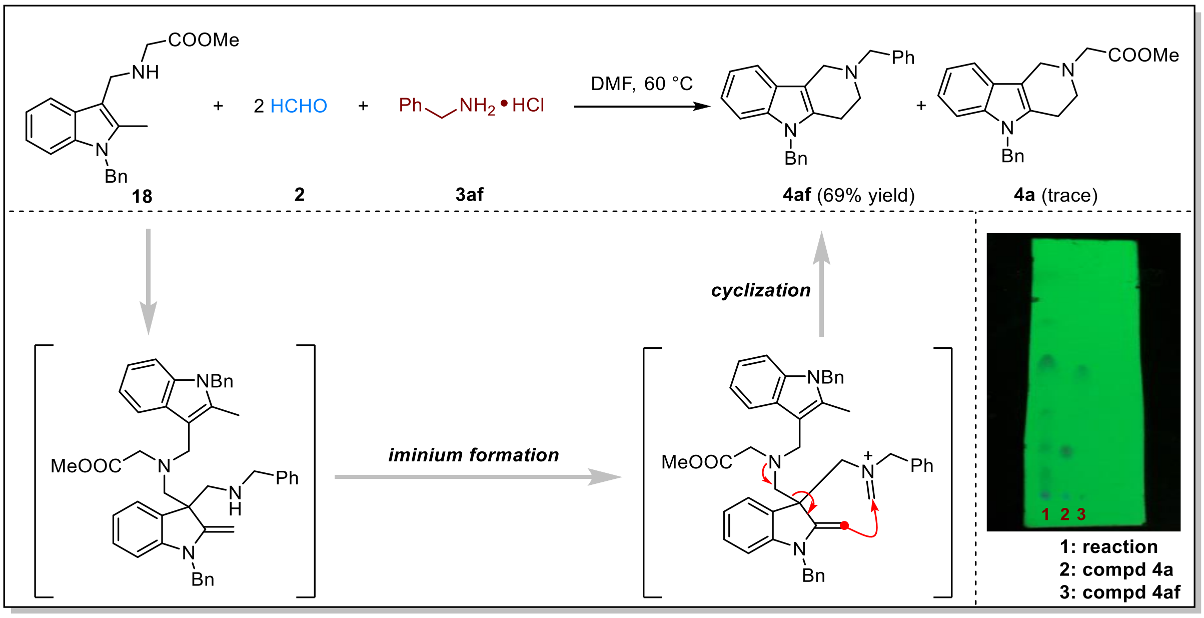

A mixture of **18** (0.1 mmol), formaldehyde **2** (37% in water, 0.04 mL, 5 equiv.) and benzylamine hydrochloride **3af** (0.2 mmol, 2 equiv.) in DMF (1.5 mL) was stirred at 60 °C for 3 hours. The reaction was quenched by saturated aqueous $NaHCO_3$. The aqueous layer was extracted with ethyl acetate (three times), and the combined organic layer was dried over Na_2SO_4 and concentrated. Purification by silica gel column chromatography to give corresponding indole alkaloids products **4af** (24 mg, 69%) and **4a** (trace).

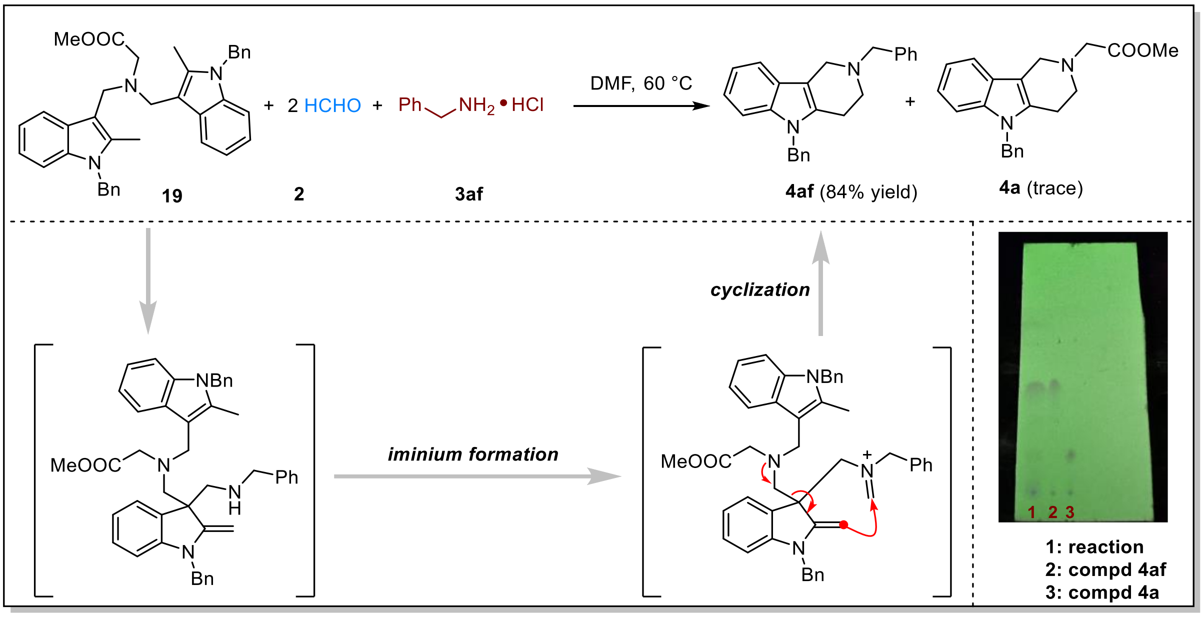

A mixture of **19** (0.1 mmol), formaldehyde **2** (37% in water, 0.08 mL, 10 equiv.) and benzylamine hydrochloride **3af** (0.4 mmol, 4 equiv.) in DMF (1.5 mL) was stirred at 60 °C for 3 hours. The reaction was quenched by saturated aqueous $NaHCO_3$. The aqueous layer was extracted with ethyl acetate (three times), and the combined organic layer was dried over Na_2SO_4 and concentrated. Purification by silica gel column chromatography to give corresponding indole alkaloids products **4af** (59 mg, 84%).

We subjected the intermediates **18** and **19** to the cross-over reaction conditions respectively. As shown on the TLC, formation of **4a** and **4af** were observed. And the huge proportional gap illustrated the rationality of the mechanism (Supplementary Figure 4): **18** and **19** were involved as intermediates for the formation of the desired product.

Accordingly, **19-f** was also subjected to the reaction with glycine methyl ester hydrochloride **3a**. and the result showed that the product **4a** occupied the vast majority.

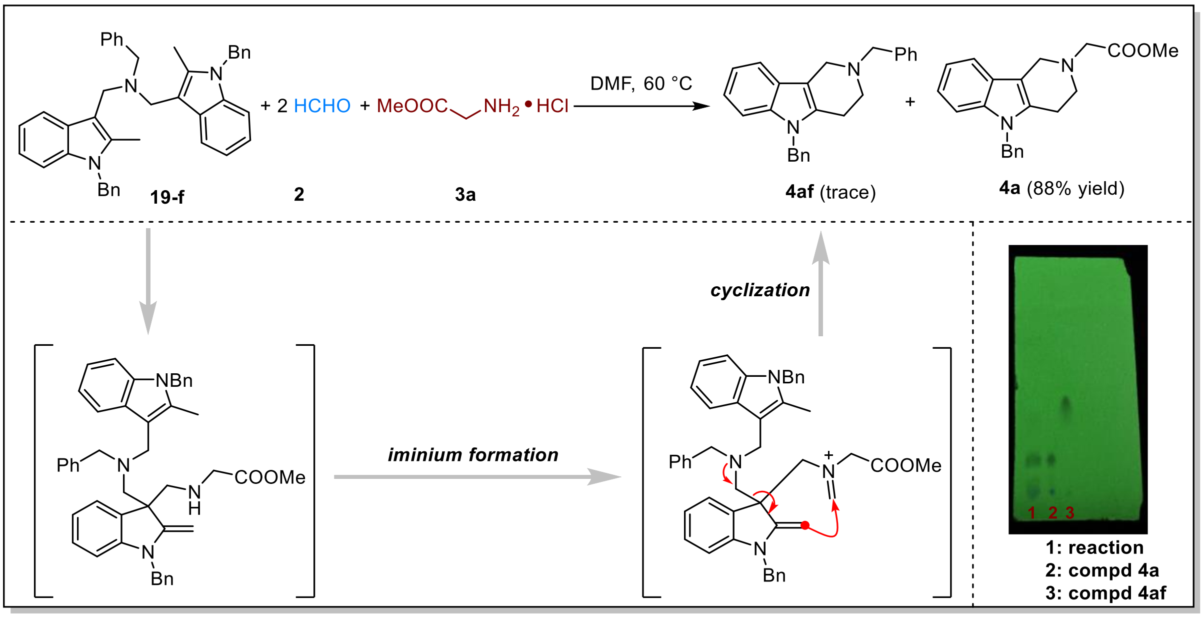

A mixture of **19-f** (0.1 mmol), formaldehyde **2** (37% in water, 0.08 mL, 10 equiv.) and **3a** (0.4 mmol, 4 equiv.) in DMF (1.5 mL) was stirred at 60 °C for 3 hours. The reaction was quenched by saturated aqueous $NaHCO_3$. The aqueous layer was extracted with ethyl acetate (three times), and the combined organic layer was dried over Na_2SO_4 and concentrated. Purification by silica gel column chromatography to give corresponding indole alkaloids products **4a** (59 mg, 88%).

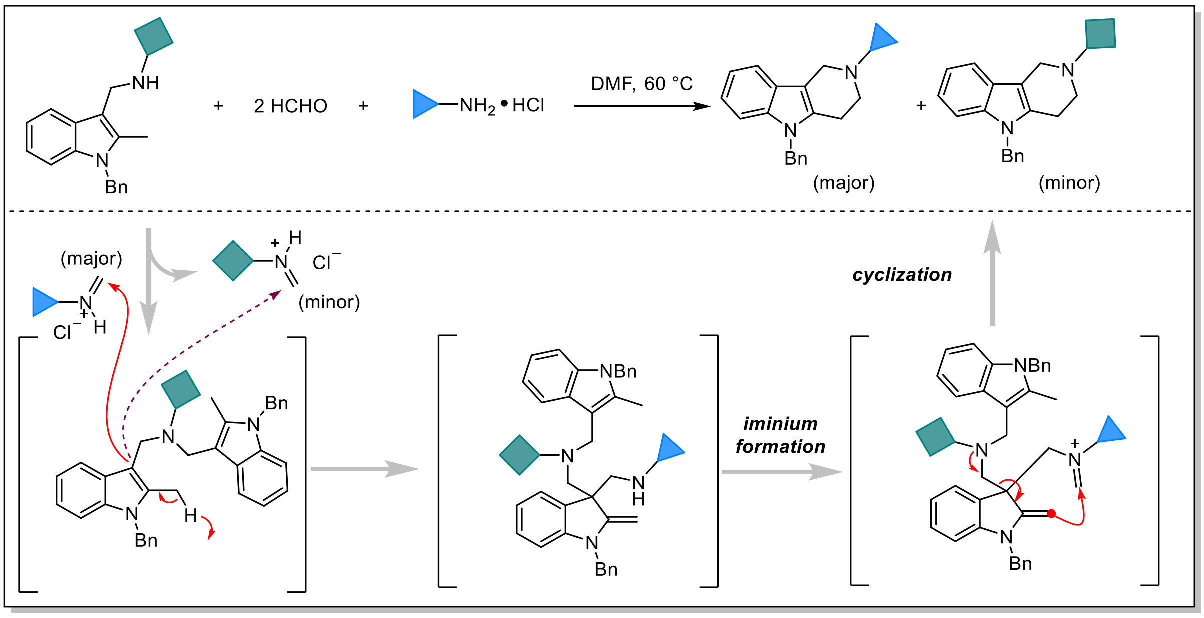

Supplementary Figure 4. Proposed reaction pathway of cross-over reactions.

Afterwards, the tertiary amine substituted indole **20** was employed to the cross-over experiment.

A mixture of **20** (0.1 mmol), formaldehyde **2** (37% in water, 0.08 mL, 10 equiv.) and **3a** (0.4 mmol, 4 equiv.) in DMF (1.5 mL) was stirred at 60 °C for 3 hours. The reaction was detected every 20 minutes. As expected, **4a** was not observed in the reaction, because the tertiary amine **20** could not form the di-alkylaminated intermediate (**Int-4**) thus failing to drive the following cascade.

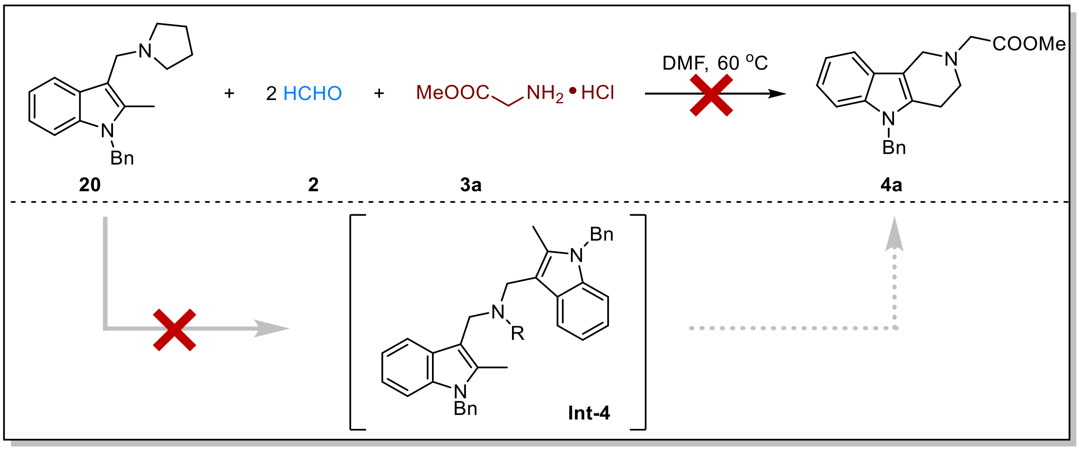

6.5 Procedure for the synthesis of 22 and 23[13]

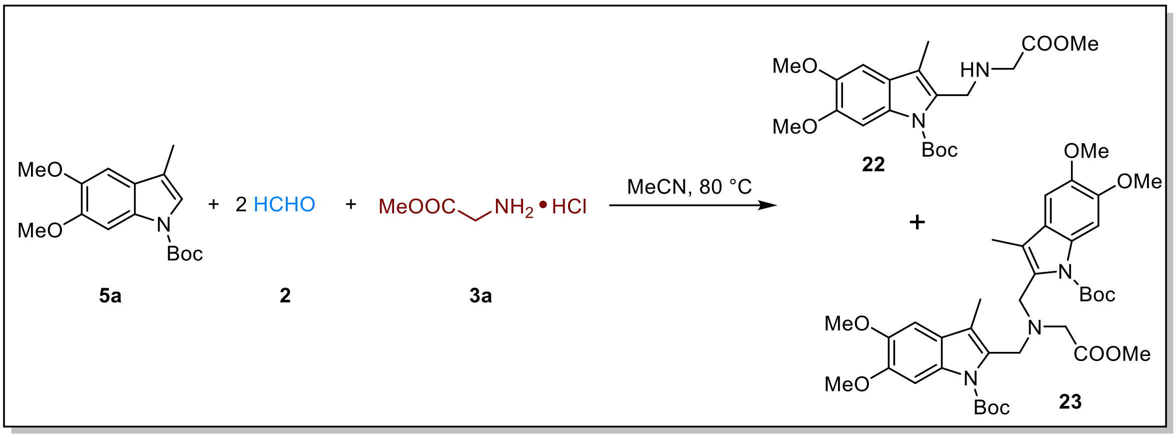

A mixture of **5a** (58 mg, 0.2 mmol), formaldehyde **2** (37% in water, 0.08 mL, 5 equiv.) and **3a** (50 mg, 0.4 mmol) in MeCN (1 mL) was stirred at 80 °C for 30 minutes. The reaction was quenched by saturated aqueous $NaHCO_3$. The aqueous layer was extracted with ethyl acetate (three times), and the combined organic layer was dried over Na_2SO_4 and concentrated. Purification by silica gel column chromatography to give the intermediates **22** and **23**.

6.6 Deuterium-labelling and stepwise control reaction for β-tetrahydrocarboline

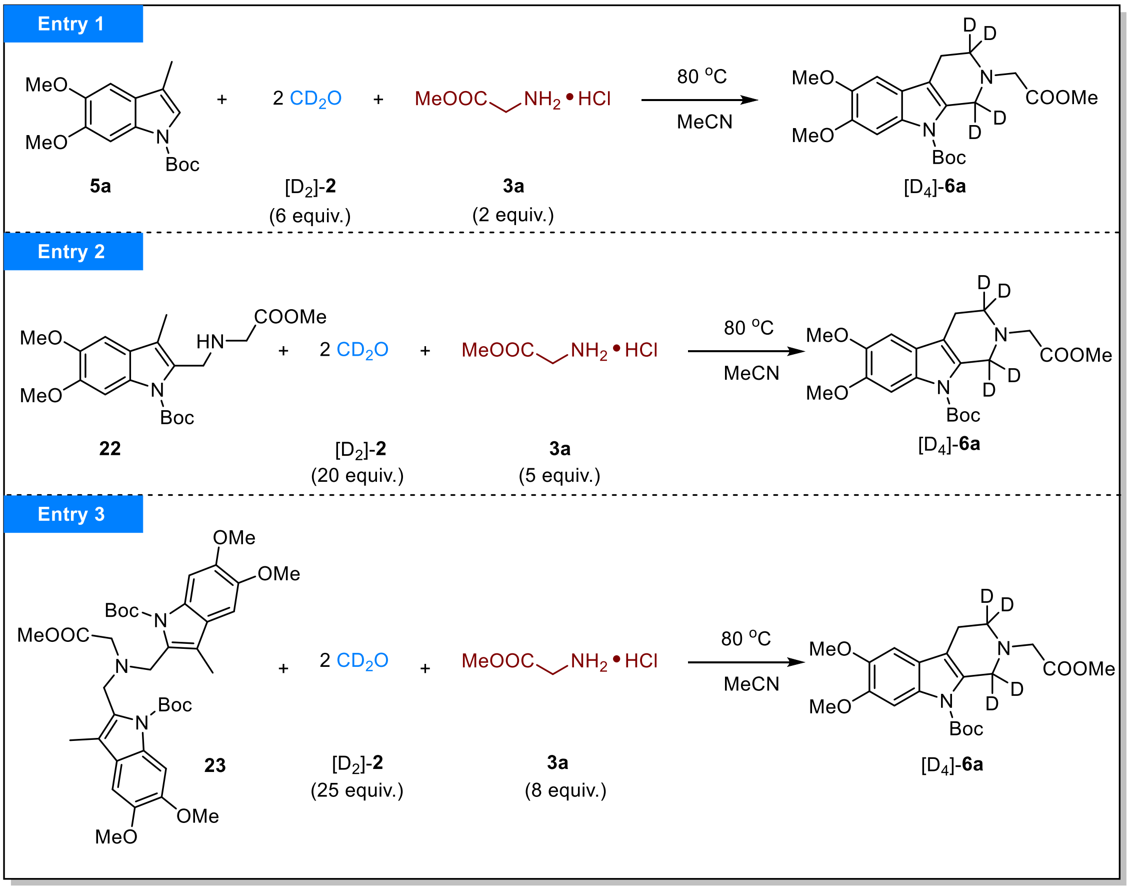

Entry 1: A mixture of **5a** (0.2 mmol), CD_2O **2** (20% in water, 0.2 mL, 6 equiv.) and corresponding primary amine hydrochloride **3** (0.4 mmol, 2 equiv.) in MeCN (1 mL) was stirred at 80 °C until the reaction was completed. The reaction was quenched by saturated aqueous $NaHCO_3$. The aqueous layer was extracted with ethyl acetate (three times), and the combined organic layer was dried over Na_2SO_4 and concentrated. Purification by silica gel column chromatography to give the product [D_4]-**6a**.

Entry 2: A mixture of **22** (39 mg, 0.1 mmol), CD_2O **2** (20% in D_2O, 0.3 mL, 20 equiv.) and **3a** (62.5 mg, 0.5 mmol) in MeCN (1 mL) was stirred at 80 °C until the reaction was completed. The reaction was quenched by saturated aqueous $NaHCO_3$. The aqueous layer was extracted with ethyl acetate (three times), and the combined organic layer was dried over Na_2SO_4 and concentrated. Purification by silica gel column chromatography to give the product [D_4]-**6a**.

Entry 3: A mixture of **23** (35 mg, 0.05 mmol), CD_2O **2** (20% in D_2O, 0.2 mL, 25 equiv.) and **3a** (60 mg, 0.4 mmol) in MeCN (1 mL) was stirred at 80 °C until the reaction was completed. The reaction was quenched by saturated aqueous $NaHCO_3$. The aqueous layer was extracted with

ethyl acetate (three times), and the combined organic layer was dried over Na_2SO_4 and concentrated. Purification by silica gel column chromatography to give the product [D_4]-**6a**.

For deuterium-labelling reaction of β-tetrahydrocarbolines, the product [D_4]-**6a** could be obtained by all entries. As expected, the position-15 is almost deuterated methylene (Supplementary Figure 5). As shown in the Supplementary Figure 5. **Entry 2** and **3**, the methylene at position-13 was almost deuterized, which was probably same as the result of γ-tetrahydrocarbolines.

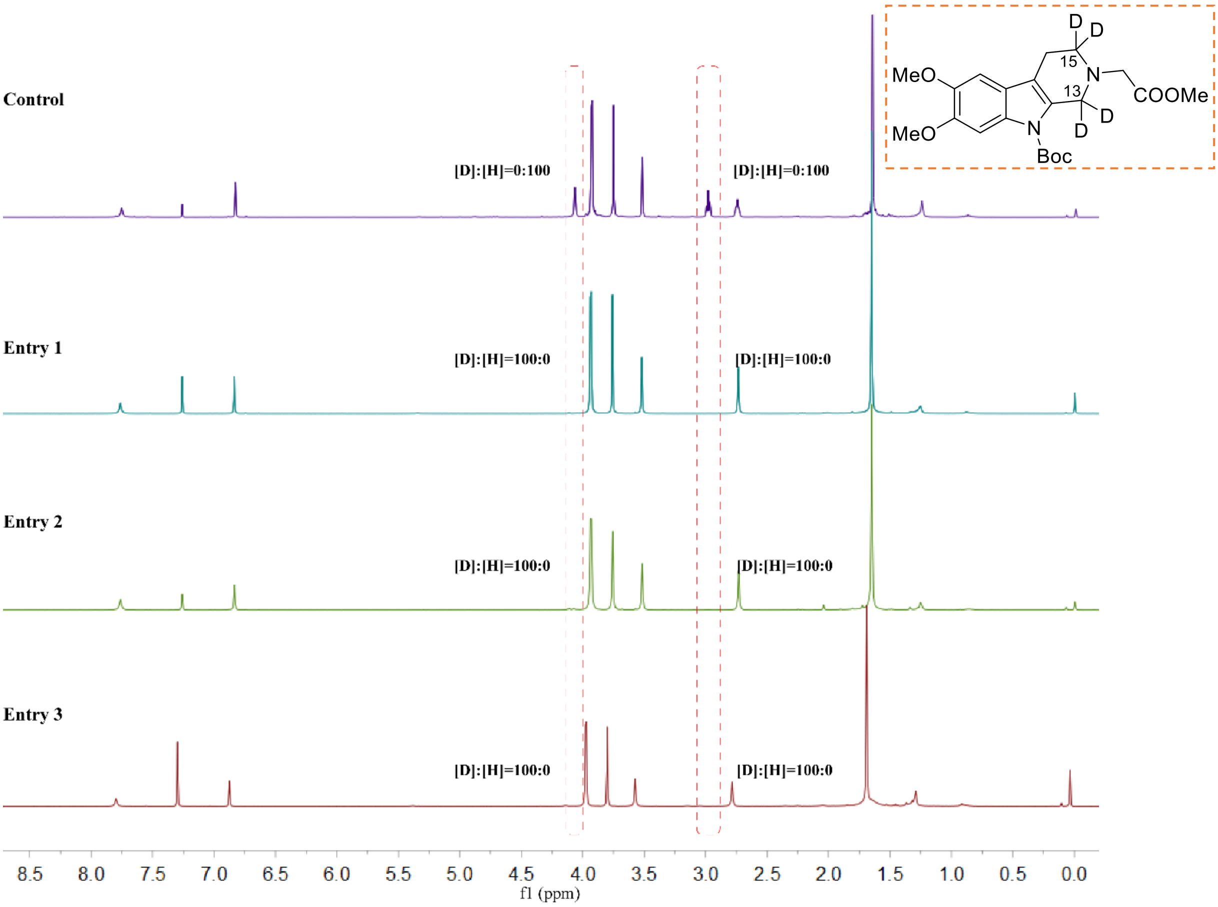

Supplementary Figure 5. ^{1}H NMR Comparison of Deuterium-labelling experiments for [D_4]-**6a**.

6.7 Cross-over reaction of β-tetrahydrocarboline

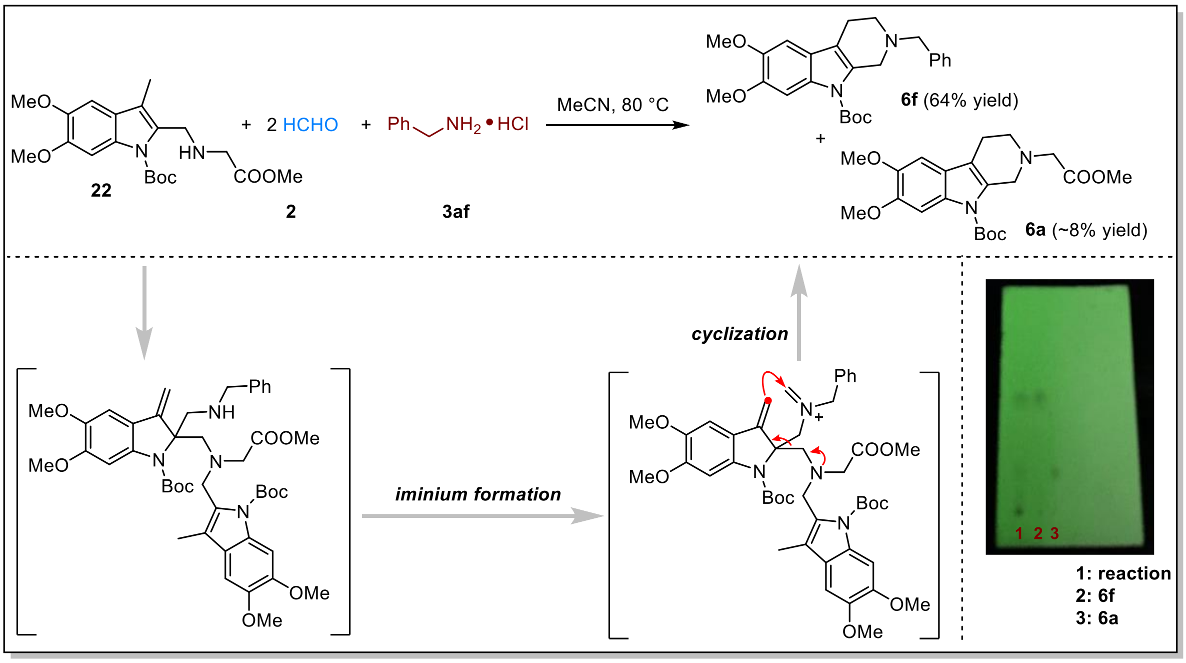

A mixture of **22** (39 mg, 0.1 mmol), formaldehyde **2** (37% in water, 0.04 mL, 5 equiv.) and benzylamine hydrochloride **3af** (0.2 mmol, 2 equiv.) in MeCN (1.5 mL) was stirred at 80 °C for 3 hours. The reaction was quenched by saturated aqueous $NaHCO_3$. The aqueous layer was extracted with ethyl acetate (three times), and the combined organic layer was dried over Na_2SO_4 and concentrated. Purification by silica gel column chromatography to give corresponding indole alkaloids products **6f** (27 mg, 64%) and **6a** (3 mg, 8%).

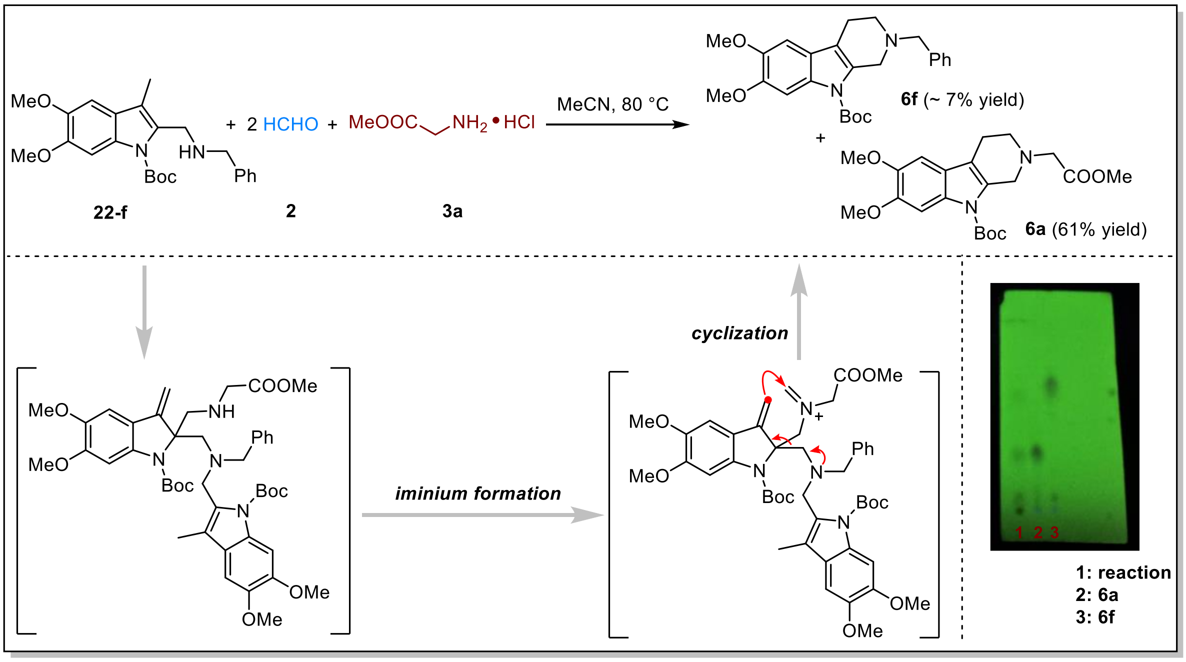

A mixture of **22-f** (41 mg, 0.1 mmol), formaldehyde **2** (37% in water, 0.04 mL, 5 equiv.) and **3a** (0.2 mmol, 2 equiv.) in MeCN (1.5 mL) was stirred at 80 °C for 3 hours. The reaction was quenched by saturated aqueous $NaHCO_3$. The aqueous layer was extracted with ethyl acetate (three times), and the combined organic layer was dried over Na_2SO_4 and concentrated. Purification by silica gel column chromatography to give corresponding indole alkaloids products **6a** (25 mg, 61%) and **6f** (3 mg, 7%).

In summary, we have conducted several experiments for investigating the mechanism of β-tetrahydrocarbolines, whose results illustrating the probably same mechanism as γ-tetrahydrocarbolines.

6.8 Synthesis of [D$_2$]-4a and [D$_2$]-6a

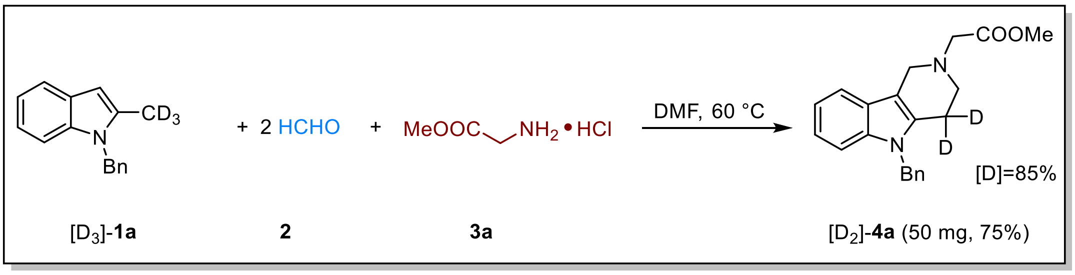

An oven-dried Schlenk tube equipped with a magnetic stirrer bar was charged with [D$_3$]-**1a** (45 mg, 0.2 mmol), **2** (37% in water, 0.08 mL, 5 equiv.) and **3a** (50 mg, 0.4 mmol, 2 equiv.), Then 1.5 mL DMF was added and the solution was stirred at 60 °C for 3 hours. The reaction was quenched by the saturated aqueous $NaHCO_3$, and the aqueous layer was extracted with ethyl acetate (three times). The combined organic layer was dried over Na_2SO_4 and concentrated. The crude product was purified by silica gel column chromatography to give the product [D$_2$]-**4a** (50 mg, 75% yield).

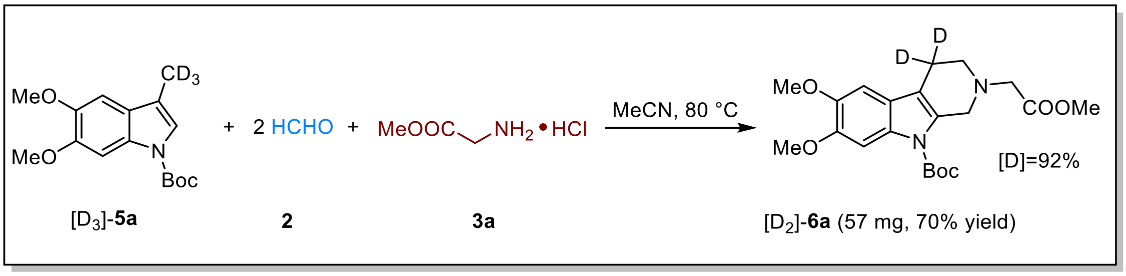

An oven-dried Schlenk tube equipped with a magnetic stirrer bar was charged with [D$_3$]-**5a** (59 mg, 0.2 mmol), **2** (37% in water, 0.08 mL, 5 equiv.) and **3a** (50 mg, 0.4 mmol, 2 equiv.), Then 1.5 mL MeCN was added and the solution was stirred at 80 °C for 6 hours. The reaction was

quenched by the saturated aqueous $NaHCO_3$, and the aqueous layer was extracted with ethyl acetate (three times). The combined organic layer was dried over Na_2SO_4 and concentrated. The crude product was purified by silica gel column chromatography to give the product [D_2]-**6a** (57 mg, 70% yield).

6.9 Kinetic isotopic effect experiments

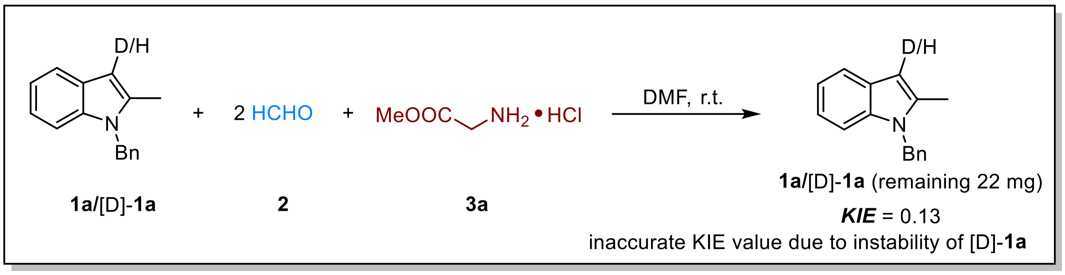

An oven-dried Schlenk tube equipped with a magnetic stirrer bar was charged with [D]-**1a** (22 mg, 0.1 mmol, 1 equiv.), **1a** (22 mg, 0.1 mmol, 1 equiv.) **2** (37% in water, 0.08 mL, 10 equiv.) and **3a** (50 mg, 0.4 mmol, 4 equiv.), Then 1.5 mL DMF was added and the solution was stirred at room temperature for 10 seconds. The reaction was quenched by the saturated aqueous $NaHCO_3$, and the aqueous layer was extracted with ethyl acetate (three times). The combined organic layer was dried over Na_2SO_4 and concentrated. The crude product was purified by column chromatography on silica gel eluted with petroleum ether/ethyl acetate (v/v, 5/1) to afford the [D]-**1a** and **1a** (remaining 22 mg, *KIE* = 0.13).

Due to the *KIE* value being much less than 1.0, we conducted stability experiments on the compound [D]-**1a**. Due to the high boiling point of DMF, ^1H NMR results have no obvious comparison. Therefore, the solvent was replaced with DCM. A Schlenk tube was charged with [D]-**1a** (22 mg), DCM was added as solvent and the mixture was kept at room temperature. The samples were collected at 0.5 h, 1 h. All samples were concentrated under vacuum and analysed with ^1H NMR spectra. As shown in the Supplementary Figure 6, the compound [D]-**1a** could transform into **1a** spontaneously. Thus, the inaccurate KIE vale of [D]-**1a** is due to its instability.

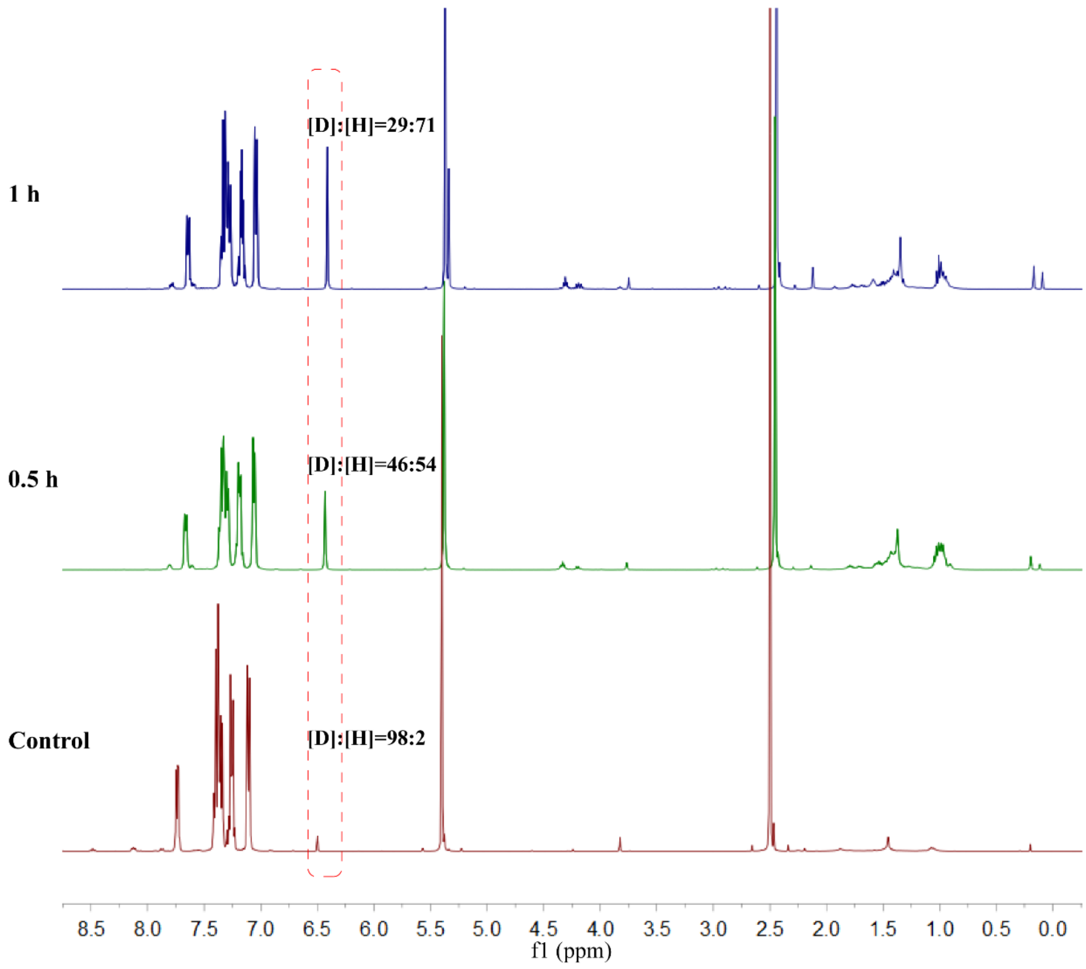

Supplementary Figure 6. ^{1}H NMR comparison of deuterium-labelling experiments for [D]-**1a**.

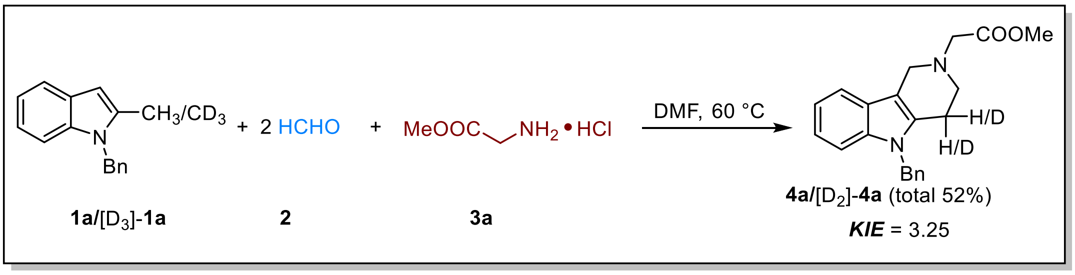

An oven-dried Schlenk tube equipped with a magnetic stirrer bar was charged with [D_3]-**1a** (22 mg, 0.1 mmol, 1 equiv.), **1a** (22 mg, 0.1 mmol, 1 equiv.) **2** (37% in water, 0.08 mL, 10 equiv.) and **3a** (50 mg, 0.4 mmol, 4 equiv.), Then 1.5 mL DMF was added and the solution was stirred at 60 °C for 30 minutes. The reaction was quenched by the saturated aqueous $NaHCO_3$, and the aqueous layer was extracted with ethyl acetate (three times). The combined organic layer was

dried over Na_2SO_4 and concentrated. The crude product was purified by silica gel column chromatography to give the product [D_2]-**4a** and **4a** (35 mg, 52% yield, *KIE* = 3.25).

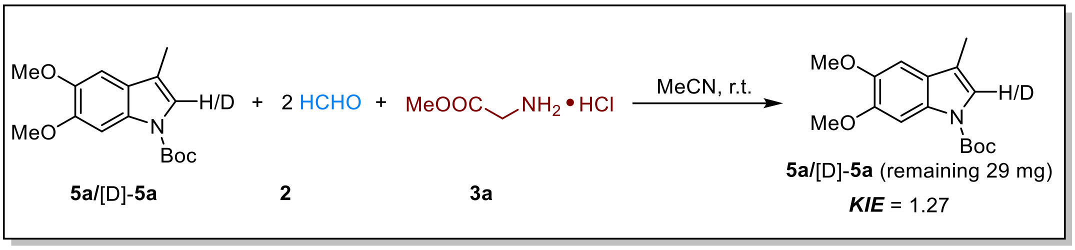

An oven-dried Schlenk tube equipped with a magnetic stirrer bar was charged with [D]-**5a** (29 mg, 0.1 mmol, 1 equiv.), **5a** (29 mg, 0.1 mmol, 1 equiv.) **2** (37% in water, 0.08 mL, 10 equiv.) and **3a** (50 mg, 0.4 mmol, 4 equiv.), Then 1.5 mL MeCN was added and the solution was stirred at room temperature for 2 minutes. The reaction was quenched by the saturated aqueous $NaHCO_3$, and the aqueous layer was extracted with ethyl acetate (three times). The combined organic layer was dried over Na_2SO_4 and concentrated. The crude product was purified by silica gel column chromatography to afford the [D]-**5a** and **5a** (remaining 29 mg, *KIE* = 1.27).

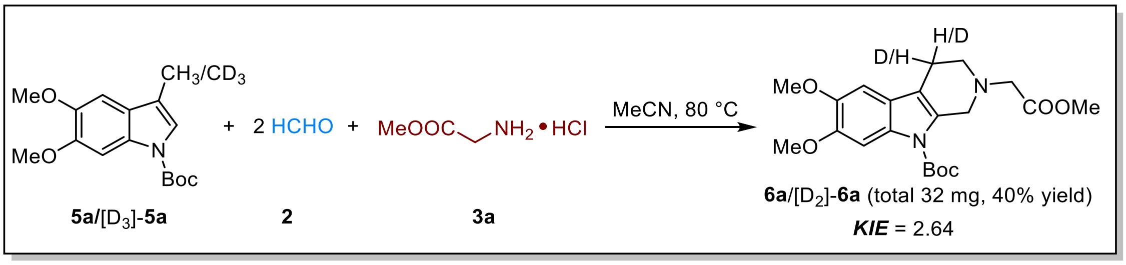

An oven-dried Schlenk tube equipped with a magnetic stirrer bar was charged with [D_3]-**5a** (29 mg, 0.1 mmol, 1 equiv.), **5a** (29 mg, 0.1 mmol, 1 equiv.) **2** (37% in water, 0.08 mL, 10 equiv.) and **3a** (50 mg, 0.4 mmol, 4 equiv.), Then 1.5 mL MeCN was added and the solution was stirred at 80 ^{o}C for 40 minutes. The reaction was quenched by the saturated aqueous $NaHCO_3$, and the aqueous layer was extracted with ethyl acetate (three times). The combined organic layer was dried over Na_2SO_4 and concentrated. The crude product was purified by silica gel column chromatography to give the product [D_2]-**6a** and **6a** (32 mg, 40% yield, *KIE* = 2.64).

7. Characterization of Corresponding Compounds

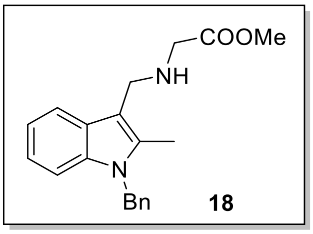

methyl ((1-benzyl-2-methyl-1*H*-indol-3-yl)methyl)glycinate (18): light yellow gel, 38 mg, 60% yield. **^{1}H NMR (500 MHz, CDCl$_3$)** δ 7.68 (dd, J_1 = 6.0 Hz, J_2 = 3.2 Hz, 1H), 7.24 – 7.13 (m, 4H), 7.10 – 7.01 (m, 2H), 6.91 (d, *J* = 7.2 Hz, 2H), 5.24 (s, 2H), 4.03 (s, 2H), 3.75 (s, 1H), 3.48 (s, 3H), 3.44 (s, 2H), 2.28 (s, 3H). **^{13}C NMR (126 MHz, CDCl$_3$)** δ 172.7, 138.1, 136.6, 135.4, 128.9, 128.7, 127.3, 126.0, 121.0, 119.5, 118.9, 109.0, 108.7, 51.16, 51.0, 46.5, 46.1, 10.3. **HRMS (ESI-TOF)** calcd for $C_{20}H_{22}N_2O_2$ (M+Na$^+$): 345.1573; Found:345.1575.

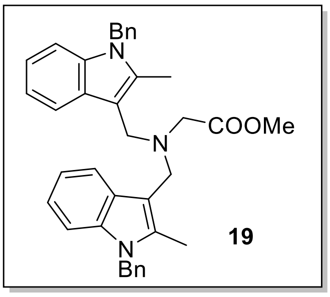

methyl bis((1-benzyl-2-methyl-1*H*-indol-3-yl)methyl)glycinate (19): light yellow gel, 10 mg, 18% yield. **^{1}H NMR (400 MHz, CDCl$_3$)** δ 7.68 (d, *J* = 7.2 Hz, 2H), 7.22 (m, 8H), 7.14 – 7.03 (m, 4H), 6.95 (d, *J* = 6.8 Hz, 4H), 5.32 (s, 4H), 4.06 (s, 4H), 3.59 (s, 3H), 3.30 (s, 2H), 2.33 (s, 6H). **^{13}C NMR (101 MHz, CDCl$_3$)** δ 172.7, 138.0, 136.5, 135.4, 128.8, 127.2, 126.0, 120.9, 119.3, 118.9, 109.1, 108.9, 52.5, 50.9, 47.9, 46.5, 10.3. **HRMS (ESI-TOF)** calcd for $C_{37}H_{37}N_3O_2$ (M+H$^+$): 556.2959; Found: 556.2965.

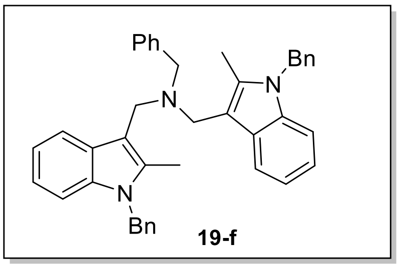

***N*-benzyl-1-(1-benzyl-2-methyl-1*H*-indol-3-yl)-*N*-((1-benzyl-2-methyl-1*H*-indol-3-yl)methyl)methanamine (19-f):** colorless gel, 40% yield. **^{1}H NMR (400 MHz, CDCl$_3$)** δ 7.63 (d, *J* = 7.2 Hz, 2H), 7.33 (d, *J* = 8.8 Hz, 3H), 7.28 – 7.20 (m, 10H), 7.08 (d, *J* = 8.4 Hz, 4H), 6.95

(d, J = 5.2 Hz, 4H), 5.34 (s, 4H), 3.74 (s, 4H), 3.57 (s, 2H), 2.28 (s, 6H). **^{13}C NMR (101 MHz, CDCl$_3$)** δ 141.0, 138.2, 136.4, 135.1, 129.4, 129.0, 128.8, 127.9, 127.2, 126.6, 125.9, 120.7, 119.1, 118.8, 109.8, 108.8, 58.8, 48.6, 46.4, 10.4. **HRMS (ESI-TOF)** calcd for $C_{41}H_{39}N_3$ ($M+H^+$): 574.3217; Found: 574.3219.

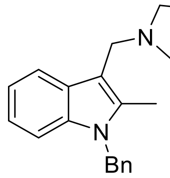

20

1-benzyl-2-methyl-3-(pyrrolidin-1-ylmethyl)-1*H*-indole (20): light yellow gel, 46 mg, 75% yield. **^{1}H NMR (400 MHz, CDCl$_3$)** δ 7.54 (dd, J_1 = 6.0 Hz, J_2 = 2.4 Hz, 1H), 7.28 (t, J = 4.4 Hz, 3H), 7.23 (d, J = 7.2 Hz, 1H), 7.19 (dd, J_1 = 6.0 Hz, J_2 = 3.2 Hz, 2H), 6.96 (d, J = 6.8 Hz, 2H), 5.35 (s, 2H), 4.43 (s, 2H), 3.43 (m, 4H), 2.51 (s, 3H), 2.08 (s, 4H). **^{13}C NMR (101 MHz, CDCl$_3$)** δ 139.3, 136.7, 136.7, 129.0, 128.0, 127.7, 126.0, 122.2, 120.9, 117.3, 110.0, 100.0, 51.6, 47.0, 46.8, 22.9, 11.5. **HRMS (ESI-TOF)** calcd for $C_{21}H_{24}N_2$ ($M+H^+$): 305.2012; Found: 305.2006.

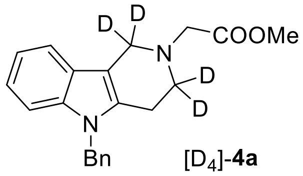

[D$_4$]-**4a**

methyl 2-(5-benzyl-1,3,4,5-tetrahydro-2*H*-pyrido[4,3-*b*]indol-2-yl-1,1,3,3-*d*$_4$)acetate ([D$_4$]-4a): According to the procedure for 3 h, light yellow gel, 61 mg, 90% yield. **^{1}H NMR (400 MHz, CDCl$_3$)** δ 7.48 (d, J = 7.6 Hz, 1H), 7.37 – 7.22 (m, 4H), 7.20 – 7.08 (m, 2H), 7.05 (d, J = 7.2 Hz, 2H), 5.29 (s, 2H), 3.81 (s, 3H), 3.58 (s, 2H), 2.83 (s, 2H). **^{13}C NMR (101 MHz, CDCl$_3$)** δ 171.3, 137.9, 136.8, 133.3, 128.8, 127.4, 126.2, 125.9, 121.1, 119.2, 117.7, 109.3, 107.7, 58.4, 51.9, 46.4, 22.3. **HRMS (ESI-TOF)** calcd for $C_{21}H_{18}D_4N_2O_2$ ($M+H^+$): 339.2005; Found: 339.2001.

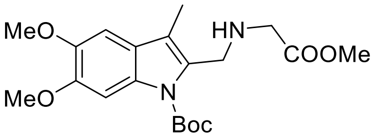

22

***tert*-butyl 5,6-dimethoxy-2-(((2-methoxy-2-oxoethyl)amino)methyl)-3-methyl-1*H*-indole-1-carboxylate (22):** light yellow gel, 50% yield. Mixture of two rotamers. **^{1}H NMR (400 MHz, $CDCl_3$)** δ 7.64 (m, 1H), 6.85 (m, 1H), 4.12 (m, 2H), 3.92 (m, 6H), 3.59 (s, 0.3H), 3.50 (m, 2H), 3.37 (m, 2H), 3.20 (s, 0.7H), 2.41 (s, 1H), 2.17 (m, 3H), 1.67 (m, 9H). **^{13}C NMR (101 MHz, $CDCl_3$)** δ 172.7, 172.3, 150.5, 150.1, 147.6, 147.3, 146.3, 145.9, 131.8, 131.5, 130.1, 129.9, 122.8, 117.4, 100.3, 99.9, 99.6, 83.8, 82.8, 56.2, 56.1, 51.7, 50.6, 49.8, 49.0, 46.2, 43.7, 28.3, 28.3, 9.0. **HRMS (ESI-TOF)** calcd for $C_{20}H_{28}N_2O_6$ (M+Na$^+$): 415.1840; Found: 415.1838.

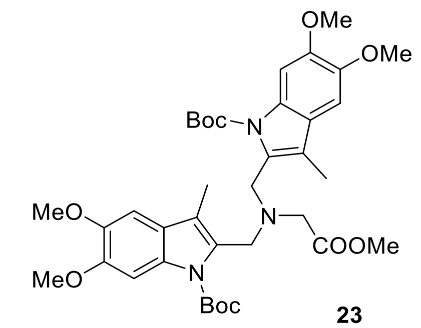

23

di-*tert*-butyl 2,2'-(((2-methoxy-2-oxoethyl)azanediyl)bis(methylene))bis(5,6-dimethoxy-3-methyl-1*H*-indole-1-carboxylate) (23): colorless gel, 8% yield. Mixture of two rotamers. **^{1}H NMR (400 MHz, $CDCl_3$)** δ 7.57 (s, 1.6H), 7.34 (s, 0.4H), 6.79 (s, 1.7H), 6.62 (s, 0.3H), 4.33 (s, 3.2H), 3.96 (s, 0.8H), 3.92 (m, 12H), 3.69 (s, 0.4H), 3.66 (s, 0.3H), 3.56 (s, 2.6H), 3.38 (s, 1.7H), 2.51 (s, 0.8H), 2.11 (s, 5H), 1.62 (s, 15.2H), 1.50 (s, 2.7H). **^{13}C NMR (101 MHz, $CDCl_3$)** δ 172.8, 150.3, 147.2, 146.0, 132.2, 130.2, 123.0, 117.7, 100.1, 99.7, 82.9, 56.2, 56.1, 53.4, 50.9, 48.0, 28.3, 9.0. **HRMS (ESI-TOF)** calcd for $C_{37}H_{49}N_3O_{10}$ (M+H$^+$): 696.3491; Found: 696.3490.

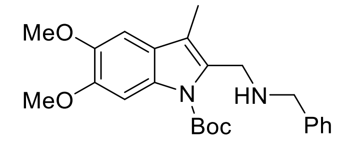

22-f

***tert*-butyl 2-((benzylamino)methyl)-5,6-dimethoxy-3-methyl-1*H*-indole-1-carboxylate (22-f):** colorless gel, 67% yield. **^{1}H NMR (400 MHz, $CDCl_3$)** δ 7.75 (s, 1H), 7.40 – 7.31 (m, 4H), 7.25 (d, *J* = 7.2 Hz, 1H), 6.93 (s, 1H), 4.11 (s, 2H), 3.99 (d, *J* = 2.8 Hz, 6H), 3.80 (s, 2H), 2.65 (s, 1H), 2.18 (s, 3H), 1.74 (s, 9H). **^{13}C NMR (101 MHz, $CDCl_3$)** δ 150.7, 147.5, 146.4, 140.6, 132.7, 129.8, 128.3, 128.1, 126.8, 123.2, 117.0, 100.4, 100.1, 83.6, 56.3, 56.2, 52.0, 43.8, 28.4, 9.1. **HRMS (ESI-TOF)** calcd for $C_{24}H_{30}O_2N_4$ (M+H$^+$): 411.2278; Found: 411.2278.

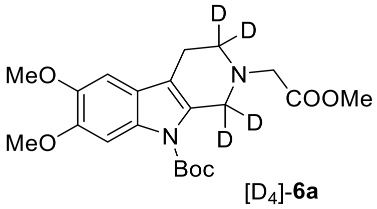

[D4]-**6a**

***tert*-butyl 6,7-dimethoxy-2-(2-methoxy-2-oxoethyl)-1,2,3,4-tetrahydro-9*H*-pyrido[3,4-*b*]indole-9-carboxylate-1,1,3,3-*d*4** ([D4]-**6a):** light yellow gel, 50 mg, 61% yield. **^{1}H NMR (400 MHz, CDCl$_3$)** δ 7.76 (s, 1H), 6.84 (s, 1H), 3.94 (d, J = 3.2 Hz, 6H), 3.76 (s, 3H), 3.52 (s, 2H), 2.73 (s, 2H), 1.65 (s, 9H). **^{13}C NMR (101 MHz, CDCl$_3$)** δ 171.1, 150.3, 147.1, 146.3, 129.8, 121.9, 114.5, 99.8, 99.8, 83.5, 57.8, 56.2, 56.2, 51.8, 28.3, 20.7. **HRMS (ESI-TOF)** calcd for $C_{21}H_{24}D_4N_2O_6$ (M+H$^+$): 409.2271; Found: 409.2267.

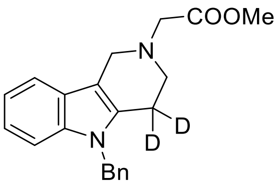

[D2]-**4a**

methyl 2-(5-benzyl-1,3,4,5-tetrahydro-2*H*-pyrido[4,3-*b*]indol-2-yl-4,4-*d*2)acetate ([D2]-**4a):** light yellow gel, 50 mg, 75% yield. **^{1}H NMR (400 MHz, CDCl$_3$)** δ 7.51 – 7.44 (m, 1H), 7.34 – 7.22 (m, 4H), 7.19 – 7.10 (m, 2H), 7.05 (m, 2H), 5.29 (s, 2H), 3.98 (s, 2H), 3.81 (s, 3H), 3.58 (s, 2H), 3.07 (s, 2H). **^{13}C NMR (101 MHz, CDCl$_3$)** δ 171.2, 137.9, 136.9, 133.2, 128.8, 127.4, 126.2, 125.8, 121.1, 119.2, 117.7, 109.3, 107.9, 58.6, 51.8, 50.2, 49.5, 46.4. **HRMS (ESI-TOF)** calcd for $C_{21}H_{20}D_2N_2O_2$ (M+H$^+$): 337.1880; Found: 337.1881.

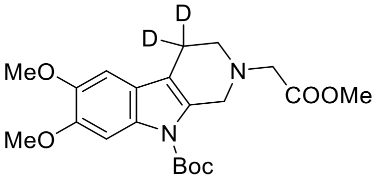

[D2]-**6a**

***tert*-butyl 6,7-dimethoxy-2-(2-methoxy-2-oxoethyl)-1,2,3,4-tetrahydro-9*H*-pyrido[3,4-*b*]indole-9-carboxylate-4,4-*d*2 (**[D2]-**6a):** light yellow gel, 57 mg, 70% yield. **^{1}H NMR (400 MHz, CDCl$_3$)** δ 7.76 (s, 1H), 6.83 (s, 1H), 4.07 (s, 2H), 3.93 (m, 6H), 3.76 (s, 3H), 3.52 (s, 2H), 2.97 (s, 2H), 1.65 (s, 9H). **^{13}C NMR (101 MHz, CDCl$_3$)** δ 171.1, 150.3, 147.0, 146.3, 130.8,

129.8, 122.0, 114.3, 99.8, 99.8, 83.5, 58.1, 56.2, 56.1, 52.2, 51.8, 49.3, 28.3. **HRMS (ESI-TOF)** calcd for $C_{21}H_{26}D_2N_2O_6$ (M+H$^+$): 407.2146; Found: 407.2141.

7. X-ray Crystallographic Data

7.1 X-Ray crystal structure of 4f

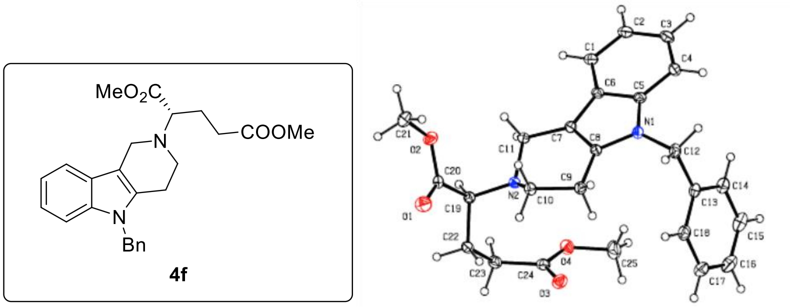

Supplementary Figure 7. X-Ray Crystallography of **4f**.

A single crystal of **4f** was obtained through slow evaporation from its solution in EtOAc. The structure and absolute configuration of **4f** was then determined by x-ray crystallographic analysis (CCDC No: 2211851).

Supplementary Table 8. Crystal data and structure refinement for **4f**.

**Compound**	**4f**
**CCDC code**	2211851
**Empirical formula**	$C_{25}H_{28}N_2O_4$
**Formula weight**	420.49
**Temperature/K**	170
**Crystal system**	triclinic
**Space group**	P1
**a/Å**	5.7505(6)
**b/Å**	10.1642(10)
**c/Å**	10.1660(11)
**α/°**	64.806(4)
**β/°**	86.086(4)
**γ/°**	89.691(4)
**Volume/Å^3**	536.23(10)
**Z**	1
**ρ_{calc}g/cm^3**	1.302
**μ/mm^{-1}**	0.455
**F(000)**	224

**Crystal size/mm^3**	0.03 × 0.02 × 0.01
**Radiation**	GaKα (λ = 1.34139)
**2Θ range for data collection/°**	8.366 to 118.814
**Index ranges**	$-7 \leq h \leq 7$<br>$-13 \leq k \leq 13$<br>$-12 \leq l \leq 12$
**Reflections collected**	17745
**Independent reflections**	4479 [R_{int} = 0.0516, R_{sigma} = 0.0497]
**Data/restraints/parameters**	4479/3/282
**Goodness-of-fit on F^2**	1.108
**Final R indexes [I>=2σ (I)]**	R_1 = 0.0416<br>wR_2 = 0.0959
**Final R indexes [all data]**	R_1 = 0.0452<br>wR_2 = 0.0978
**Largest diff. peak/hole / e Å^{-3}**	0.18/-0.20
**Flack parameter**	0.19(10)

7.2 X-Ray crystal structure of 4af

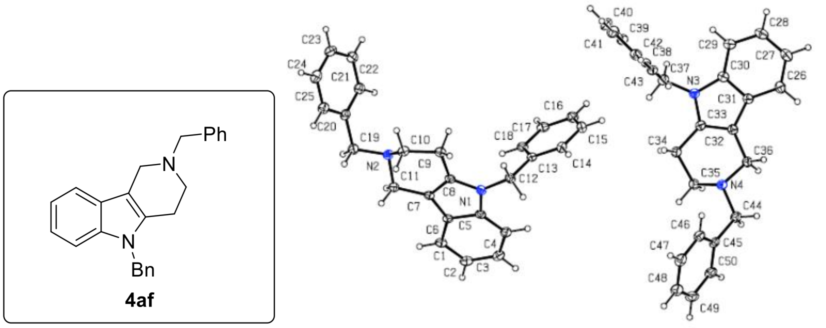

Supplementary Figure 8. X-Ray Crystallography of **4af**.

A single crystal of **4af** was obtained through slow evaporation from its solution in EtOAc. The structure of **4af** was then determined by x-ray crystallographic analysis (CCDC No: 2211850).

Supplementary Table 9. Crystal data and structure refinement for **4af**.

Compound	4af
**CCDC code**	2211850

**Empirical formula**	$C_{25}H_{24}N_2$
**Formula weight**	352.46
**Temperature/K**	170
**Crystal system**	triclinic
**Space group**	P-1
**a/Å**	8.2105(10)
**b/Å**	10.3212(13)
**c/Å**	22.424(3)
**α/°**	81.892(5)
**β/°**	87.983(5)
**γ/°**	87.261(5)
**Volume/Å^3**	1878.4(4)
**Z**	4
**ρ_{calc}g/cm^3**	1.246
**μ/mm^{-1}**	0.354
**F(000)**	752
**Crystal size/mm^3**	0.3 × 0.11 × 0.08
**Radiation**	GaKα (λ = 1.34139)
**2Θ range for data collection/°**	3.464 to 119.048
**Index ranges**	$-10 \leq h \leq 10$<br>$-13 \leq k \leq 13$<br>$-28 \leq l \leq 28$
**Reflections collected**	29938
**Independent reflections**	7947 [R_{int} = 0.0594, R_{sigma} = 0.0606]
**Data/restraints/parameters**	7947/0/487
**Goodness-of-fit on F^2**	1.272
**Final R indexes [I>=2σ (I)]**	R_1 = 0.0863<br>wR_2 = 0.2144
**Final R indexes [all data]**	R_1 = 0.0934<br>wR_2 = 0.2170
**Largest diff. peak/hole / e Å^{-3}**	0.39/-0.32

7.3 X-Ray crystal structure of 6v

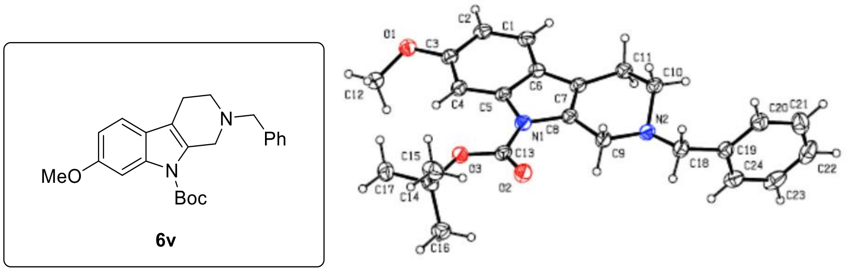

Supplementary Figure 9. X-Ray Crystallography of **6v**.

A single crystal of **6v** was obtained through slow evaporation from its solution in EtOAc. The structure of **6v** was then determined by x-ray crystallographic analysis (CCDC No: 2235528).

Supplementary Table 10. Crystal data and structure refinement for **6v**.

Compound	6v
**CCDC code**	2235528
**Empirical formula**	$C_{24}H_{28}N_2O_3$
**Formula weight**	392.48
**Temperature/K**	170
**Crystal system**	orthorhombic
**Space group**	$P2_12_12_1$
**a/Å**	6.1303(12)
**b/Å**	10.1600(18)
**c/Å**	33.259(6)
**α/°**	90
**β/°**	90
**γ/°**	90
**Volume/Å^3**	2071.5(7)
**Z**	4
**ρ_{calc}g/cm^3**	1.258
**μ/mm^{-1}**	0.422
**F(000)**	840
**Crystal size/mm^3**	0.35 × 0.04 × 0.03
**Radiation**	GaKα (λ = 1.34139)
**2Θ range for data collection/°**	7.916 to 121.744
**Index ranges**	$-7 \le h \le 7$<br>$-13 \le k \le 13$<br>$-43 \le l \le 42$
**Reflections collected**	27319
**Independent reflections**	4743 [R_{int} = 0.1291, R_{sigma} = 0.0955]
**Data/restraints/parameters**	4743/0/266
**Goodness-of-fit on F^2**	0.918
**Final R indexes [I>=2σ (I)]**	R_1 = 0.0654<br>wR_2 = 0.1487

**Final R indexes [all data]**	R_1 = 0.1083<br>wR_2 = 0.1704
**Largest diff. peak/hole / e Å^{-3}**	0.23/-0.30
**Flack parameter**	0.4(2)

8. Copies of NMR Spectra

^{1}H-NMR spectrum of compound **1b** (400 MHz, $CDCl_3$)

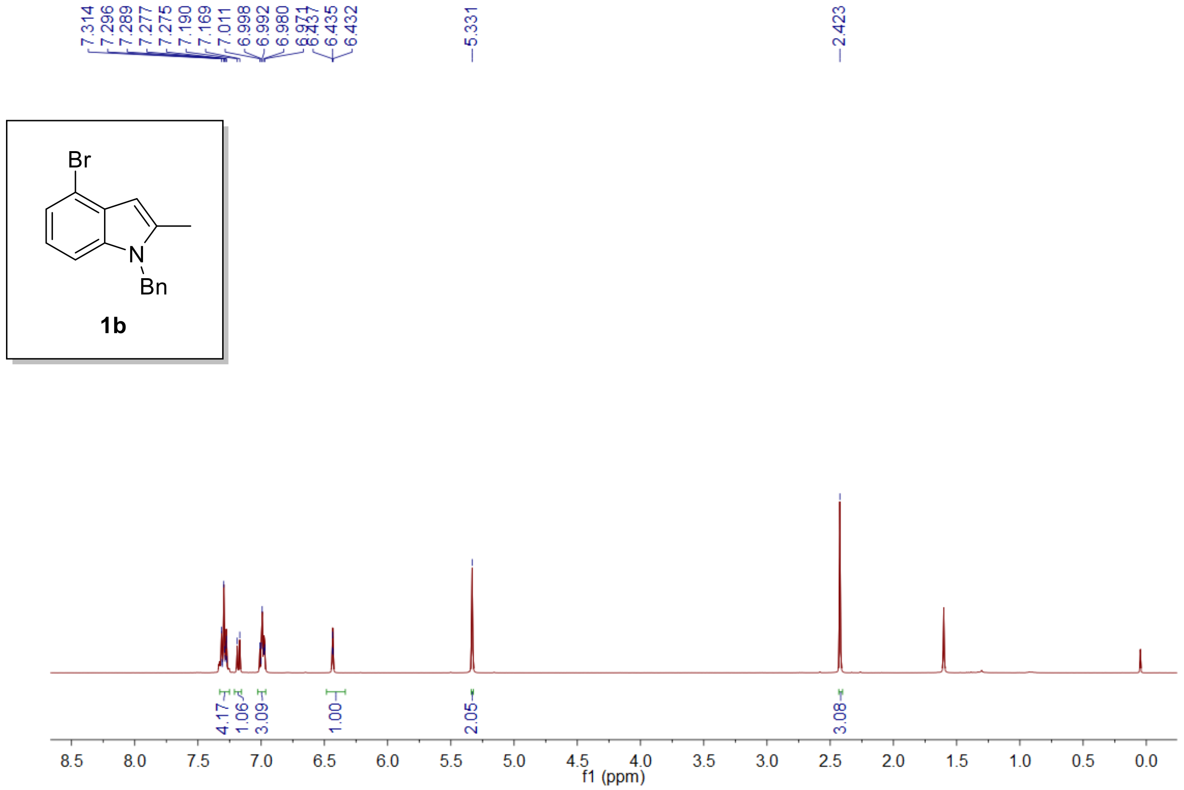

^{13}C-NMR spectrum of compound **1b** (101 MHz, $CDCl_3$)

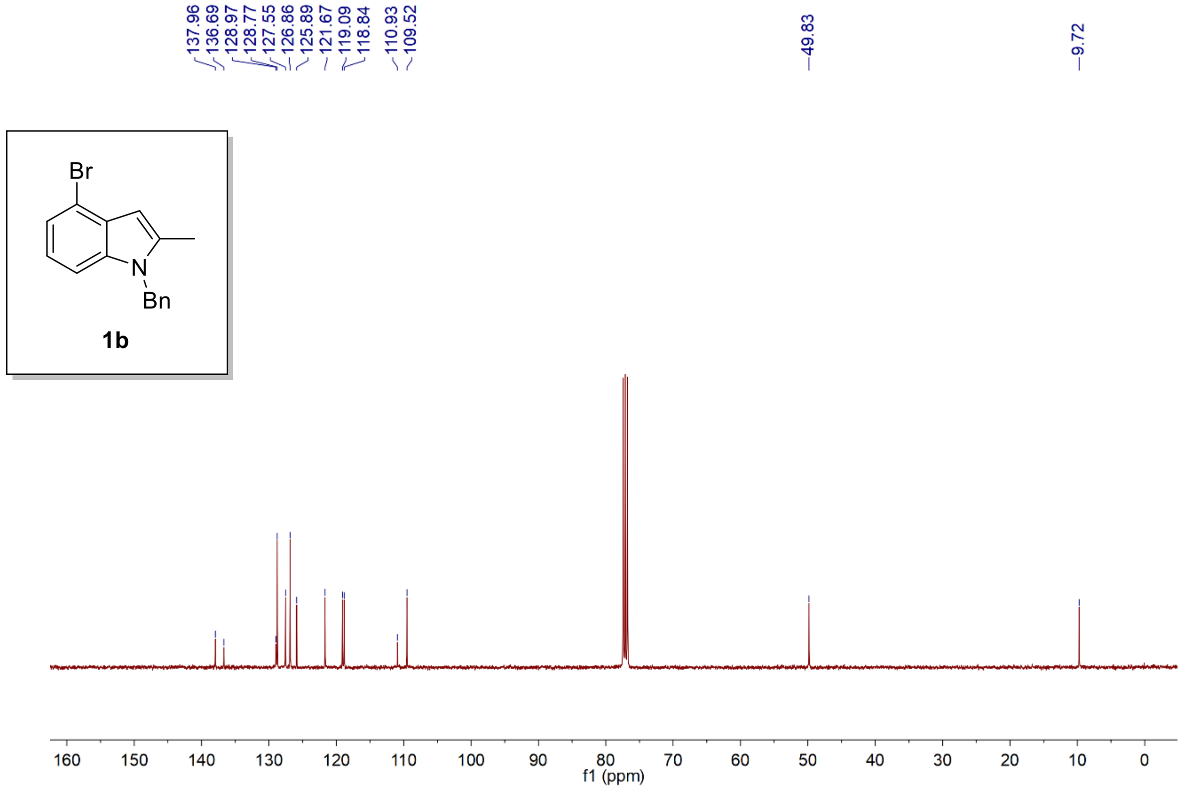

^{1}H-NMR spectrum of compound **1d** (400 MHz, $CDCl_3$)

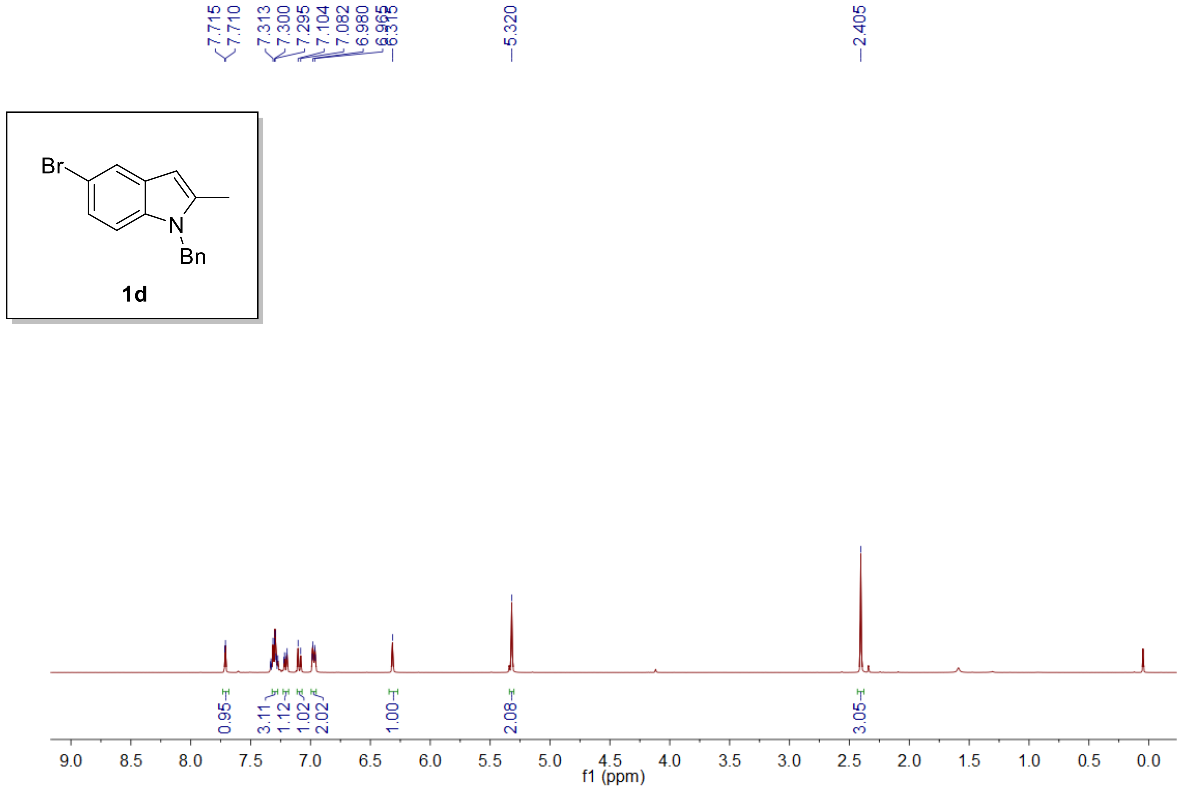

^{13}C-NMR spectrum of compound **1d** (101 MHz, $CDCl_3$)

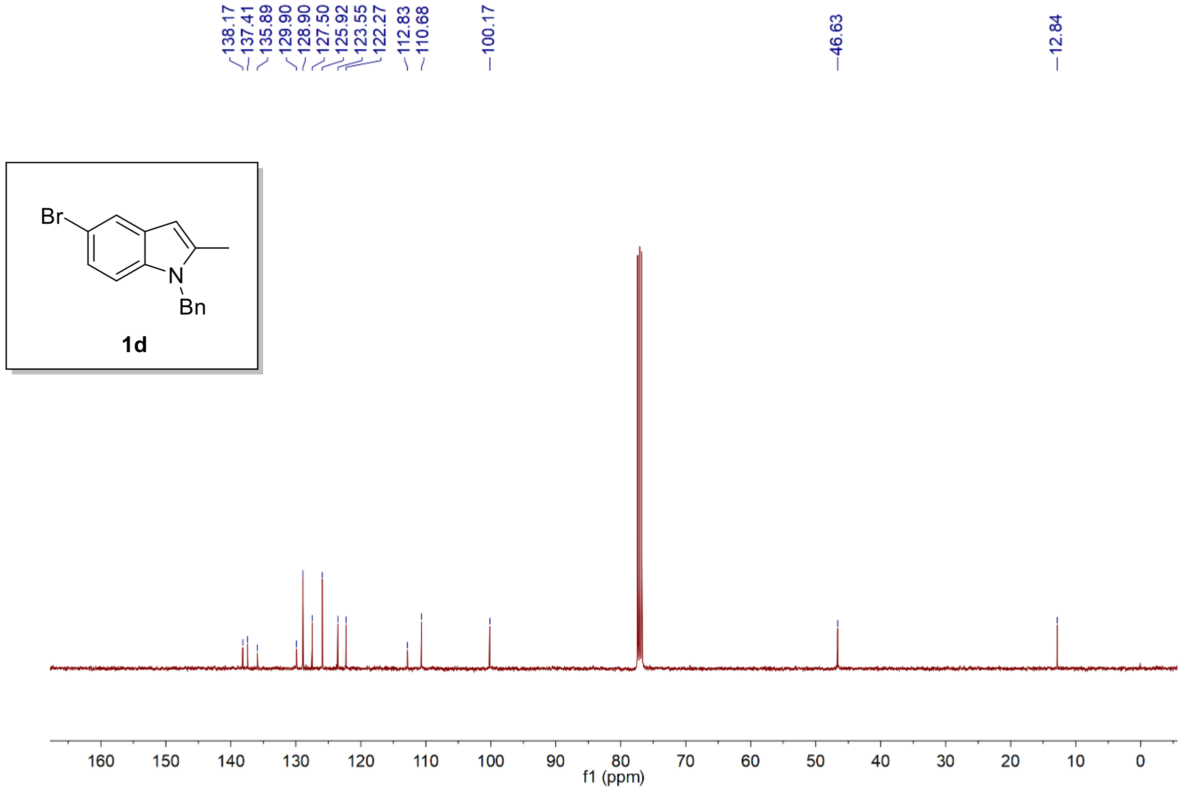

^{1}H-NMR spectrum of compound **1e** (400 MHz, $CDCl_3$)

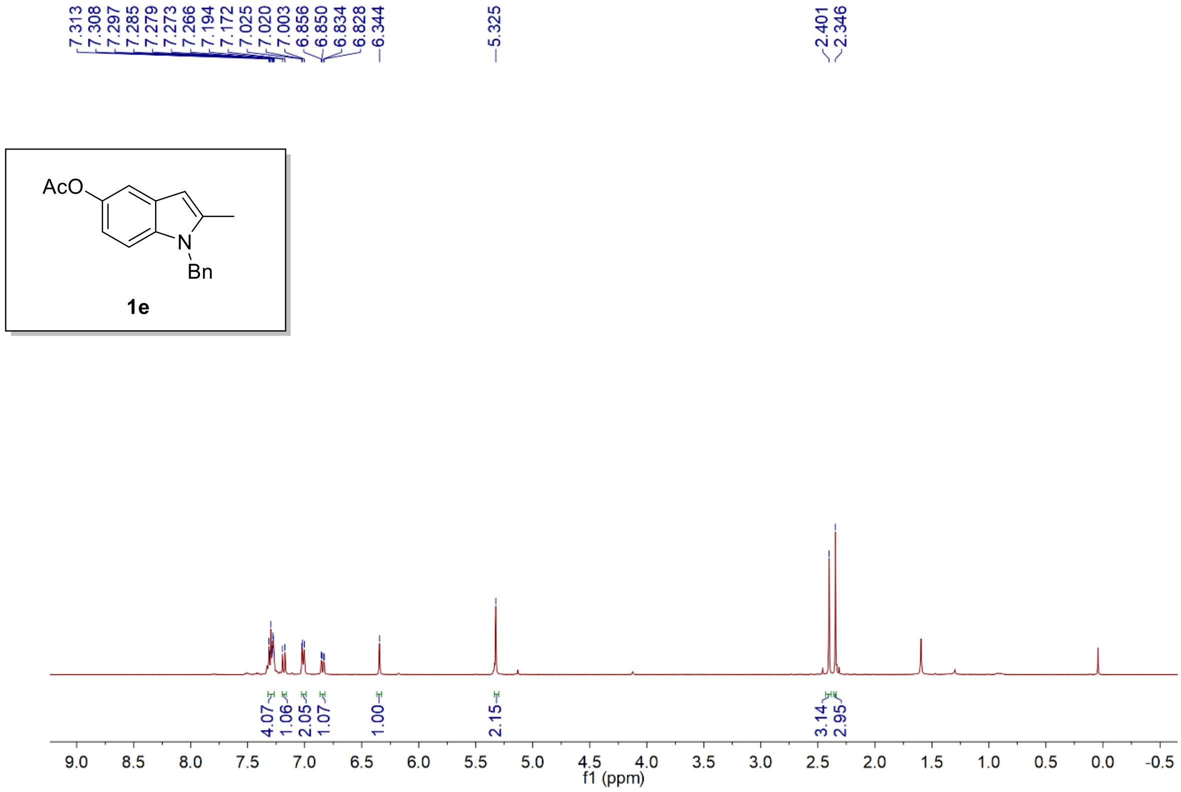

^{13}C-NMR spectrum of compound **1e** (101 MHz, $CDCl_3$)

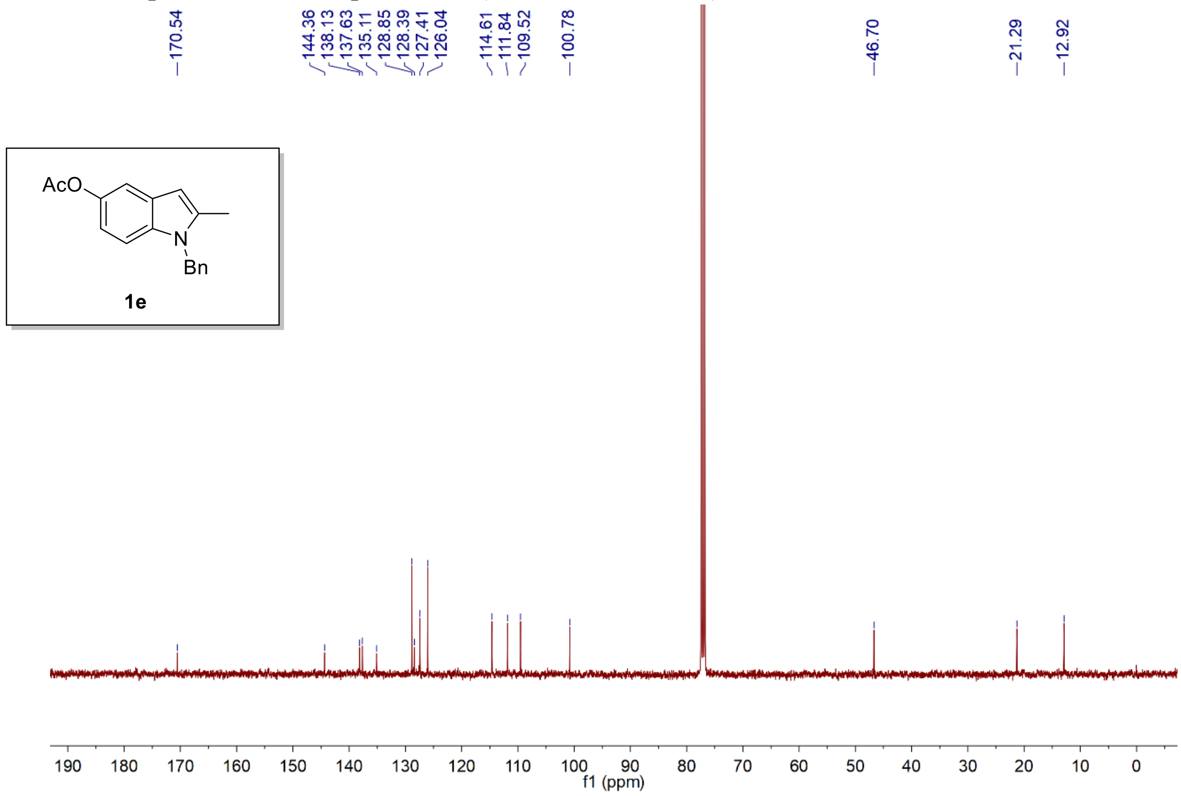

^{1}H-NMR spectrum of compound **1f** (400 MHz, $CDCl_3$)

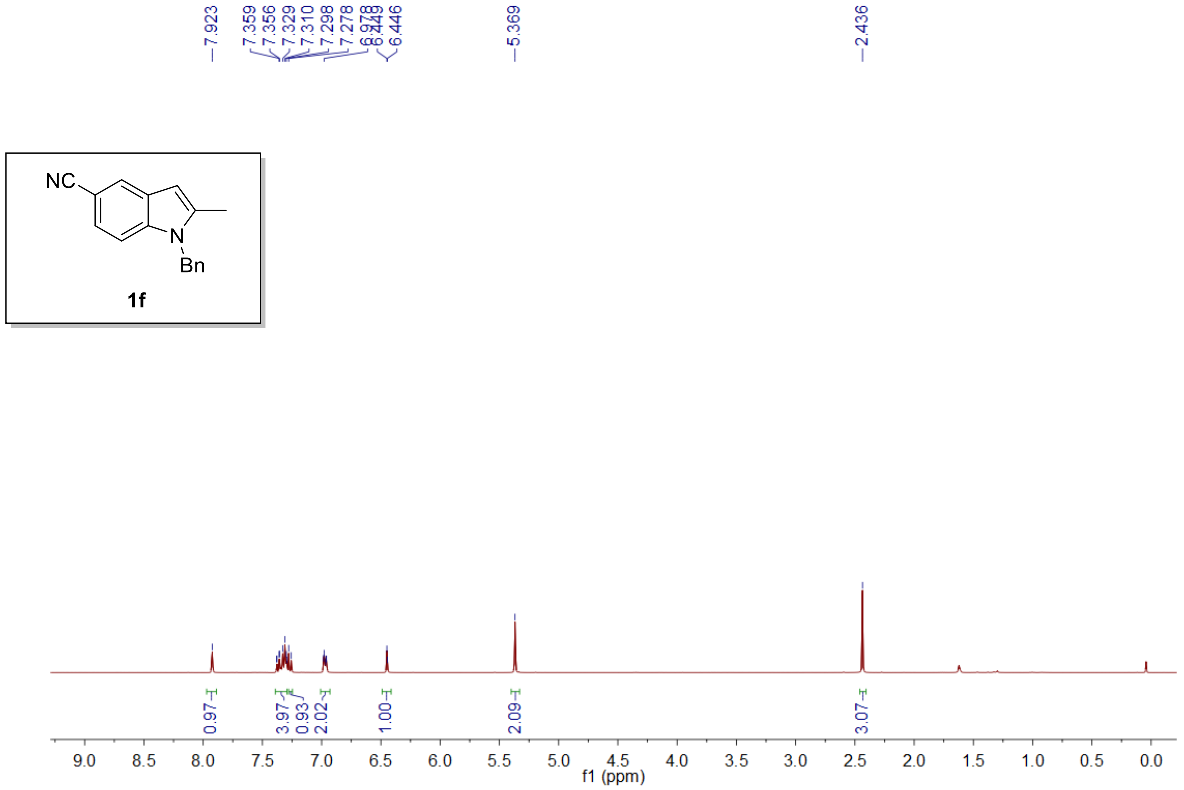

^{13}C-NMR spectrum of compound **1f** (101 MHz, $CDCl_3$)

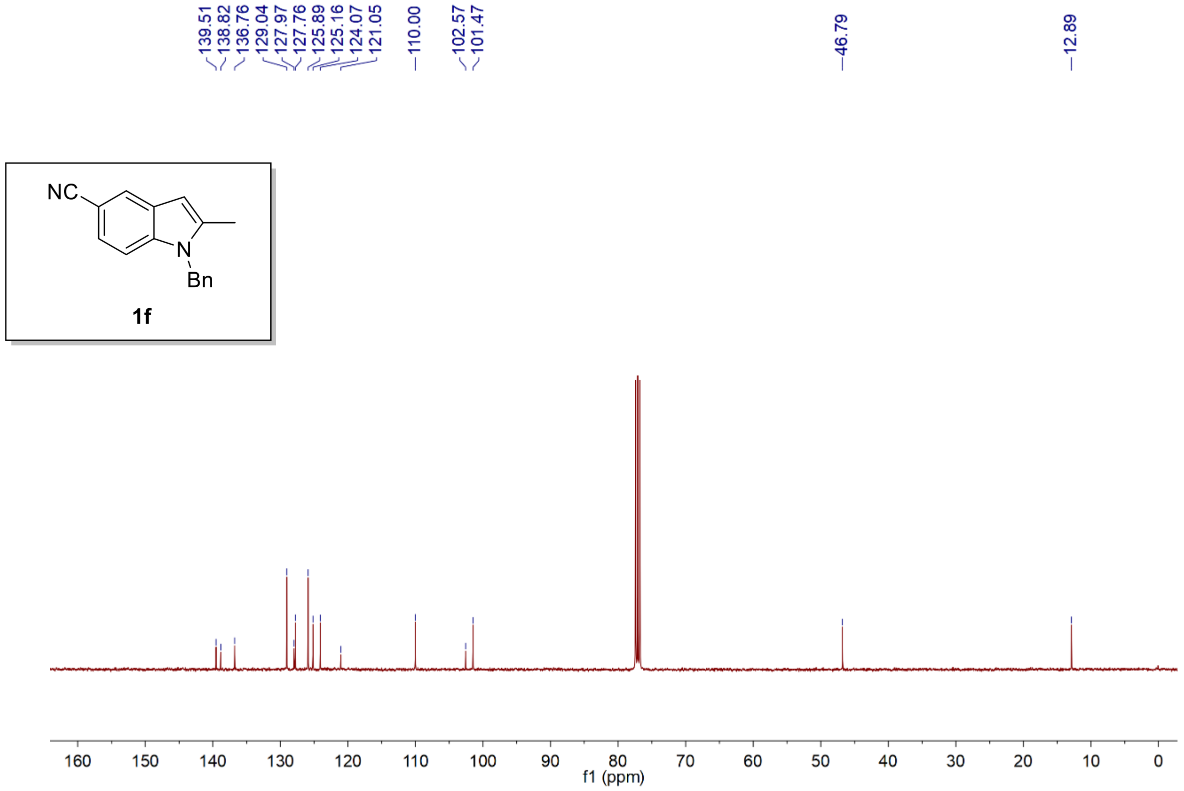

^{1}H-NMR spectrum of compound **1h** (400 MHz, $CDCl_3$)

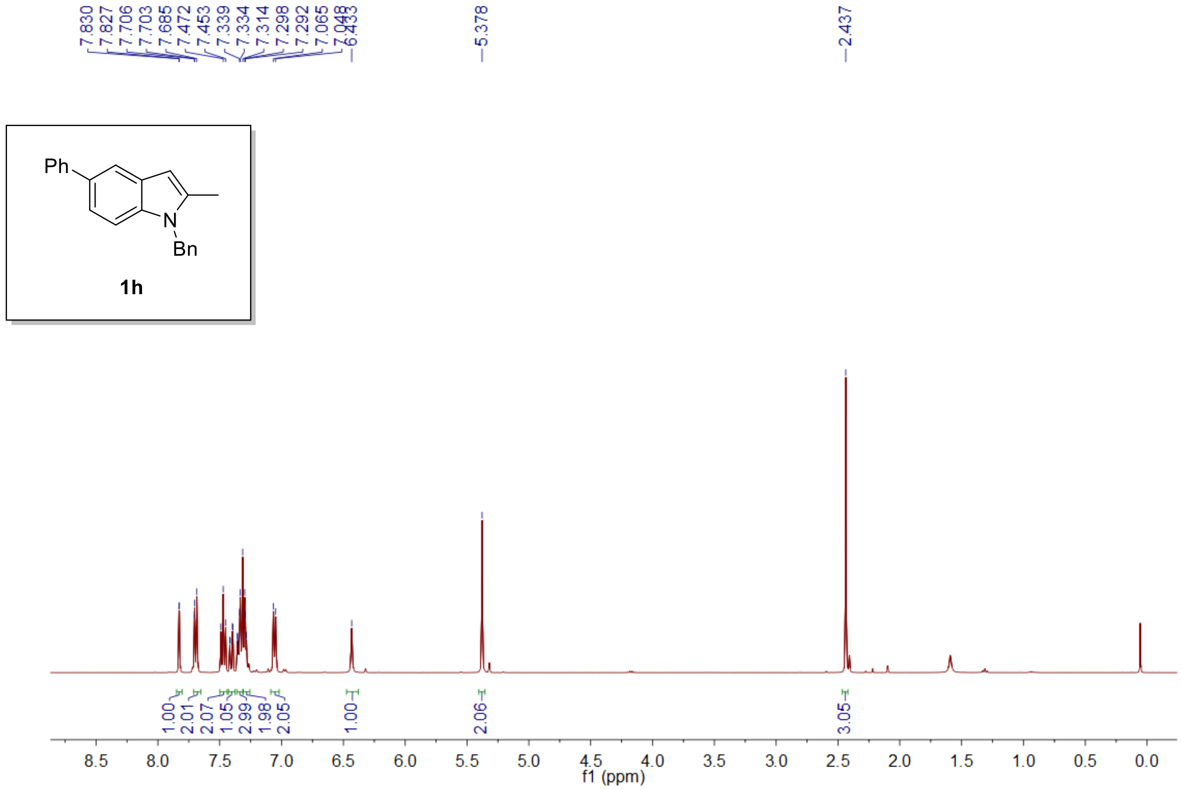

^{13}C-NMR spectrum of compound **1h** (101 MHz, $CDCl_3$)

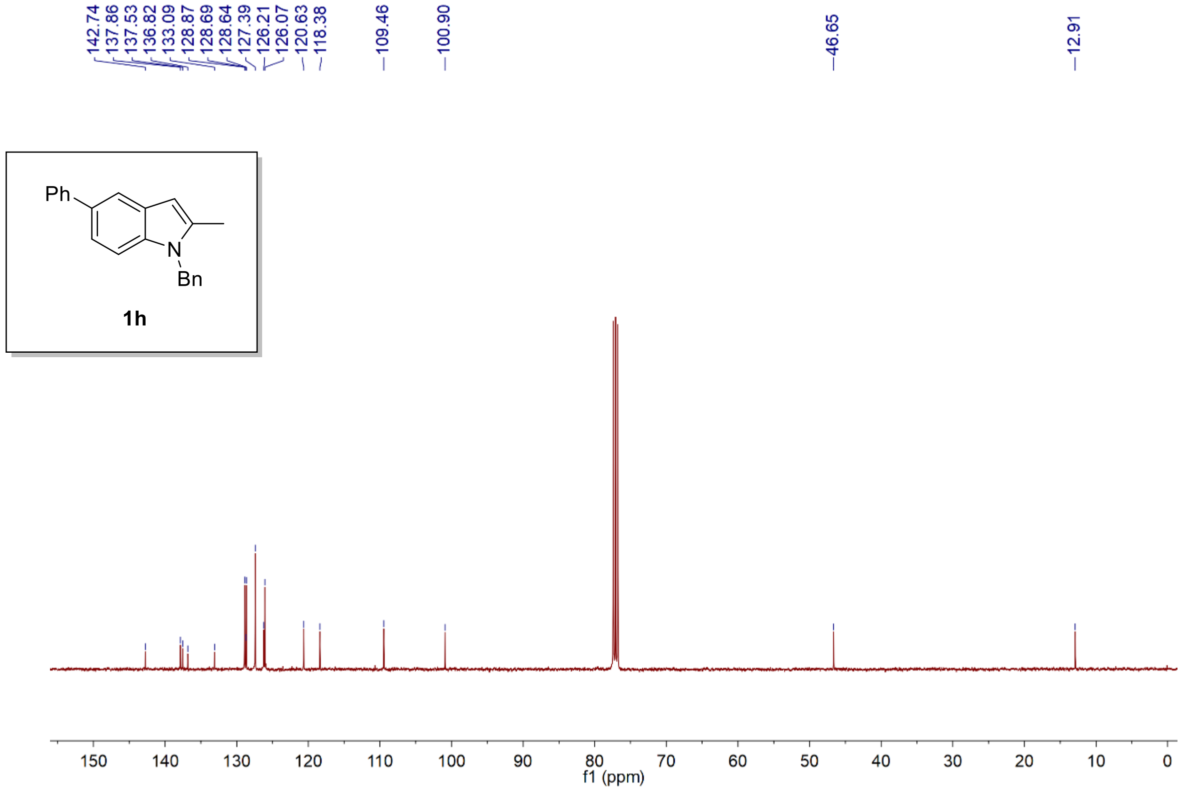

^{1}H-NMR spectrum of compound **1i** (400 MHz, $CDCl_3$)

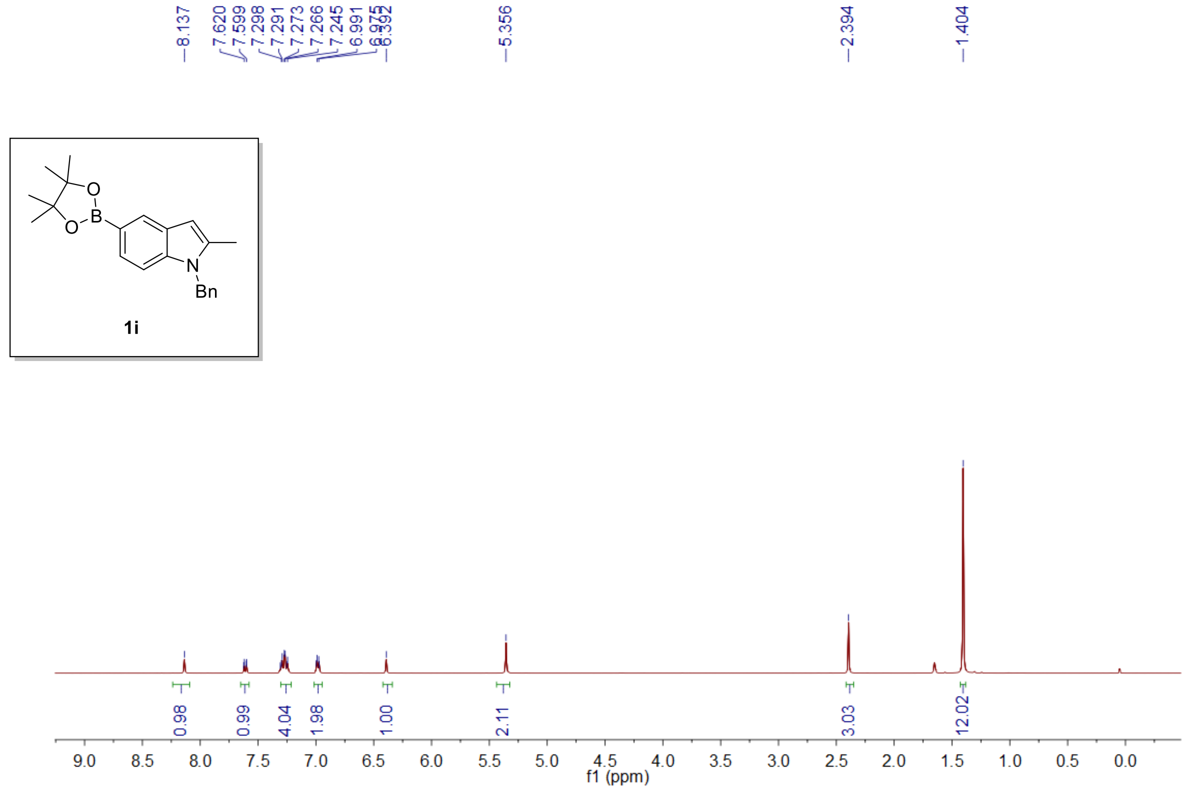

^{13}C-NMR spectrum of compound **1i** (101 MHz, $CDCl_3$)

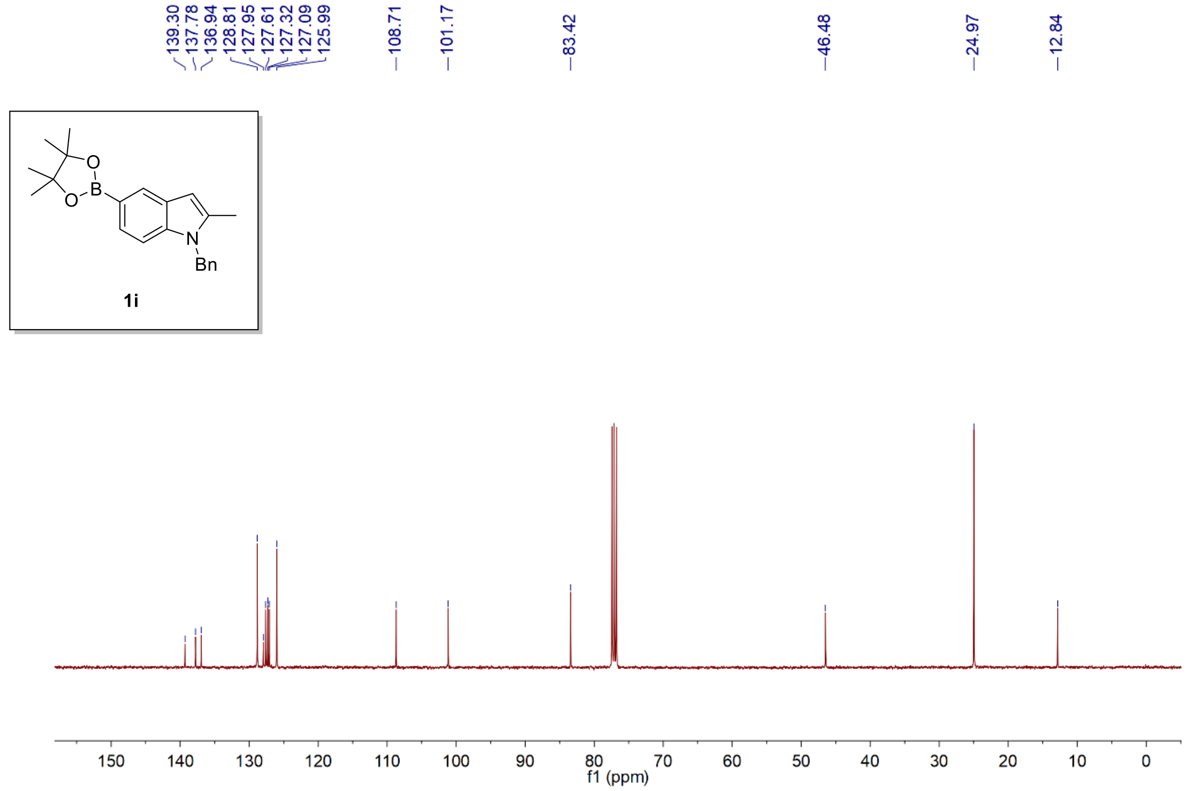

^{1}H-NMR spectrum of compound **1j** (400 MHz, $CDCl_3$)

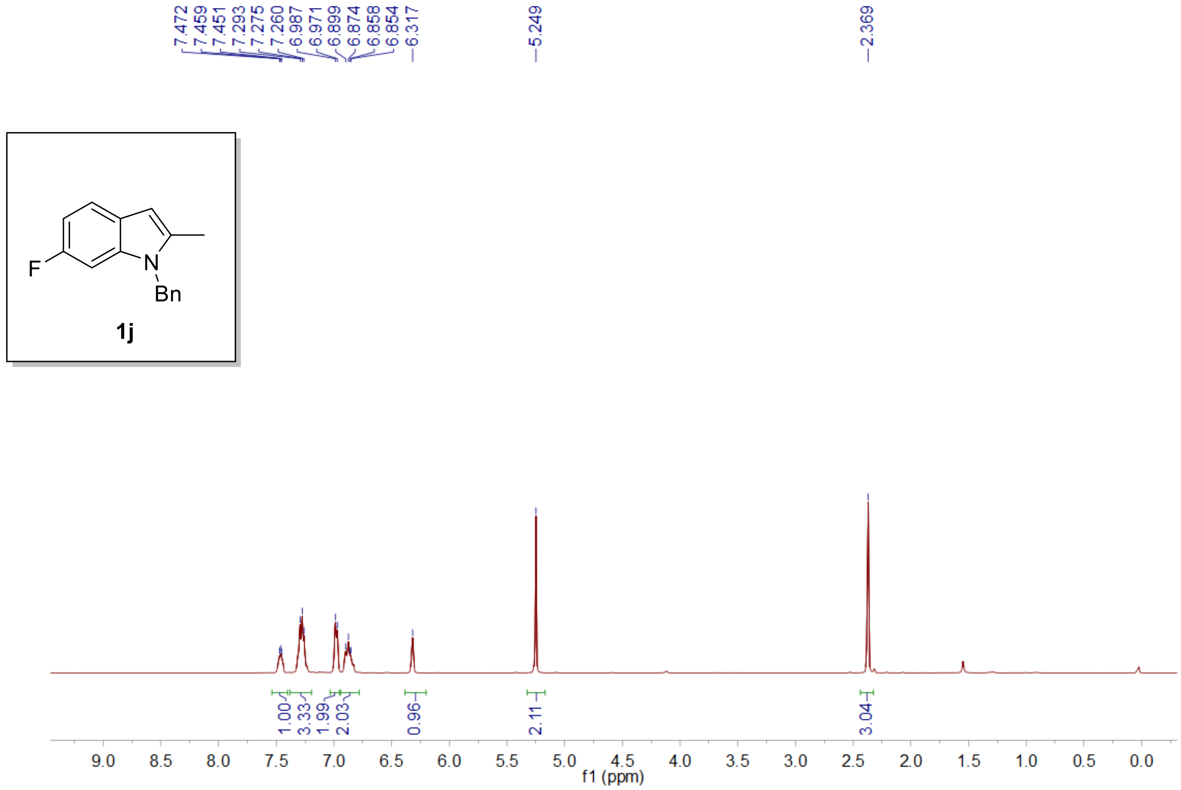

^{13}C-NMR spectrum of compound **1j** (101 MHz, $CDCl_3$)

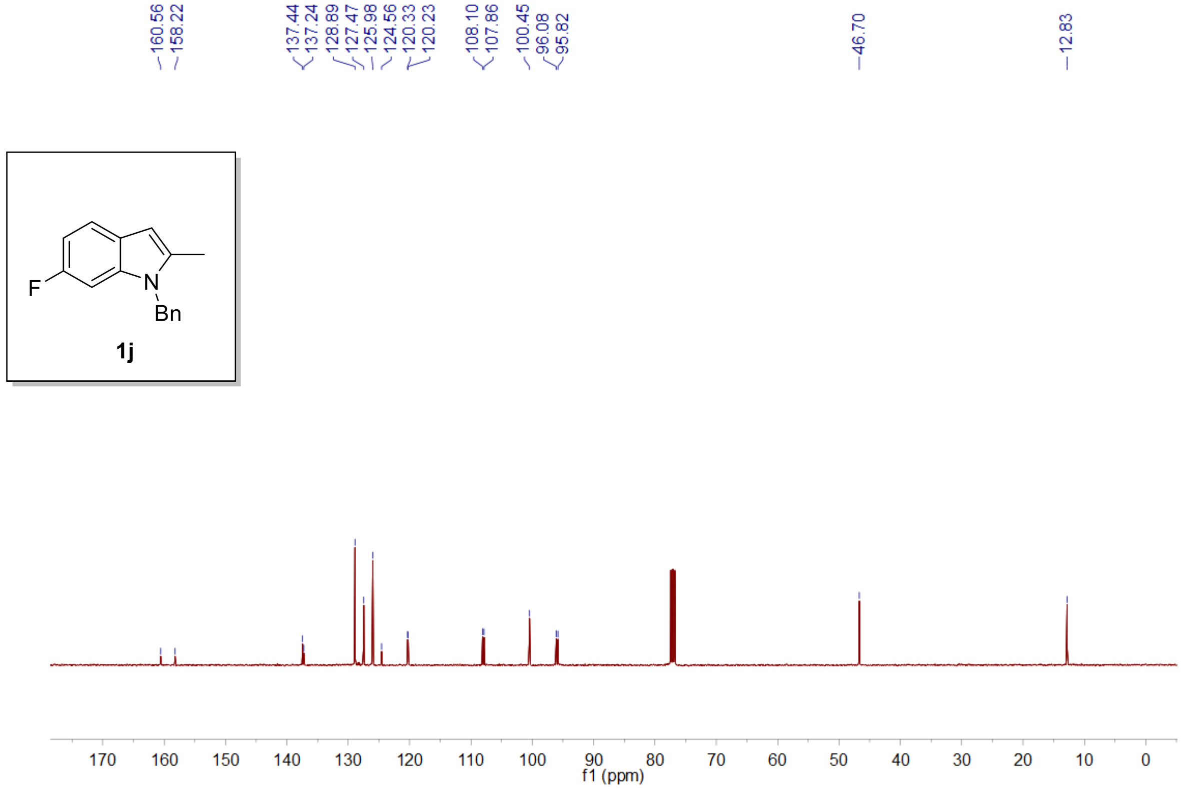

^{19}F-NMR spectrum of compound **1j** (565 MHz, $CDCl_3$)

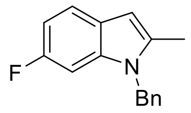

1j

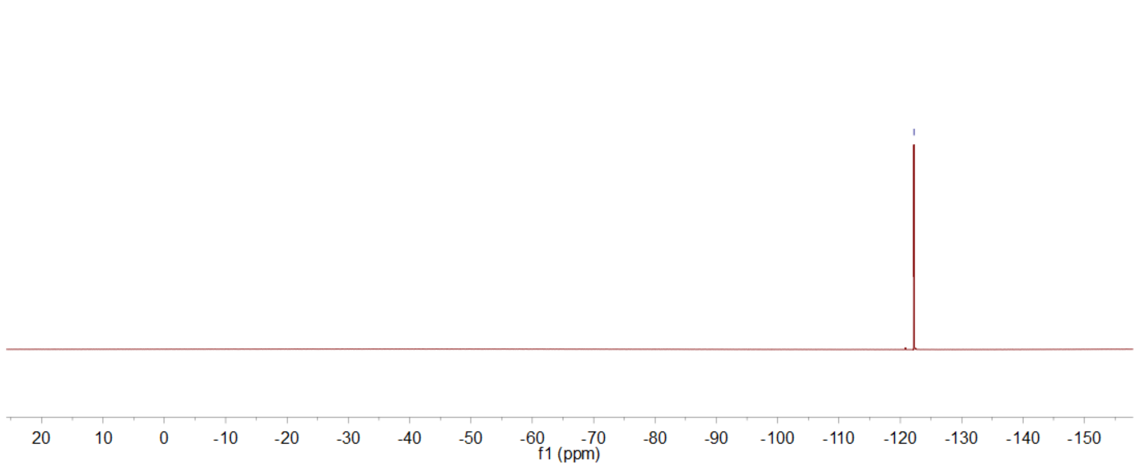

^{1}H-NMR spectrum of compound **1k** (400 MHz, $CDCl_3$)

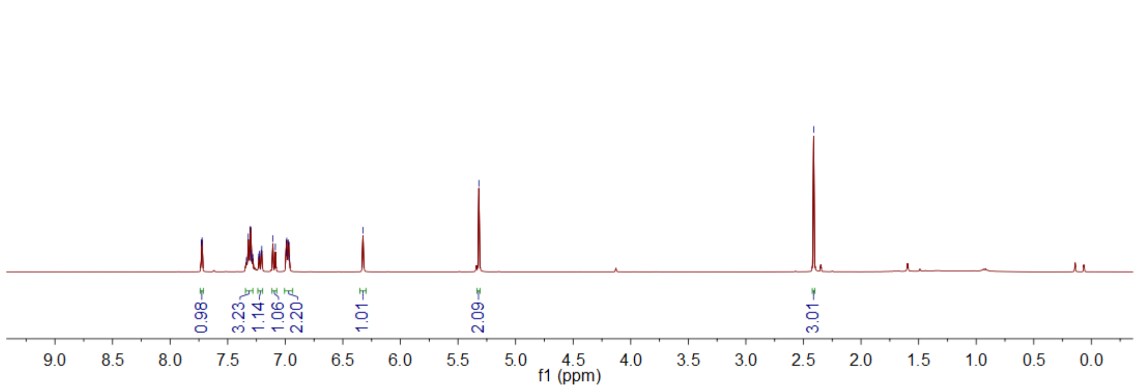

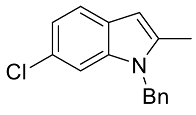

1k

^{13}C-NMR spectrum of compound **1k** (101 MHz, $CDCl_3$)

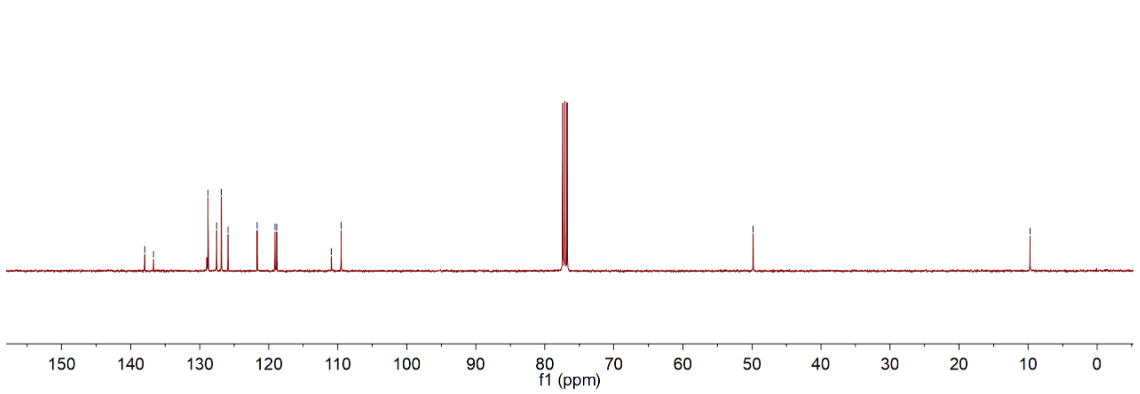

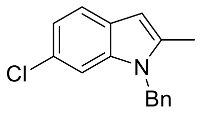

1k

^{1}H-NMR spectrum of compound **1l** (400 MHz, $CDCl_3$)

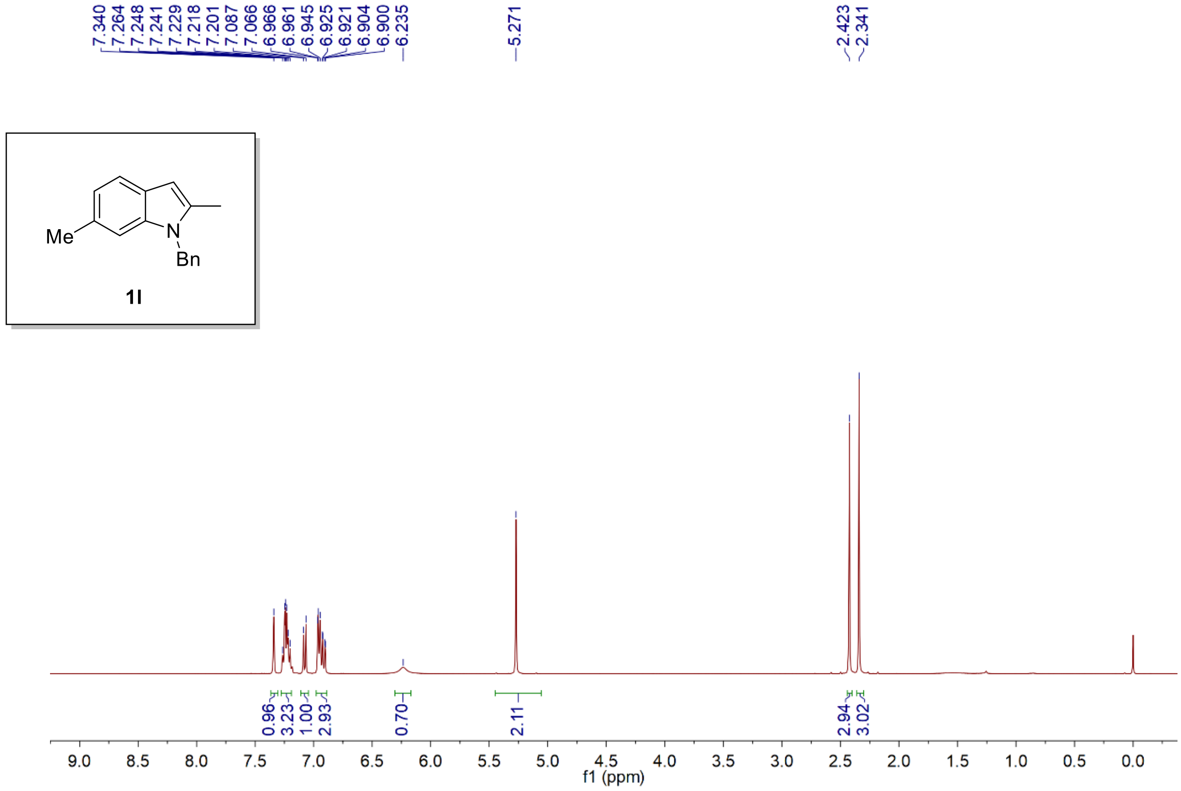

^{13}C-NMR spectrum of compound **1l** (101 MHz, $CDCl_3$)

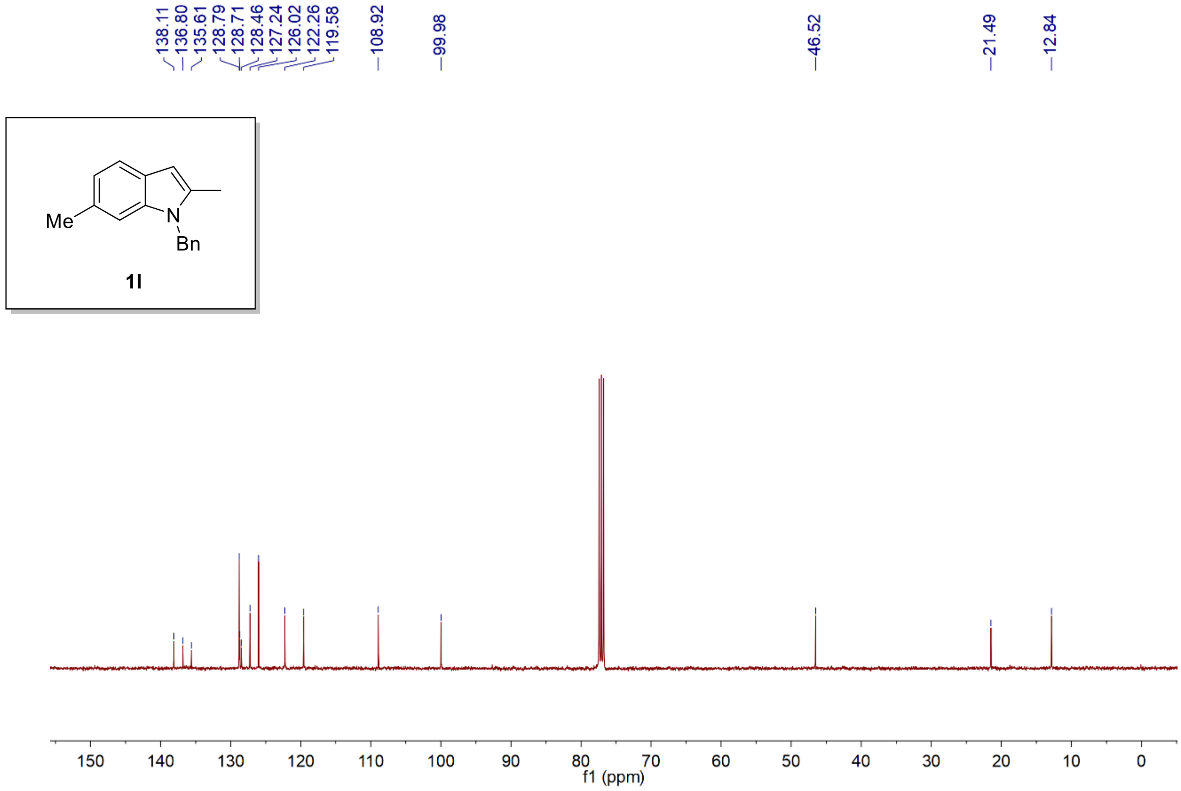

^{1}H-NMR spectrum of compound **1p** (400 MHz, $CDCl_3$)

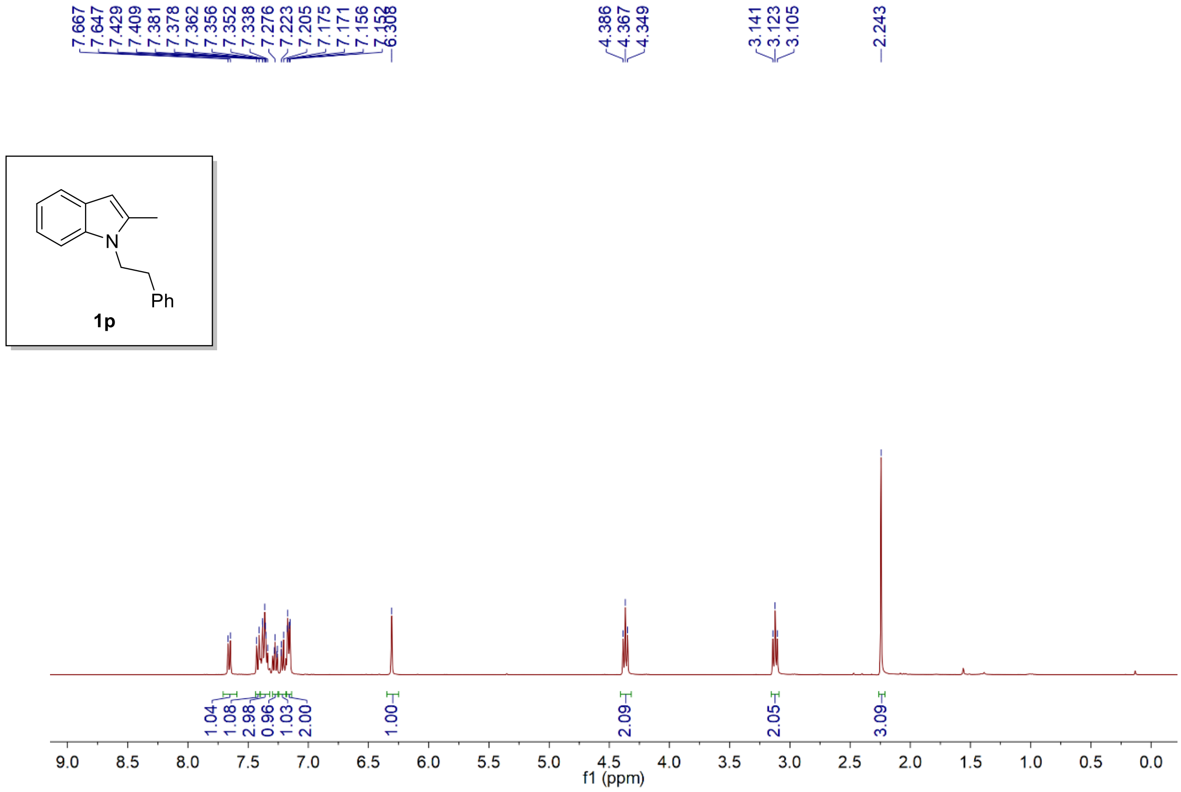

^{13}C-NMR spectrum of compound **1p** (101 MHz, $CDCl_3$)

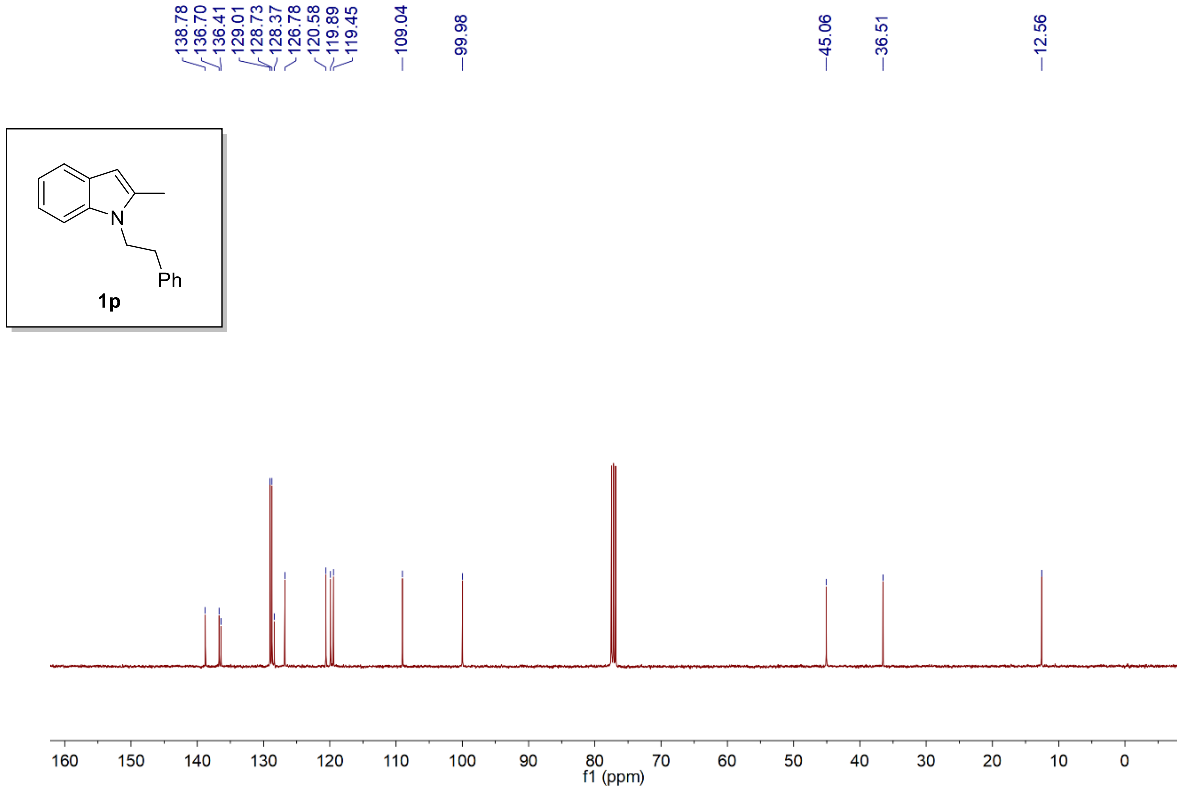

^{1}H-NMR spectrum of compound **1r** (400 MHz, $CDCl_3$)

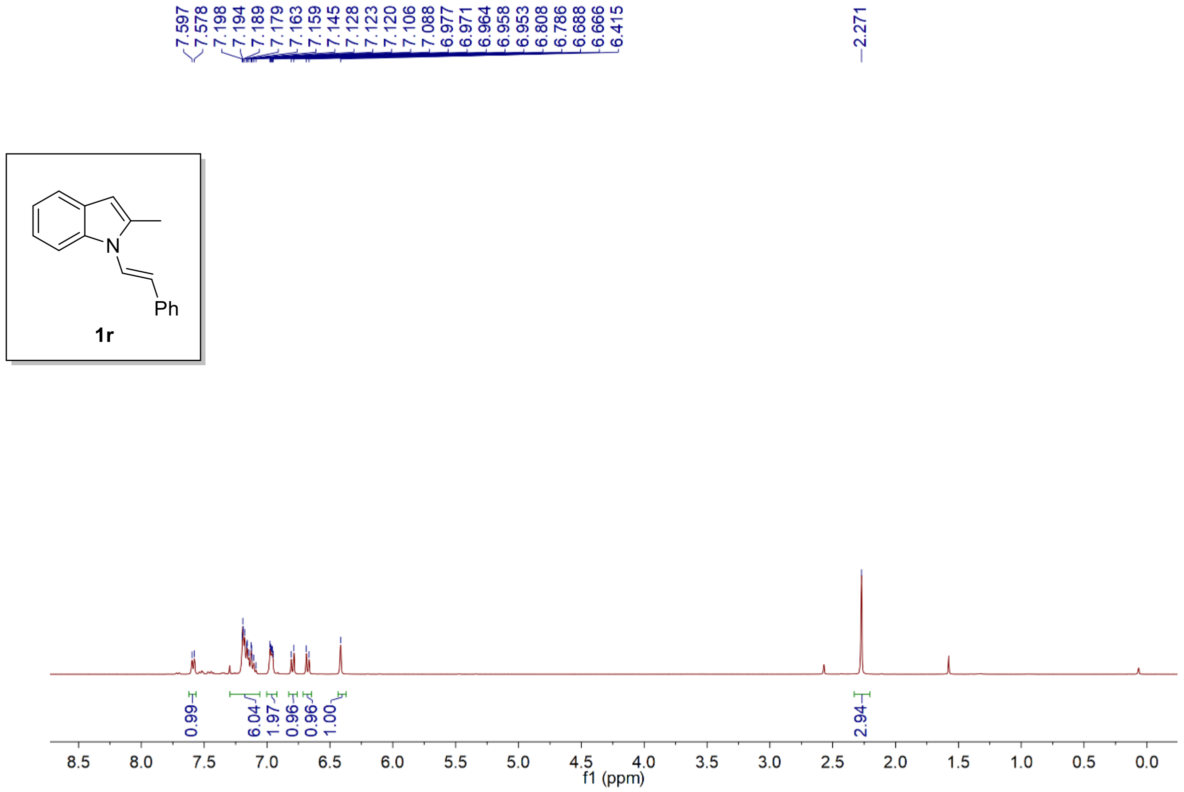

^{13}C-NMR spectrum of compound **1r** (101 MHz, $CDCl_3$)

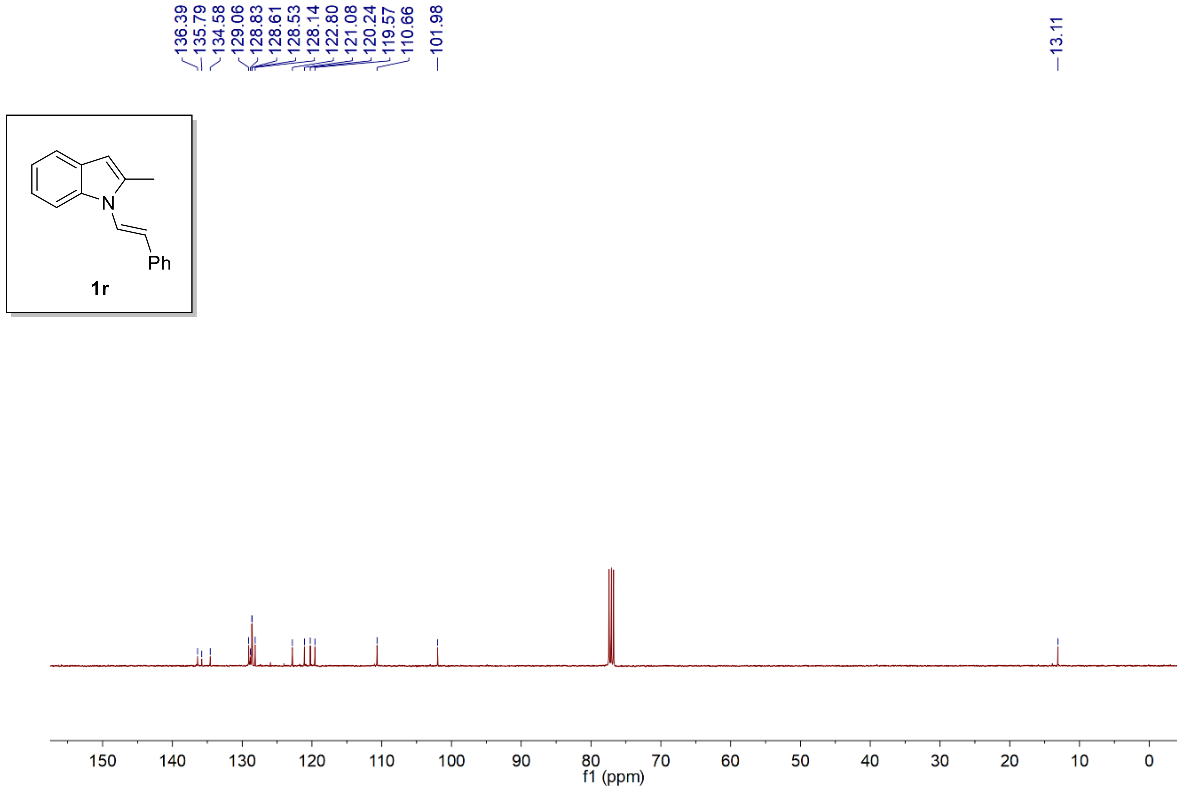

^{1}H-NMR spectrum of compound **1t** (400 MHz, $CDCl_3$)

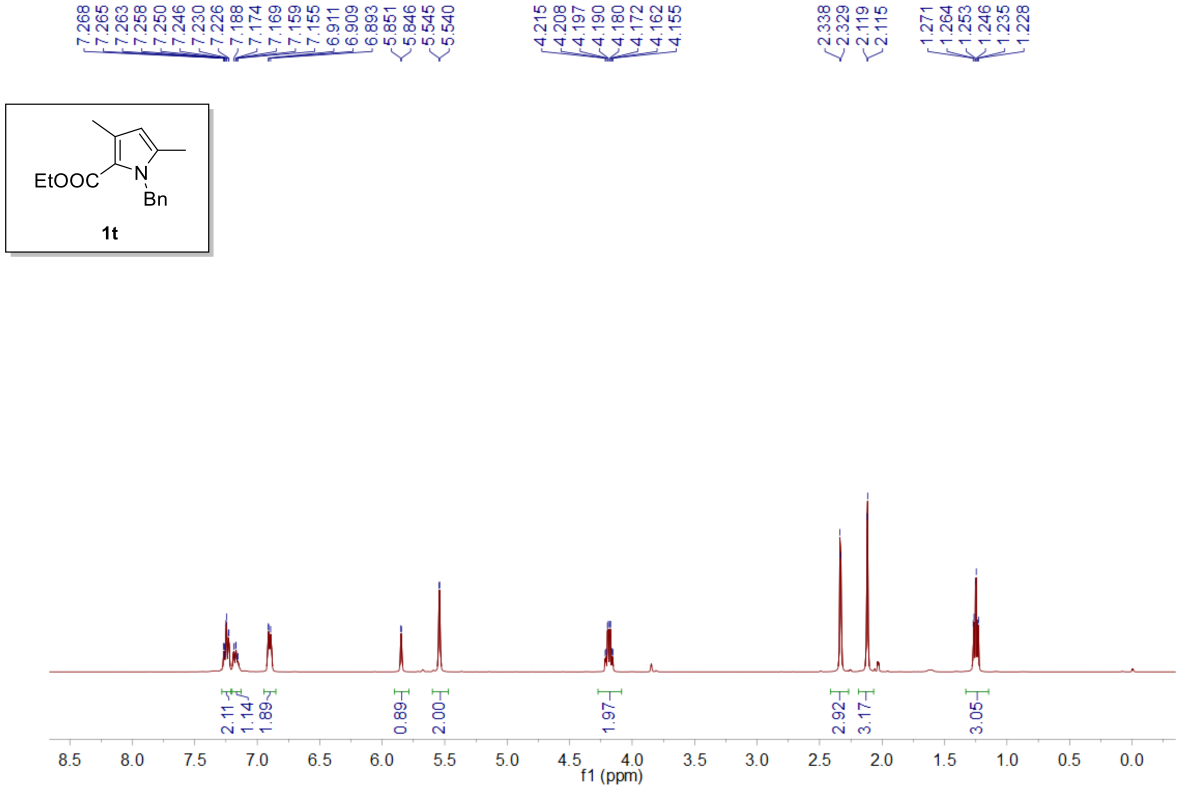

^{13}C-NMR spectrum of compound **1t** (101 MHz, $CDCl_3$)

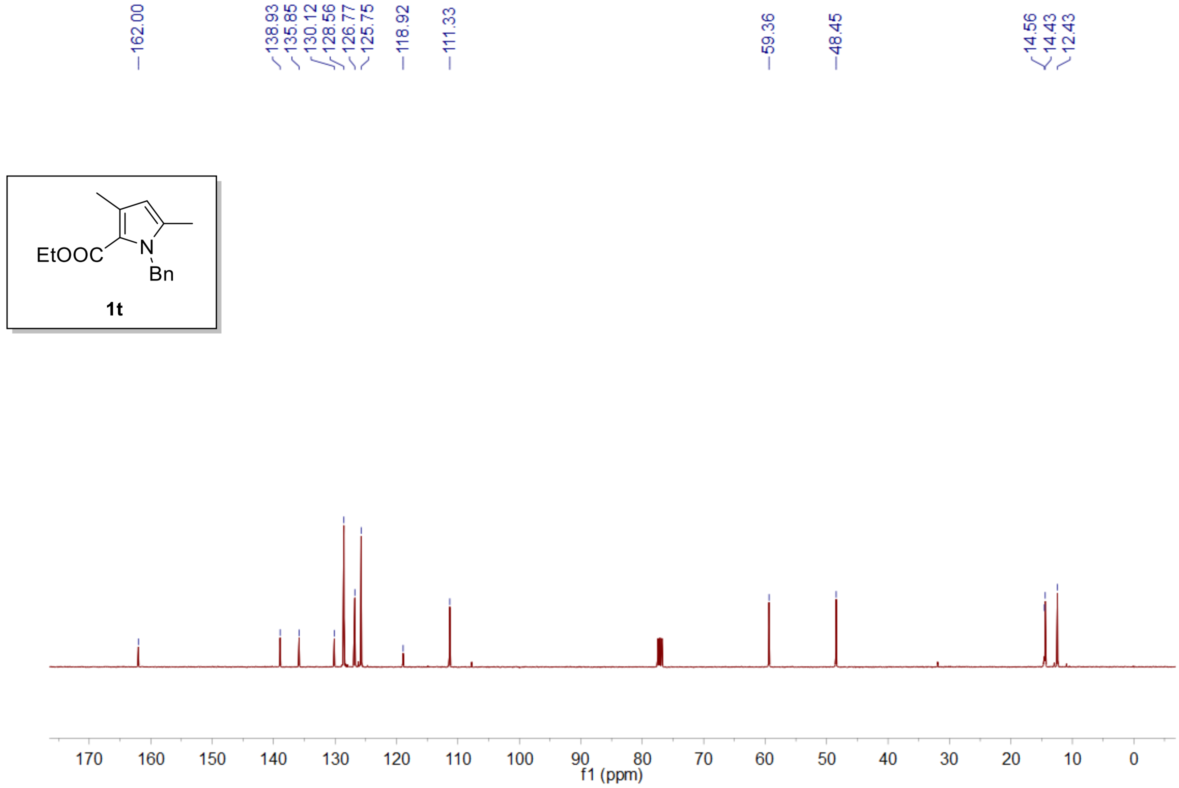

^{1}H-NMR spectrum of compound **1u** (400 MHz, $CDCl_3$)

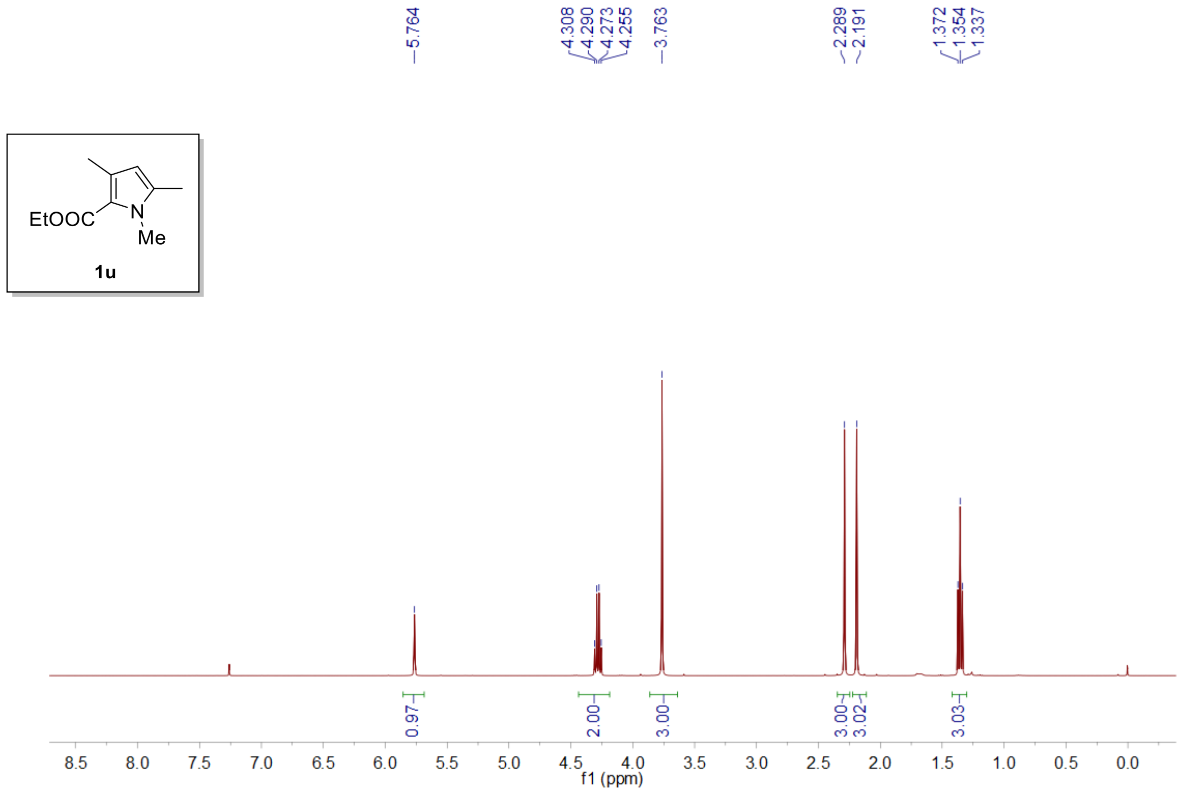

^{13}C-NMR spectrum of compound **1u** (101 MHz, $CDCl_3$)

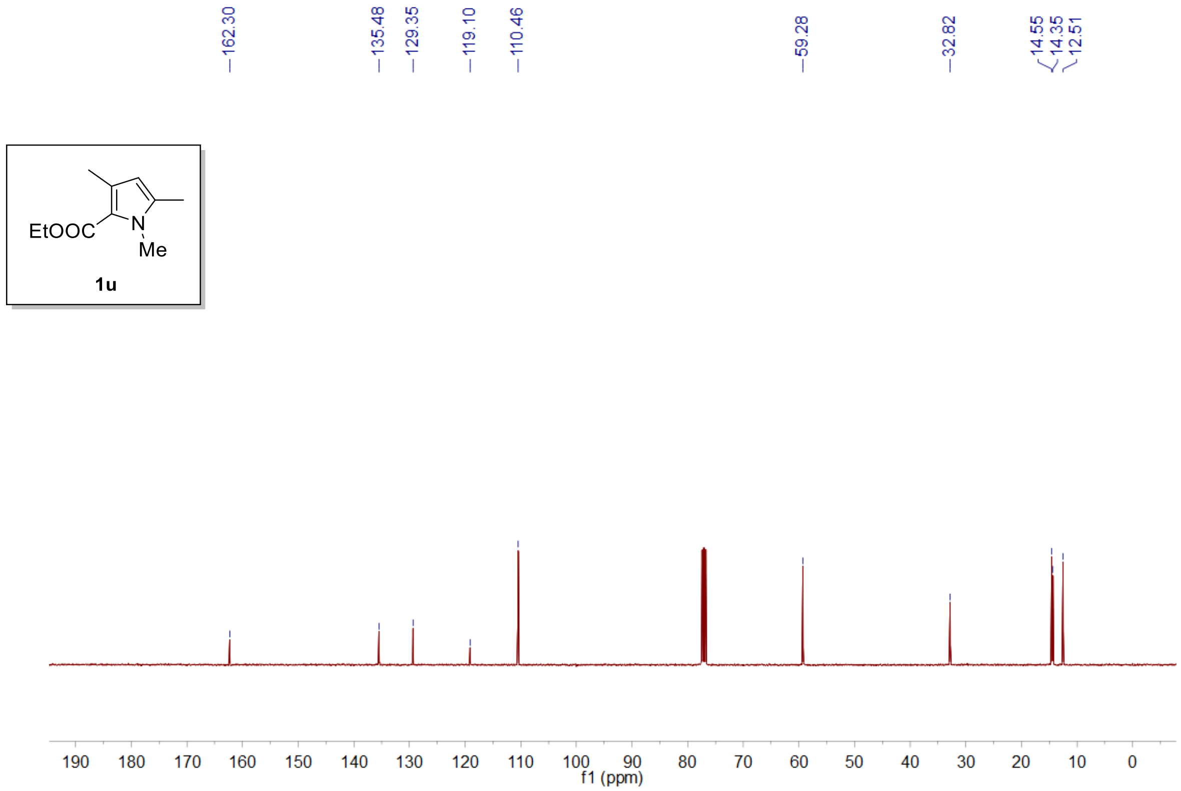

^{1}H-NMR spectrum of compound **5a** (400 MHz, $CDCl_3$)

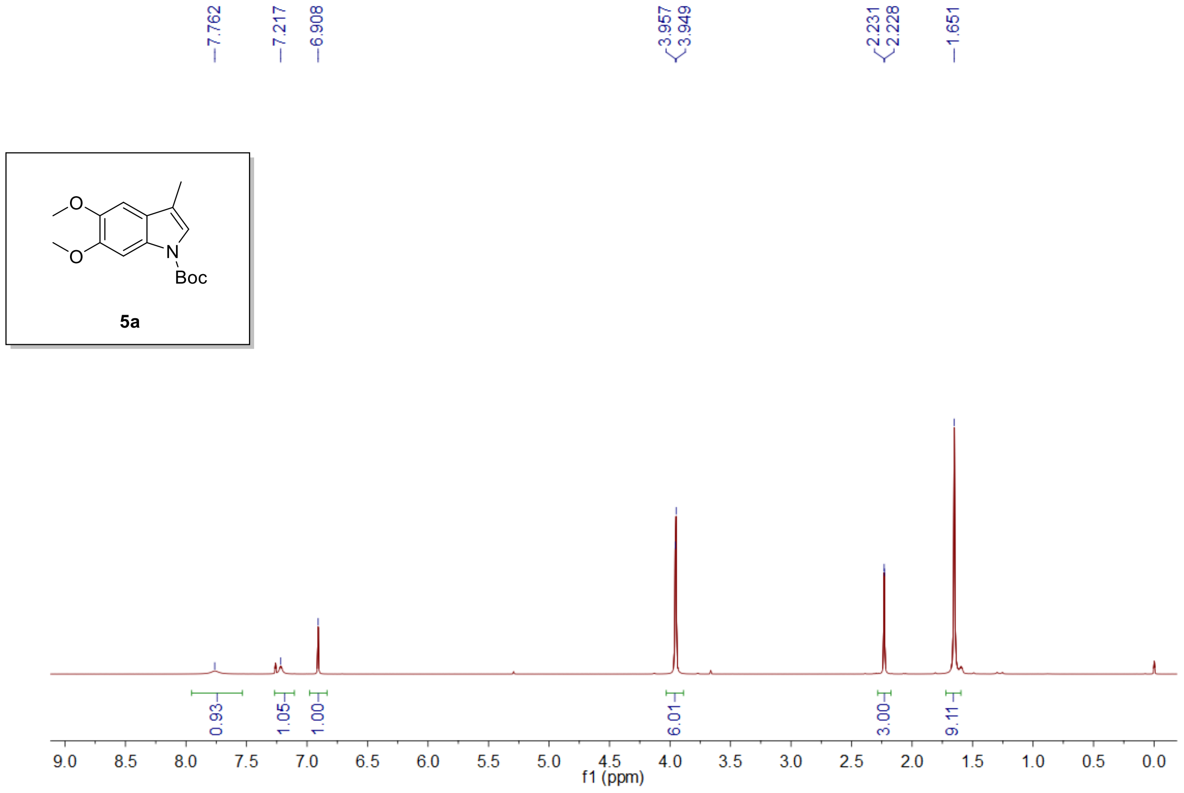

^{13}C-NMR spectrum of compound **5a** (101 MHz, $CDCl_3$)

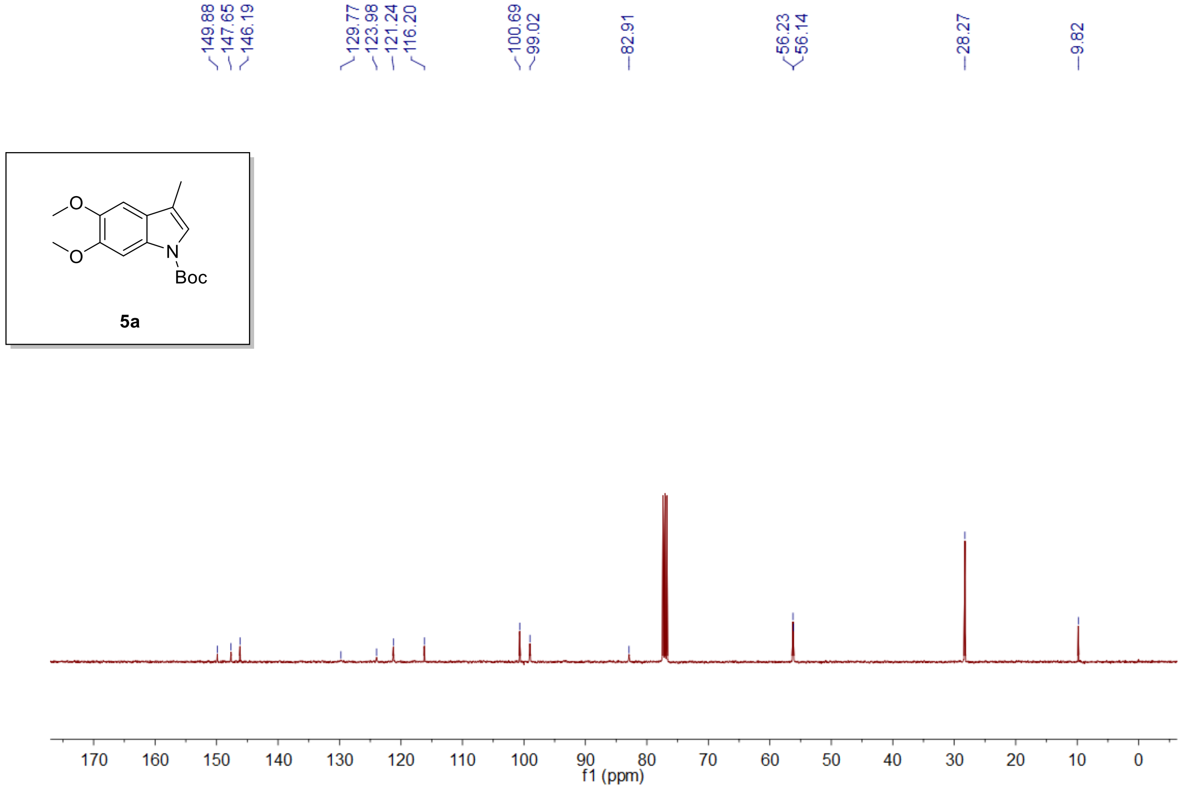

^{1}H-NMR spectrum of compound **5b** (400 MHz, $CDCl_3$)

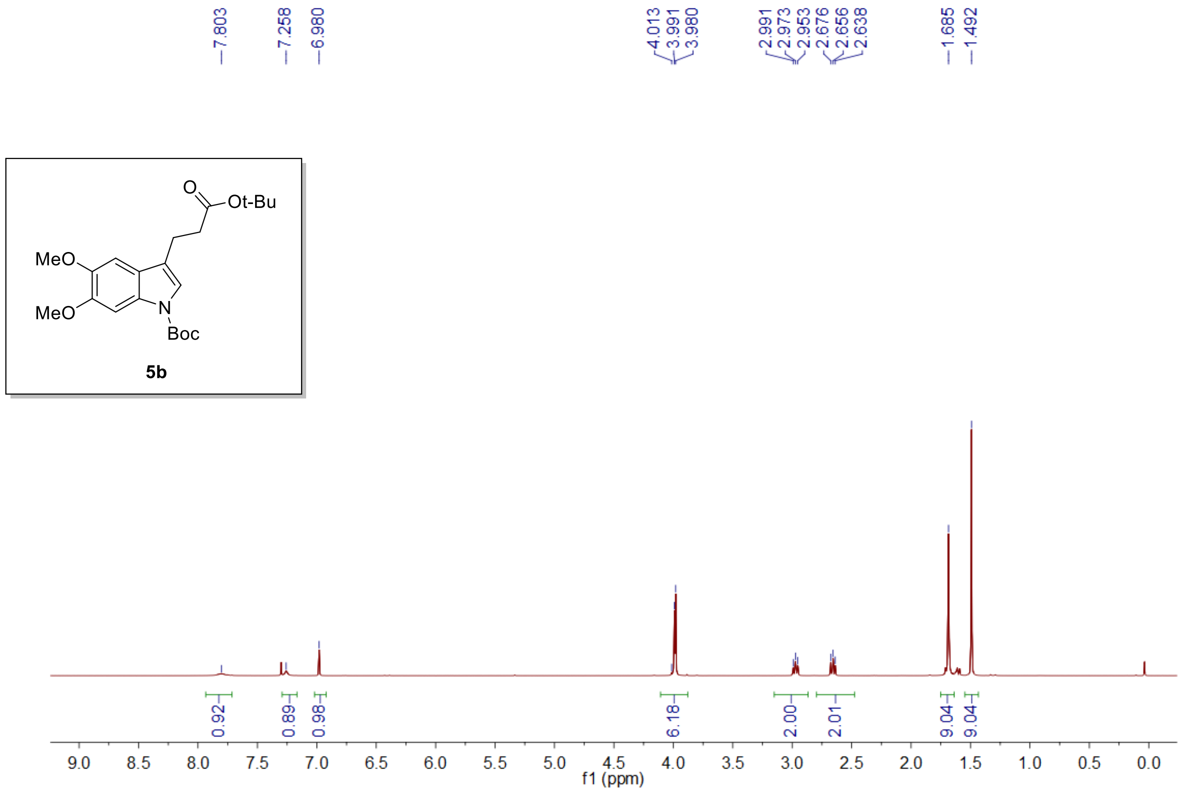

^{13}C-NMR spectrum of compound **5b** (101 MHz, $CDCl_3$)

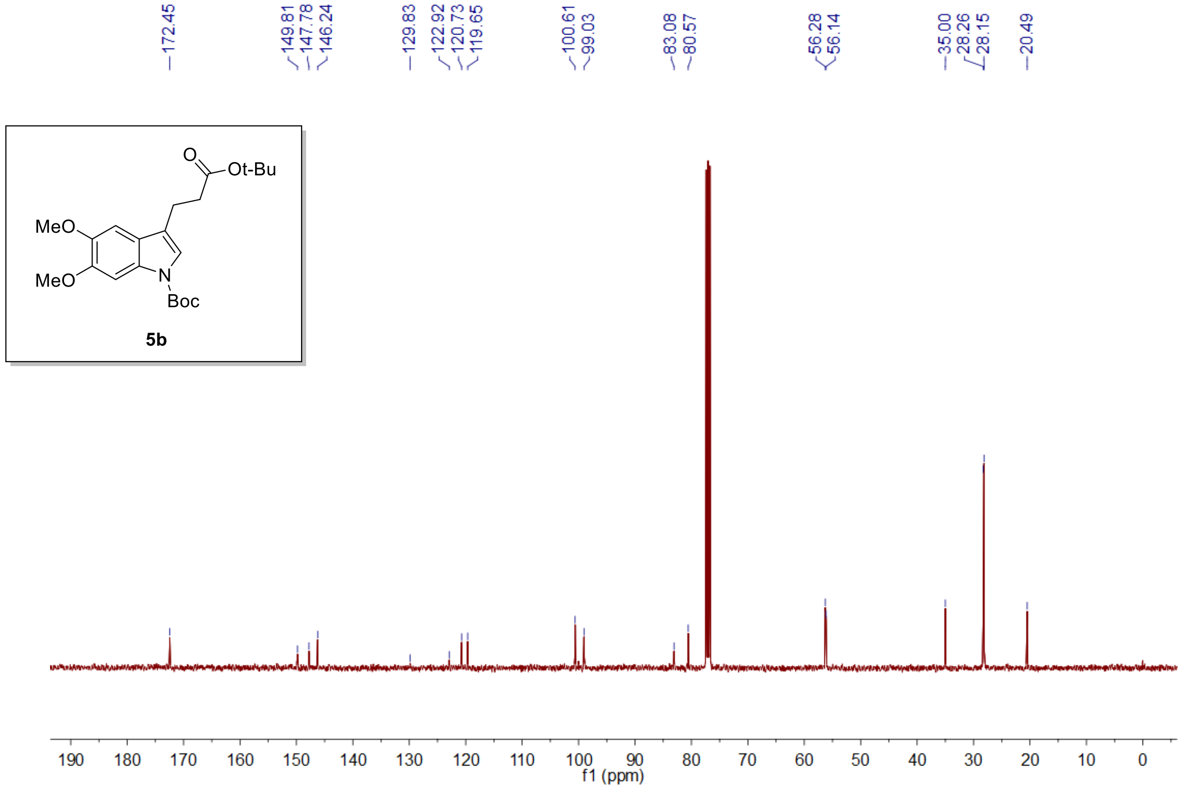

^{1}H-NMR spectrum of compound **5c** (400 MHz, $CDCl_3$)

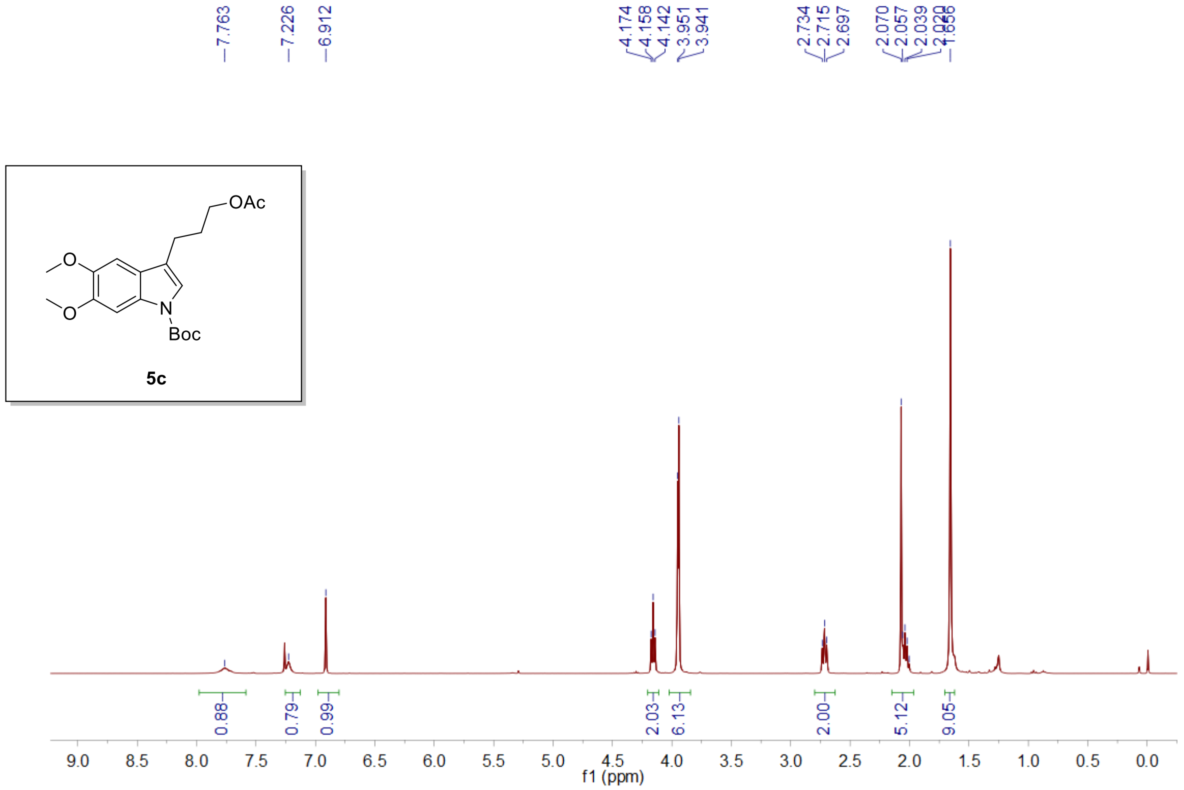

^{13}C-NMR spectrum of compound **5c** (101 MHz, $CDCl_3$)

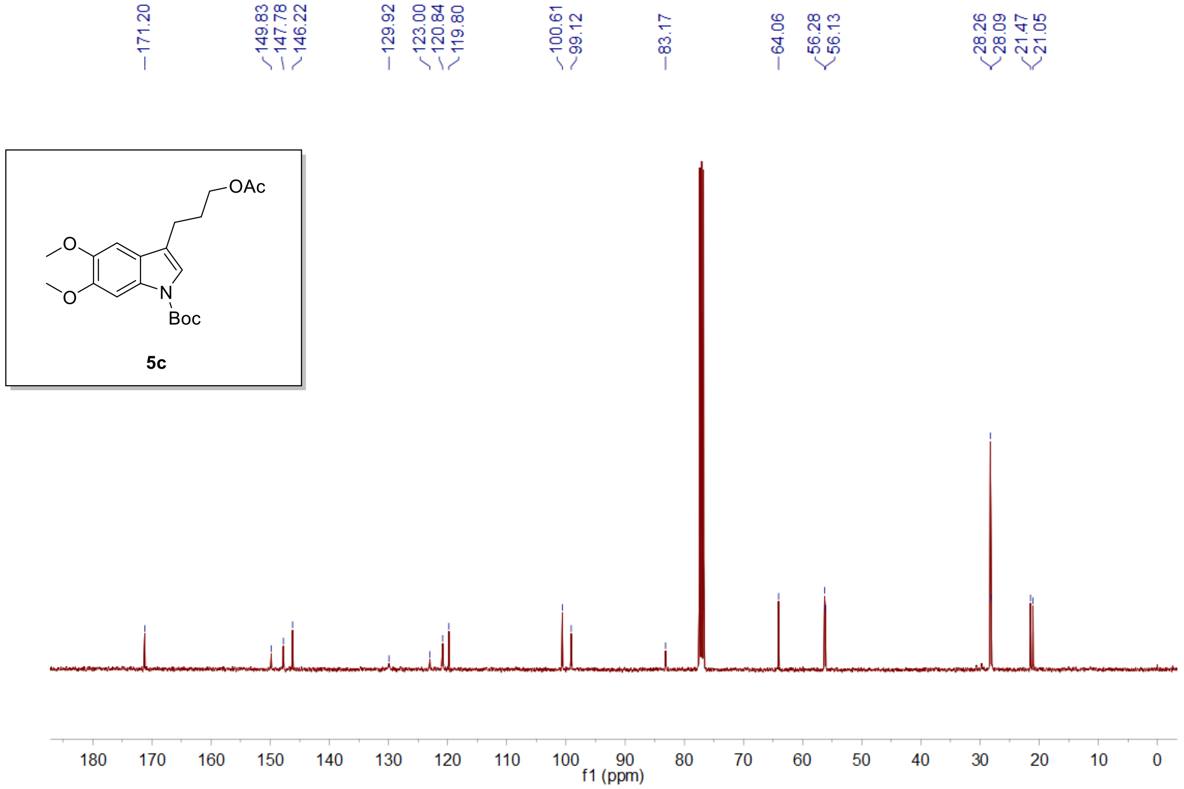

^{1}H-NMR spectrum of compound **5d** (400 MHz, $CDCl_3$)

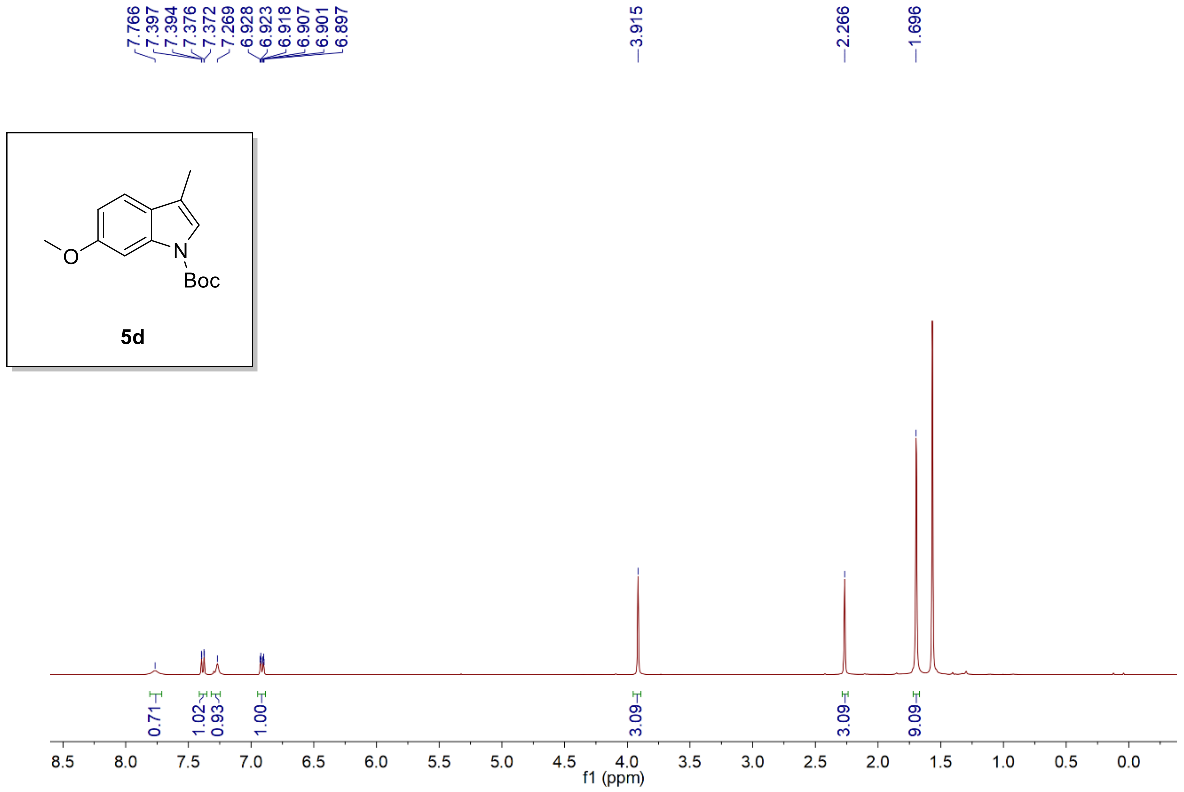

^{13}C-NMR spectrum of compound **5d** (101 MHz, $CDCl_3$)

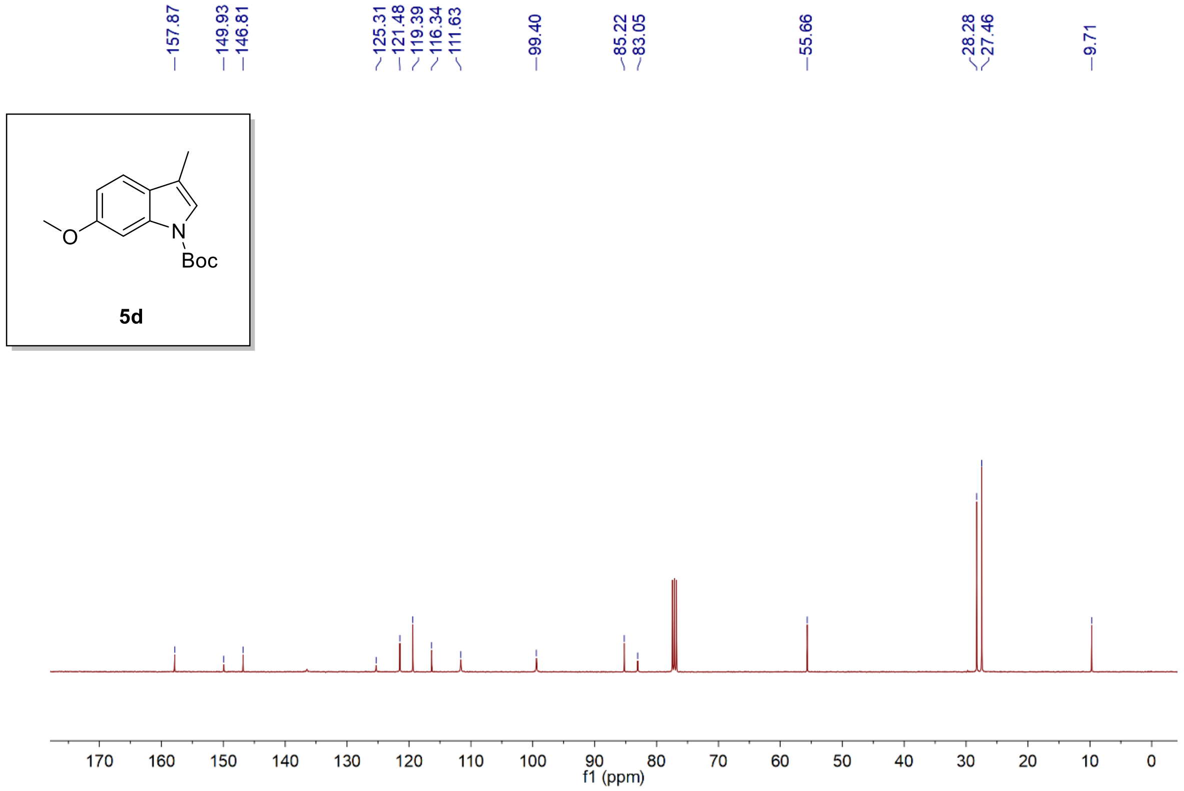

^{1}H-NMR spectrum of compound **5e** (400 MHz, $CDCl_3$)

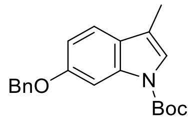

5e

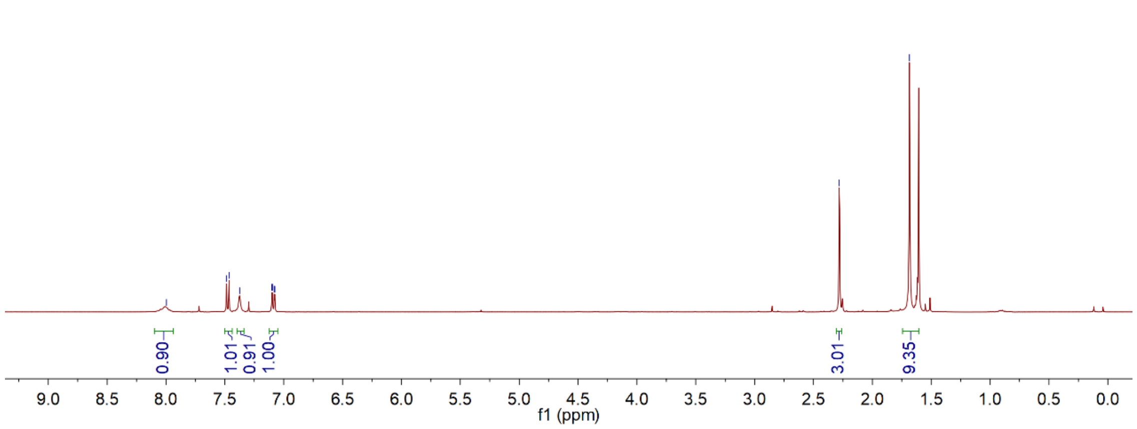

^{13}C-NMR spectrum of compound **5e** (101 MHz, $CDCl_3$)

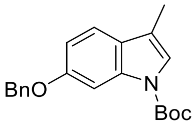

5e

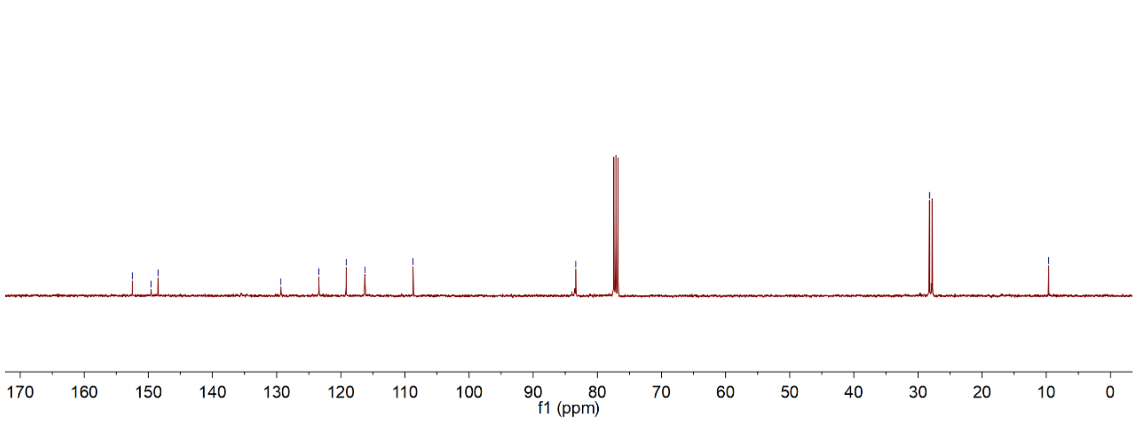

^{1}H-NMR spectrum of compound **5f** (400 MHz, $CDCl_3$)

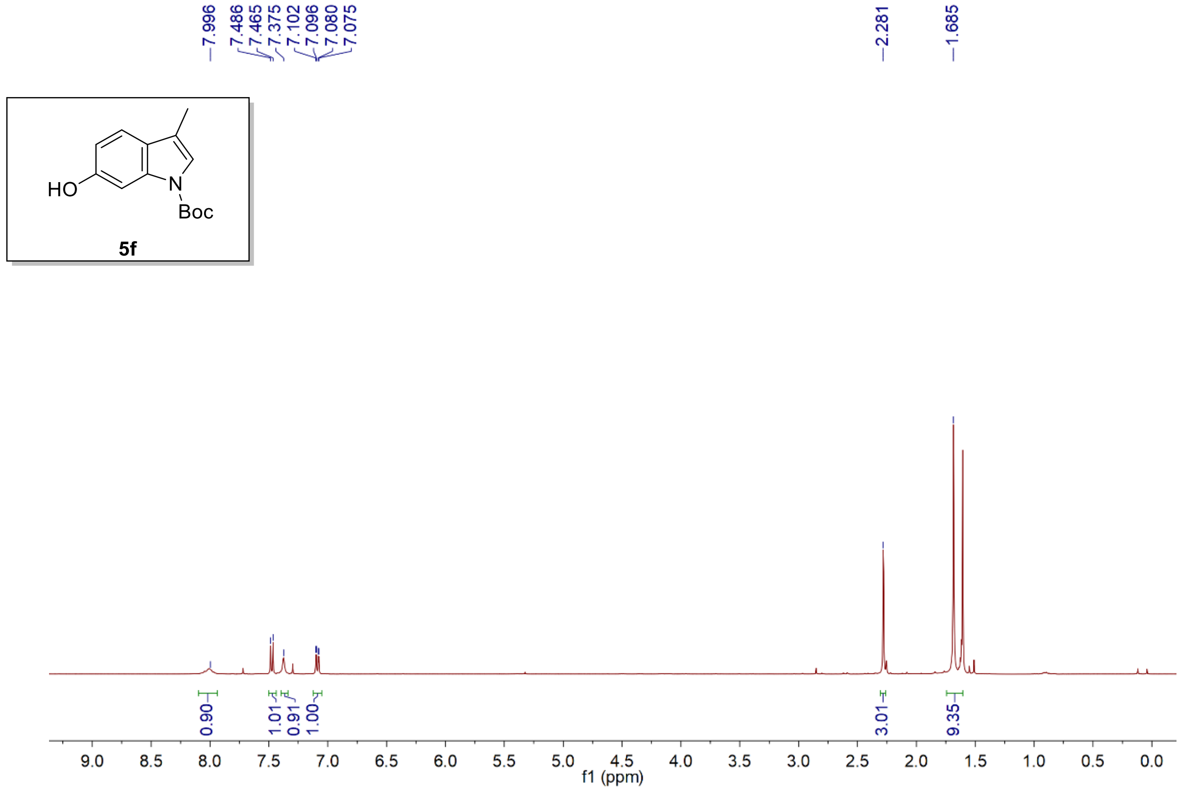

^{13}C-NMR spectrum of compound **6af** (101 MHz, $CDCl_3$)

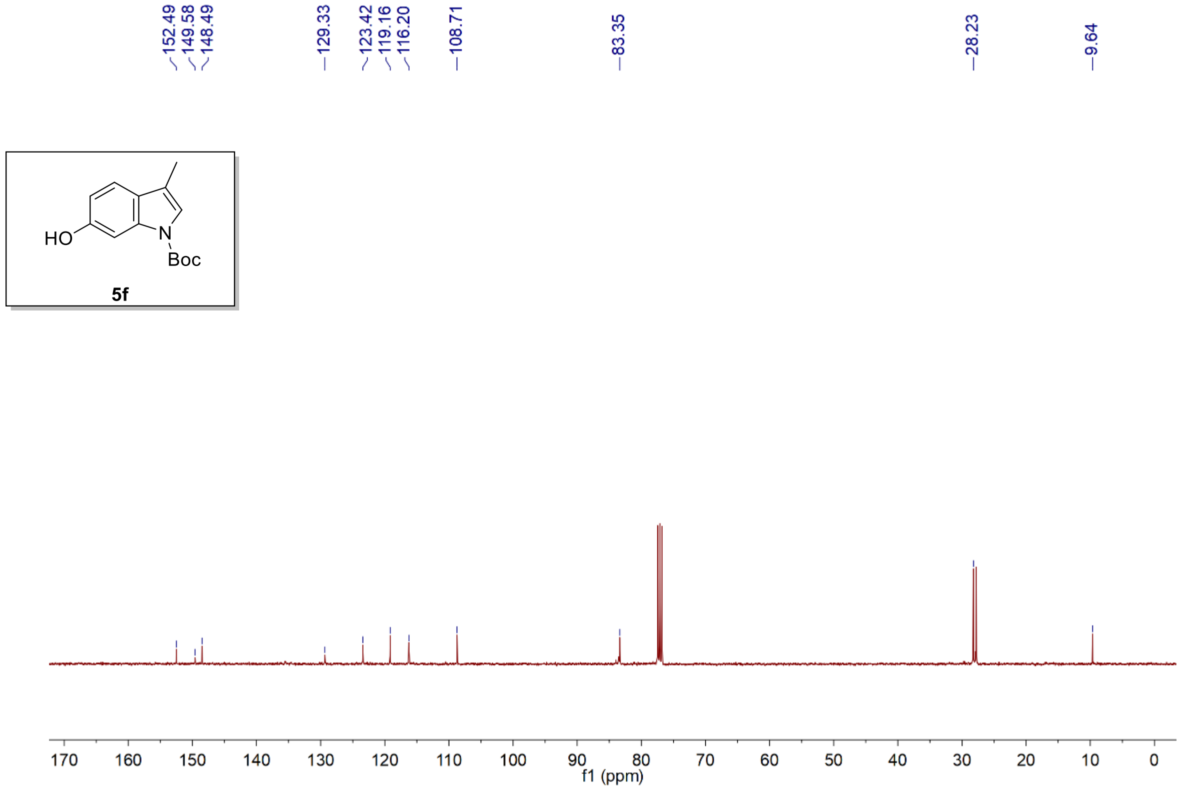

^{1}H-NMR spectrum of compound **5g** (400 MHz, $CDCl_3$)

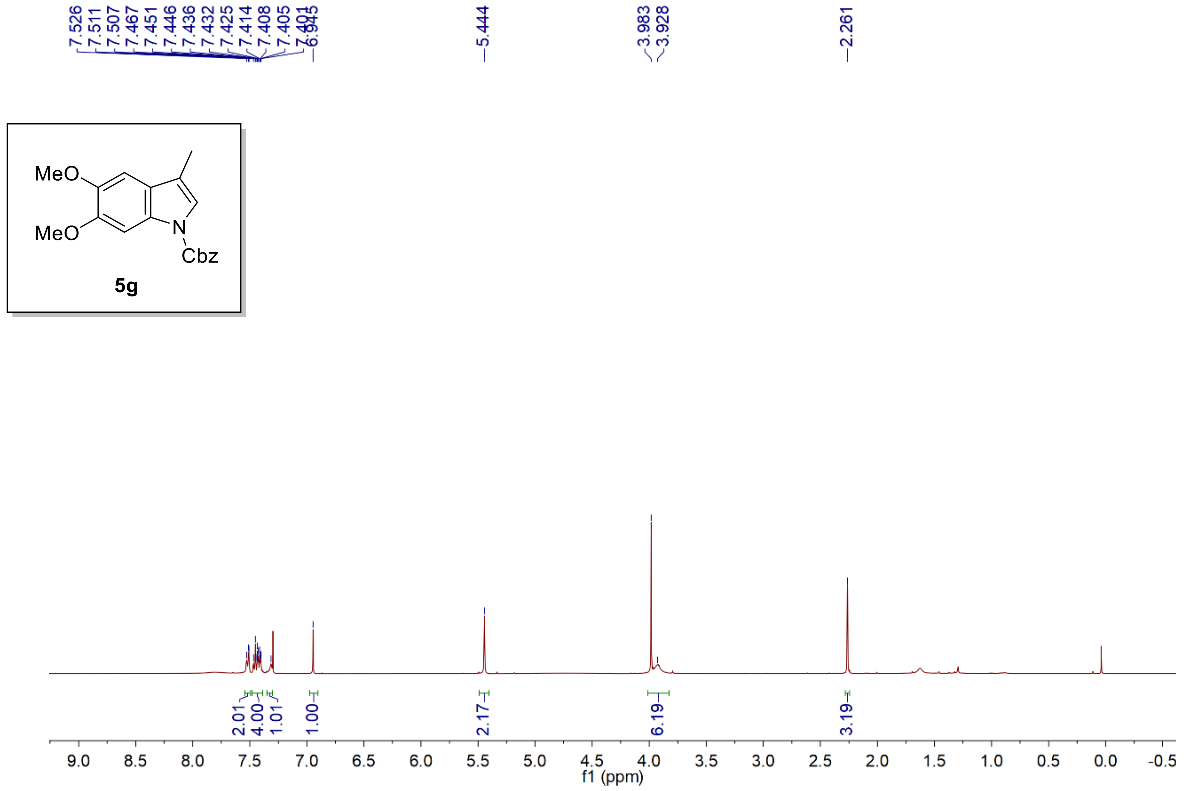

^{13}C-NMR spectrum of compound **5g** (101 MHz, $CDCl_3$)

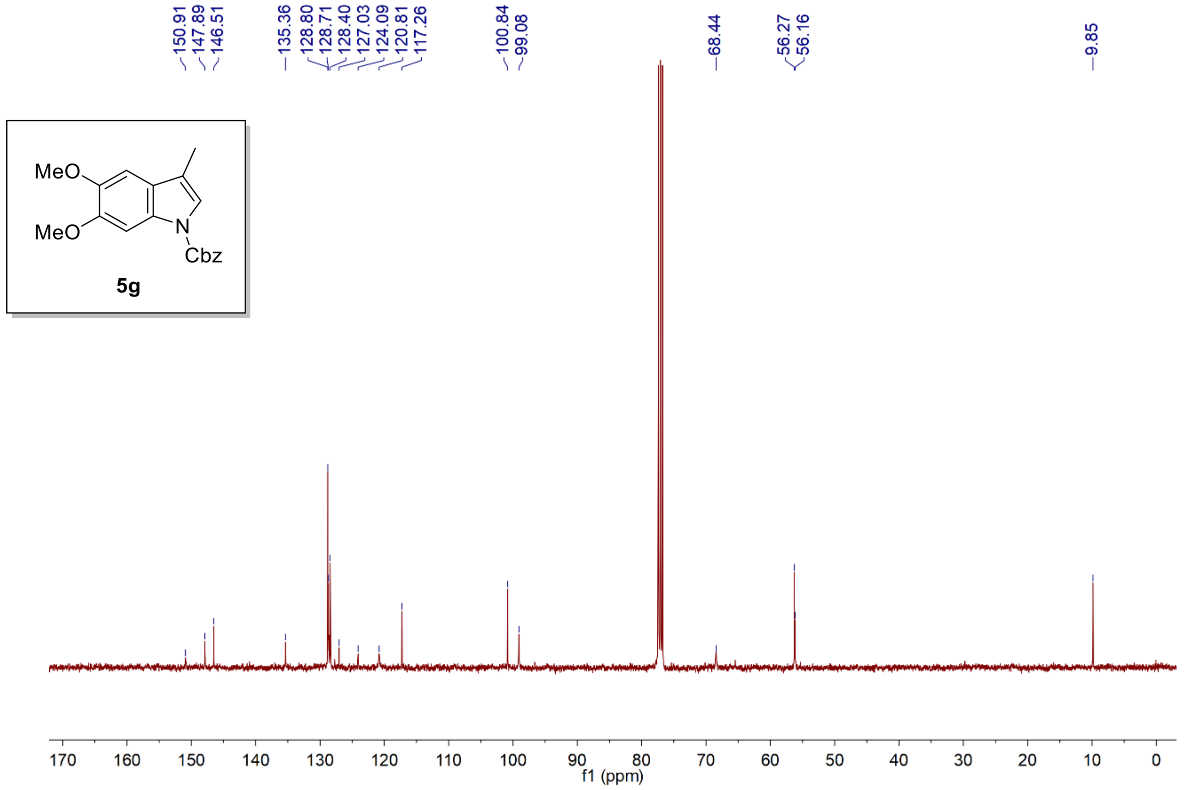

^{1}H-NMR spectrum of compound **5h** (400 MHz, $CDCl_3$)

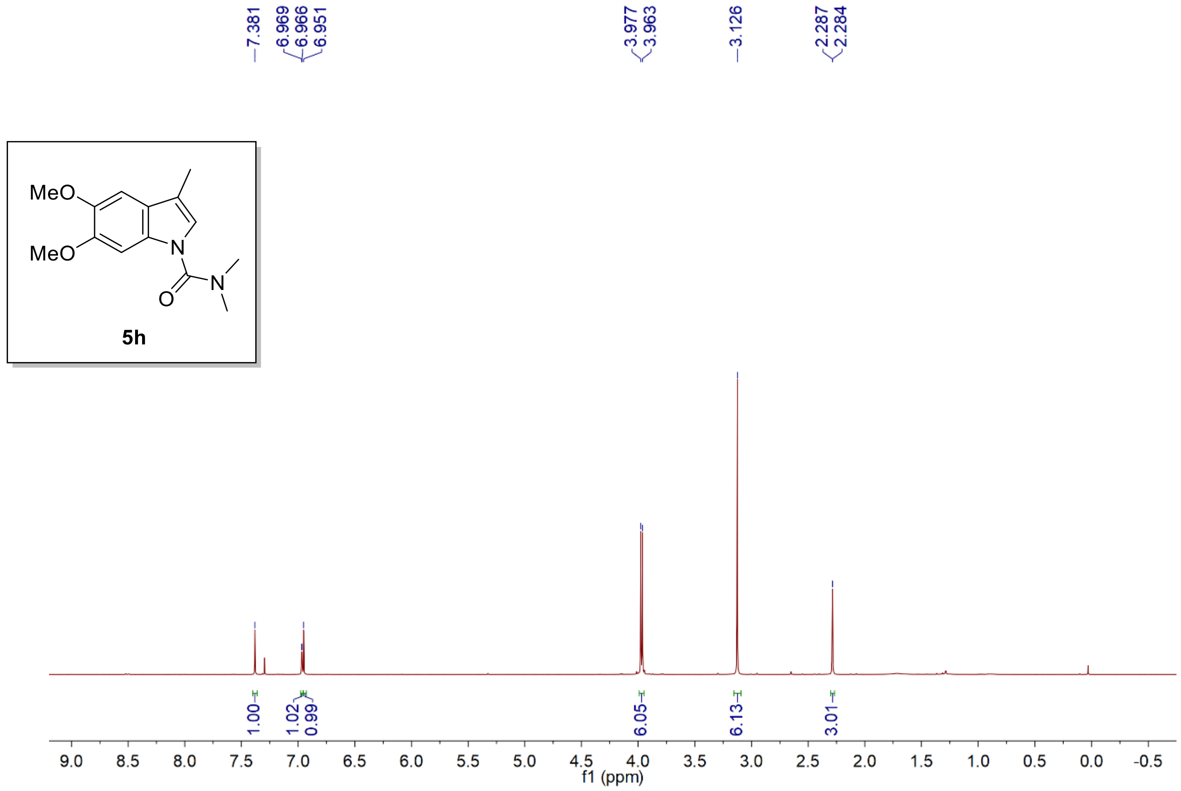

^{13}C-NMR spectrum of compound **5h** (101 MHz, $CDCl_3$)

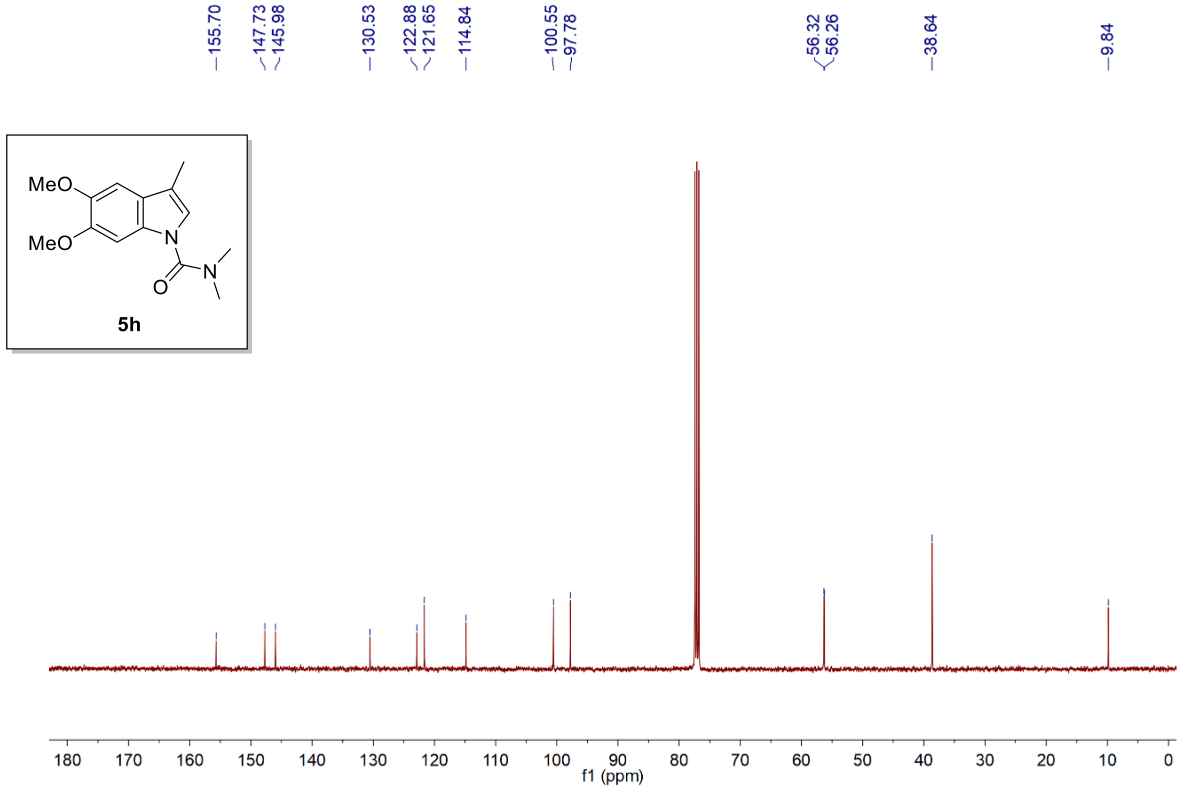

^{1}H-NMR spectrum of compound **10a** (400 MHz, $CDCl_3$)

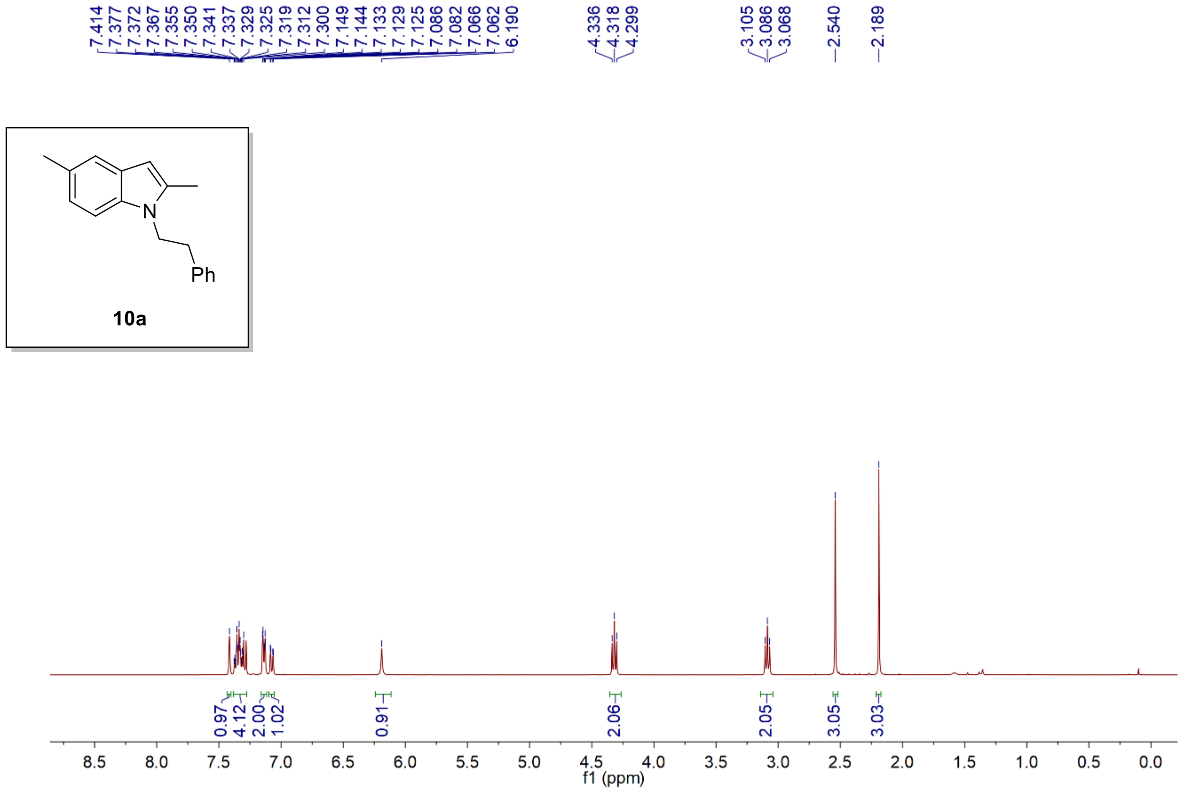

^{13}C-NMR spectrum of compound **10a** (101 MHz, $CDCl_3$)

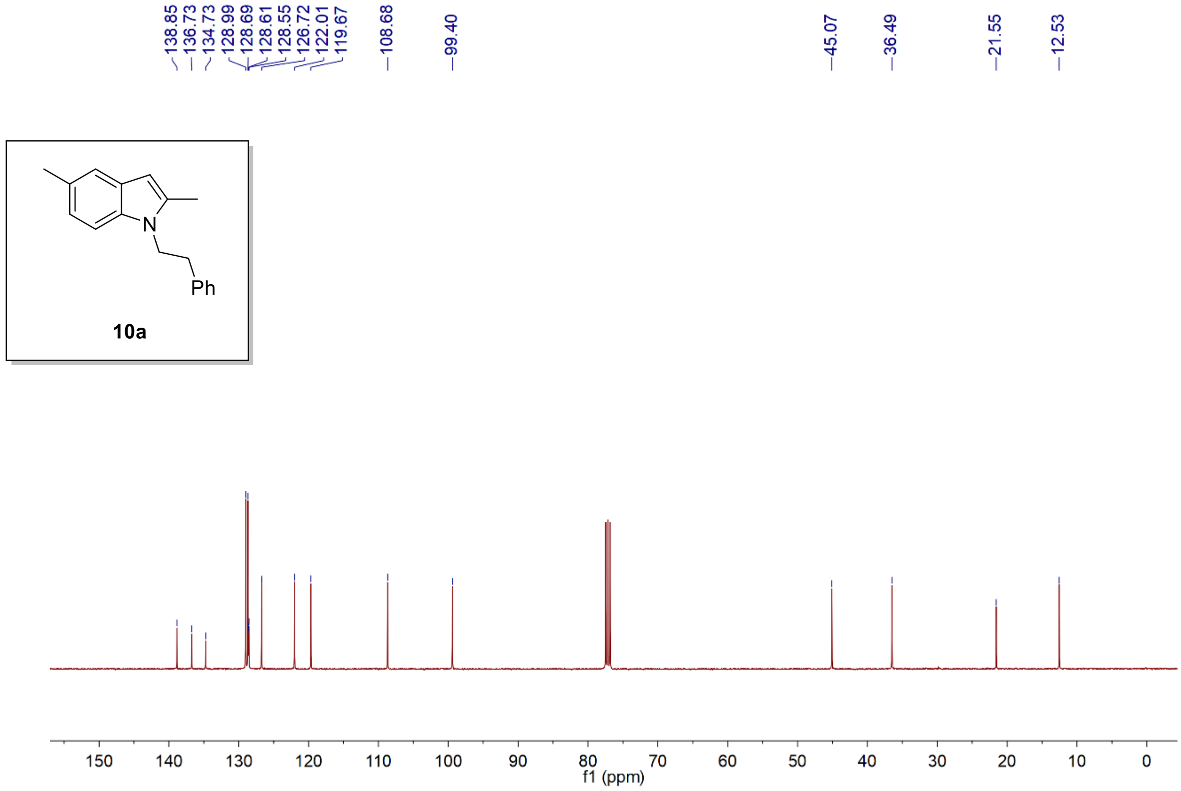

^{1}H-NMR spectrum of compound **11a** (400 MHz, $CDCl_3$)

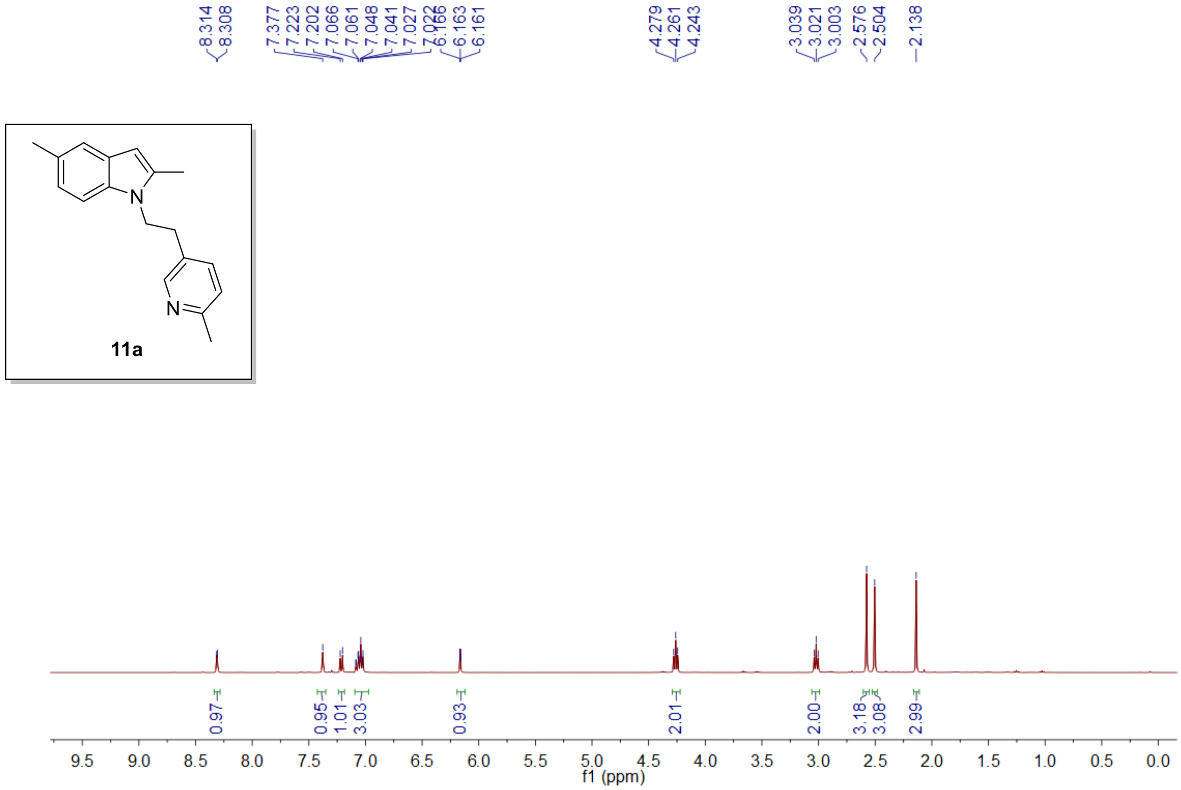

^{13}C-NMR spectrum of compound **11a** (101 MHz, $CDCl_3$)

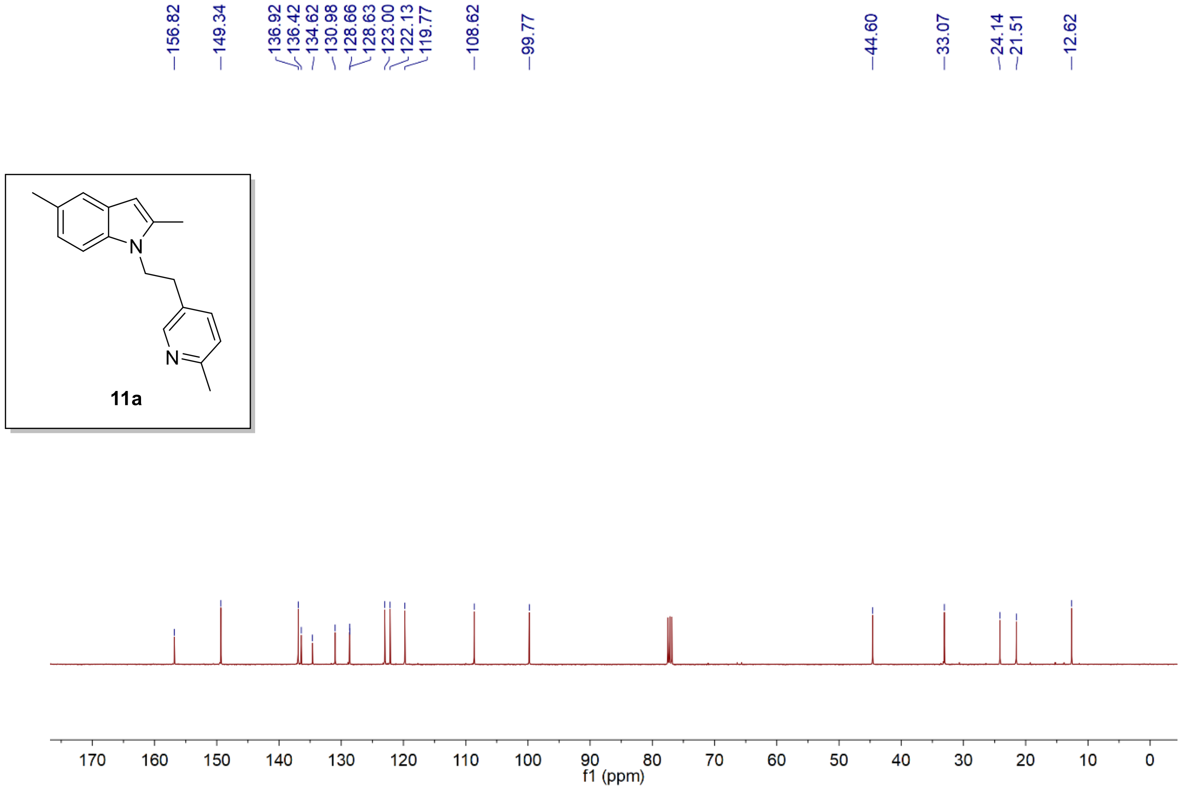

^{1}H-NMR spectrum of compound [D]-**1a** (400 MHz, $CDCl_3$)

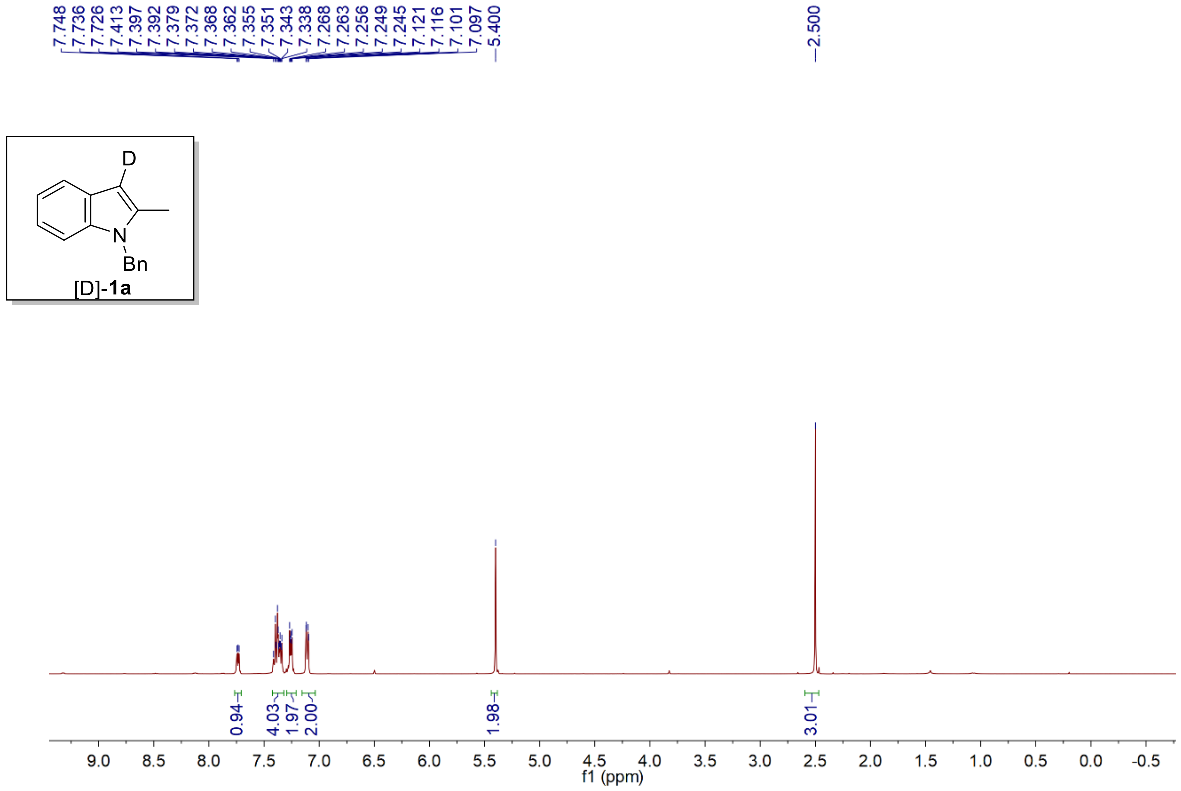

^{13}C-NMR spectrum of compound [D]-**1a** (101 MHz, $CDCl_3$)

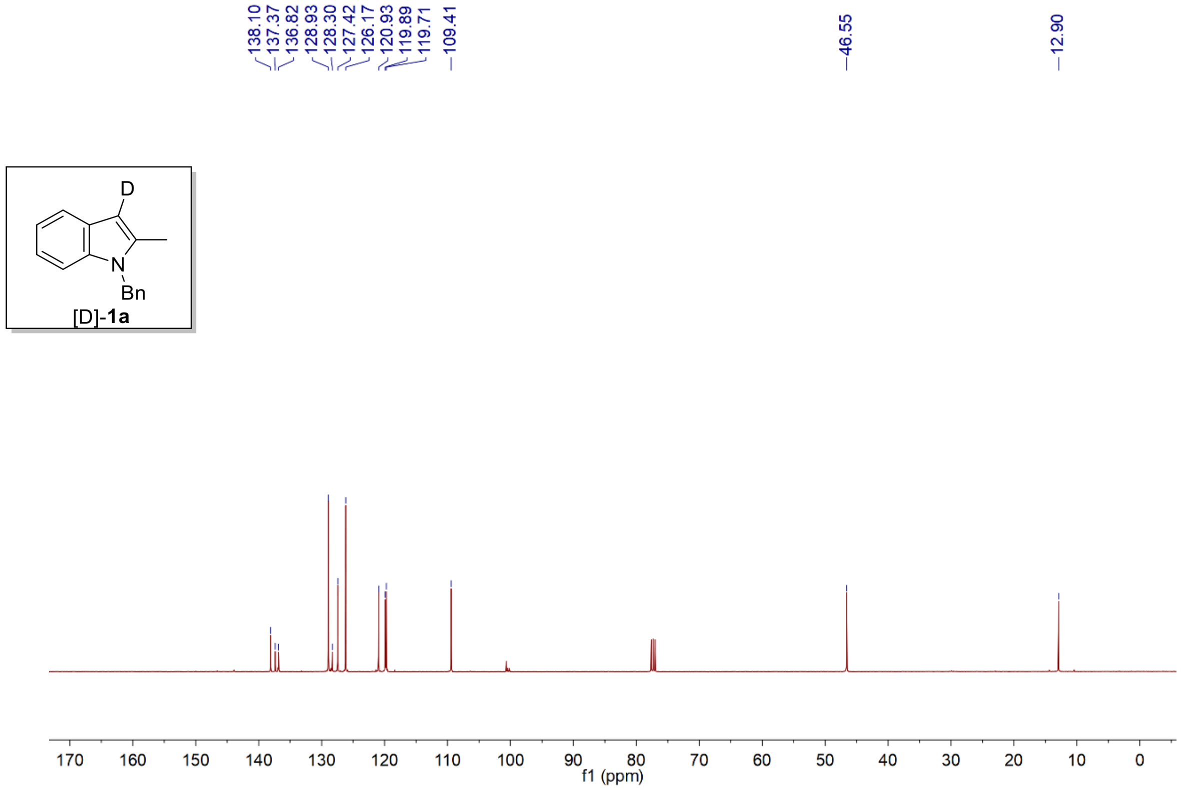

^{1}H-NMR spectrum of compound [D3]-**7a** (400 MHz, $CDCl_3$)

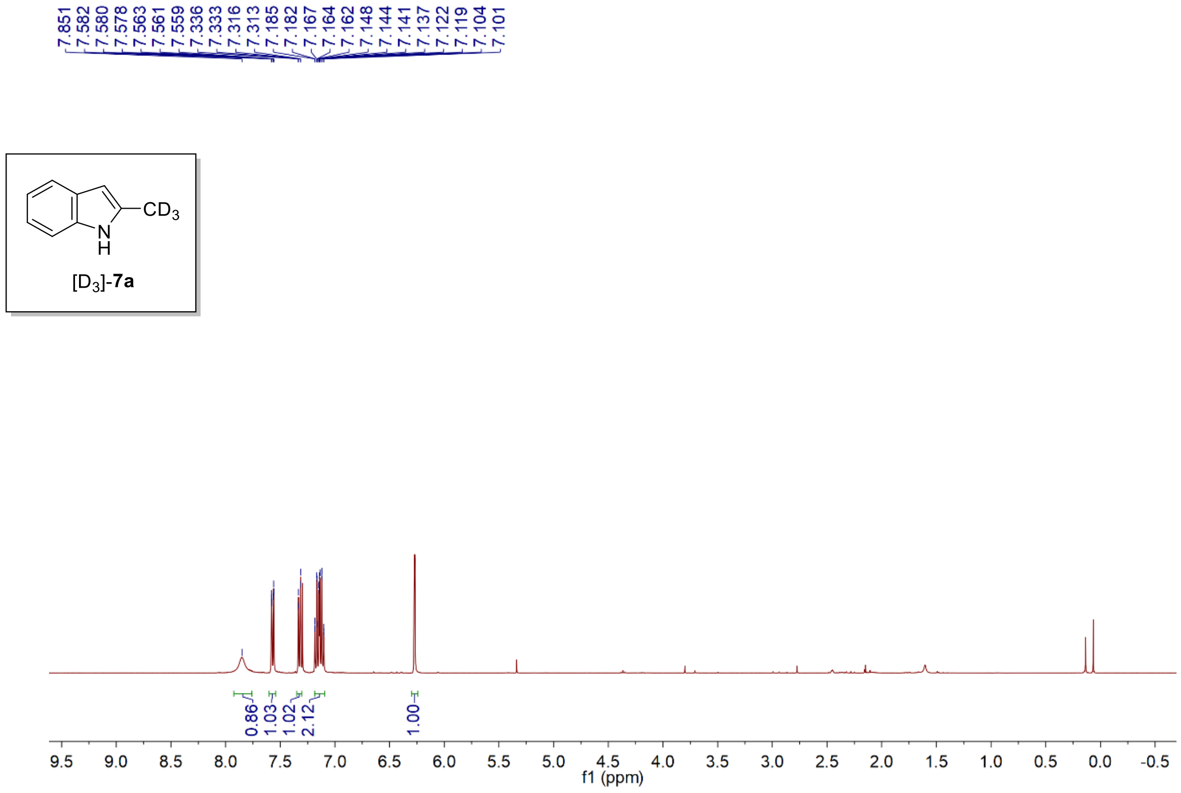

^{13}C-NMR spectrum of compound [D3]-**7a** (101 MHz, $CDCl_3$)

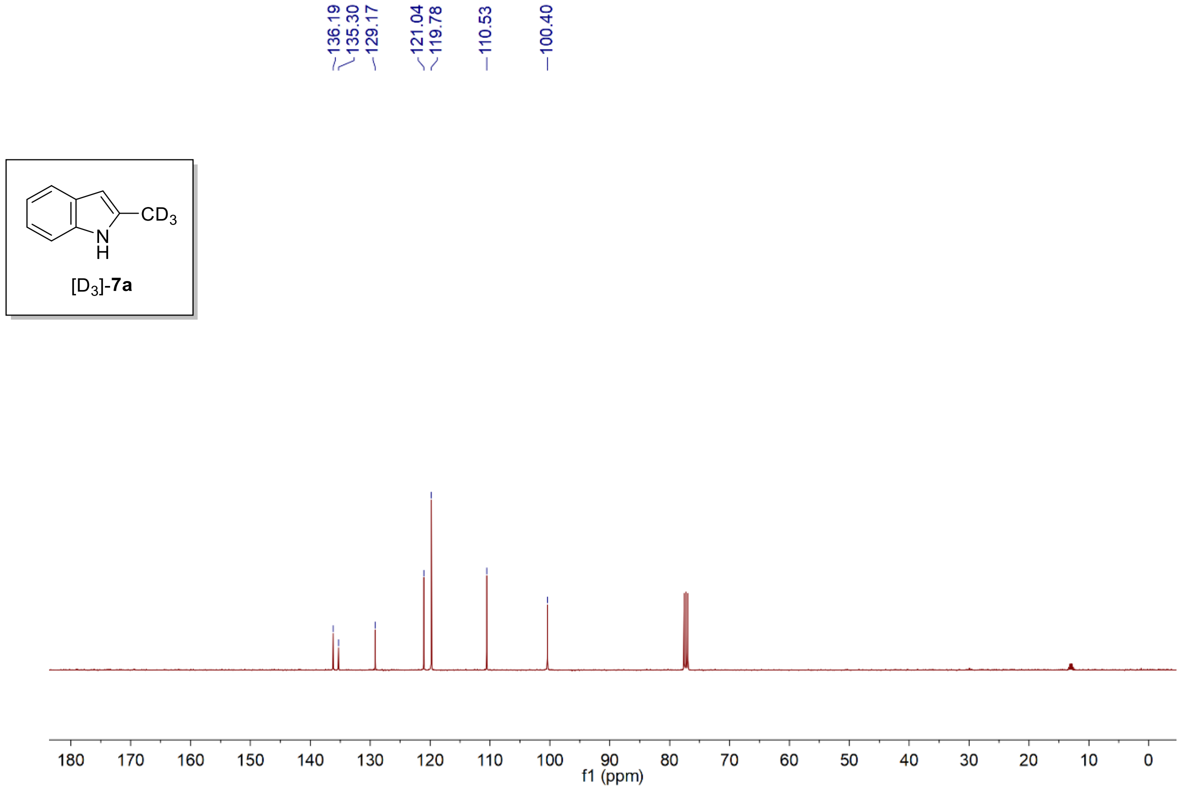

^{1}H-NMR spectrum of compound [D_3]-**1a** (400 MHz, $CDCl_3$)

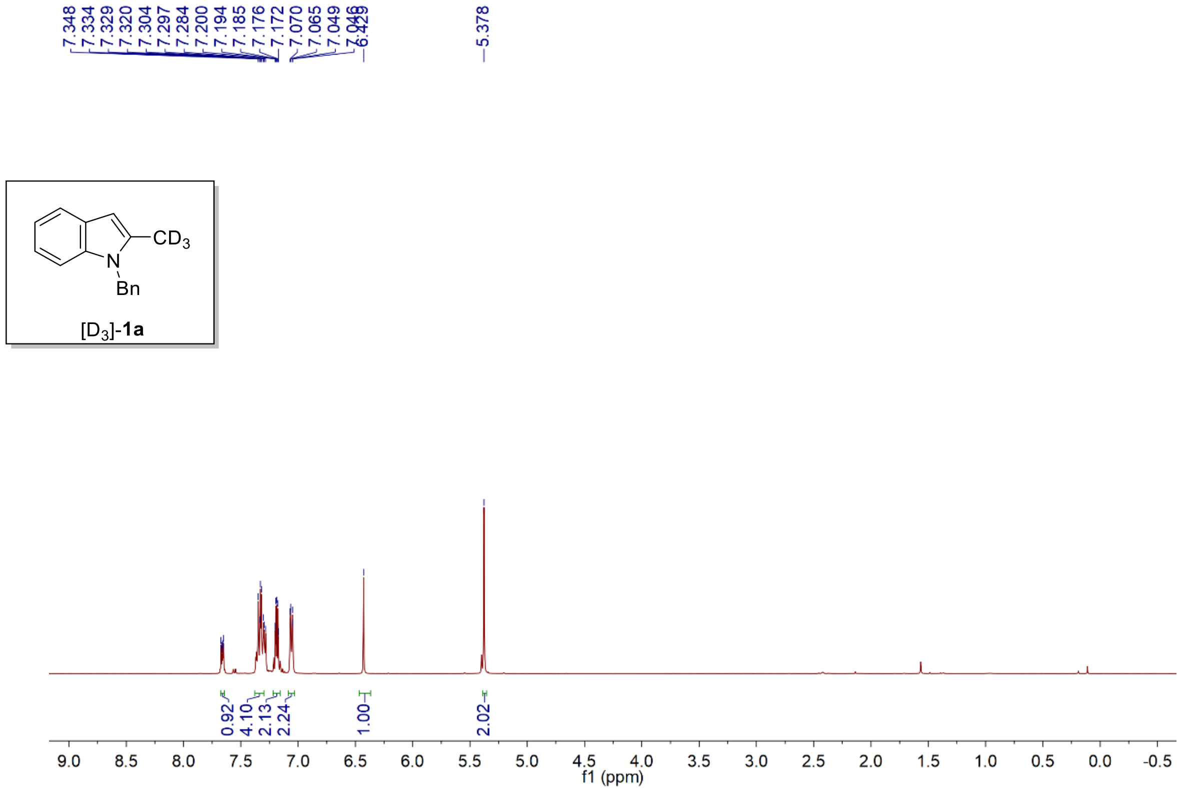

^{13}C-NMR spectrum of compound [D_3]-**1a** (101 MHz, $CDCl_3$)

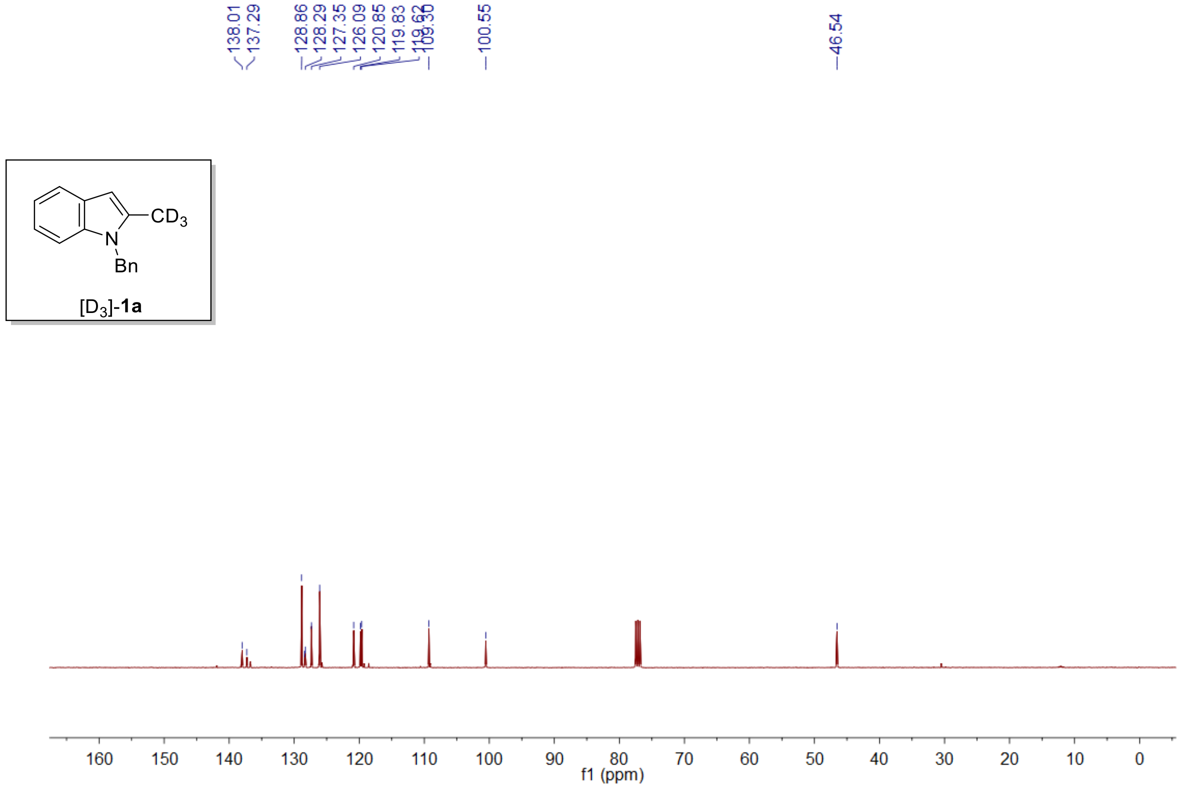

^{1}H-NMR spectrum of compound **M-S2** (400 MHz, $CDCl_3$)

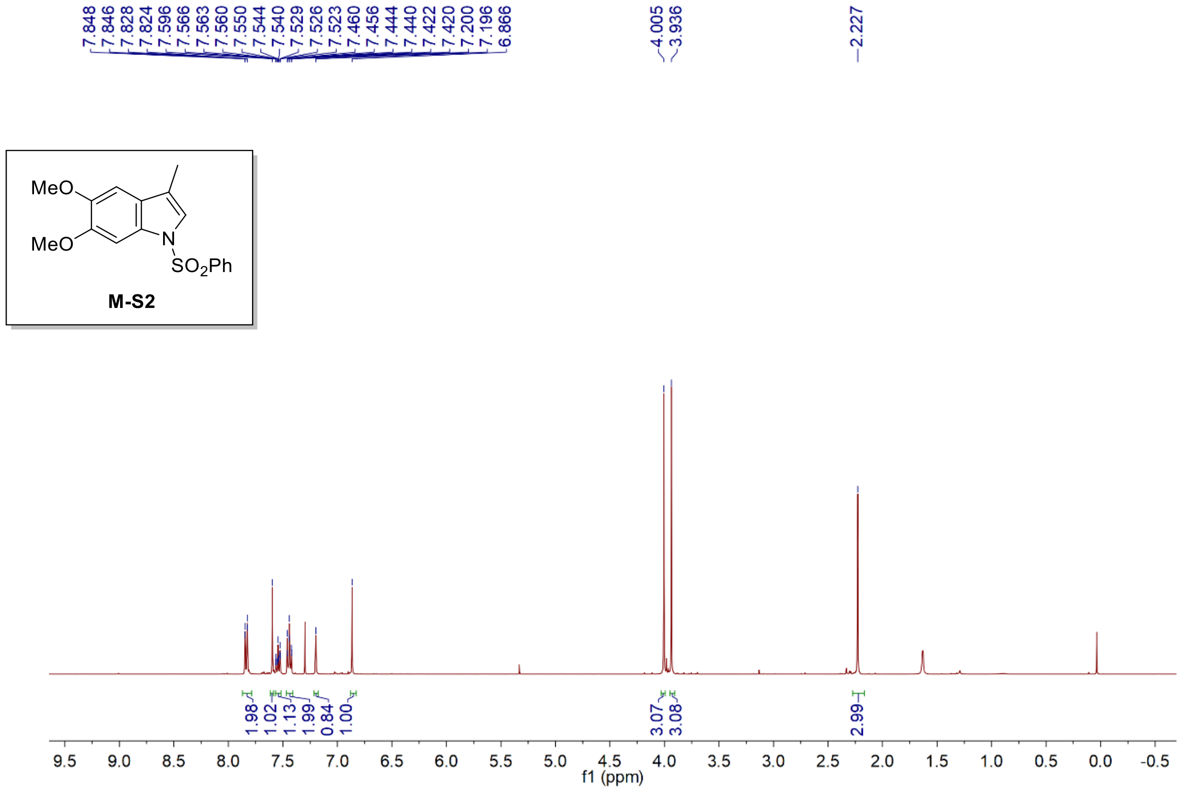

^{13}C-NMR spectrum of compound **M-S2** (101 MHz, $CDCl_3$)

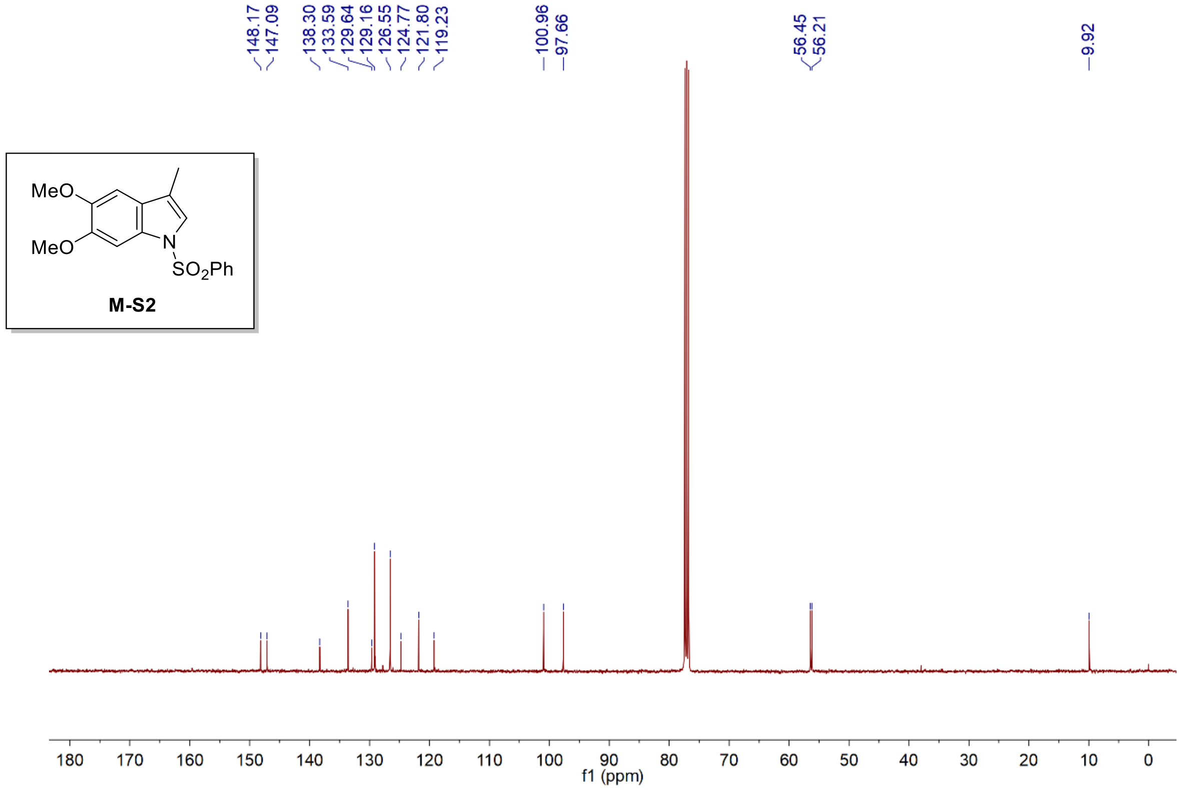

^{1}H-NMR spectrum of compound [D]-**7o** (400 MHz, $CDCl_3$)

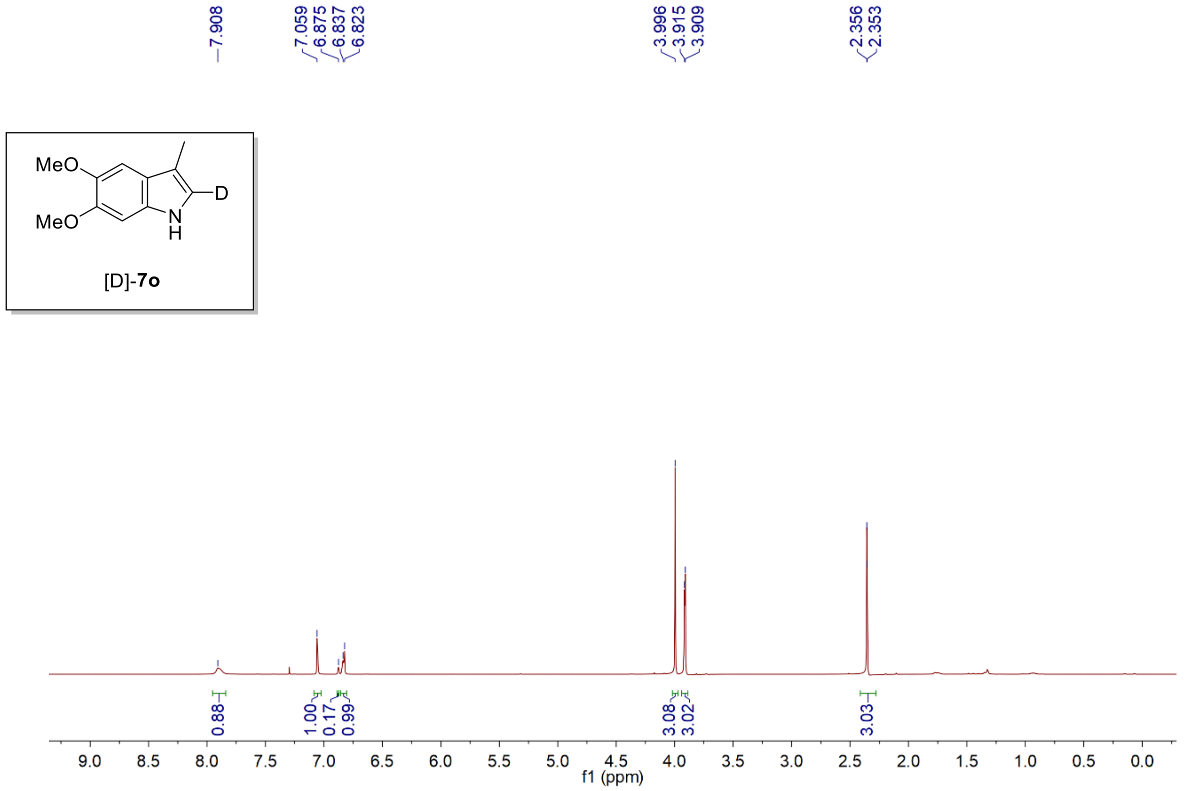

^{13}C-NMR spectrum of compound [D]-**7o** (101 MHz, $CDCl_3$)

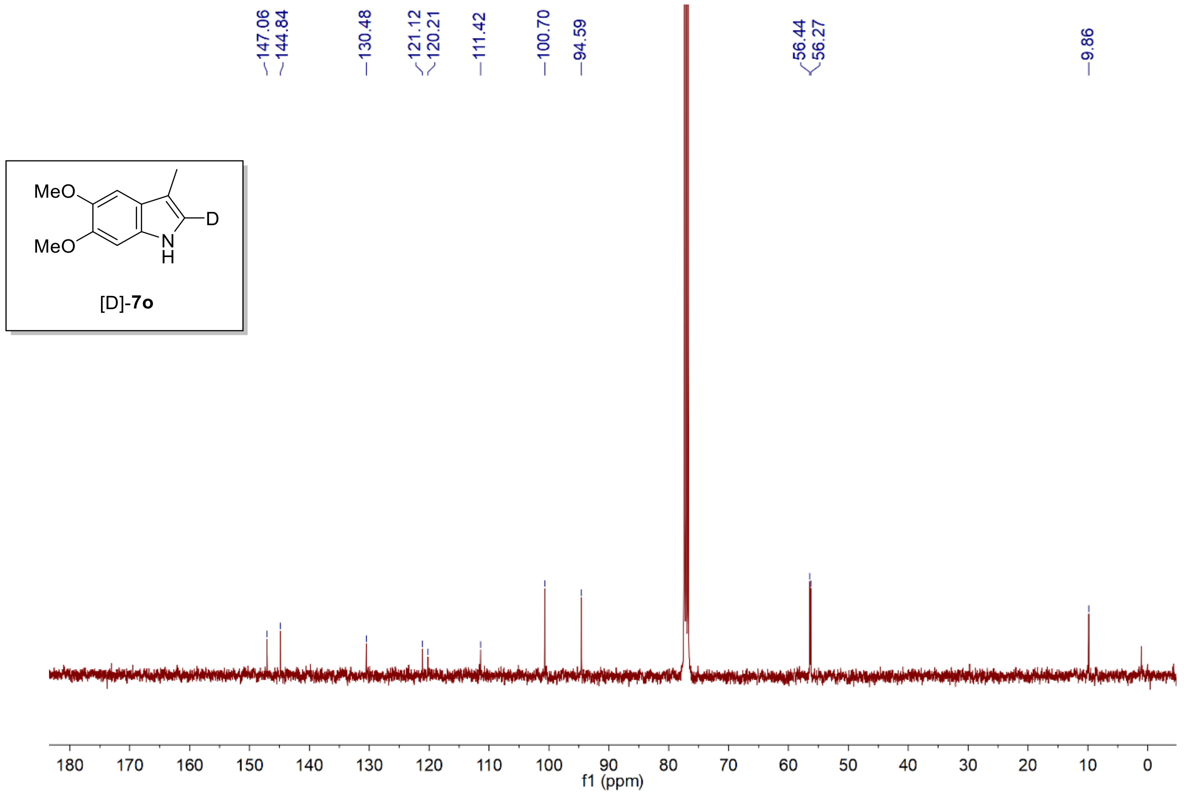

^{1}H-NMR spectrum of compound [D]-**5a** (400 MHz, $CDCl_3$)

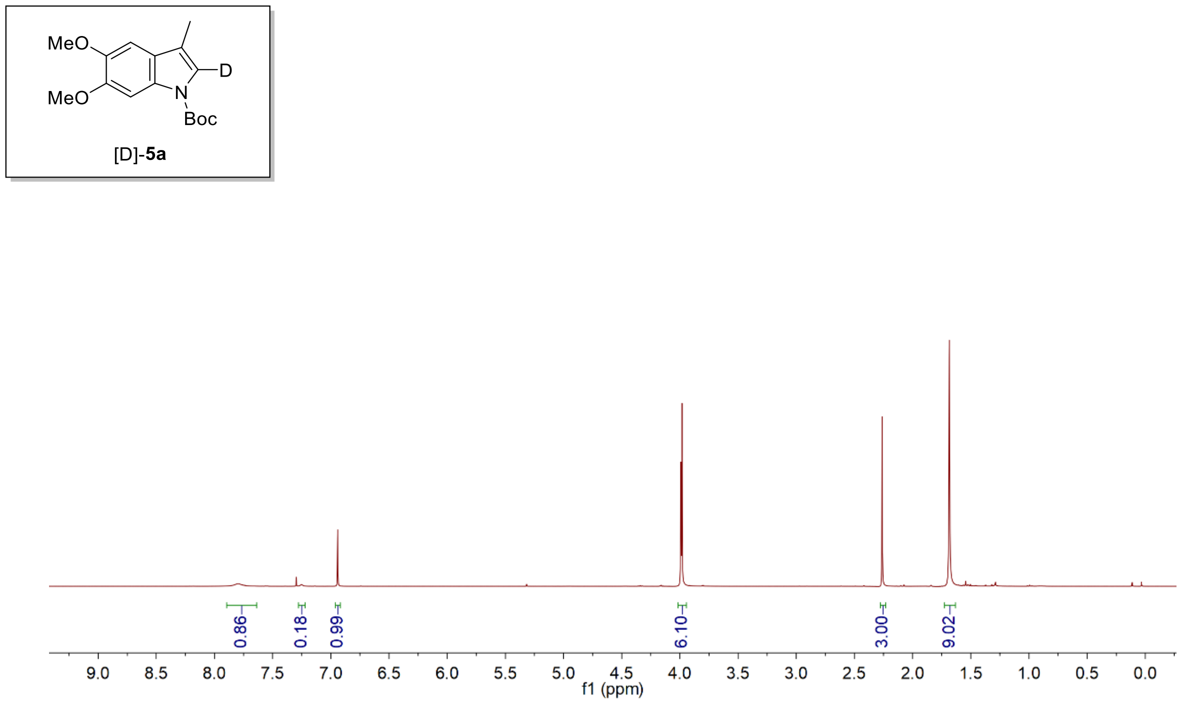

^{13}C-NMR spectrum of compound [D]-**5a** (101 MHz, $CDCl_3$)

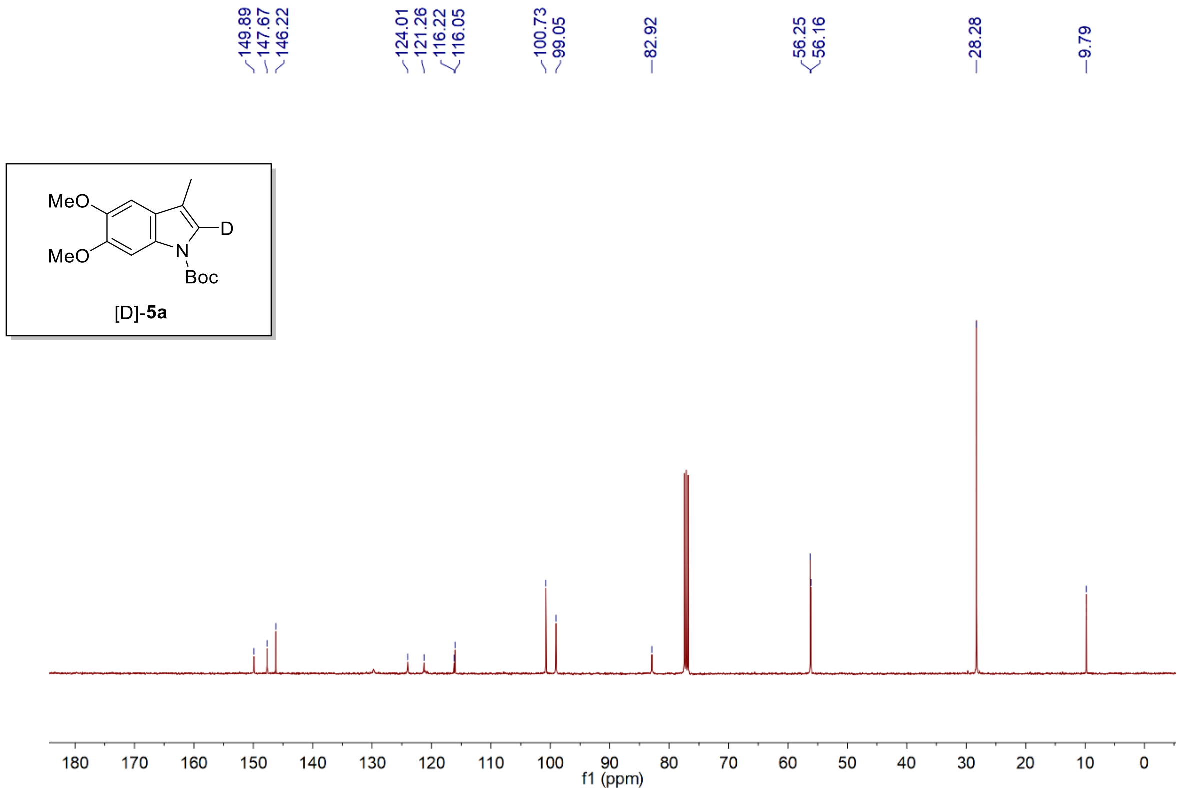

^{1}H-NMR spectrum of compound **N-S2** (400 MHz, $CDCl_3$)

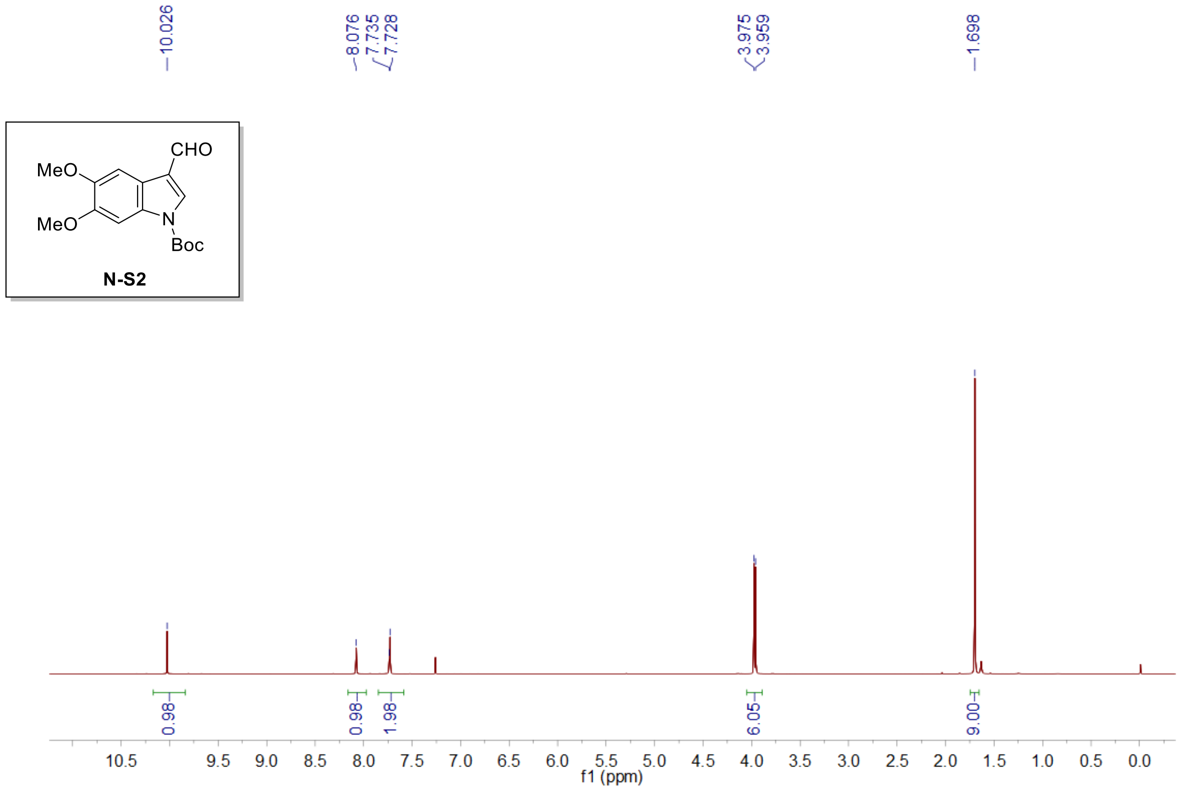

^{13}C-NMR spectrum of compound **N-S2** (101 MHz, $CDCl_3$)

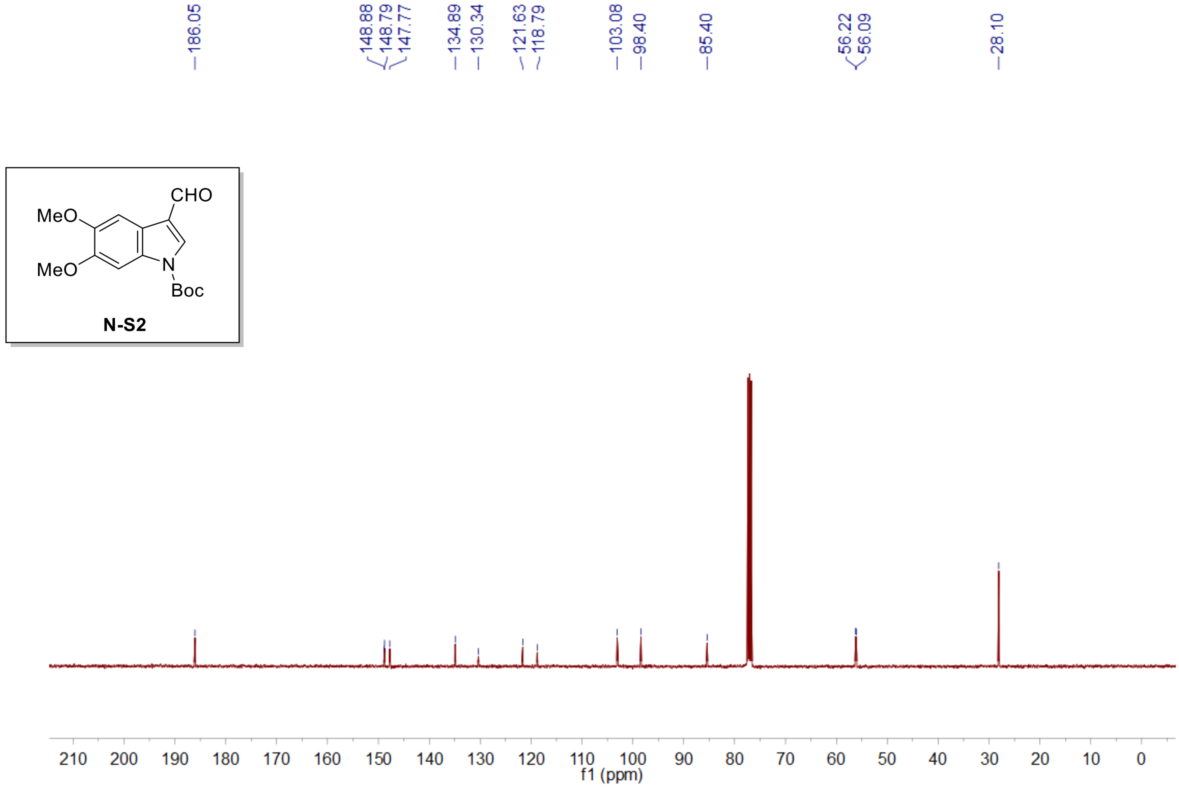

^{1}H-NMR spectrum of compound **N-S4** (400 MHz, $CDCl_3$)

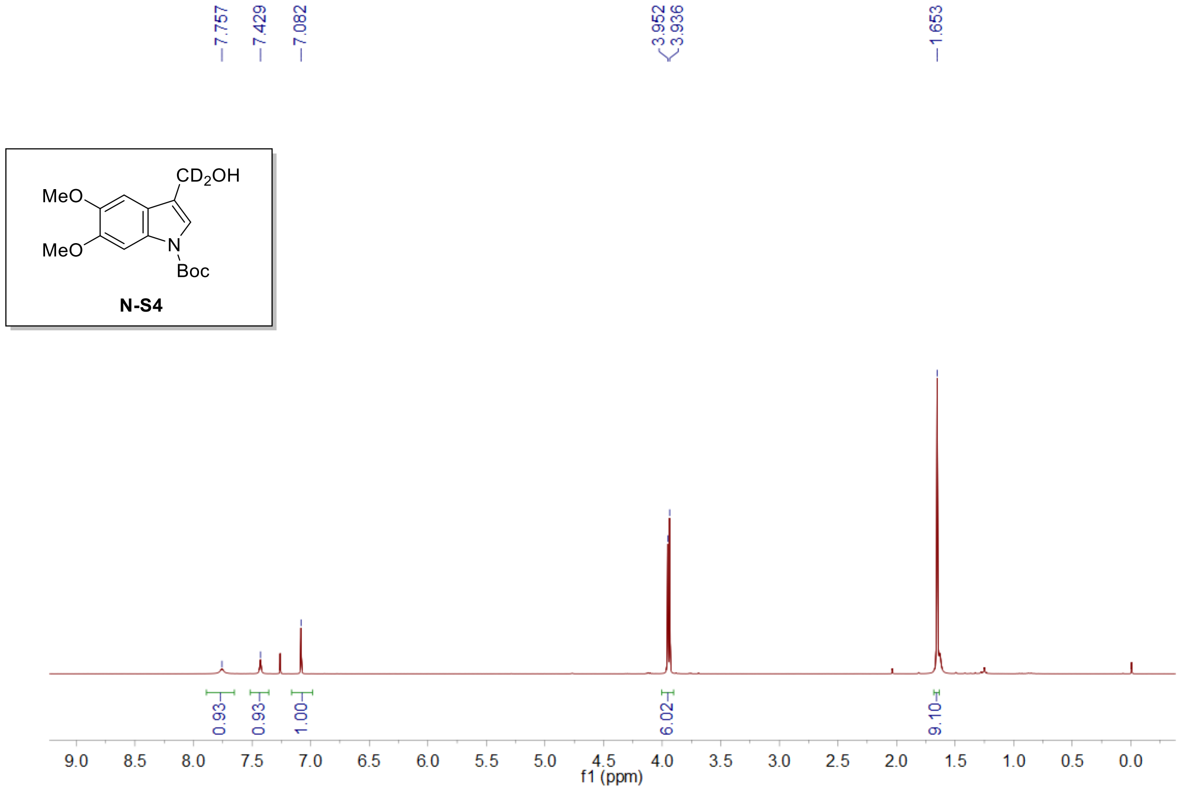

^{13}C-NMR spectrum of compound **N-S4** (101 MHz, $CDCl_3$)

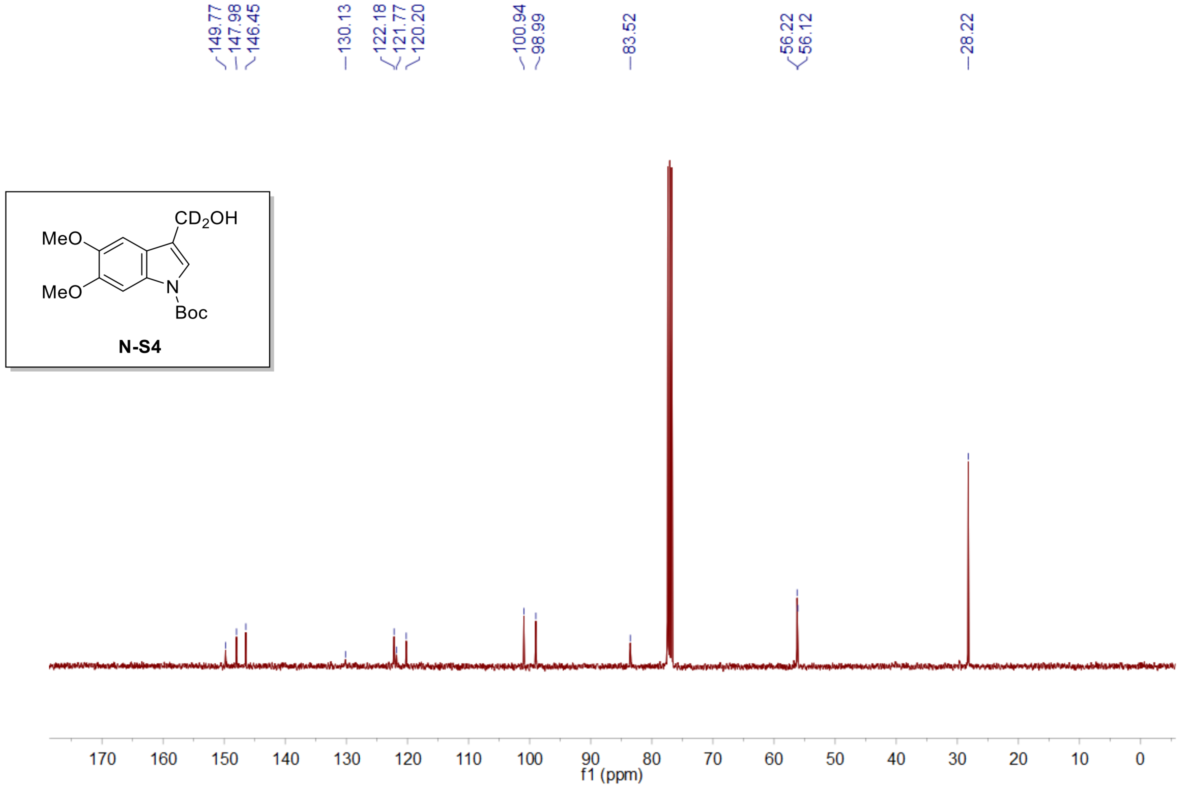

^{1}H-NMR spectrum of compound [D3]-**5a** (400 MHz, $CDCl_3$)

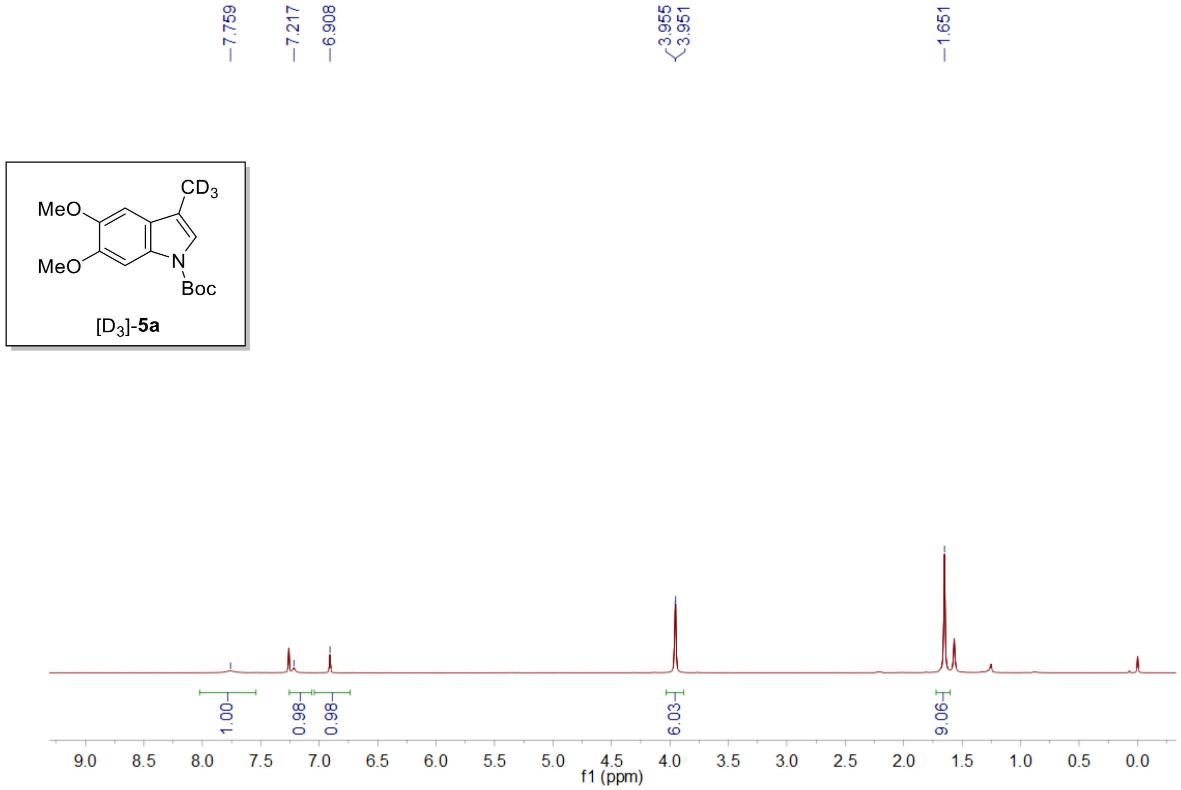

^{13}C-NMR spectrum of compound [D3]-**5a** (101 MHz, $CDCl_3$)

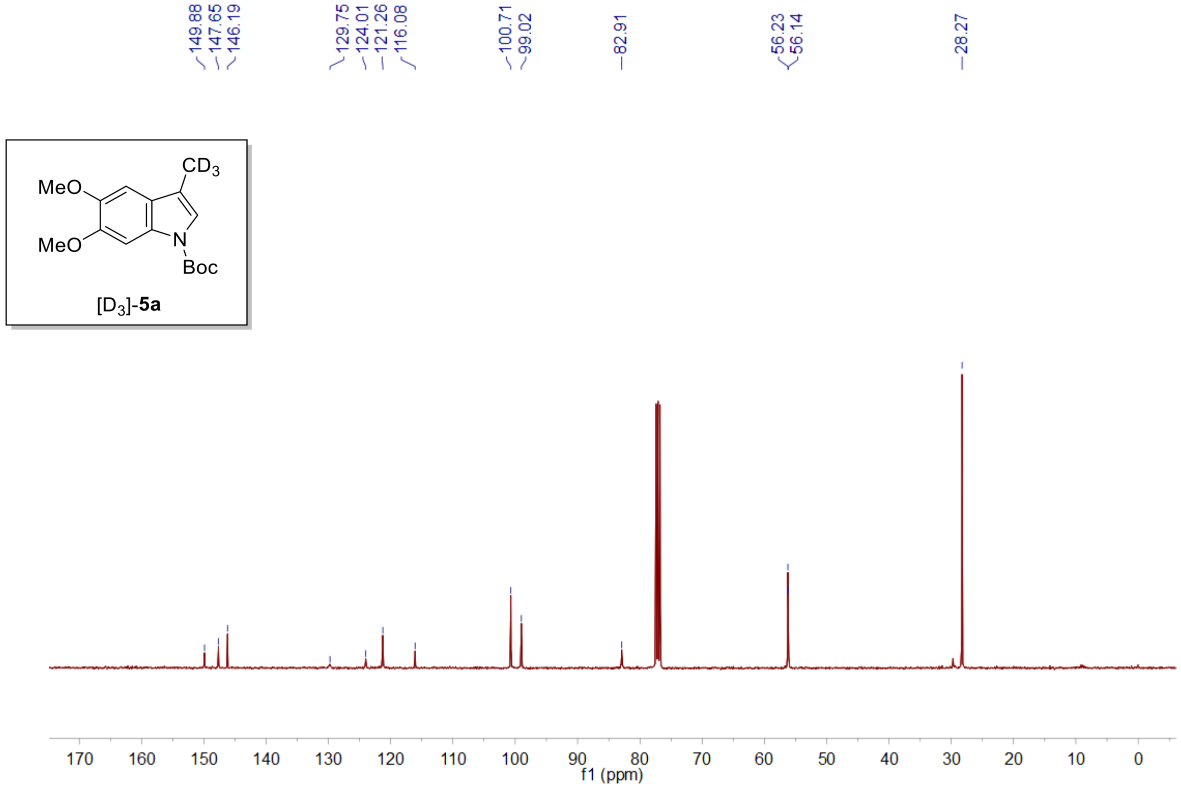

^{1}H-NMR spectrum of compound **4a** (400 MHz, $CDCl_3$)

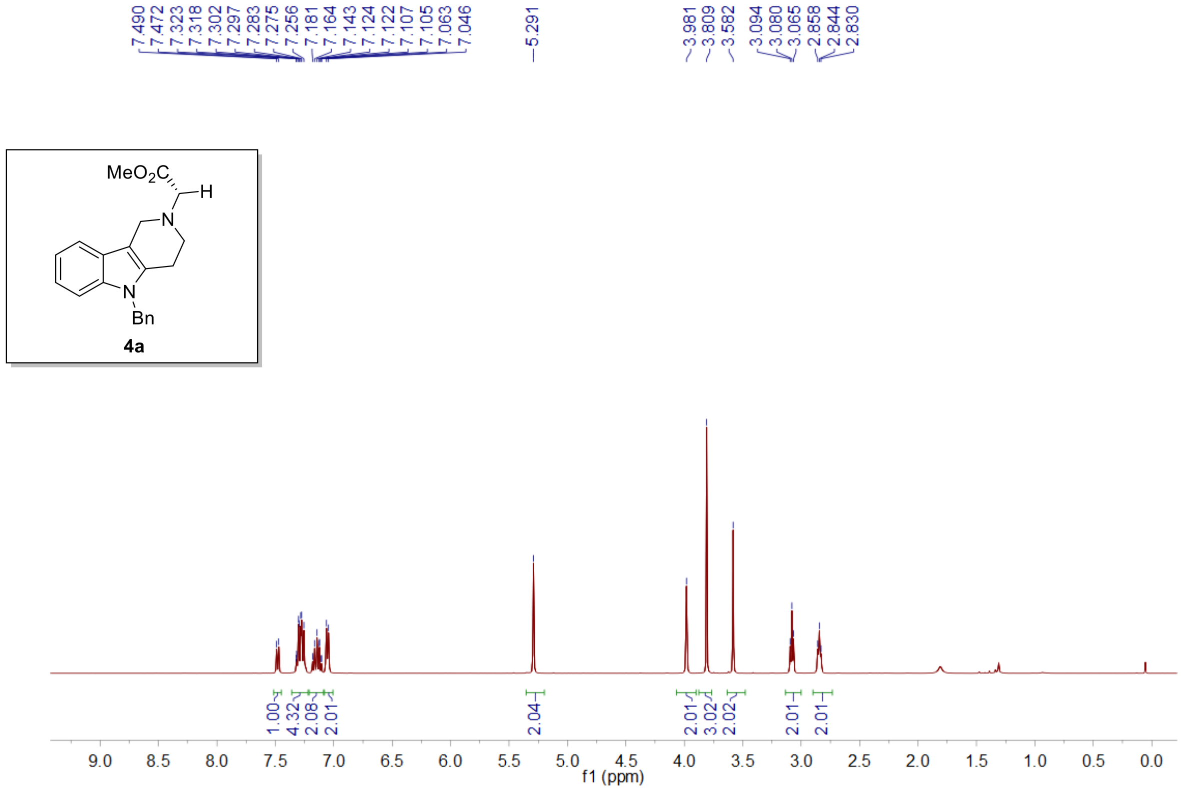

^{13}C-NMR spectrum of compound **4a** (101 MHz, $CDCl_3$)

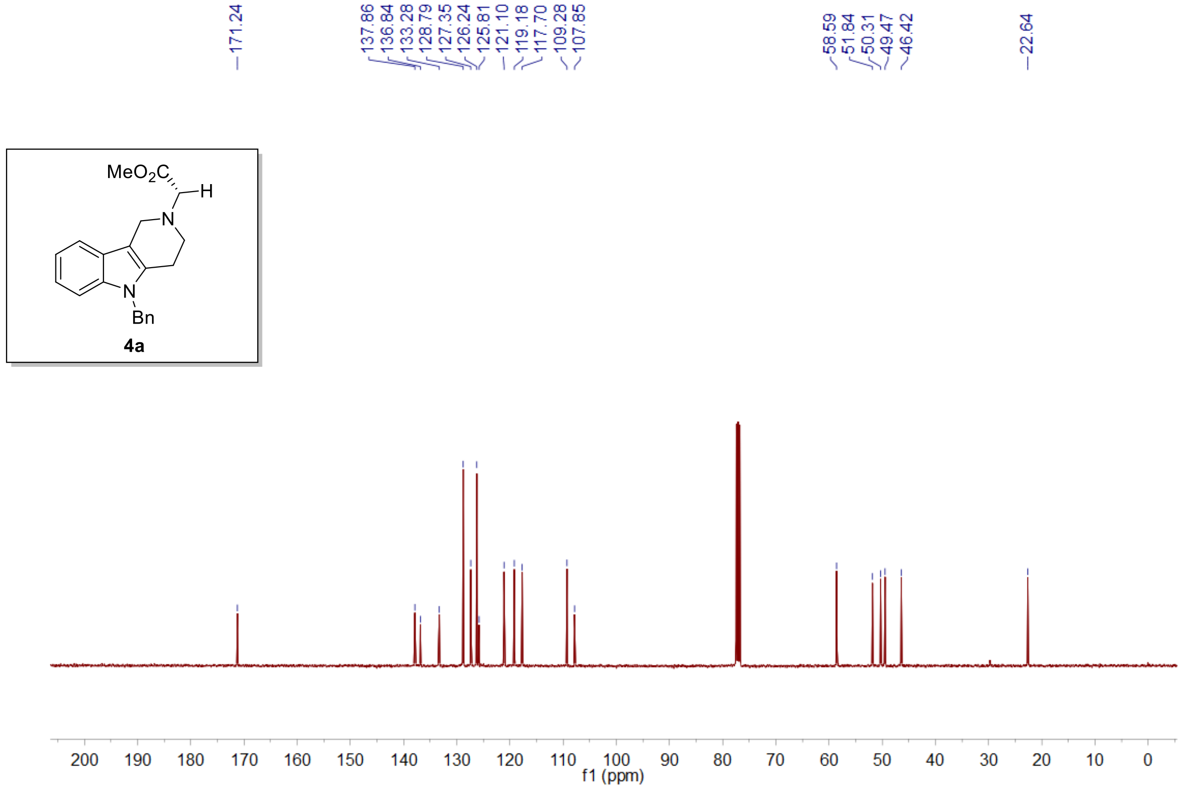

^{1}H-NMR spectrum of compound **4b** (400 MHz, $CDCl_3$)

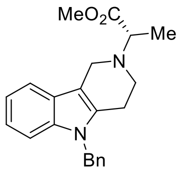

4b

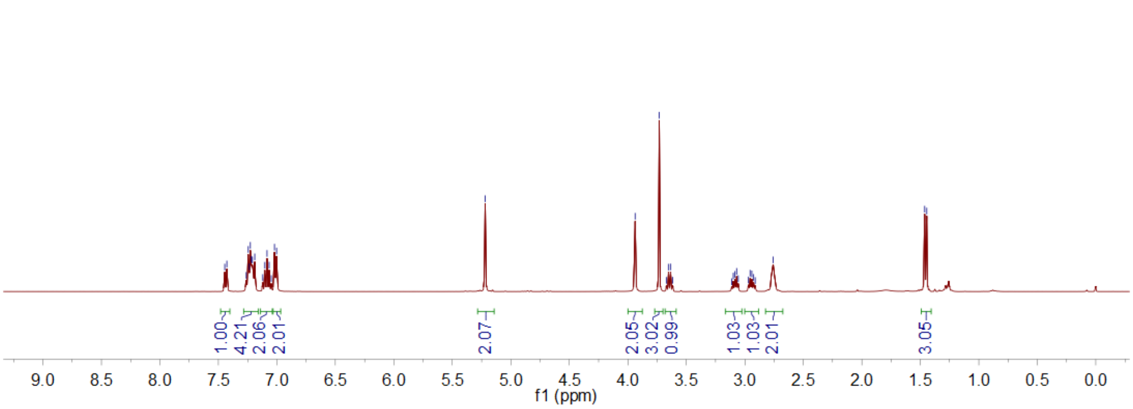

^{13}C-NMR spectrum of compound **4b** (101 MHz, $CDCl_3$)

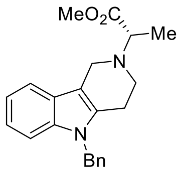

4b

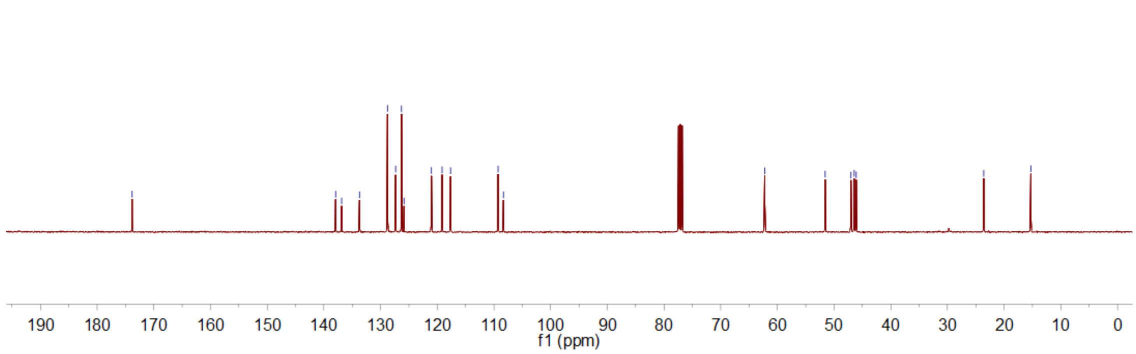

^{1}H-NMR spectrum of compound **4c** (400 MHz, $CDCl_3$)

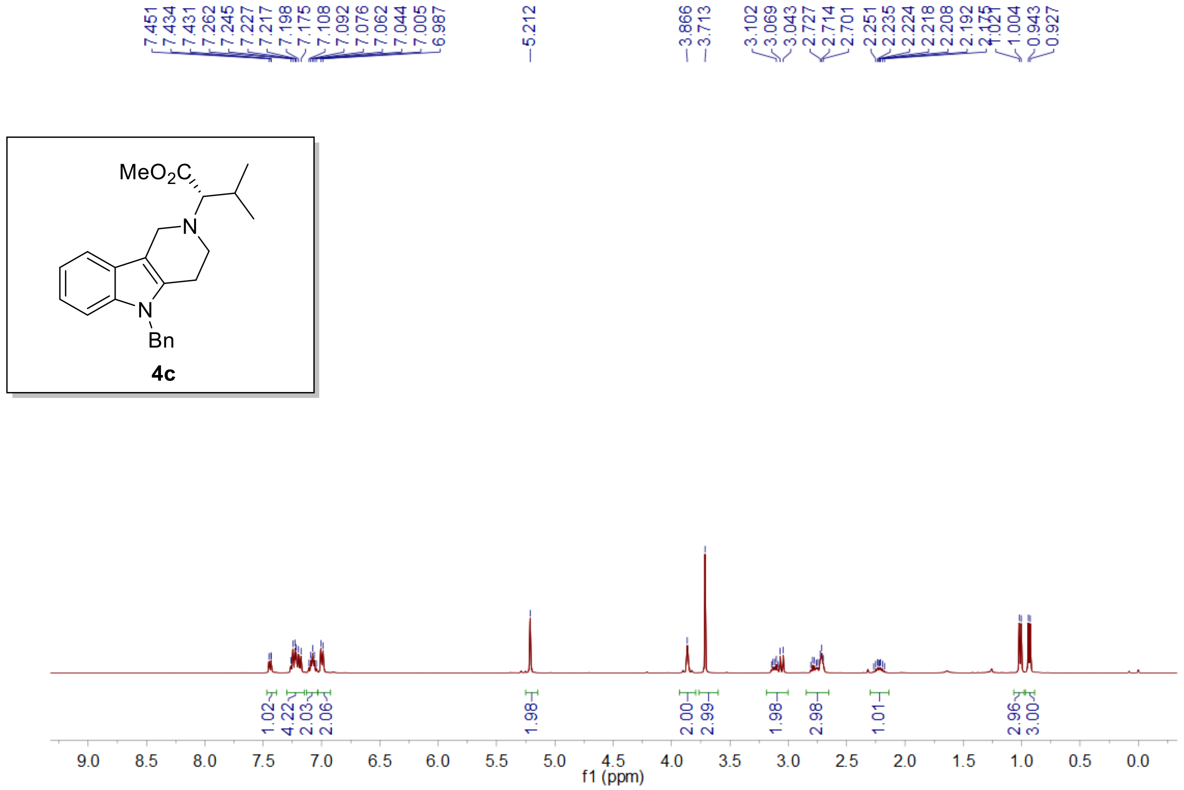

^{13}C-NMR spectrum of compound **4c** (101 MHz, $CDCl_3$)

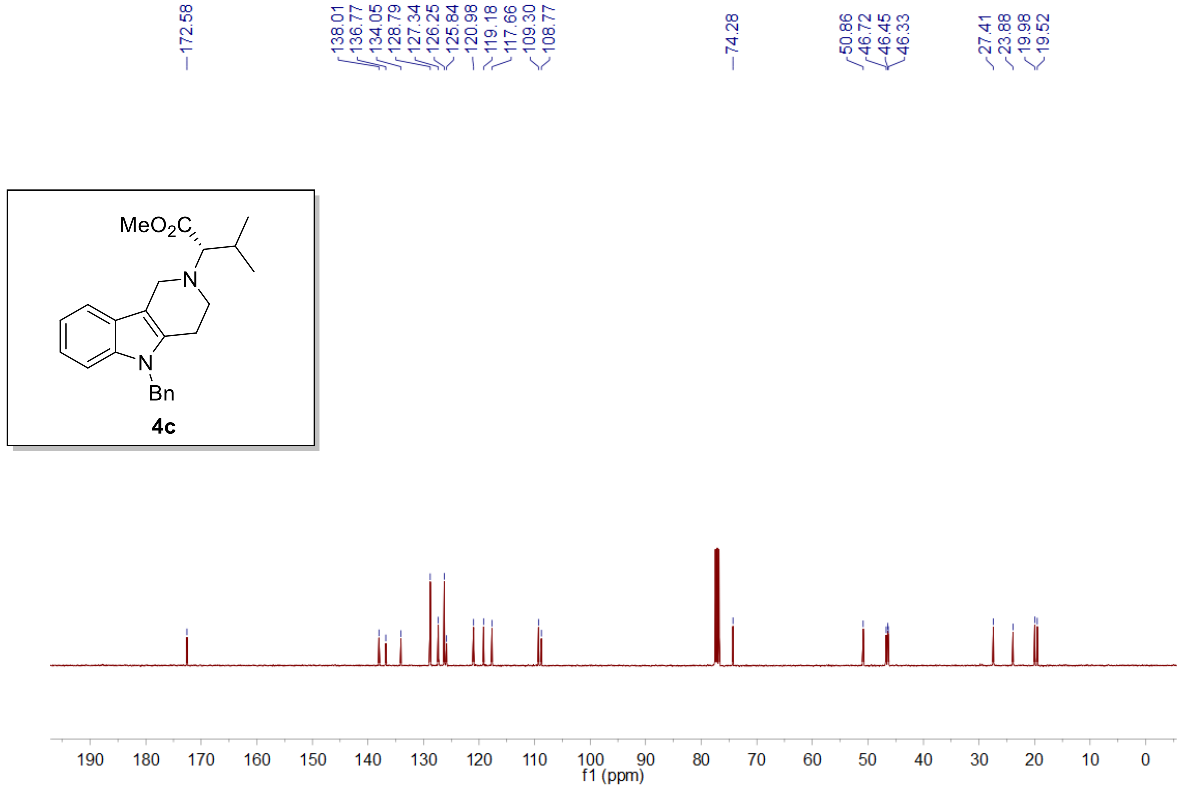

^{1}H-NMR spectrum of compound **4d** (400 MHz, $CDCl_3$)

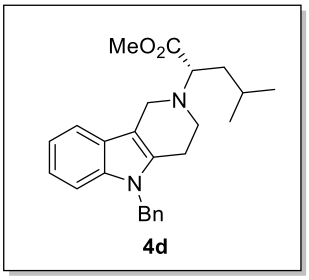

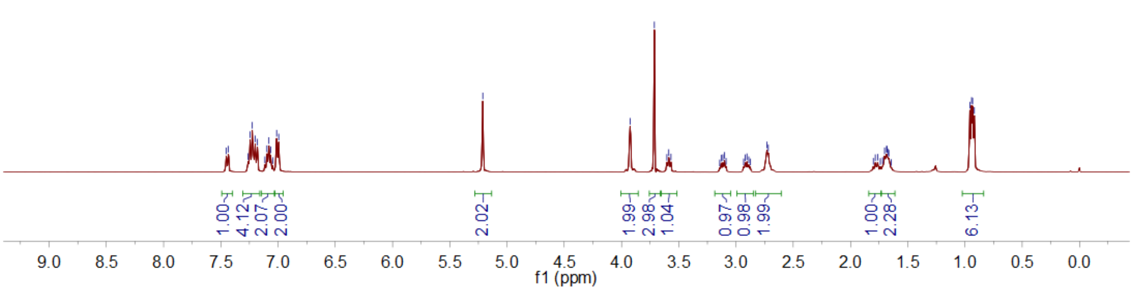

^{13}C-NMR spectrum of compound **4d** (101 MHz, $CDCl_3$)

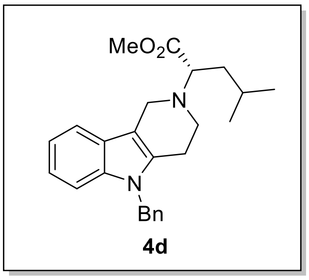

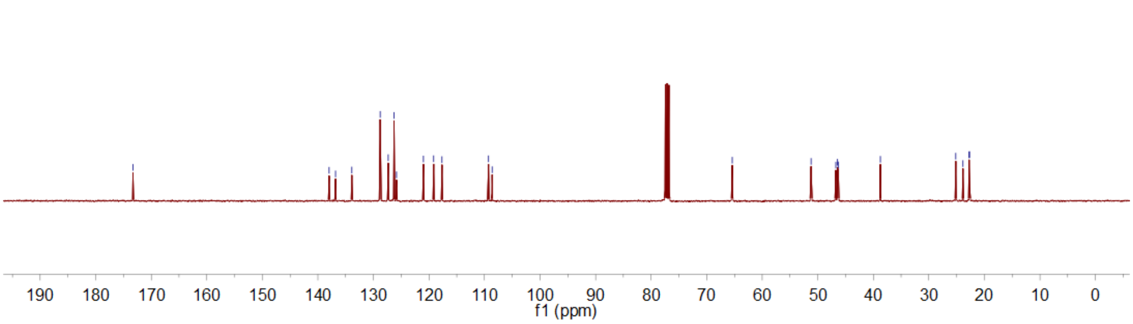

^{1}H-NMR spectrum of compound **4e** (400 MHz, $CDCl_3$)

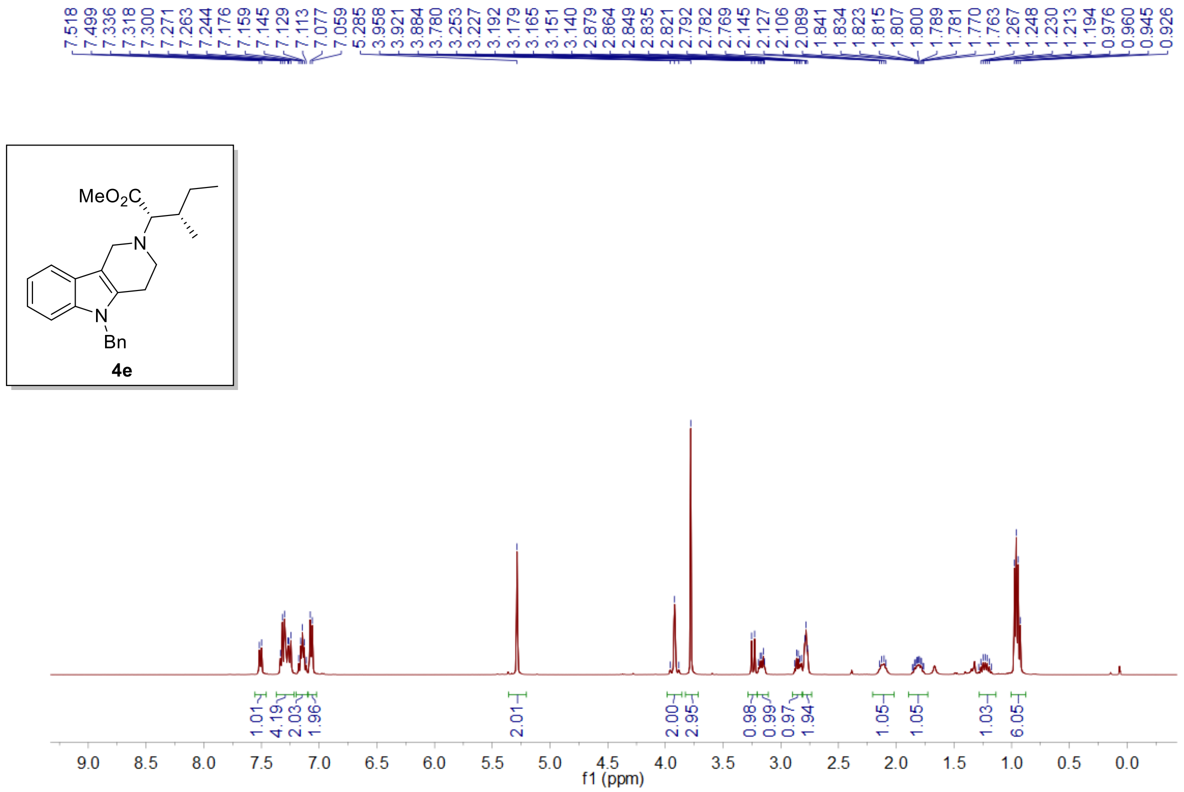

^{13}C-NMR spectrum of compound **4e** (101 MHz, $CDCl_3$)

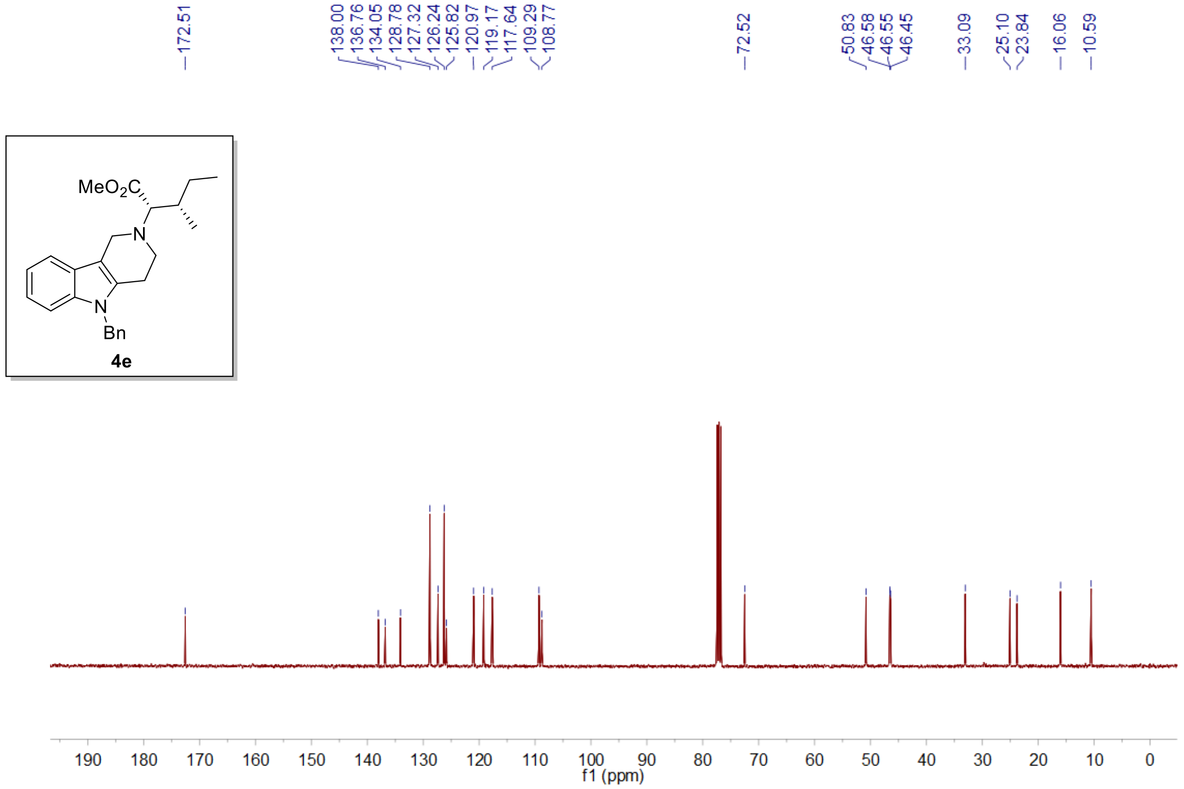

^{1}H-NMR spectrum of compound **4f** (400 MHz, $CDCl_3$)

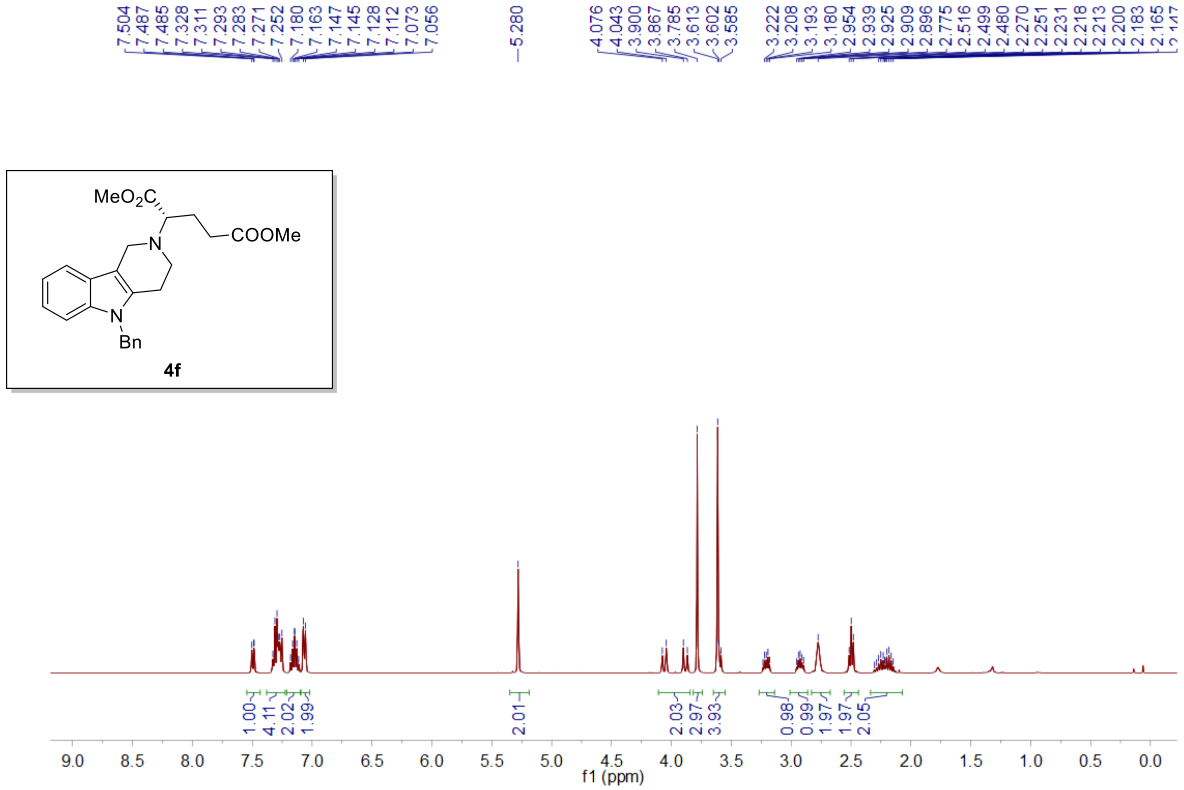

^{13}C-NMR spectrum of compound **4f** (101 MHz, $CDCl_3$)

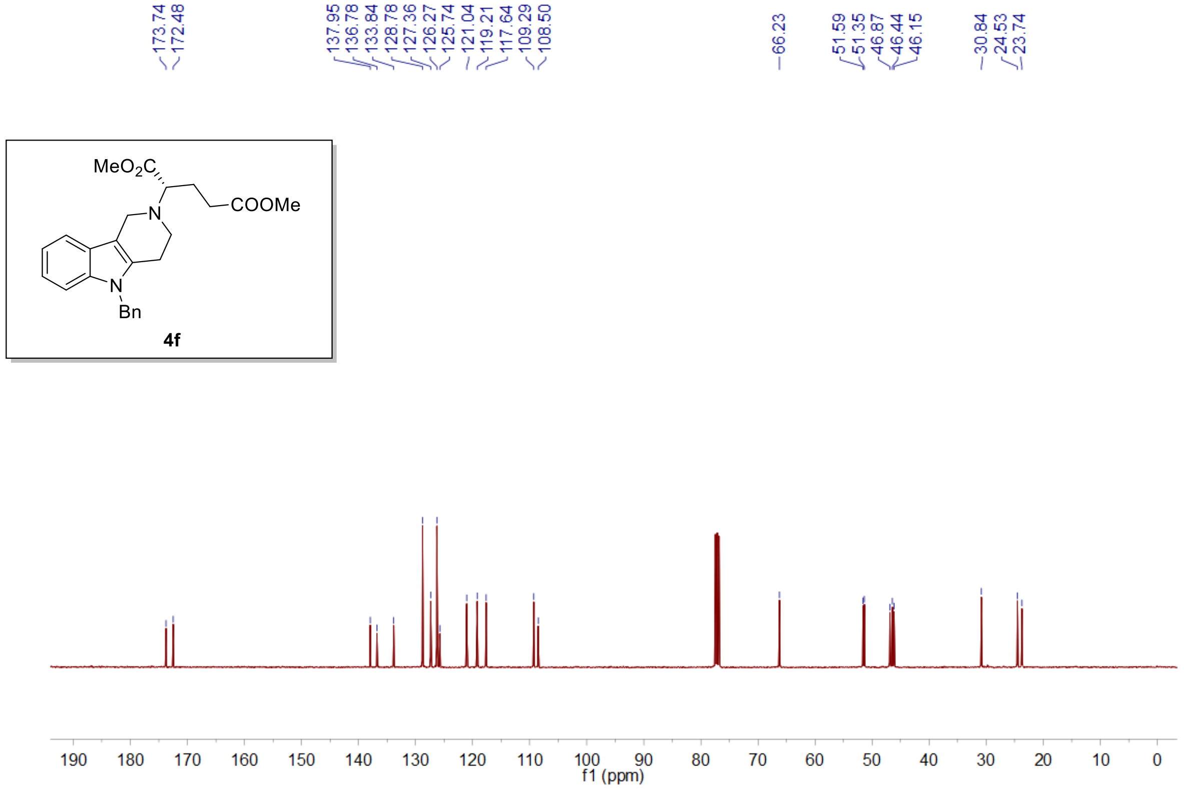

^{1}H-NMR spectrum of compound **4g** (400 MHz, $CDCl_3$)

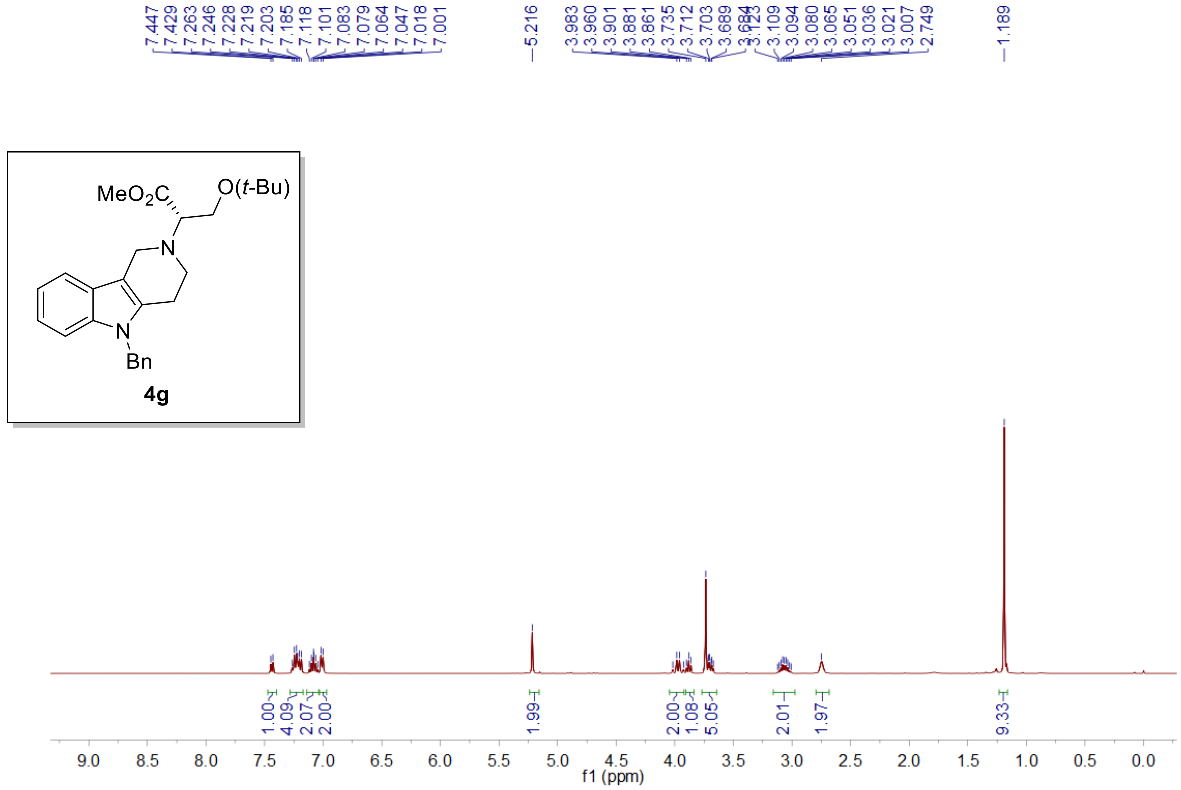

^{13}C-NMR spectrum of compound **4g** (101 MHz, $CDCl_3$)

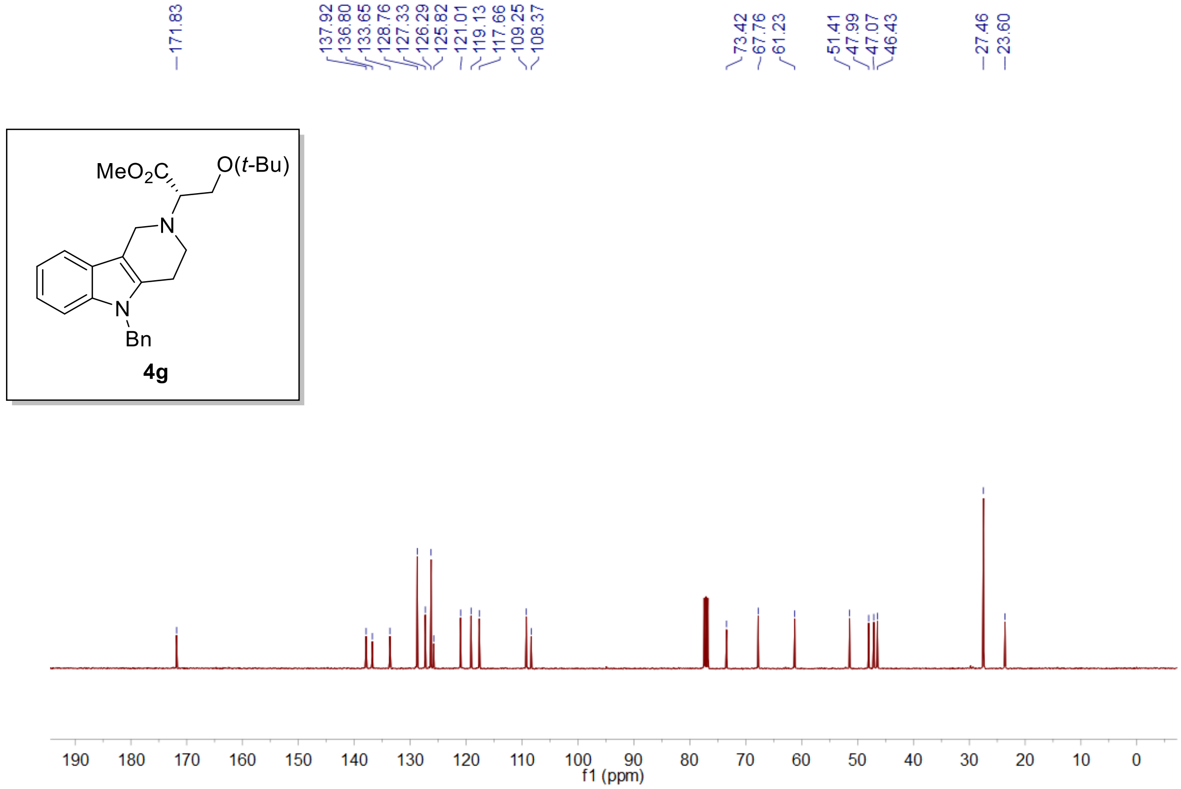

^{1}H-NMR spectrum of compound **4h** (400 MHz, $CDCl_3$)

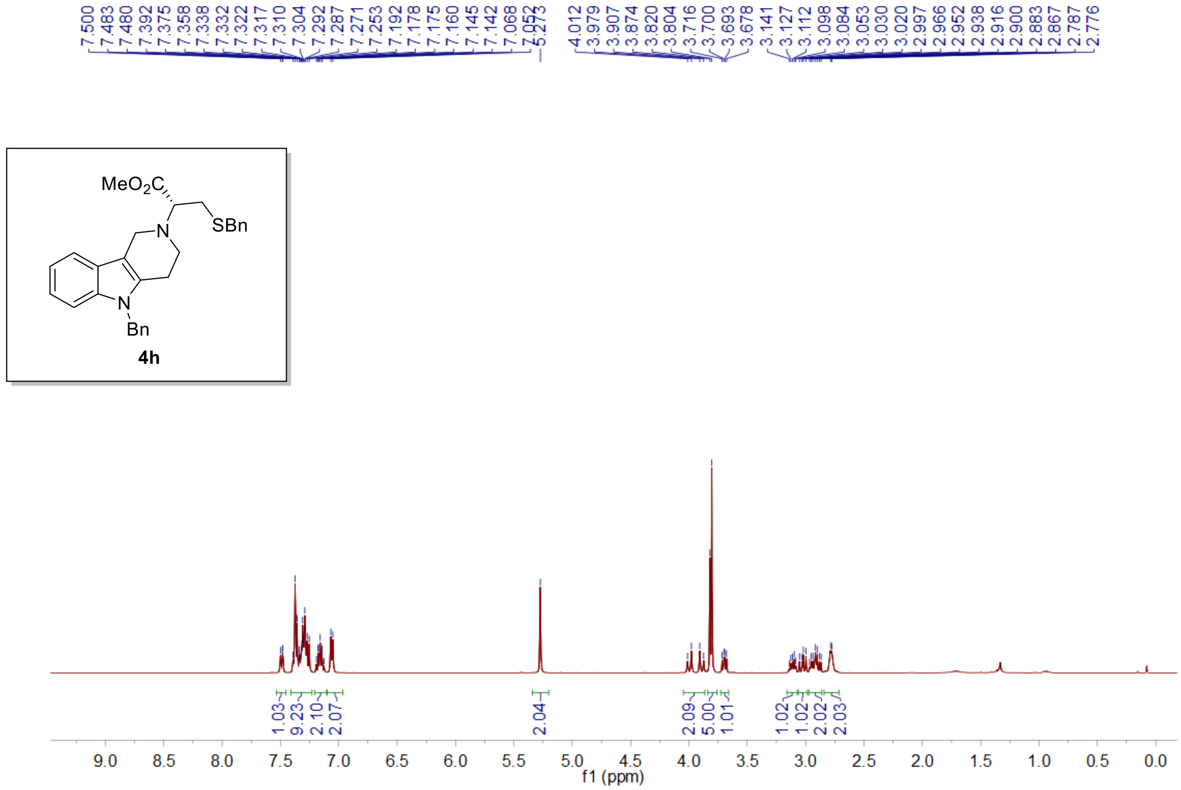

^{13}C-NMR spectrum of compound **4h** (101 MHz, $CDCl_3$)

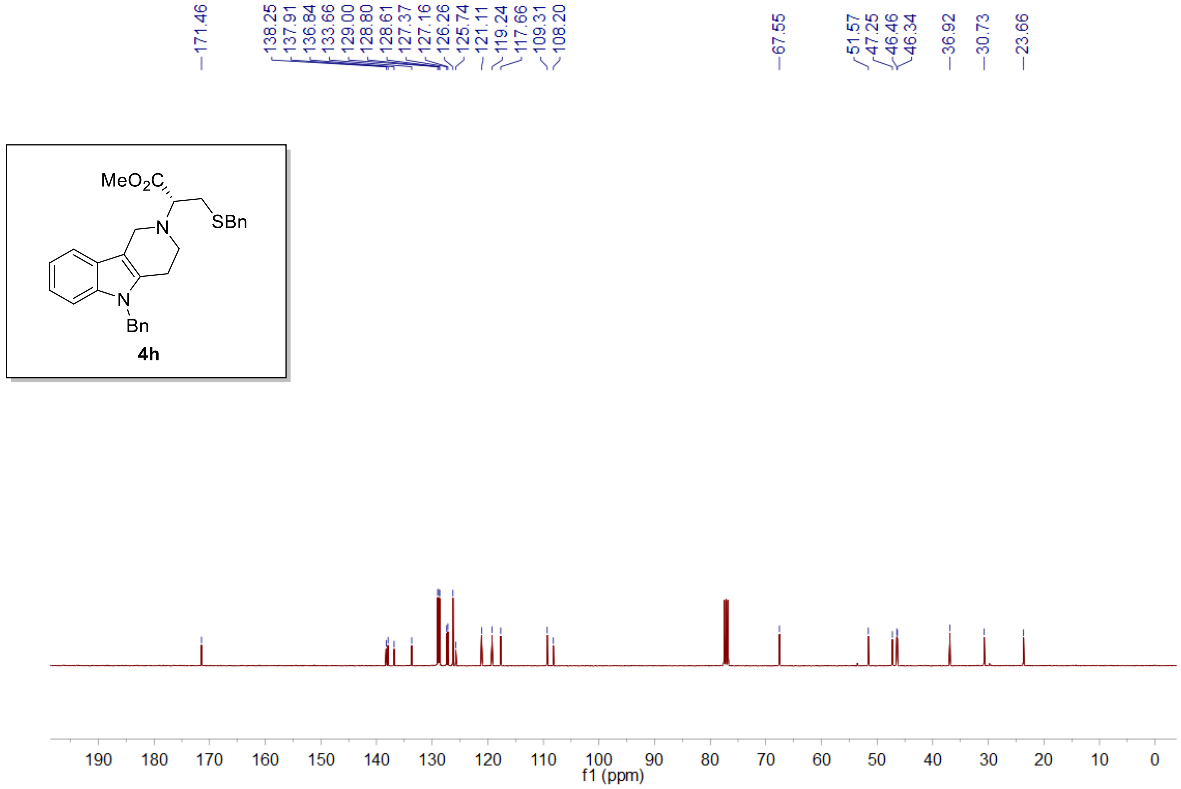

^{1}H-NMR spectrum of compound **4i** (400 MHz, $CDCl_3$)

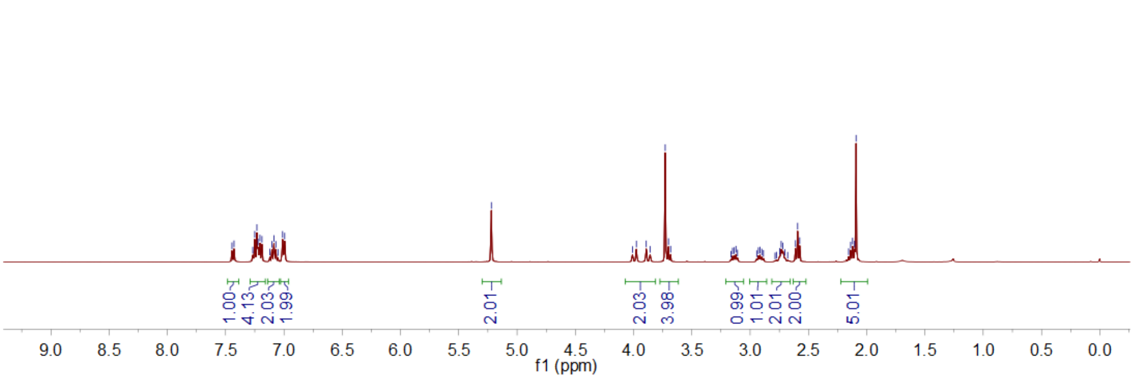

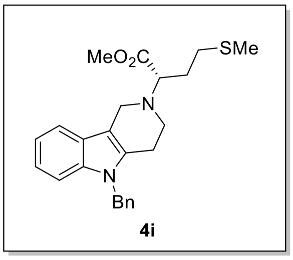

^{13}C-NMR spectrum of compound **4i** (101 MHz, $CDCl_3$)

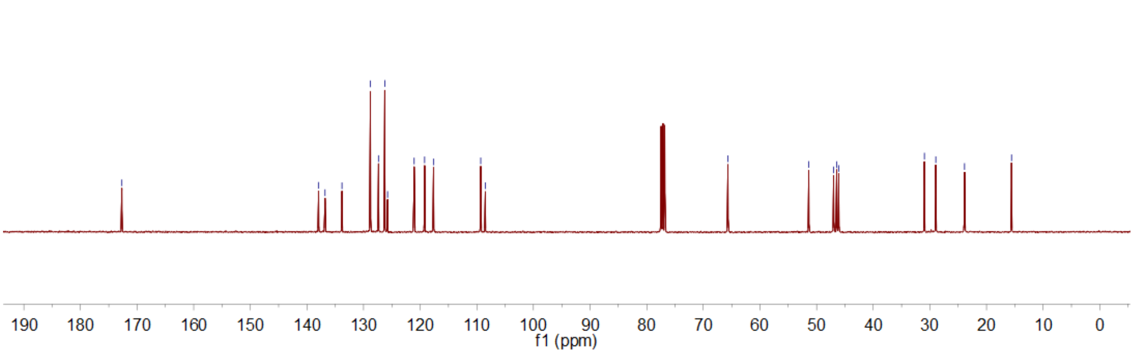

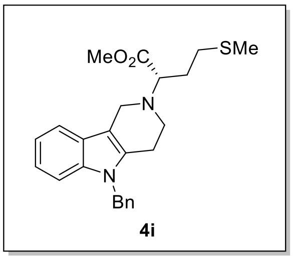

^{1}H-NMR spectrum of compound **4j** (400 MHz, $CDCl_3$)

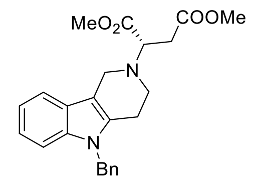

4j

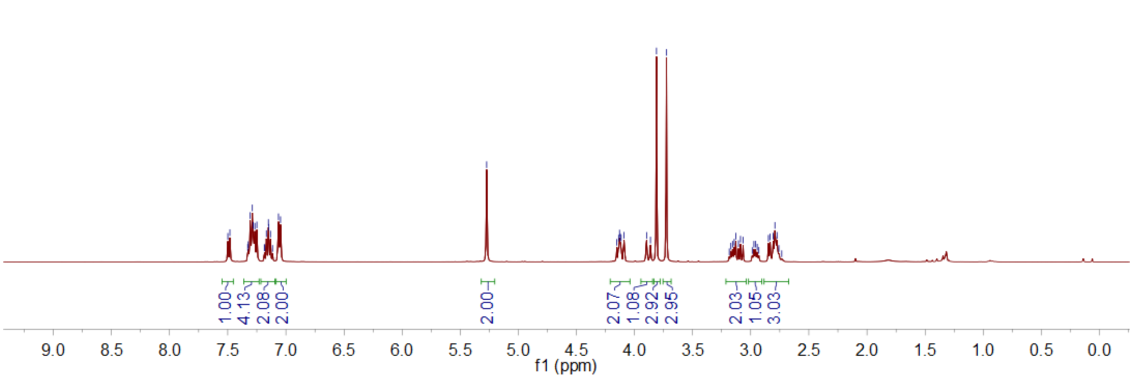

^{13}C-NMR spectrum of compound **4j** (101 MHz, $CDCl_3$)

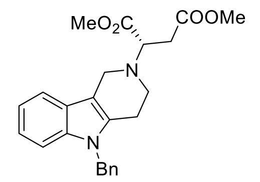

4j

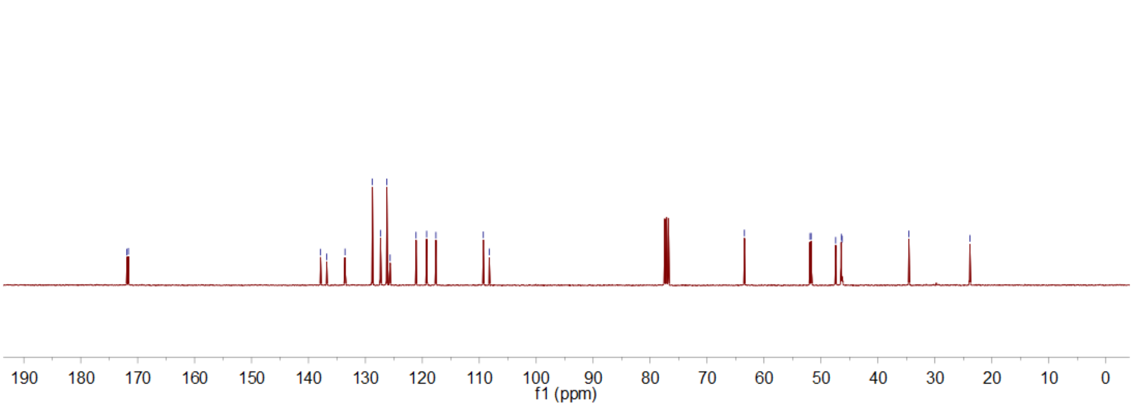

^{1}H-NMR spectrum of compound **4k** (400 MHz, $CDCl_3$)

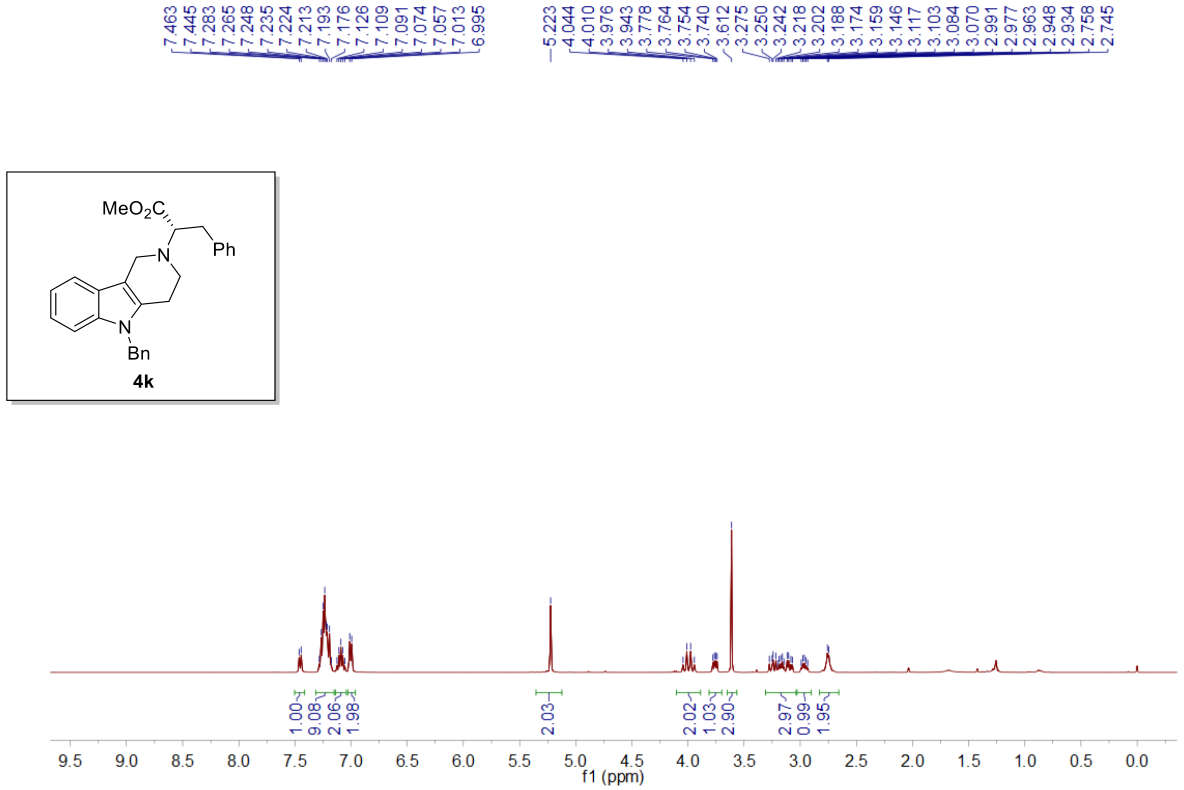

^{13}C-NMR spectrum of compound **4k** (101 MHz, $CDCl_3$)

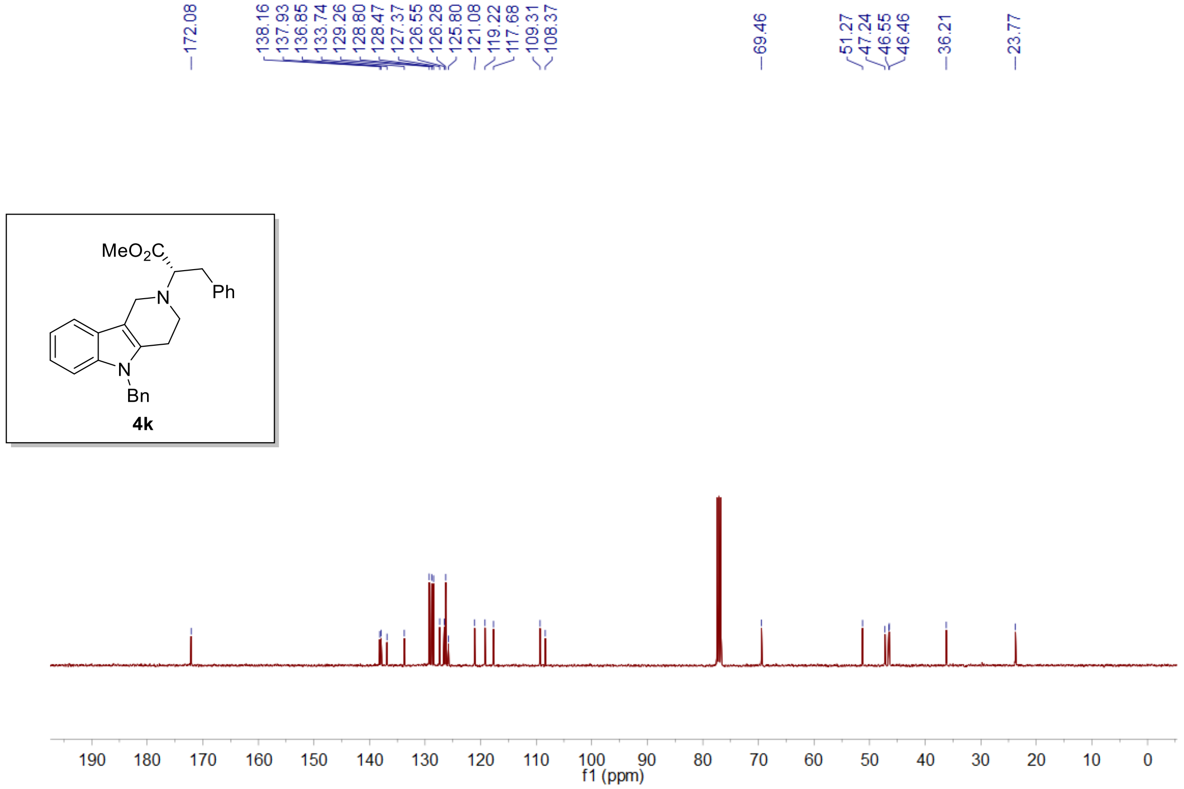

^{1}H-NMR spectrum of compound **4l** (400 MHz, $CDCl_3$)

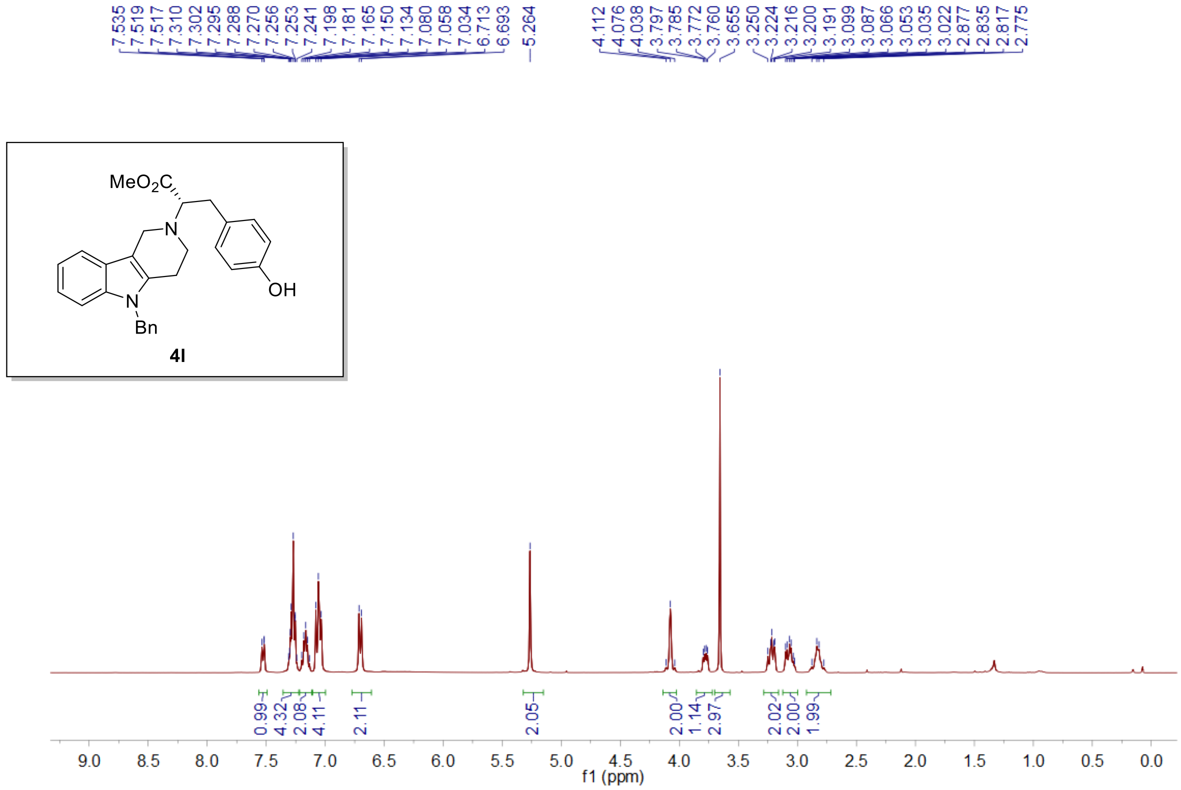

^{13}C-NMR spectrum of compound **4l** (101 MHz, $CDCl_3$)

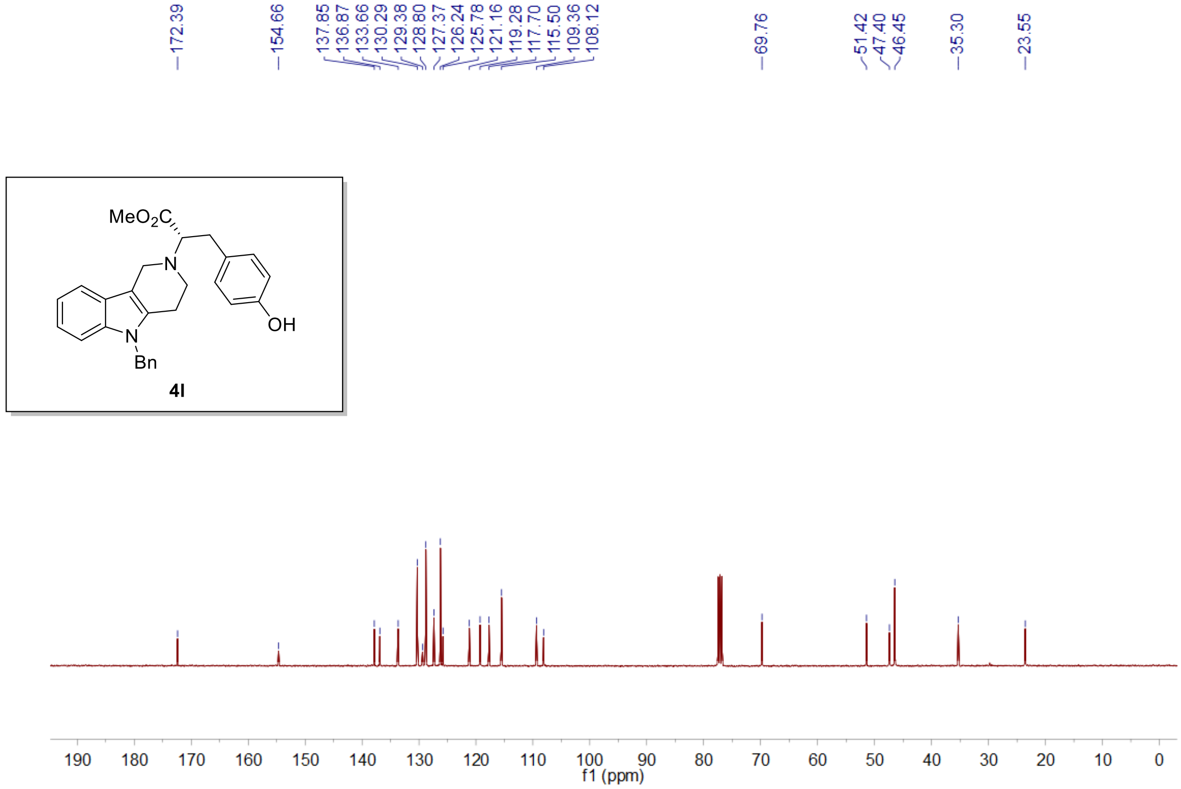

^{1}H-NMR spectrum of compound **4m** (400 MHz, $CDCl_3$)

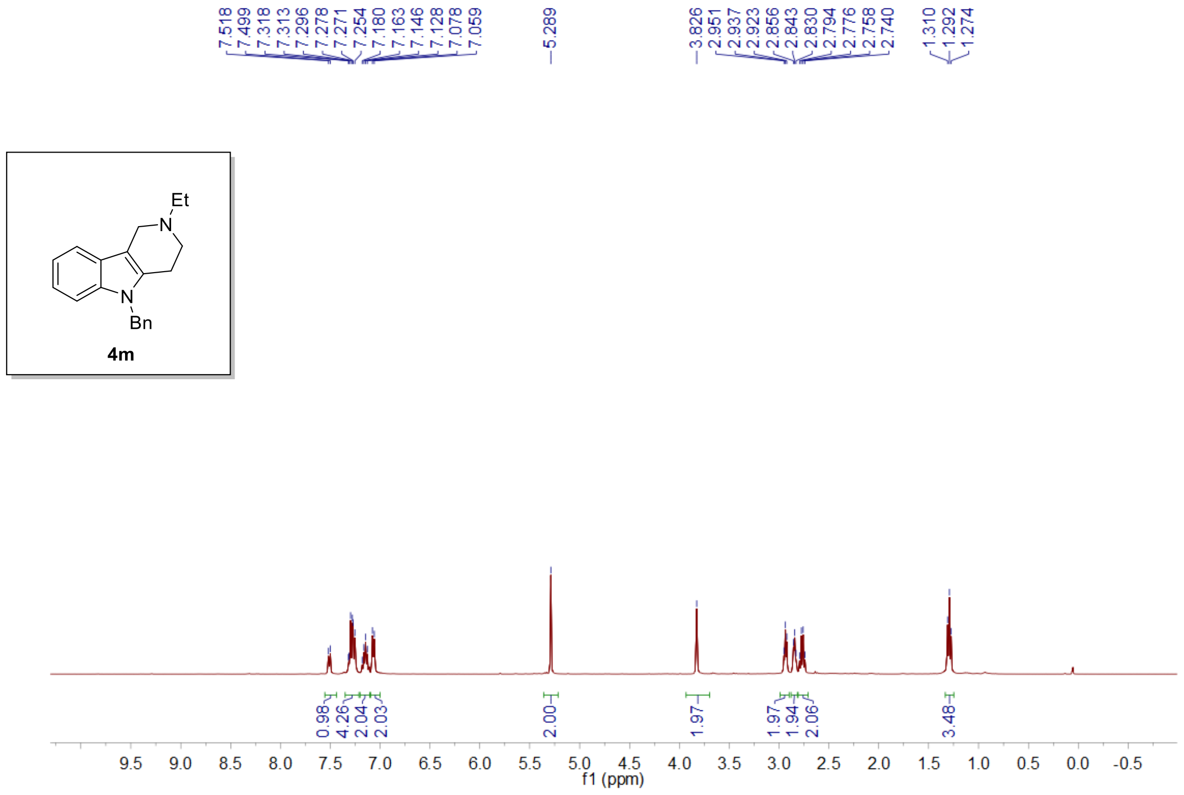

^{13}C-NMR spectrum of compound **4m** (101 MHz, $CDCl_3$)

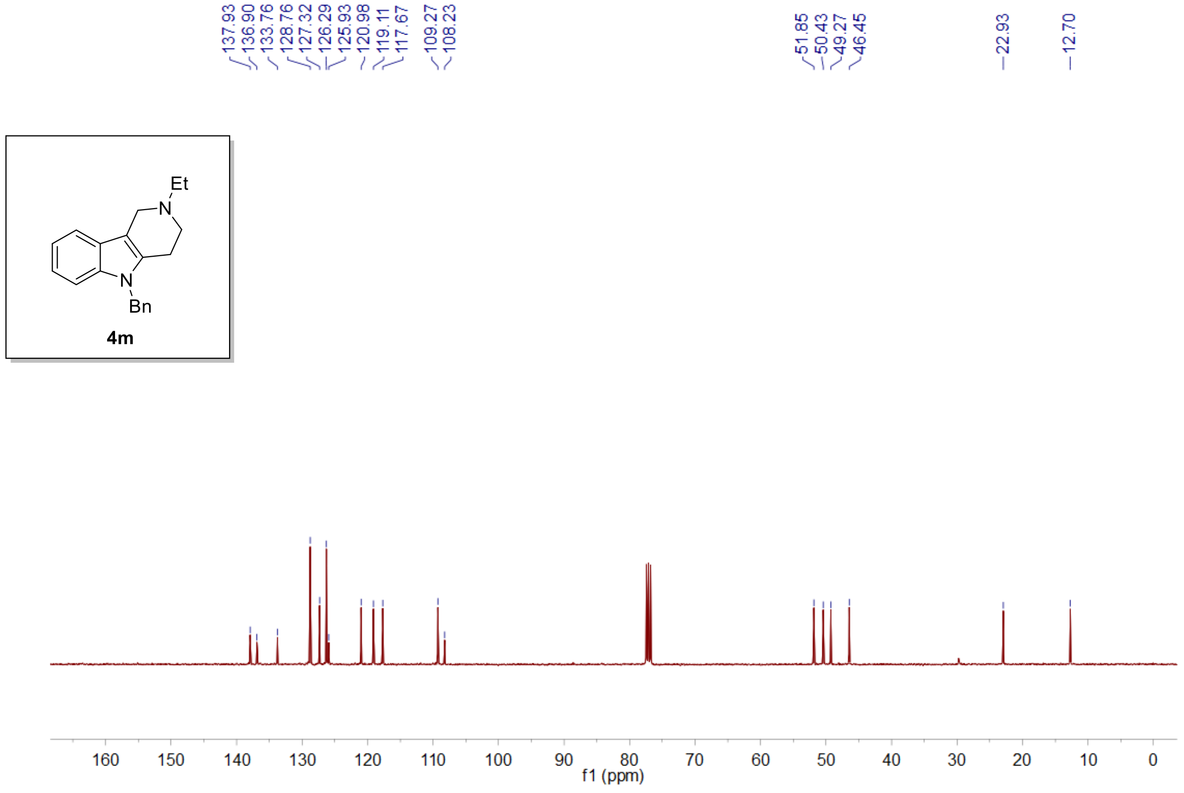

^{1}H-NMR spectrum of compound **4n** (400 MHz, $CDCl_3$)

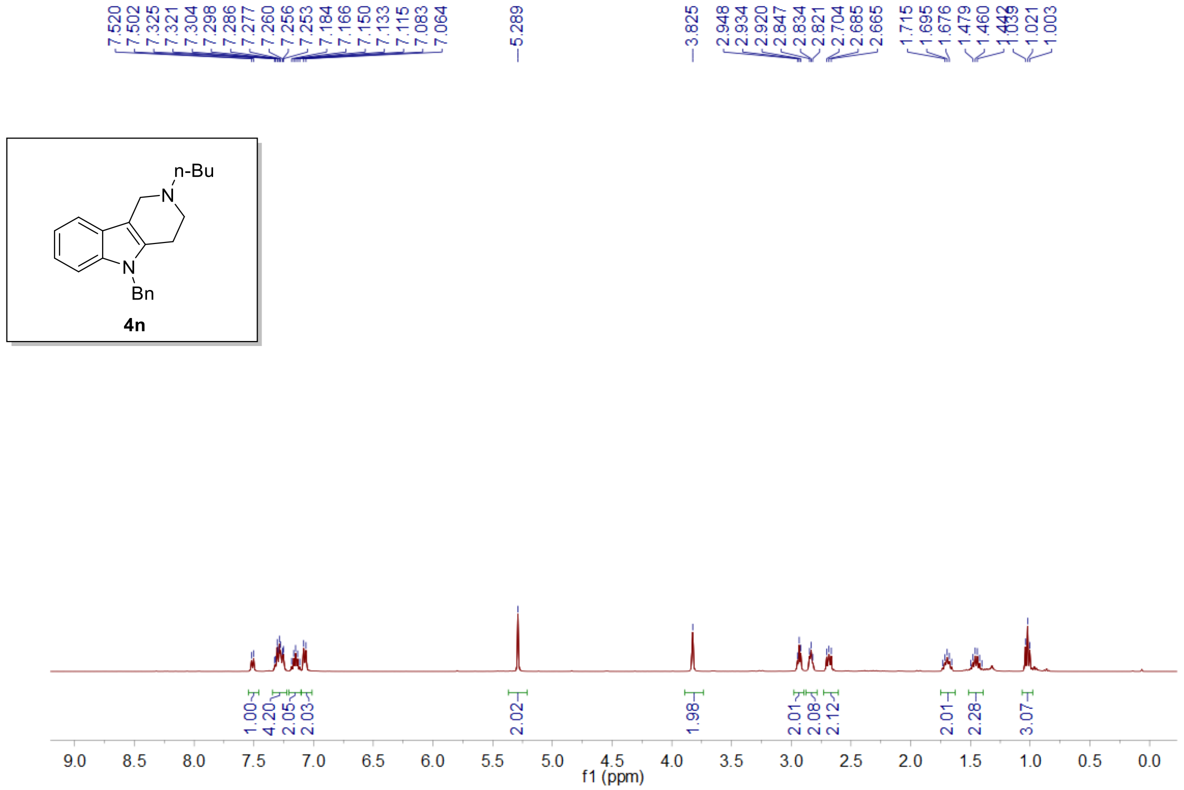

^{13}C-NMR spectrum of compound **4n** (101 MHz, $CDCl_3$)

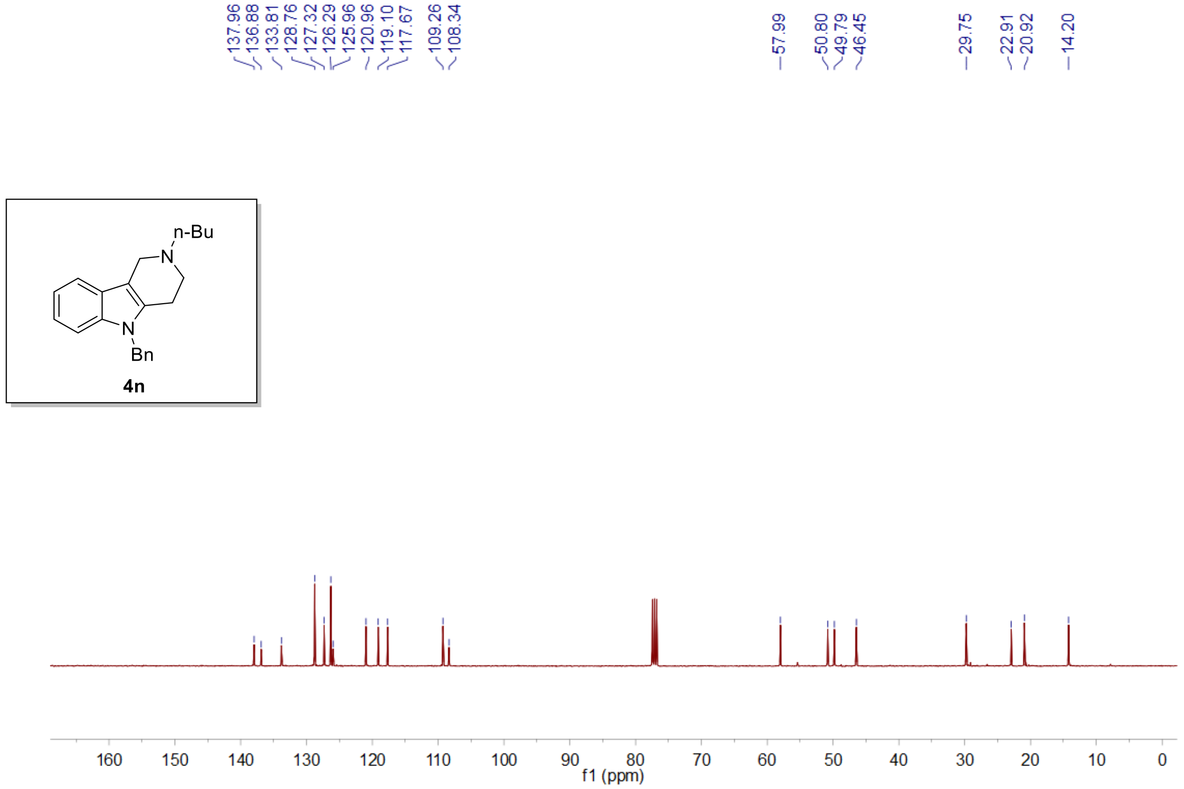

^{1}H-NMR spectrum of compound **4o** (400 MHz, $CDCl_3$)

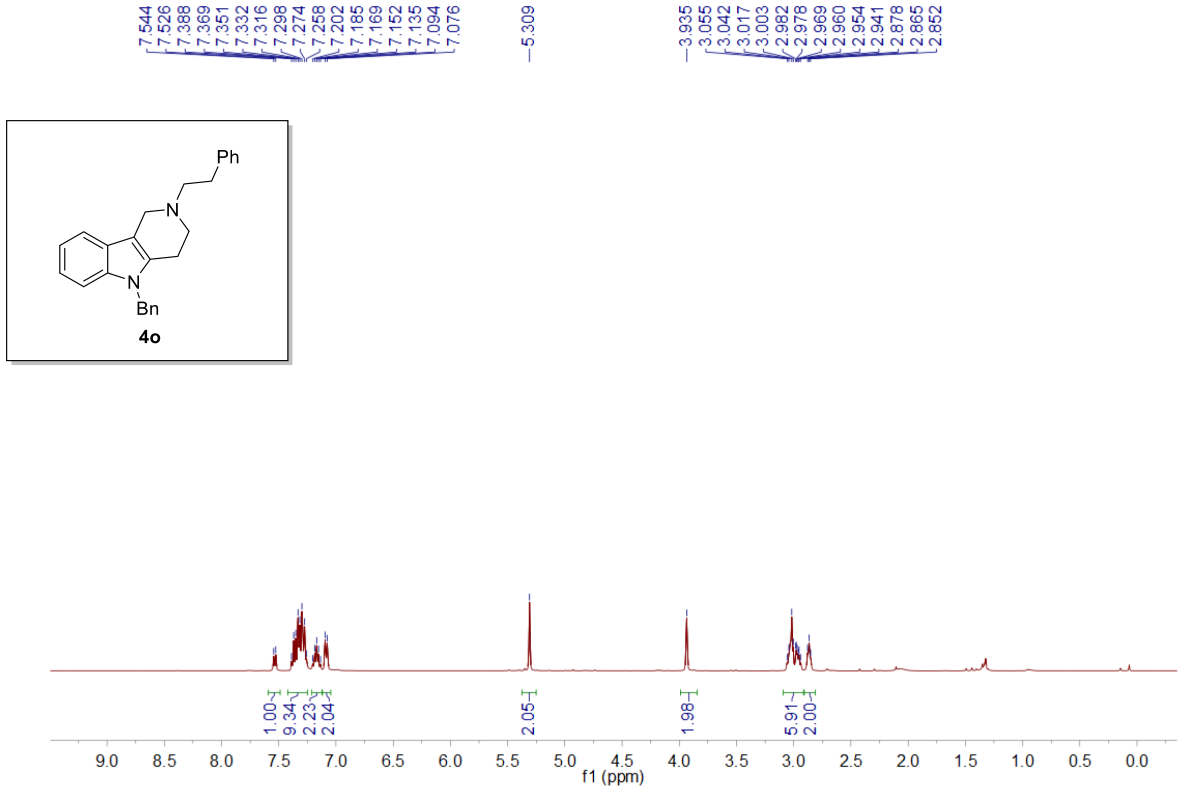

^{13}C-NMR spectrum of compound **4o** (101 MHz, $CDCl_3$)

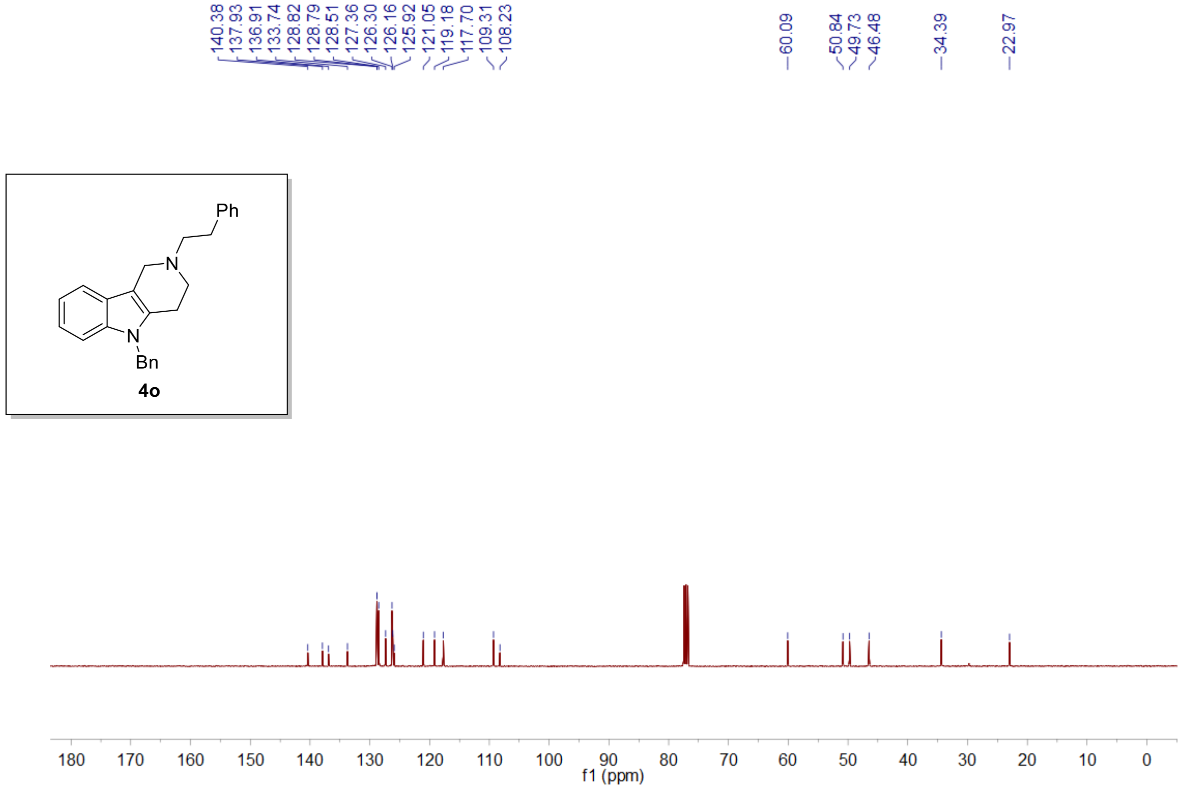

^{1}H-NMR spectrum of compound **4p** (400 MHz, $CDCl_3$)

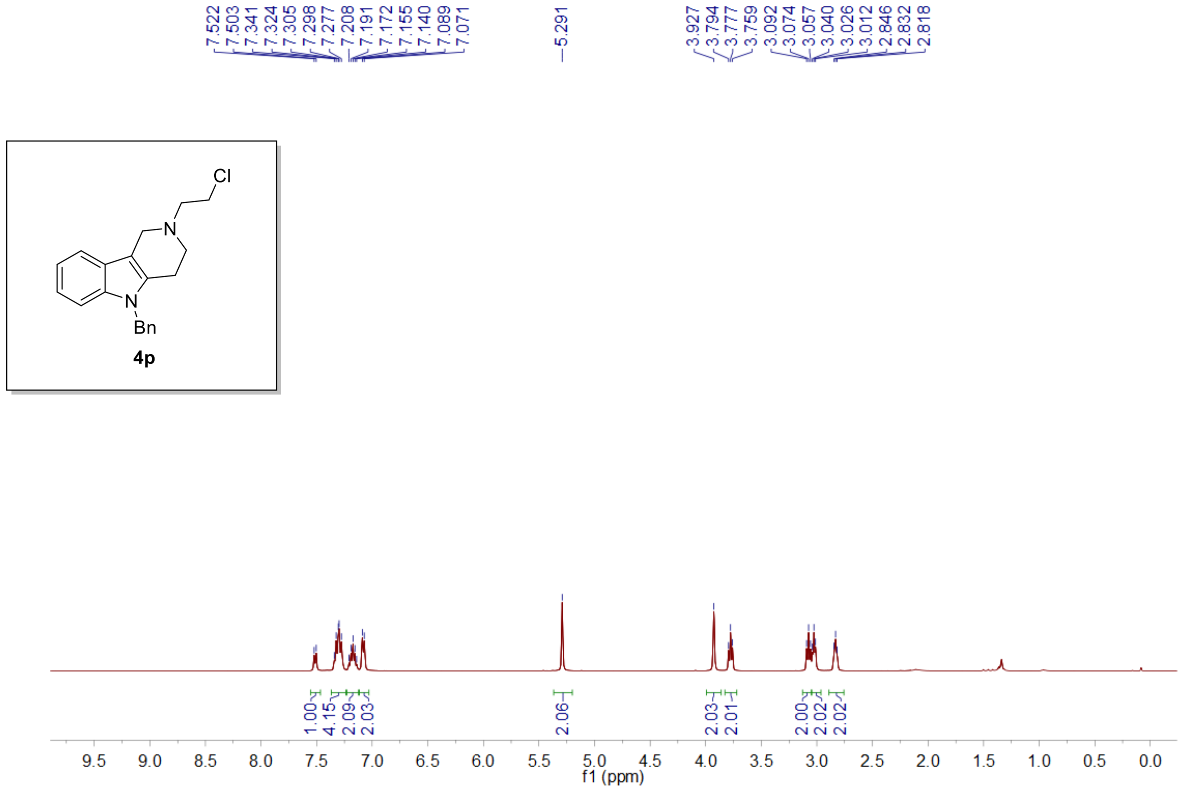

^{13}C-NMR spectrum of compound **4p** (101 MHz, $CDCl_3$)

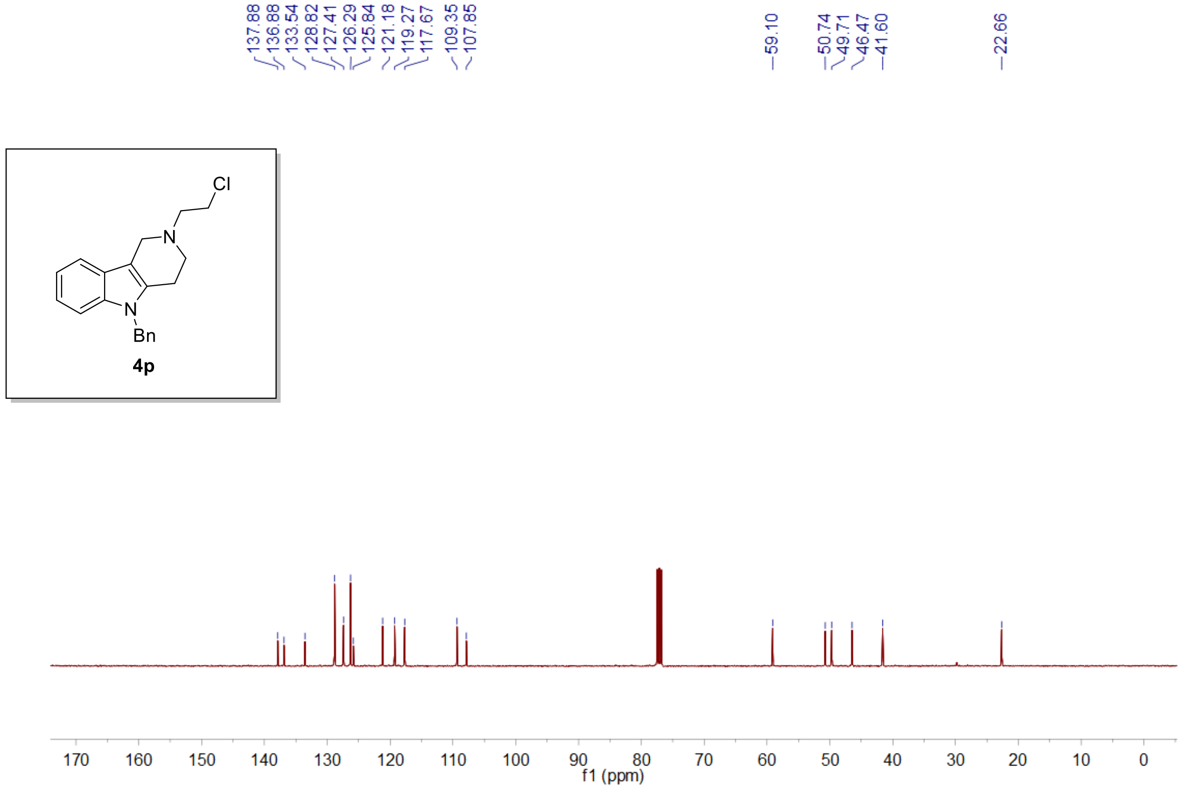

^{1}H-NMR spectrum of compound **4q** (400 MHz, $CDCl_3$)

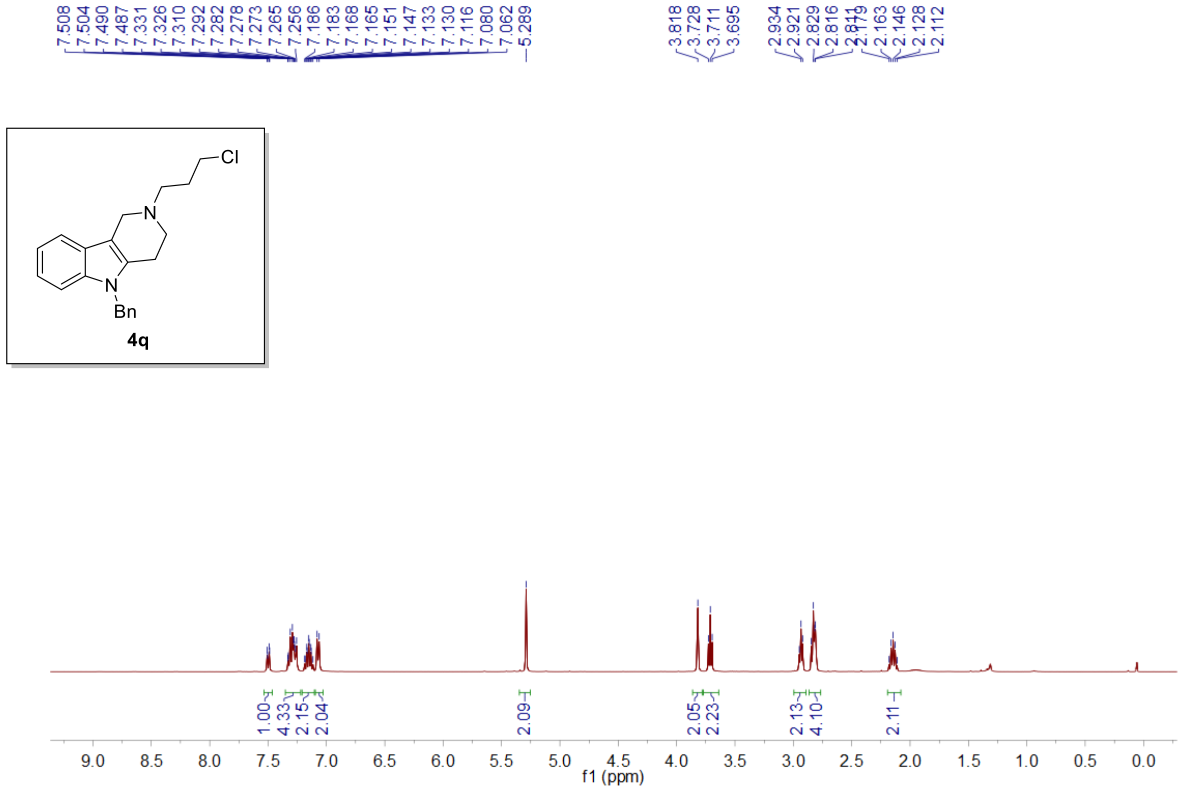

^{13}C-NMR spectrum of compound **4q** (101 MHz, $CDCl_3$)

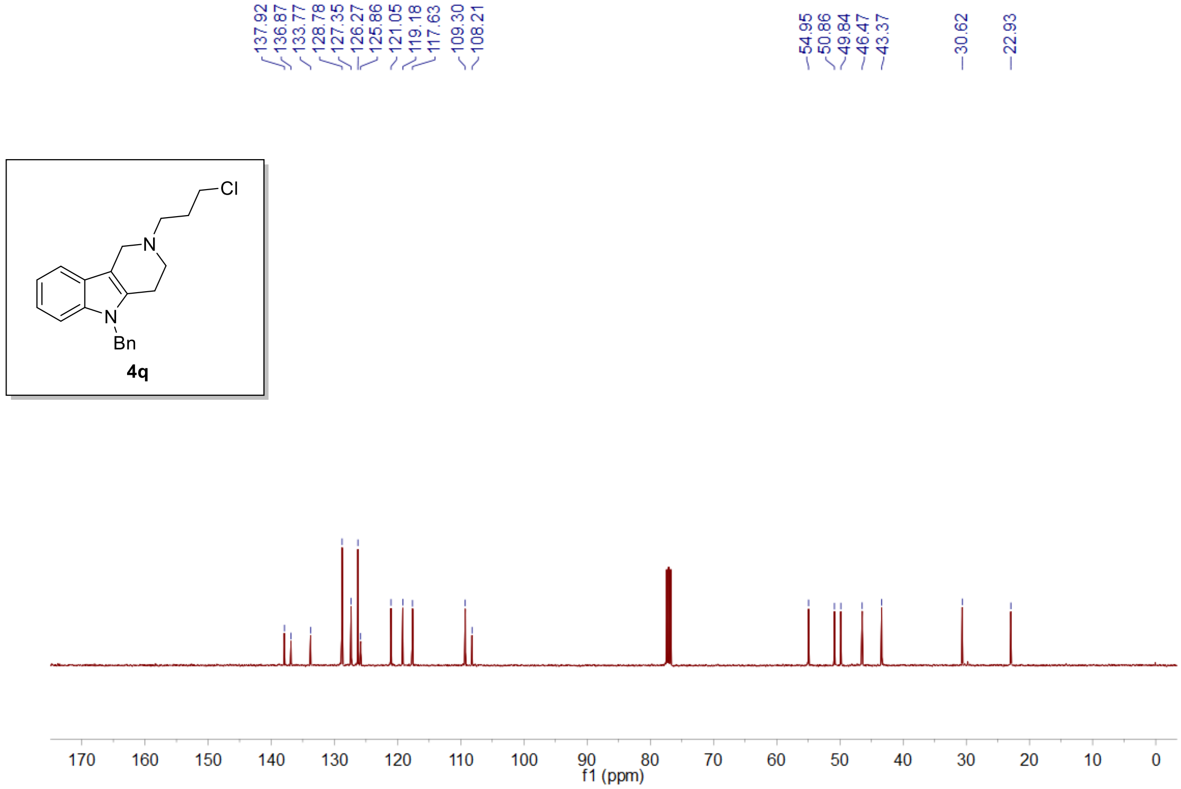

^{1}H-NMR spectrum of compound **4r** (400 MHz, DMSO-d_6)

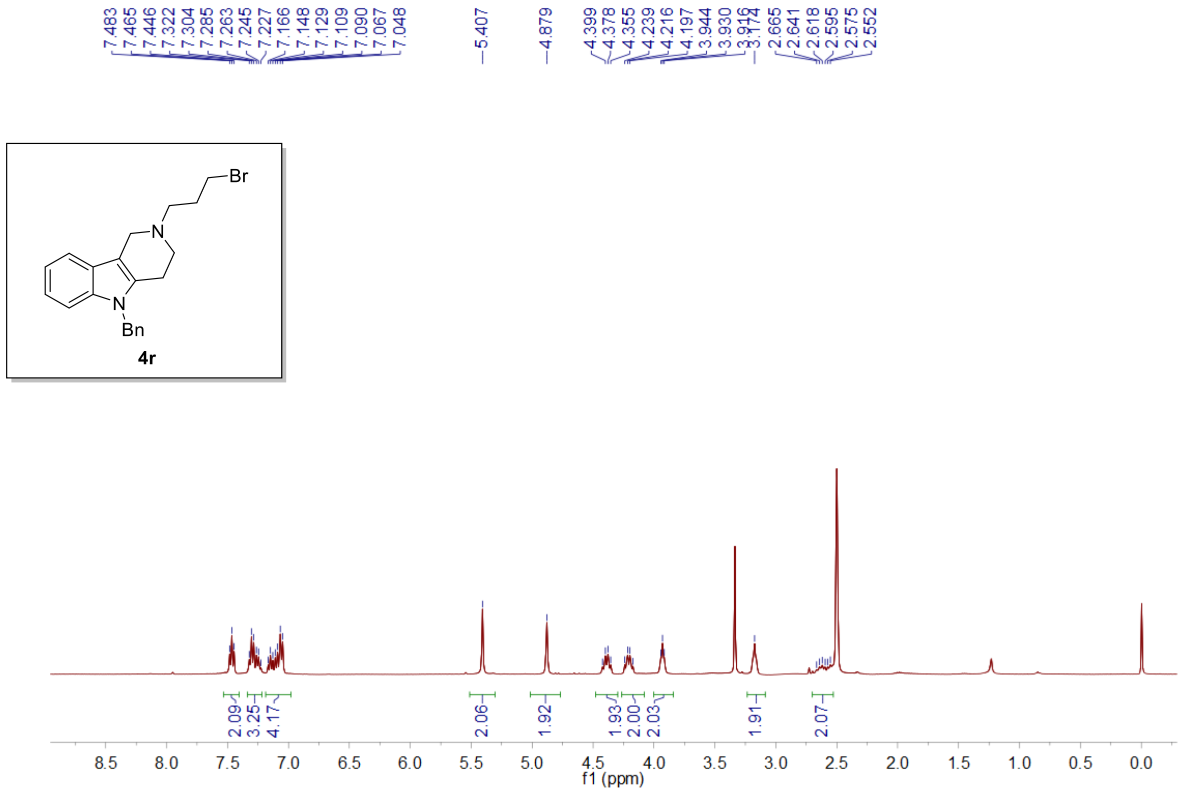

^{13}C-NMR spectrum of compound **4r** (101 MHz, DMSO-d_6)

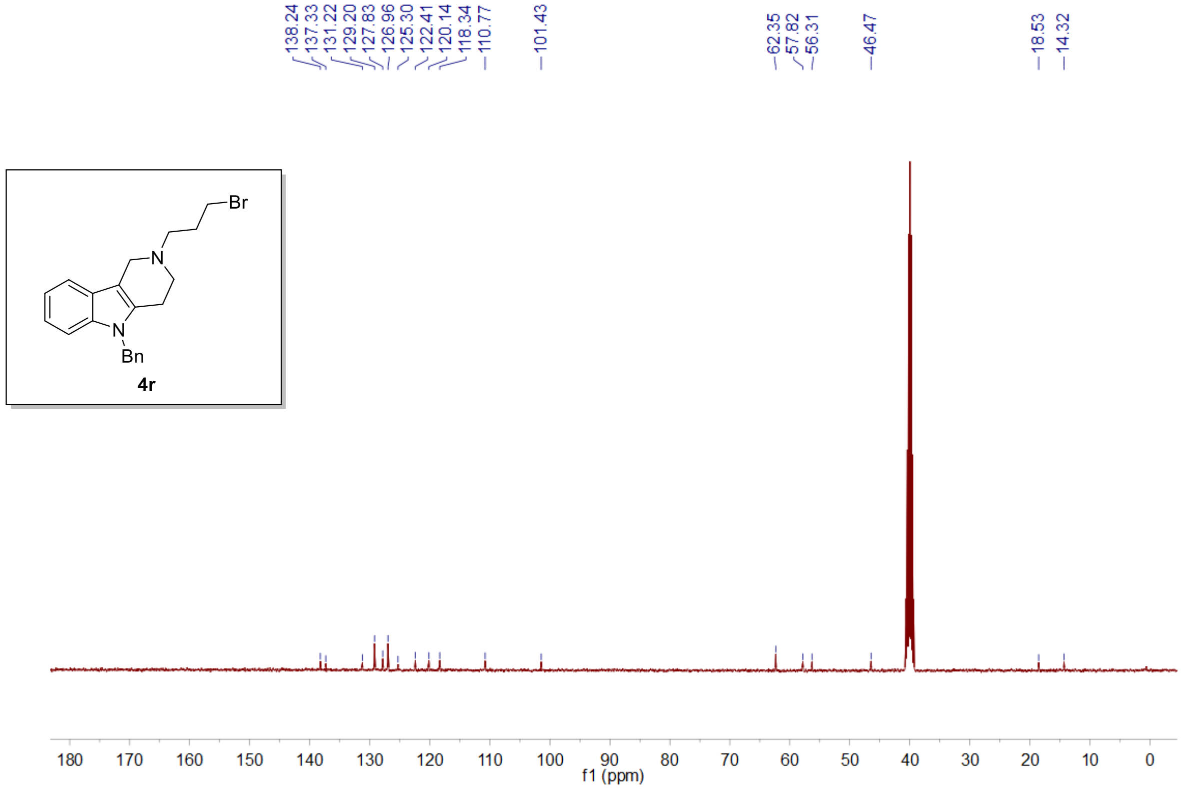

^{1}H-NMR spectrum of compound **4s** (400 MHz, $CDCl_3$)

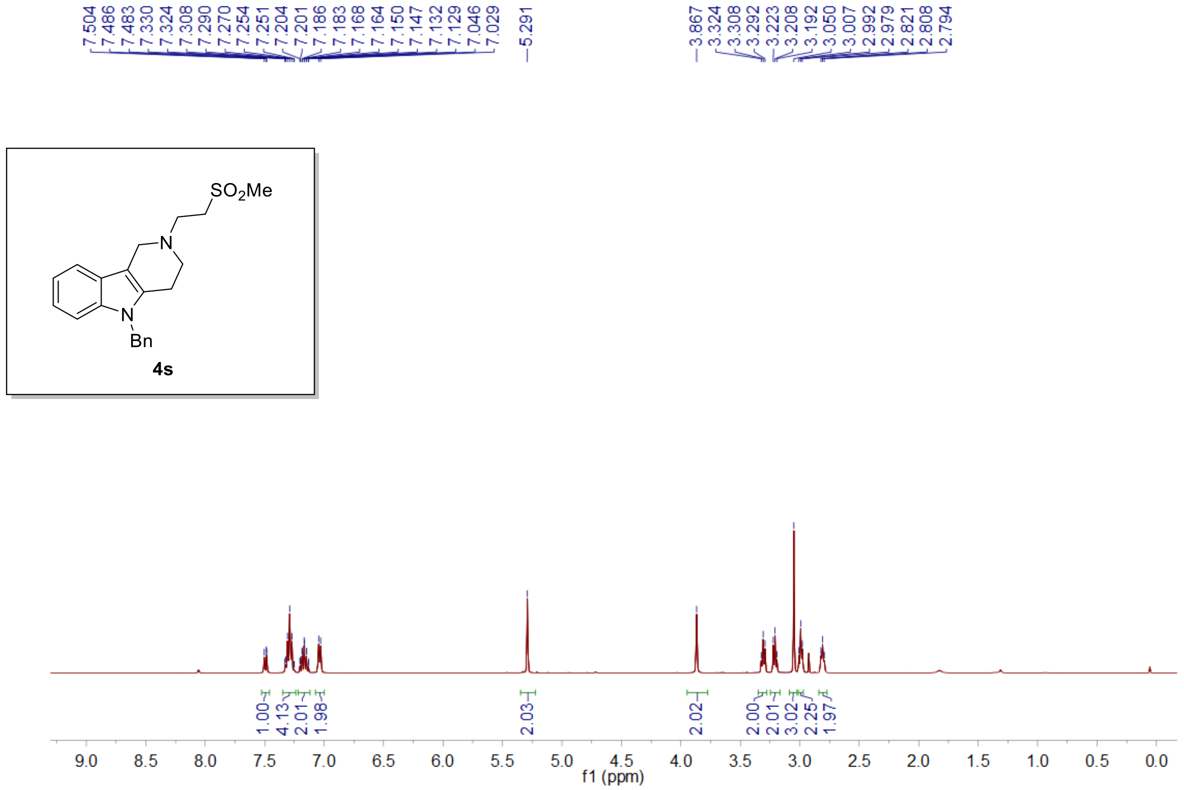

^{13}C-NMR spectrum of compound **4s** (101 MHz, $CDCl_3$)

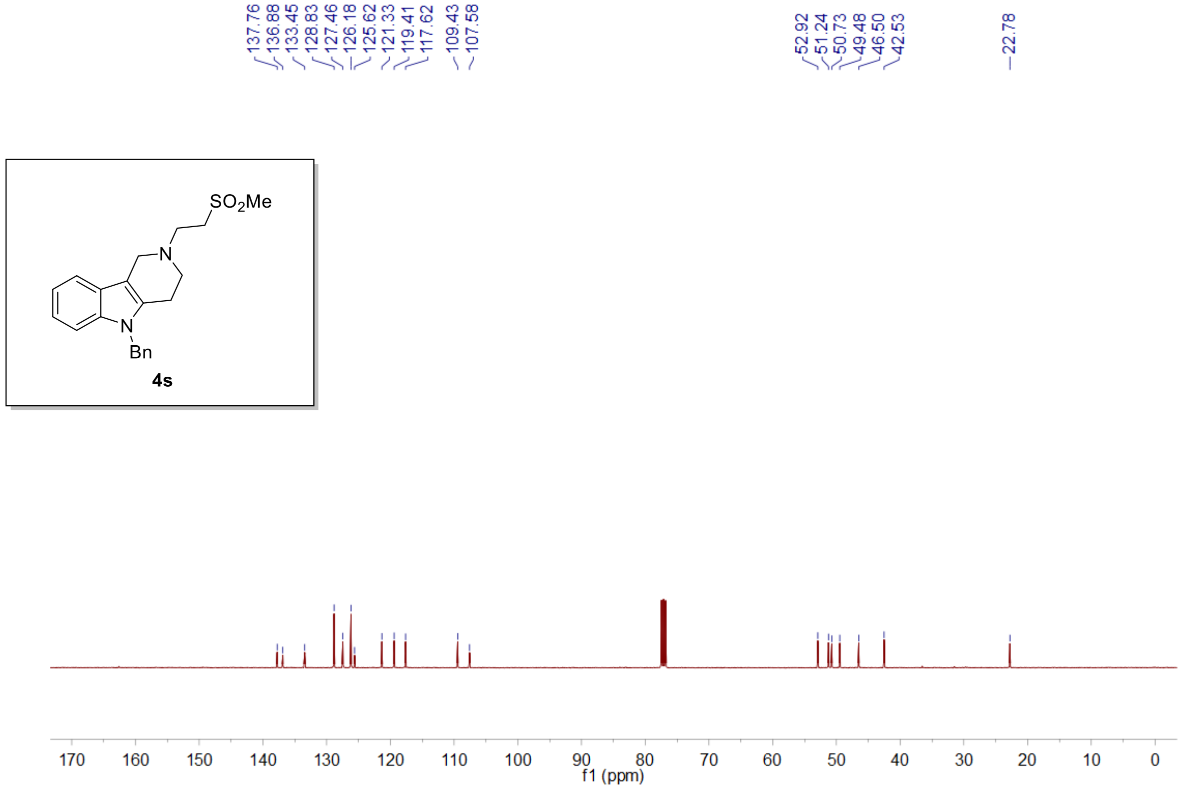

^{1}H-NMR spectrum of compound **4t** (400 MHz, $CDCl_3$)

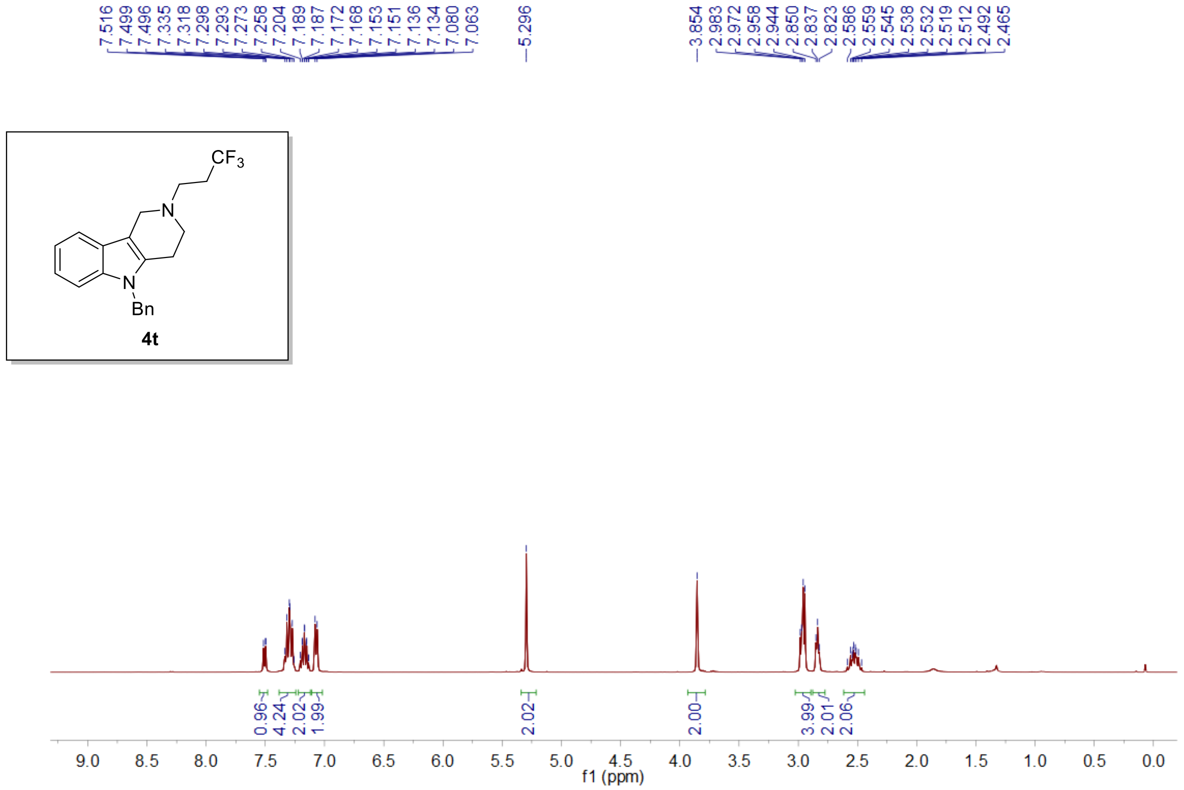

^{13}C-NMR spectrum of compound **4t** (101 MHz, $CDCl_3$)

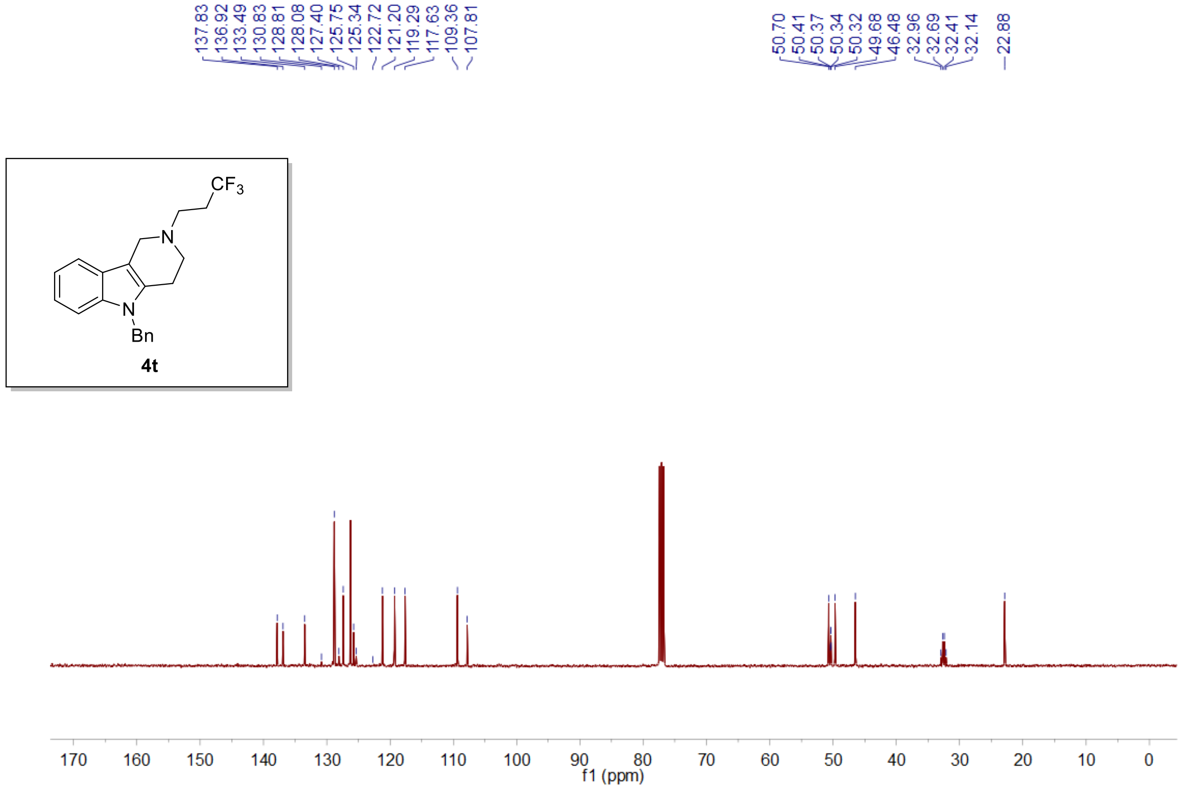

^{19}F-NMR spectrum of compound **4t** (565 MHz, $CDCl_3$)

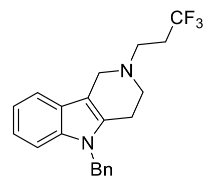

4t

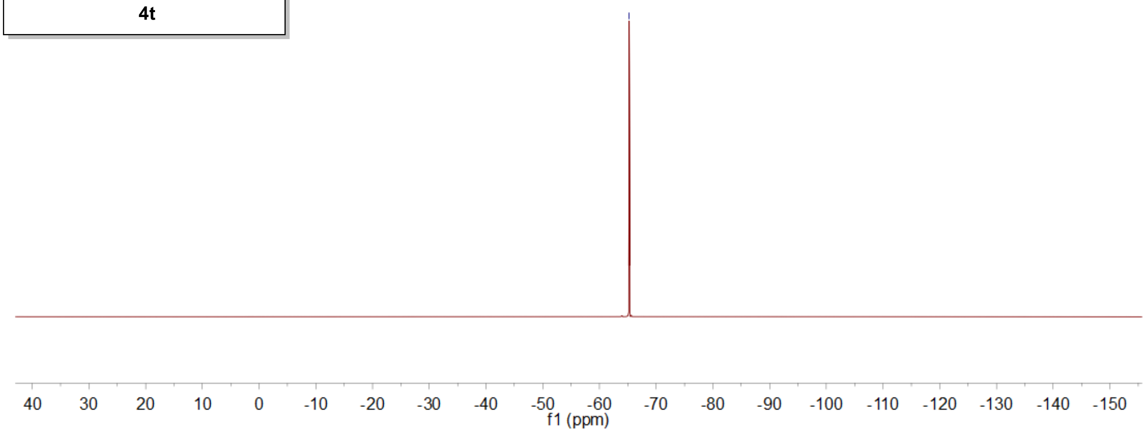

^{1}H-NMR spectrum of compound **4u** (400 MHz, $CDCl_3$)

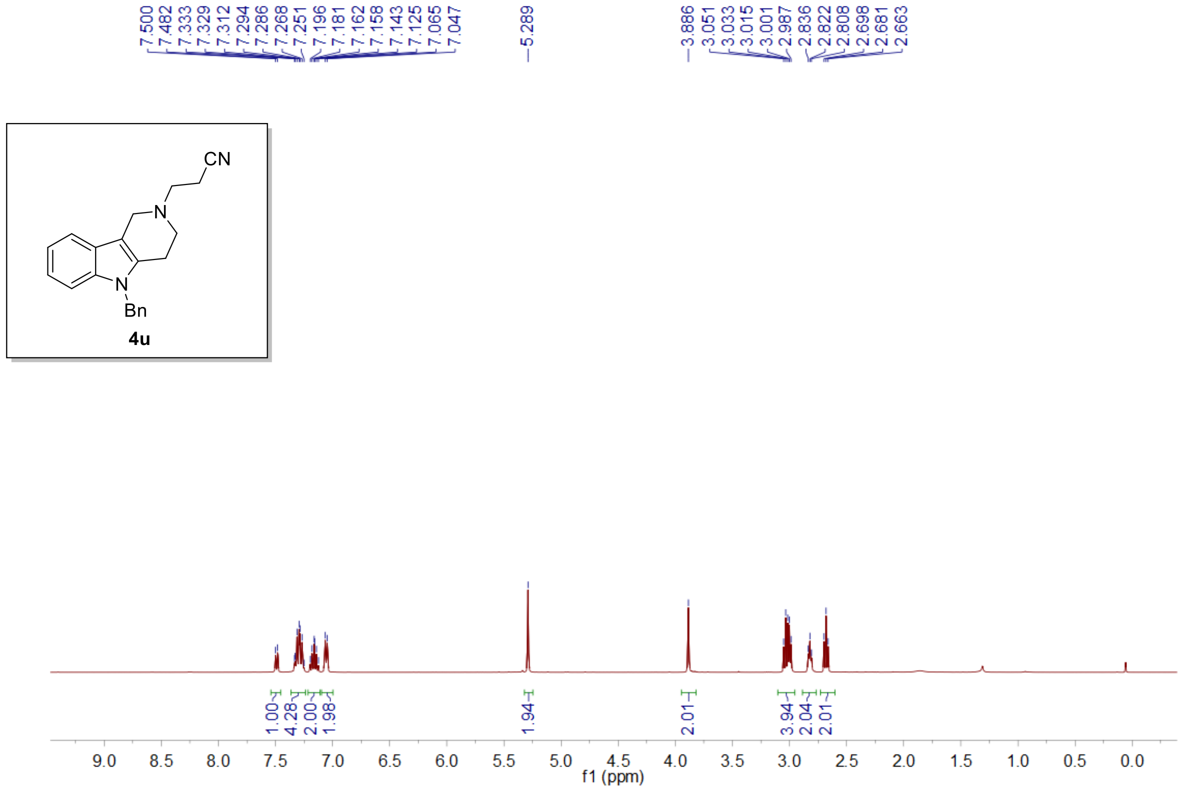

^{13}C-NMR spectrum of compound **4u** (101 MHz, $CDCl_3$)

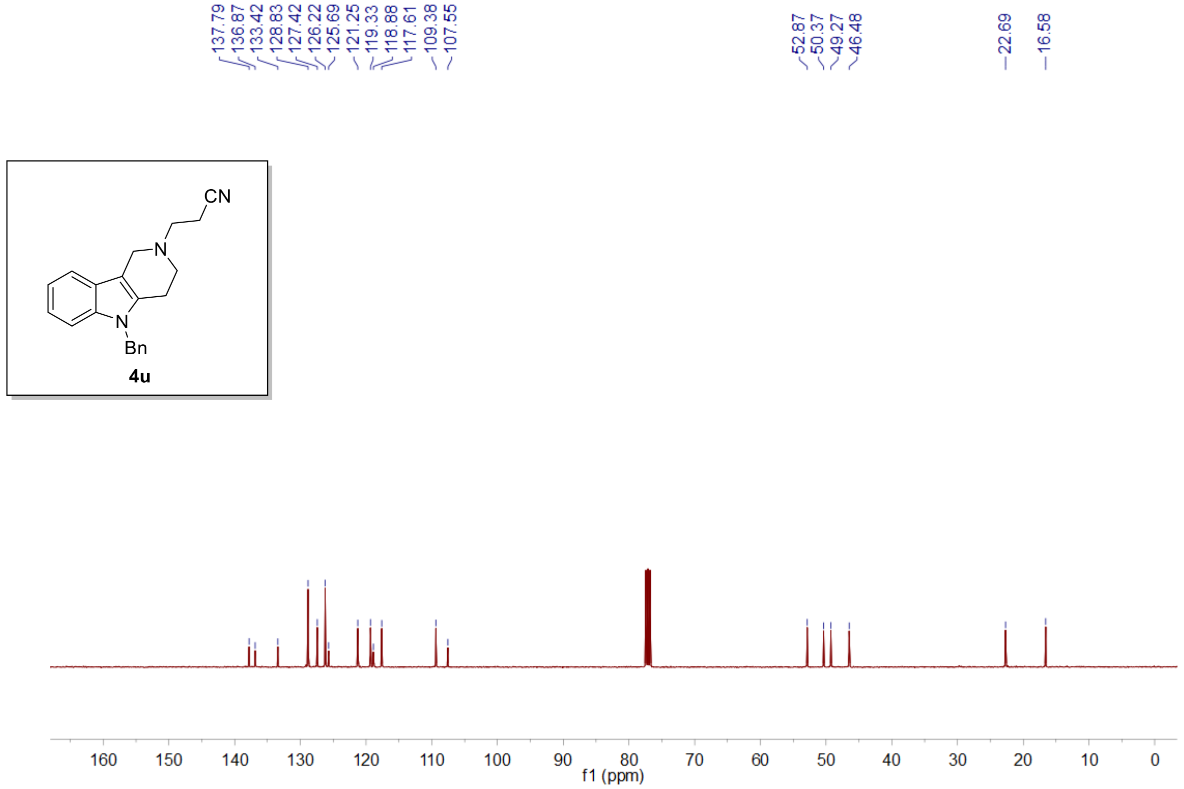

^{1}H-NMR spectrum of compound **4v** (400 MHz, $CDCl_3$)

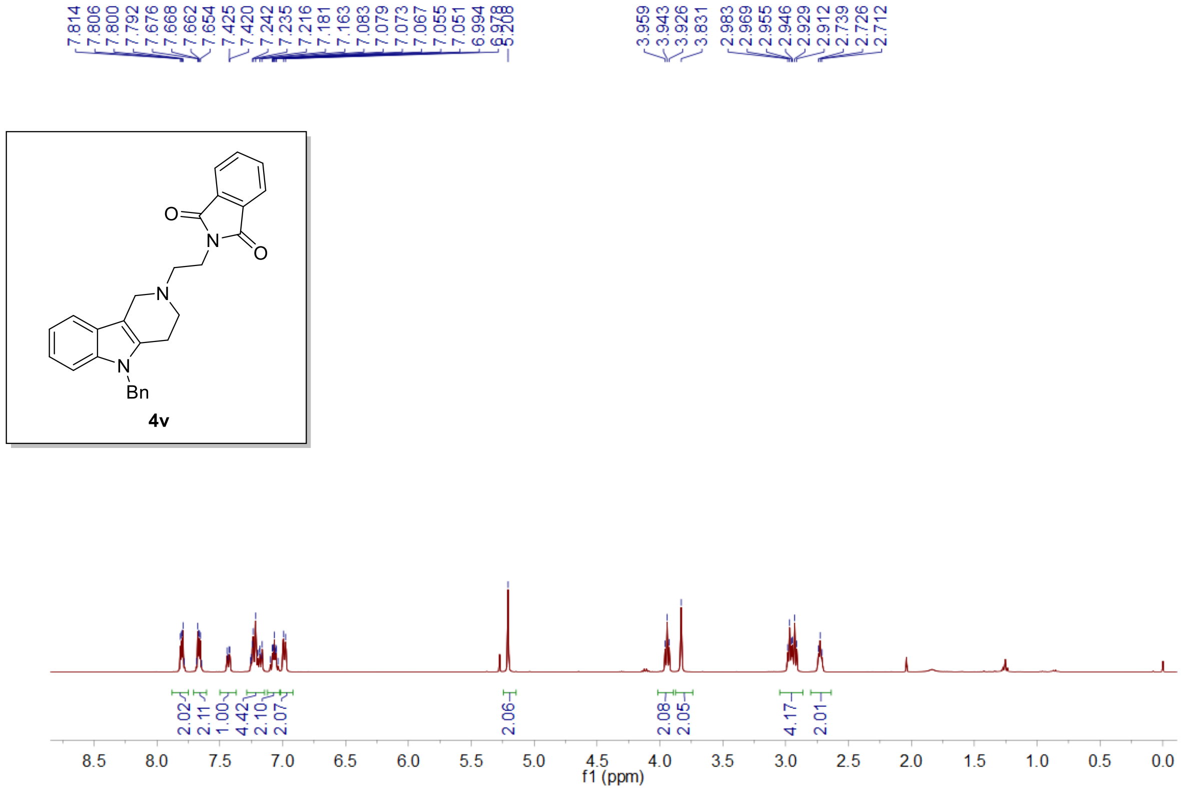

^{13}C-NMR spectrum of compound **4v** (101 MHz, $CDCl_3$)

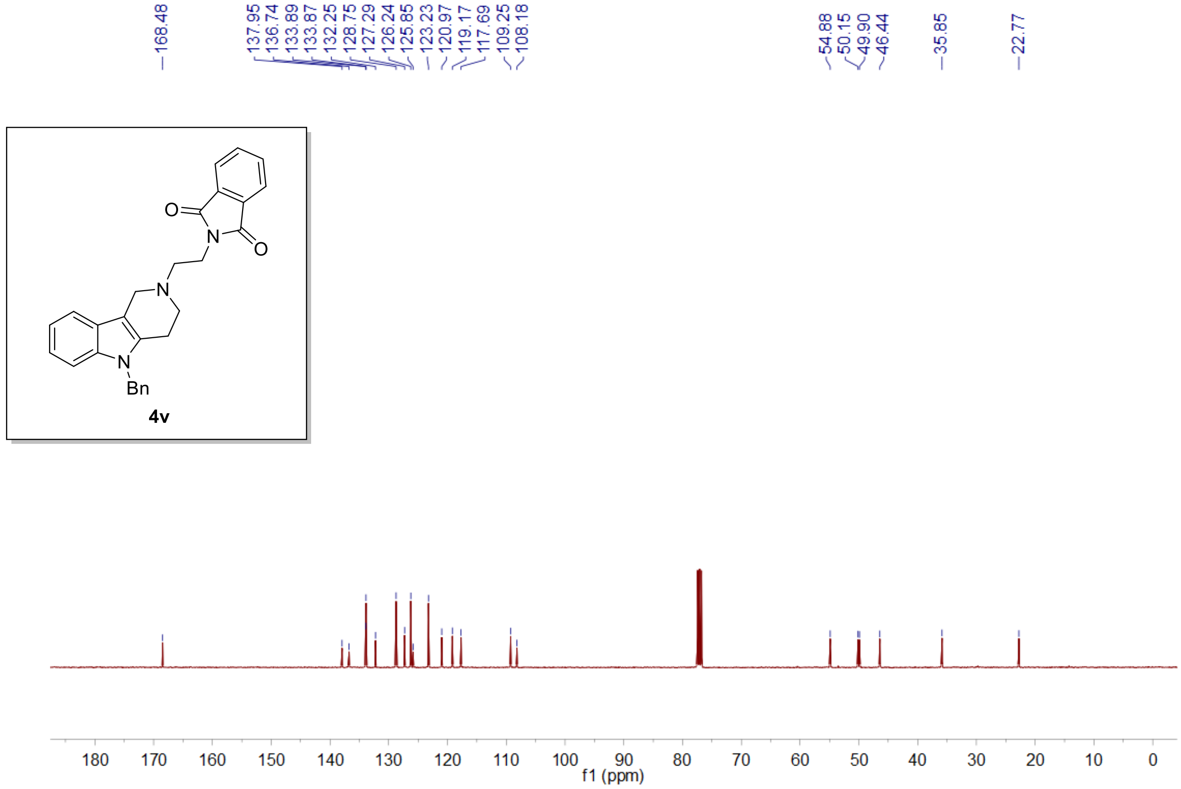

^{1}H-NMR spectrum of compound **4w** (400 MHz, $CDCl_3$)

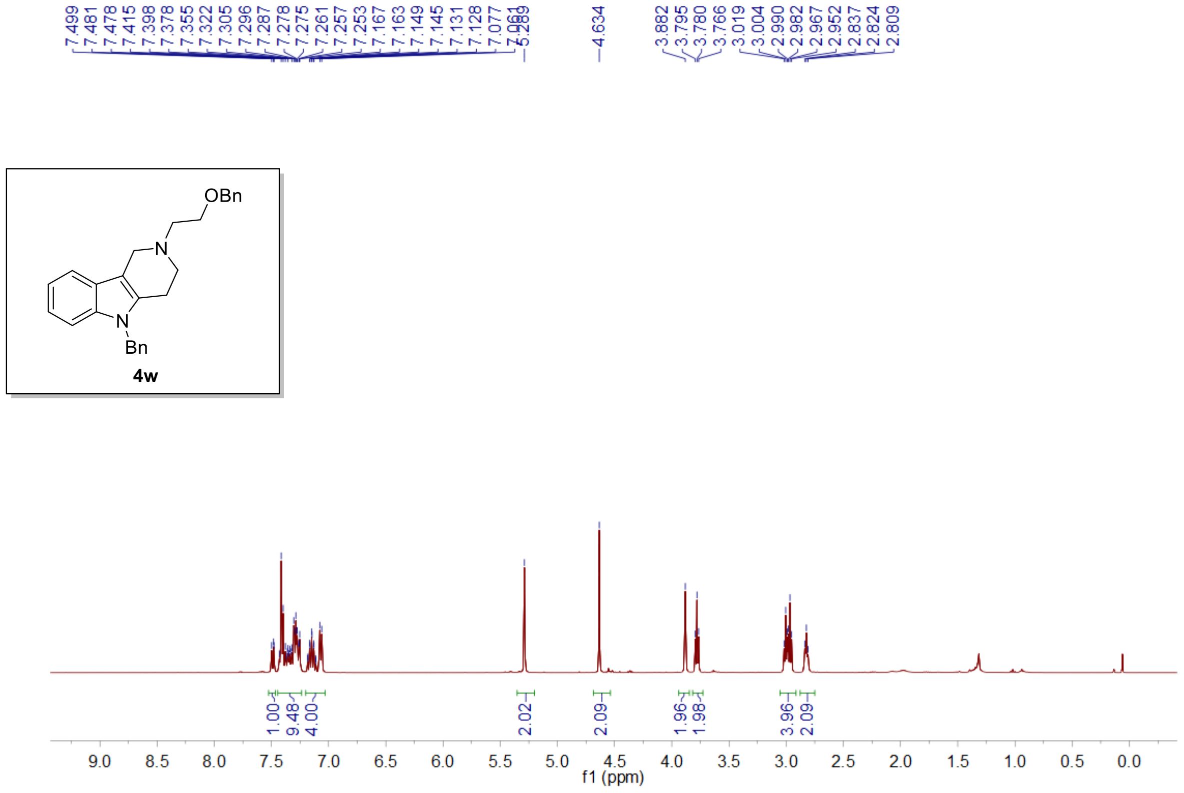

^{13}C-NMR spectrum of compound **4w** (101 MHz, $CDCl_3$)

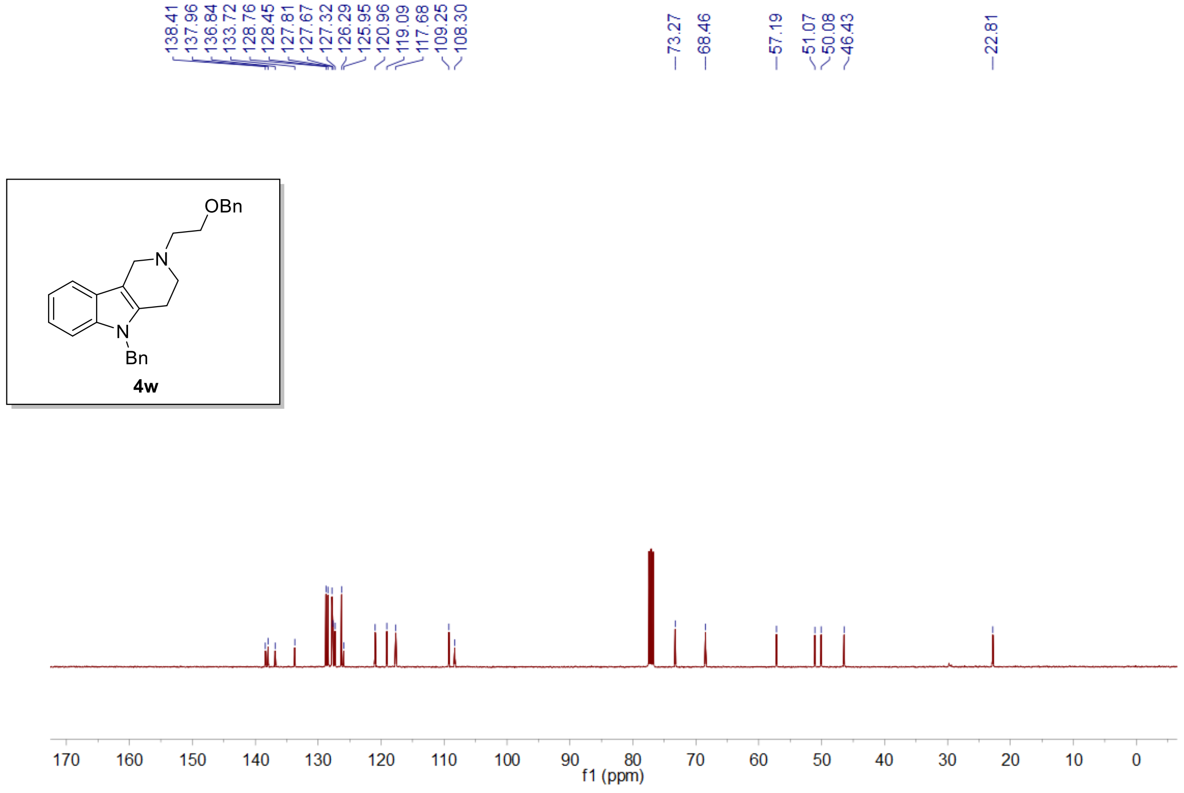

^{1}H-NMR spectrum of compound **4x** (400 MHz, $CDCl_3$)

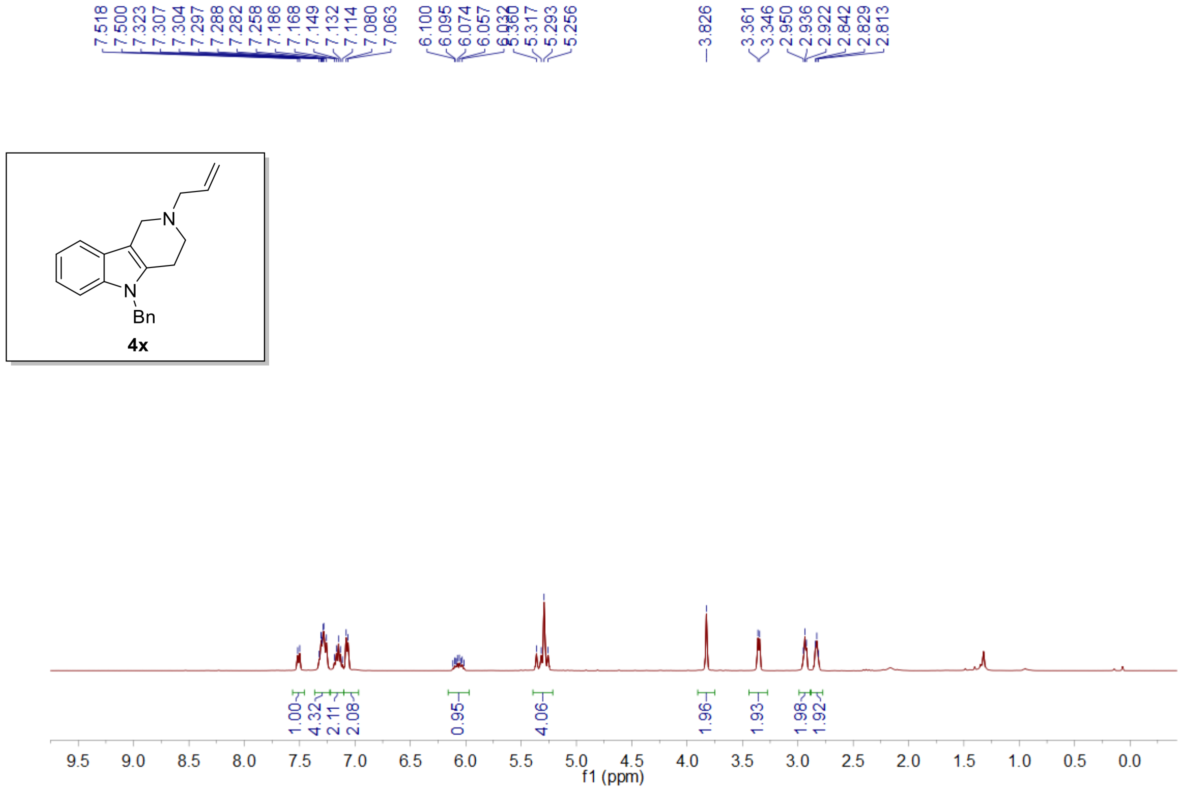

^{13}C-NMR spectrum of compound **4x** (101 MHz, $CDCl_3$)

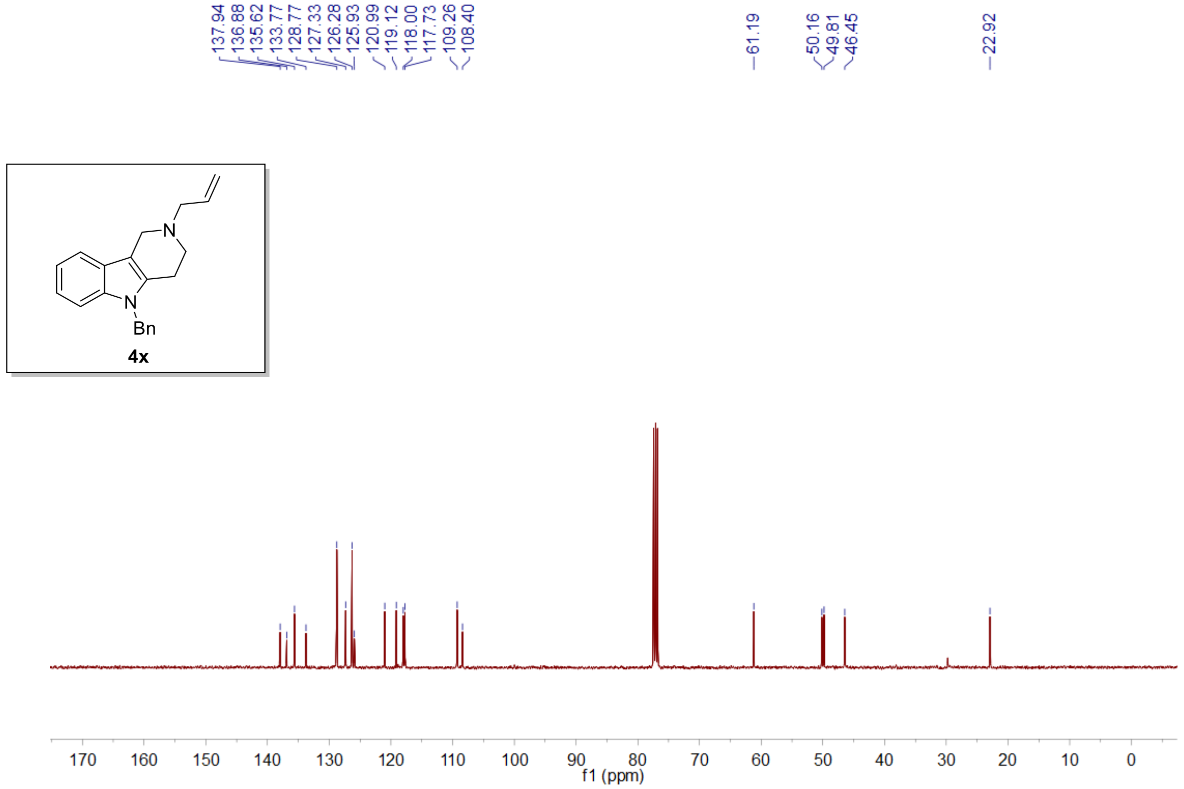

^{1}H-NMR spectrum of compound **4y** (400 MHz, $CDCl_3$)

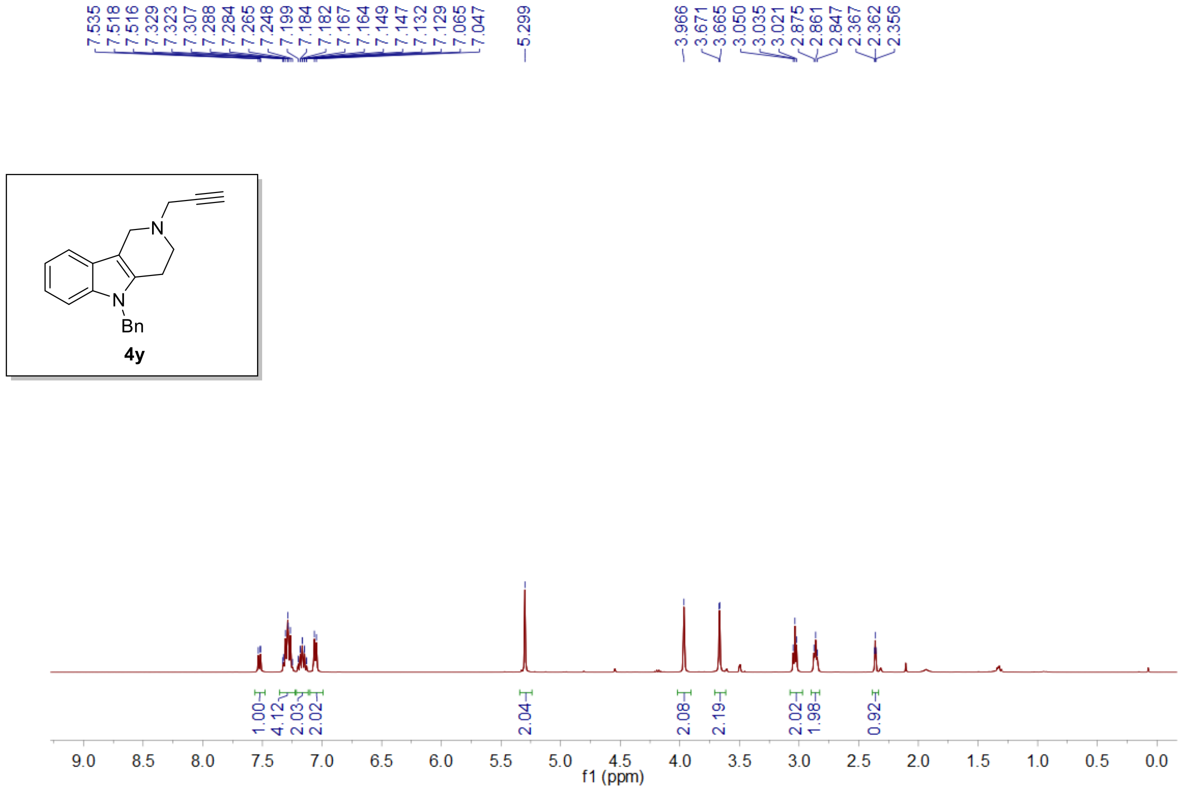

^{13}C-NMR spectrum of compound **4y** (101 MHz, $CDCl_3$)

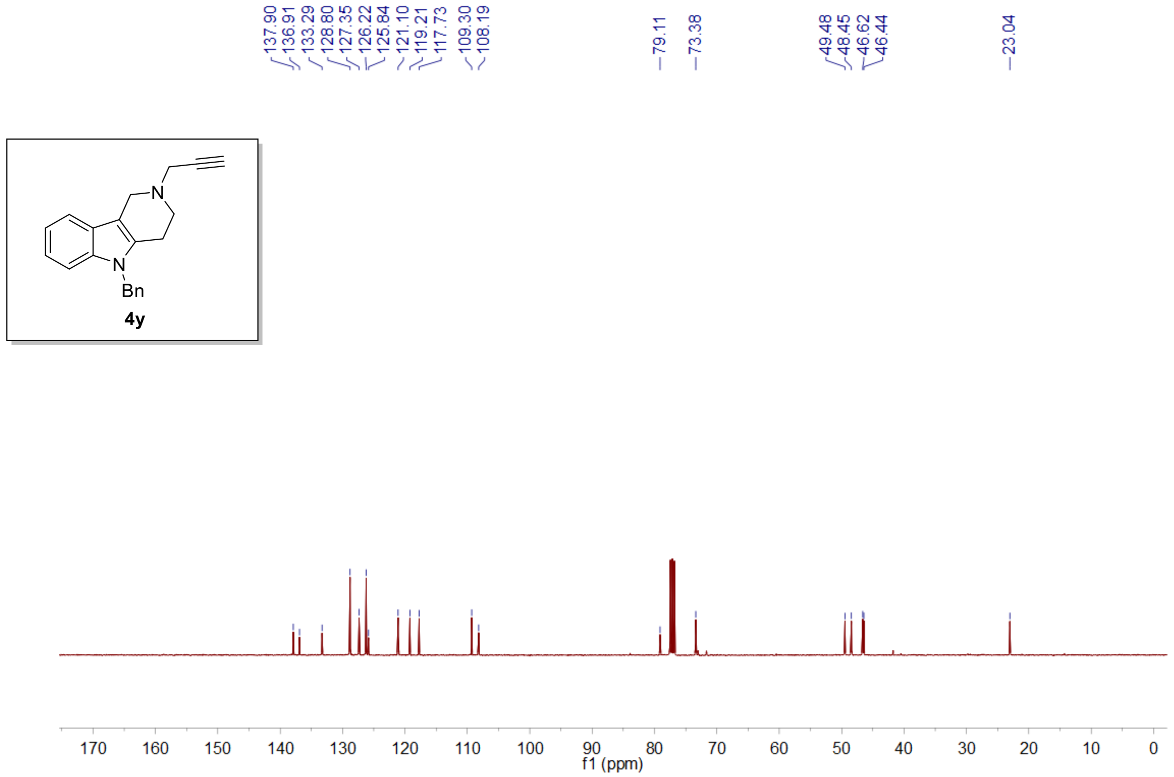

^{1}H-NMR spectrum of compound **4z** (400 MHz, $CDCl_3$)

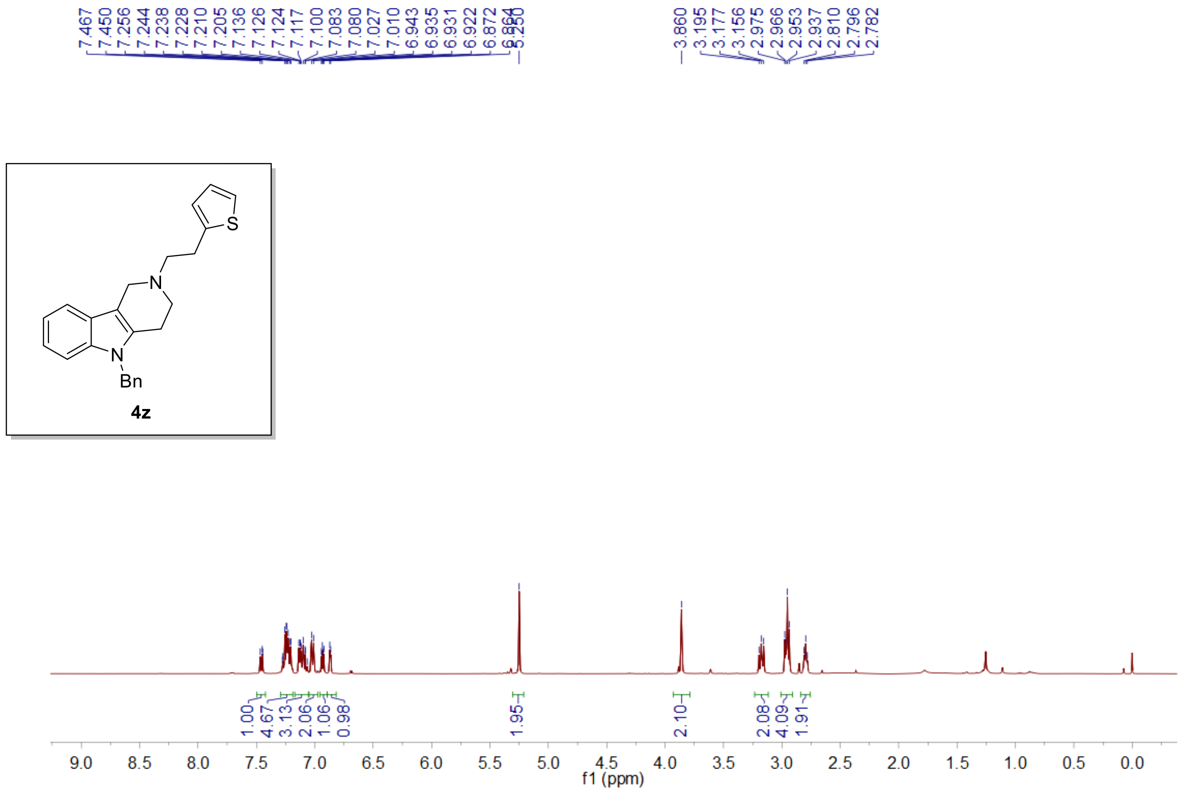

^{13}C-NMR spectrum of compound **4z** (101 MHz, $CDCl_3$)

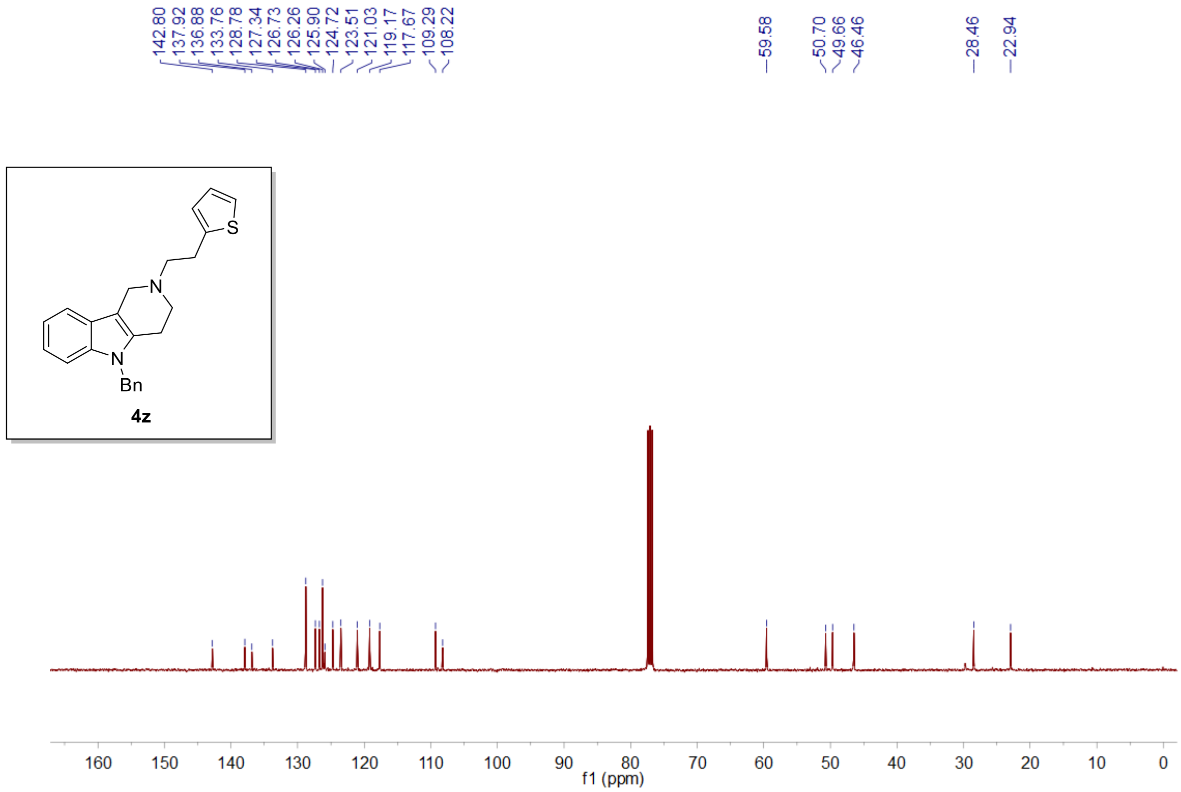

^{1}H-NMR spectrum of compound **4aa** (400 MHz, $CDCl_3$)

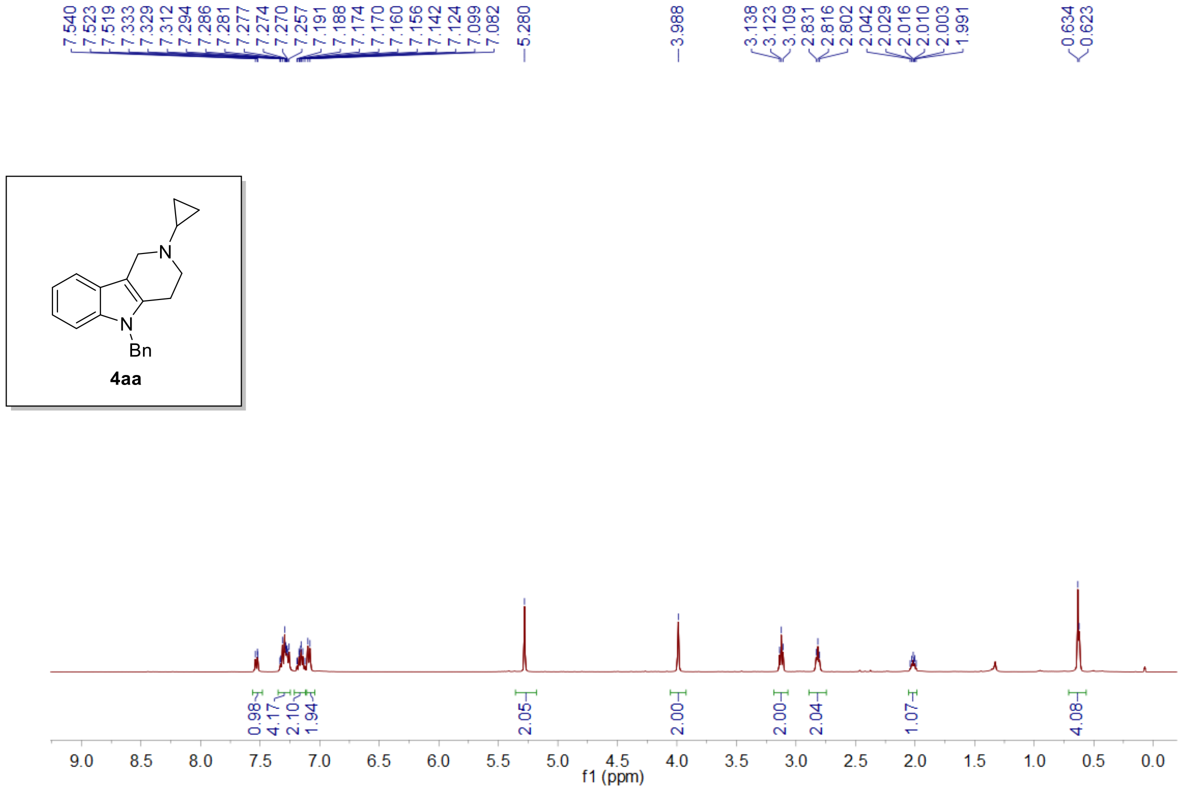

^{13}C-NMR spectrum of compound **4aa** (101 MHz, $CDCl_3$)

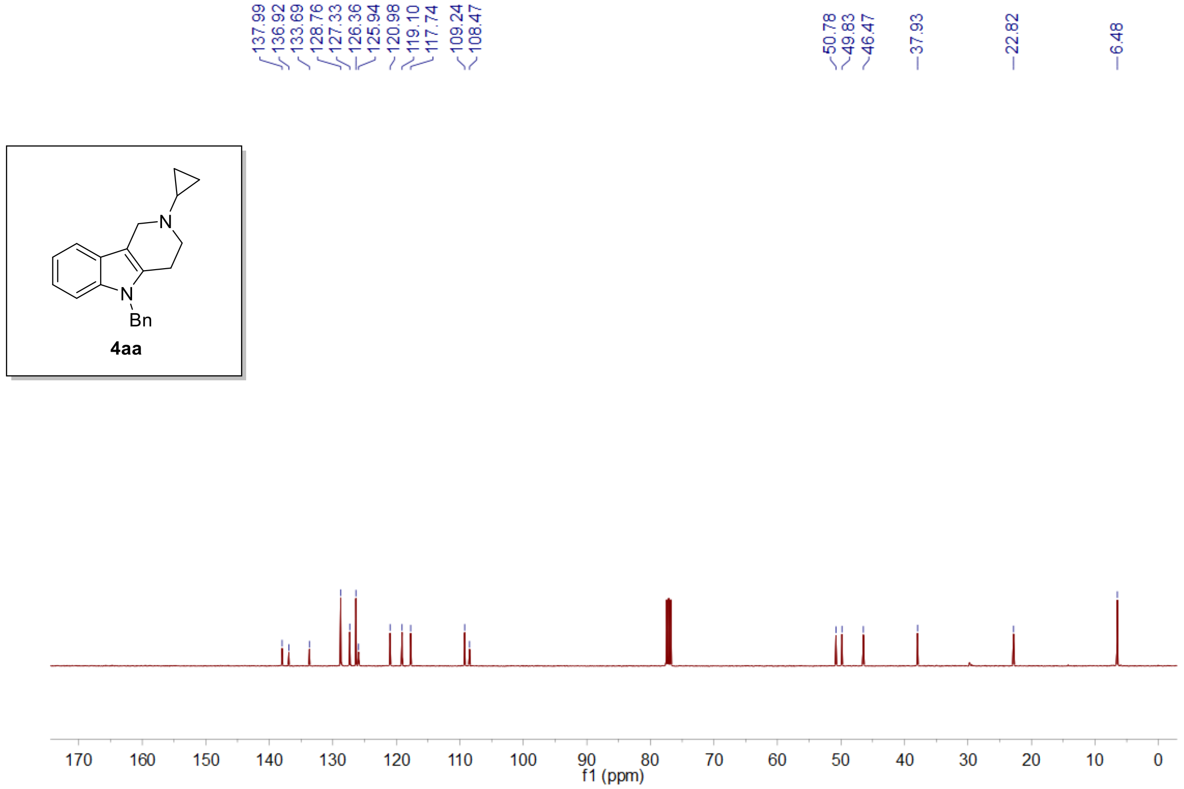

^{1}H-NMR spectrum of compound **4ab** (400 MHz, $CDCl_3$)

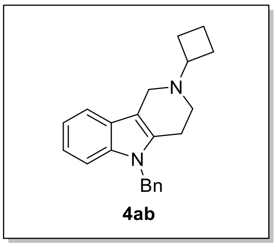

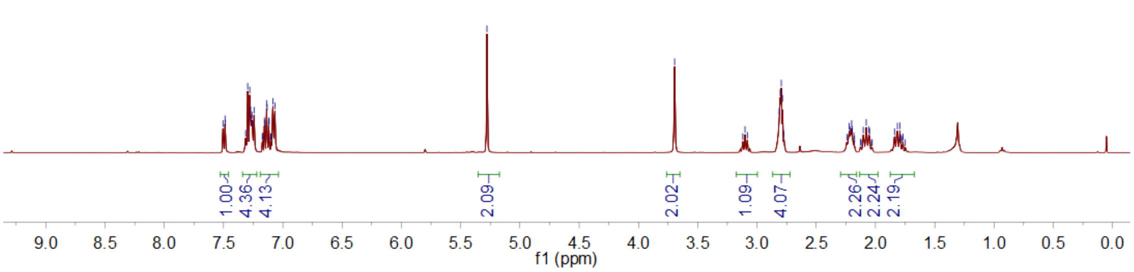

^{13}C-NMR spectrum of compound **4ab** (101 MHz, $CDCl_3$)

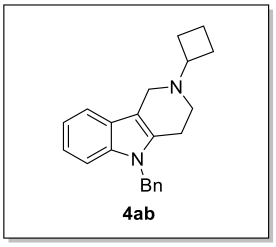

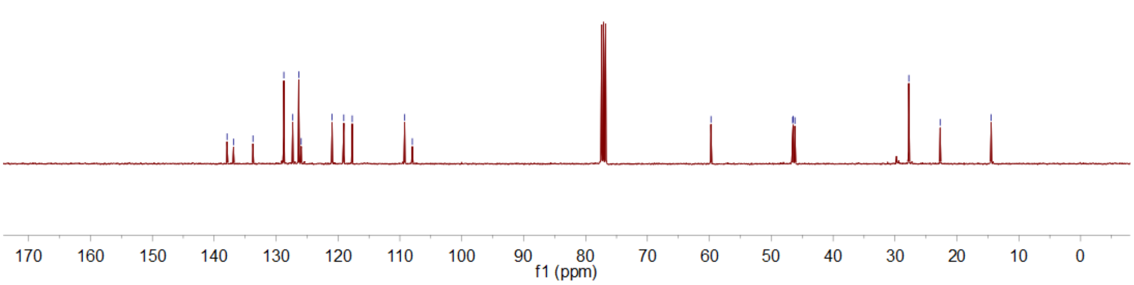

^{1}H-NMR spectrum of compound **4ac** (400 MHz, $CDCl_3$)

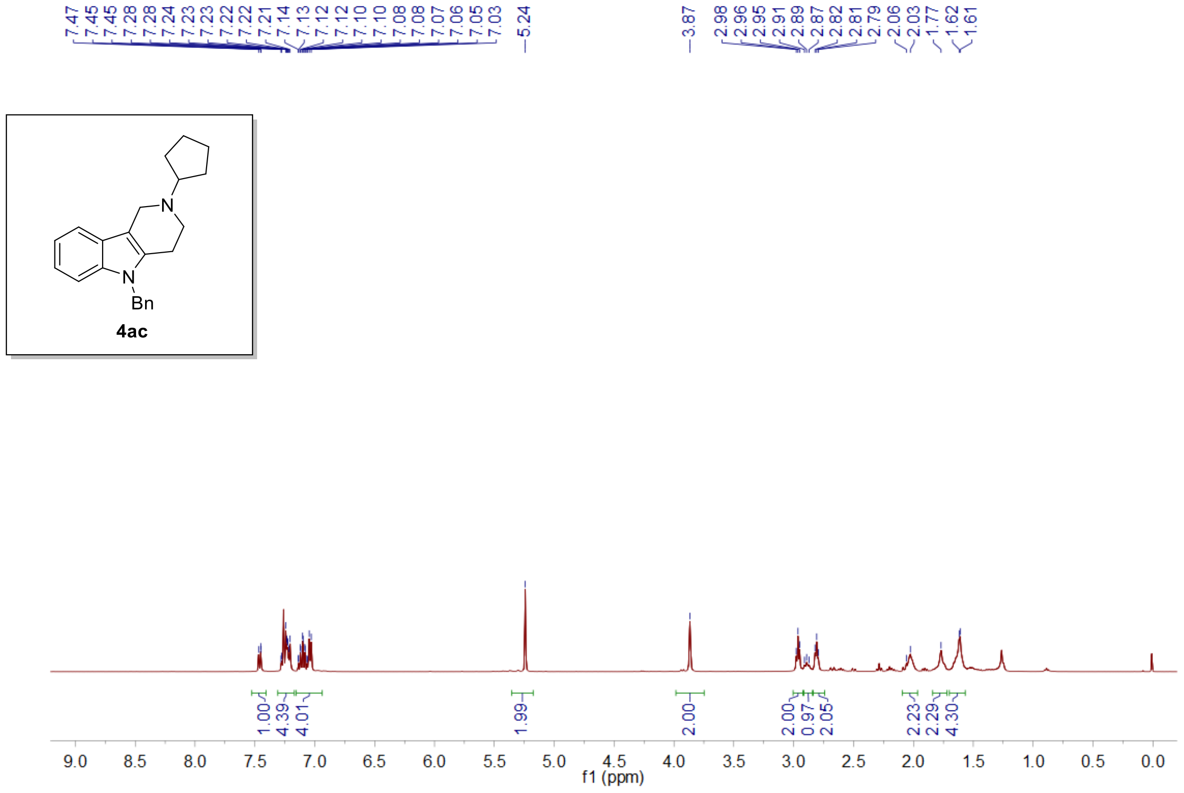

^{13}C-NMR spectrum of compound **4ac** (101 MHz, $CDCl_3$)

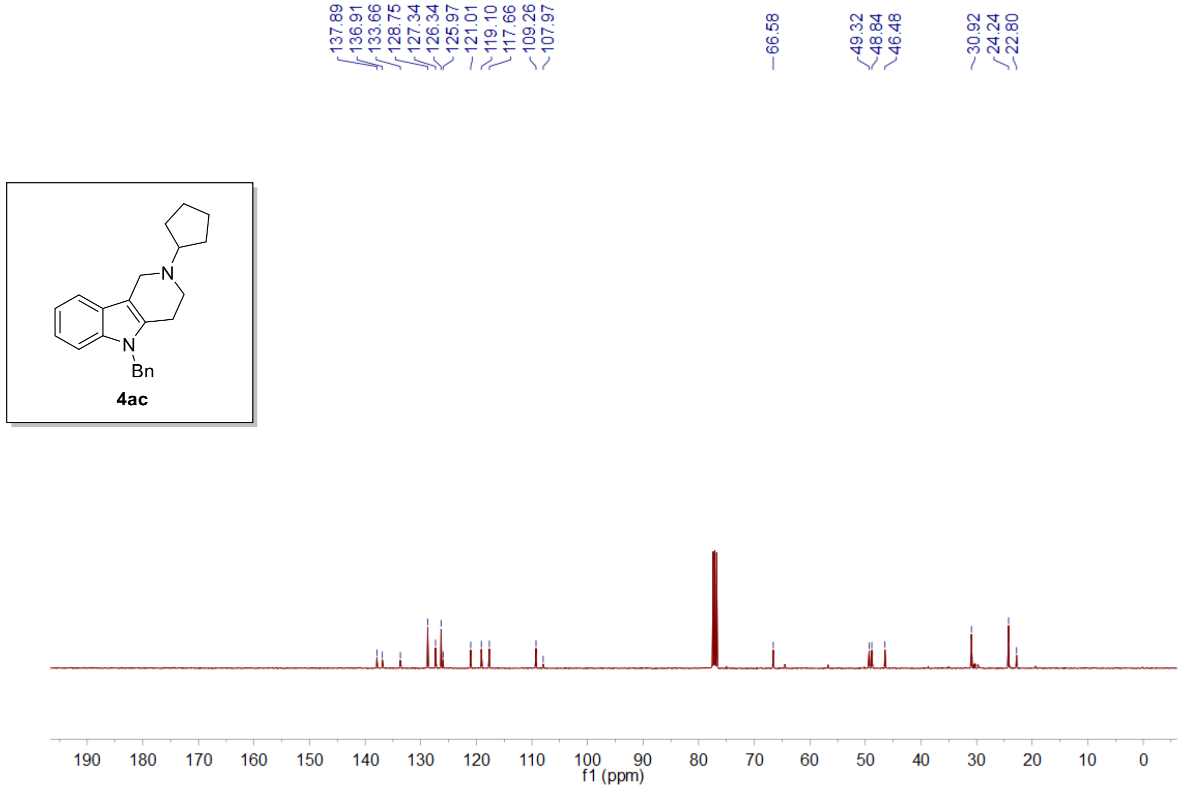

^{1}H-NMR spectrum of compound **4ad** (400 MHz, $CDCl_3$)

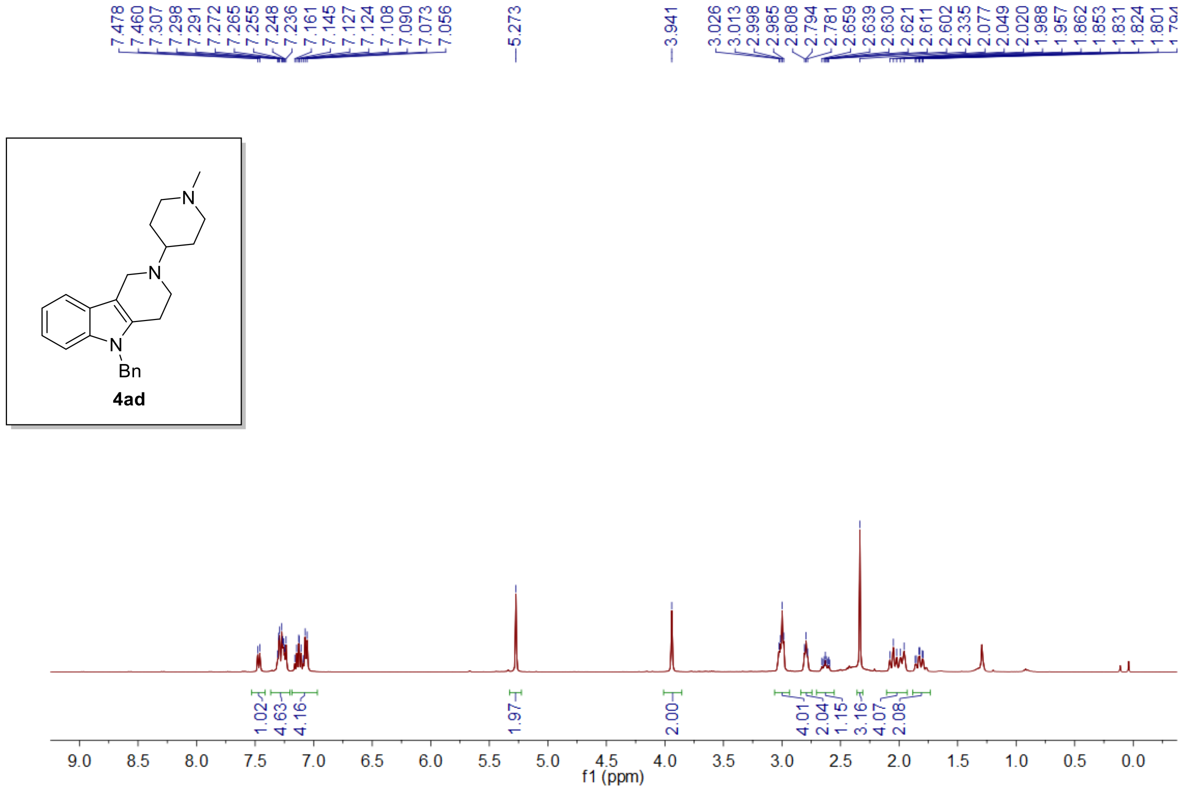

^{13}C-NMR spectrum of compound **4ad** (101 MHz, $CDCl_3$)

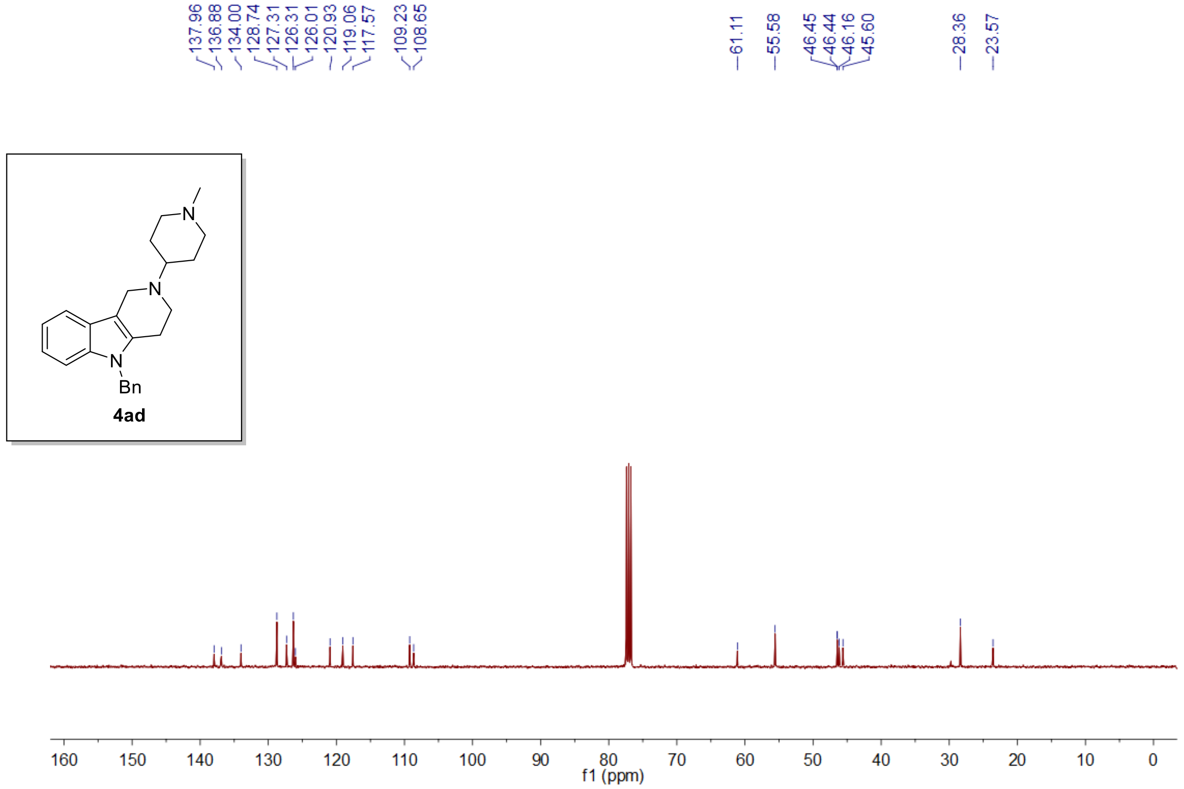

^{1}H-NMR spectrum of compound **4ae** (400 MHz, $CDCl_3$)

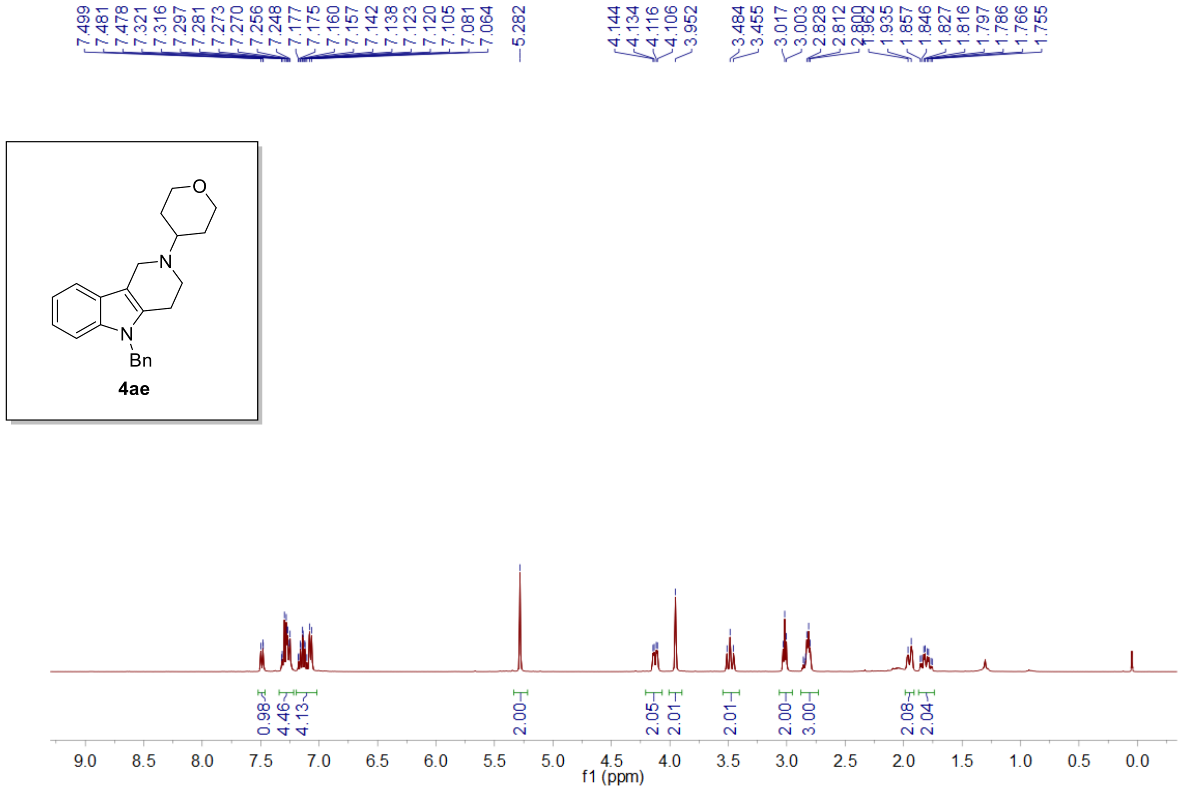

^{13}C-NMR spectrum of compound **4ae** (101 MHz, $CDCl_3$)

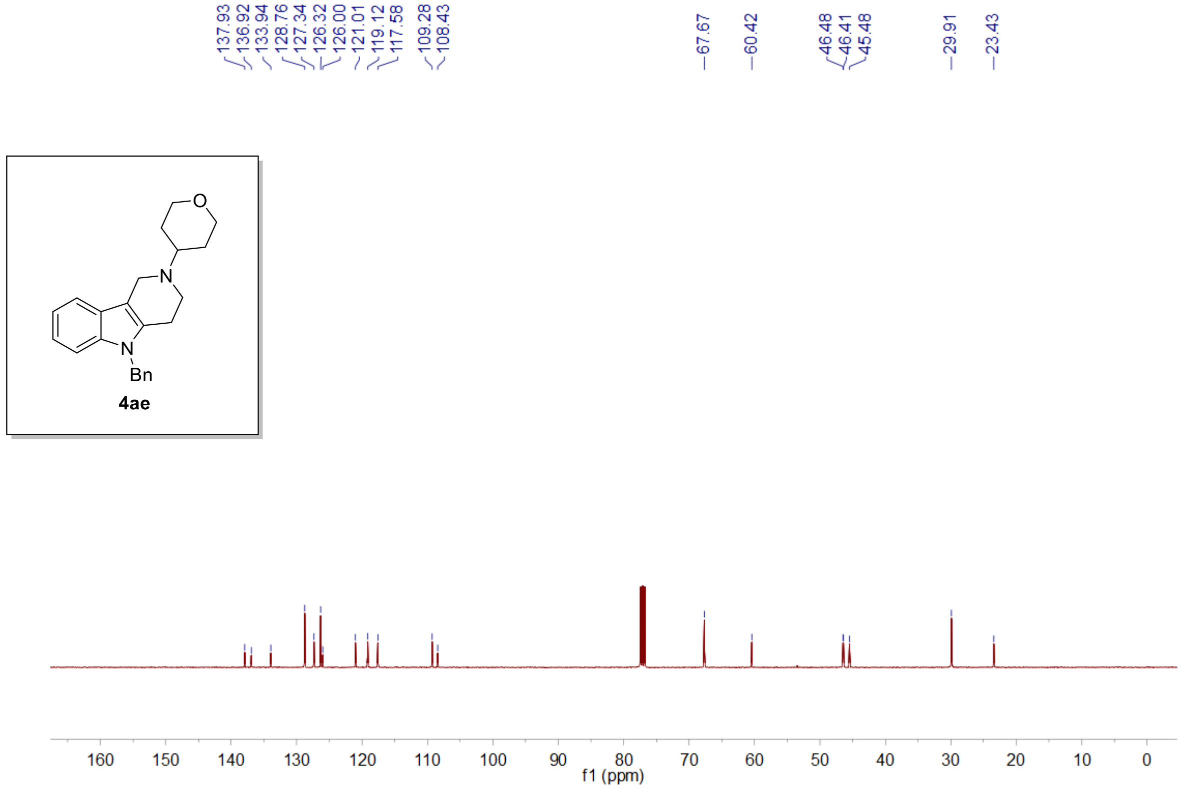

^{1}H-NMR spectrum of compound **4af** (400 MHz, $CDCl_3$)

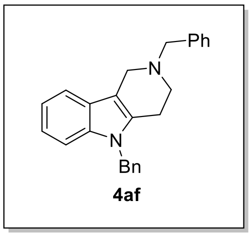

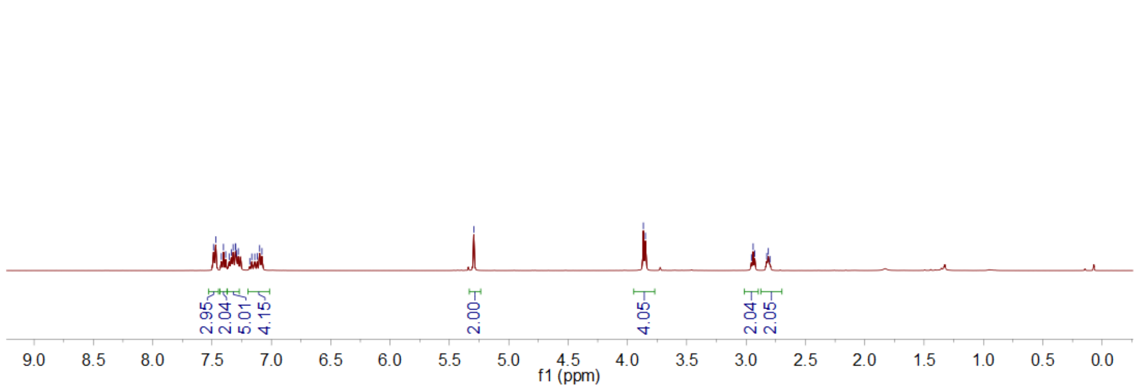

^{13}C-NMR spectrum of compound **4af** (101 MHz, $CDCl_3$)

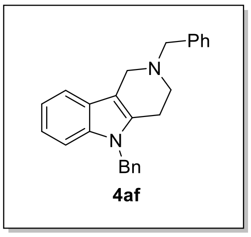

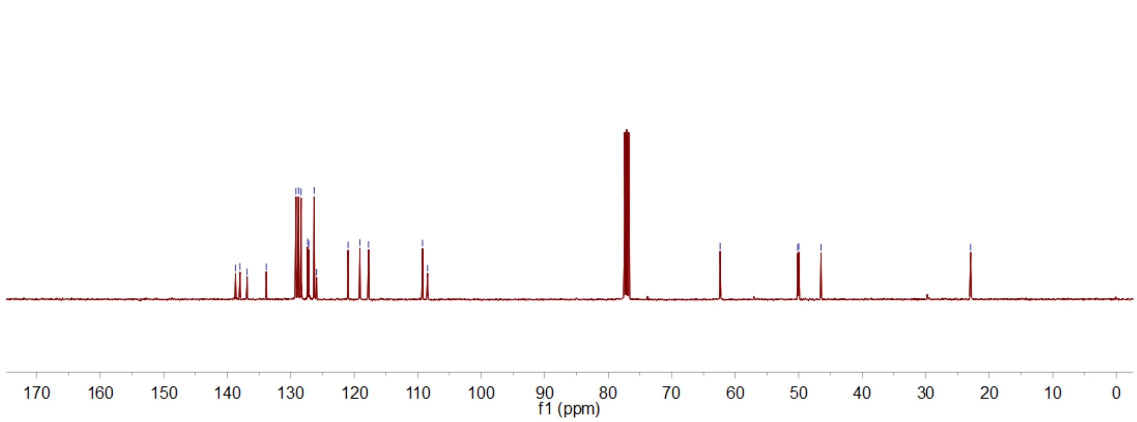

^{1}H-NMR spectrum of compound **4ag** (400 MHz, $CDCl_3$)

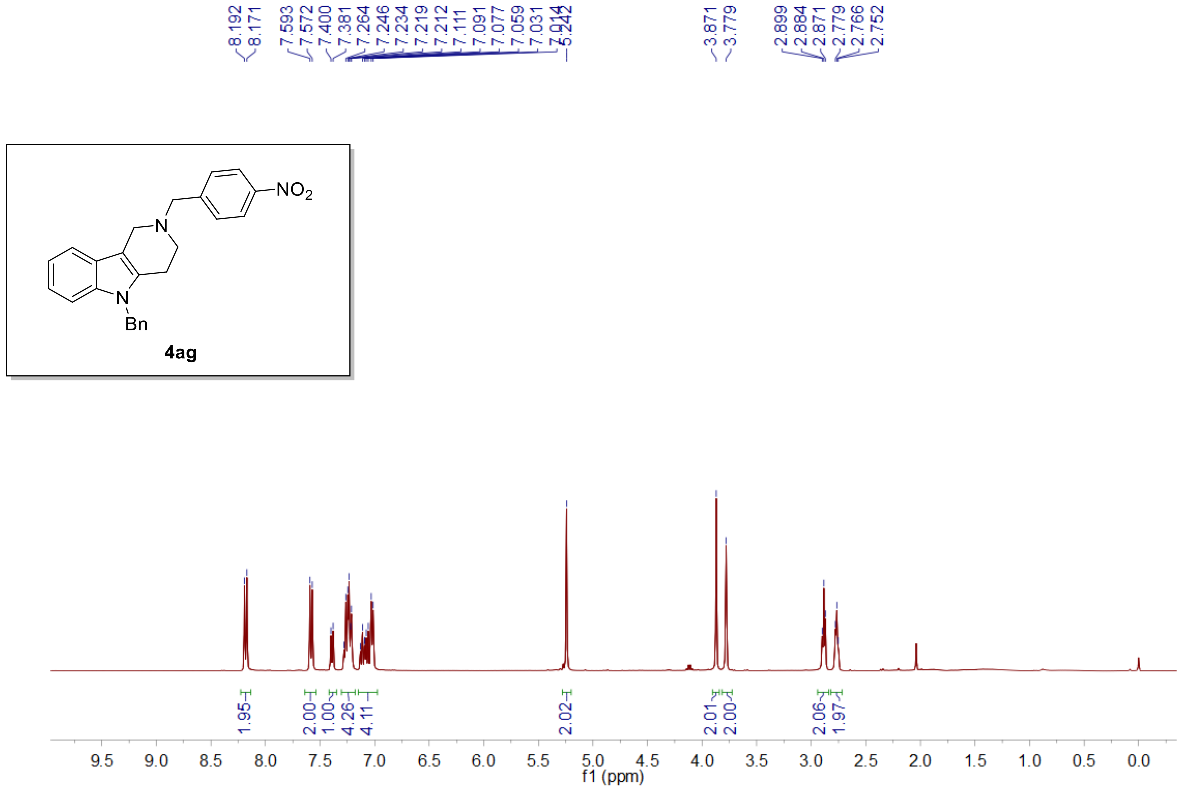

^{13}C-NMR spectrum of compound **4ag** (101 MHz, $CDCl_3$)

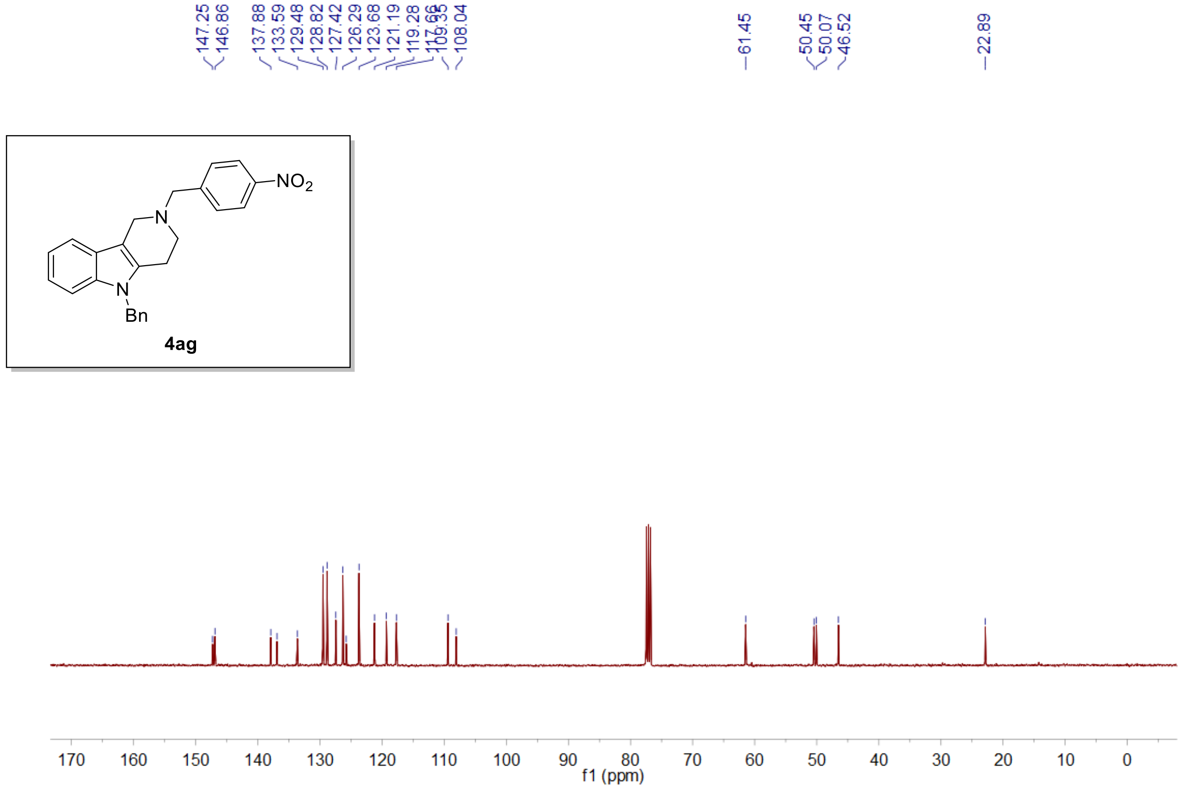

^{1}H-NMR spectrum of compound **4ah** (400 MHz, $CDCl_3$)

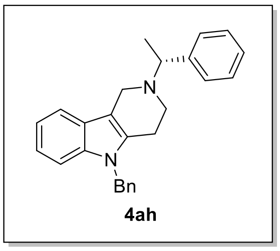

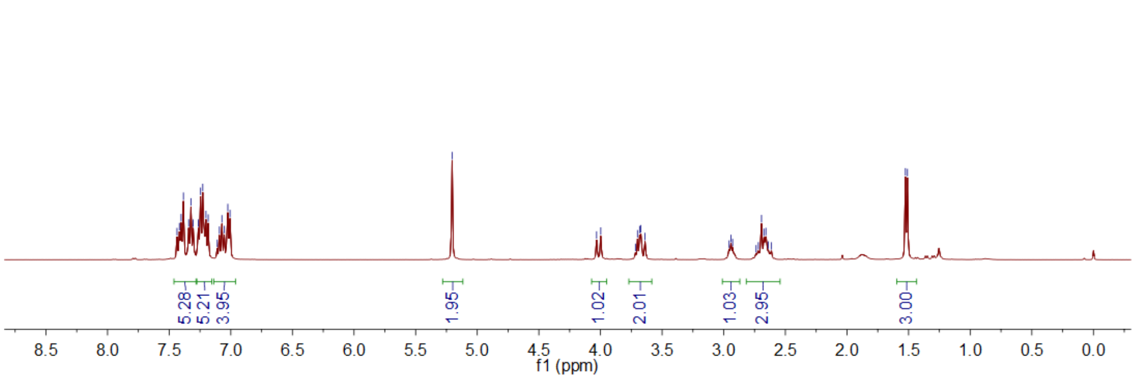

^{13}C-NMR spectrum of compound **4ah** (101 MHz, $CDCl_3$)

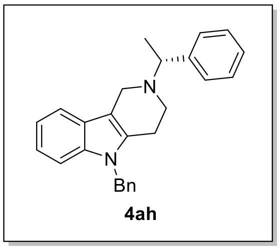

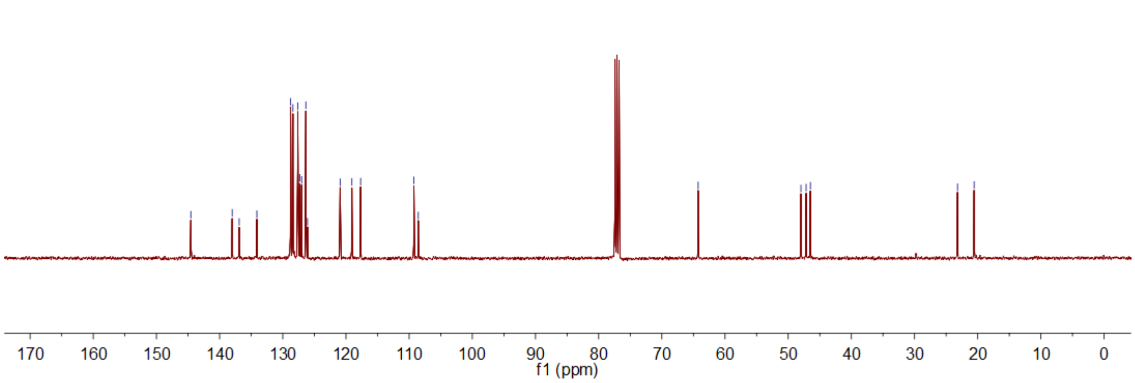

^{1}H-NMR spectrum of compound **4ai** (400 MHz, $CDCl_3$)

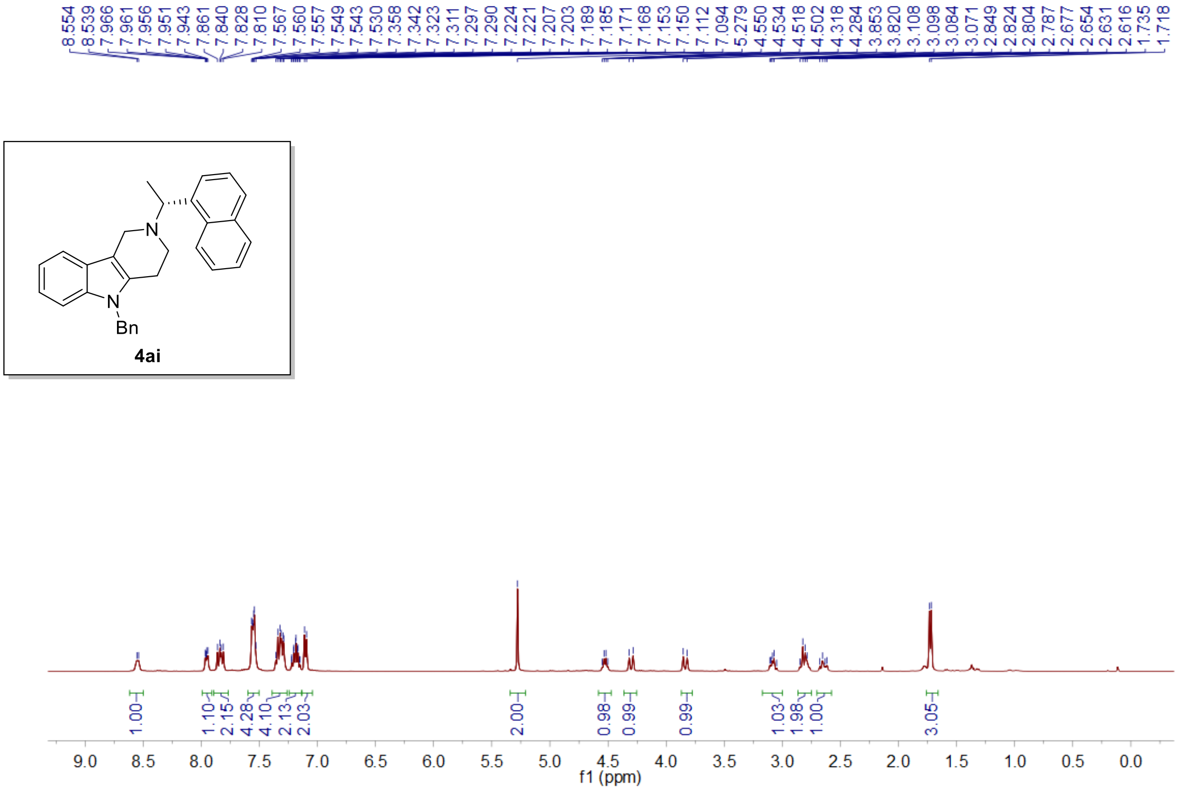

^{13}C-NMR spectrum of compound **4ai** (101 MHz, $CDCl_3$)

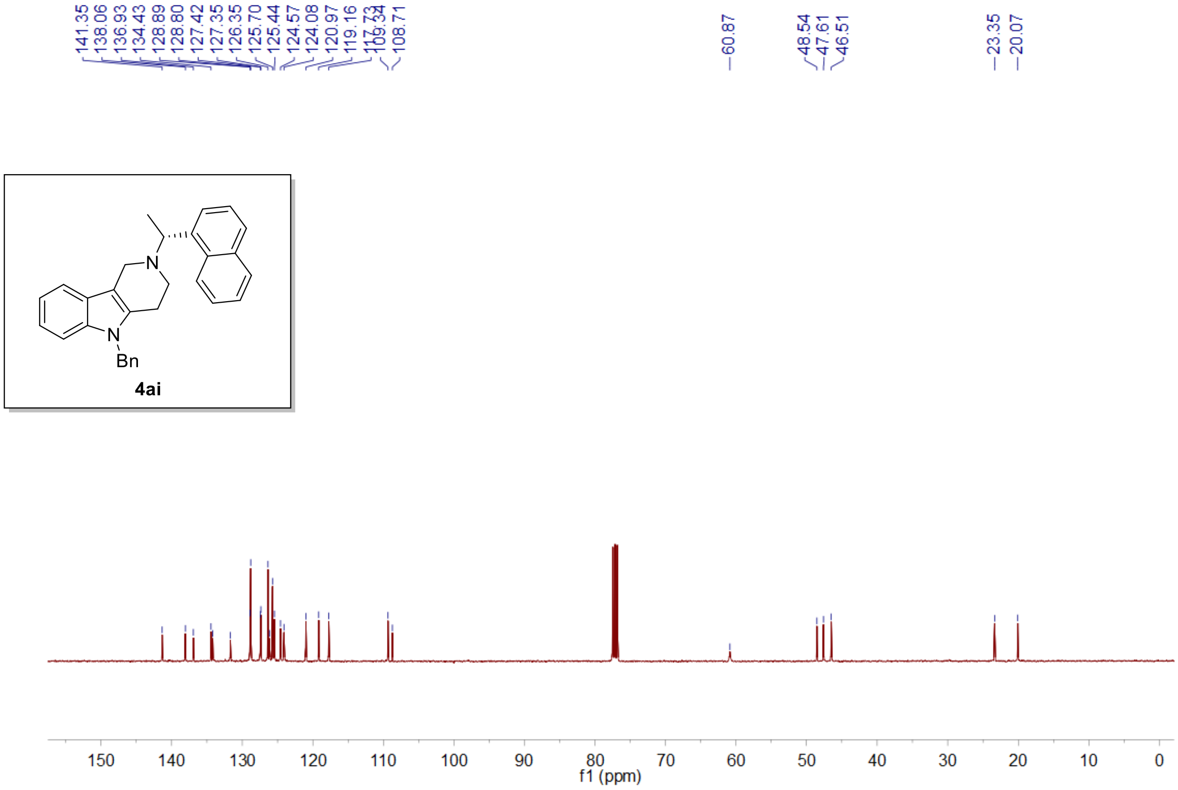

^{1}H-NMR spectrum of compound **4aj** (400 MHz, $CDCl_3$)

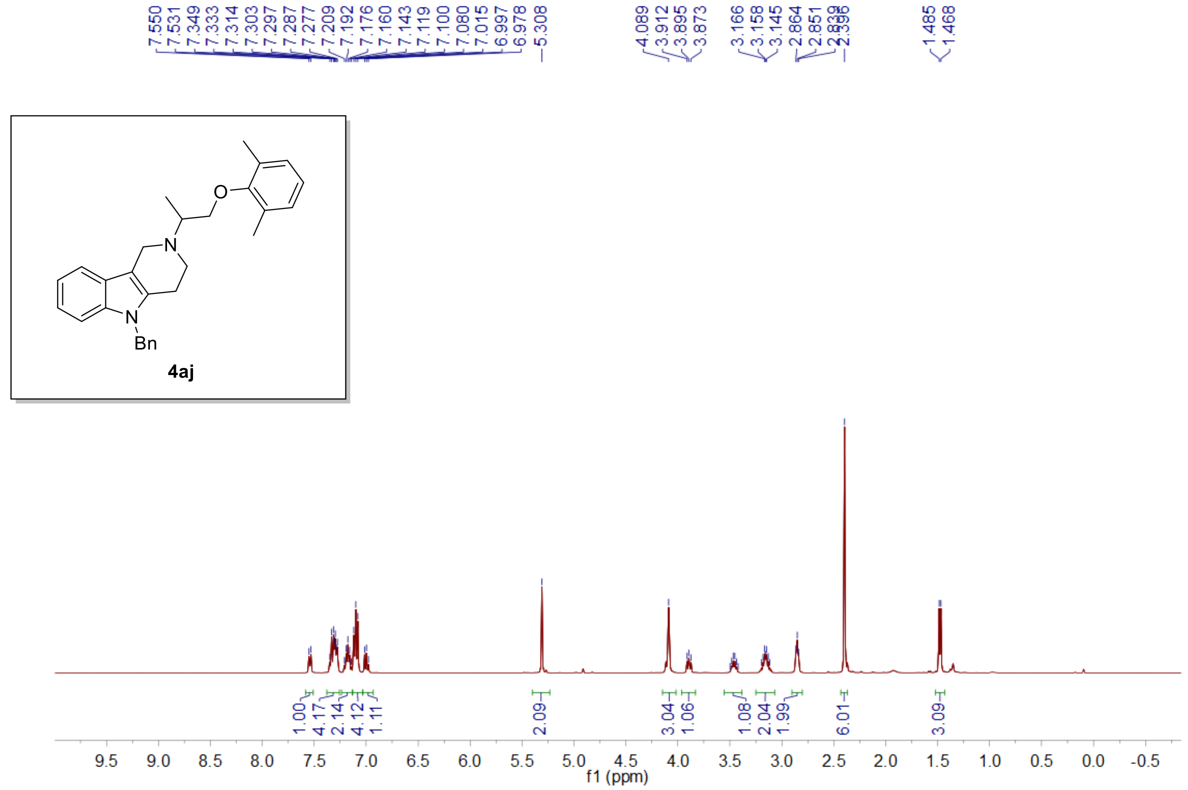

^{13}C-NMR spectrum of compound **4aj** (101 MHz, $CDCl_3$)

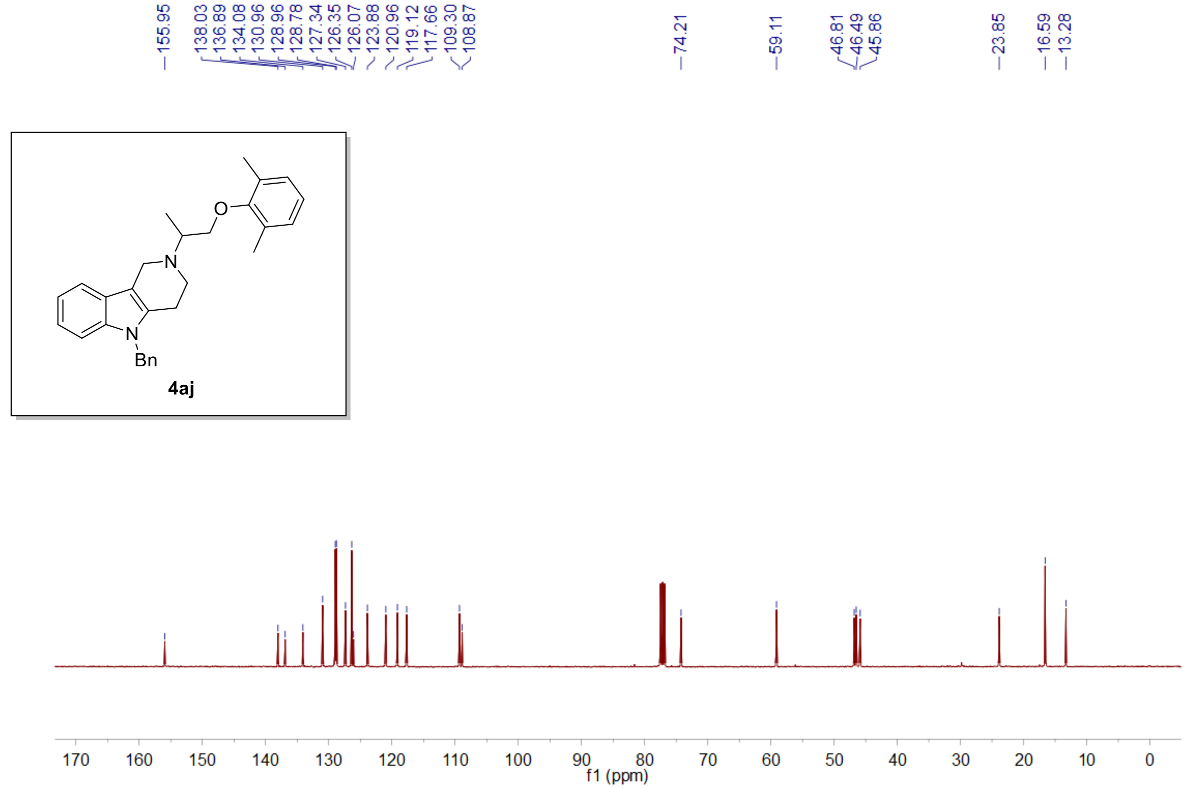

^{1}H-NMR spectrum of compound **4ak** (400 MHz, $CDCl_3$)

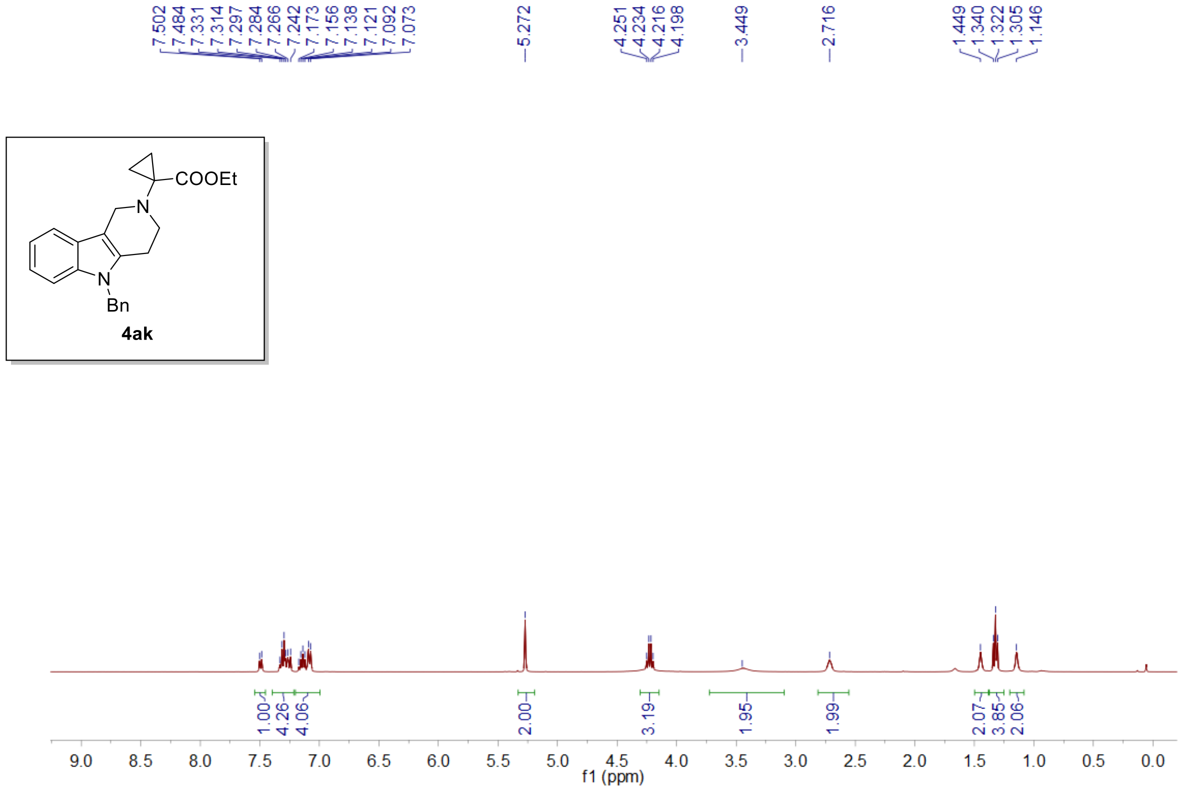

^{13}C-NMR spectrum of compound **4ak** (101 MHz, $CDCl_3$)

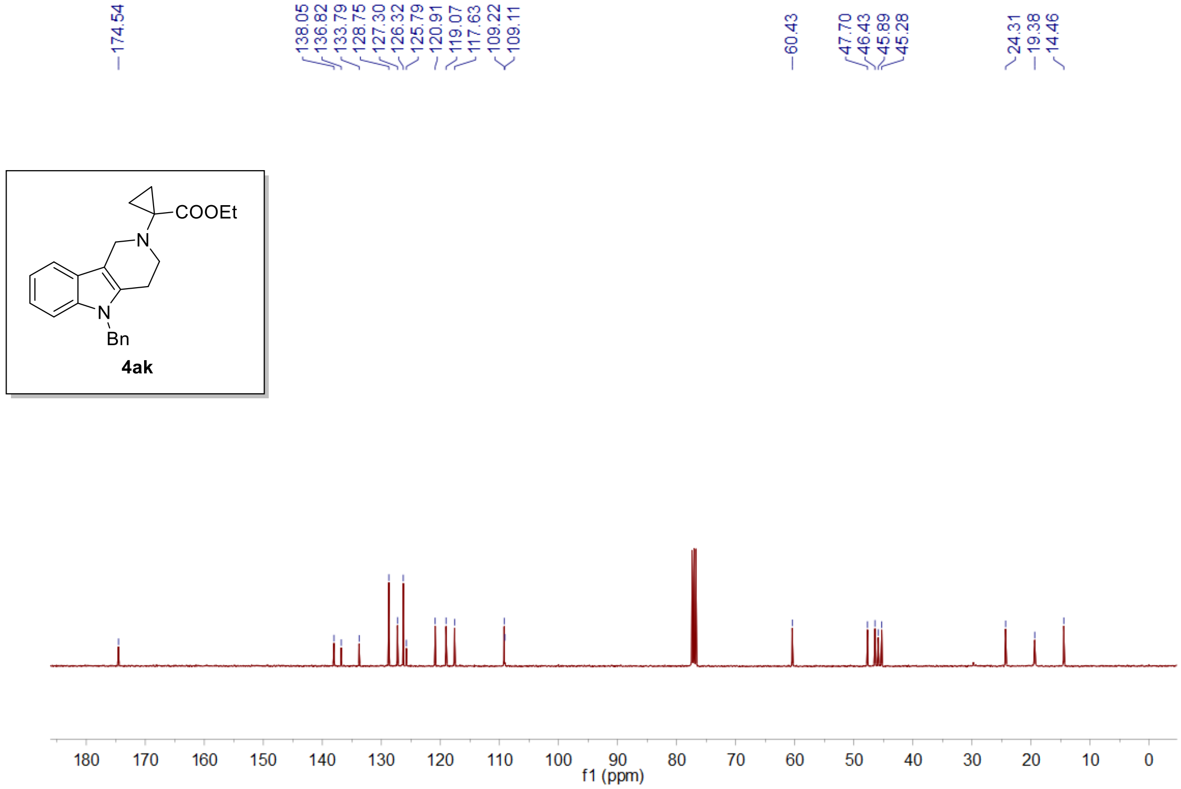

^{1}H-NMR spectrum of compound **4al** (400 MHz, $CDCl_3$)

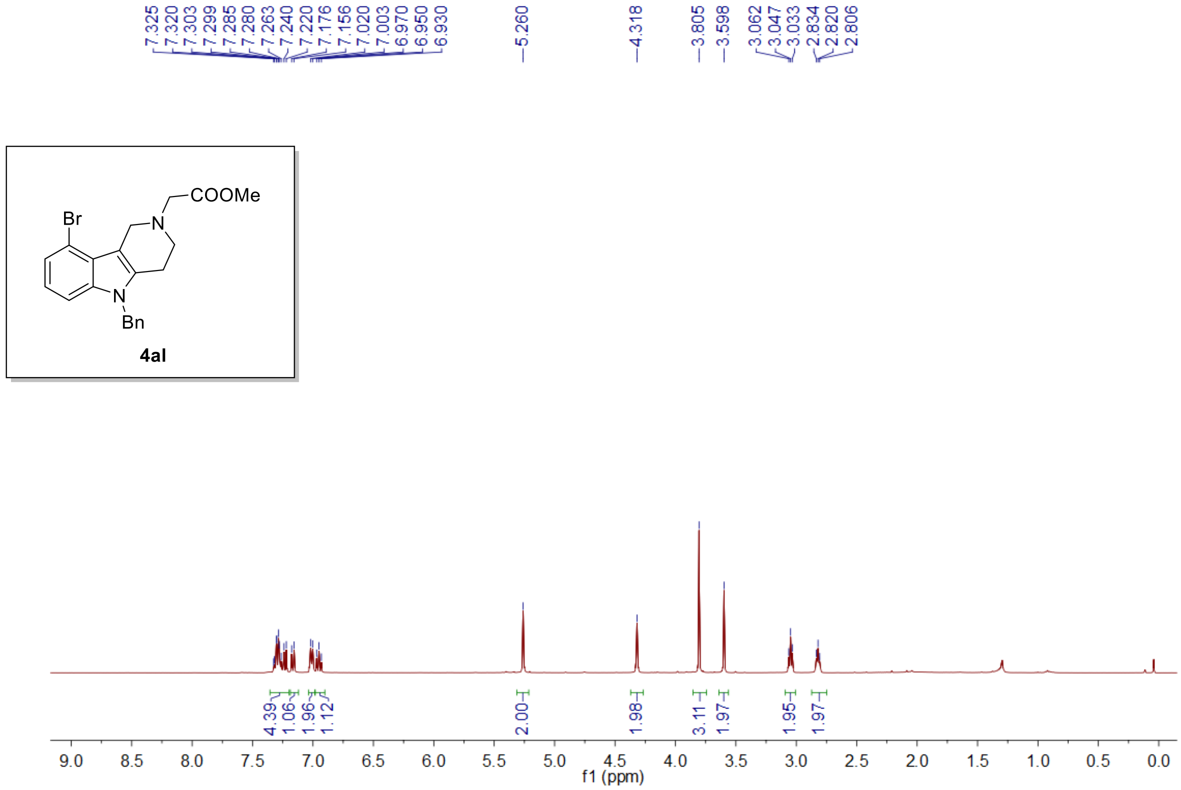

^{13}C-NMR spectrum of compound **4al** (101 MHz, $CDCl_3$)

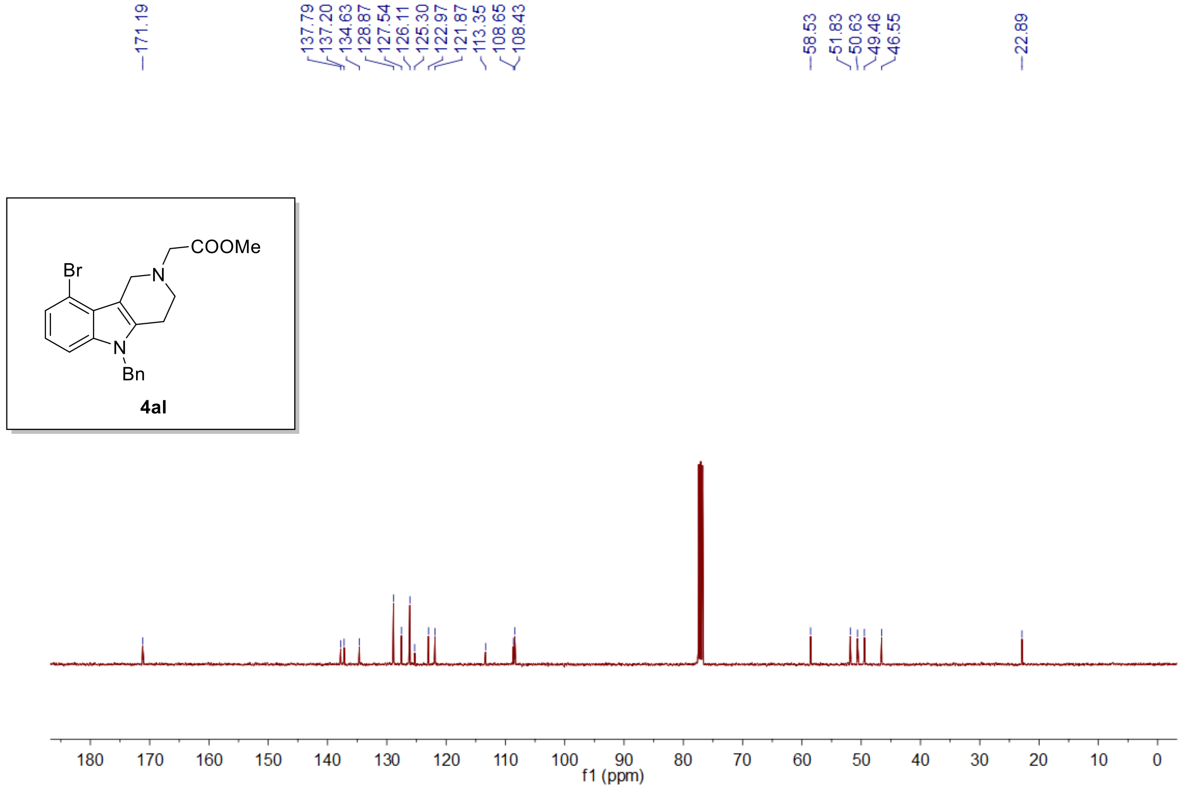

^{1}H-NMR spectrum of compound **4am** (400 MHz, $CDCl_3$)

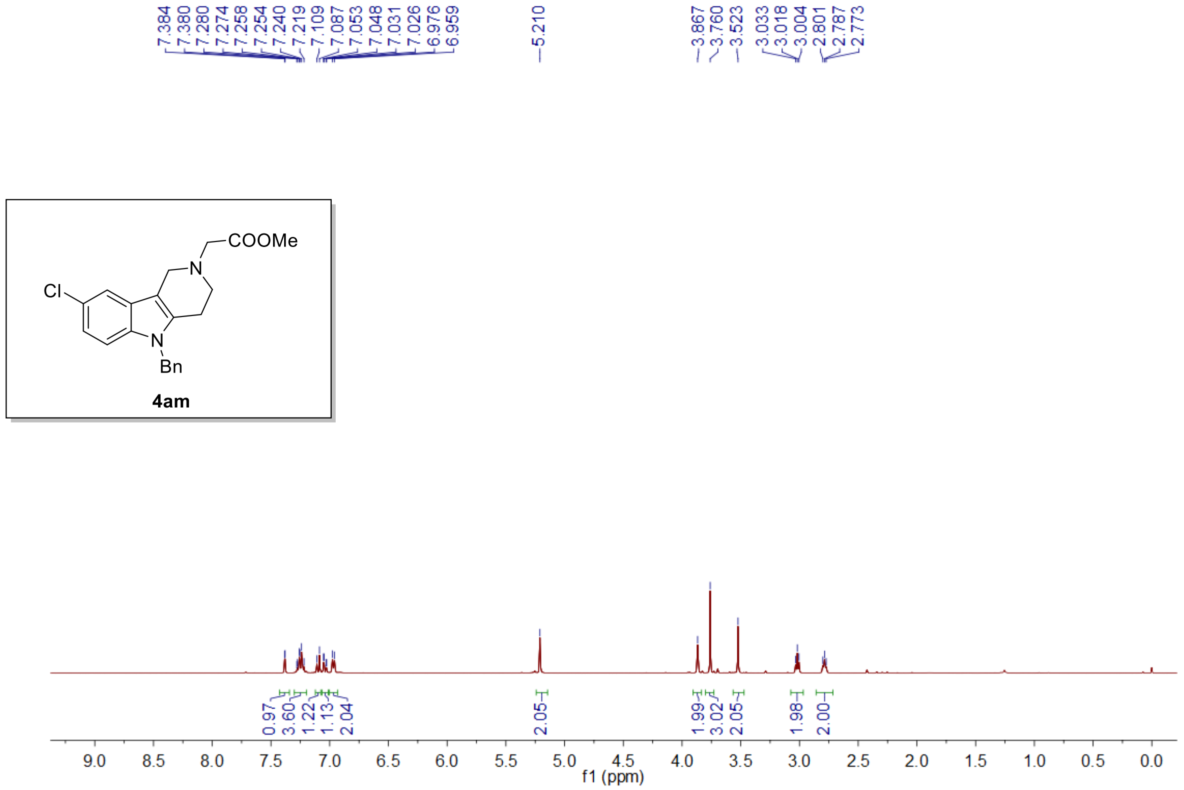

^{13}C-NMR spectrum of compound **4am** (101 MHz, $CDCl_3$)

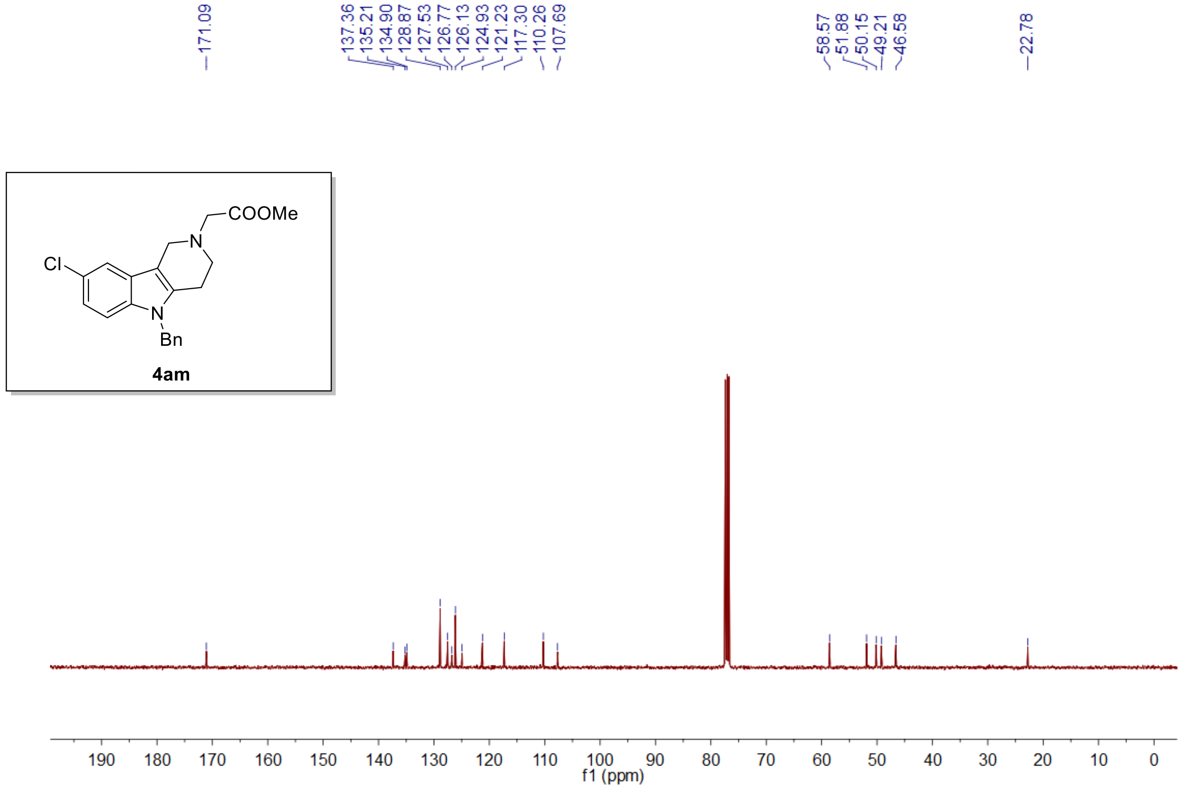

^{1}H-NMR spectrum of compound **4an** (400 MHz, $CDCl_3$)

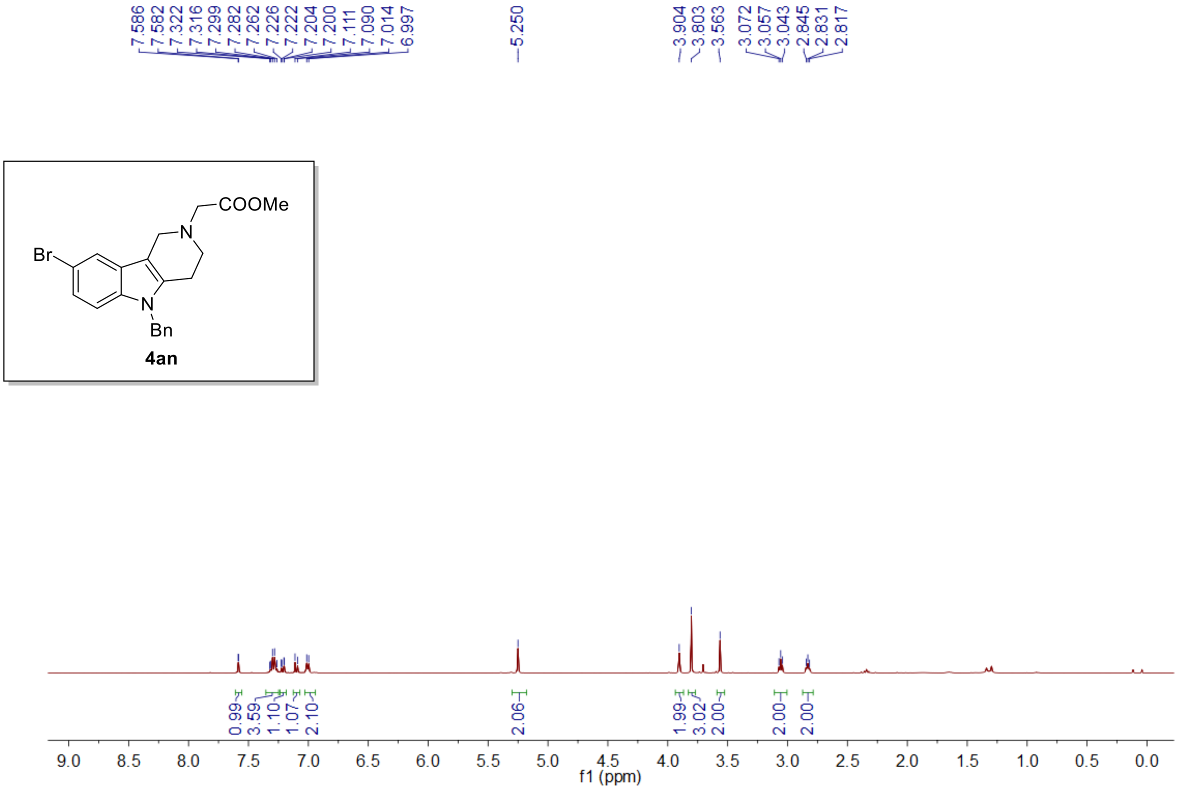

^{13}C-NMR spectrum of compound **4an** (101 MHz, $CDCl_3$)

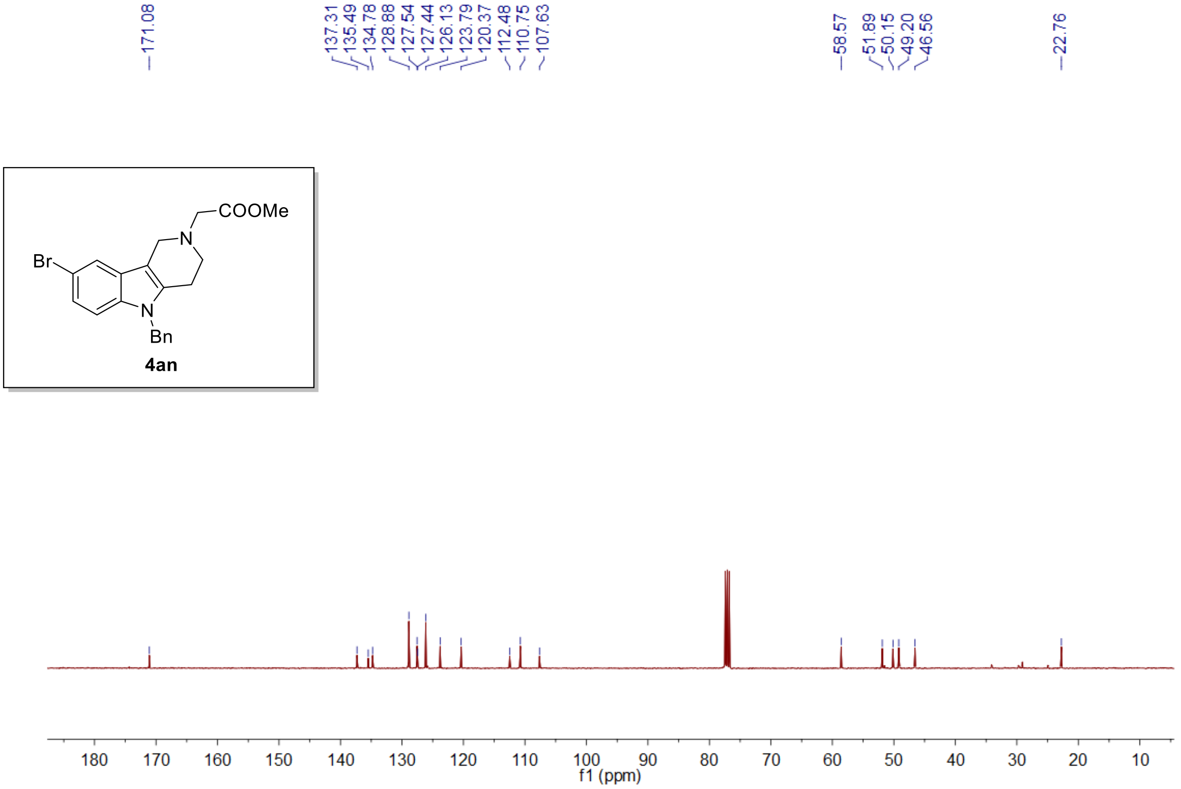

^{1}H-NMR spectrum of compound **4ao** (400 MHz, $CDCl_3$)

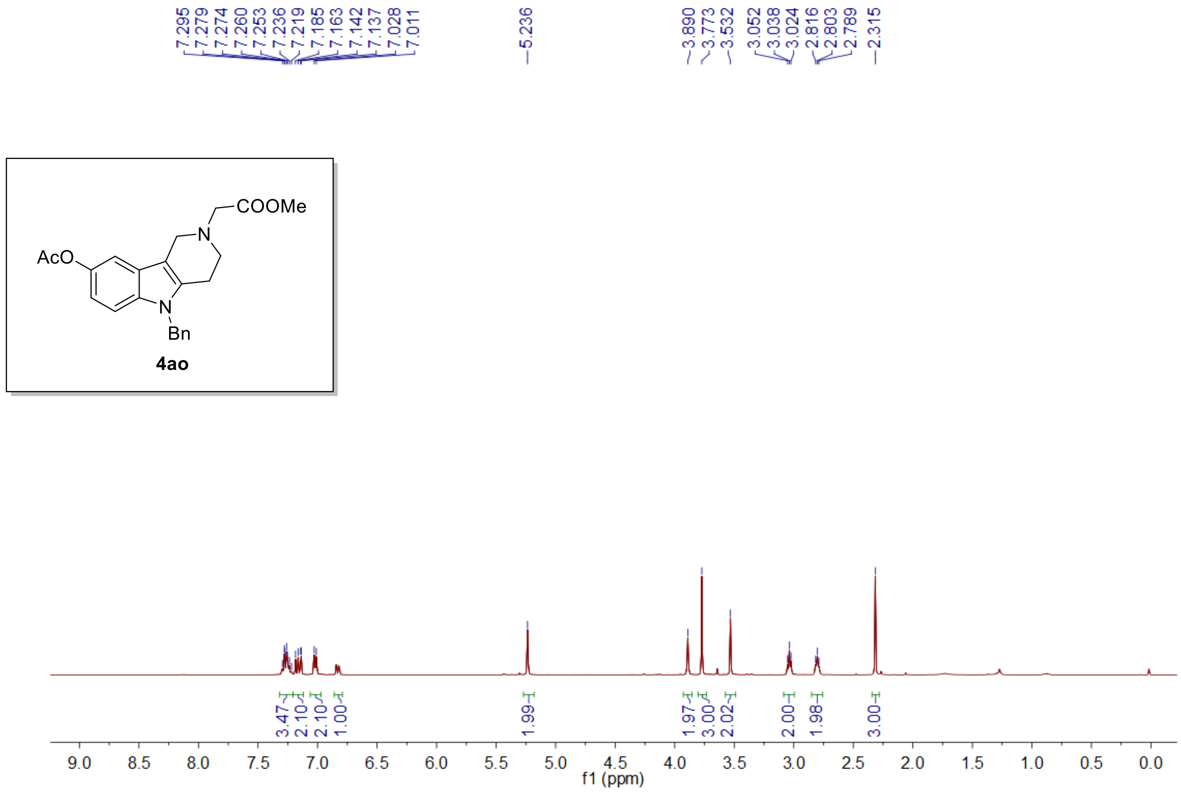

^{13}C-NMR spectrum of compound **4ao** (101 MHz, $CDCl_3$)

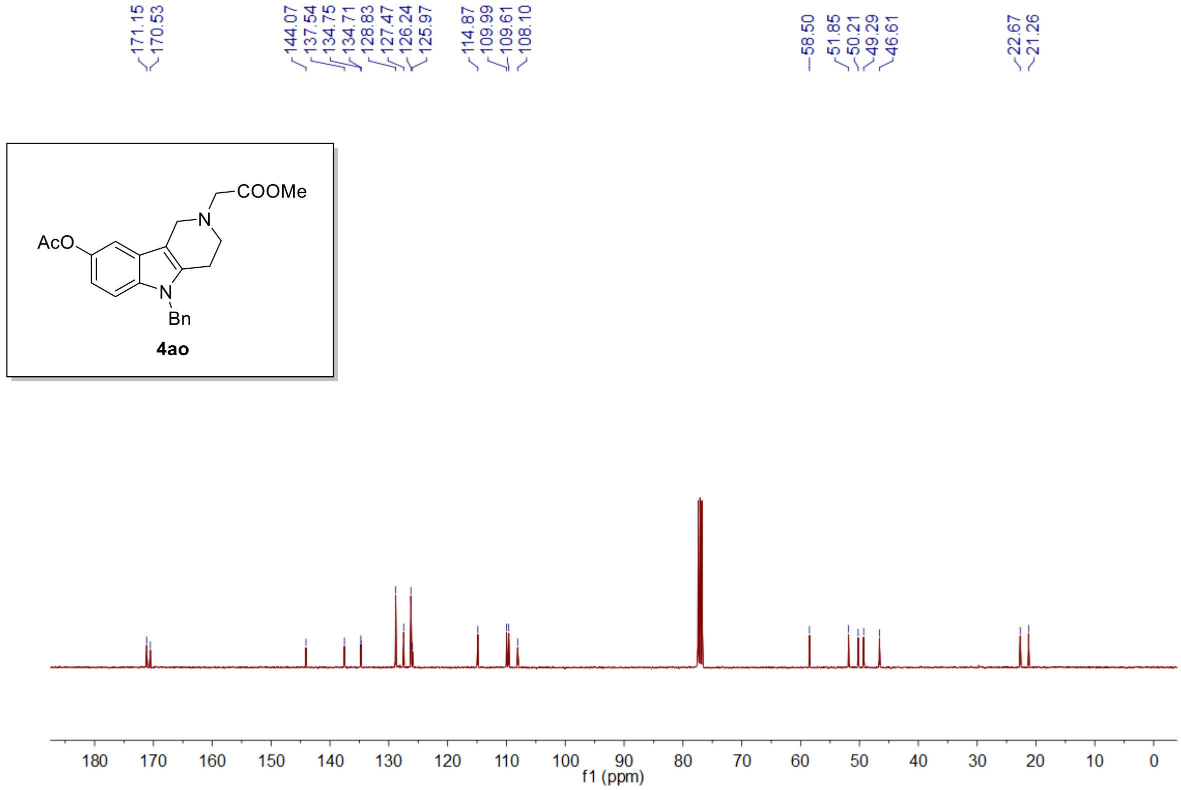

^{1}H-NMR spectrum of compound **4ap** (400 MHz, $CDCl_3$)

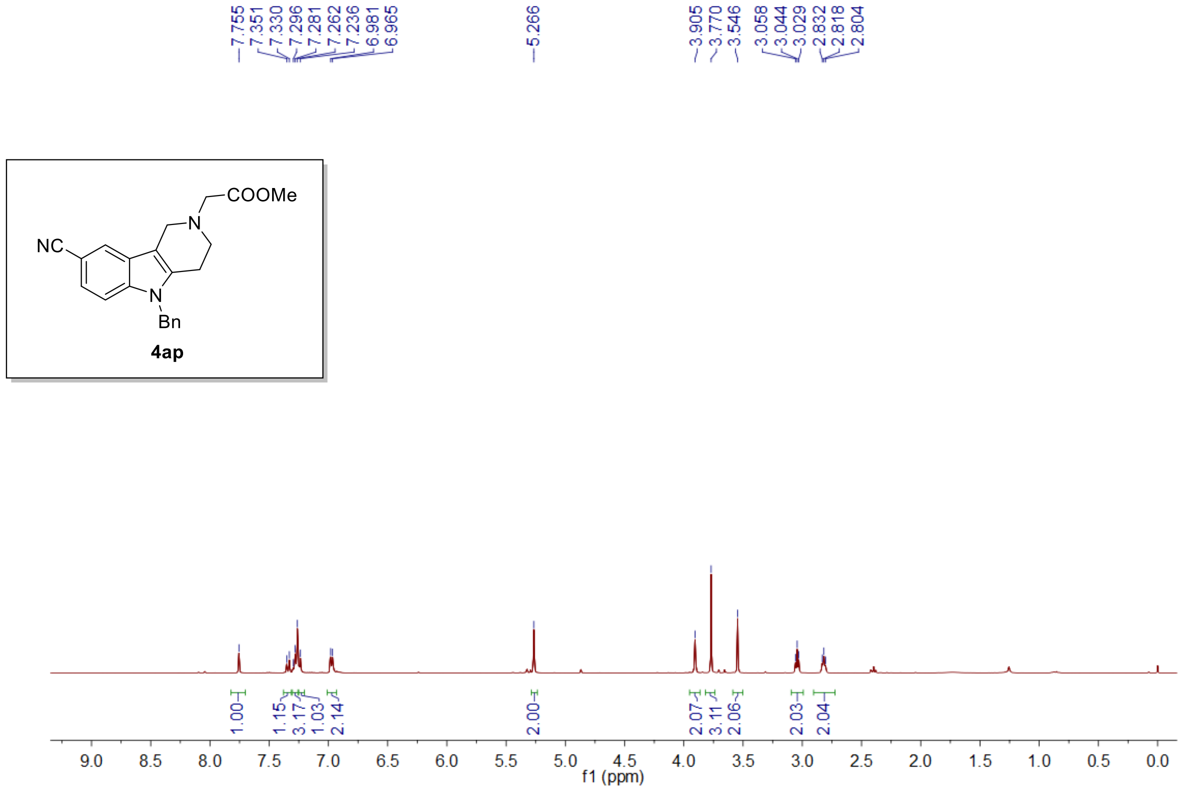

^{13}C-NMR spectrum of compound **4ap** (101 MHz, $CDCl_3$)

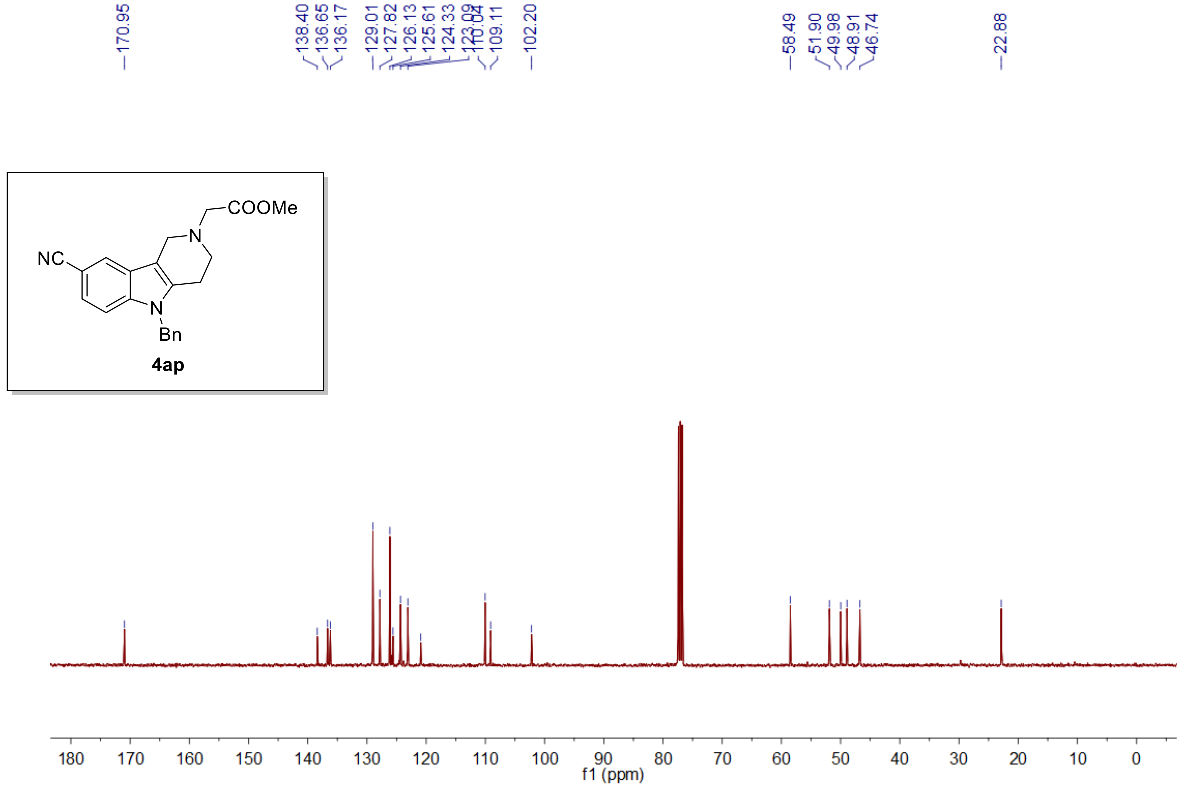

^{1}H-NMR spectrum of compound **4aq** (400 MHz, $CDCl_3$)

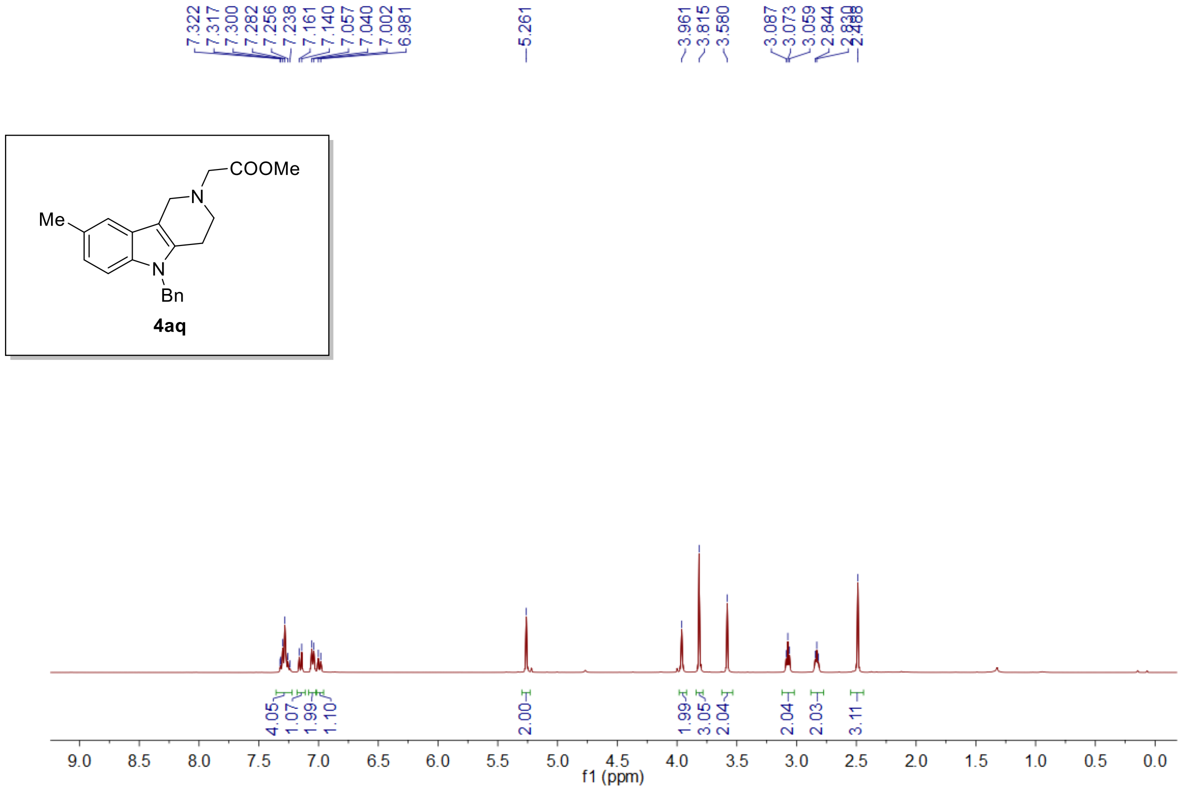

^{13}C-NMR spectrum of compound **4aq** (101 MHz, $CDCl_3$)

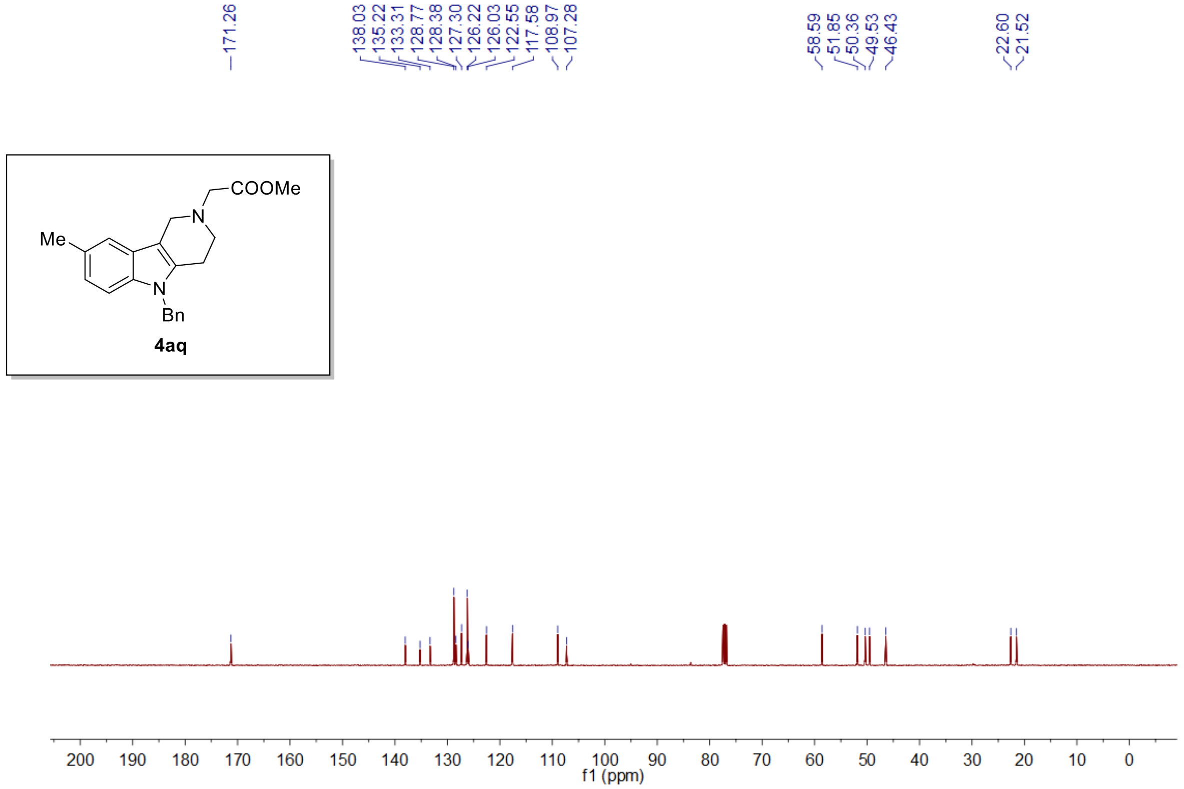

^{1}H-NMR spectrum of compound **4ar** (400 MHz, $CDCl_3$)

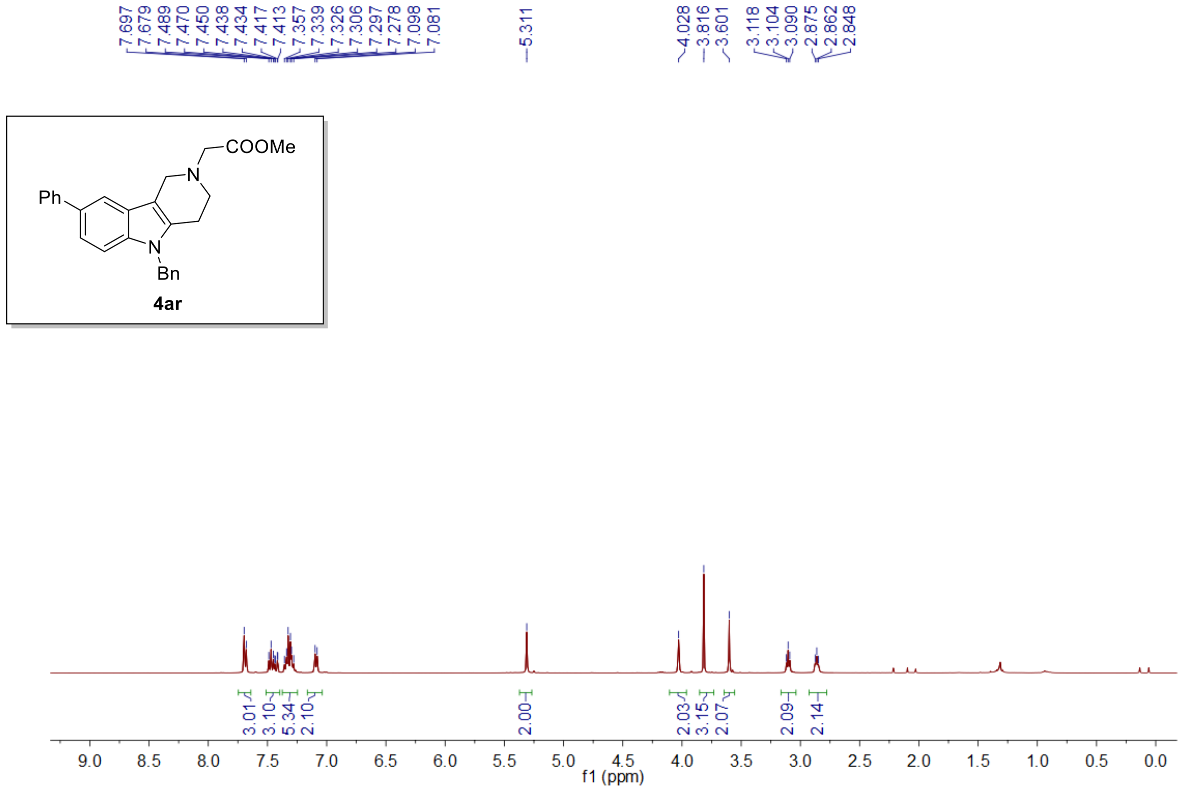

^{13}C-NMR spectrum of compound **4ar** (101 MHz, $CDCl_3$)

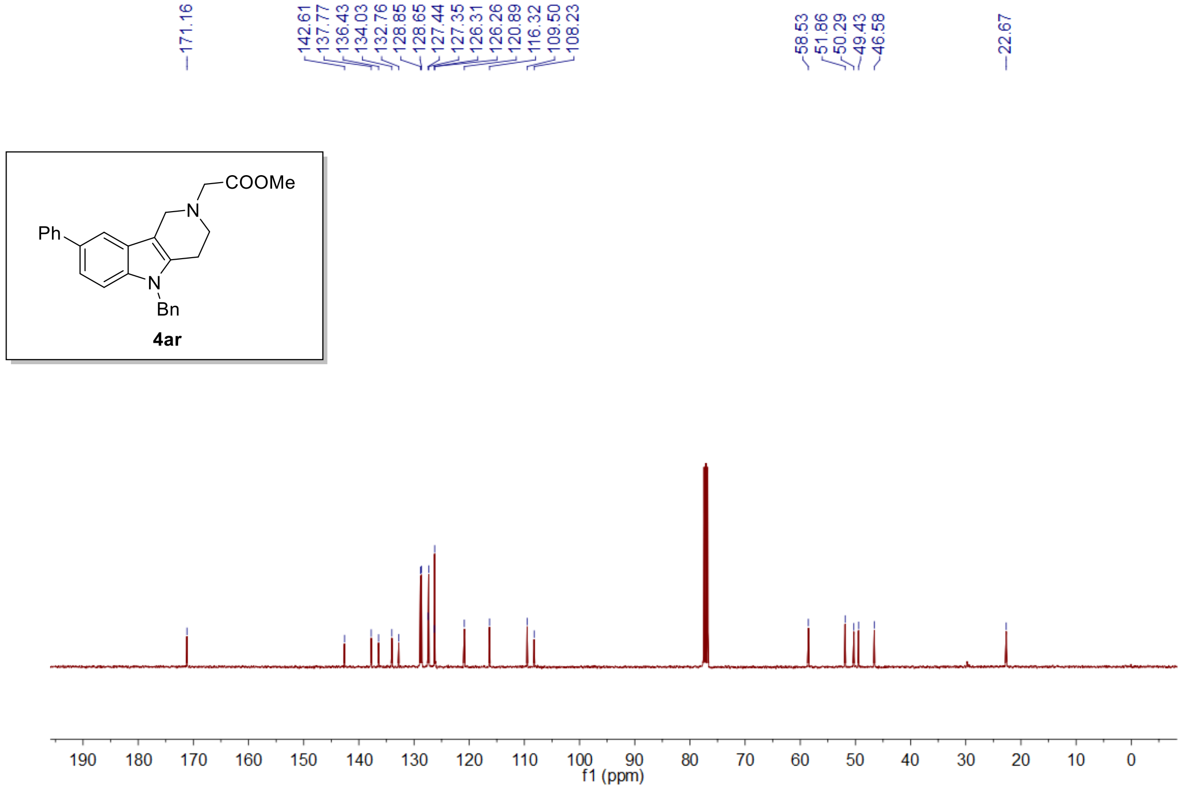

^{1}H-NMR spectrum of compound **4as** (400 MHz, $CDCl_3$)

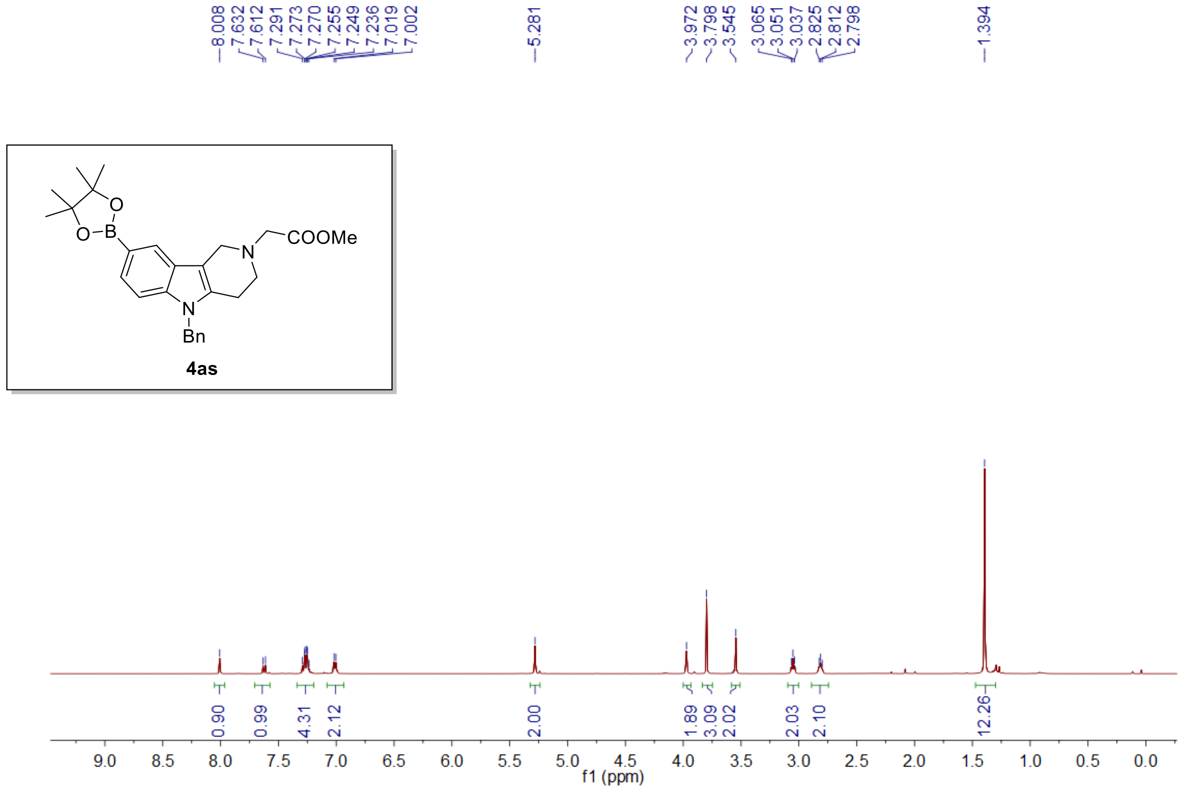

^{13}C-NMR spectrum of compound **4as** (101 MHz, $CDCl_3$)

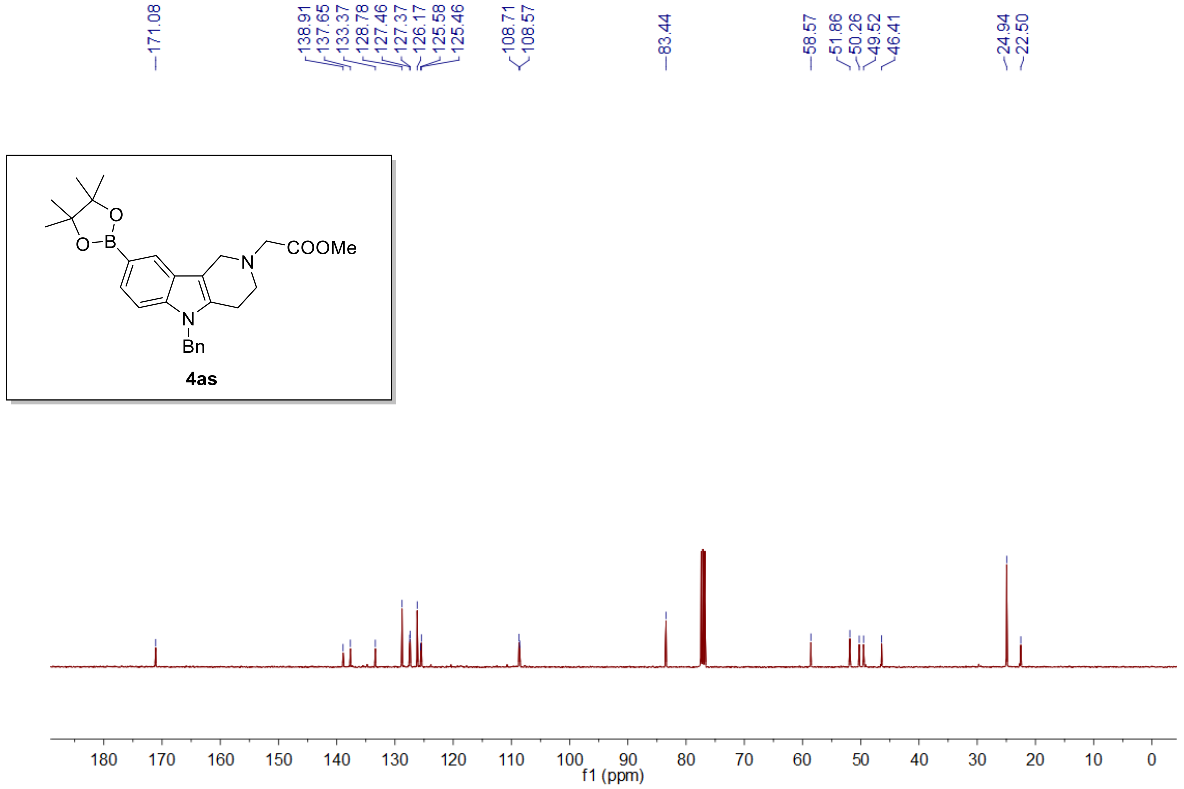

^{1}H-NMR spectrum of compound **4at** (400 MHz, $CDCl_3$)

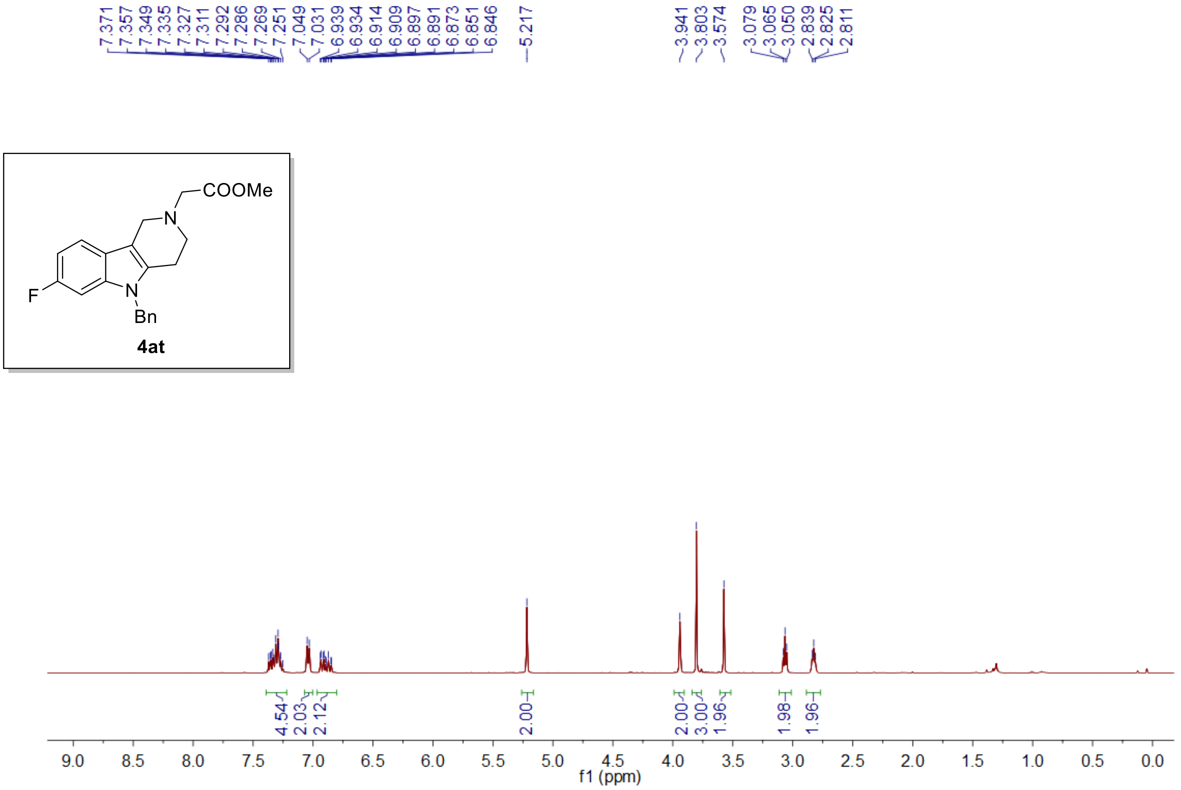

^{13}C-NMR spectrum of compound **4at** (101 MHz, $CDCl_3$)

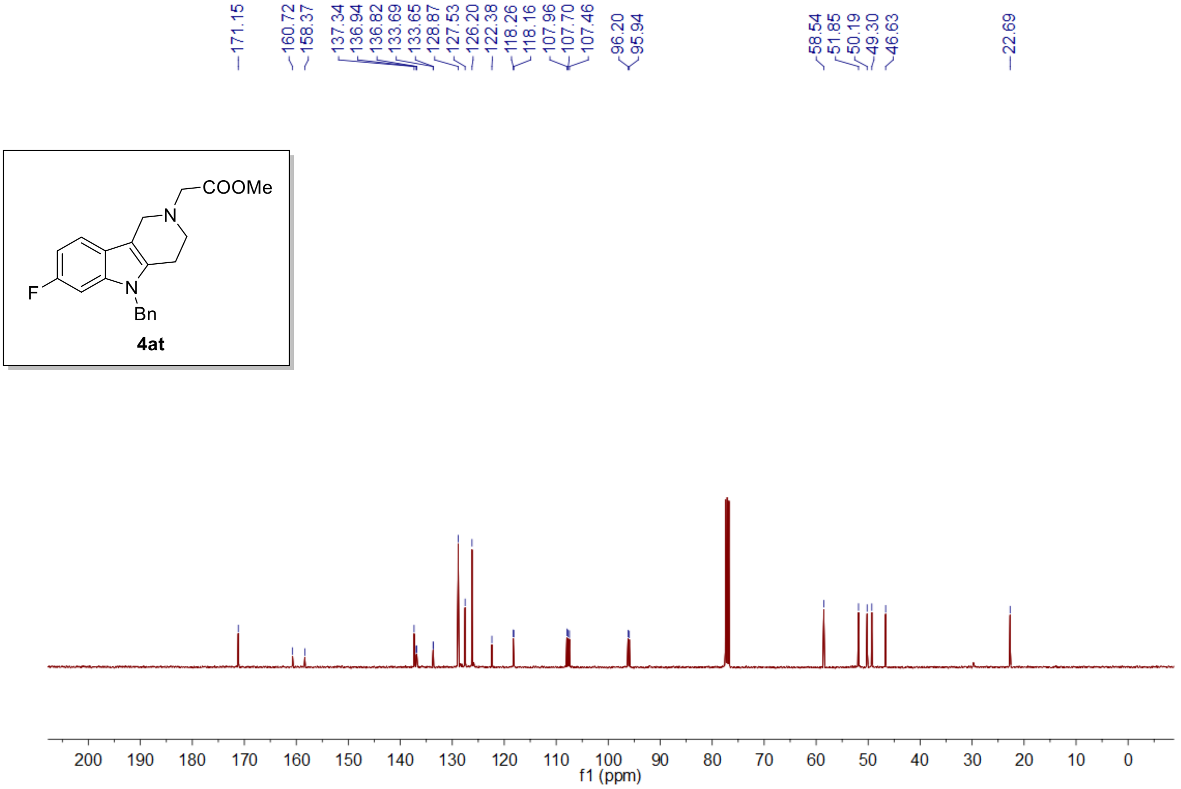

^{19}F-NMR spectrum of compound **4at** (565 MHz, $CDCl_3$)

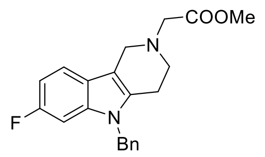

4at

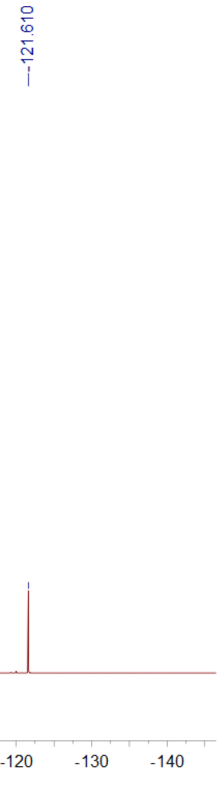

^{1}H-NMR spectrum of compound **4au** (400 MHz, $CDCl_3$)

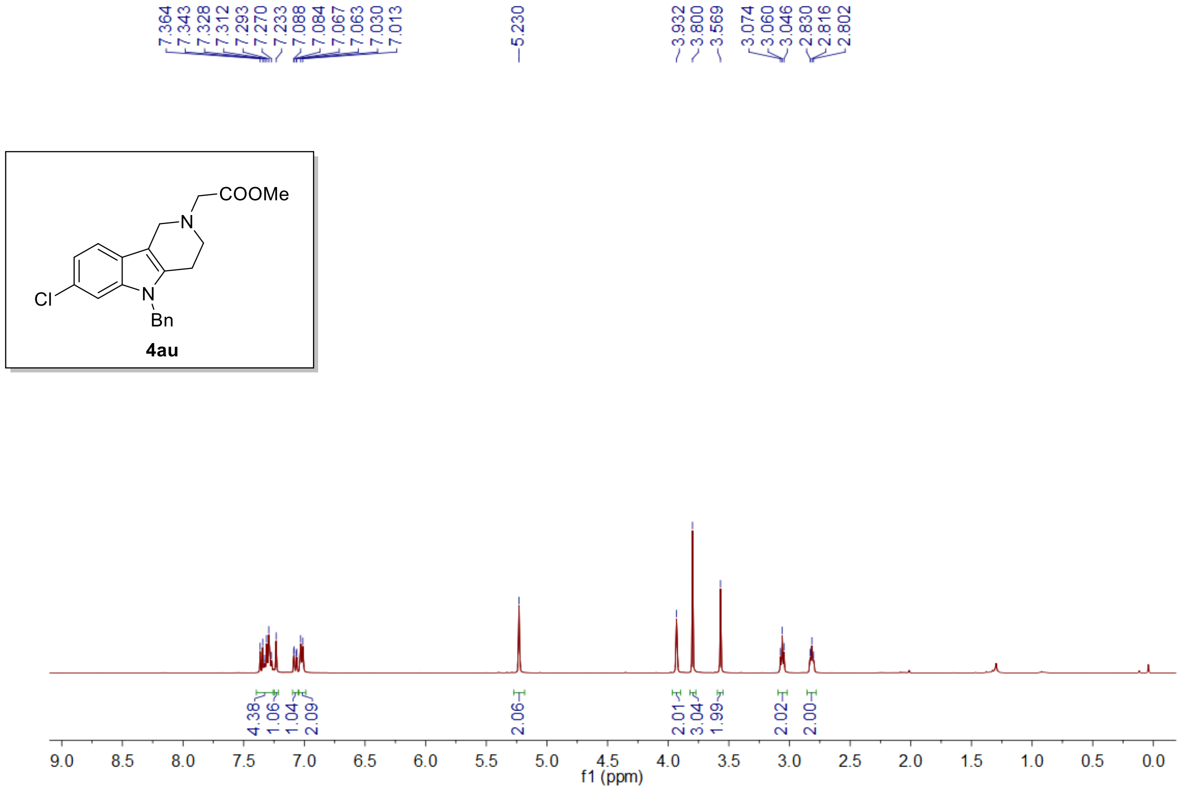

^{13}C-NMR spectrum of compound **4au** (101 MHz, $CDCl_3$)

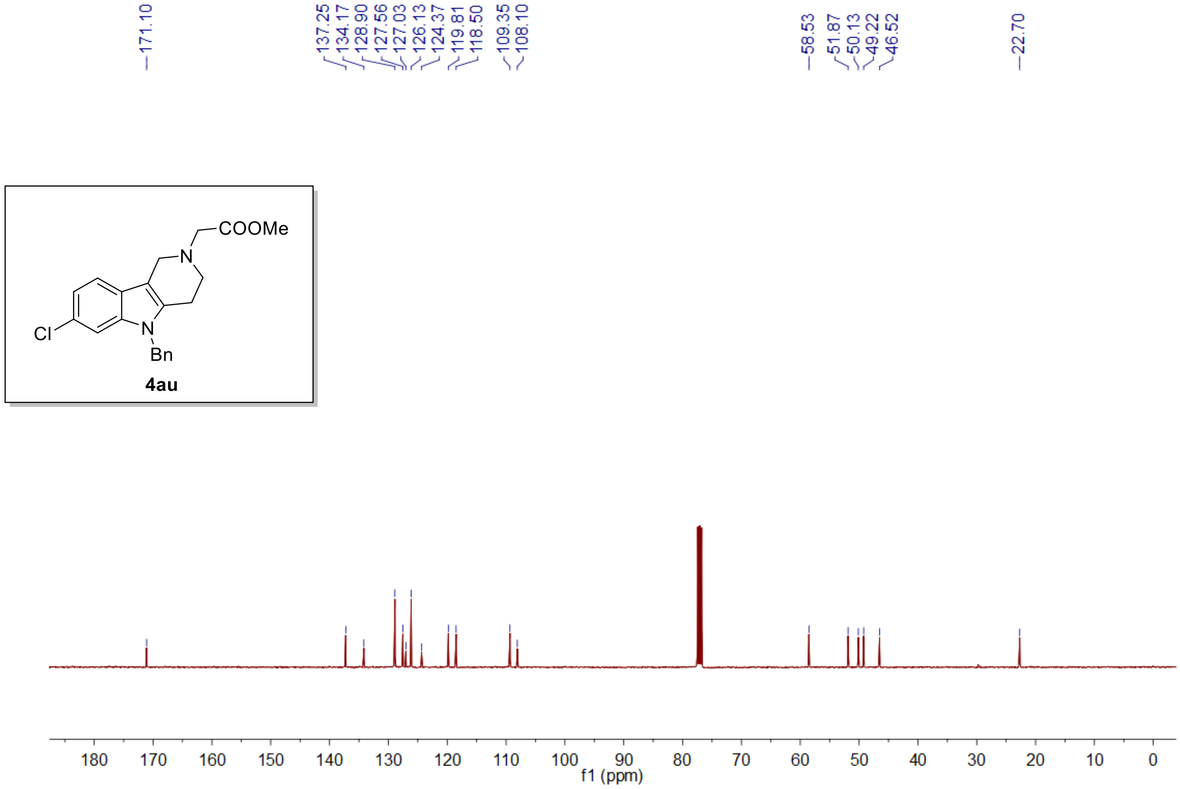

^{1}H-NMR spectrum of compound **4av** (400 MHz, $CDCl_3$)

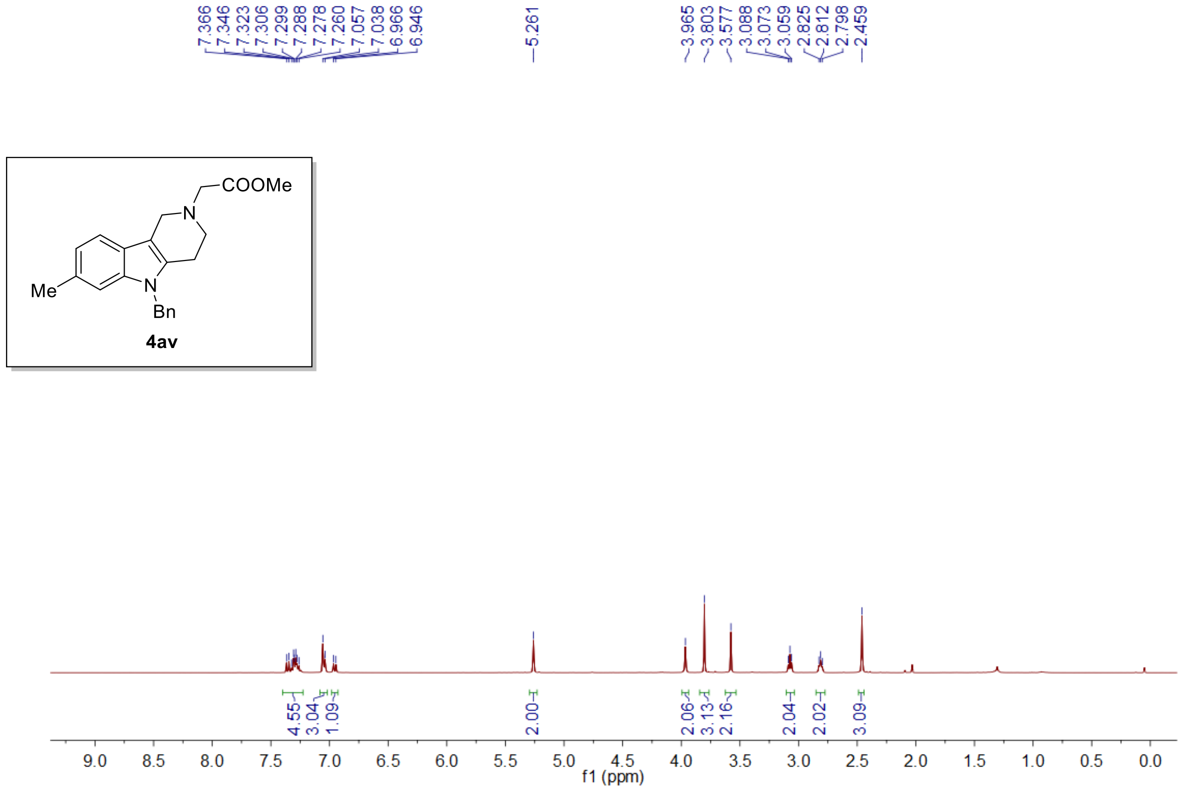

^{13}C-NMR spectrum of compound **4av** (101 MHz, $CDCl_3$)

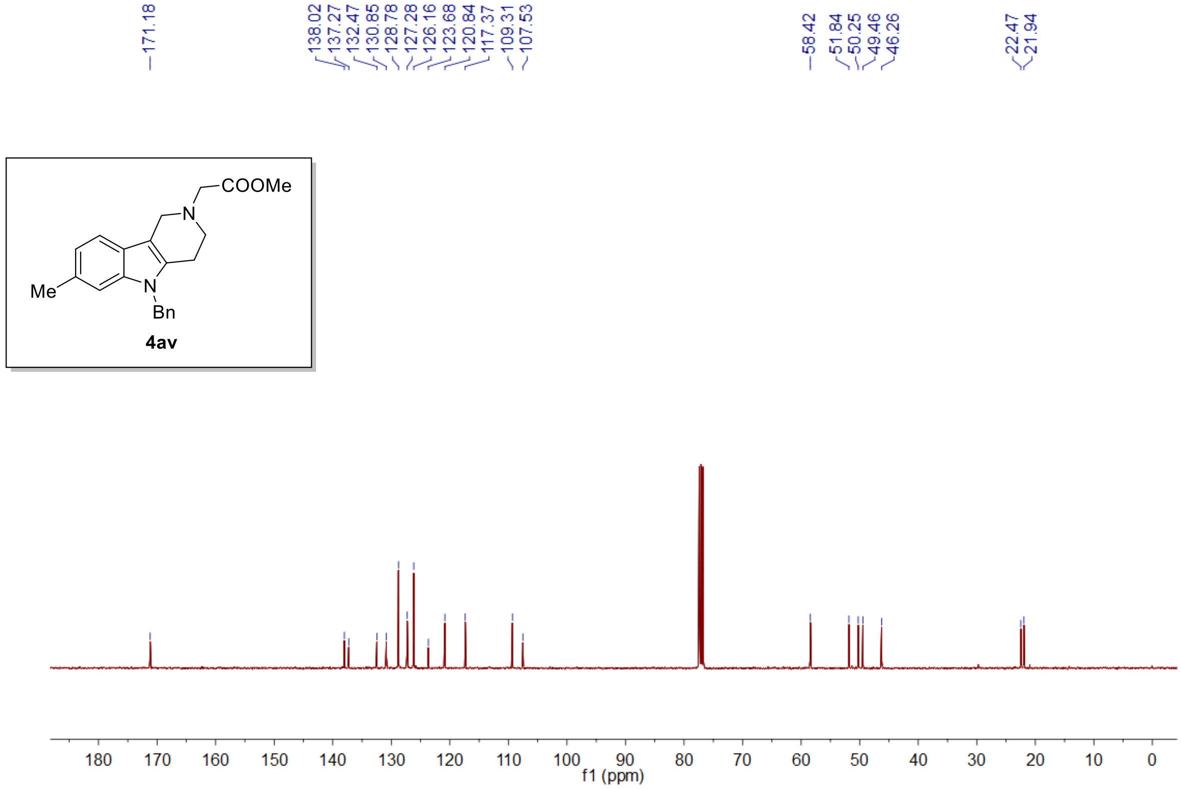

^{1}H-NMR spectrum of compound **4aw** (400 MHz, $CDCl_3$)

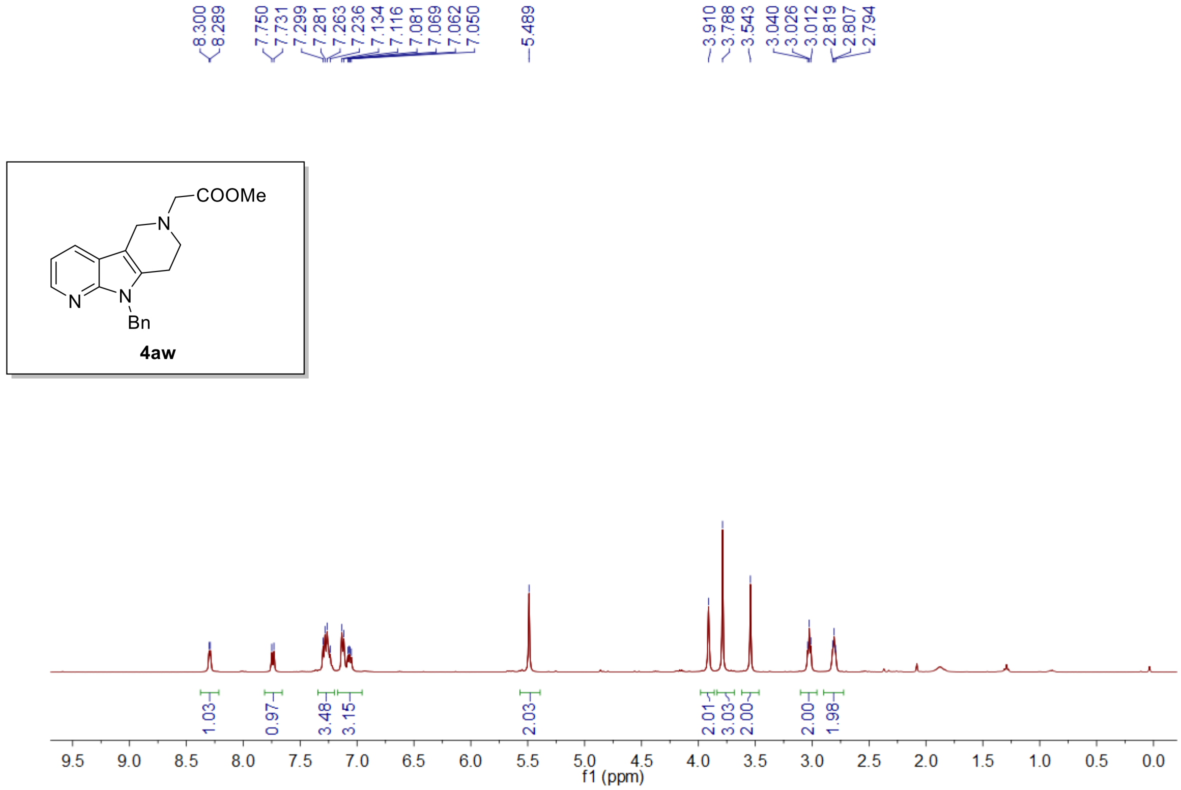

^{13}C-NMR spectrum of compound **4aw** (101 MHz, $CDCl_3$)

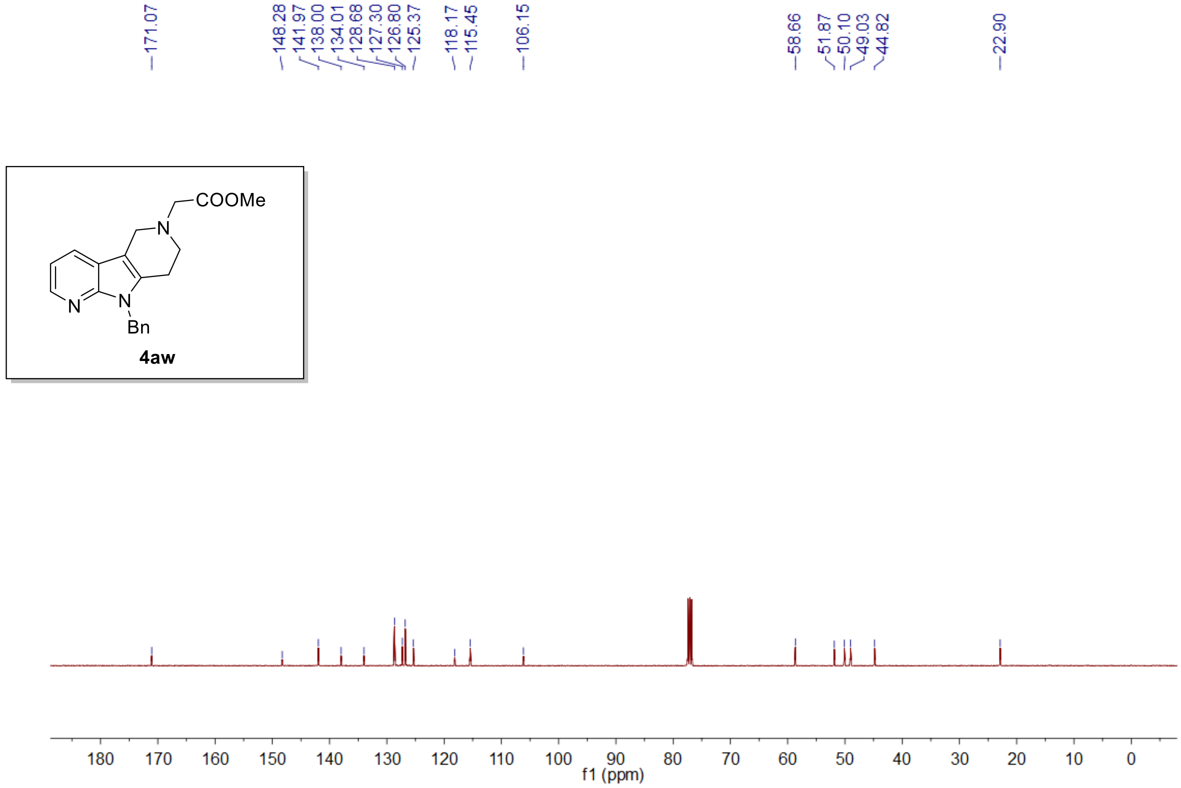

^{1}H-NMR spectrum of compound **4ax** (400 MHz, $CDCl_3$)

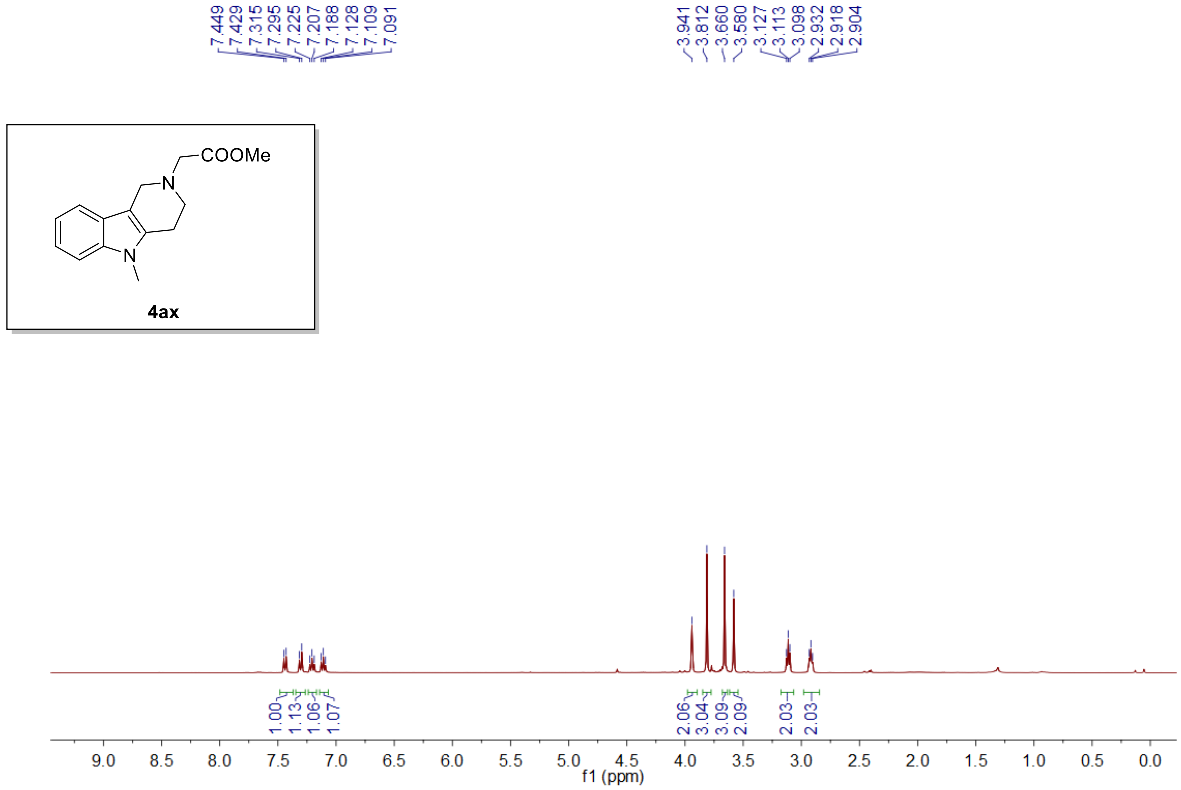

^{13}C-NMR spectrum of compound **4ax** (101 MHz, $CDCl_3$)

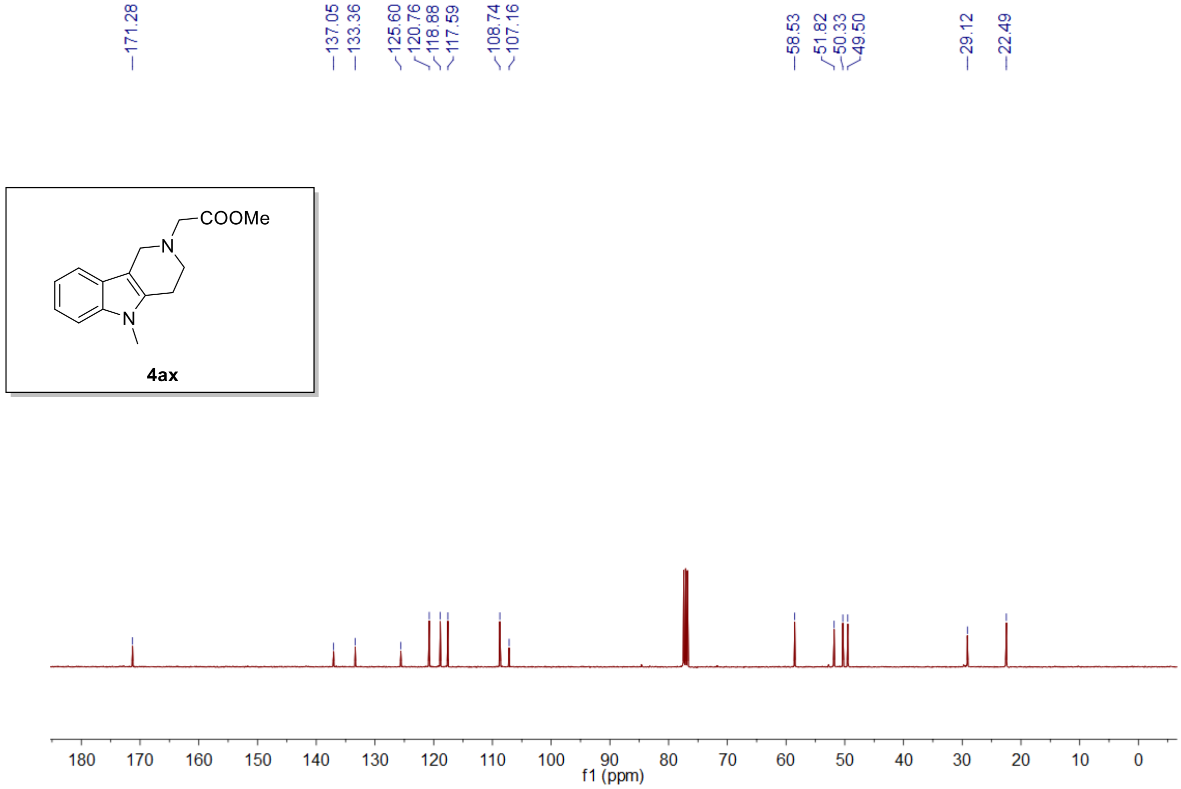

^{1}H-NMR spectrum of compound **4ay** (400 MHz, $CDCl_3$)

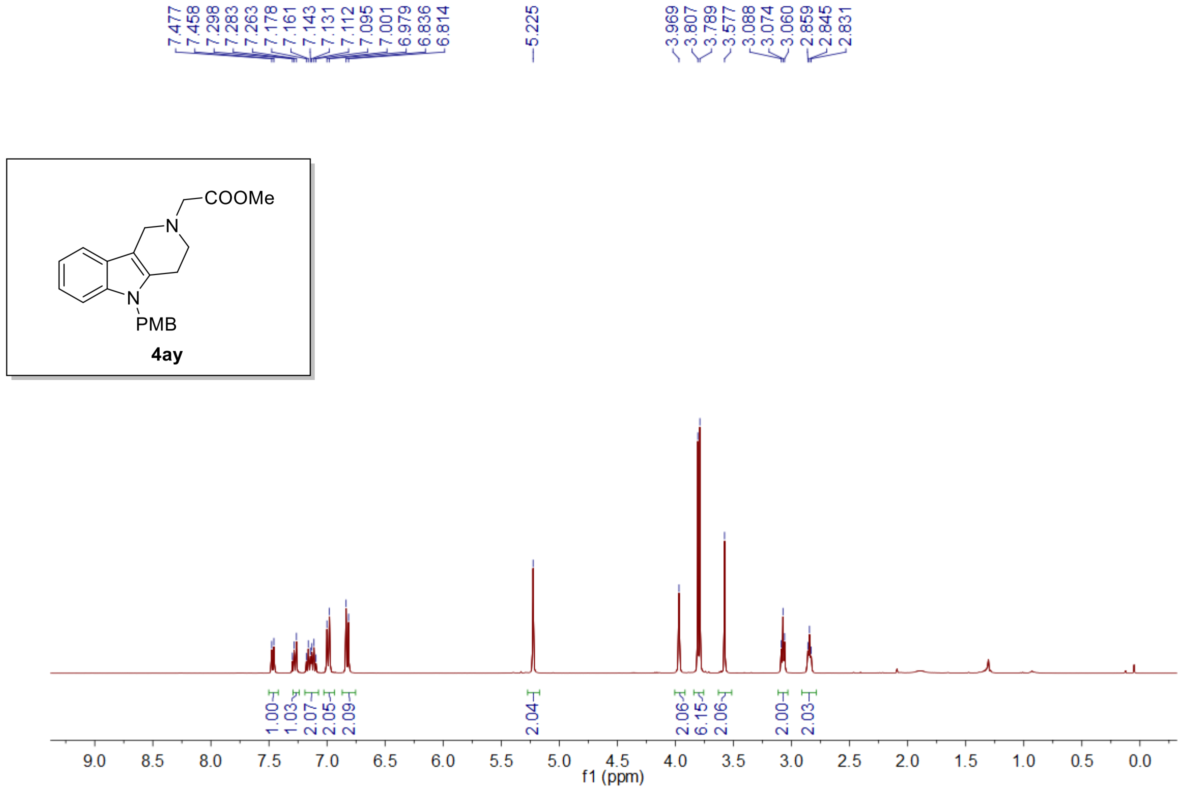

^{13}C-NMR spectrum of compound **4ay** (101 MHz, $CDCl_3$)

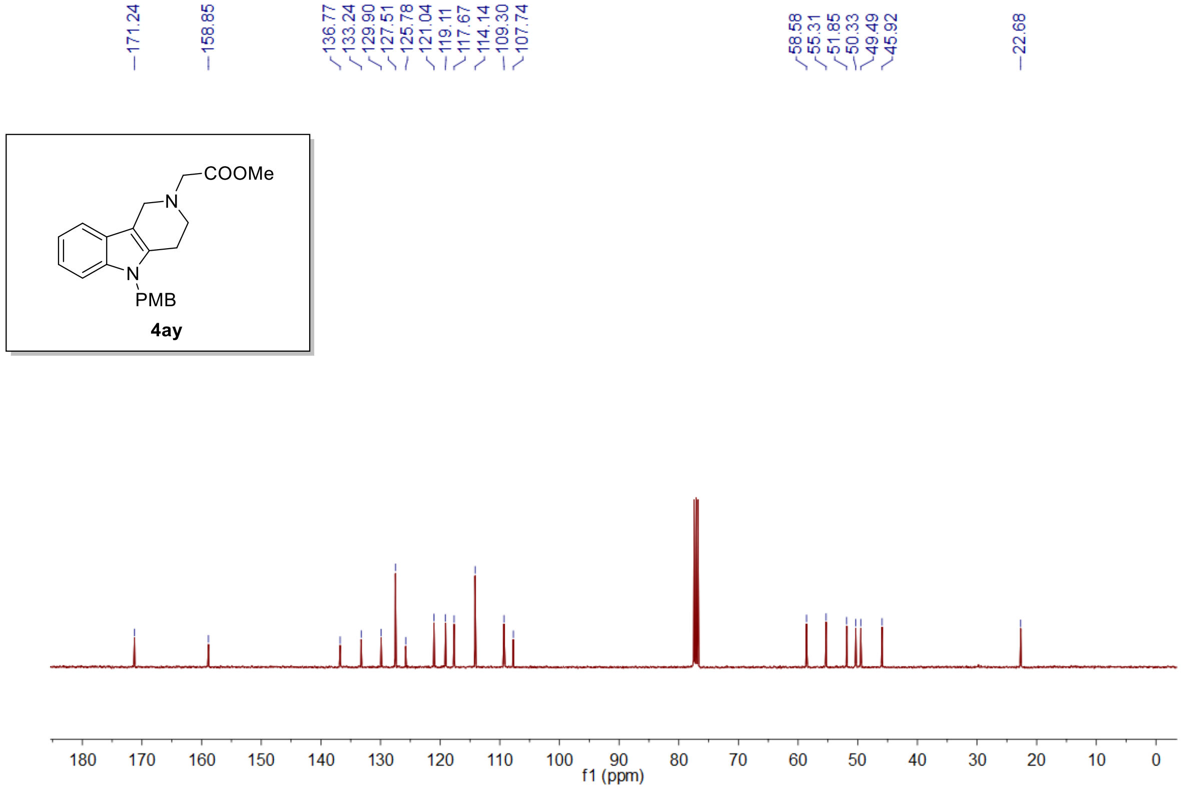

^{1}H-NMR spectrum of compound **4az** (400 MHz, $CDCl_3$)

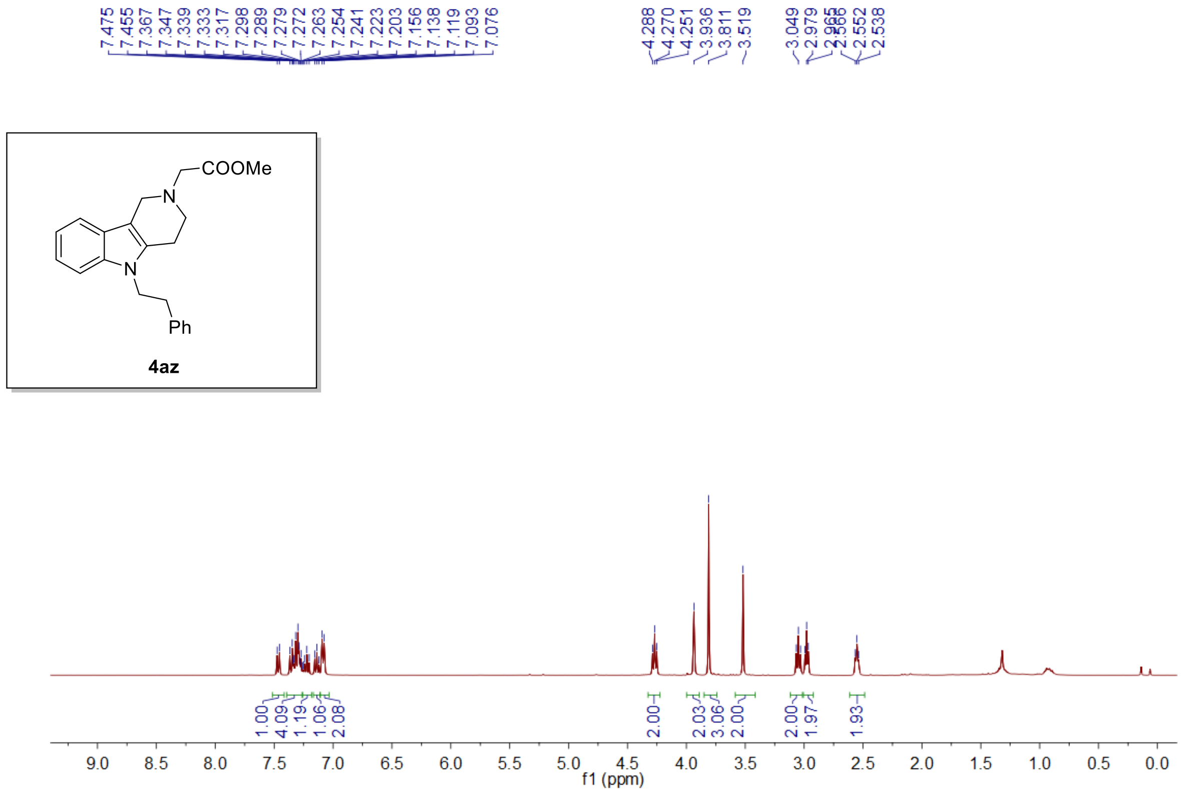

^{13}C-NMR spectrum of compound **4az** (101 MHz, $CDCl_3$)

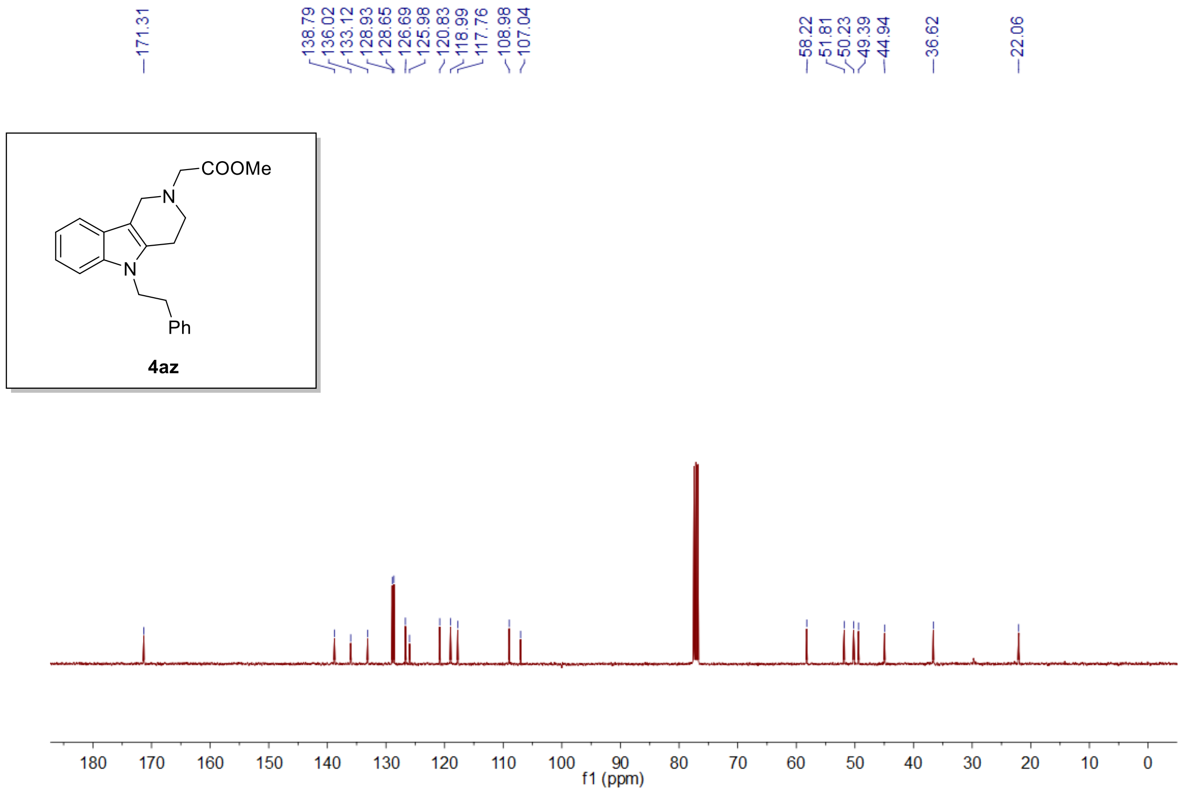

^{1}H-NMR spectrum of compound **4aaa** (400 MHz, $CDCl_3$)

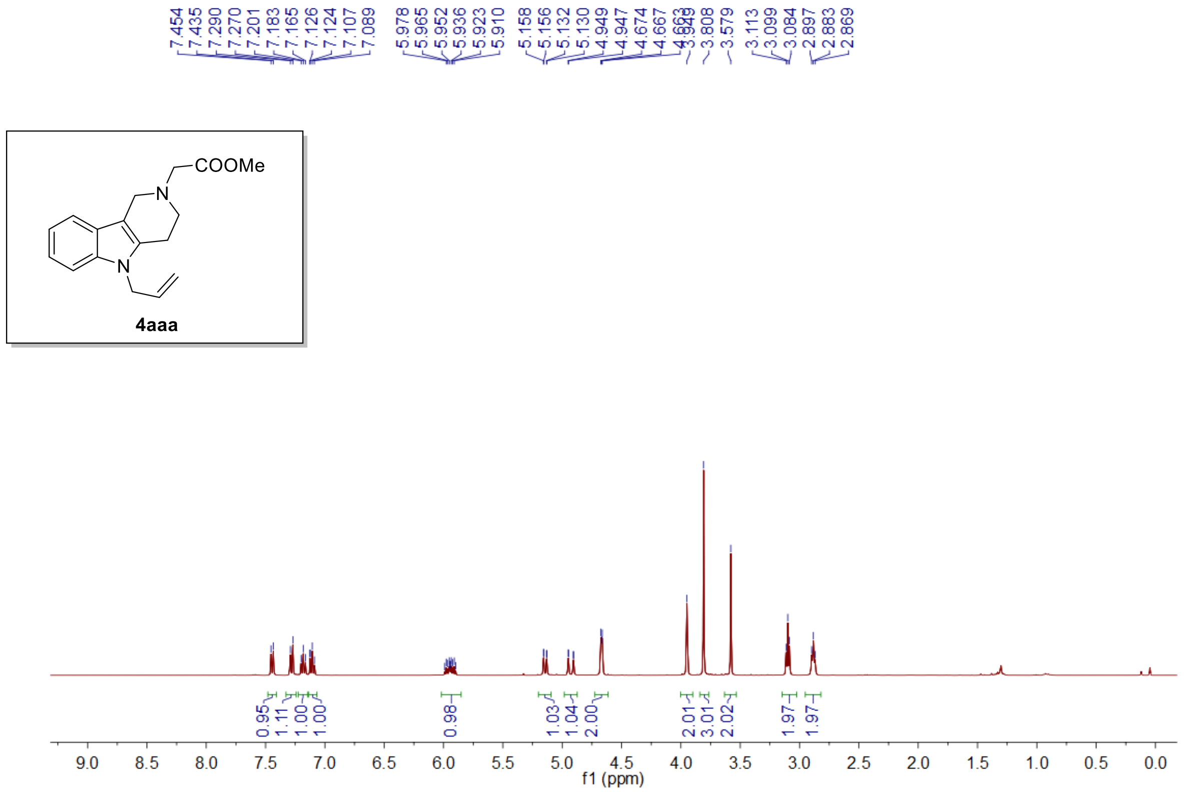

^{13}C-NMR spectrum of compound **4aaa** (101 MHz, $CDCl_3$)

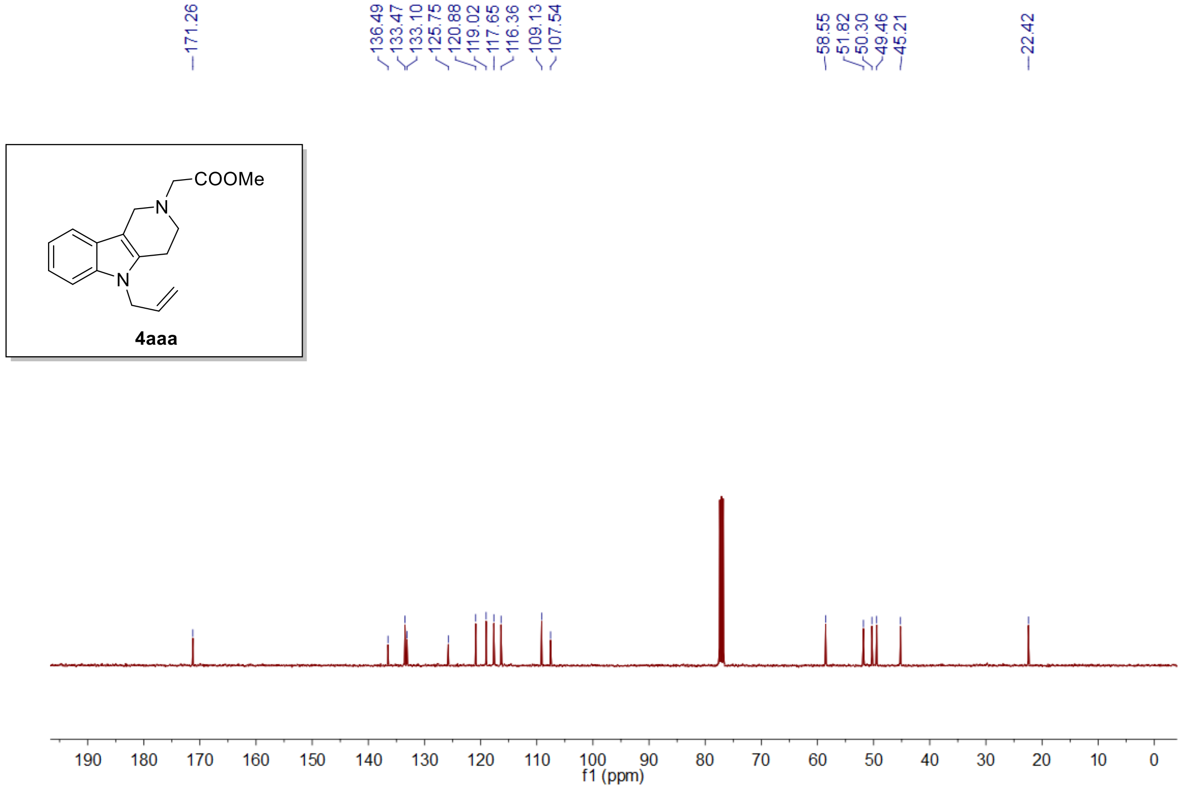

^{1}H-NMR spectrum of compound **4aab** (400 MHz, $CDCl_3$)

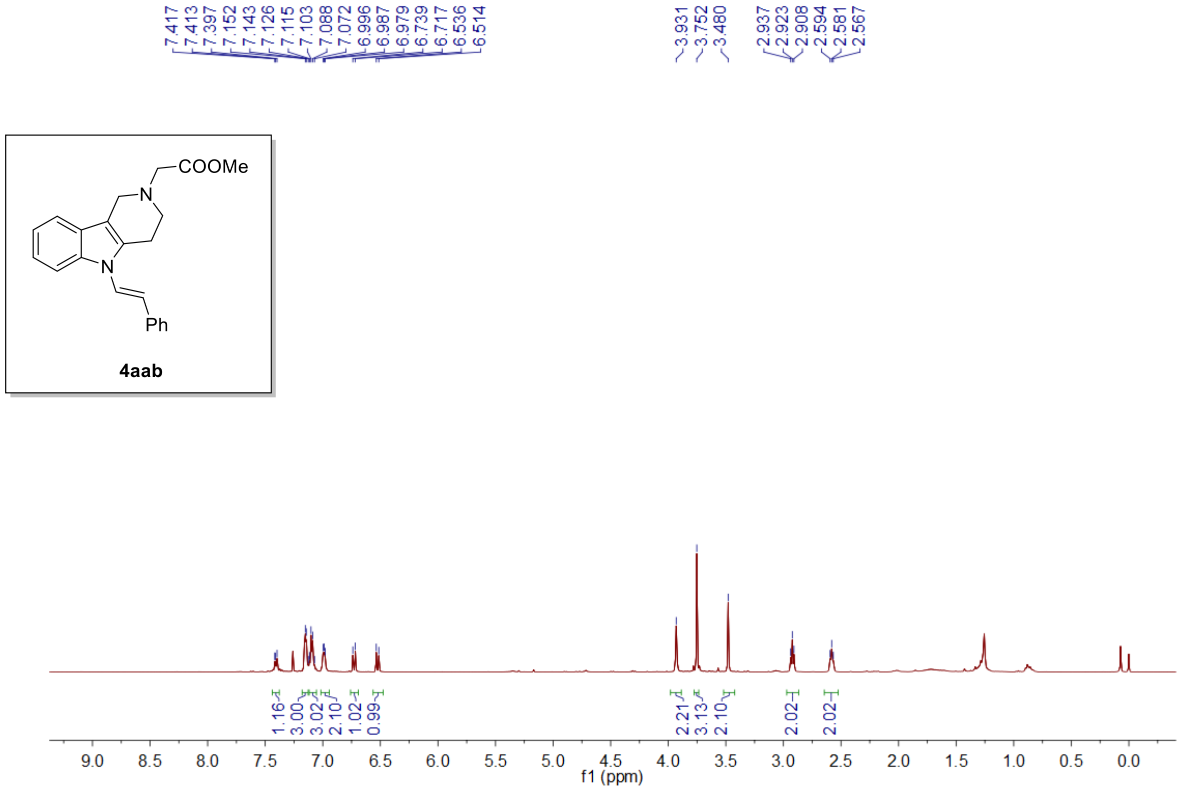

^{13}C-NMR spectrum of compound **4aab** (101 MHz, $CDCl_3$)

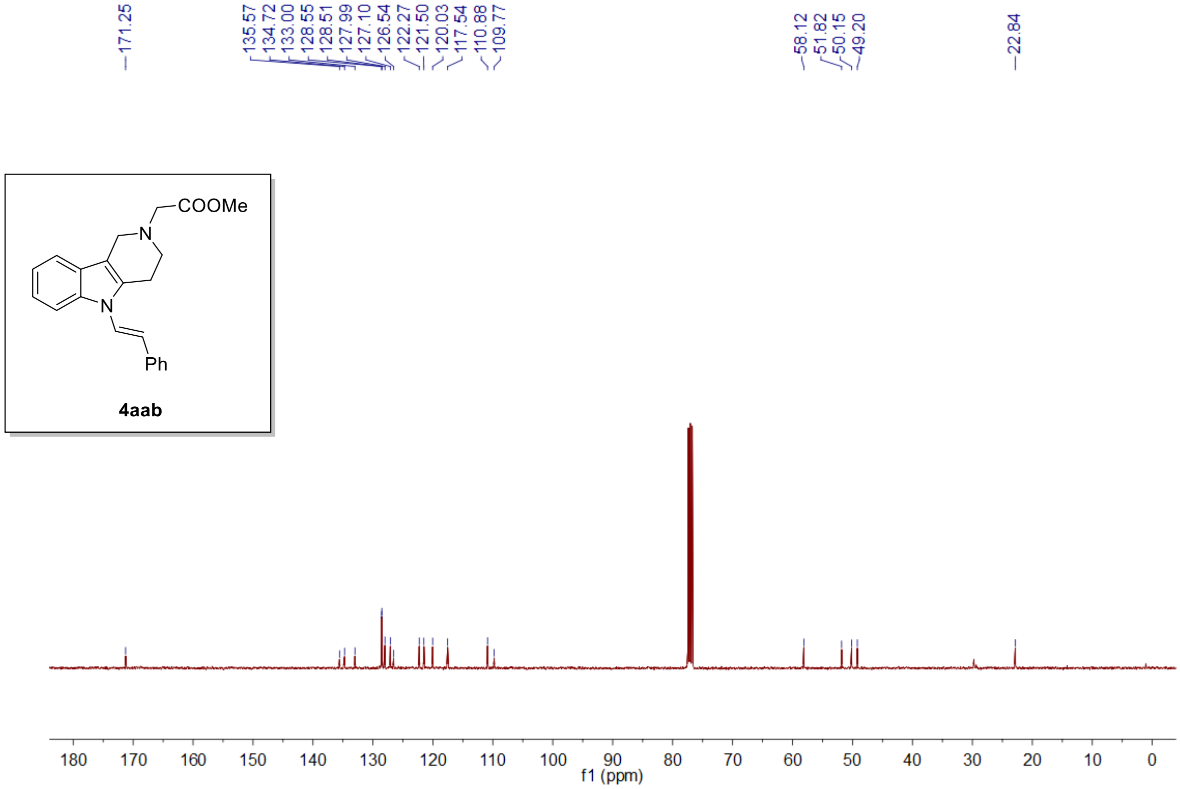

^{1}H-NMR spectrum of compound **4aac** (400 MHz, $CDCl_3$)

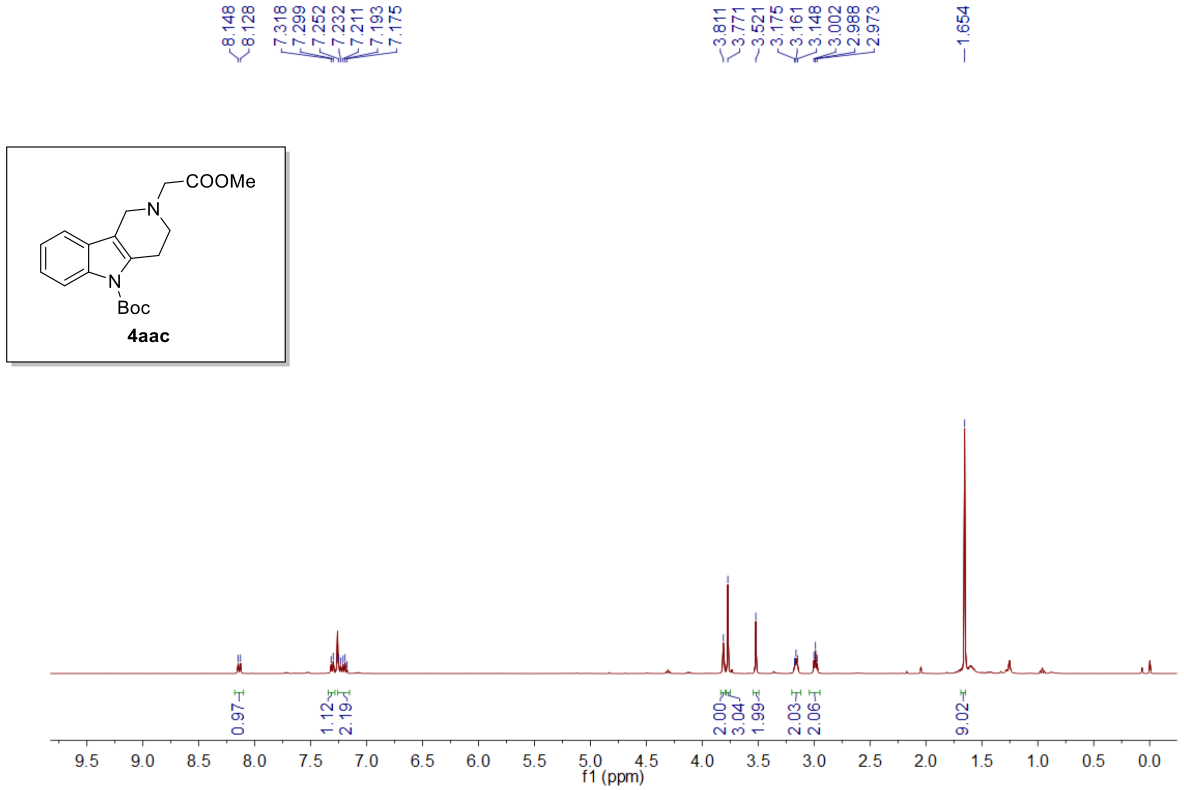

^{13}C-NMR spectrum of compound **4aac** (101 MHz, $CDCl_3$)

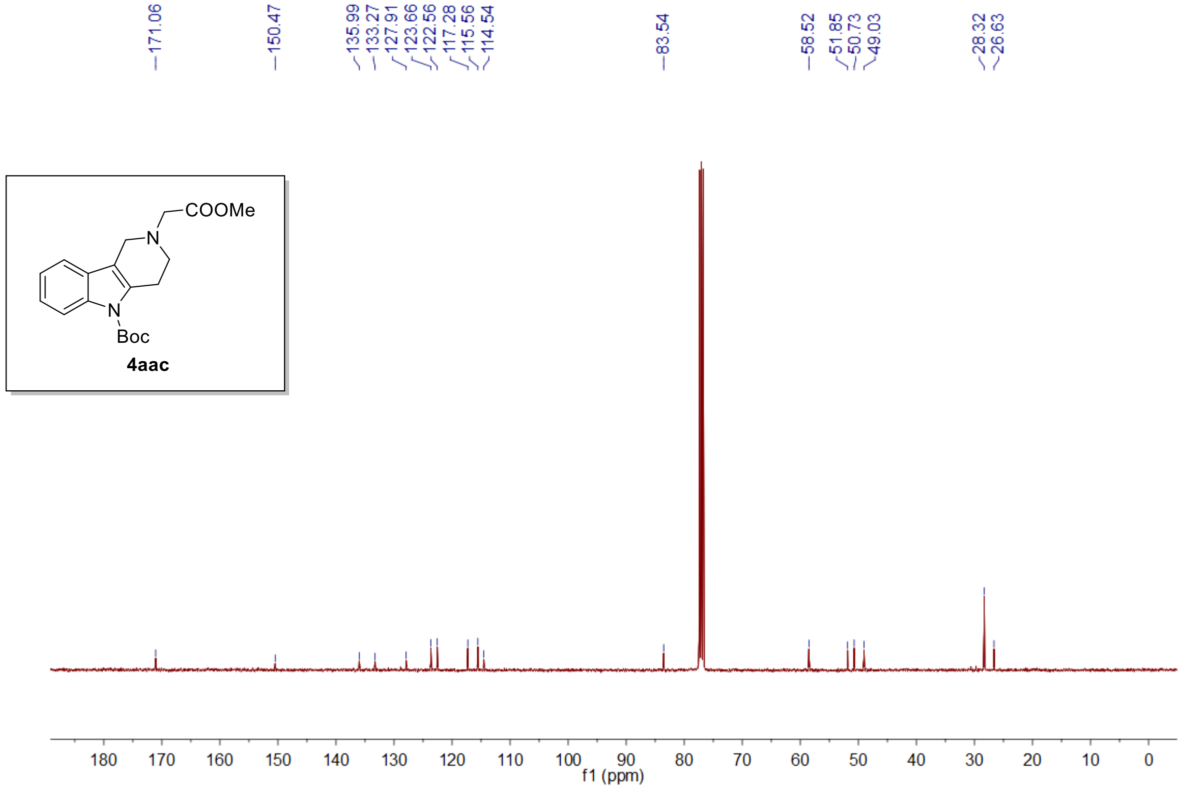

^{1}H-NMR spectrum of compound **4aad** (400 MHz, $CDCl_3$)

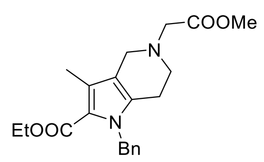

4aad

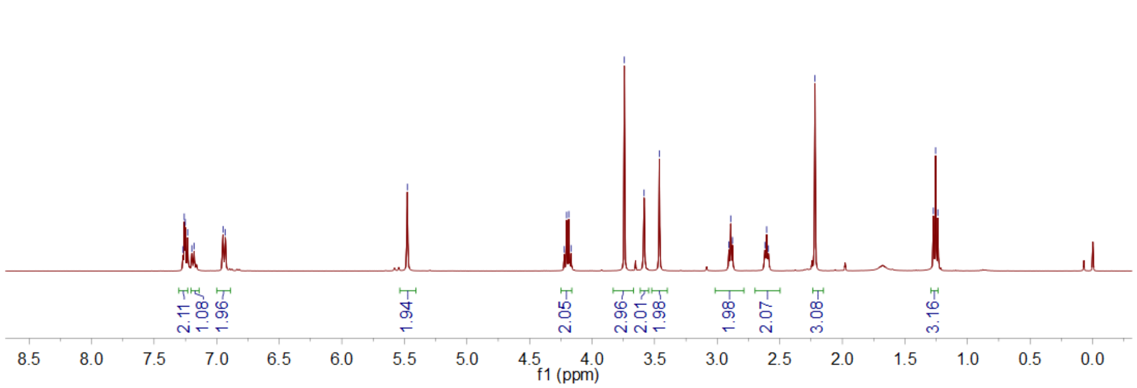

^{13}C-NMR spectrum of compound **4aad** (101 MHz, $CDCl_3$)

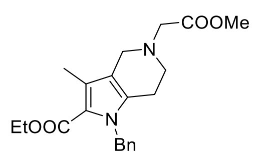

4aad

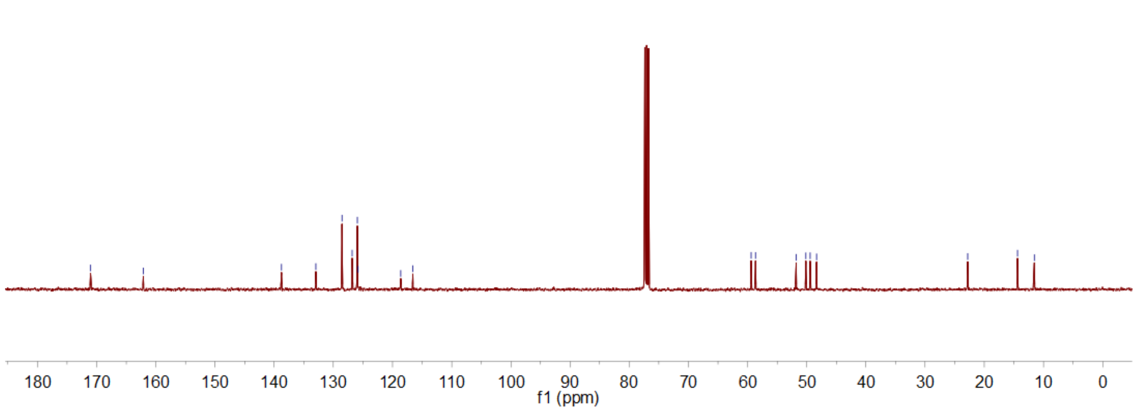

^{1}H-NMR spectrum of compound **4aae** (400 MHz, $CDCl_3$)

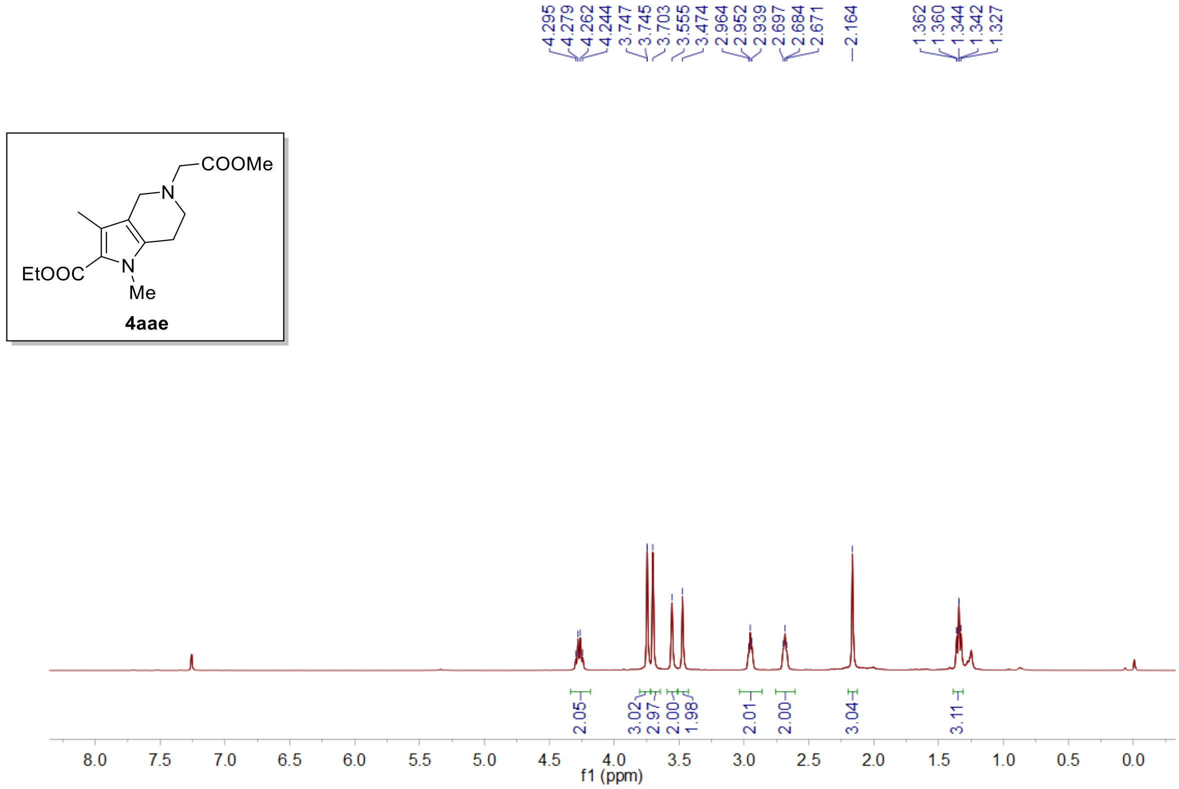

^{13}C-NMR spectrum of compound **4aae** (101 MHz, $CDCl_3$)

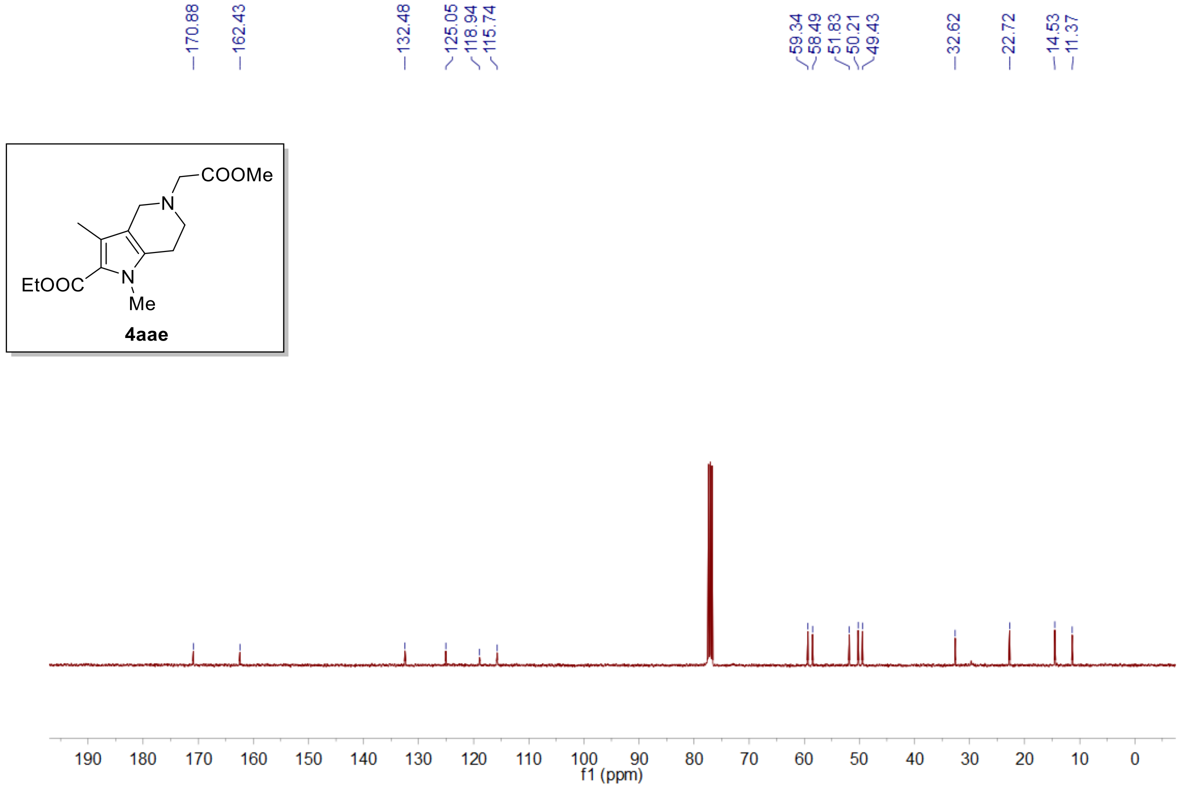

^{1}H-NMR spectrum of compound **6a** (400 MHz, $CDCl_3$)

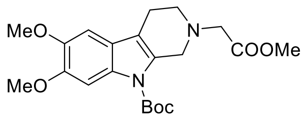

6a

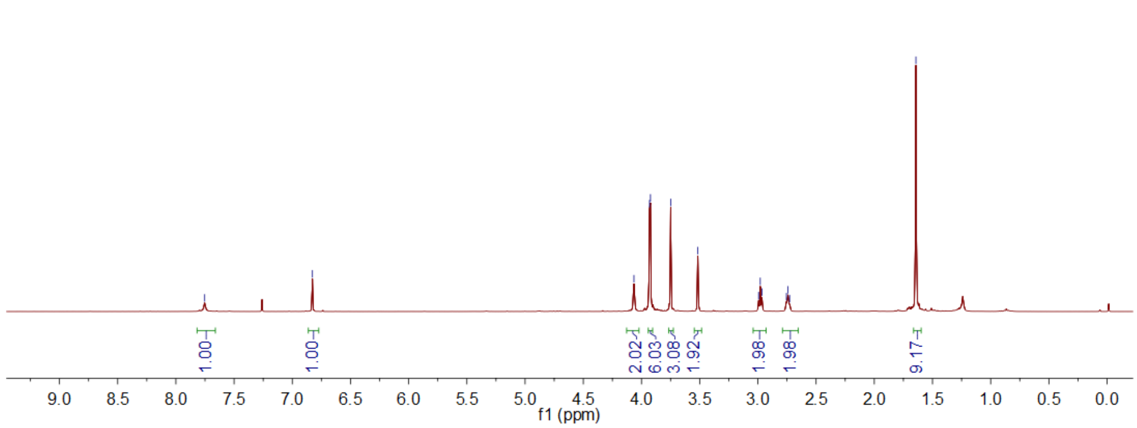

^{13}C-NMR spectrum of compound **6a** (101 MHz, $CDCl_3$)

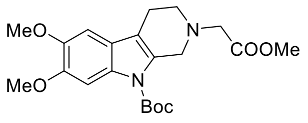

6a

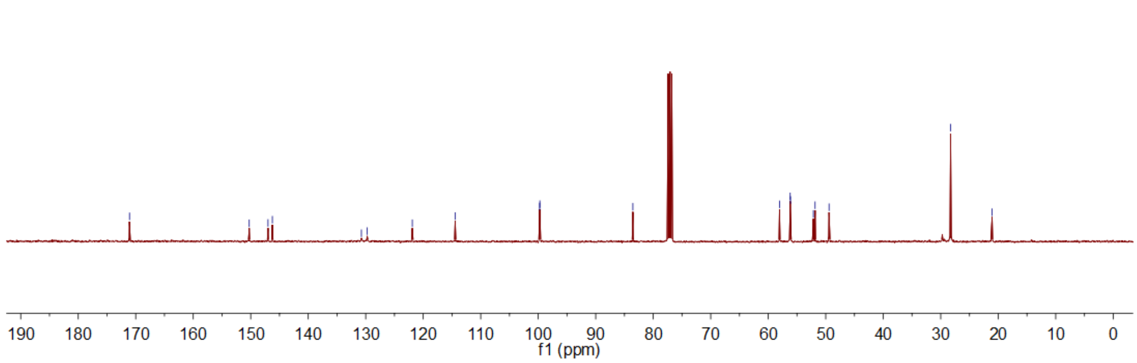

^{1}H-NMR spectrum of compound **6b** (400 MHz, $CDCl_3$)

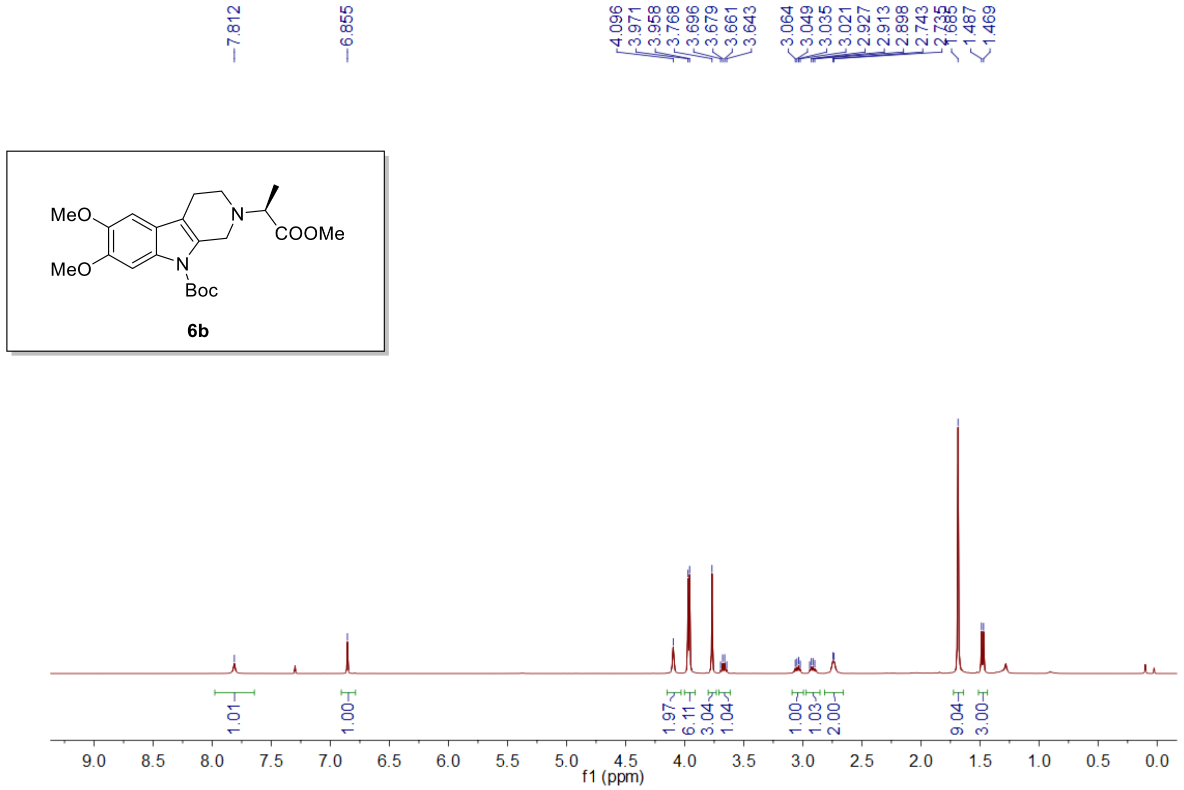

^{13}C-NMR spectrum of compound **6b** (101 MHz, $CDCl_3$)

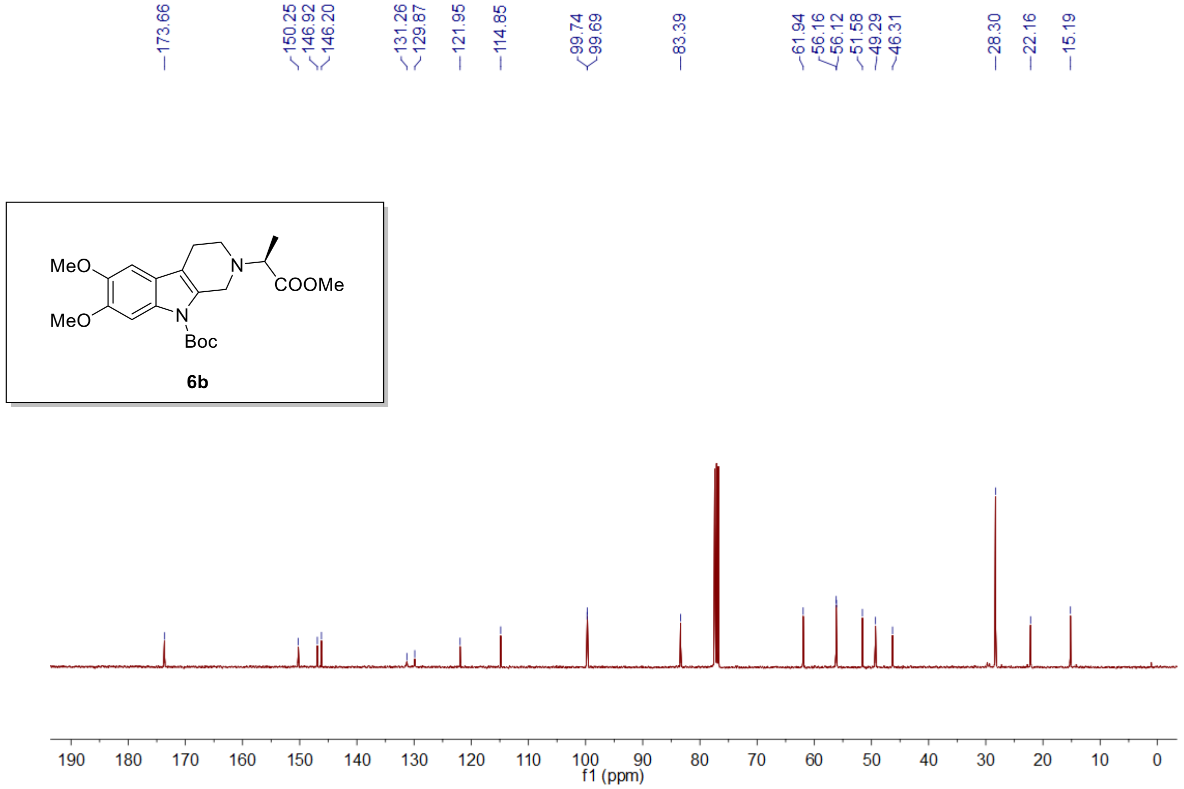

^{1}H-NMR spectrum of compound **6c** (400 MHz, $CDCl_3$)

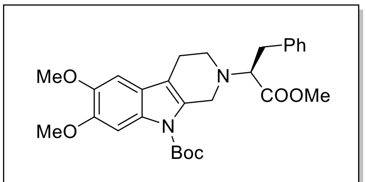

6c

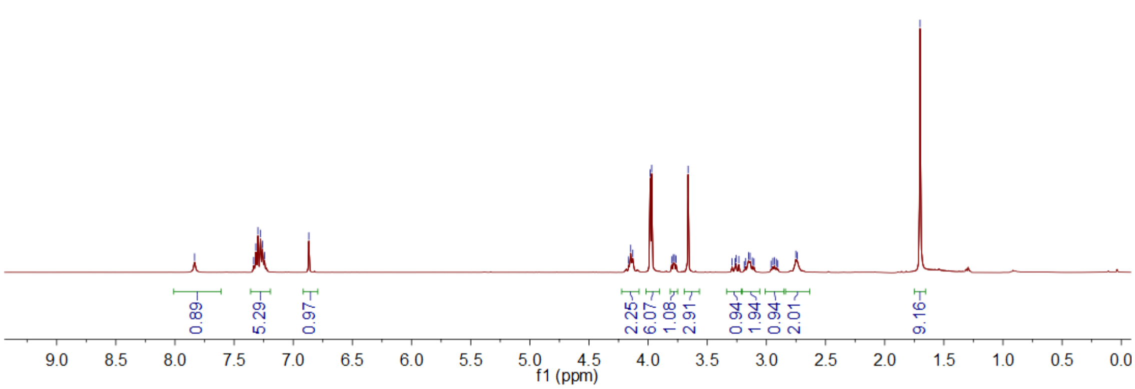

^{13}C-NMR spectrum of compound **6c** (101 MHz, $CDCl_3$)

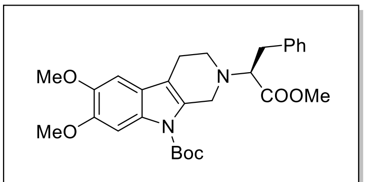

6c

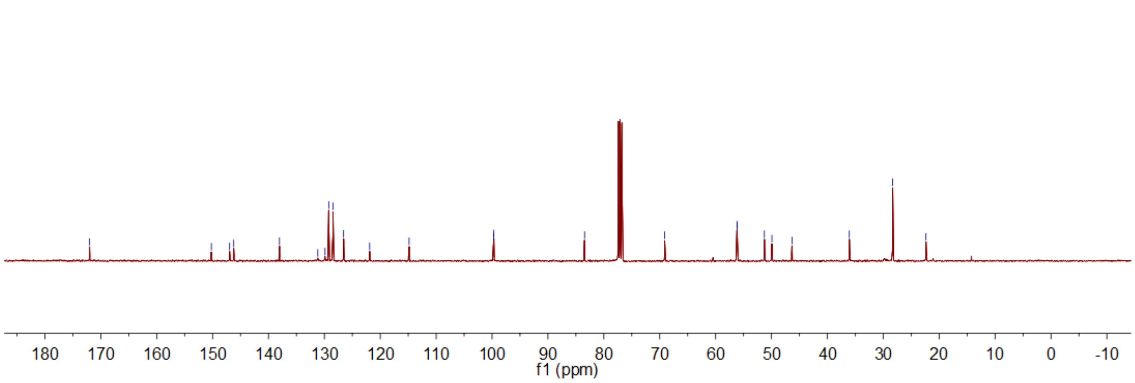

^{1}H-NMR spectrum of compound **6d** (400 MHz, $CDCl_3$)

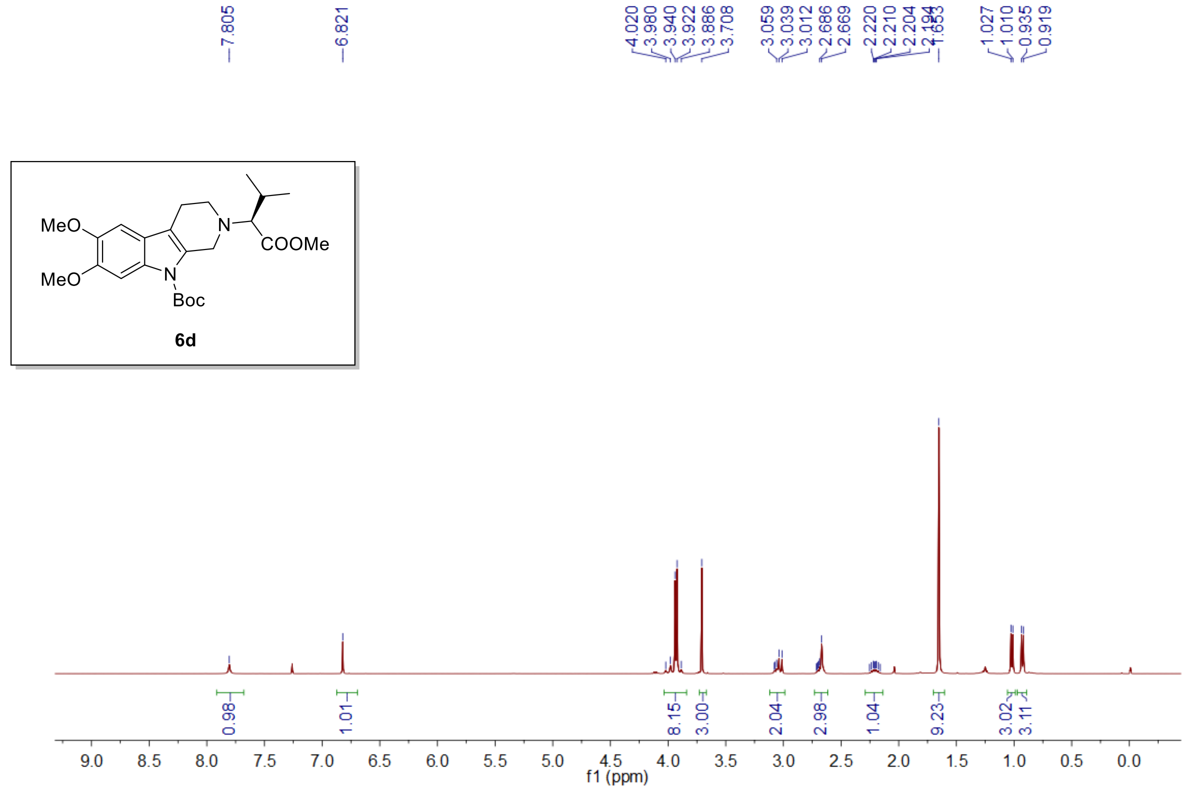

^{13}C-NMR spectrum of compound **6d** (101 MHz, $CDCl_3$)

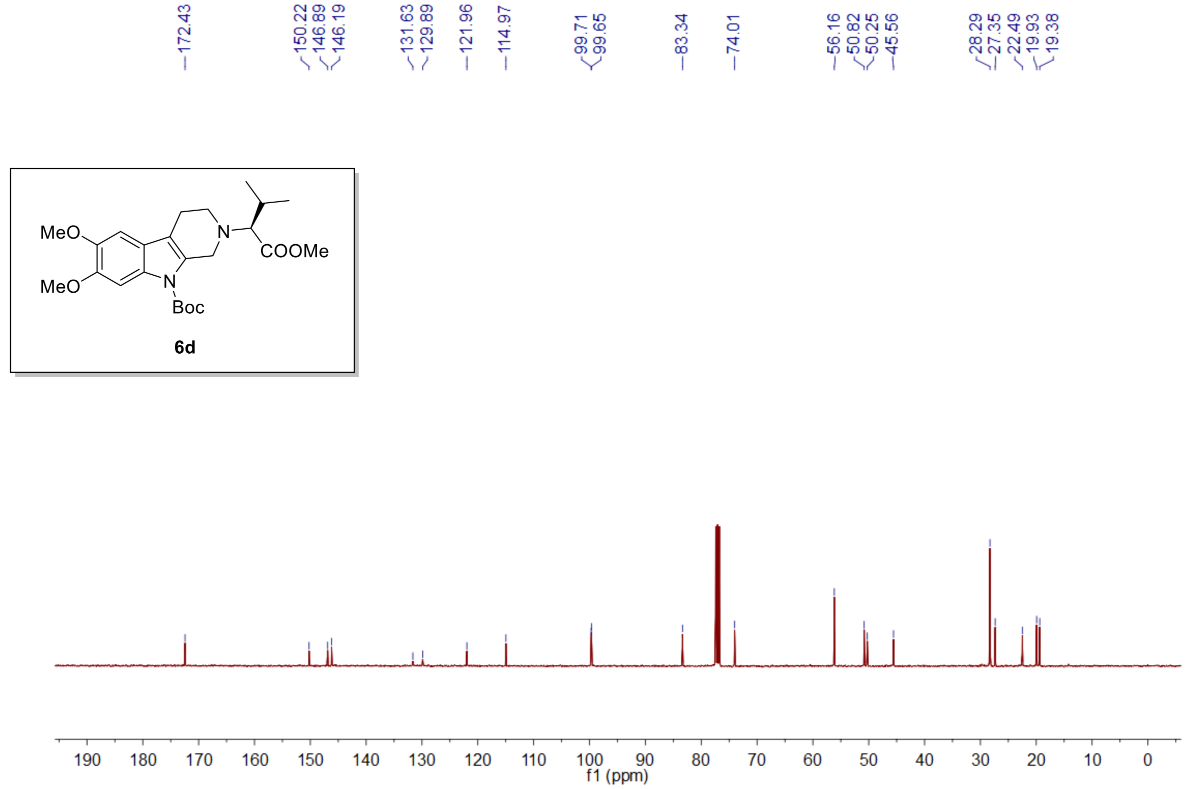

^{1}H-NMR spectrum of compound **6e** (400 MHz, $CDCl_3$)

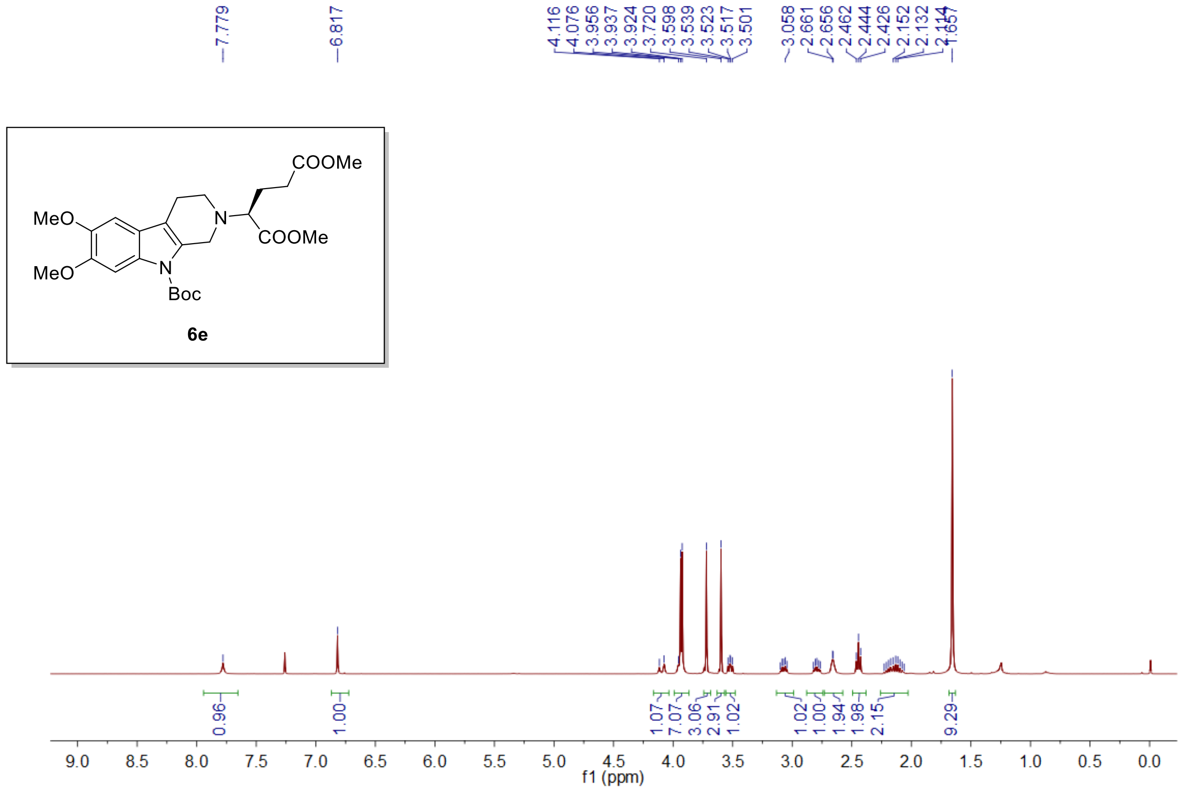

^{13}C-NMR spectrum of compound **6e** (101 MHz, $CDCl_3$)

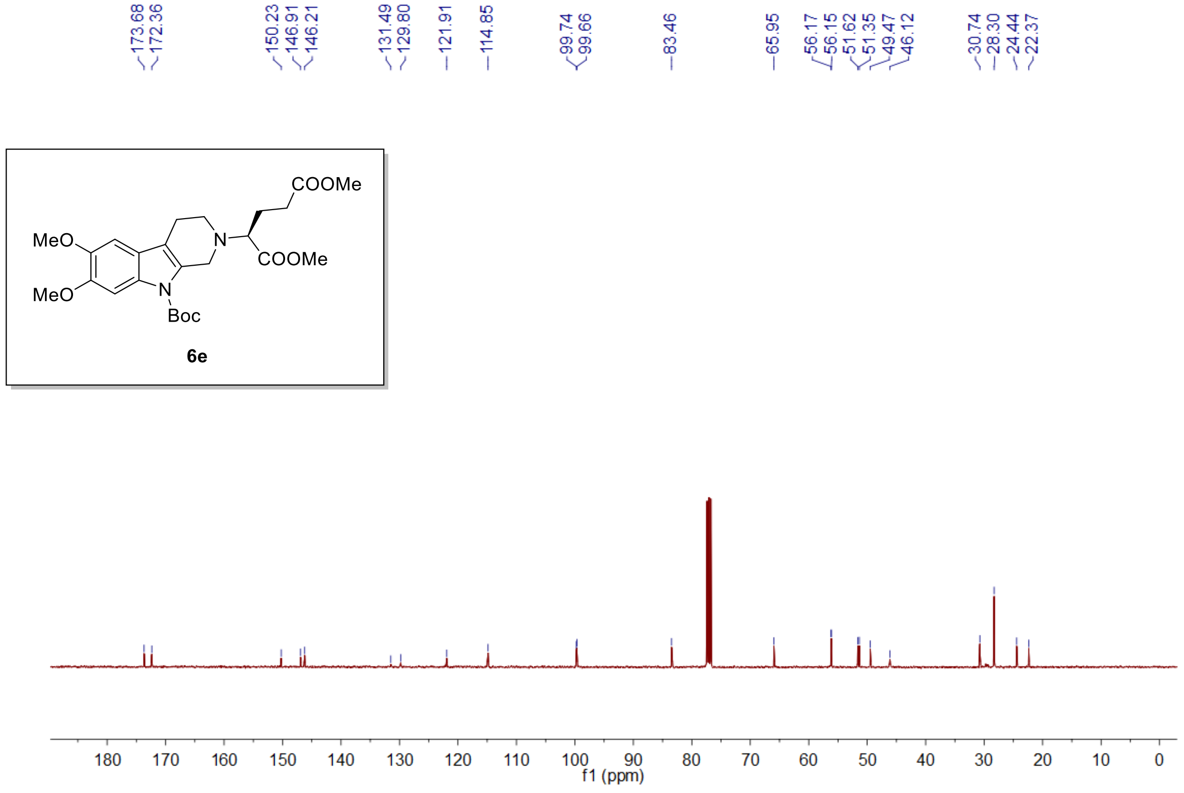

^{1}H-NMR spectrum of compound **6f** (400 MHz, $CDCl_3$)

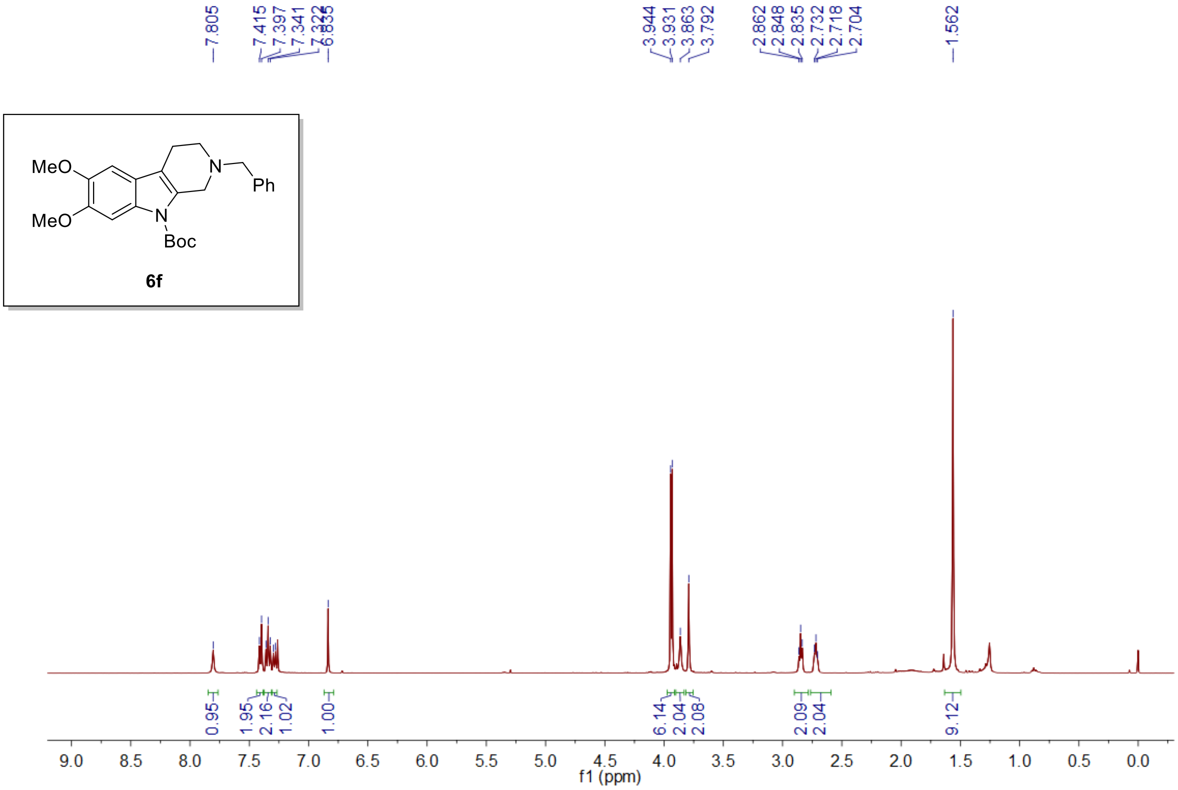

^{13}C-NMR spectrum of compound **6f** (101 MHz, $CDCl_3$)

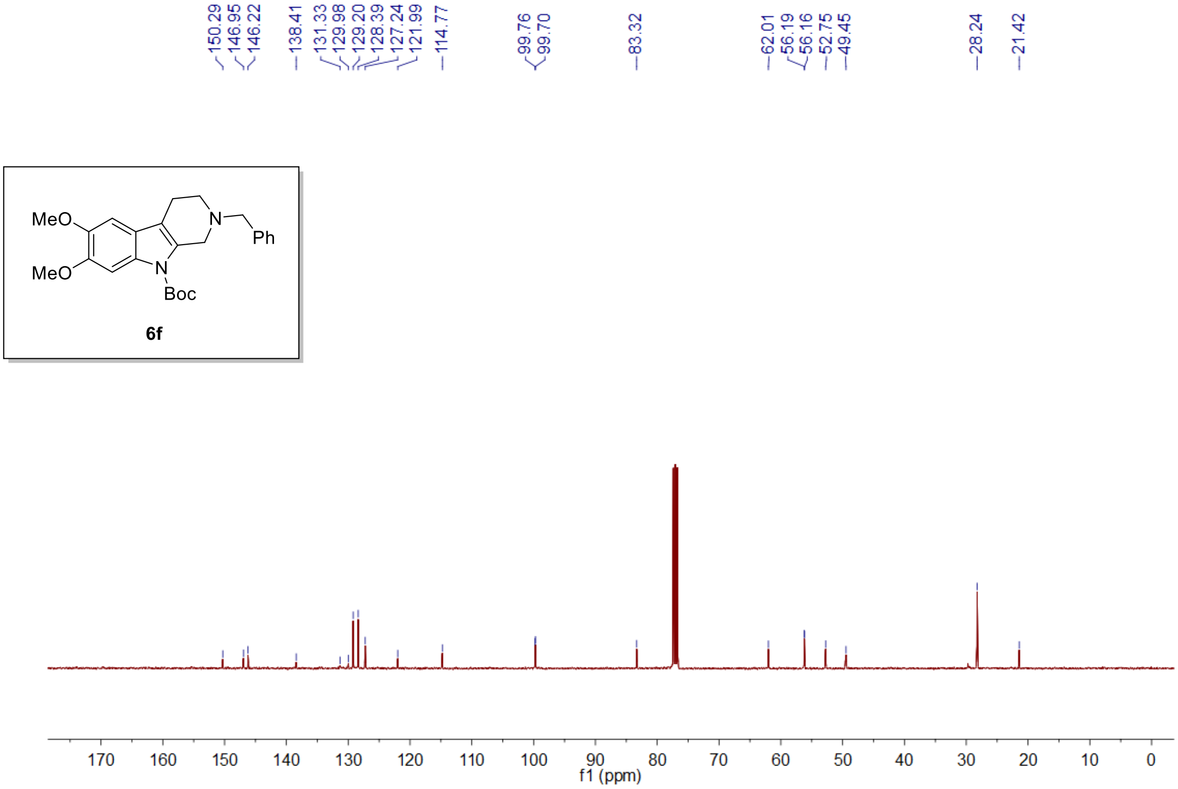

^{1}H-NMR spectrum of compound **6g** (400 MHz, $CDCl_3$)

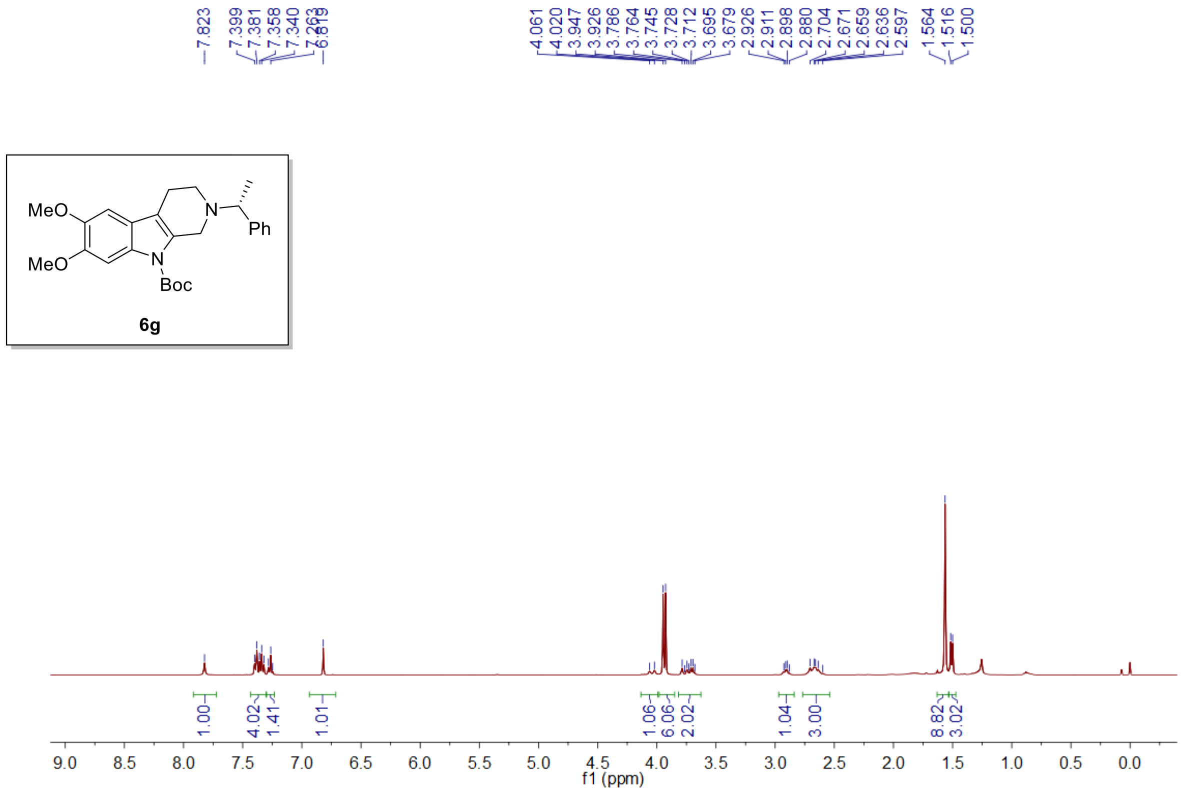

^{13}C-NMR spectrum of compound **6g** (101 MHz, $CDCl_3$)

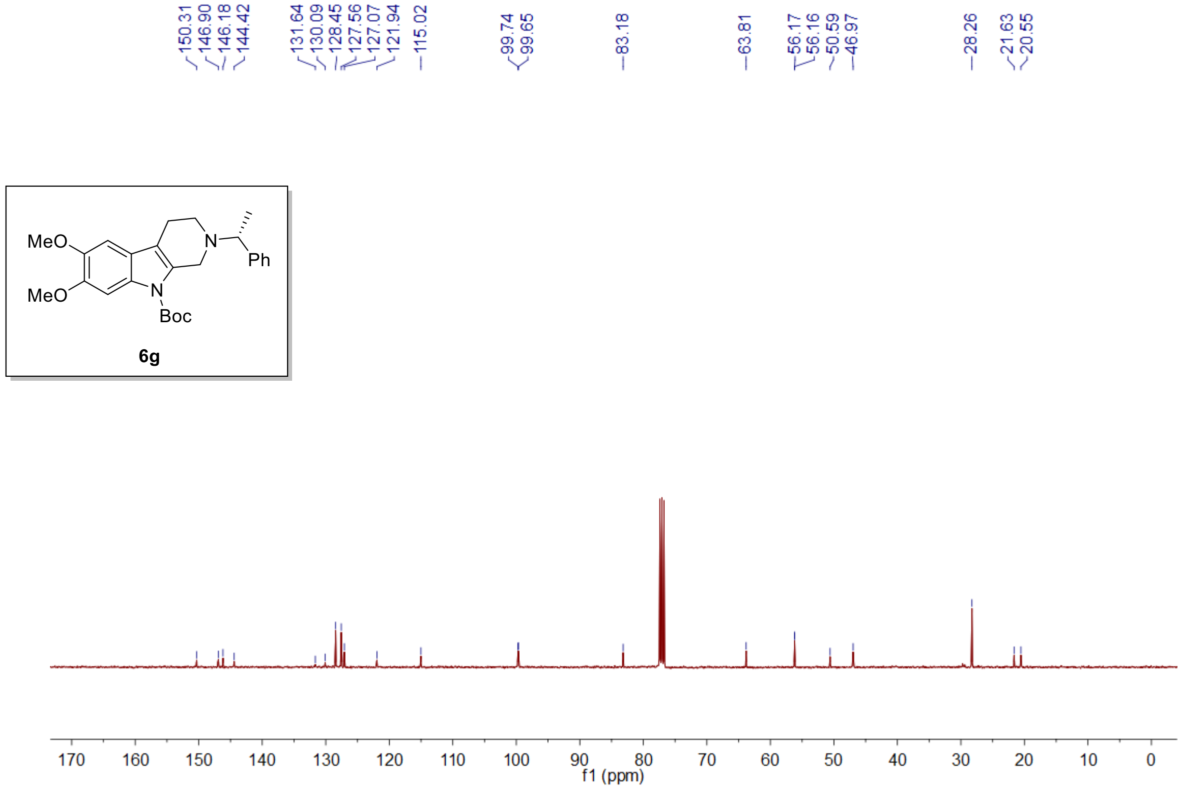

^{1}H-NMR spectrum of compound **6h** (400 MHz, $CDCl_3$)

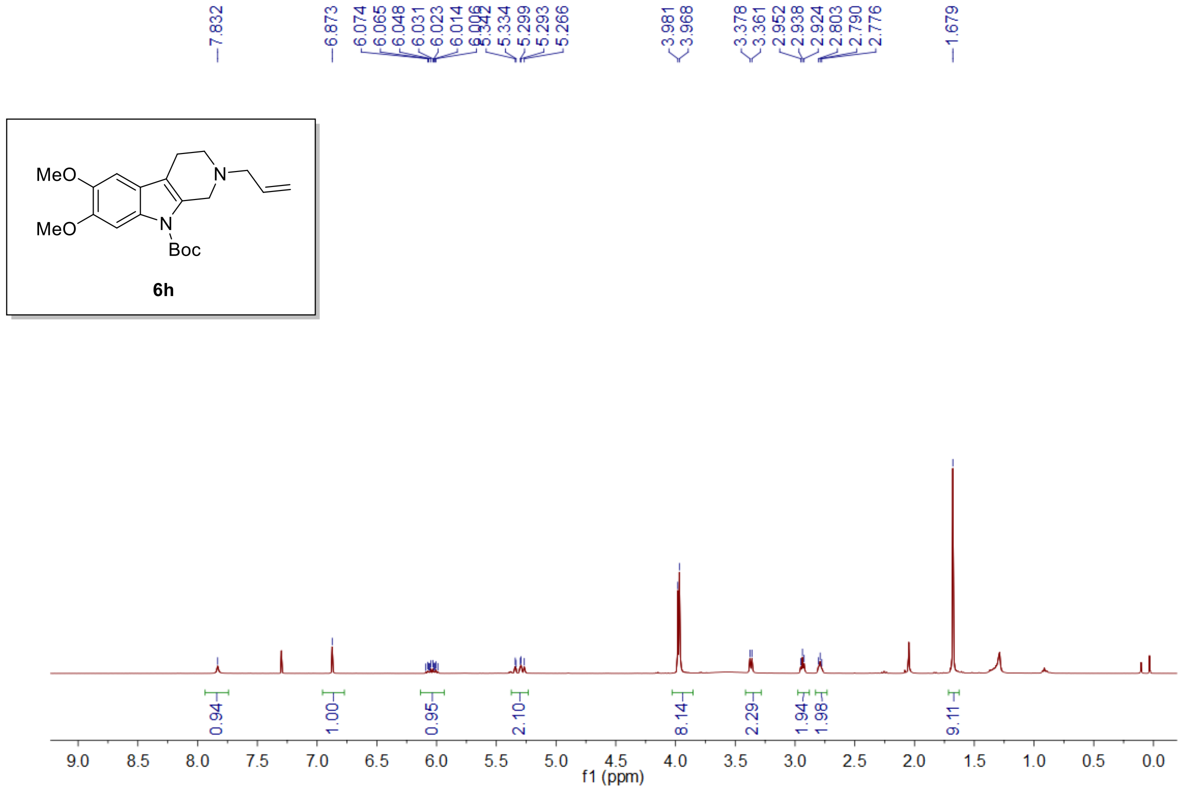

^{13}C-NMR spectrum of compound **6h** (101 MHz, $CDCl_3$)

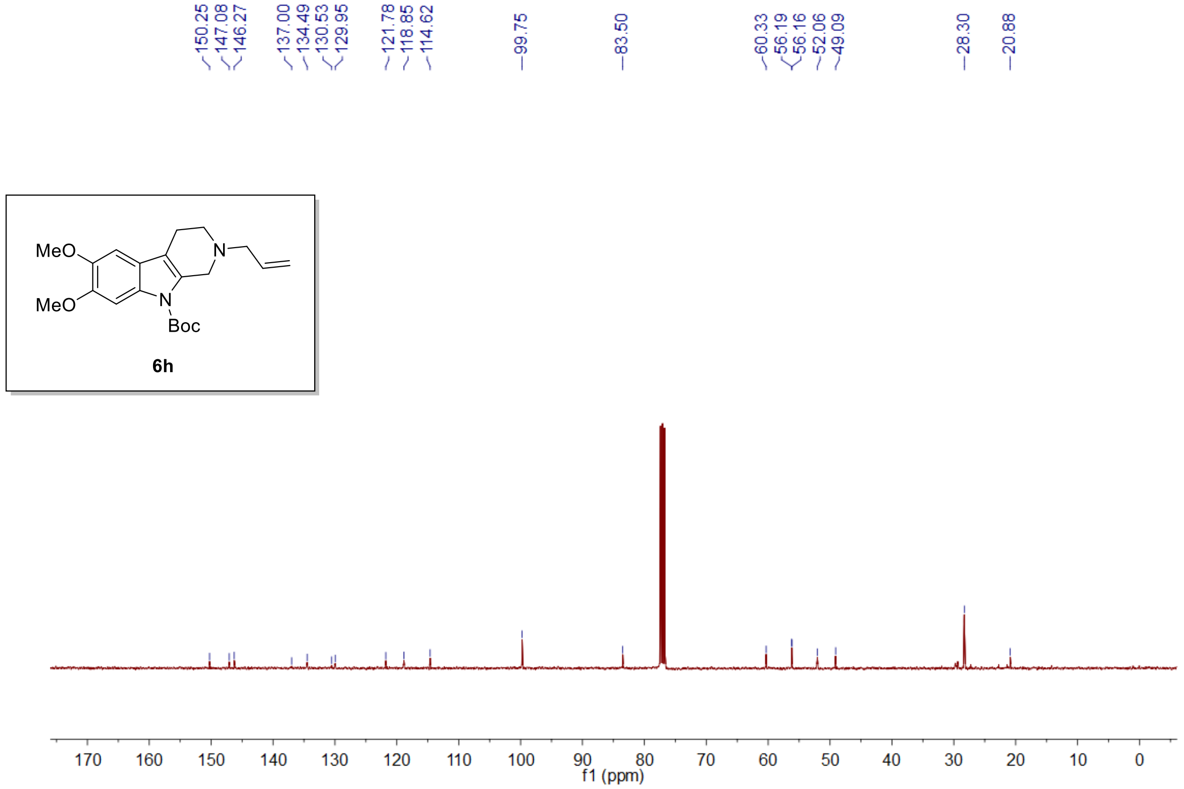

^{1}H-NMR spectrum of compound **6i** (400 MHz, $CDCl_3$)

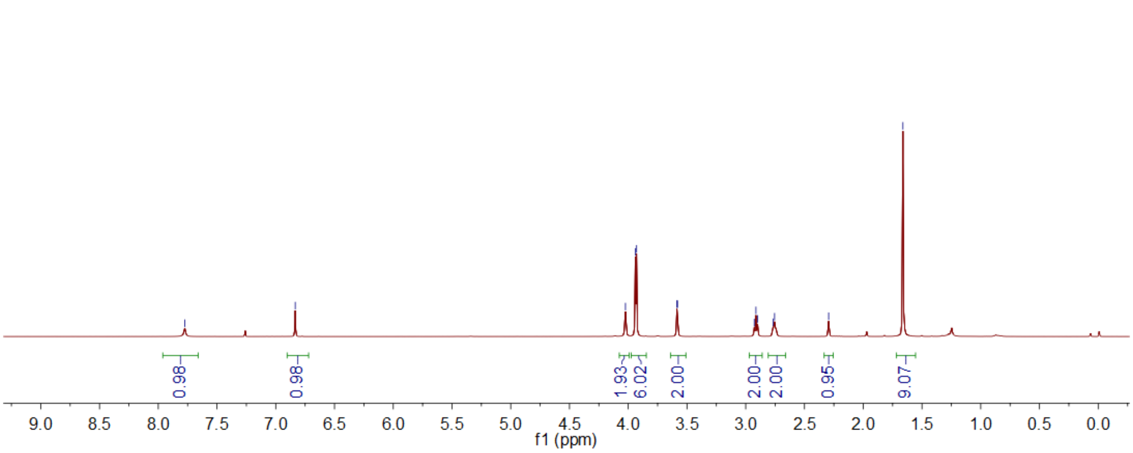

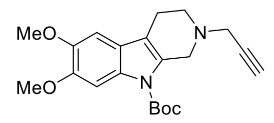

6i

^{13}C-NMR spectrum of compound **6i** (101 MHz, $CDCl_3$)

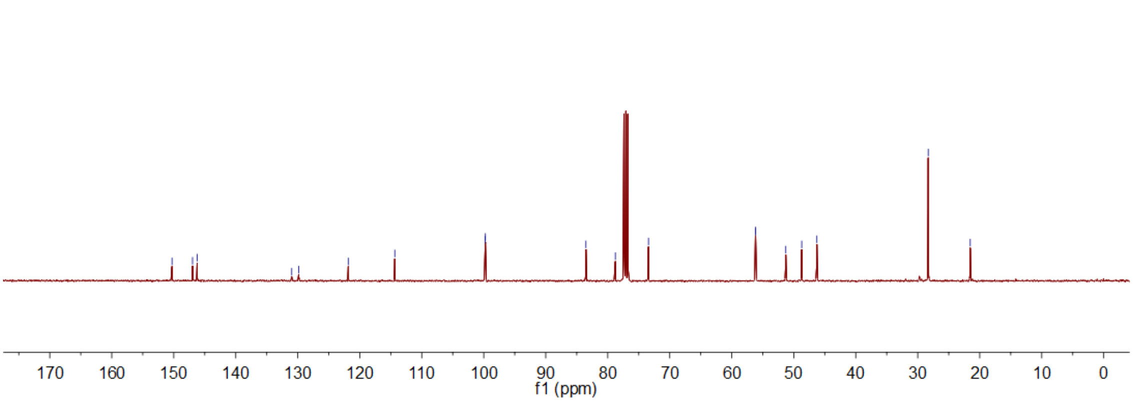

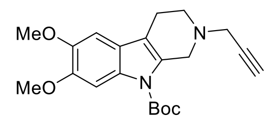

6i

^{1}H-NMR spectrum of compound **6j** (400 MHz, $CDCl_3$)

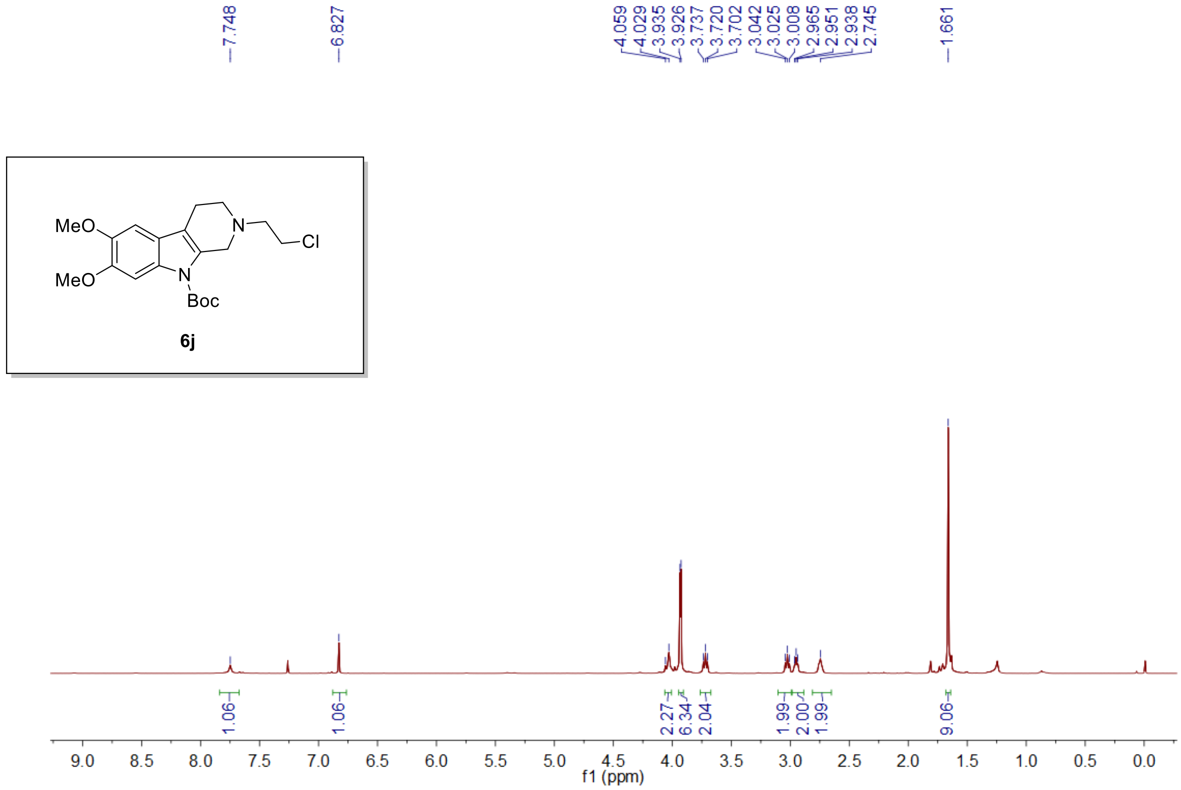

^{13}C-NMR spectrum of compound **6j** (101 MHz, $CDCl_3$)

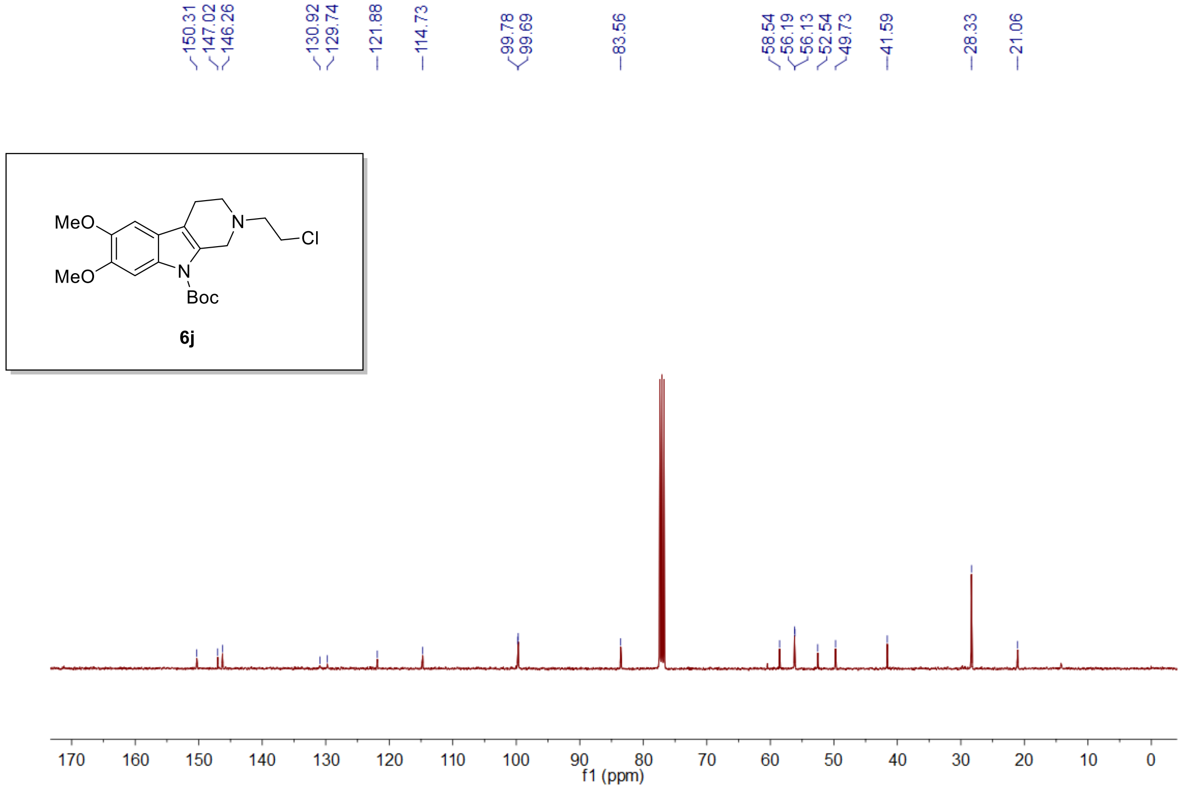

^{1}H-NMR spectrum of compound **6k** (400 MHz, $CDCl_3$)

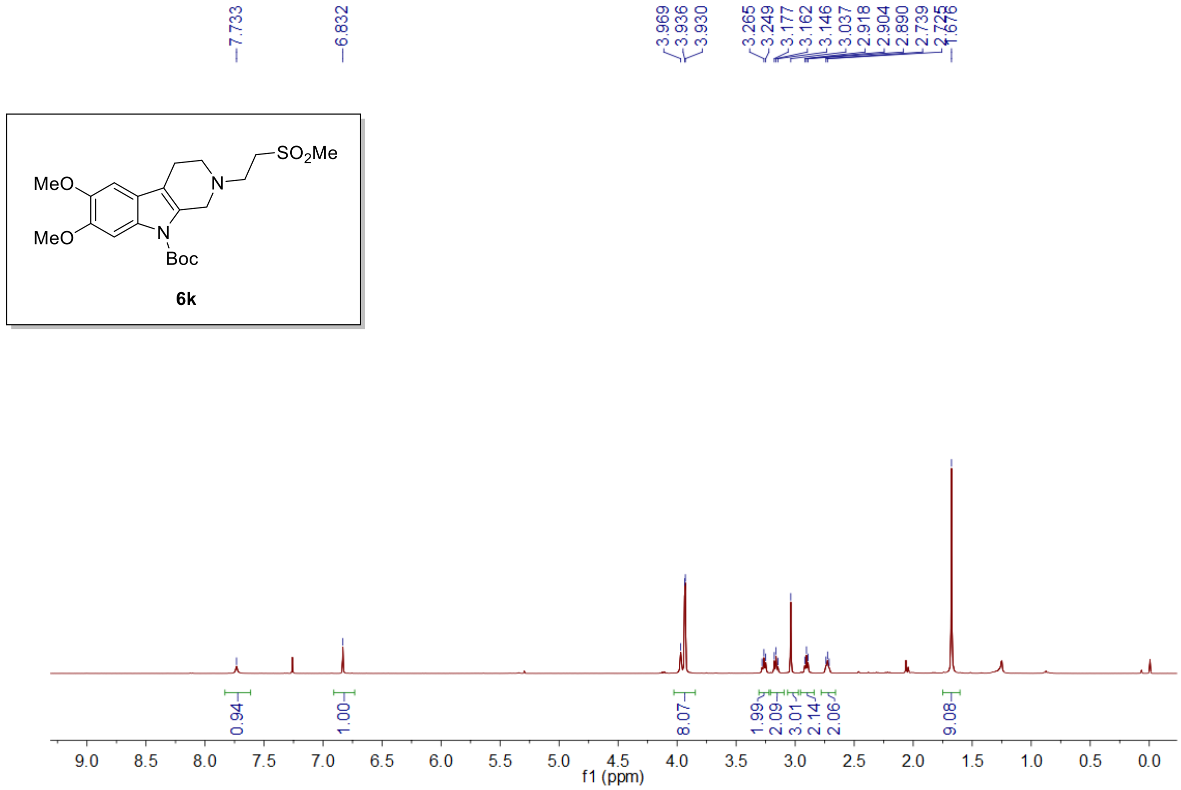

^{13}C-NMR spectrum of compound **6k** (101 MHz, $CDCl_3$)

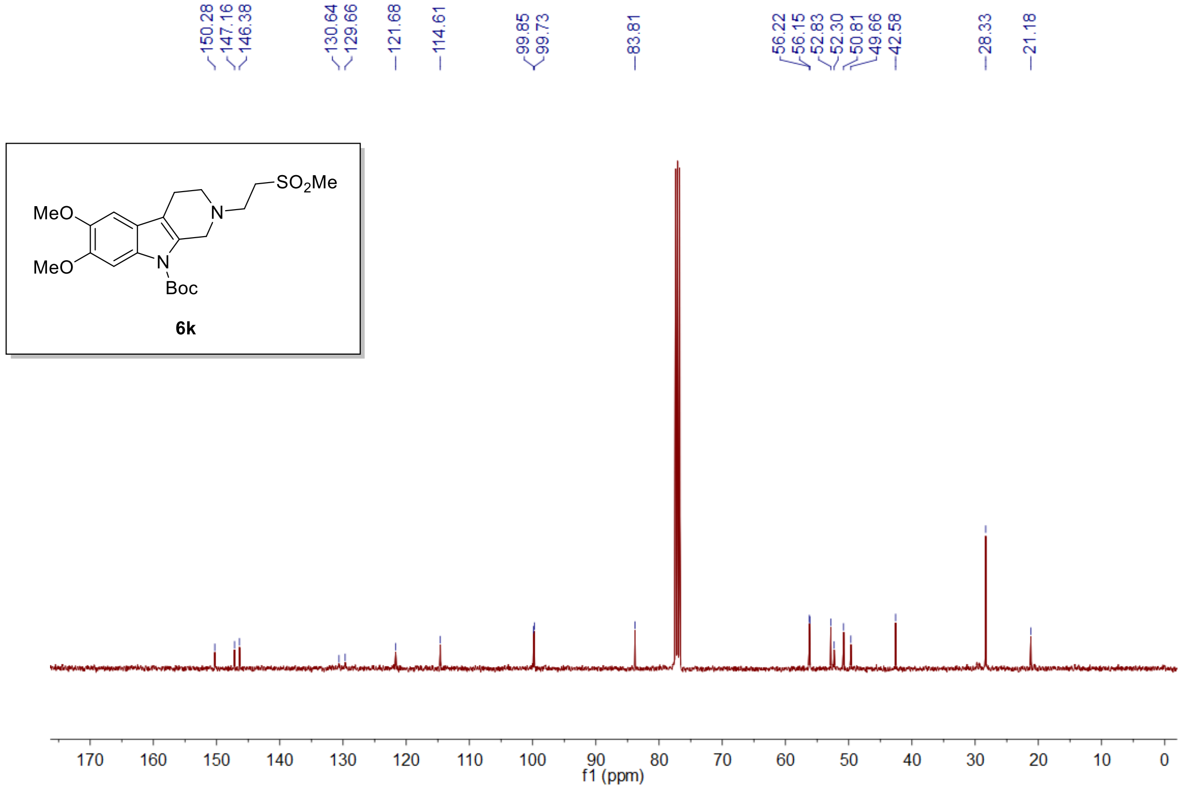

^{1}H-NMR spectrum of compound **6l** (400 MHz, $CDCl_3$)

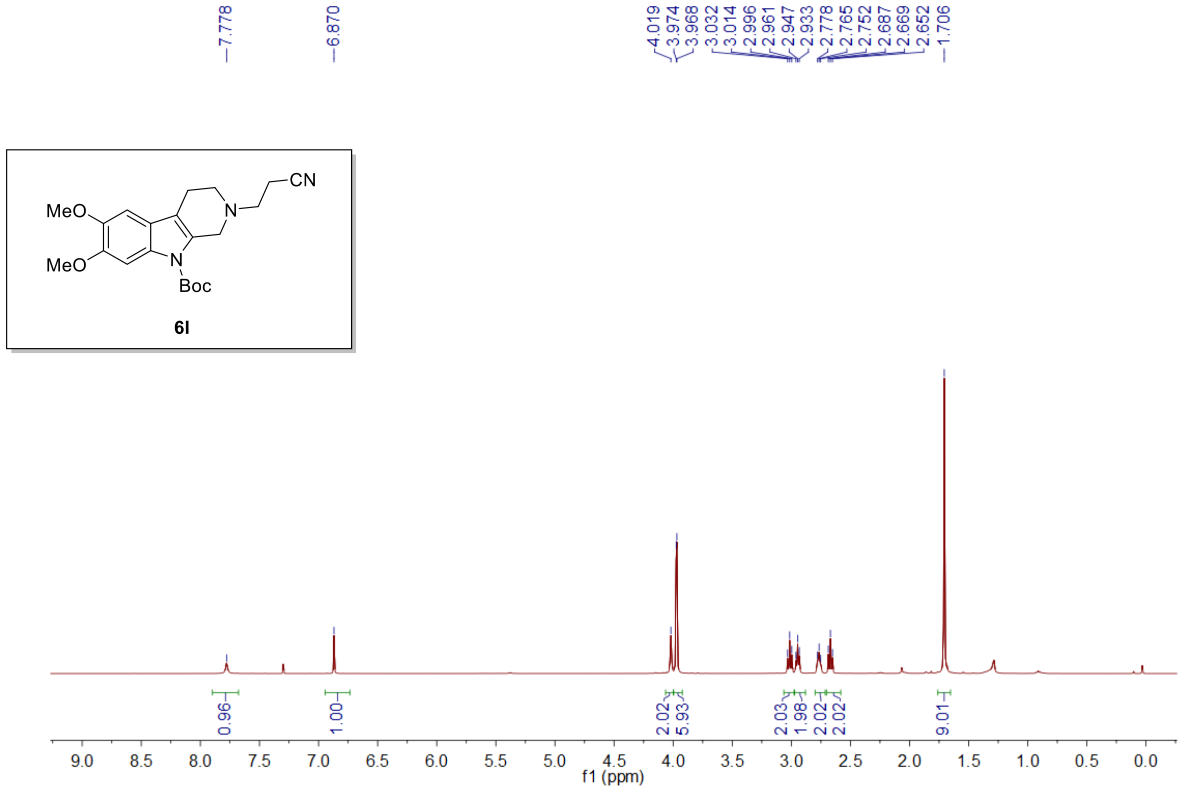

^{13}C-NMR spectrum of compound **6l** (101 MHz, $CDCl_3$)

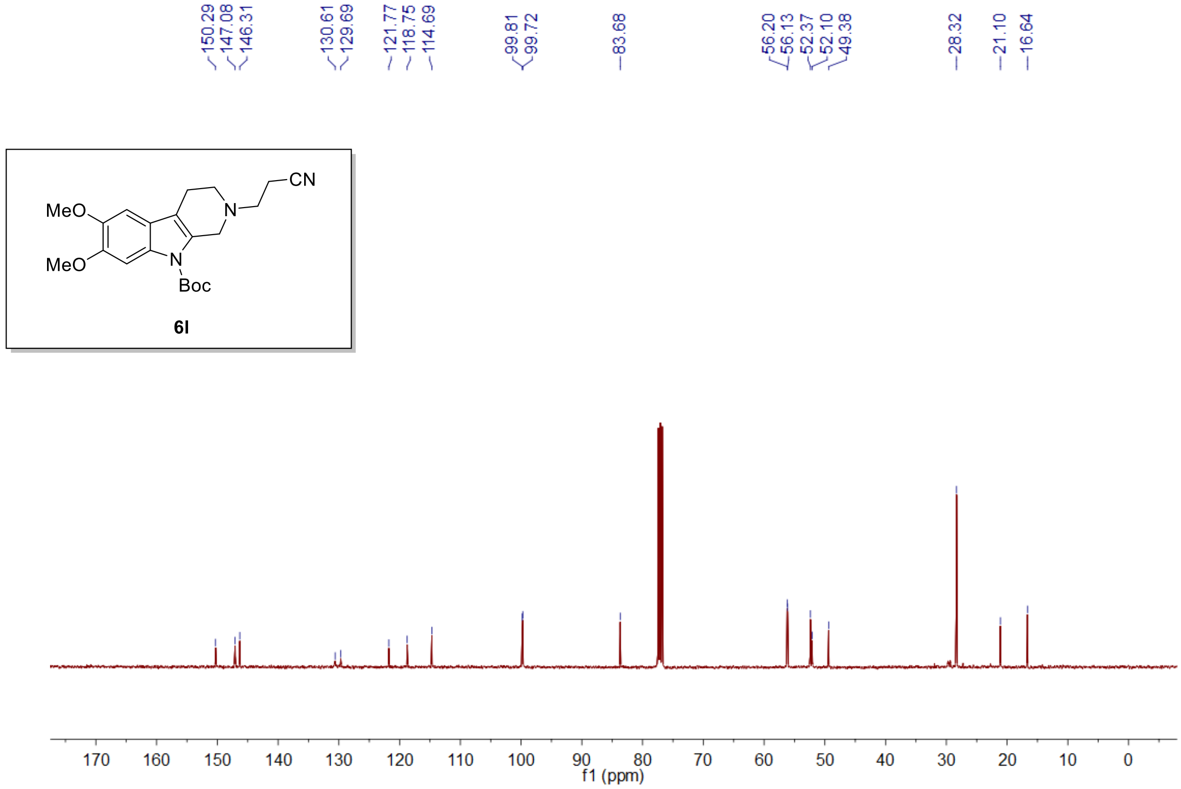

^{1}H-NMR spectrum of compound **6m** (400 MHz, $CDCl_3$)

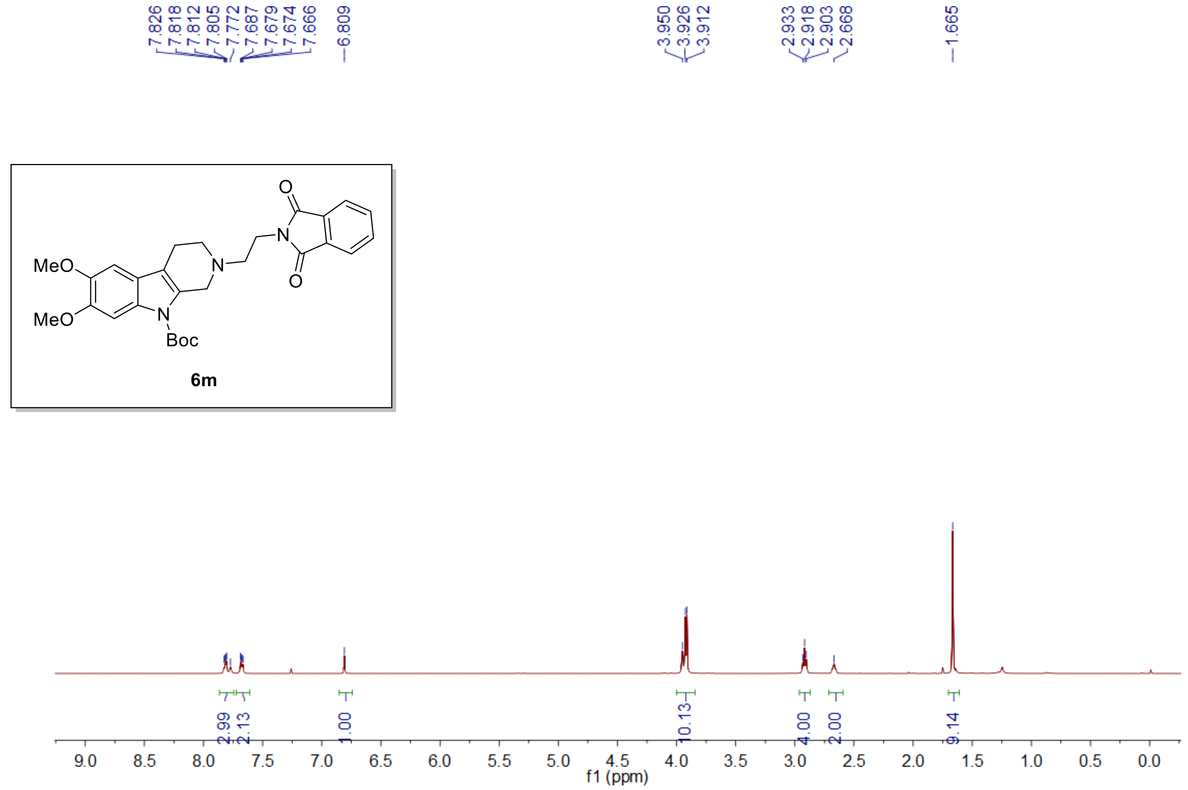

^{13}C-NMR spectrum of compound **6m** (101 MHz, $CDCl_3$)

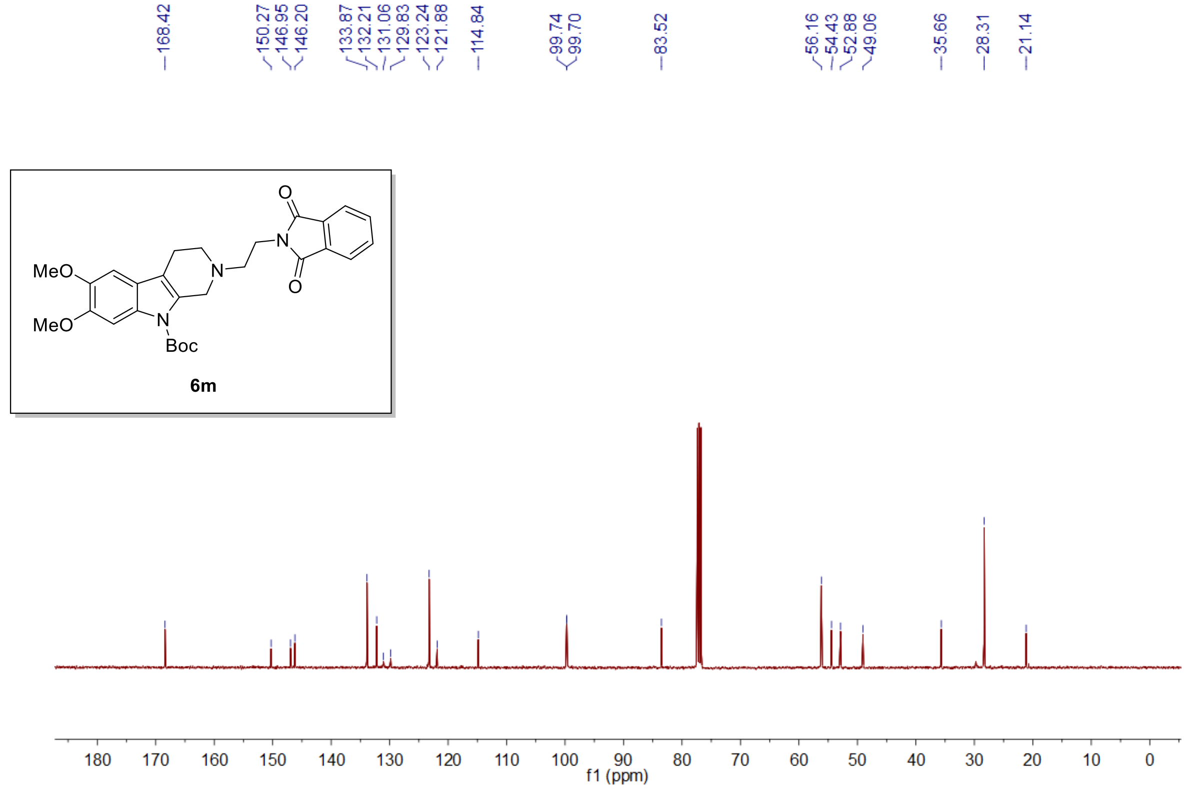

^{1}H-NMR spectrum of compound **6n** (400 MHz, $CDCl_3$)

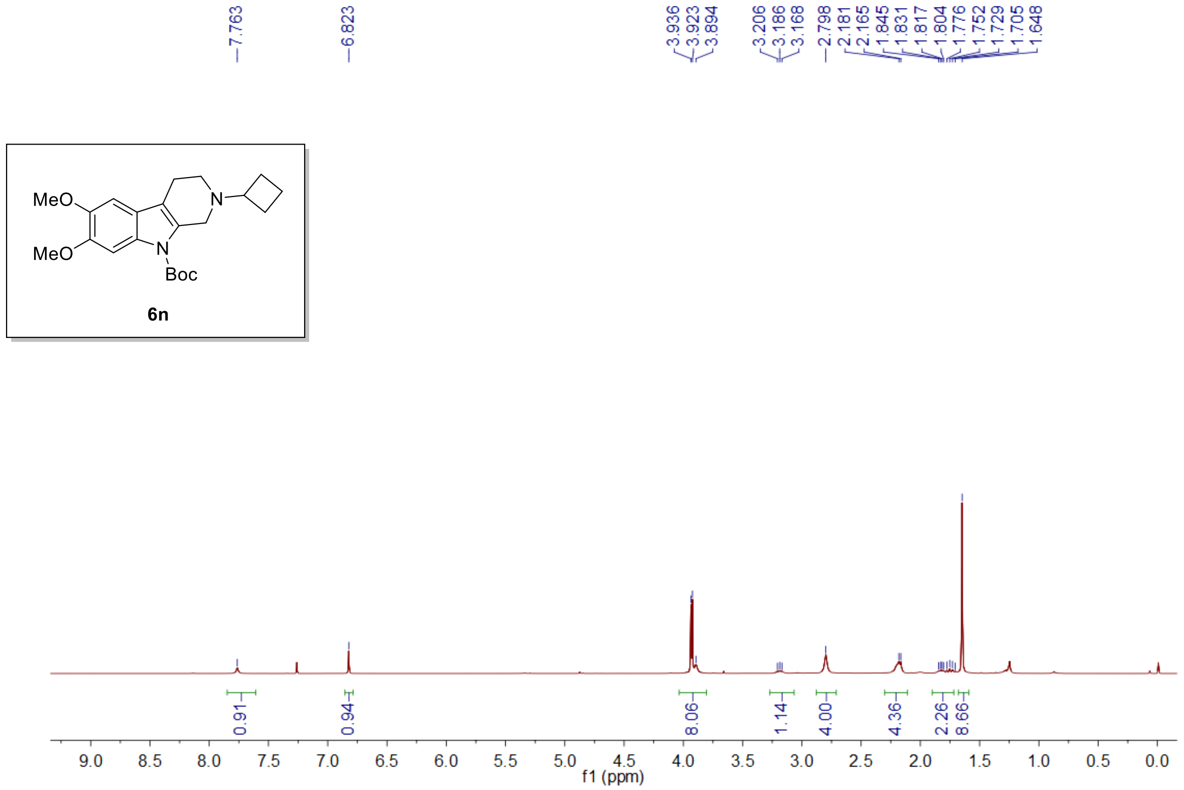

^{13}C-NMR spectrum of compound **6n** (101 MHz, $CDCl_3$)

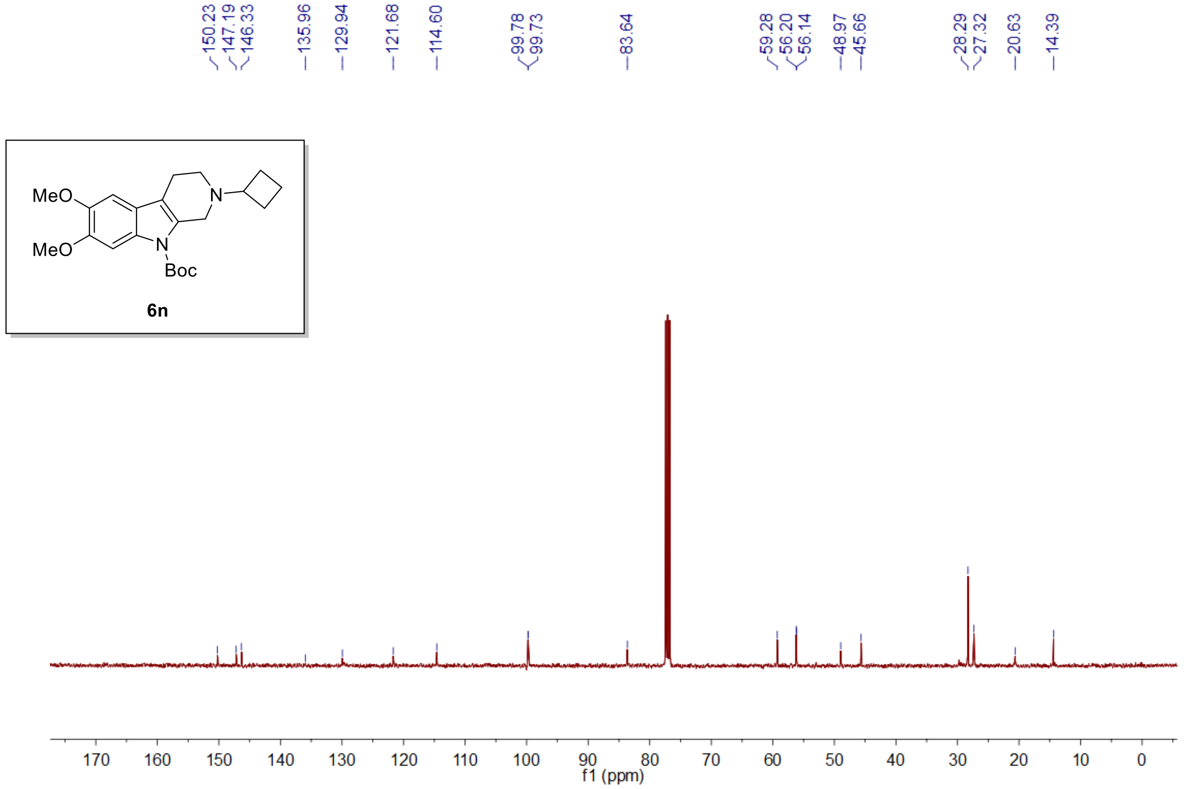

^{1}H-NMR spectrum of compound **6o** (400 MHz, $CDCl_3$)

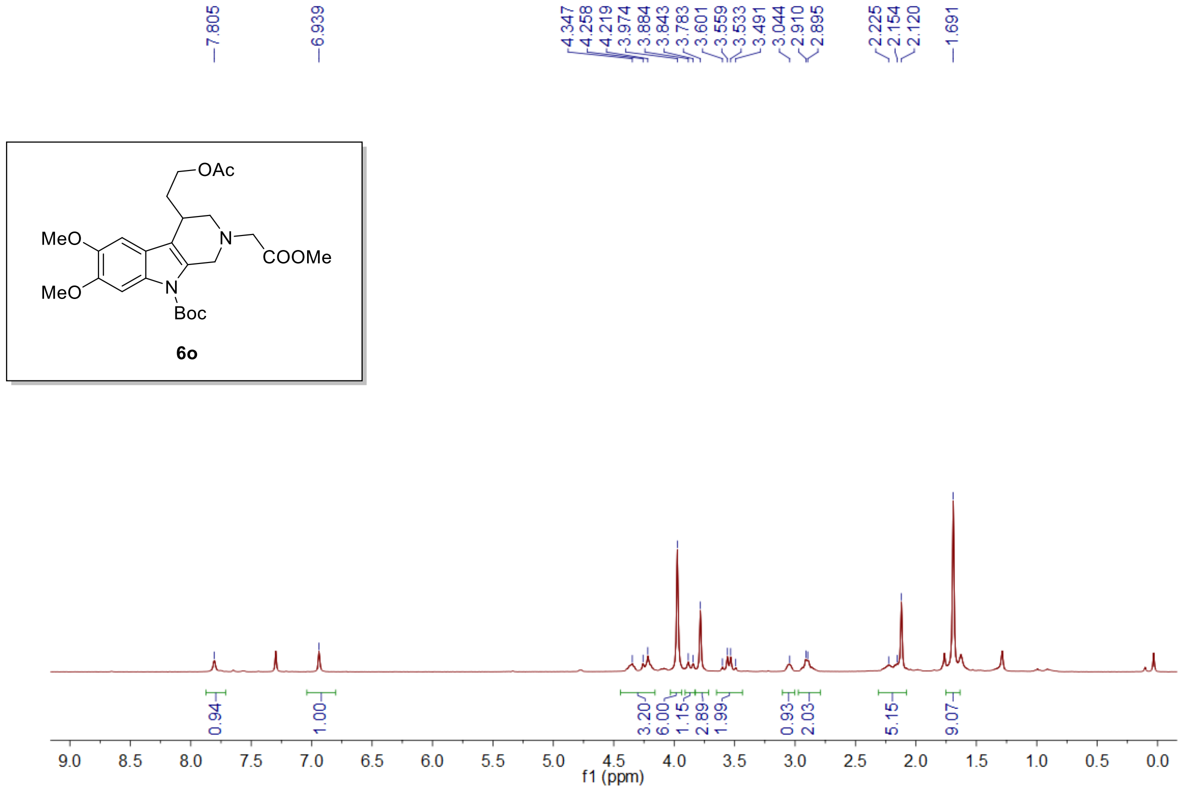

^{13}C-NMR spectrum of compound **6o** (101 MHz, $CDCl_3$)

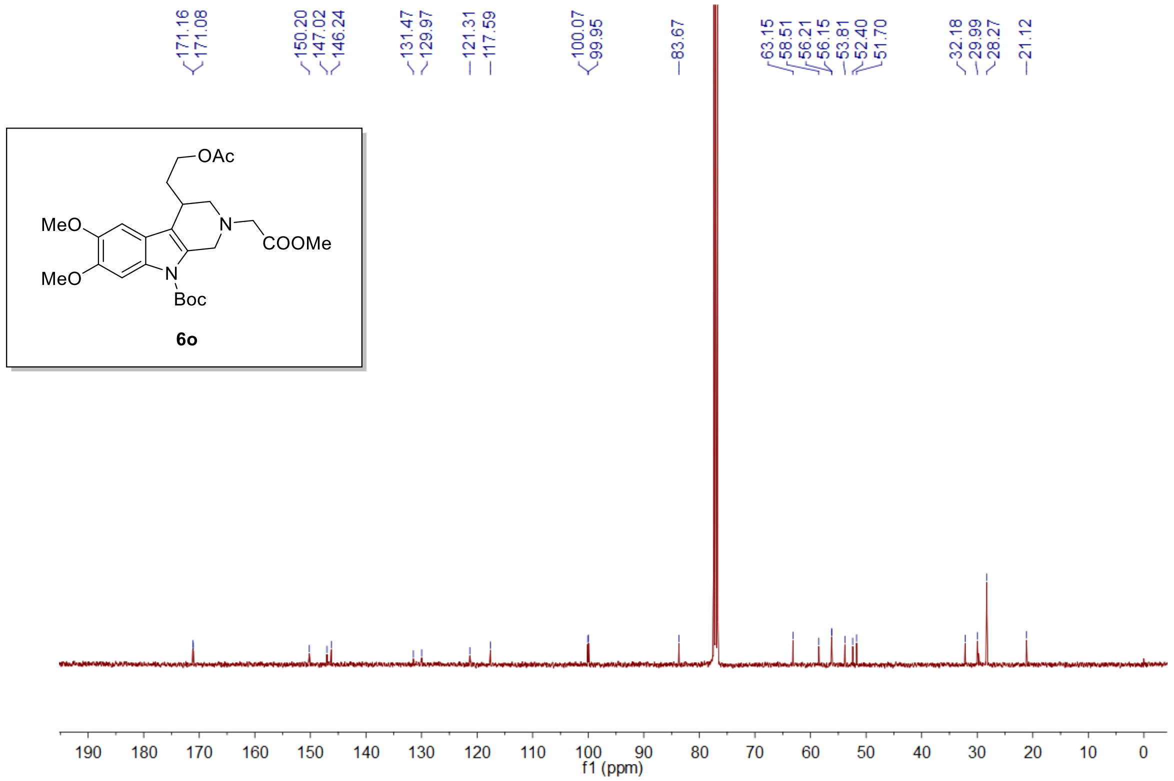

^{1}H-NMR spectrum of compound **6p** (600 MHz, $CDCl_3$)

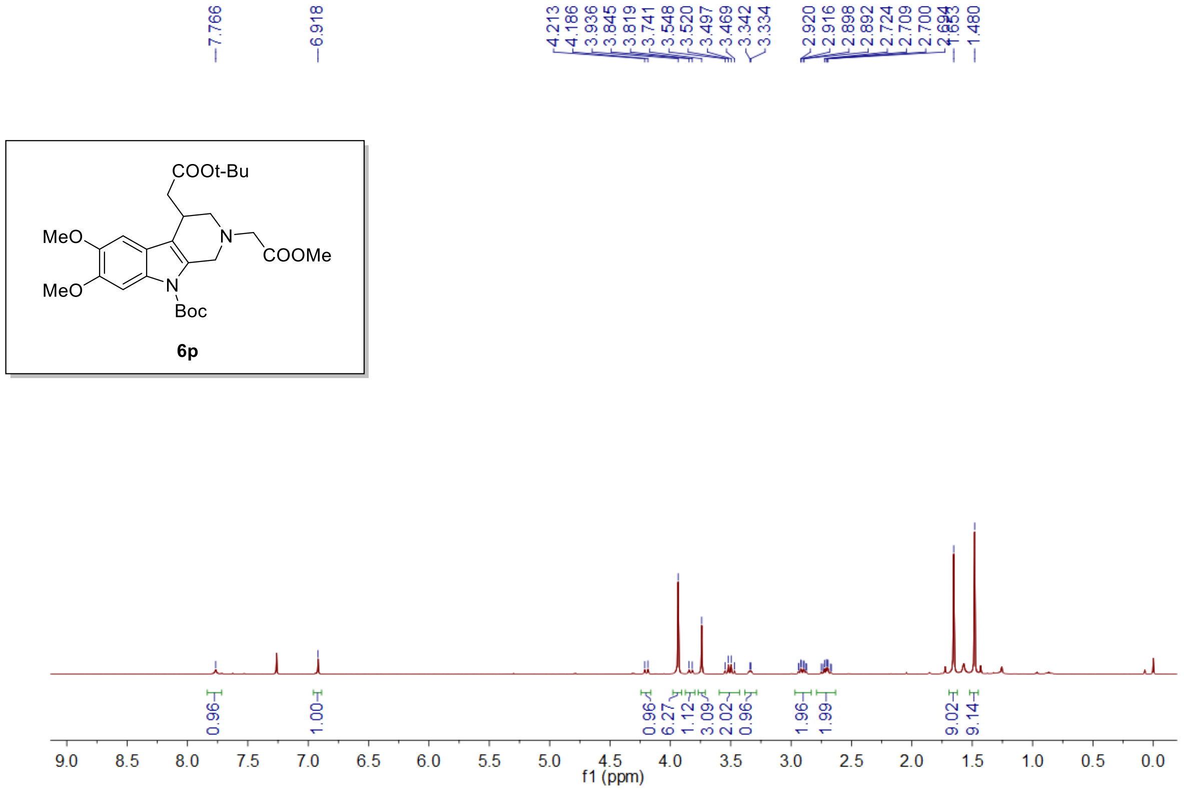

^{13}C-NMR spectrum of compound **6p** (151 MHz, $CDCl_3$)

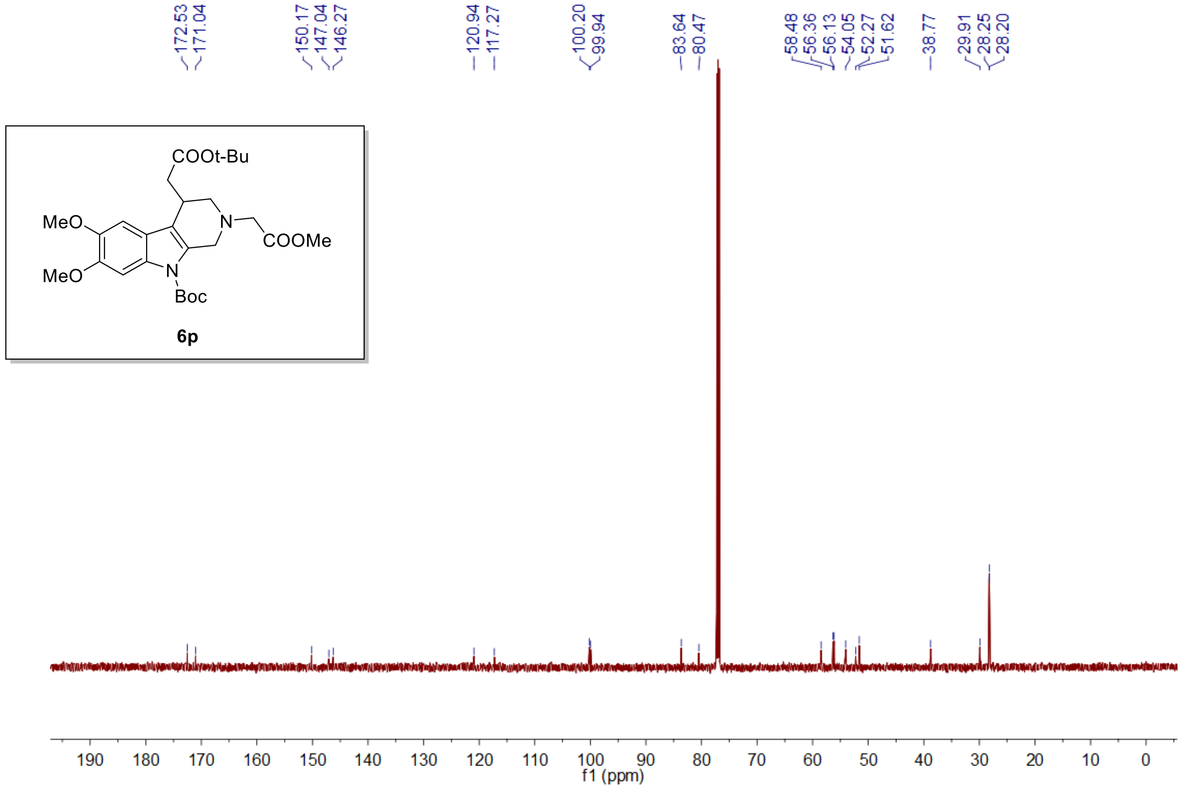

^{1}H-NMR spectrum of compound **6q** (400 MHz, $CDCl_3$)

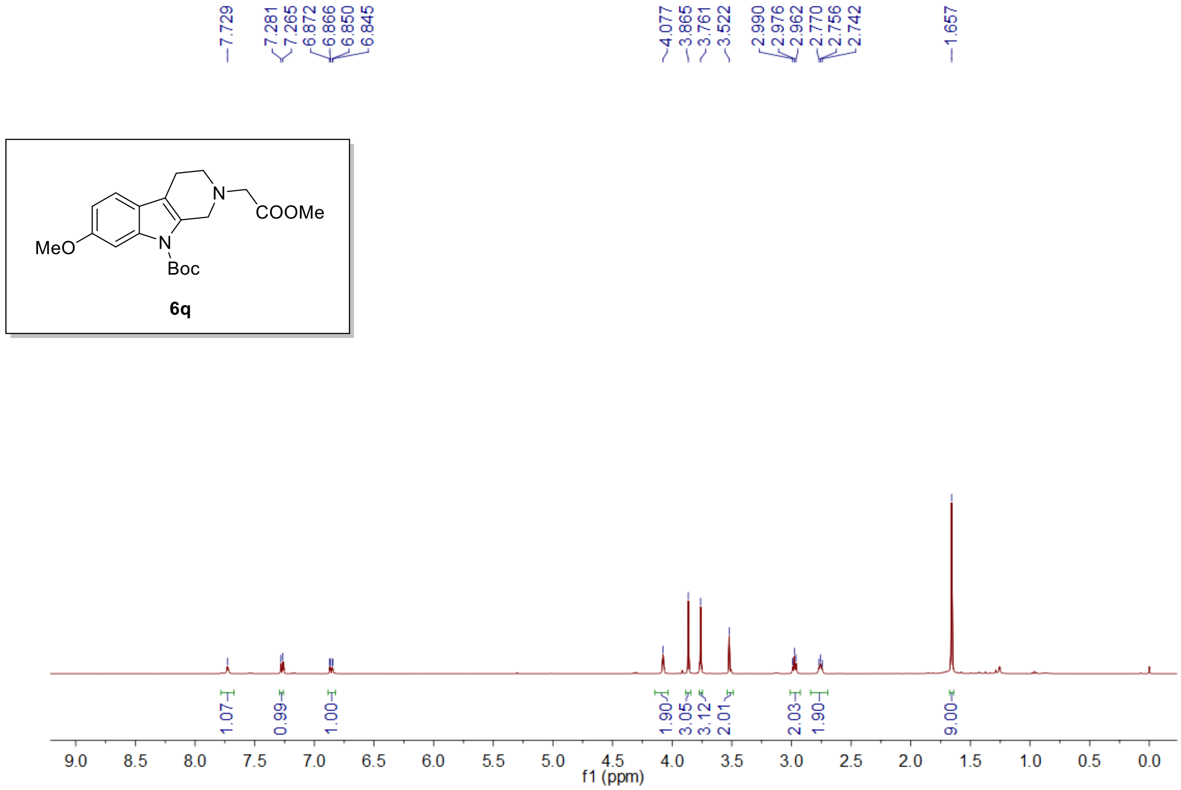

^{13}C-NMR spectrum of compound **6q** (101 MHz, $CDCl_3$)

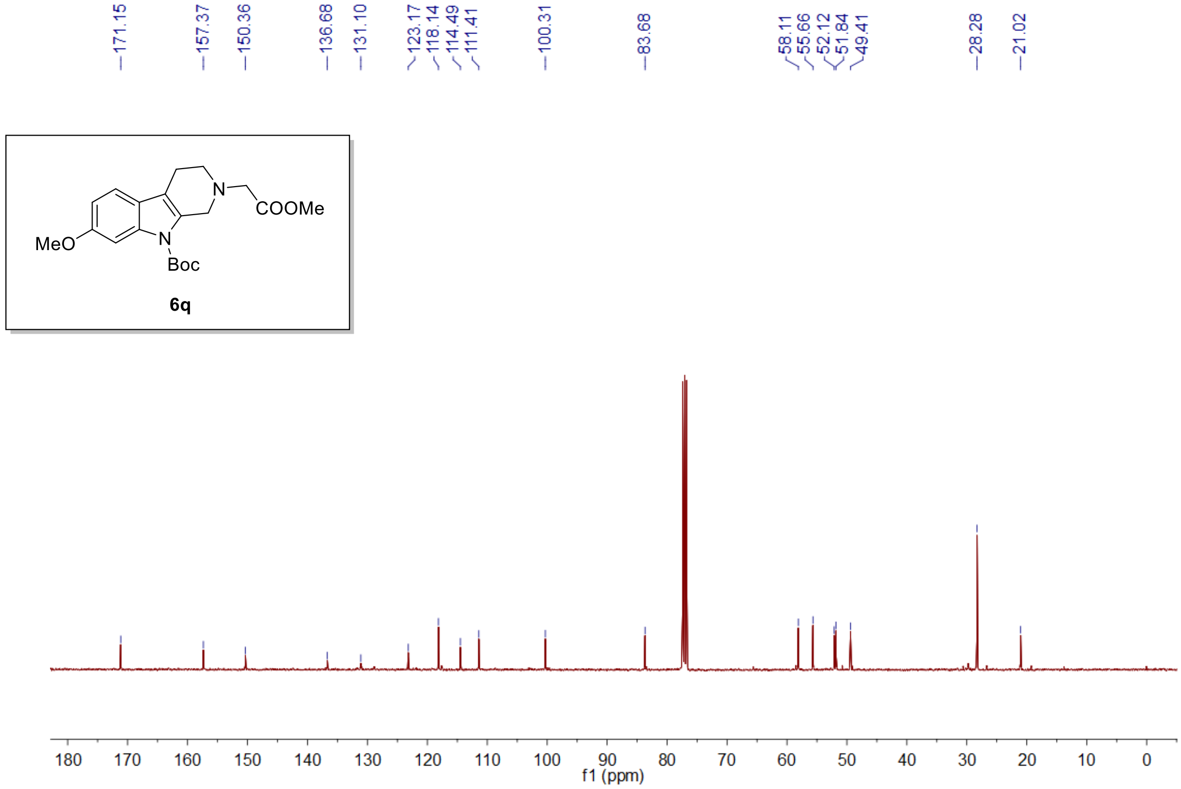

^{1}H-NMR spectrum of compound **6r** (400 MHz, $CDCl_3$)

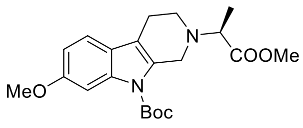

6r

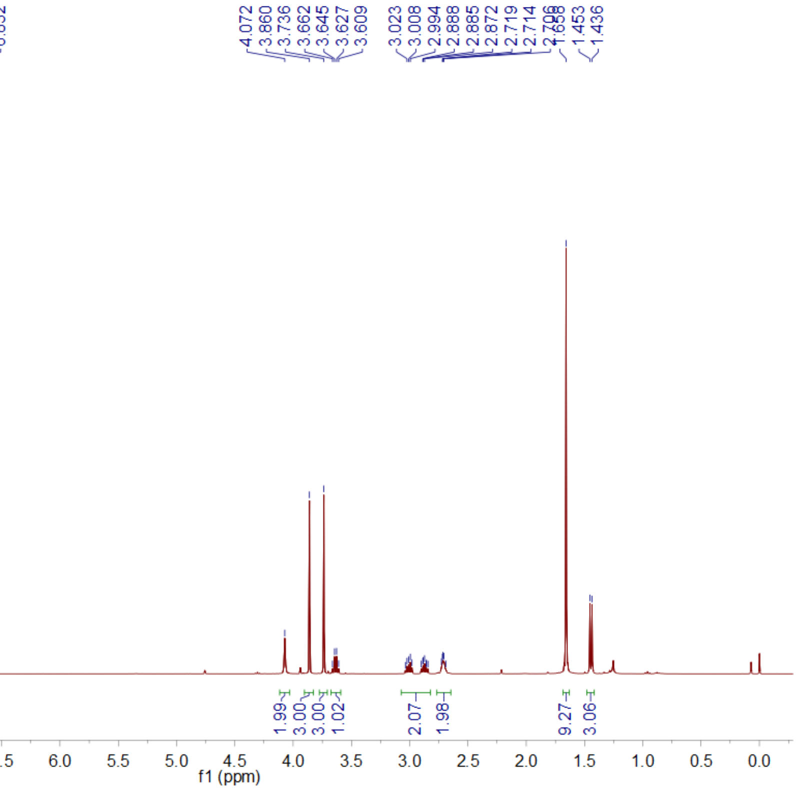

^{13}C-NMR spectrum of compound **6r** (101 MHz, $CDCl_3$)

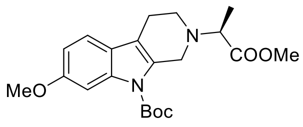

6r

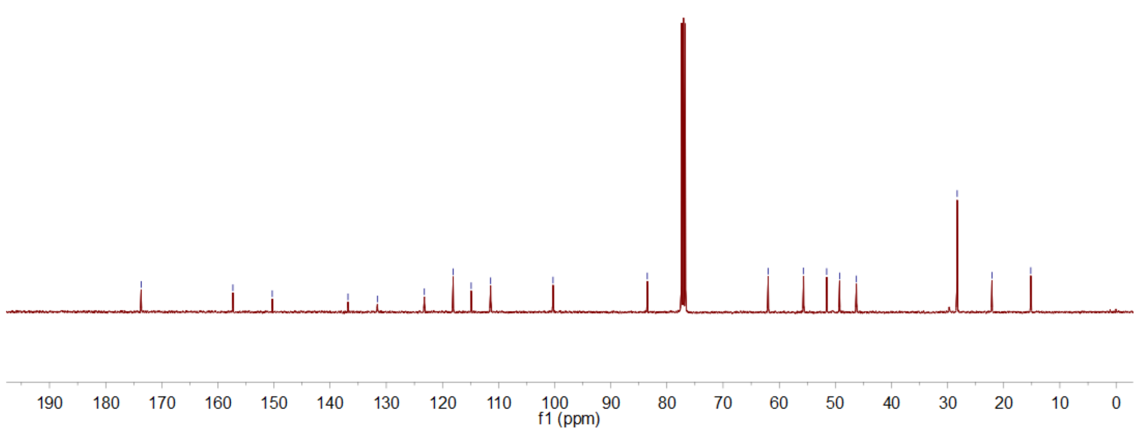

^{1}H-NMR spectrum of compound **6s** (400 MHz, $CDCl_3$)

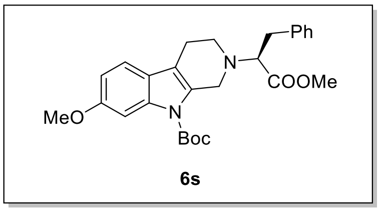

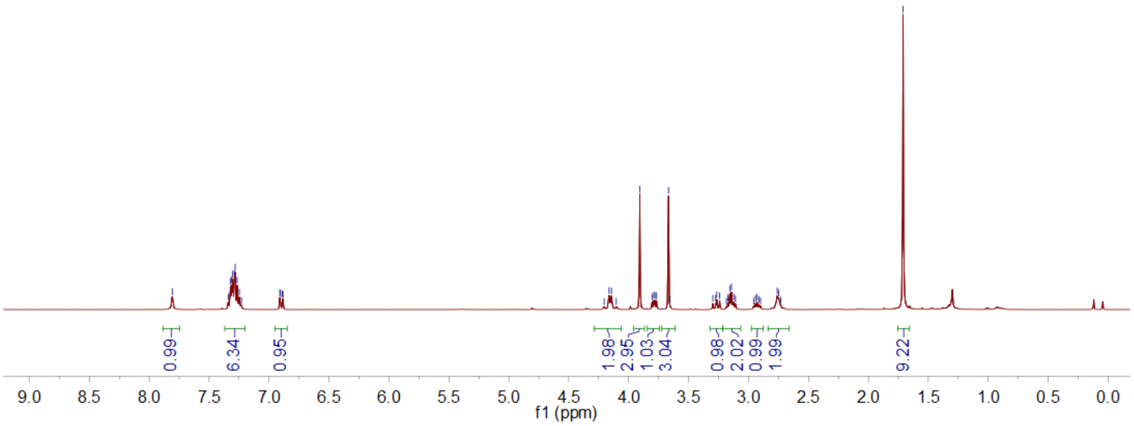

^{13}C-NMR spectrum of compound **6s** (101 MHz, $CDCl_3$)

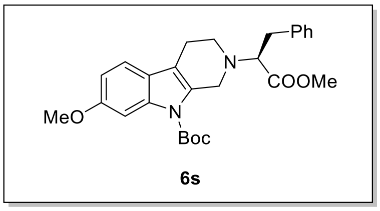

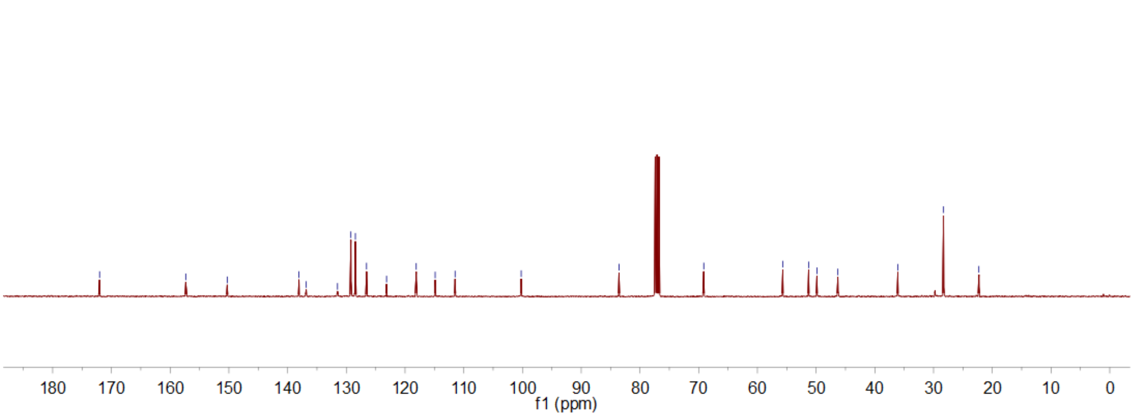

^{1}H-NMR spectrum of compound **6t** (400 MHz, $CDCl_3$)

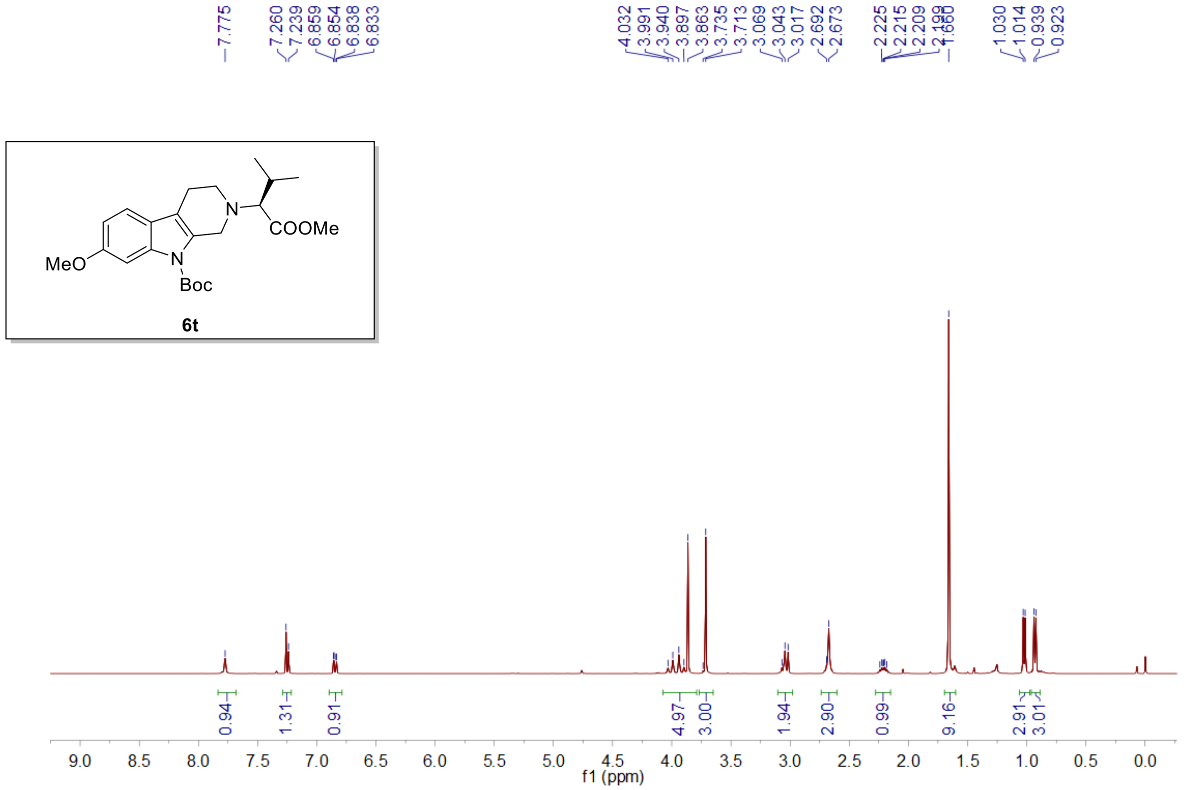

^{13}C-NMR spectrum of compound **6t** (101 MHz, $CDCl_3$)

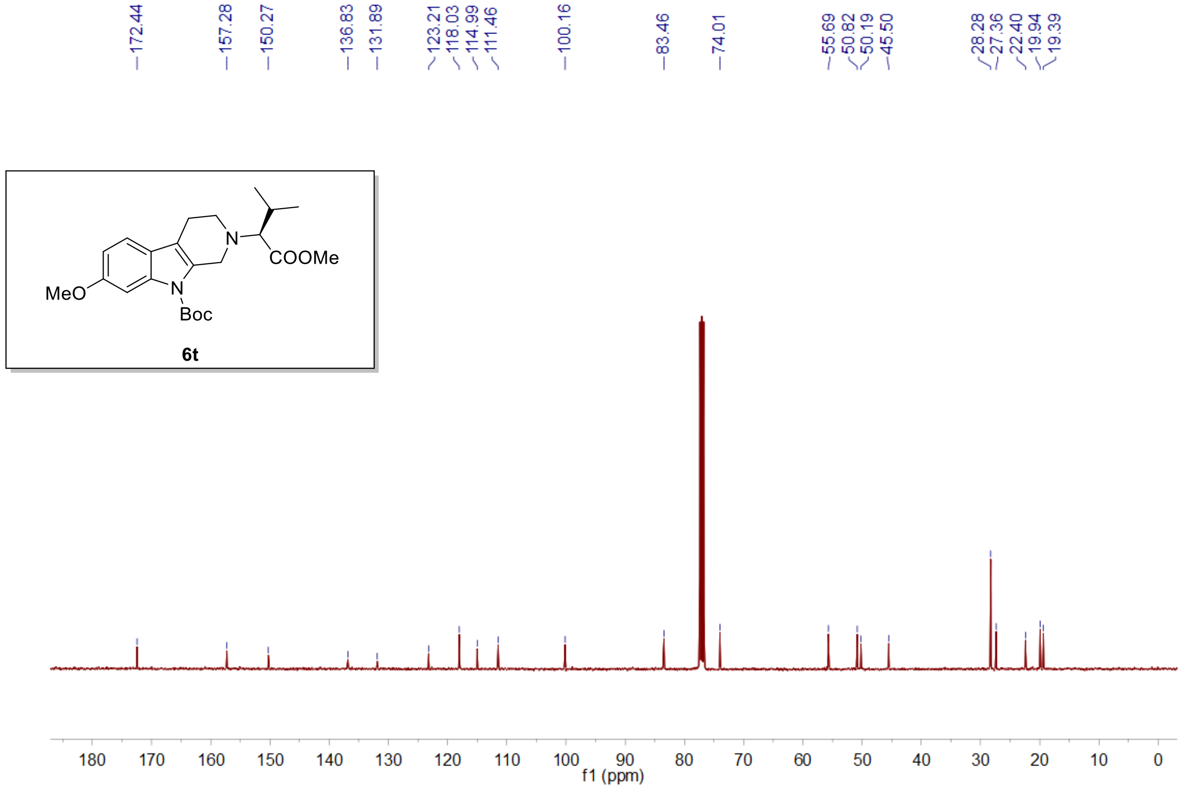

^{1}H-NMR spectrum of compound **6u** (400 MHz, $CDCl_3$)

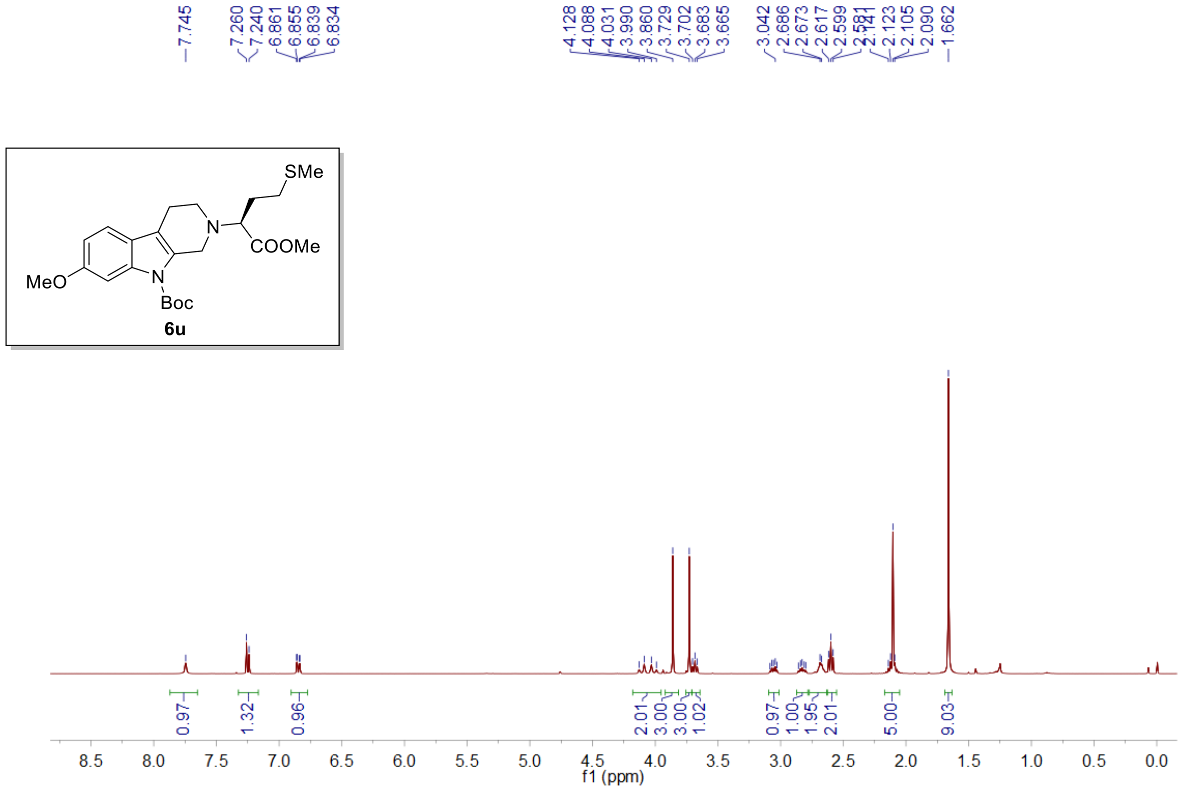

^{13}C-NMR spectrum of compound **6u** (101 MHz, $CDCl_3$)

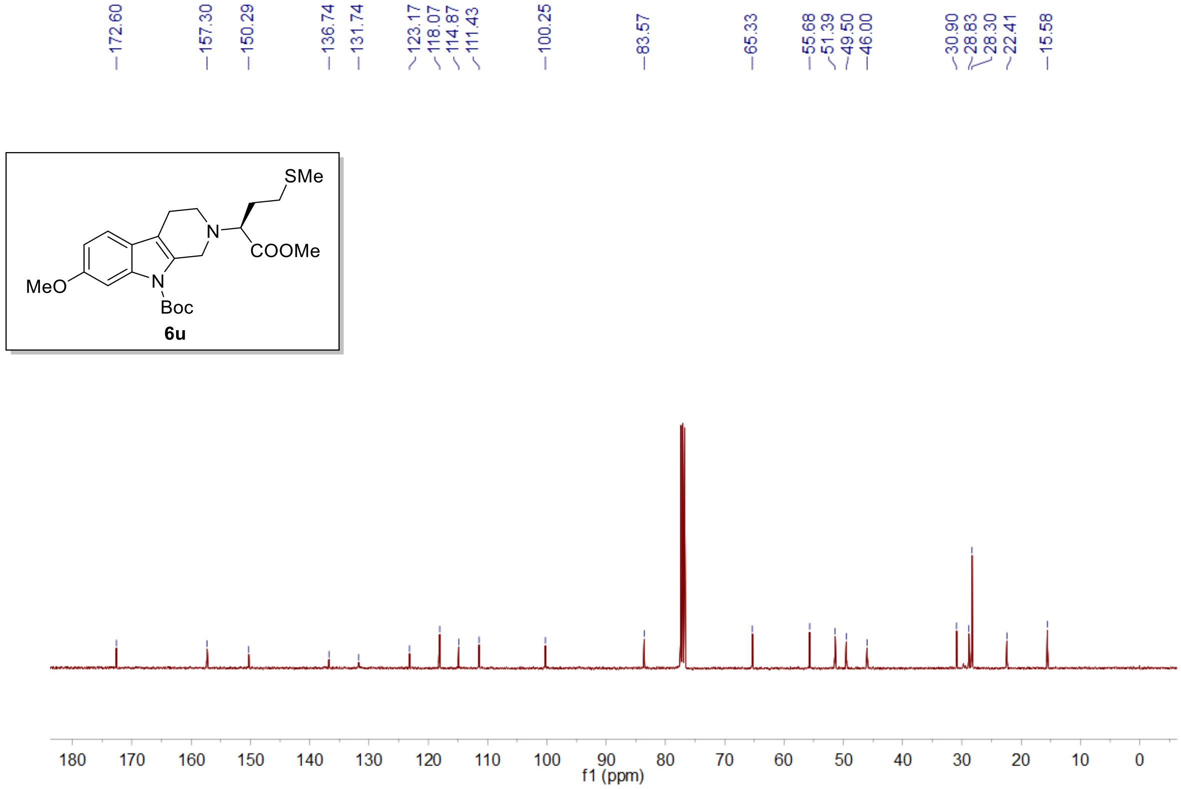

^{1}H-NMR spectrum of compound **6v** (400 MHz, $CDCl_3$)

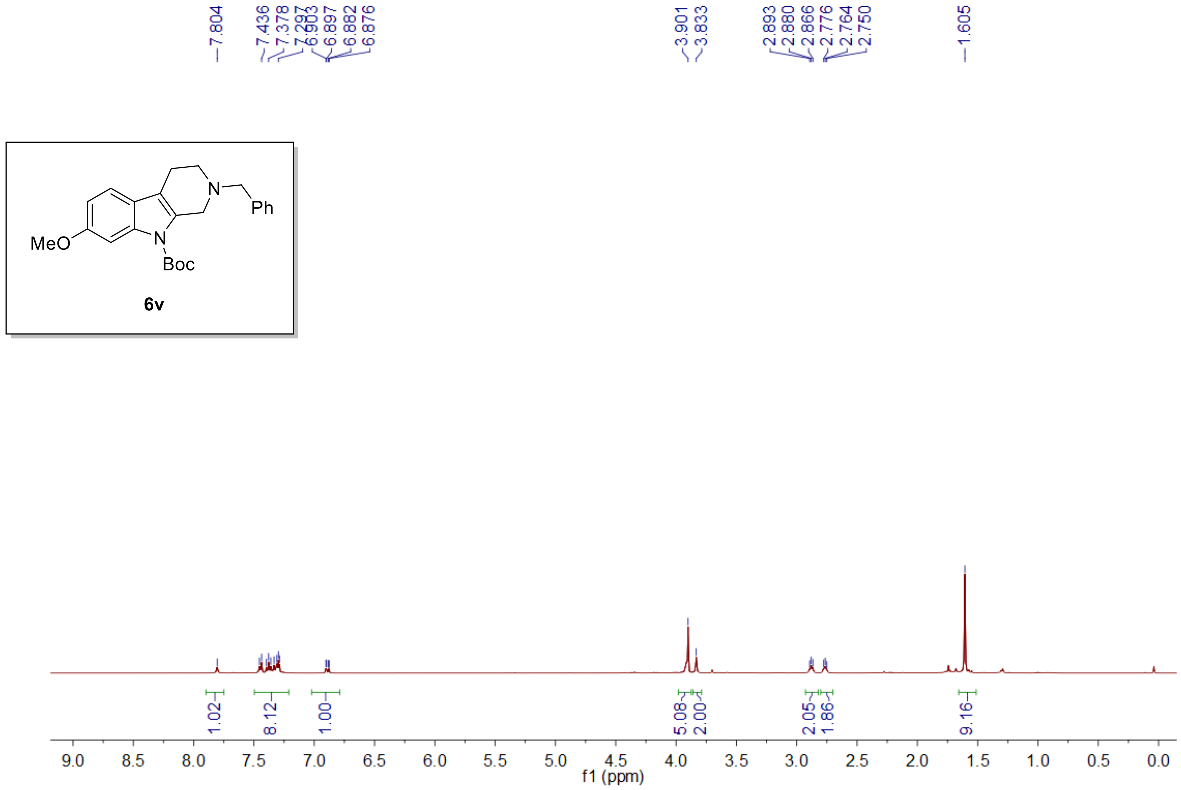

^{13}C-NMR spectrum of compound **6v** (101 MHz, $CDCl_3$)

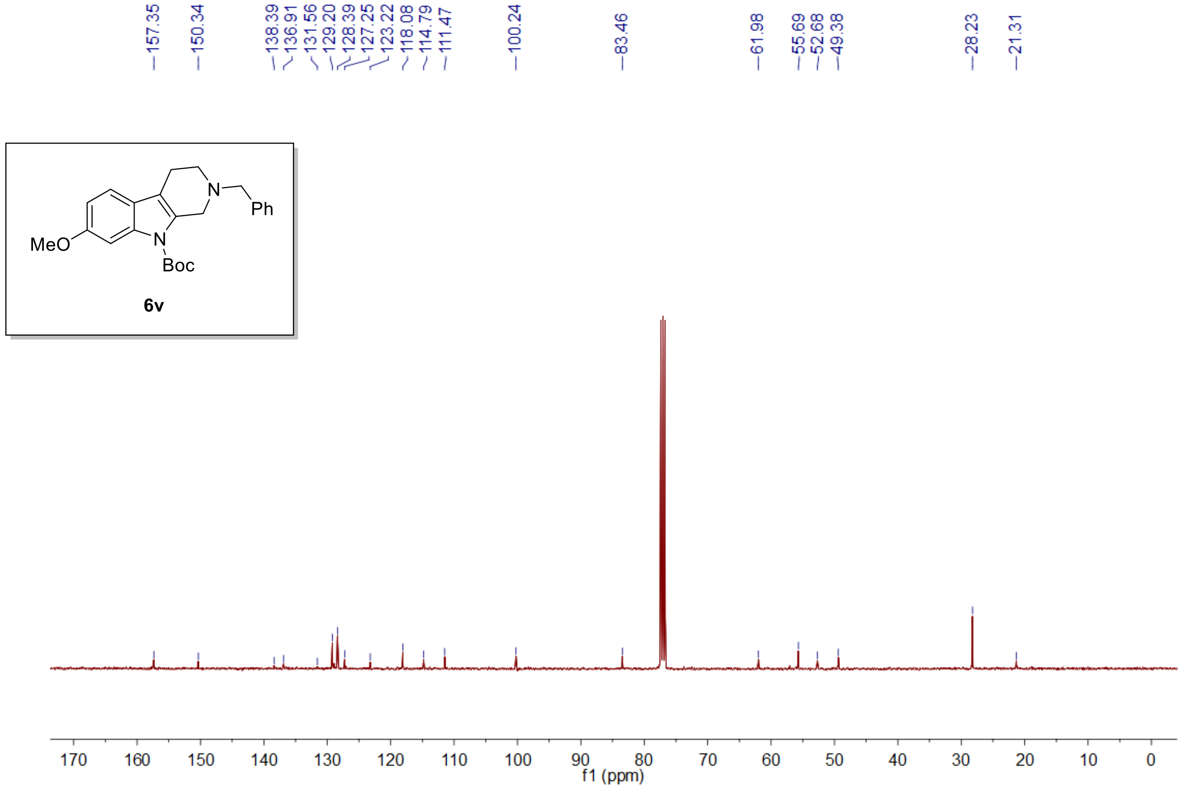

^{1}H-NMR spectrum of compound **6w** (400 MHz, $CDCl_3$)

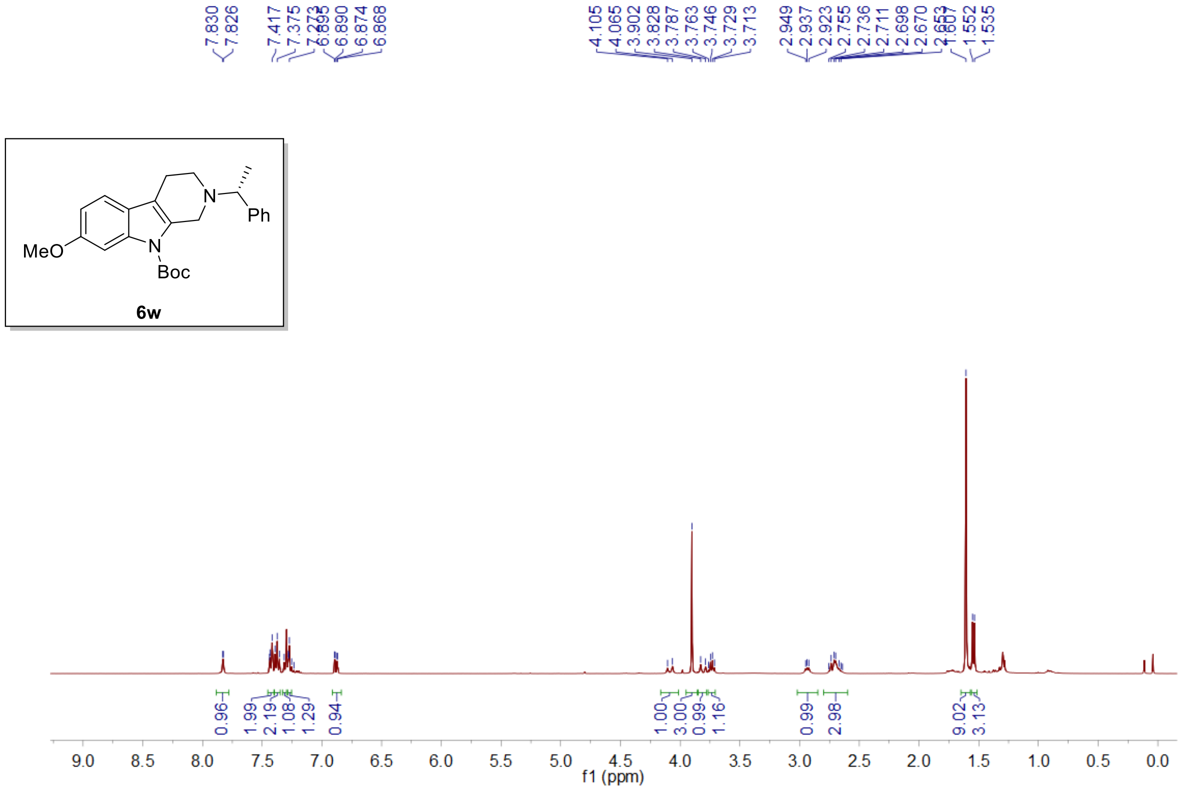

^{13}C-NMR spectrum of compound **6w** (101 MHz, $CDCl_3$)

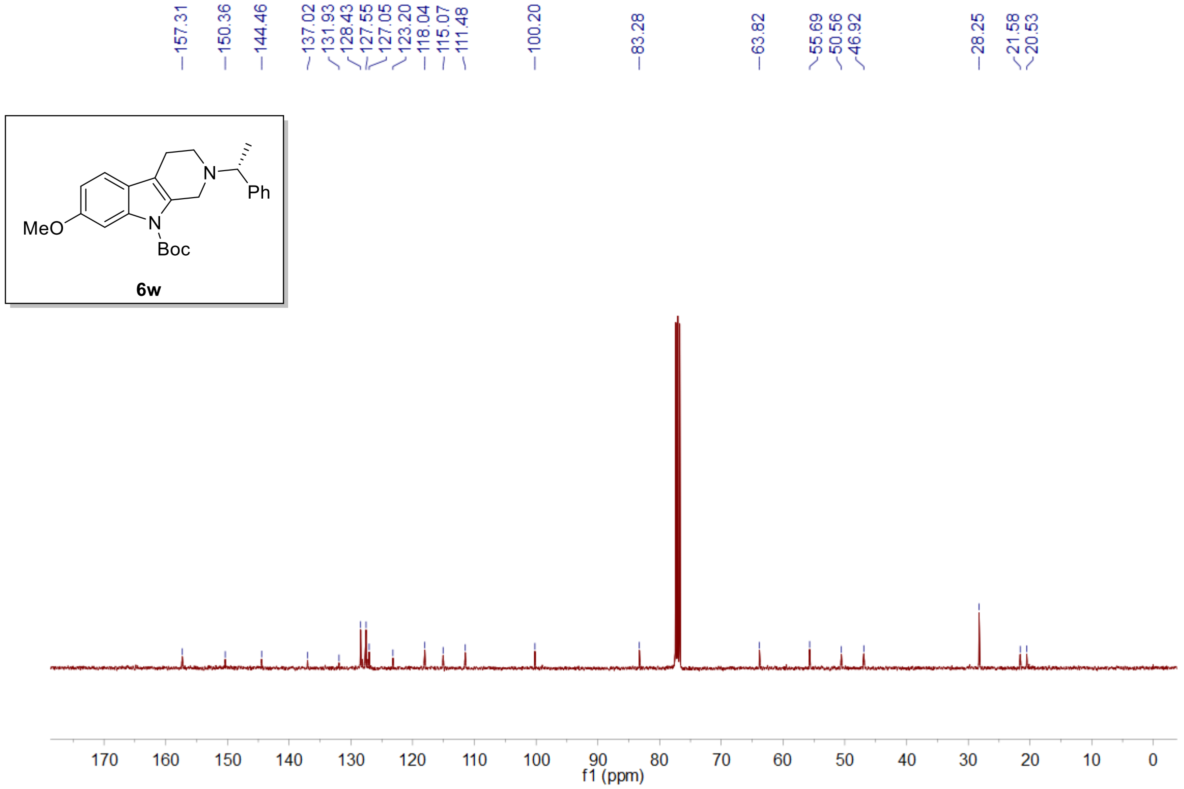

^{1}H-NMR spectrum of compound **6x** (400 MHz, $CDCl_3$)

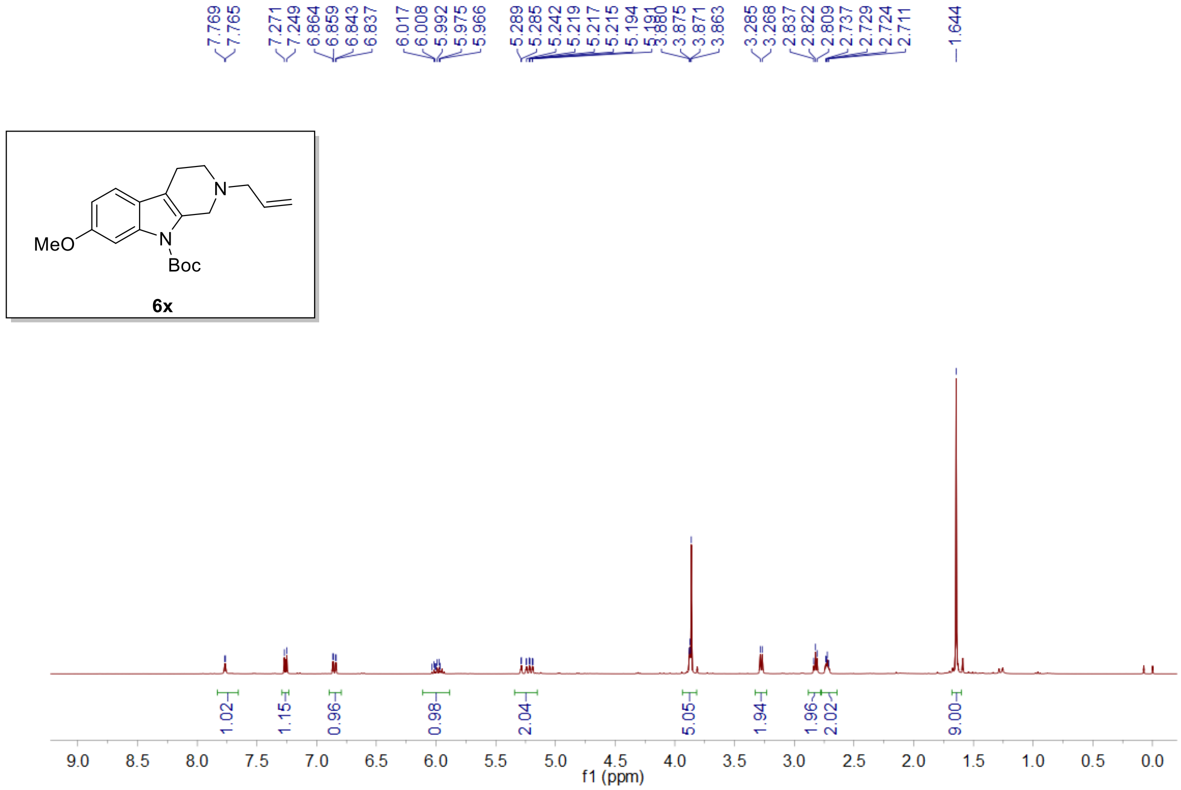

^{13}C-NMR spectrum of compound **6x** (101 MHz, $CDCl_3$)

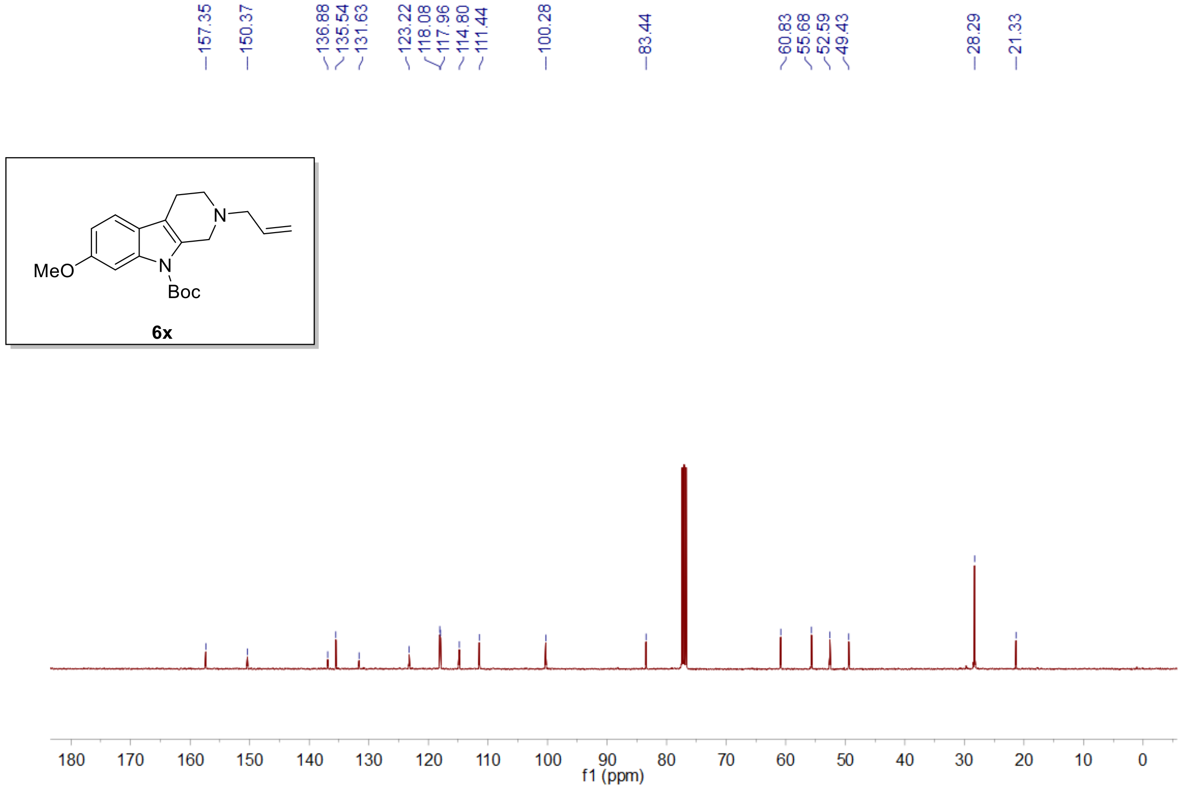

^{1}H-NMR spectrum of compound **6y** (400 MHz, $CDCl_3$)

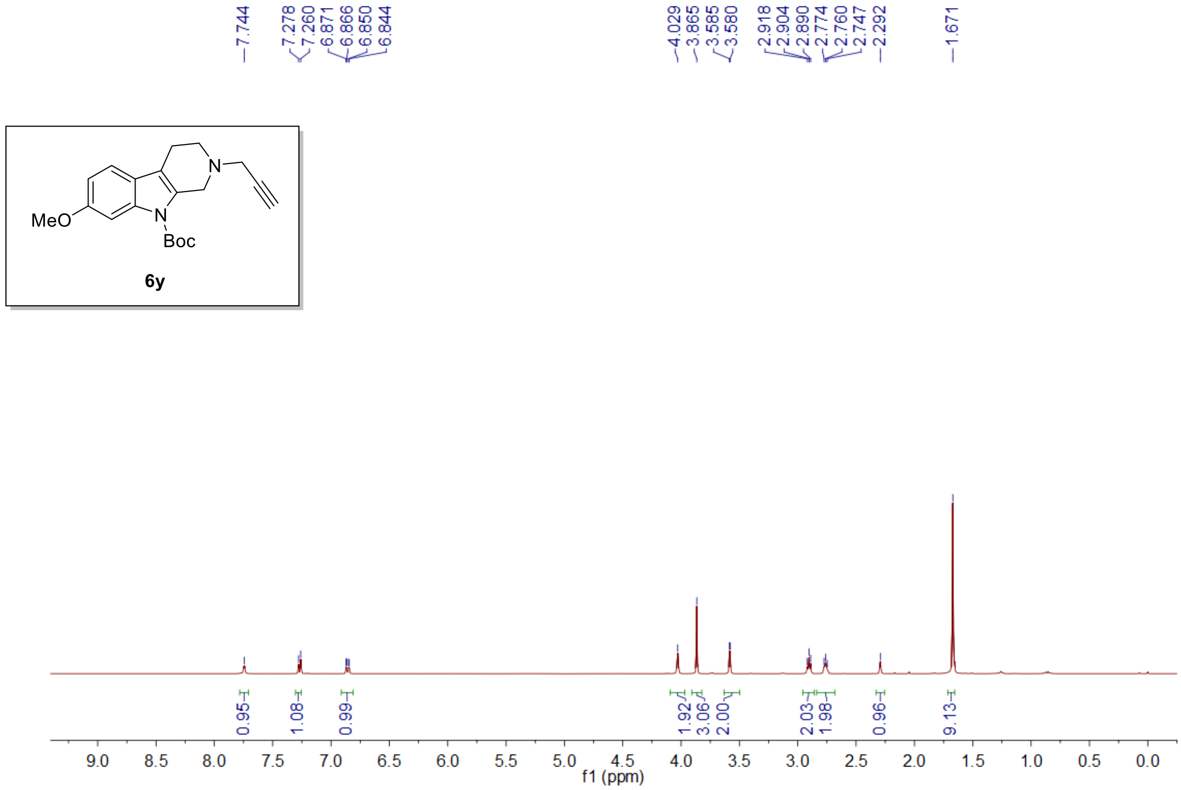

^{13}C-NMR spectrum of compound **6y** (101 MHz, $CDCl_3$)

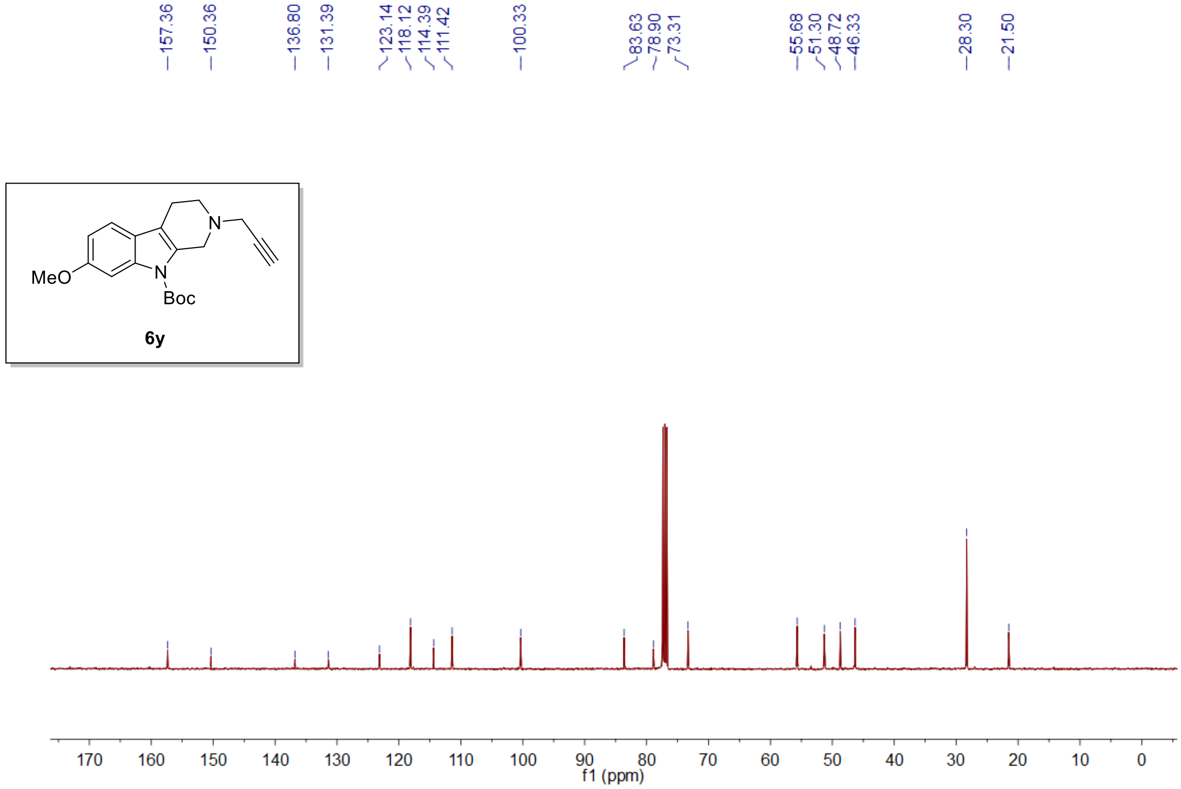

^{1}H-NMR spectrum of compound **6z** (400 MHz, $CDCl_3$)

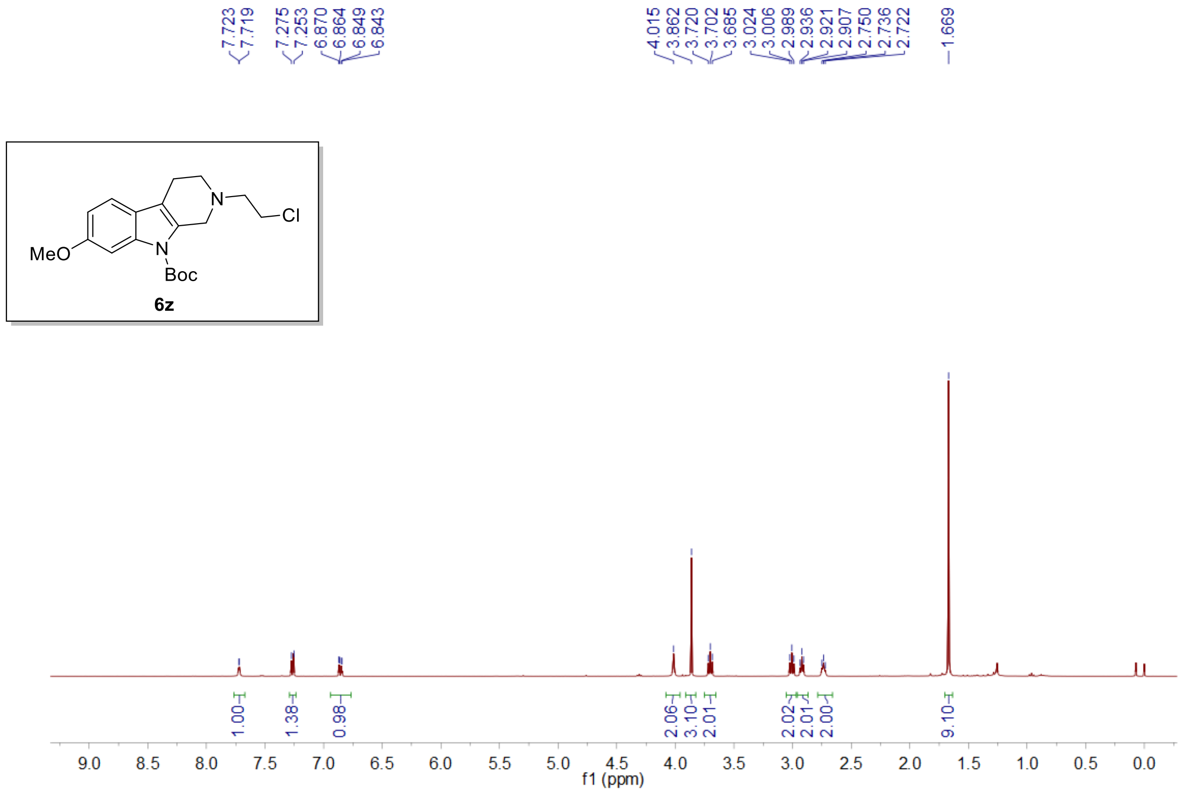

^{13}C-NMR spectrum of compound **6z** (101 MHz, $CDCl_3$)

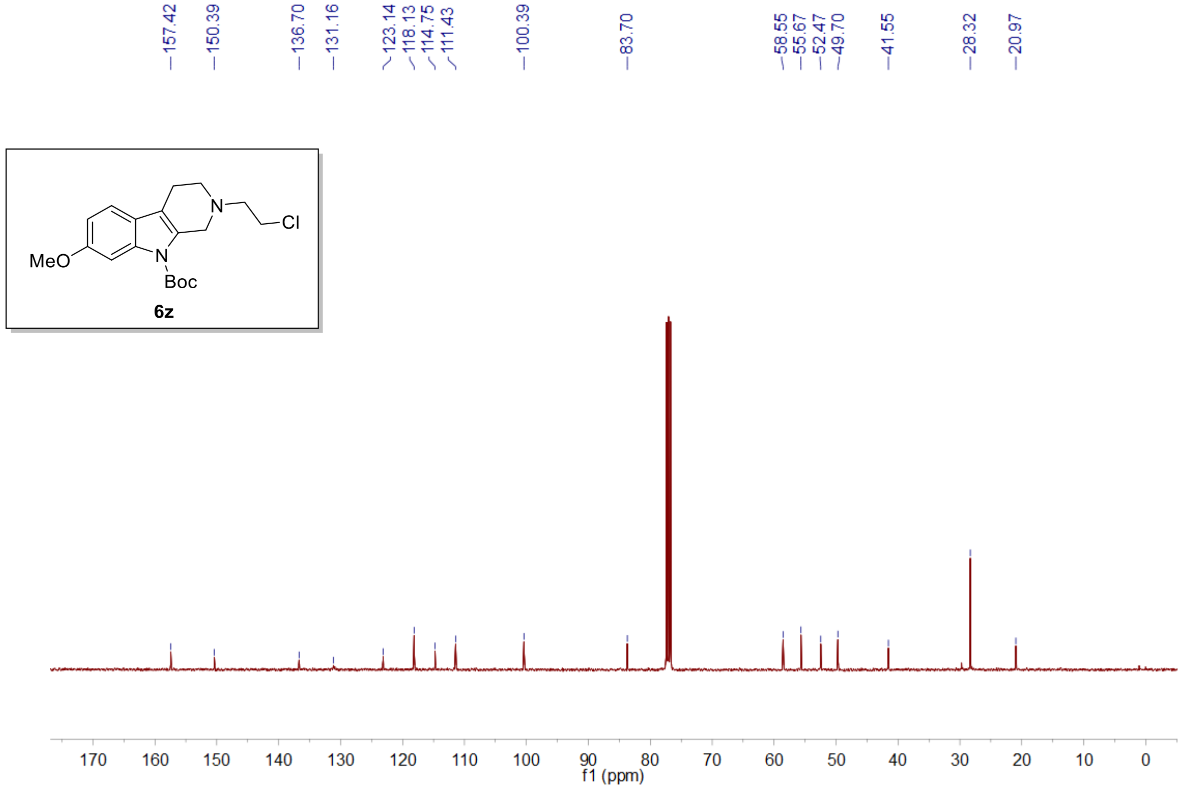

^{1}H-NMR spectrum of compound **6aa** (400 MHz, $CDCl_3$)

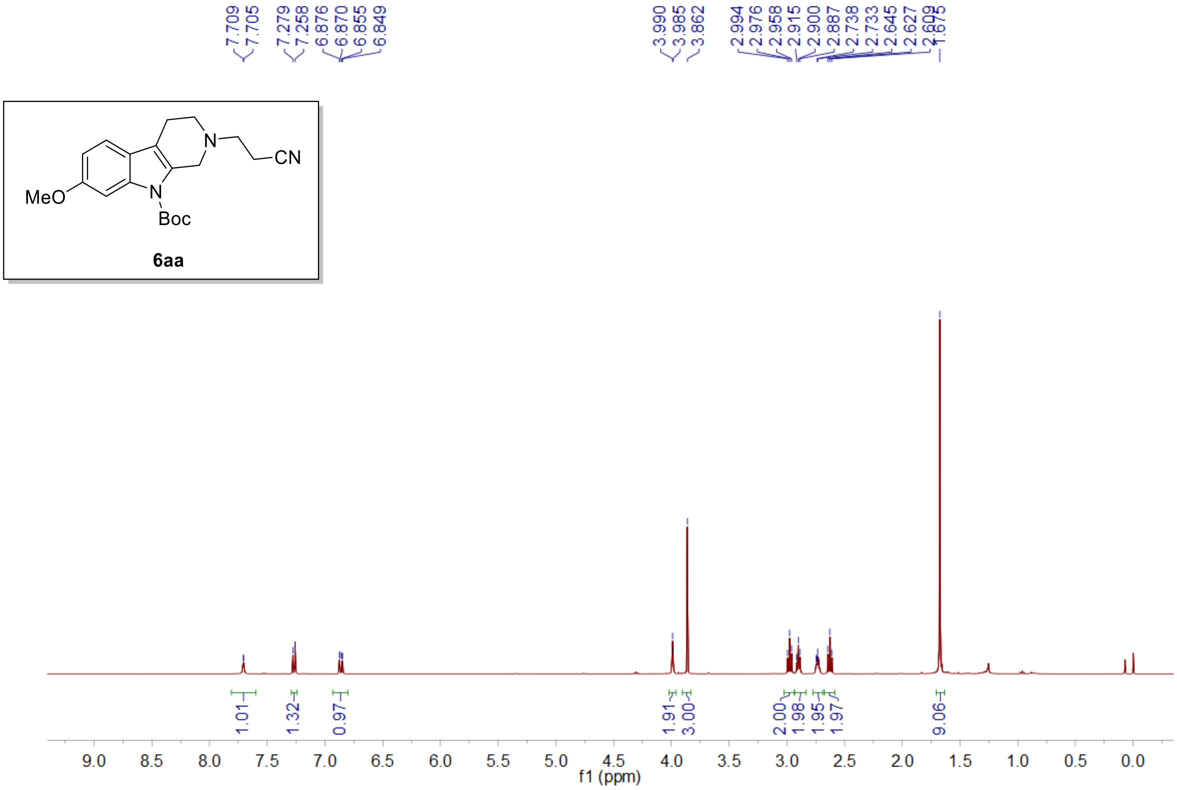

6aa

^{13}C-NMR spectrum of compound **6aa** (101 MHz, $CDCl_3$)

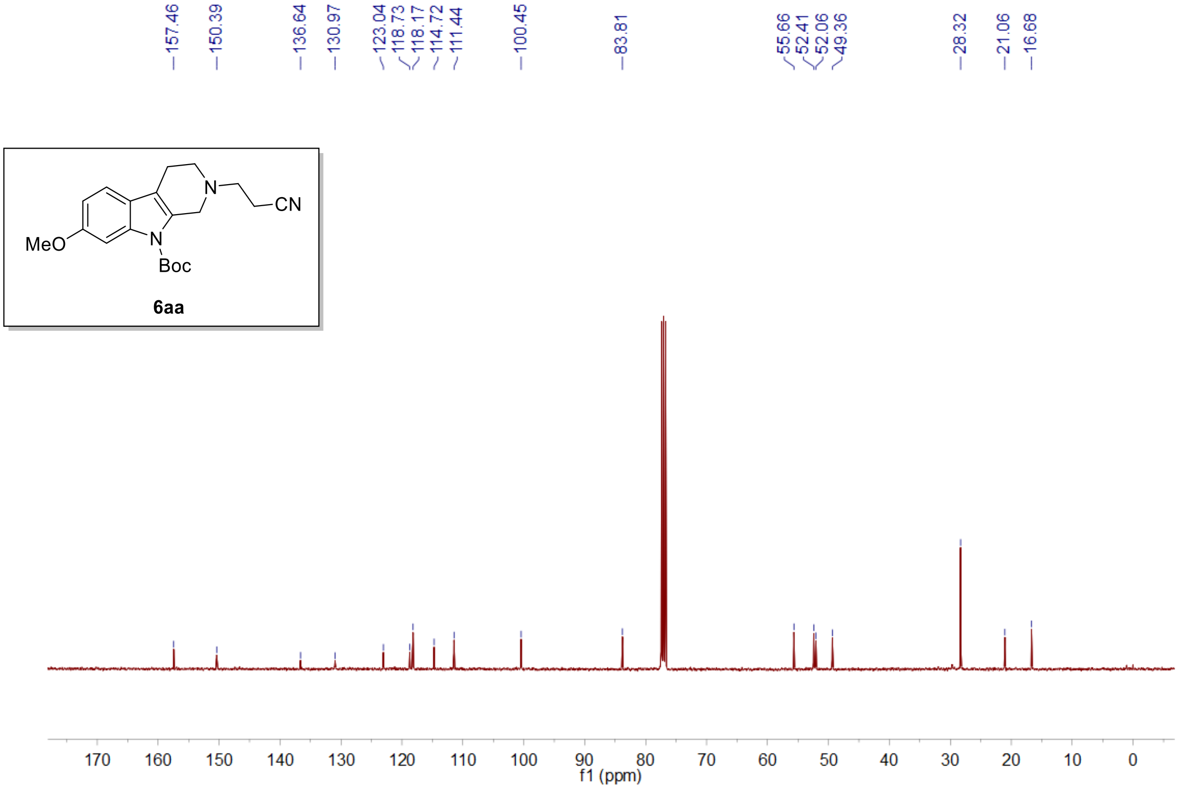

6aa

^{1}H-NMR spectrum of compound **6ab** (400 MHz, $CDCl_3$)

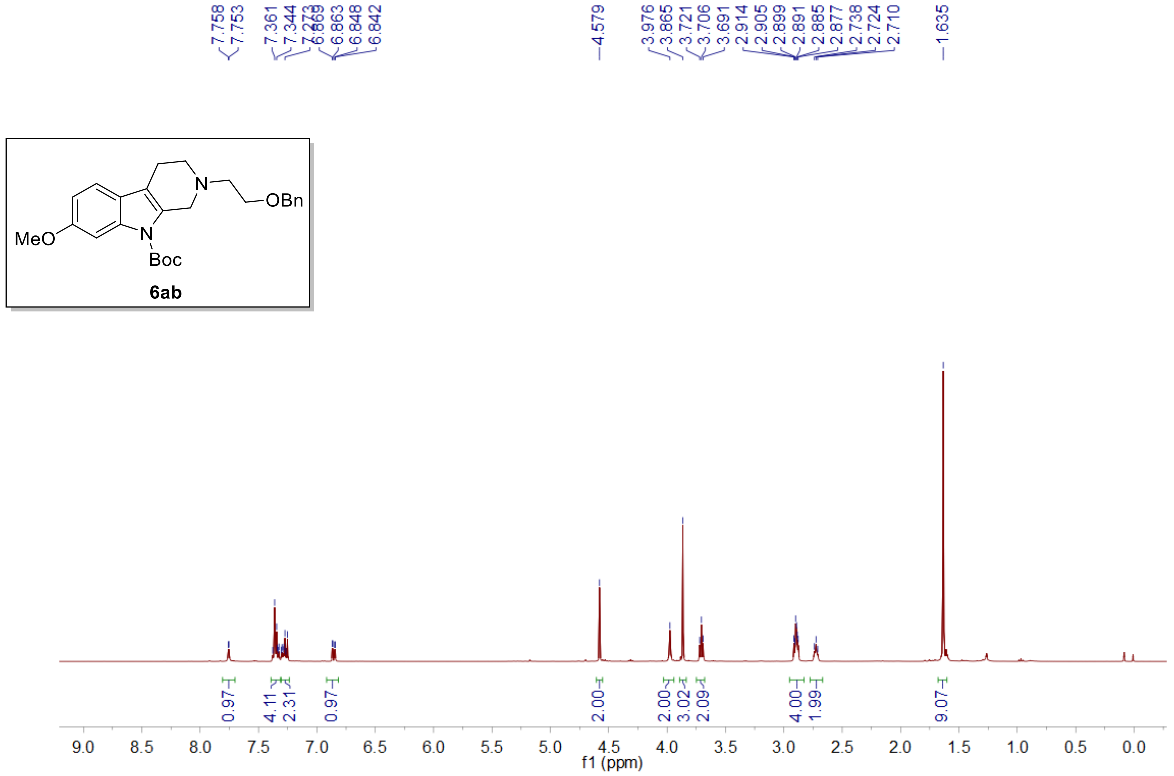

^{13}C-NMR spectrum of compound **6ab** (101 MHz, $CDCl_3$)

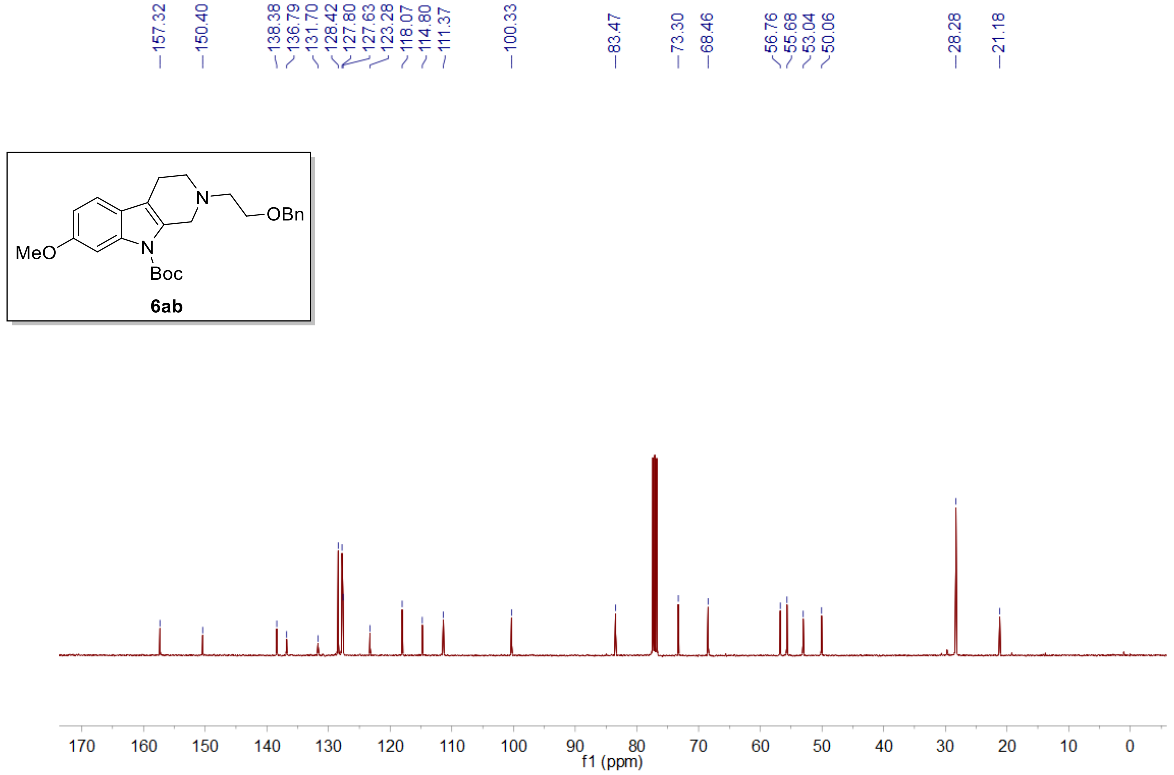

^{1}H-NMR spectrum of compound **6ac** (400 MHz, $CDCl_3$)

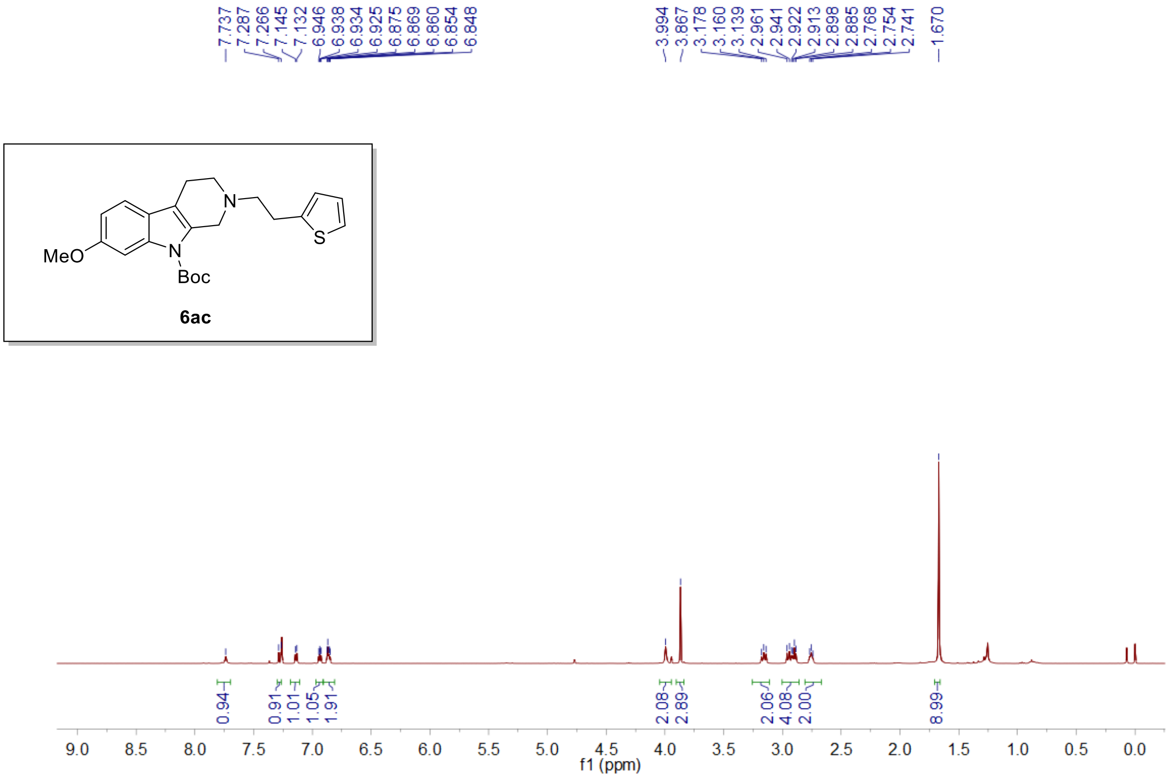

^{13}C-NMR spectrum of compound **6ac** (101 MHz, $CDCl_3$)

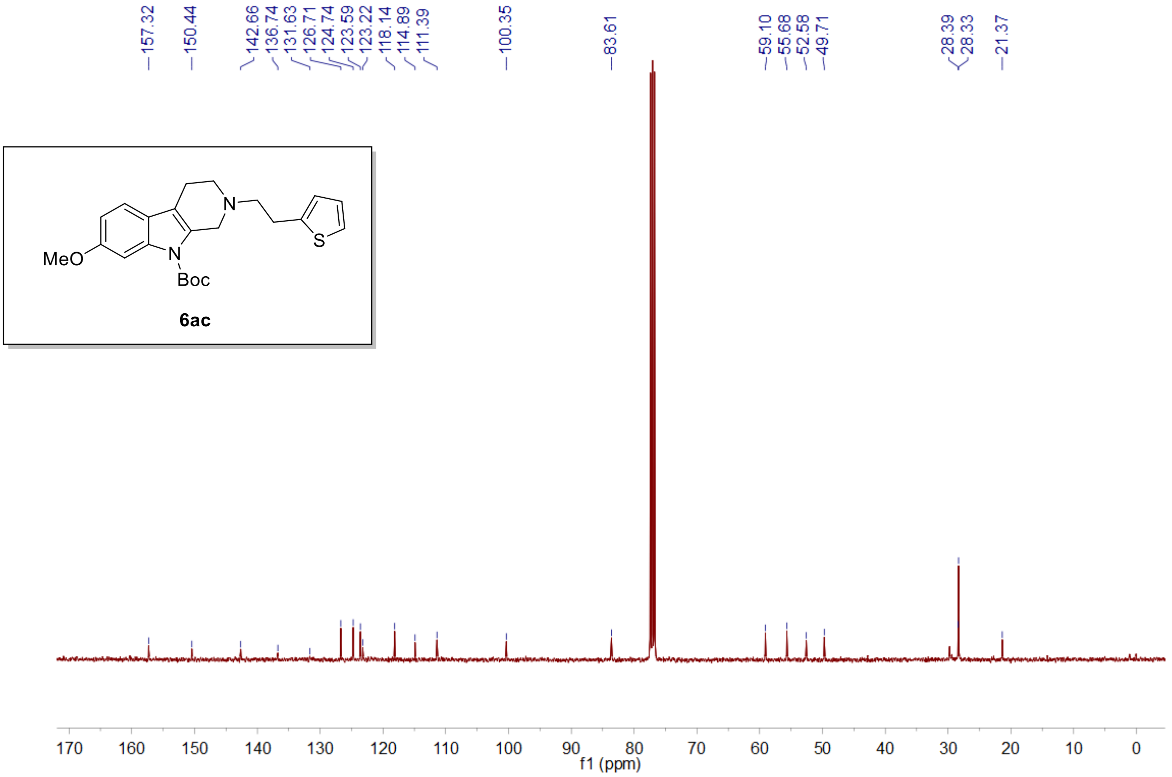

^{1}H-NMR spectrum of compound **6ad** (400 MHz, $CDCl_3$)

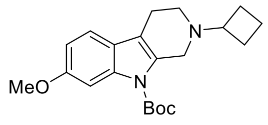

6ad

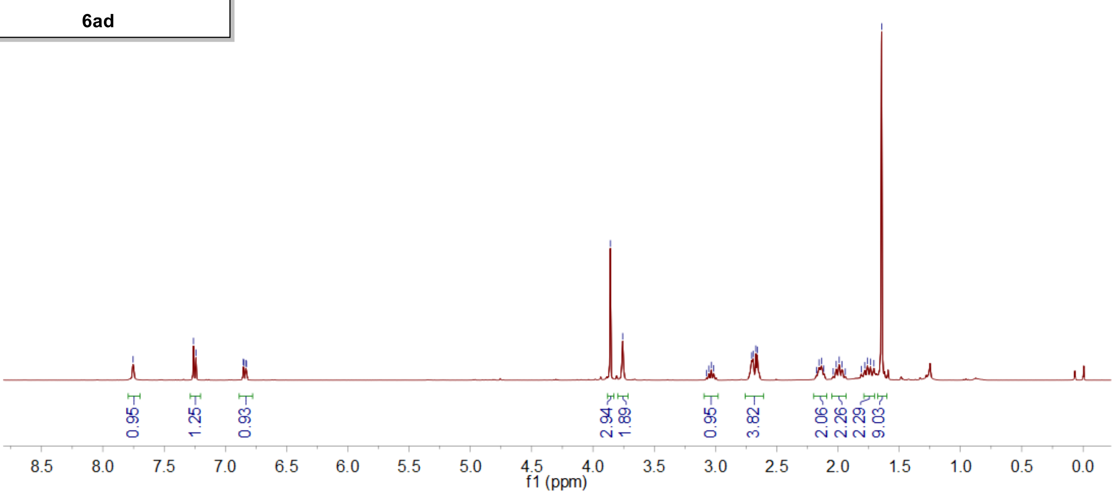

^{13}C-NMR spectrum of compound **6ad** (101 MHz, $CDCl_3$)

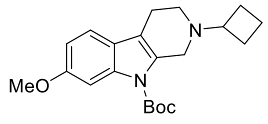

6ad

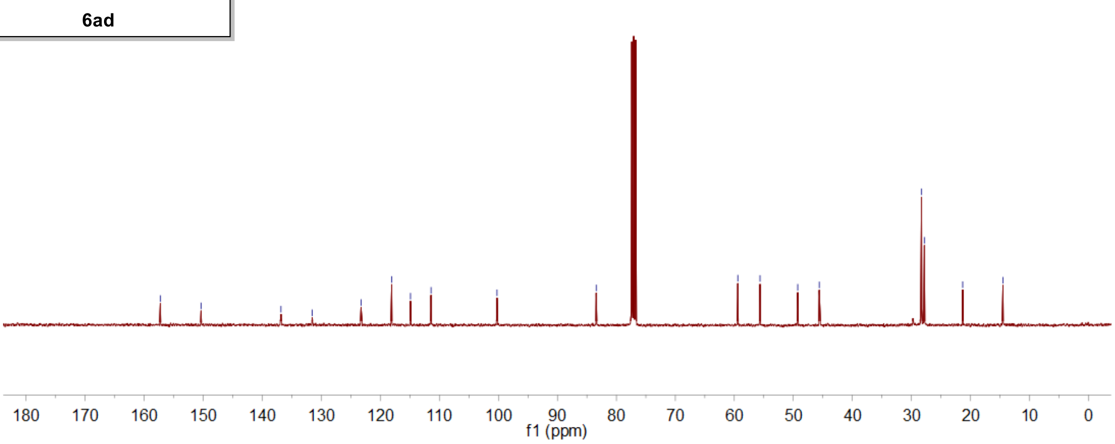

^{1}H-NMR spectrum of compound **6ae** (400 MHz, $CDCl_3$)

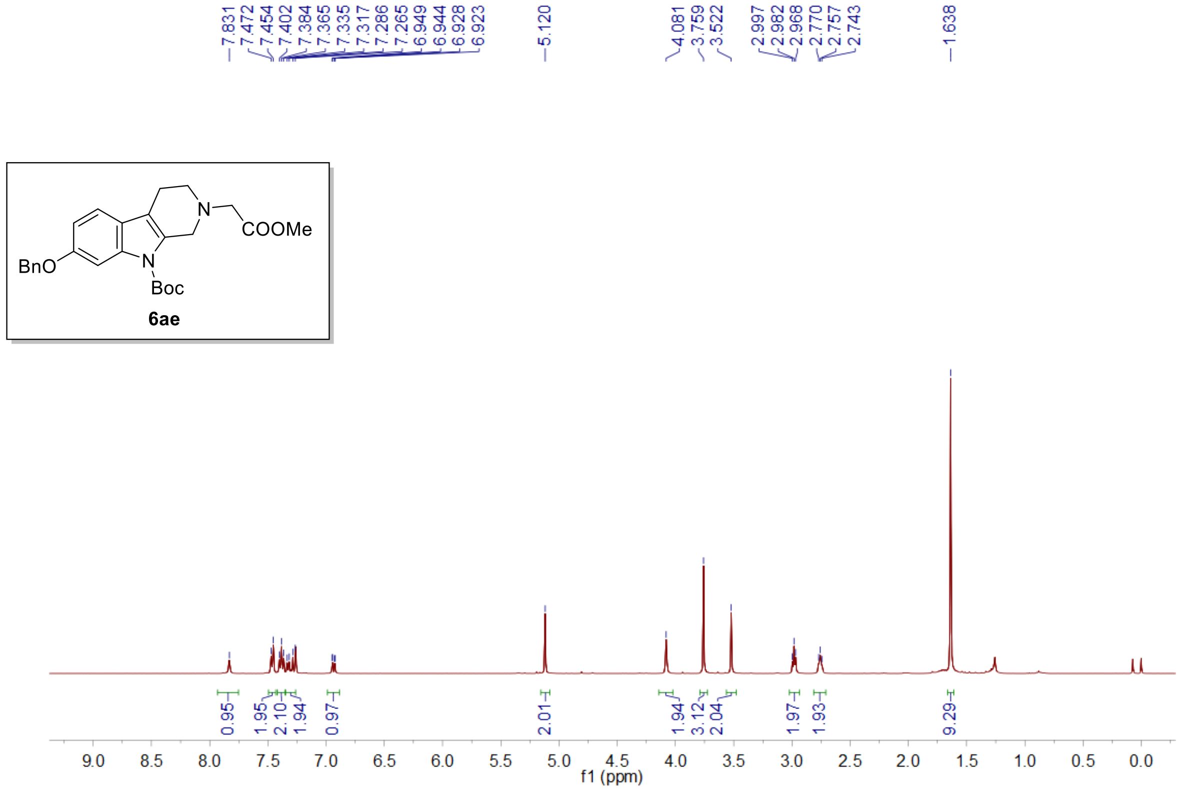

^{13}C-NMR spectrum of compound **6ae** (101 MHz, $CDCl_3$)

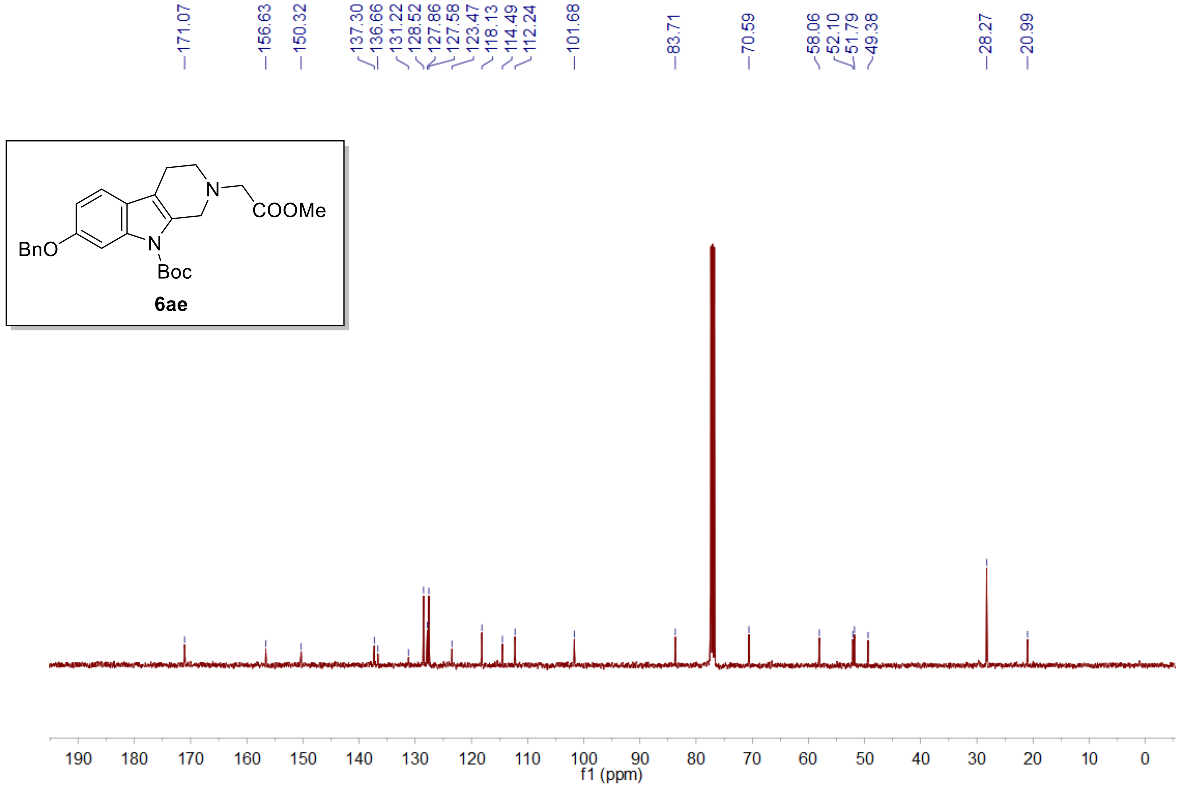

^{1}H-NMR spectrum of compound **6af** (400 MHz, $CDCl_3$)

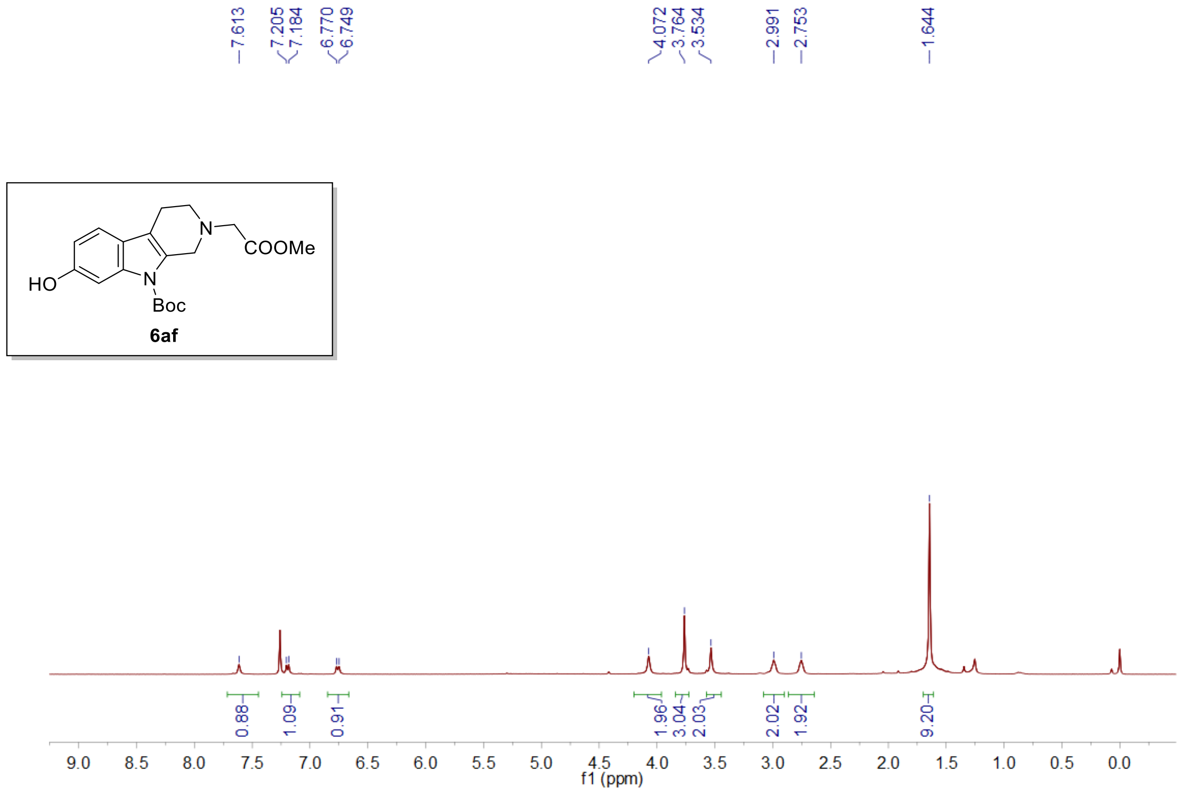

^{13}C-NMR spectrum of compound **6af** (101 MHz, $CDCl_3$)

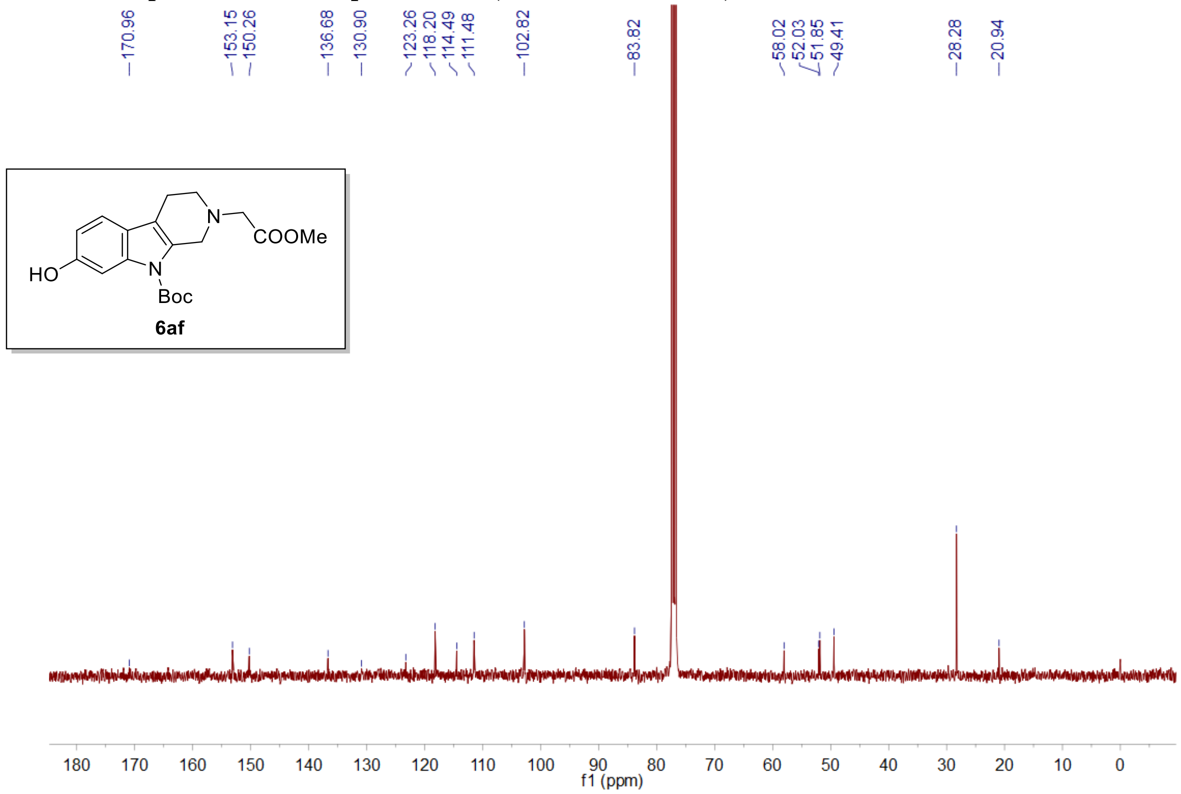

^{1}H-NMR spectrum of compound **6ag** (400 MHz, $CDCl_3$)

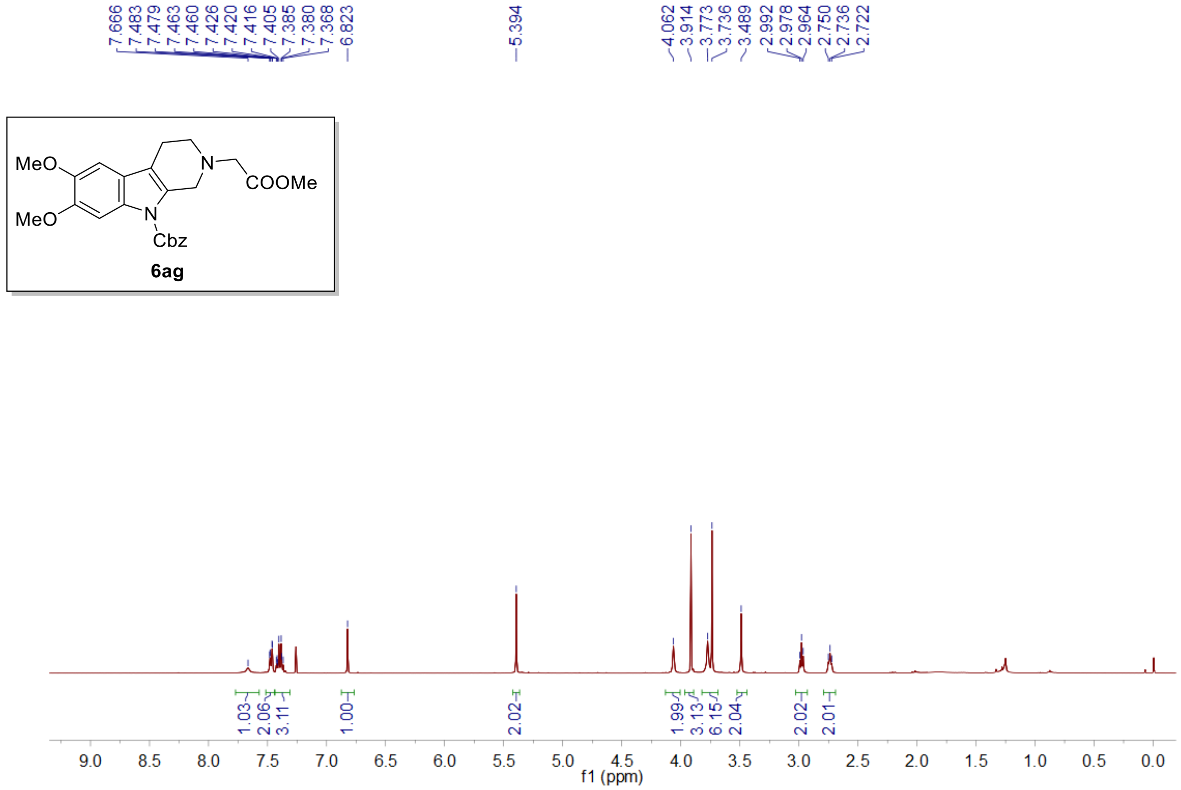

^{13}C-NMR spectrum of compound **6ag** (101 MHz, $CDCl_3$)

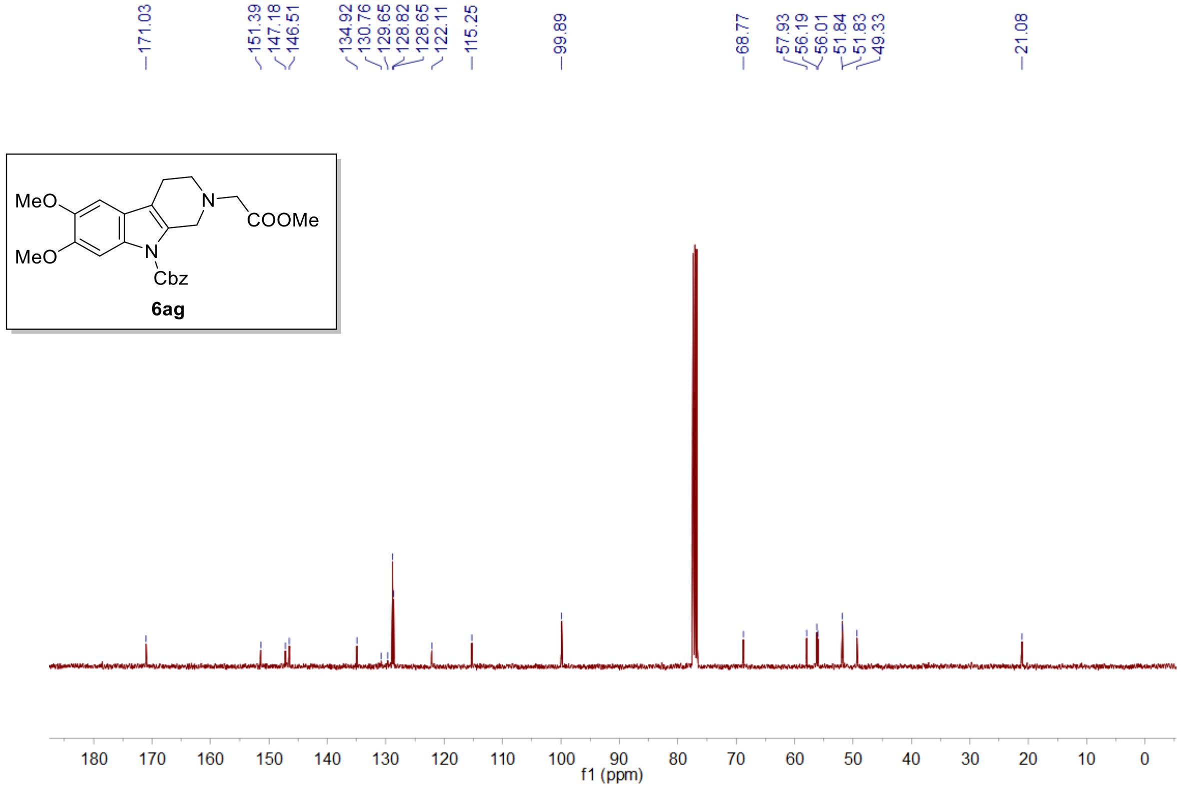

^{1}H-NMR spectrum of compound **6ah** (400 MHz, $CDCl_3$)

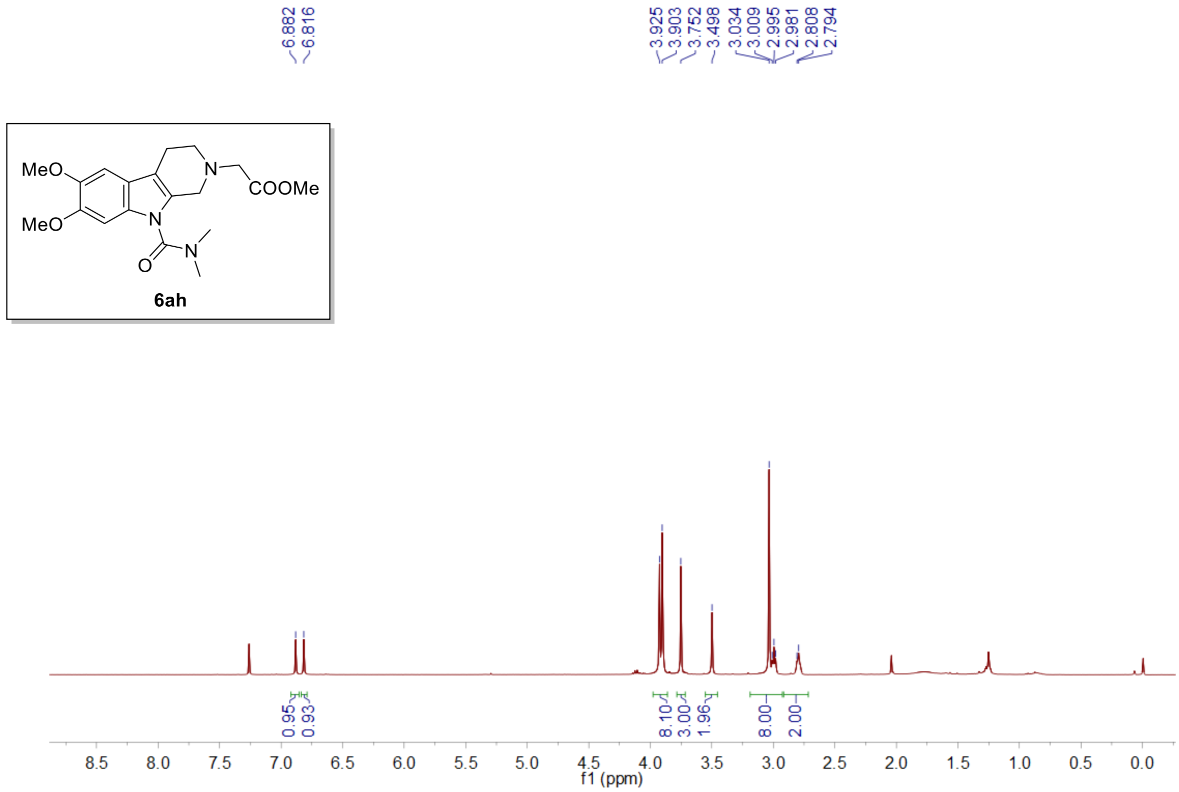

^{13}C-NMR spectrum of compound **6ah** (101 MHz, $CDCl_3$)

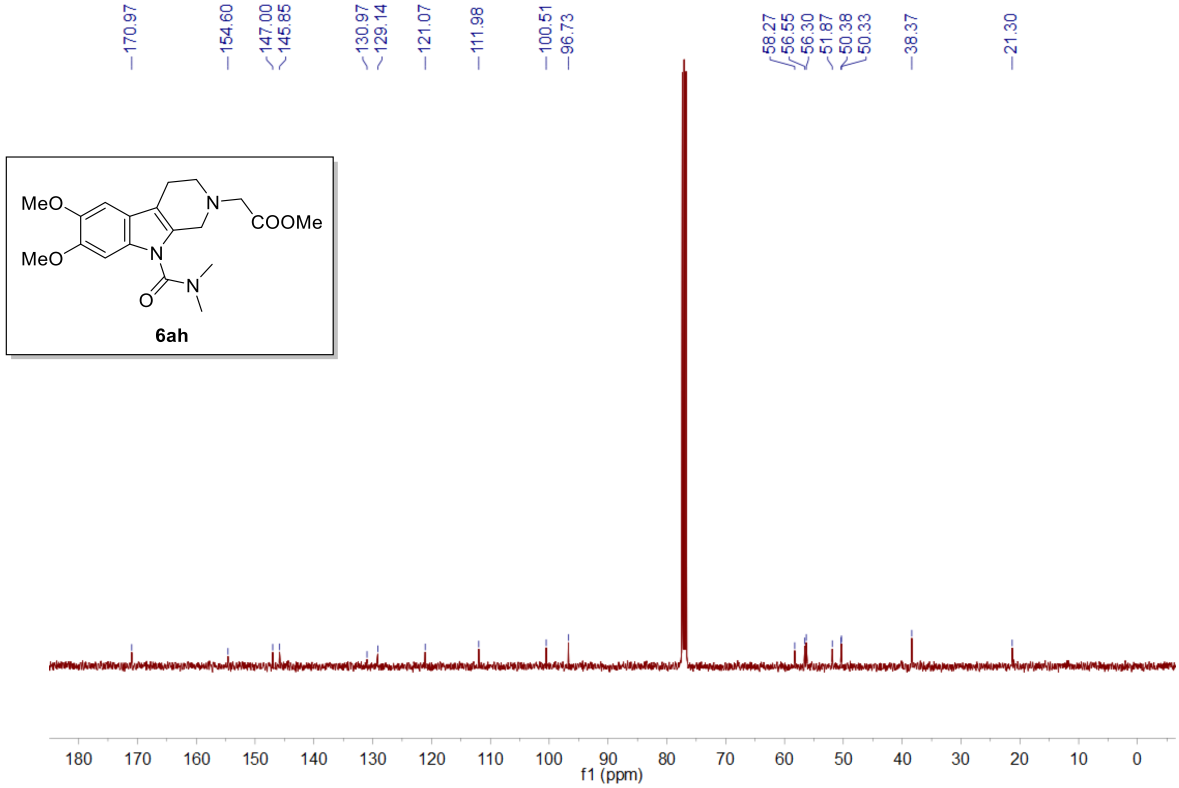

^{1}H-NMR spectrum of compound **8** (400 MHz, $CDCl_3$)

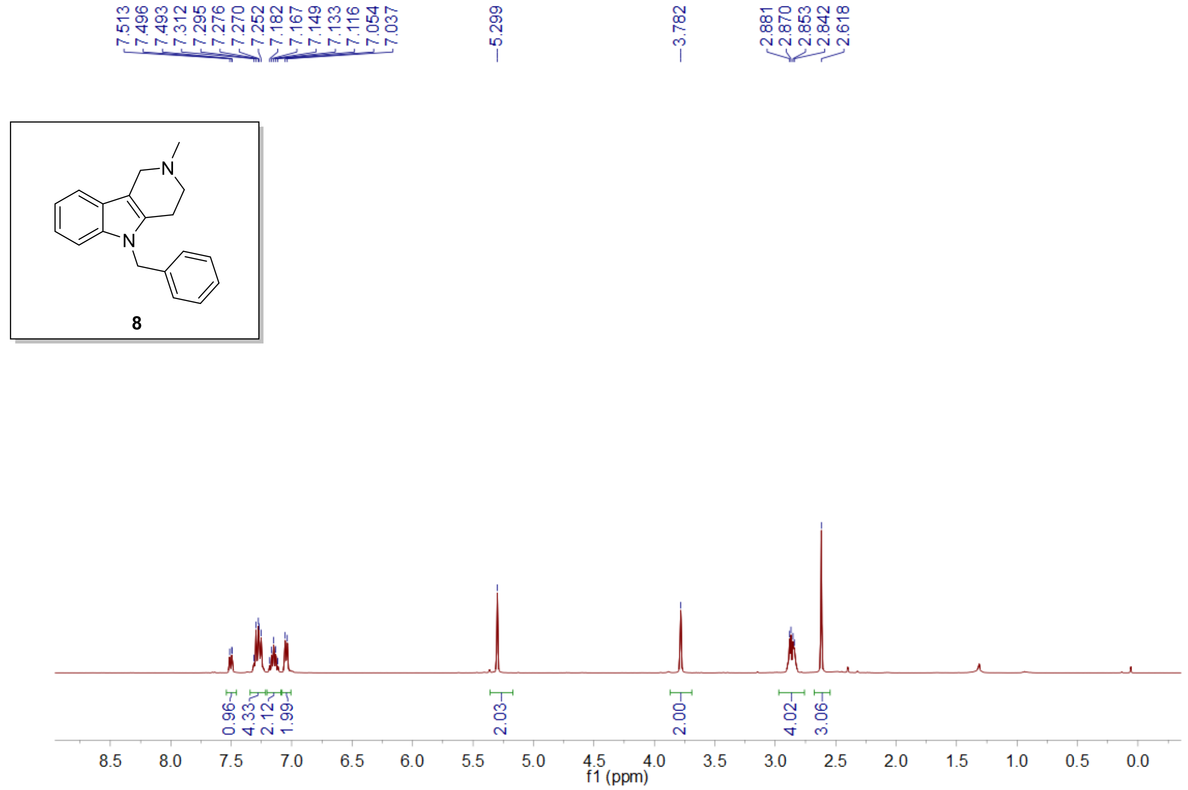

^{13}C-NMR spectrum of compound **8** (101 MHz, $CDCl_3$)

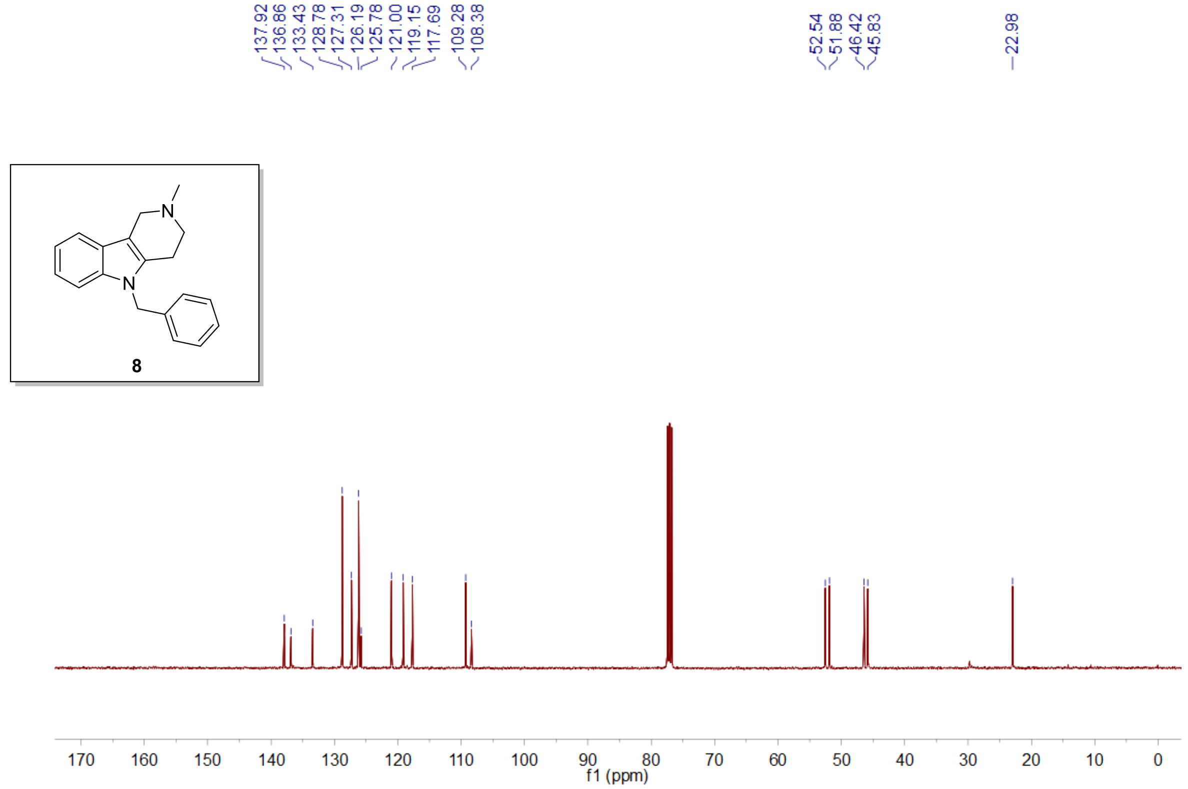

^{1}H-NMR spectrum of compound **9a** (400 MHz, $CDCl_3$)

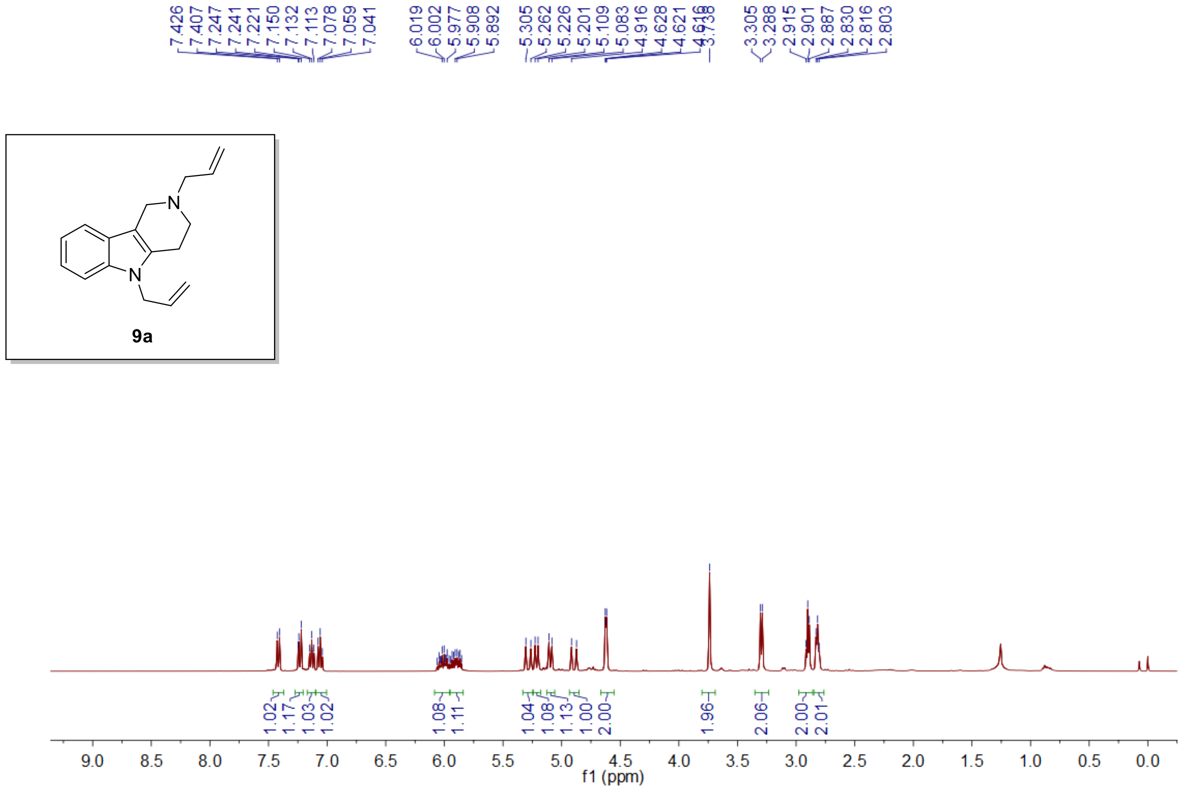

^{13}C-NMR spectrum of compound **9a** (101 MHz, $CDCl_3$)

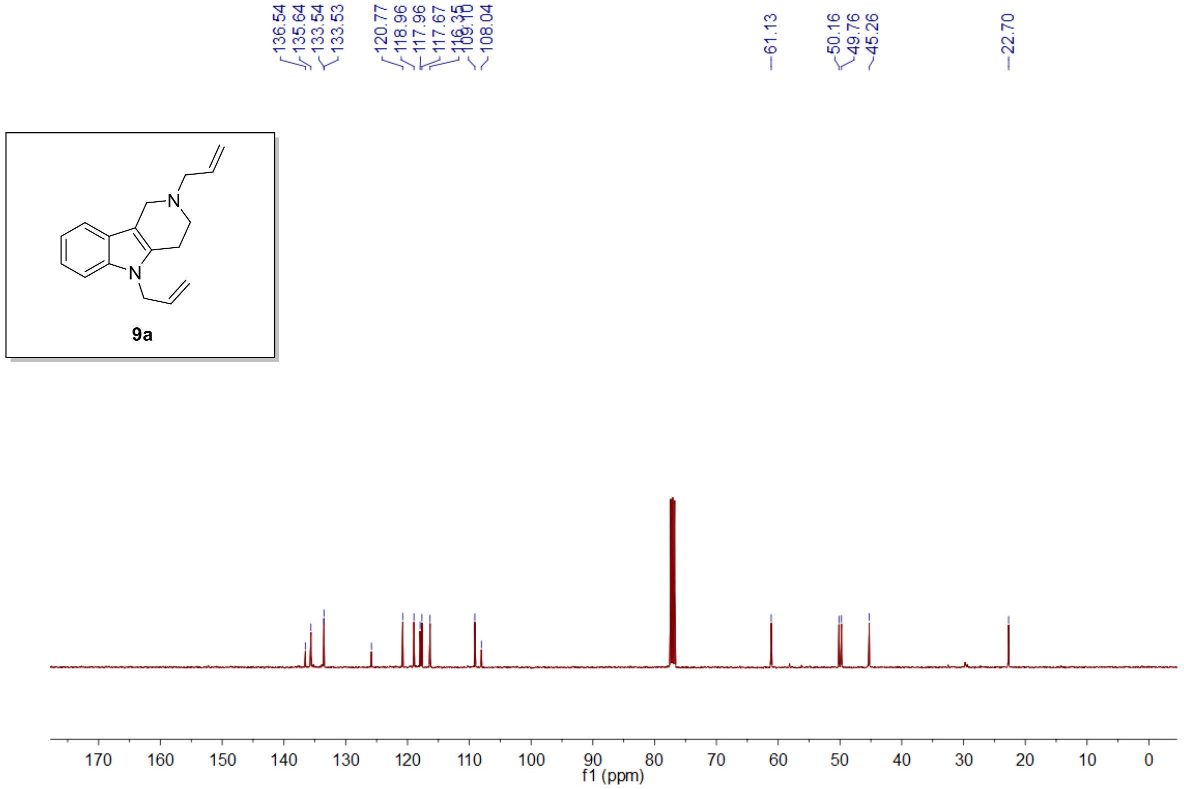

^{1}H-NMR spectrum of compound **9** (400 MHz, MeOD)

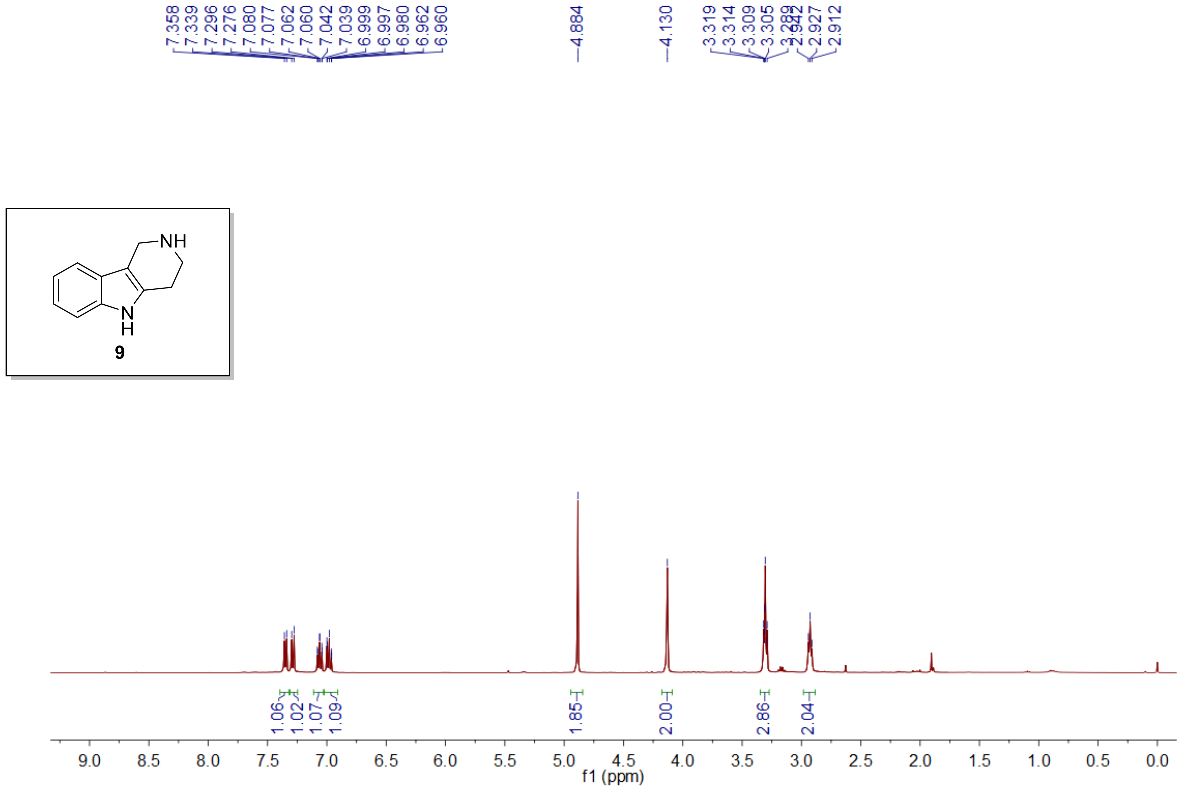

^{13}C-NMR spectrum of compound **9** (101 MHz, MeOD)

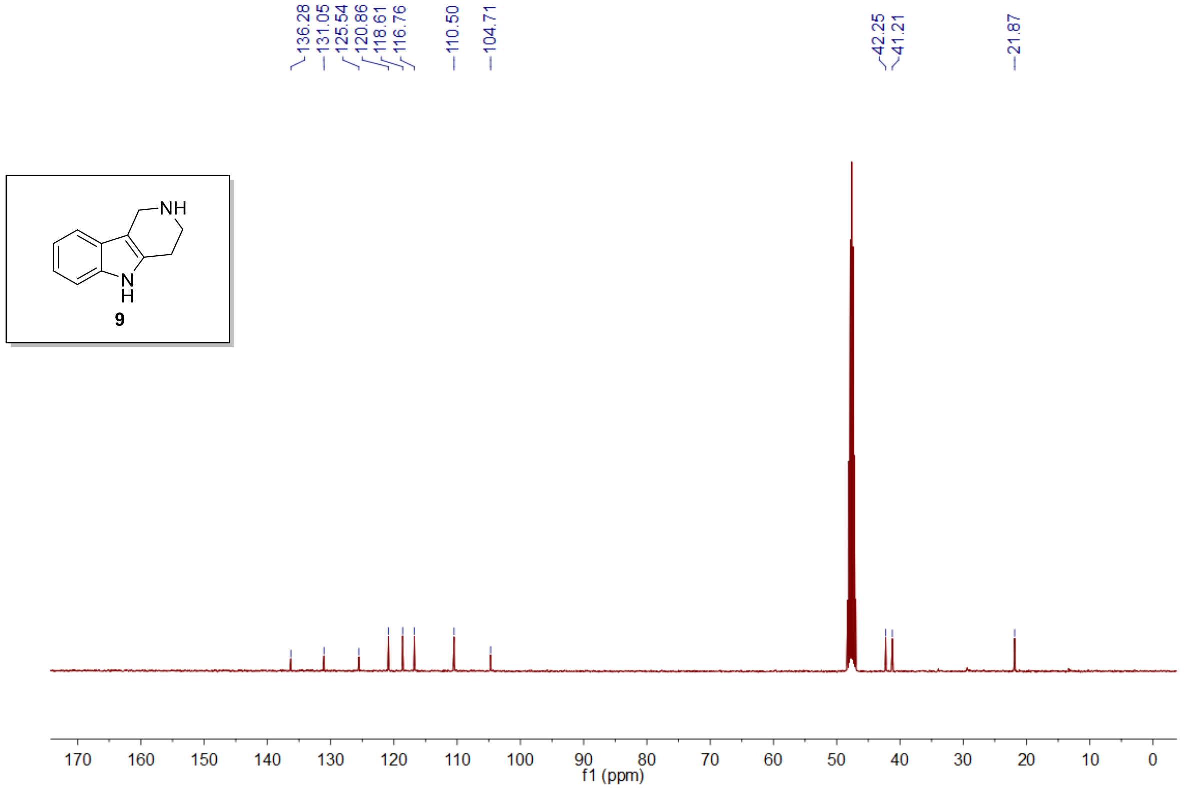

^{1}H-NMR spectrum of compound **10** (400 MHz, $CDCl_3$)

10

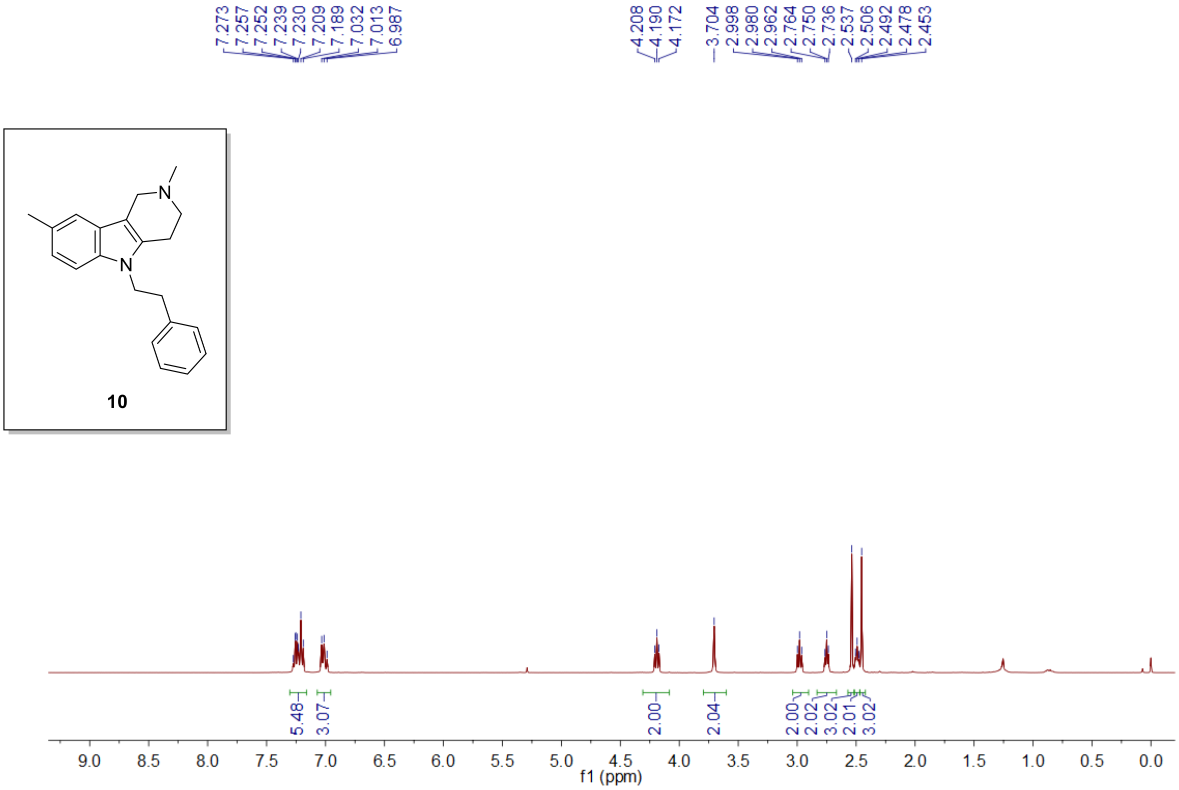

^{13}C-NMR spectrum of compound **10** (101 MHz, $CDCl_3$)

10

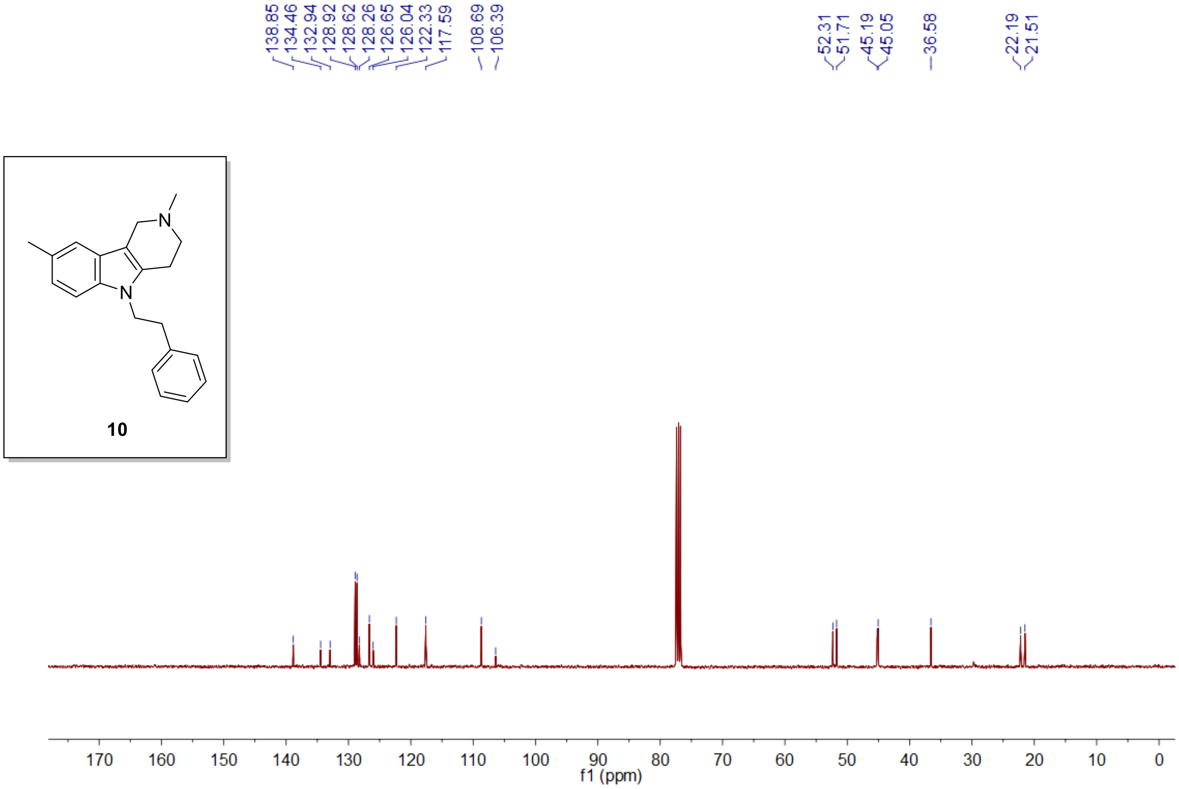

^{1}H-NMR spectrum of compound **11** (400 MHz, $CDCl_3$)

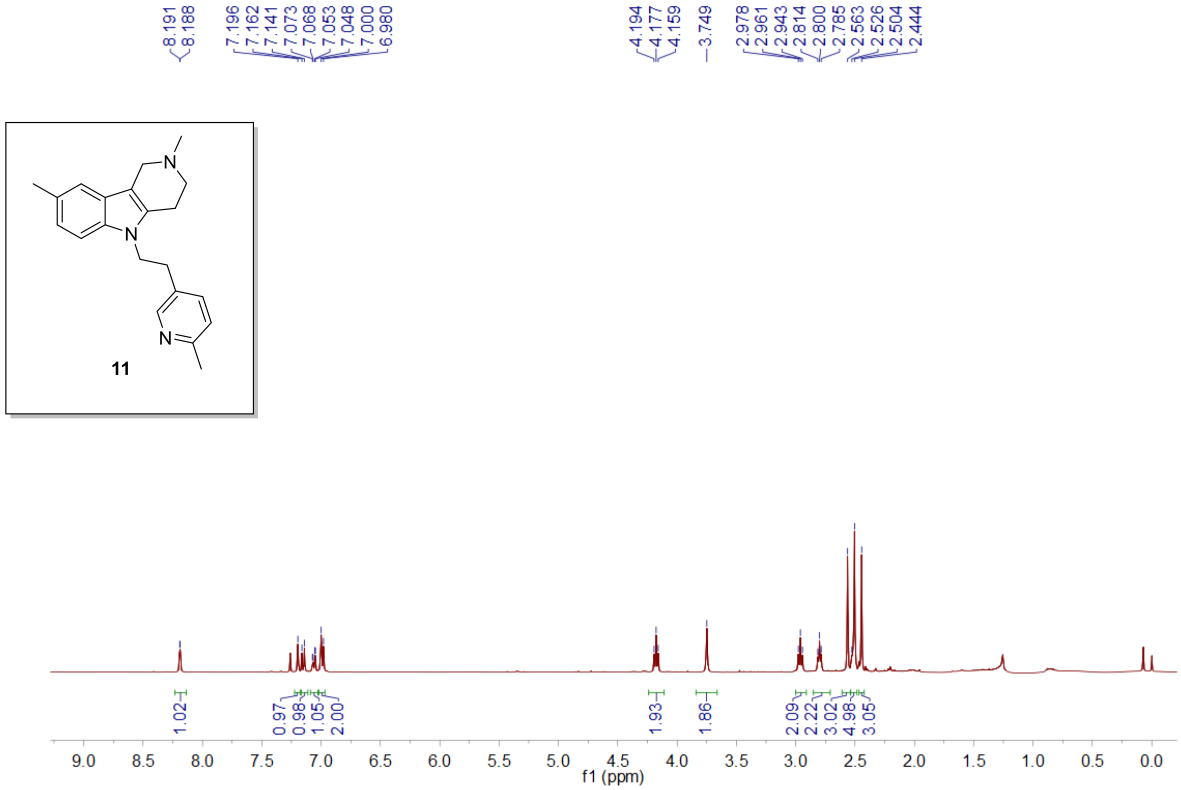

^{13}C-NMR spectrum of compound **11** (101 MHz, $CDCl_3$)

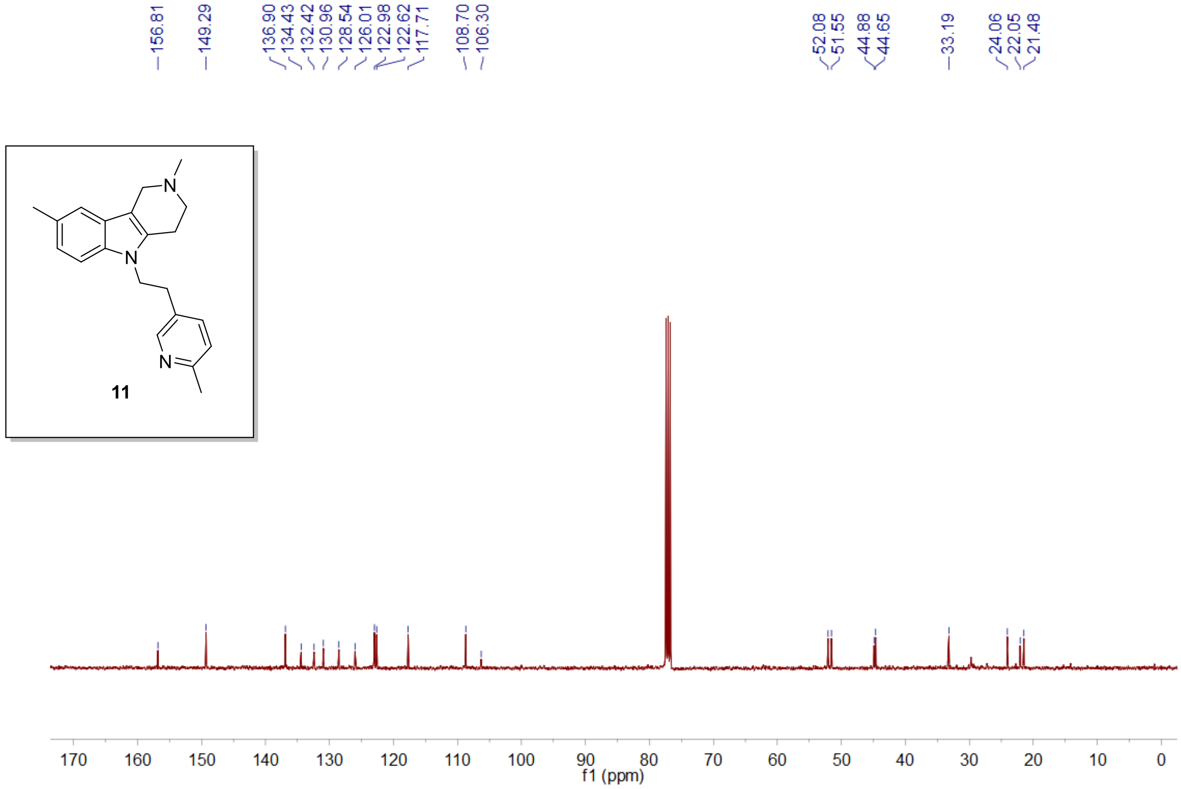

^{1}H-NMR spectrum of compound **12** (400 MHz, $CDCl_3$)

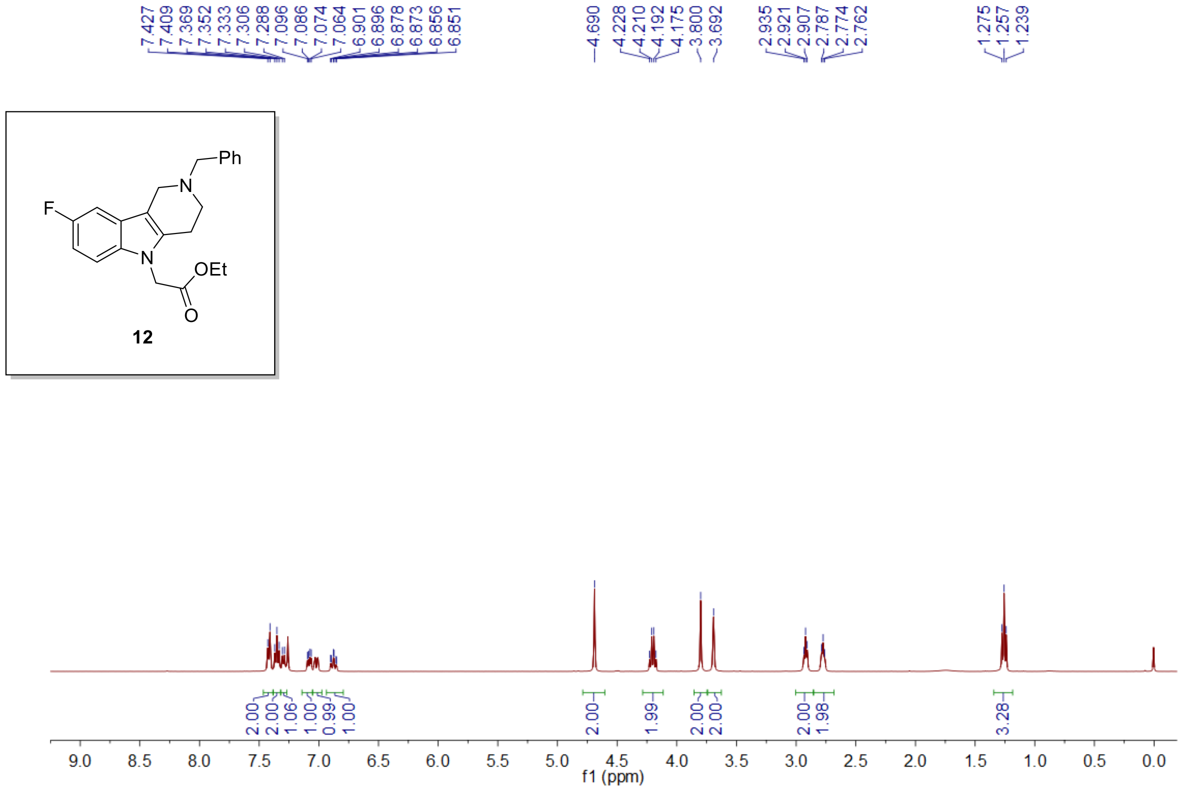

^{13}C-NMR spectrum of compound **12** (101 MHz, $CDCl_3$)

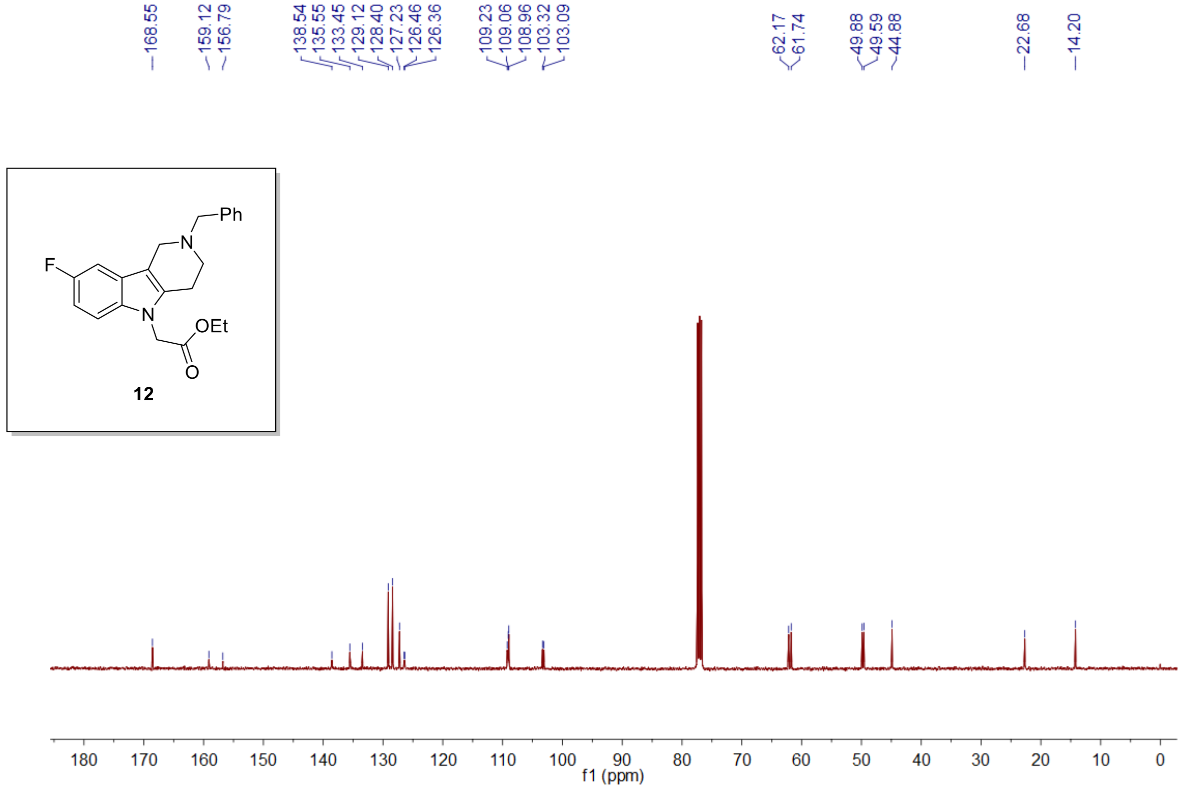

^{19}F-NMR spectrum of compound **12** (565 MHz, $CDCl_3$)

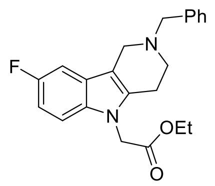

12

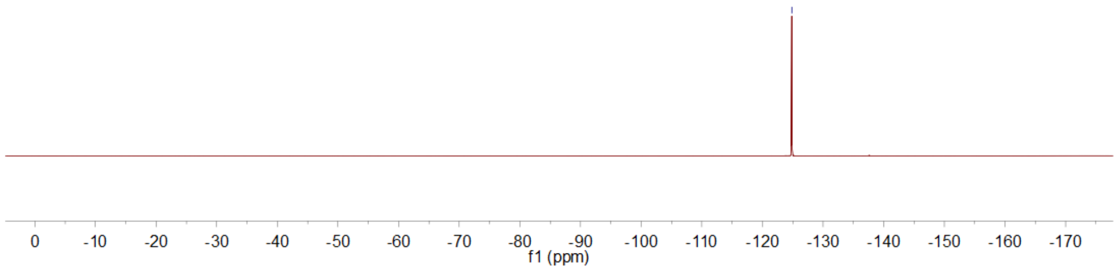

^{1}H-NMR spectrum of compound **13a** (400 MHz, MeOD)

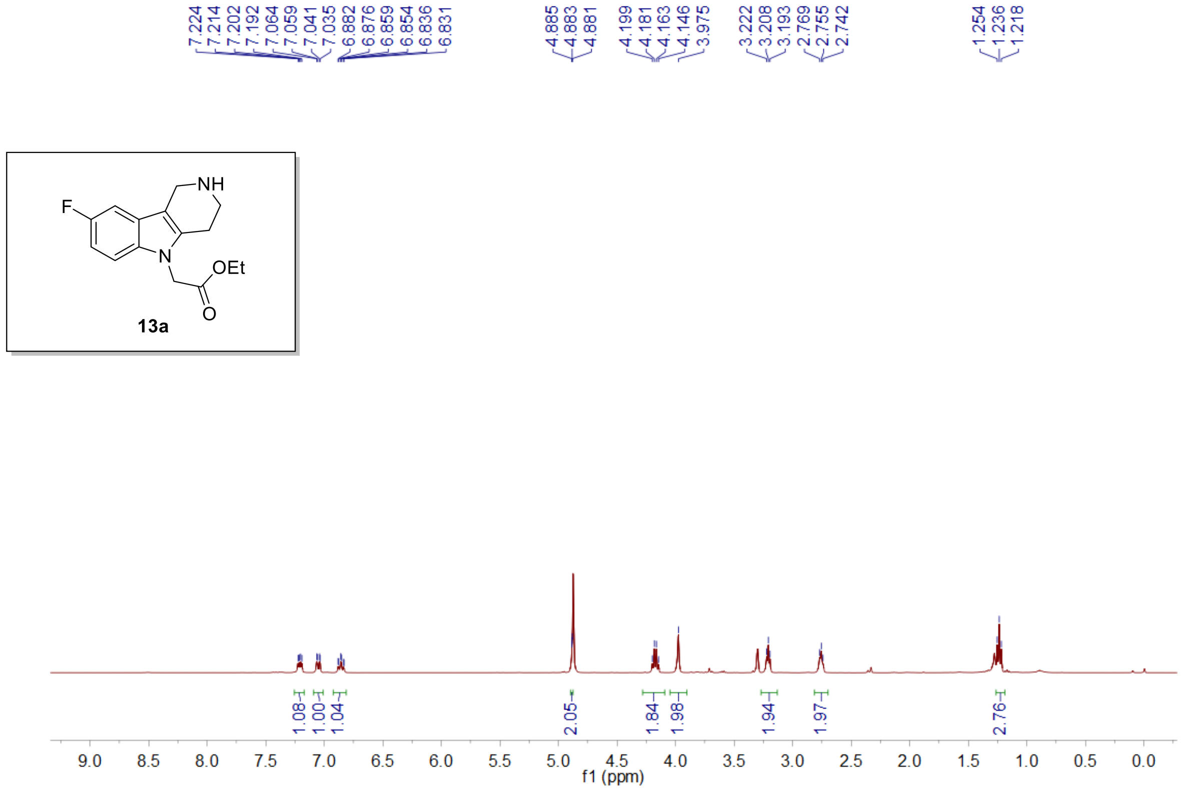

^{13}C-NMR spectrum of compound **13a** (101 MHz, MeOD)

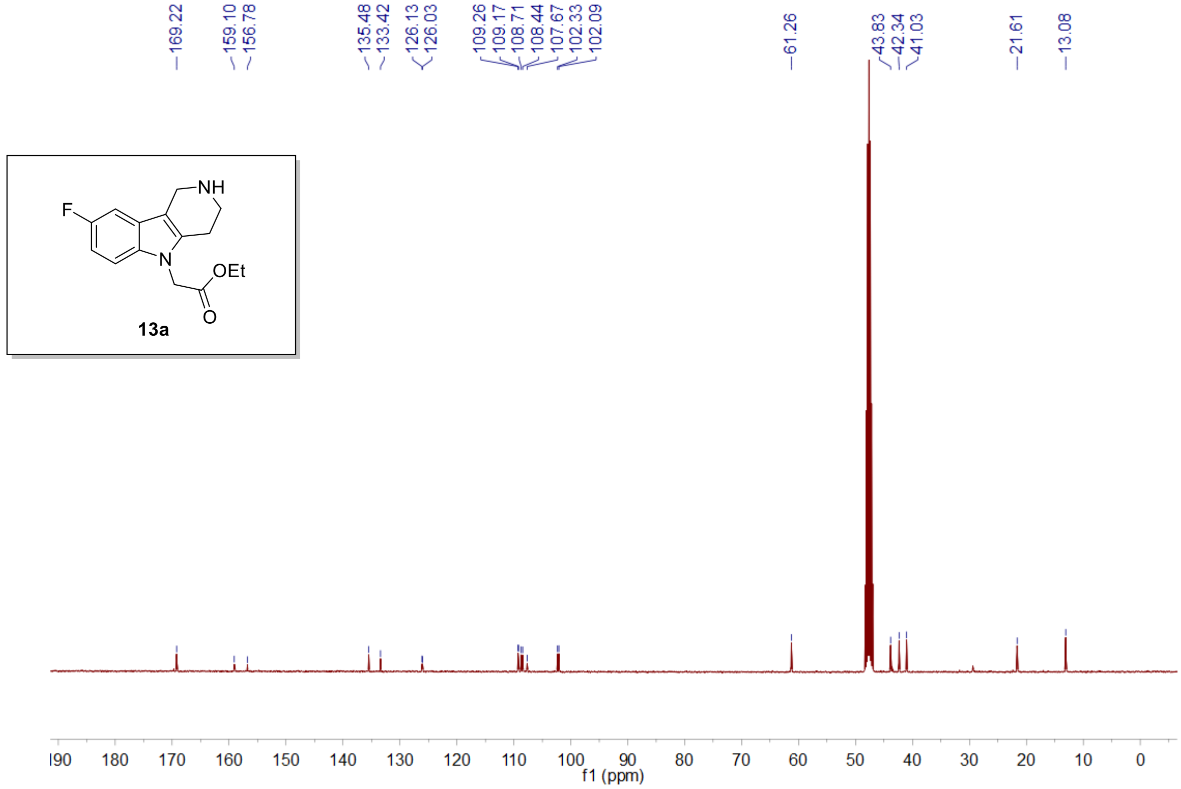

^{19}F-NMR spectrum of compound **13a** (565 MHz, MeOD)

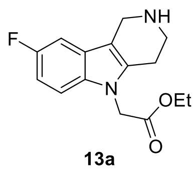

13a

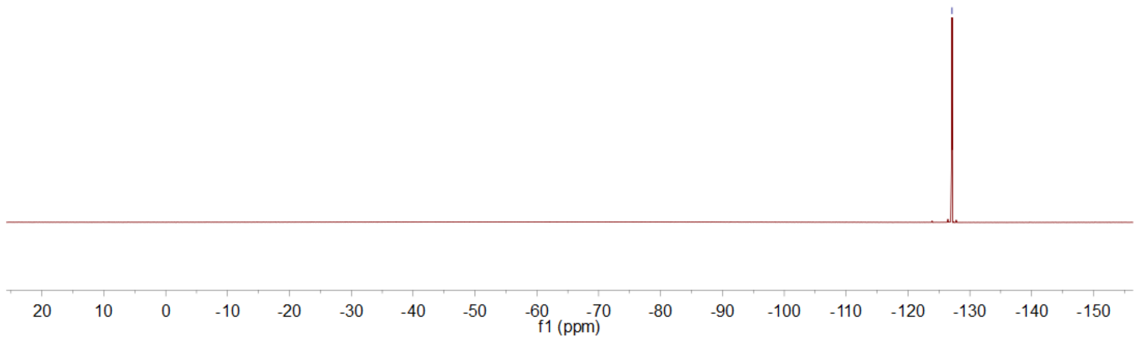

^{1}H-NMR spectrum of compound **13** (400 MHz, $CDCl_3$)

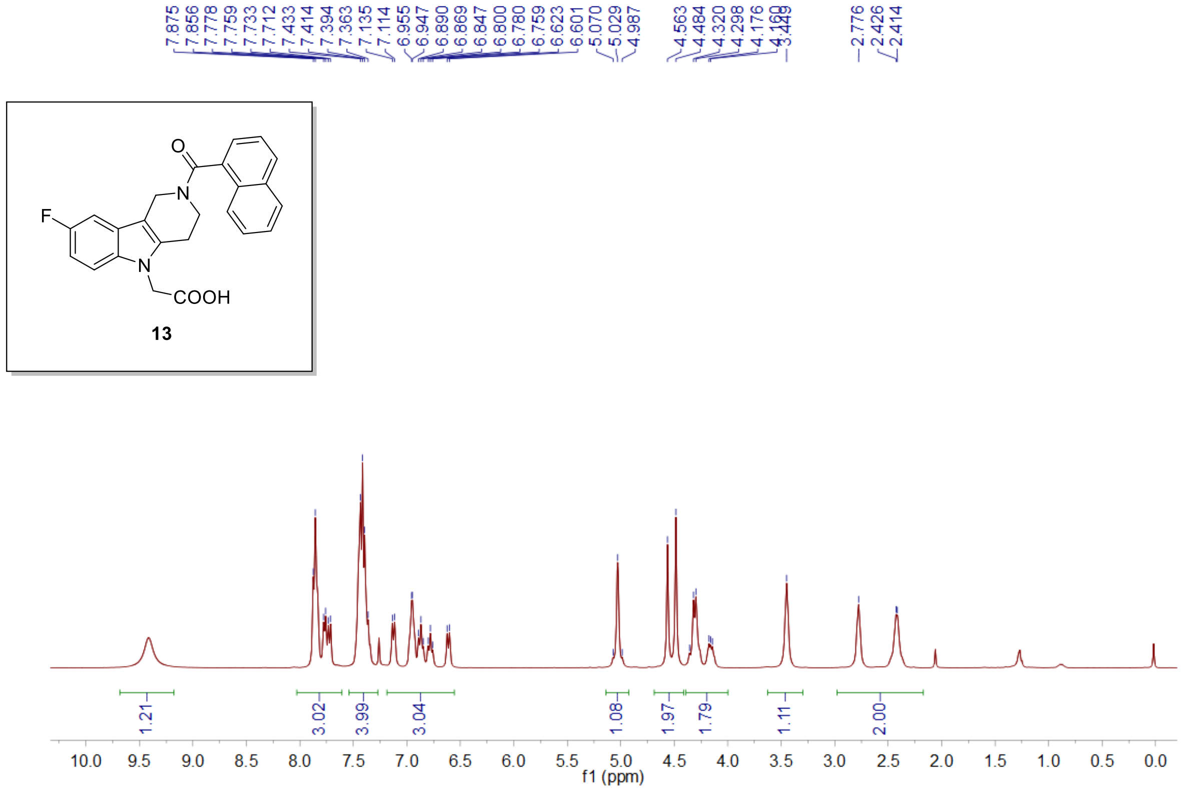

^{13}C-NMR spectrum of compound **13** (101 MHz, $CDCl_3$)

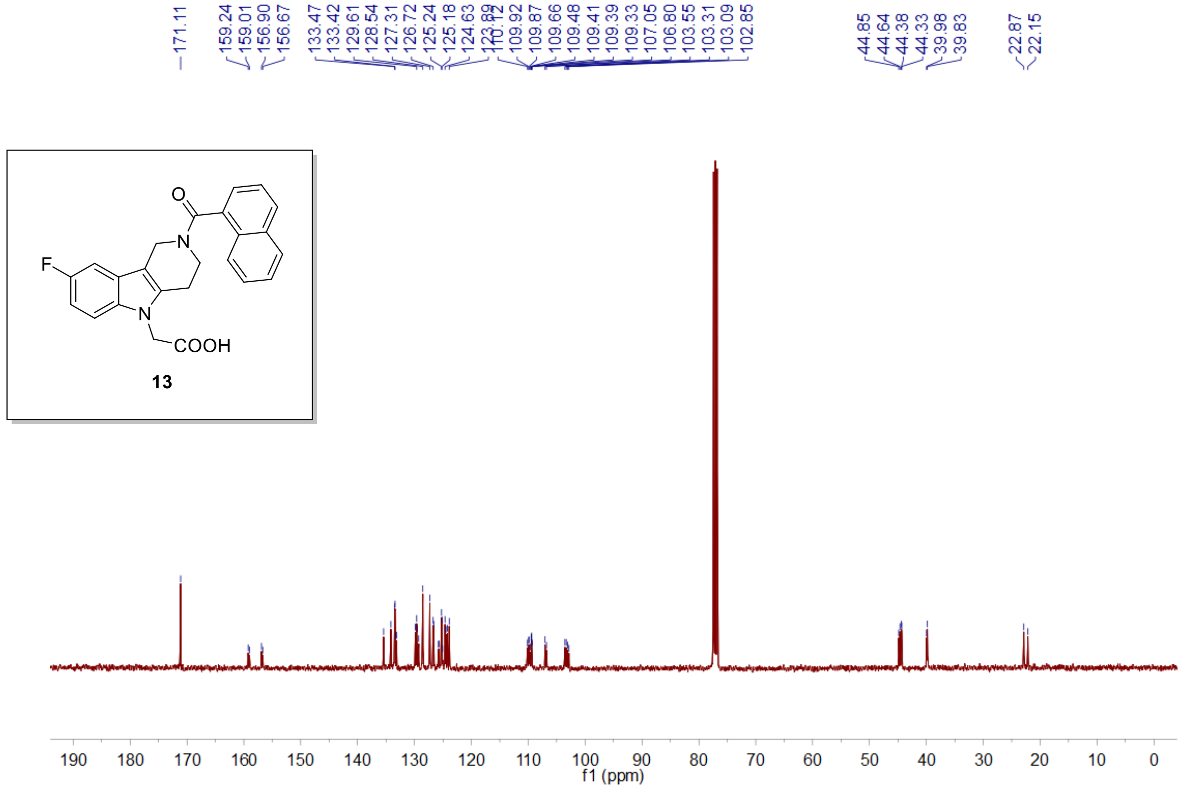

^{19}F-NMR spectrum of compound **13** (565 MHz, $CDCl_3$)

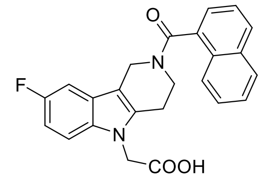

13

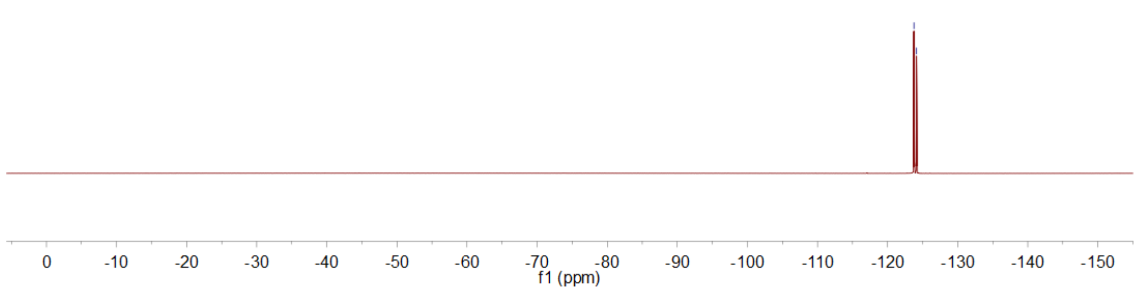

^{1}H-NMR spectrum of compound **14** (400 MHz, $CDCl_3$)

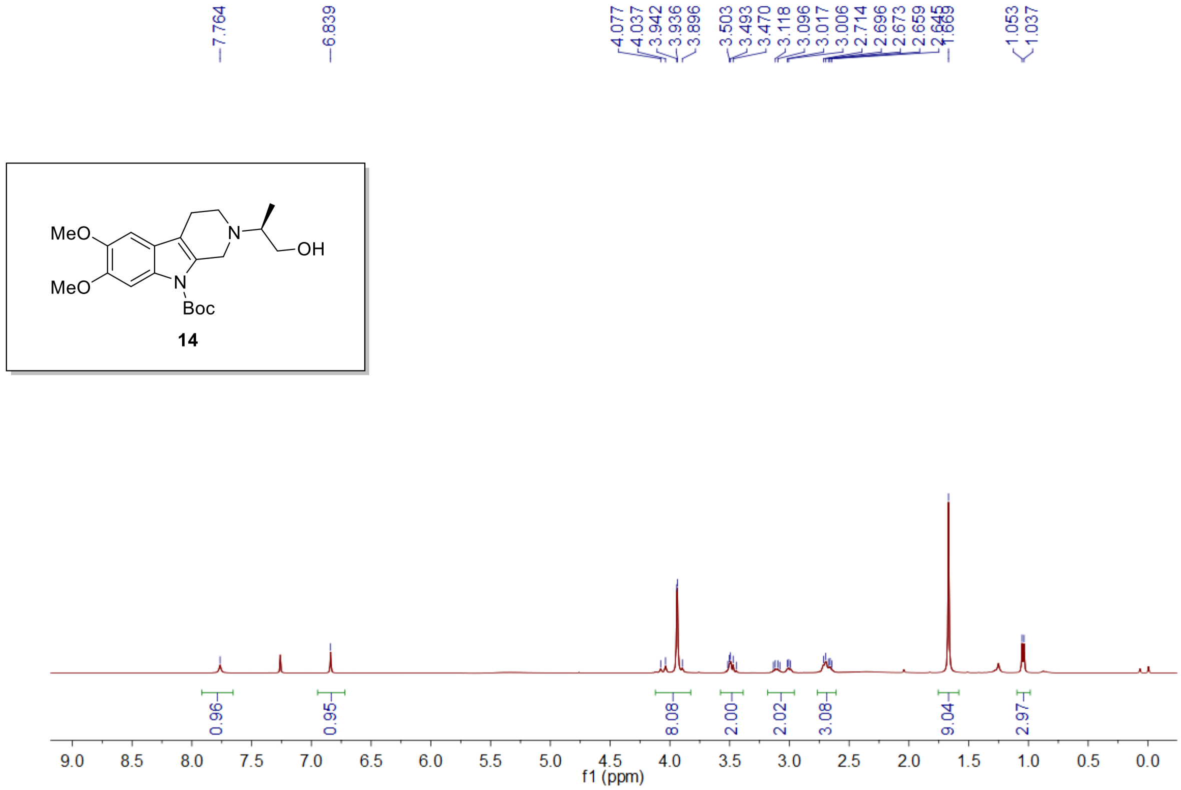

^{13}C-NMR spectrum of compound **14** (101 MHz, $CDCl_3$)

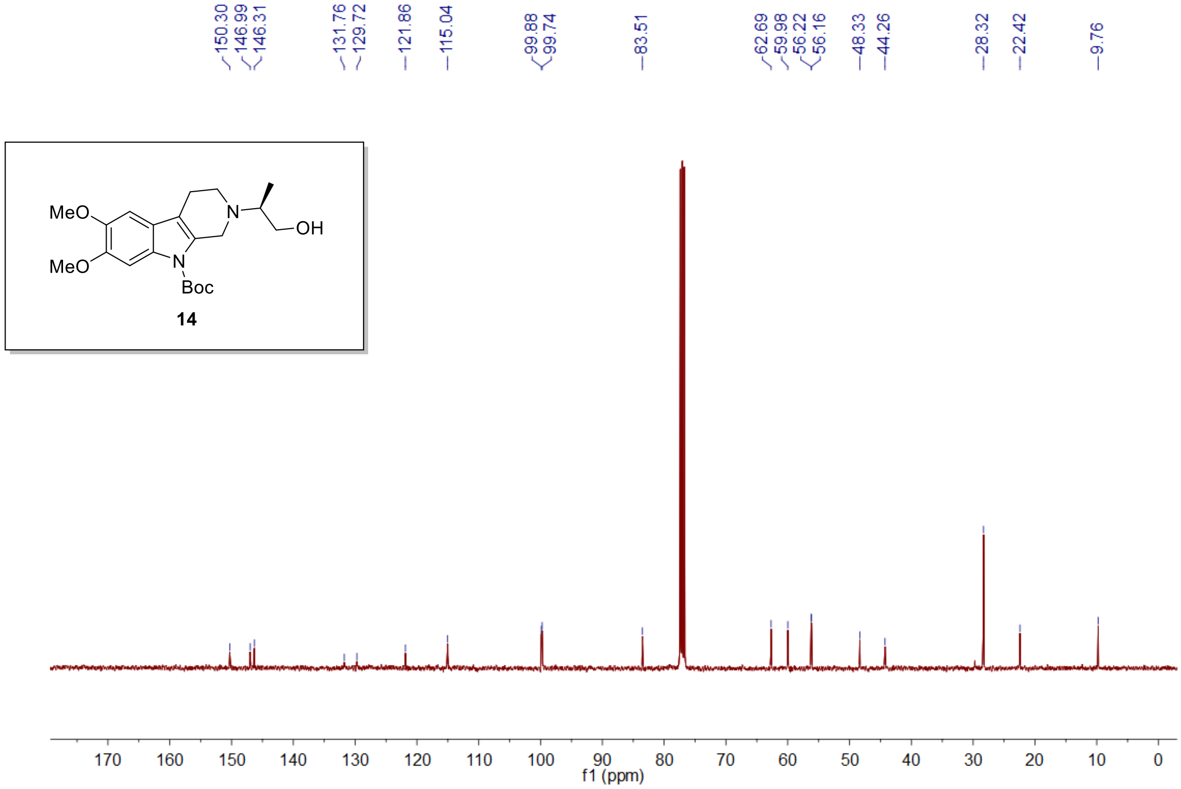

^{1}H-NMR spectrum of compound **15** (400 MHz, $CDCl_3$)

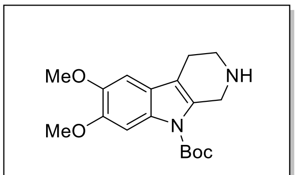

15

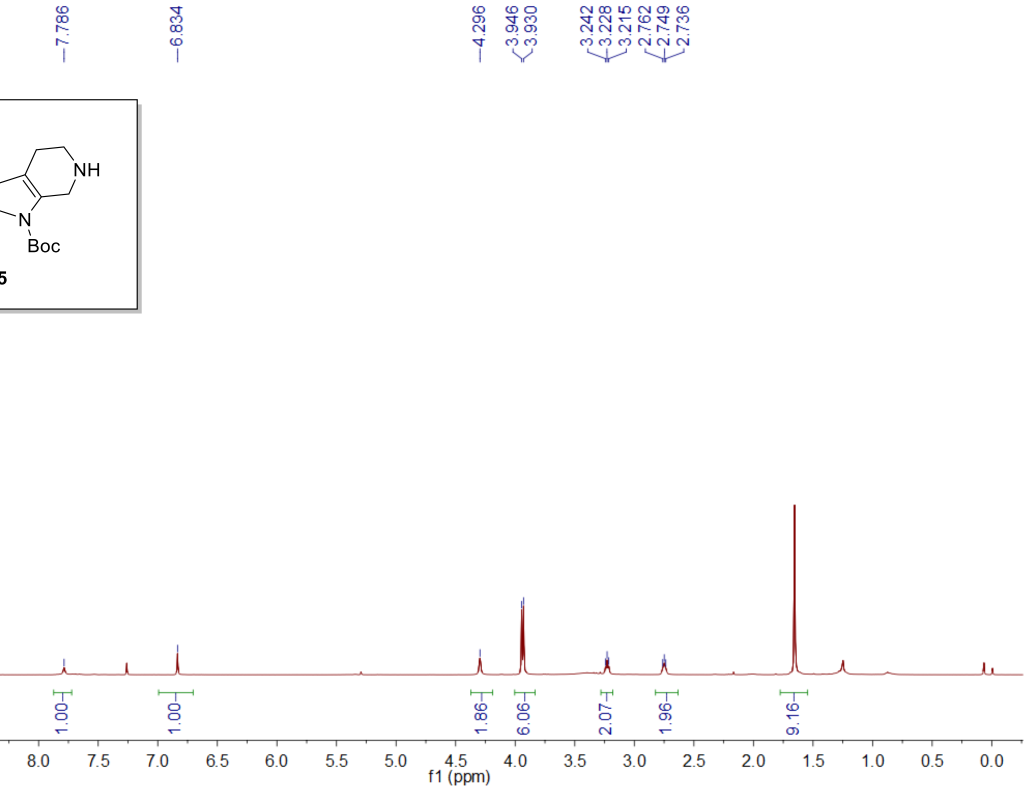

^{13}C-NMR spectrum of compound **15** (101 MHz, $CDCl_3$)

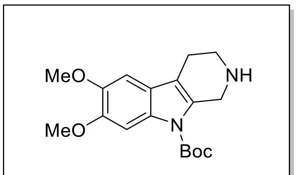

15

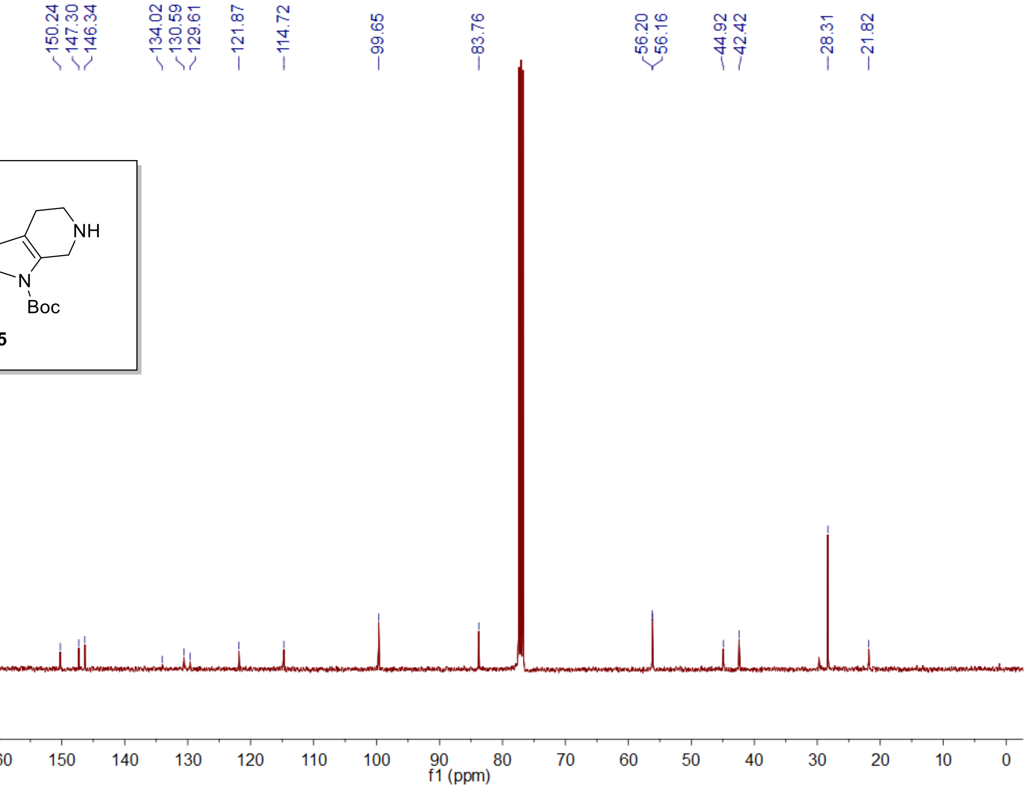

^{1}H-NMR spectrum of compound **16** (400 MHz, $CDCl_3$)

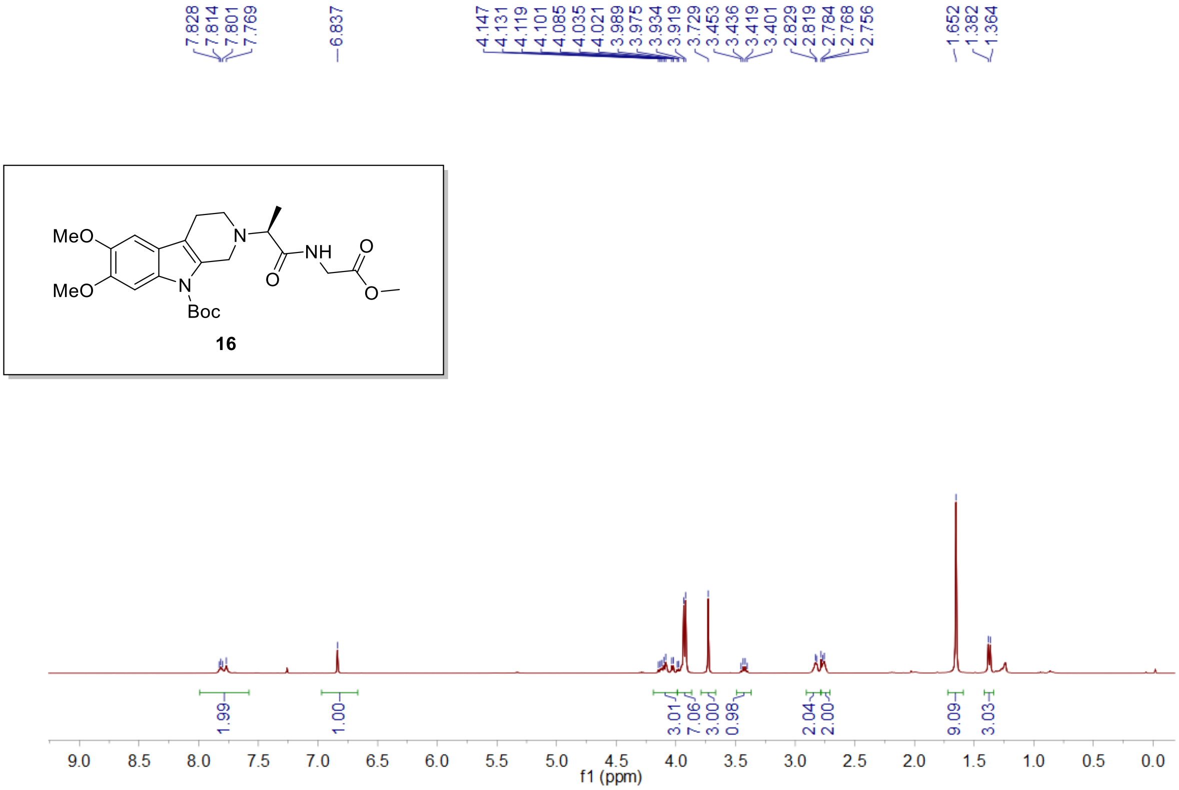

^{13}C-NMR spectrum of compound **16** (101 MHz, $CDCl_3$)

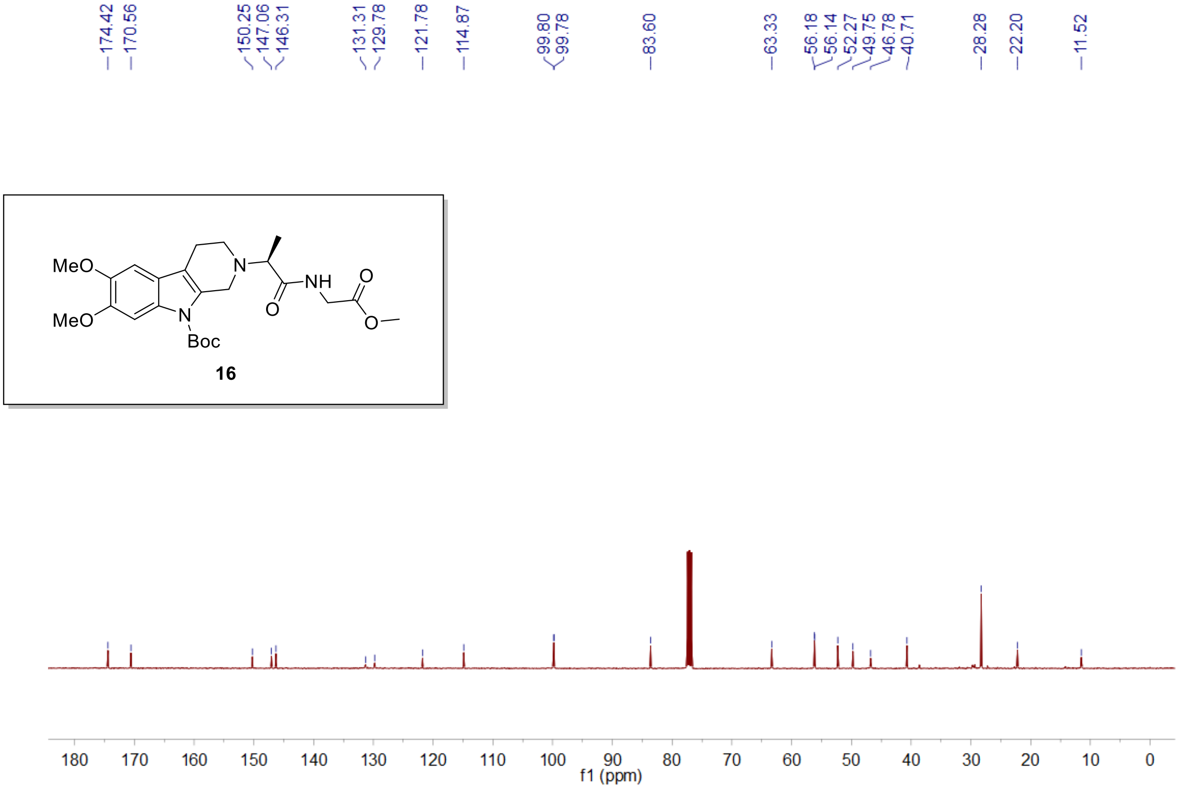

^{1}H-NMR spectrum of compound **17** (400 MHz, $CDCl_3$)

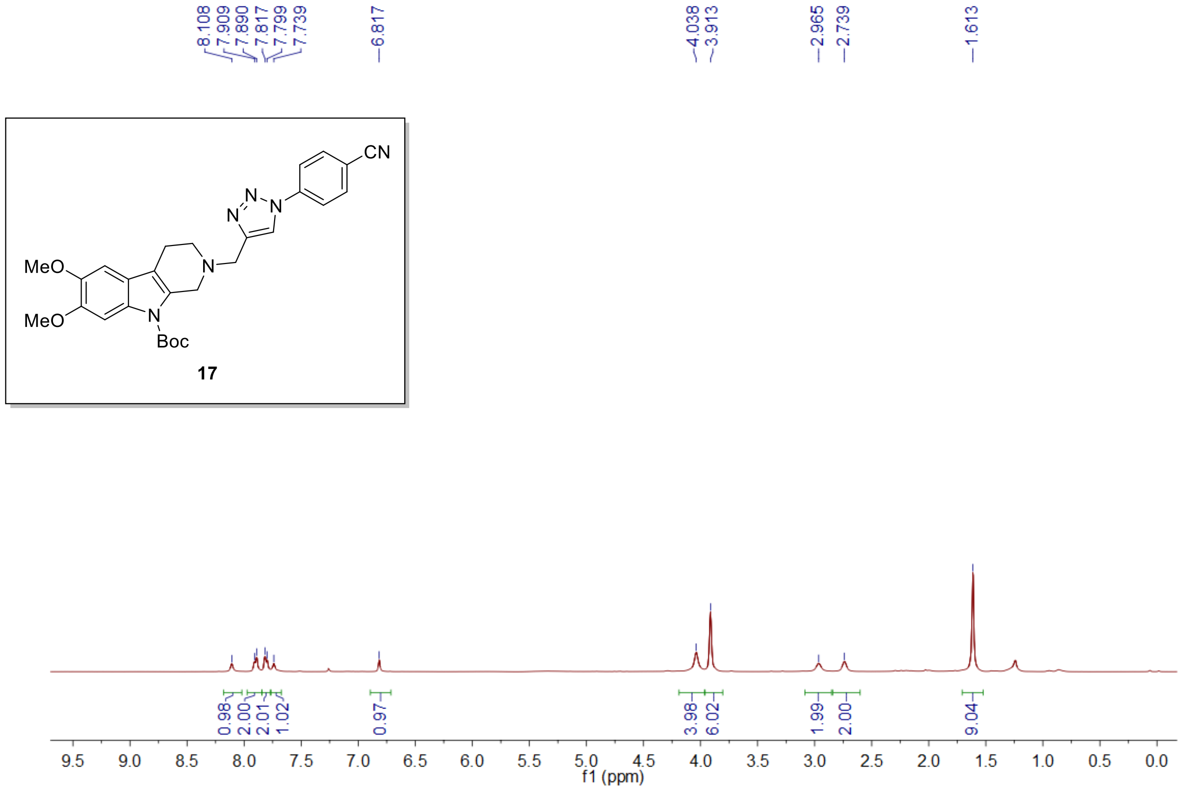

^{13}C-NMR spectrum of compound **17** (101 MHz, $CDCl_3$)

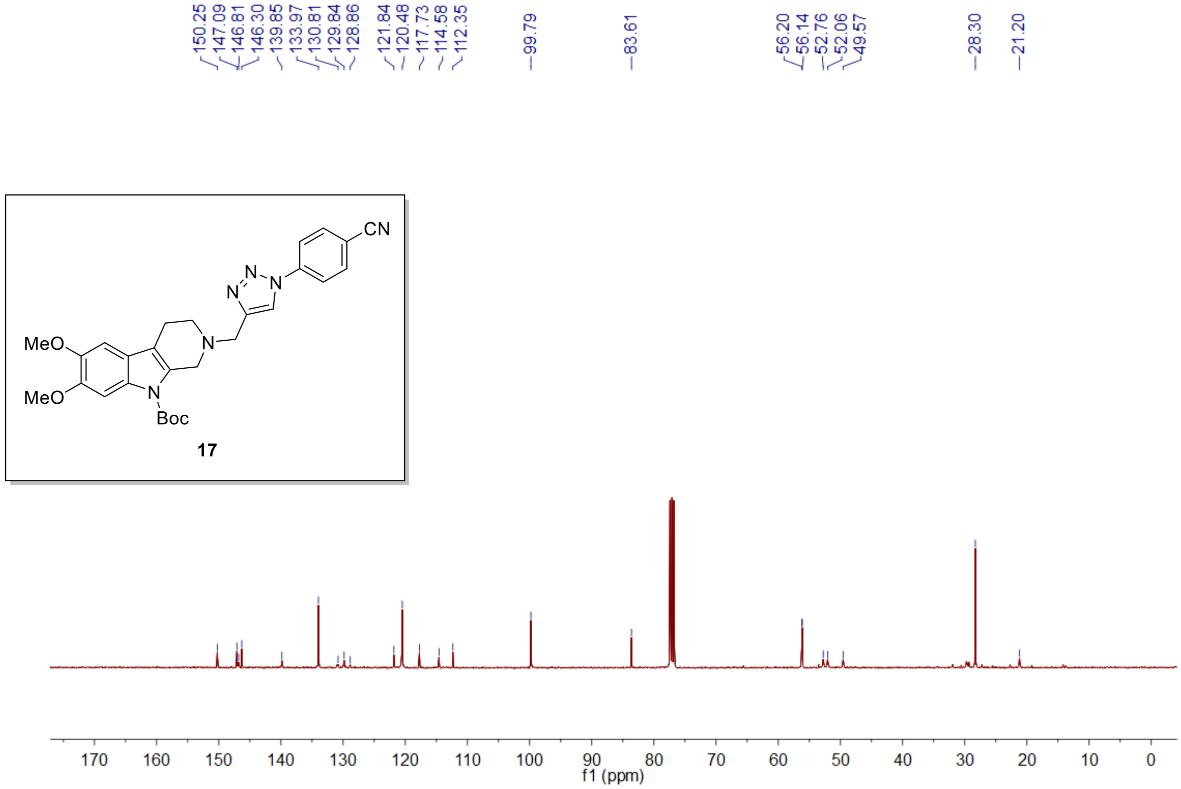

^{1}H-NMR spectrum of compound **18** (500 MHz, $CDCl_3$)

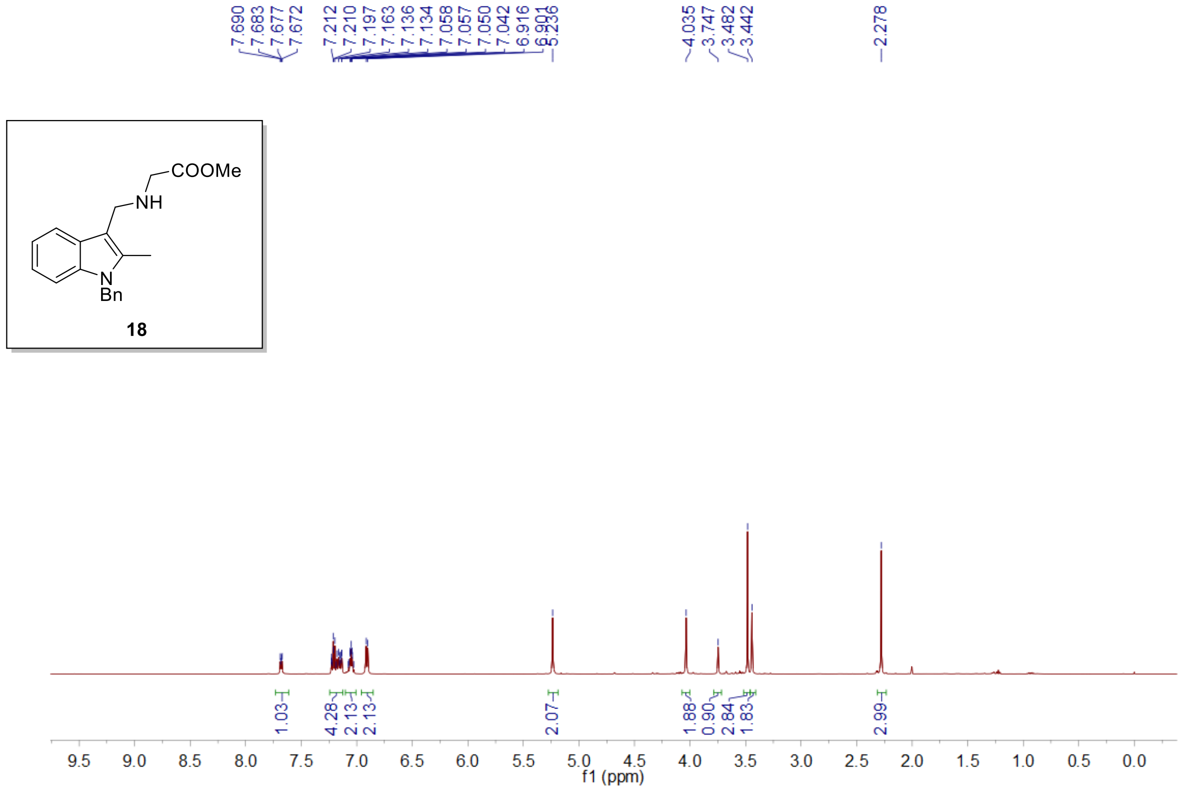

^{13}C-NMR spectrum of compound **18** (126 MHz, $CDCl_3$)

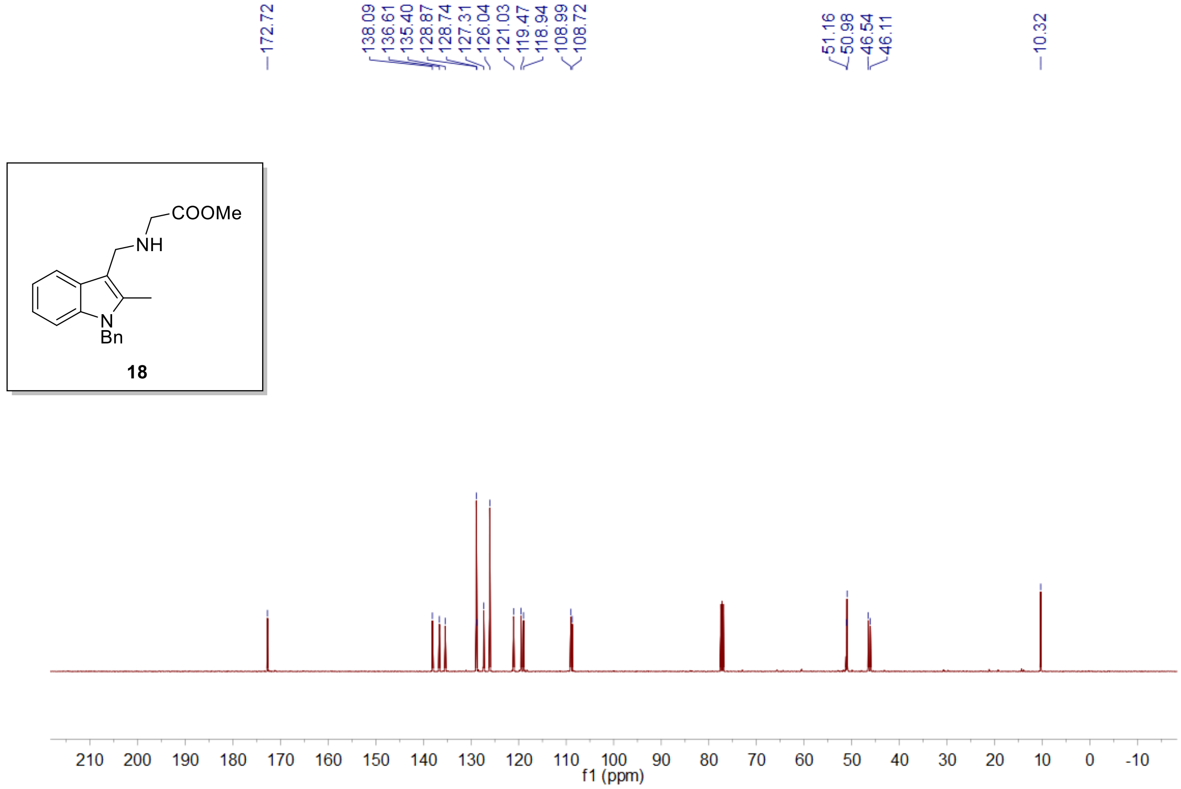

^{1}H-NMR spectrum of compound **19** (400 MHz, $CDCl_3$)

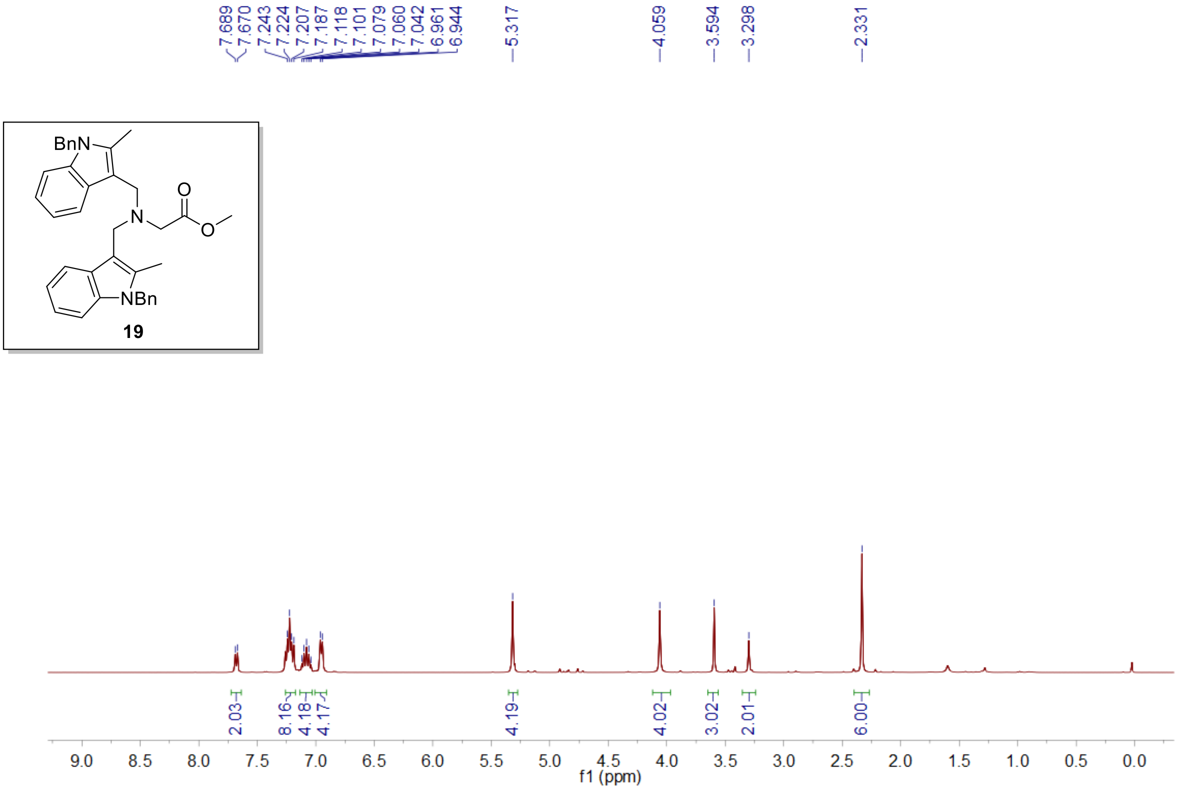

^{13}C-NMR spectrum of compound **19** (101 MHz, $CDCl_3$)

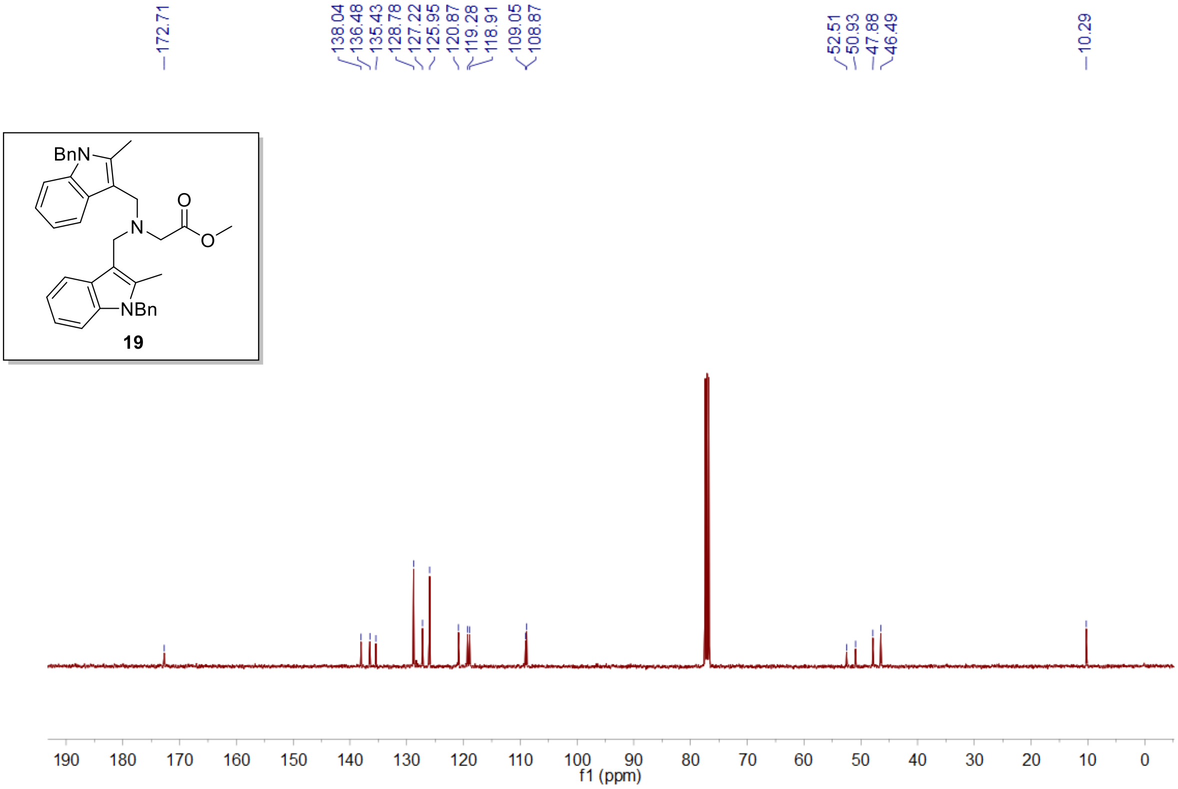

^{1}H-NMR spectrum of compound **19-f** (400 MHz, $CDCl_3$)

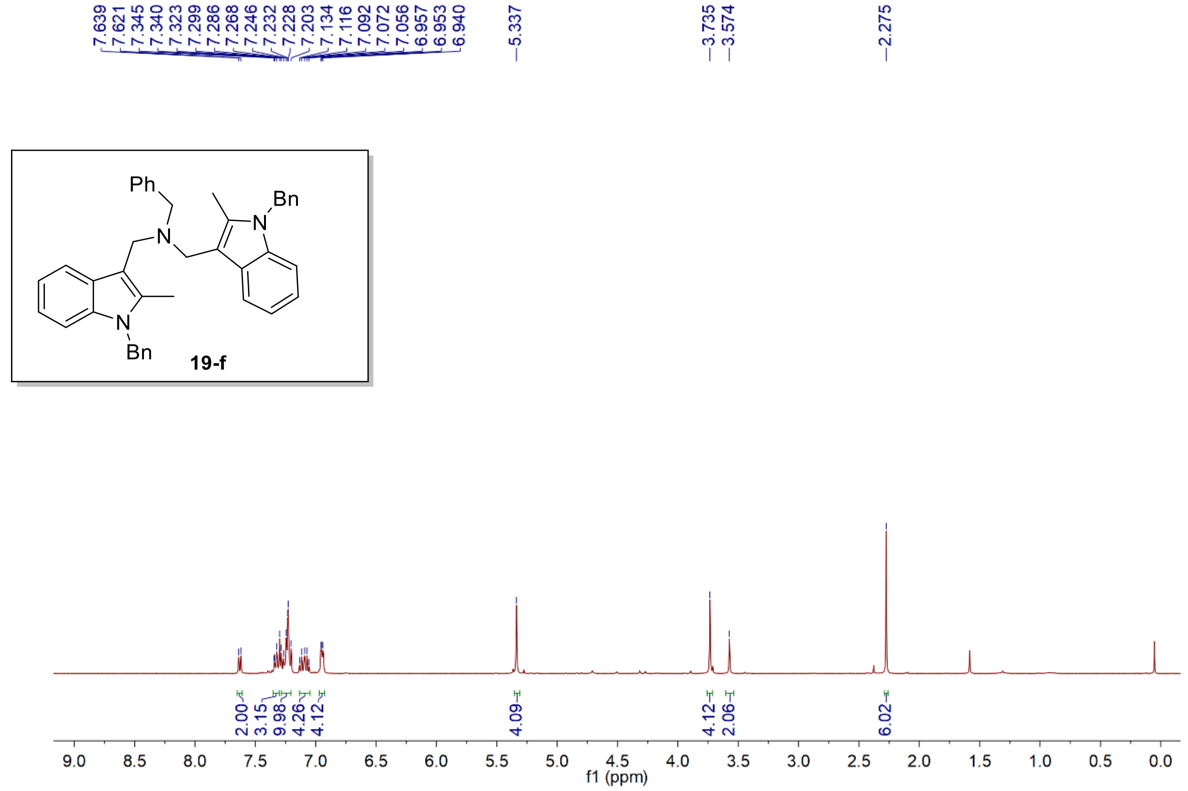

^{13}C-NMR spectrum of compound **19-f** (101 MHz, $CDCl_3$)

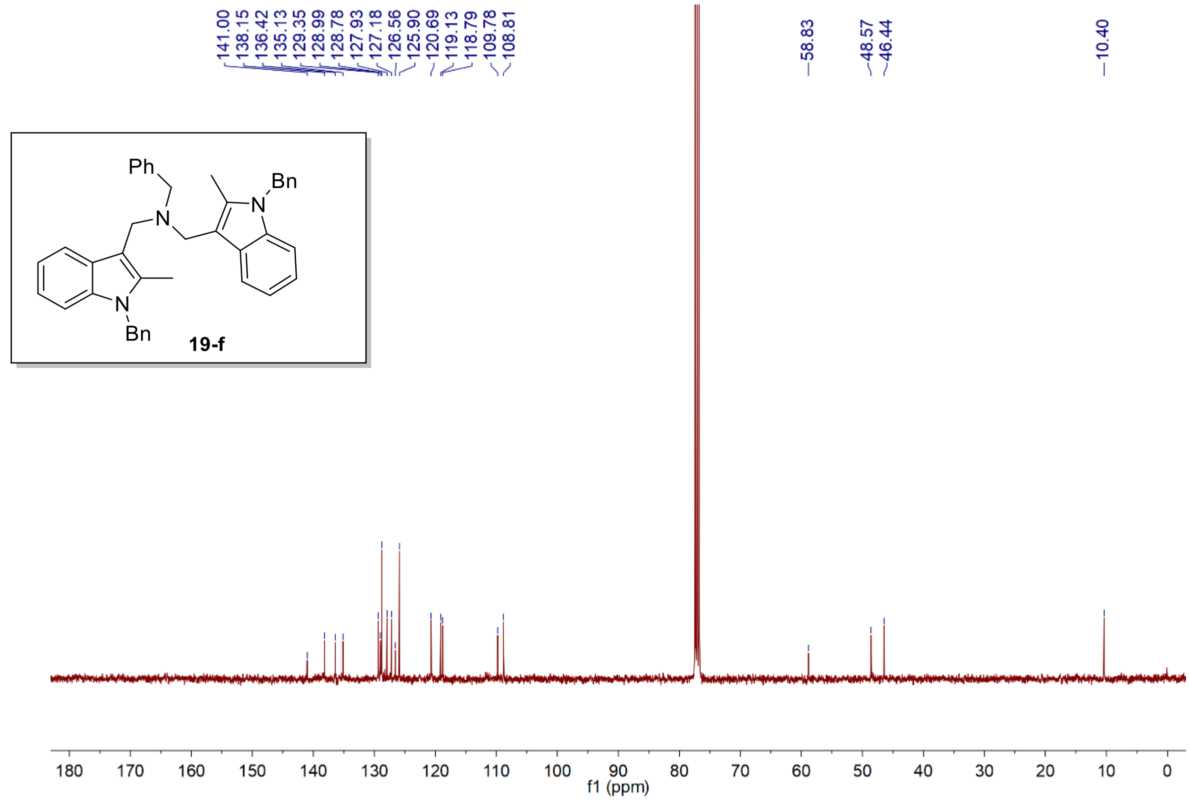

^{1}H-NMR spectrum of compound **20** (400 MHz, $CDCl_3$)

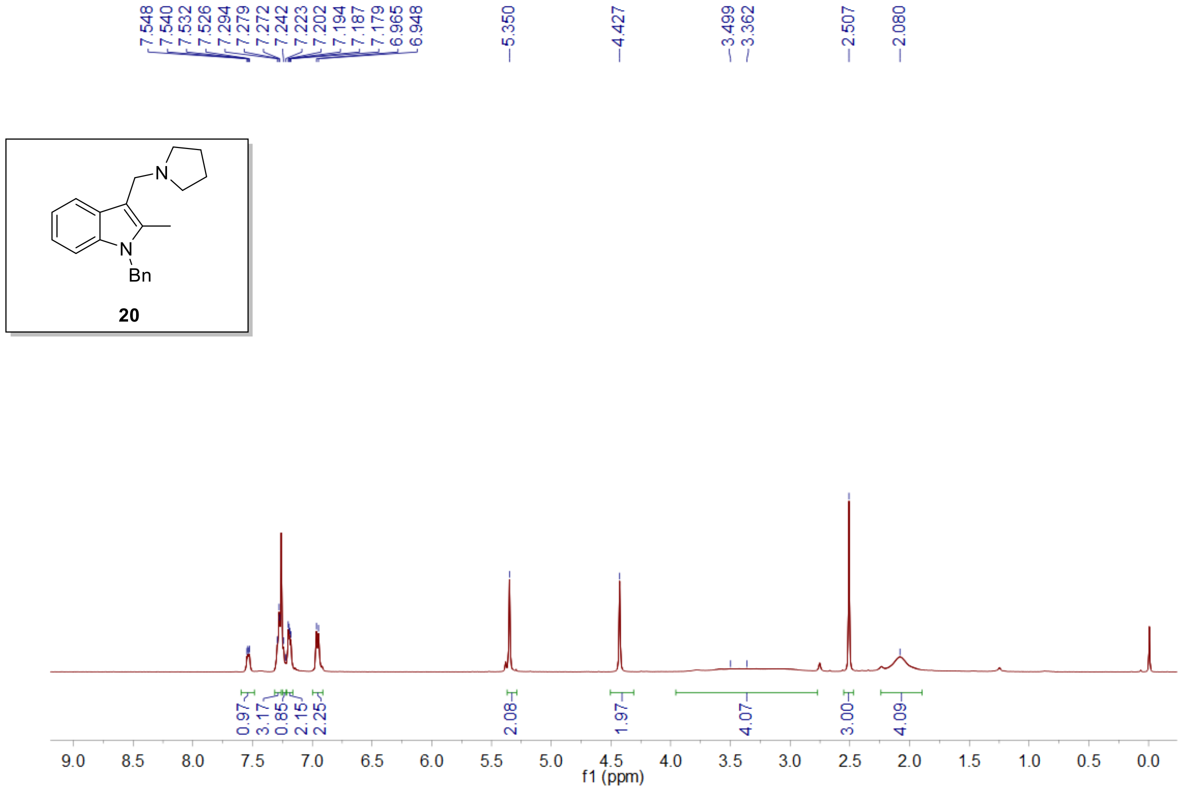

^{13}C-NMR spectrum of compound **20** (101 MHz, $CDCl_3$)

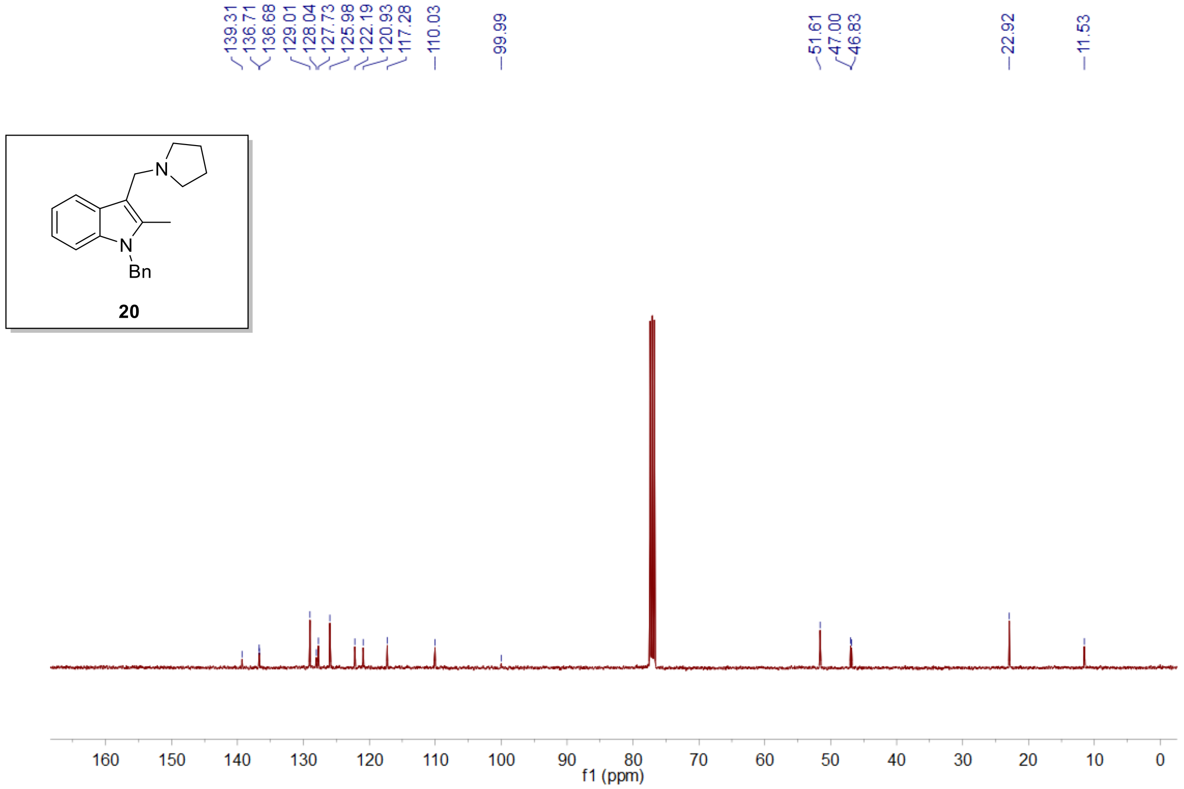

^{1}H-NMR spectrum of compound [D$_4$]-**4a** (400 MHz, $CDCl_3$)

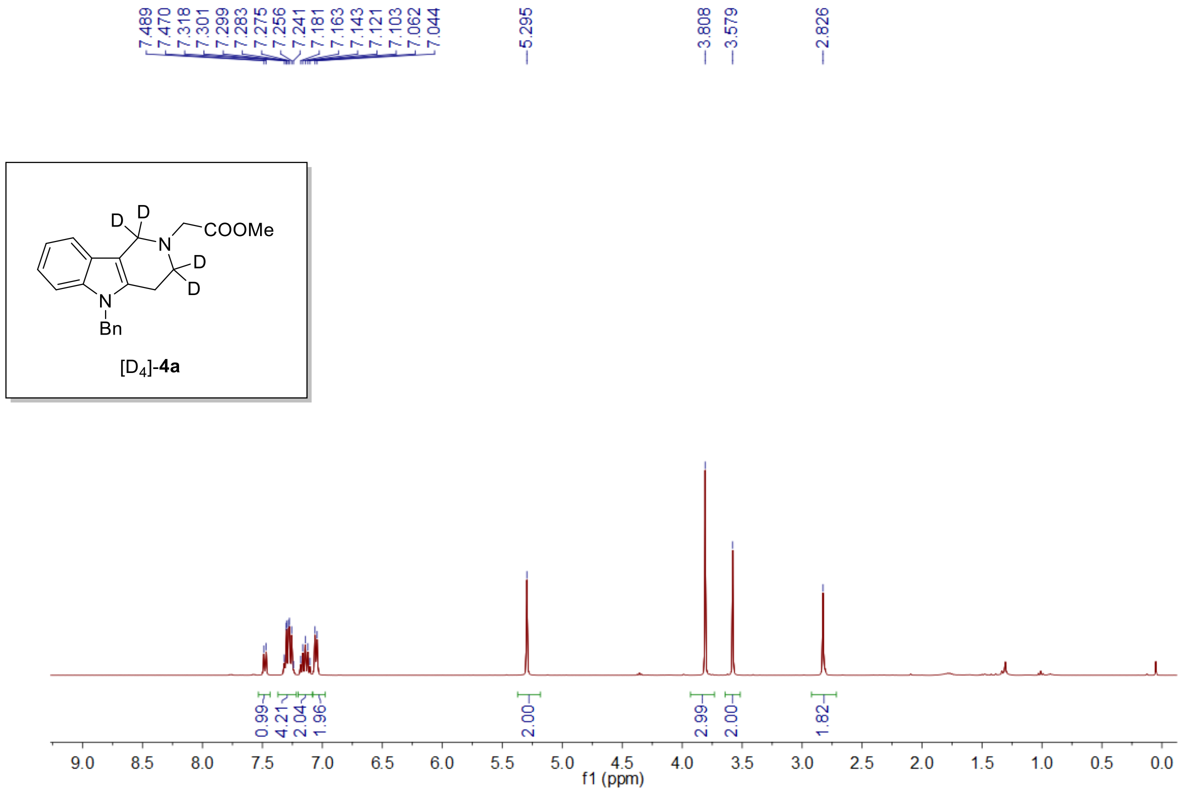

^{13}C-NMR spectrum of compound [D$_4$]-**4a** (101 MHz, $CDCl_3$)

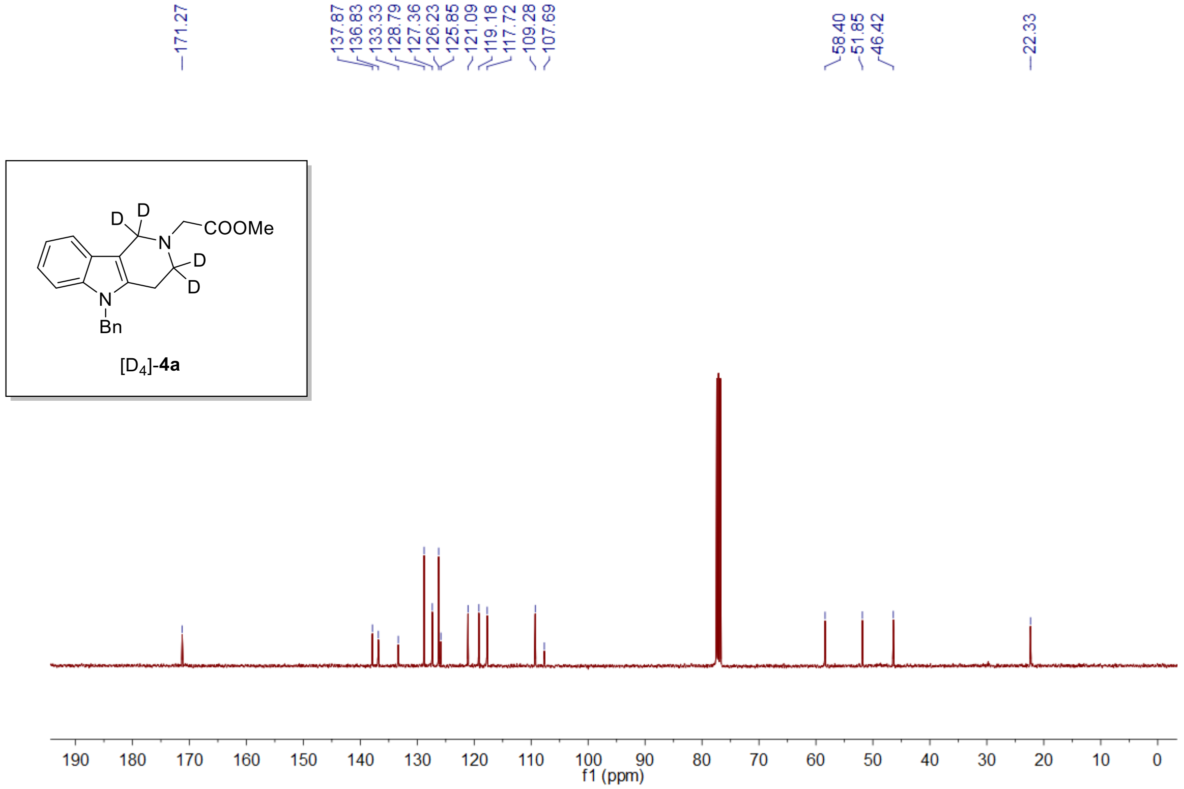

^{1}H-NMR spectrum of compound [D$_4$]-**4a**-Entry2 (400 MHz, $CDCl_3$)

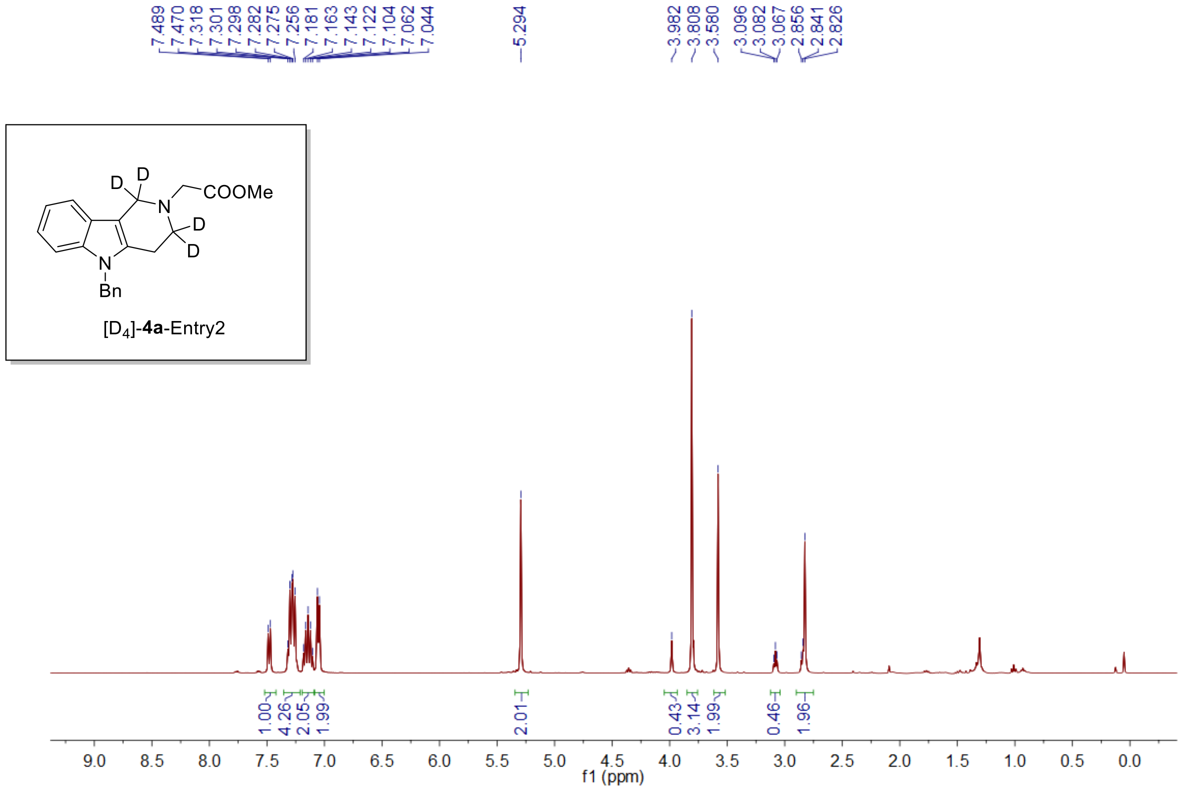

^{13}C-NMR spectrum of compound [D$_4$]-**4a**-Entry2 (101 MHz, $CDCl_3$)

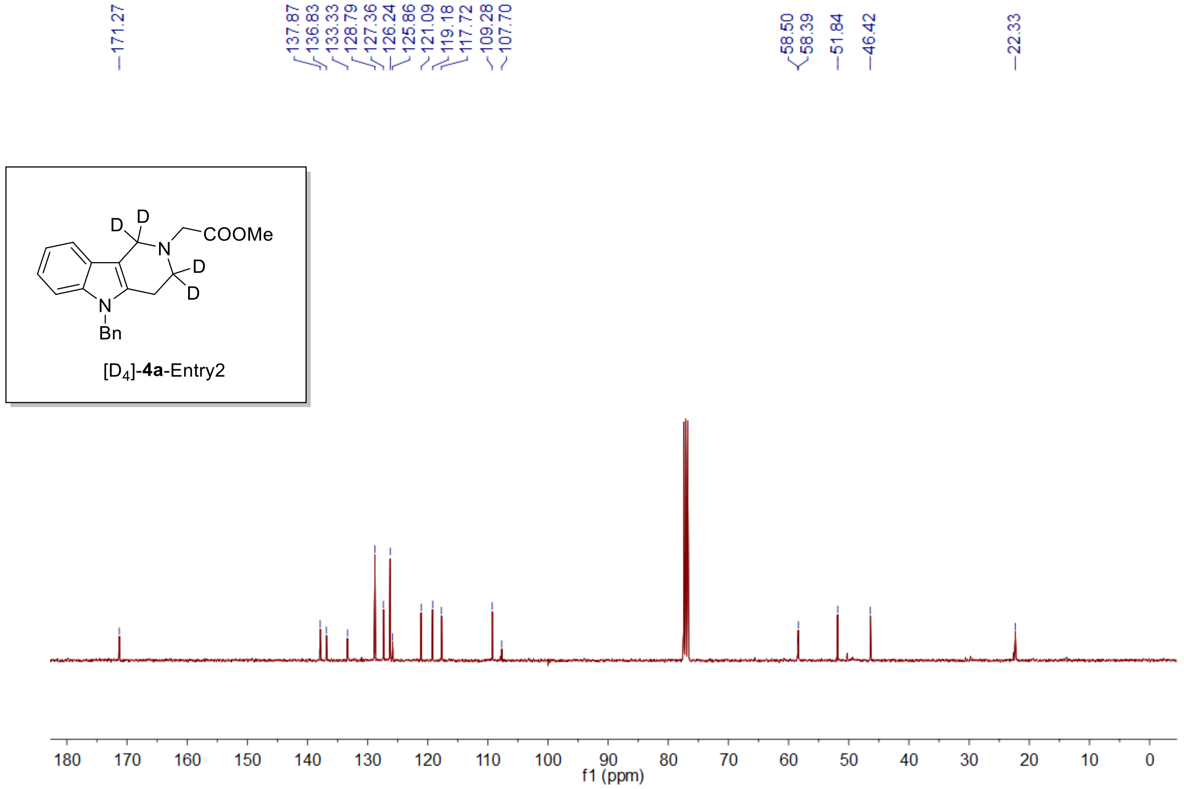

^{1}H-NMR spectrum of compound [D_4]-**4a**-Entry3 (400 MHz, $CDCl_3$)

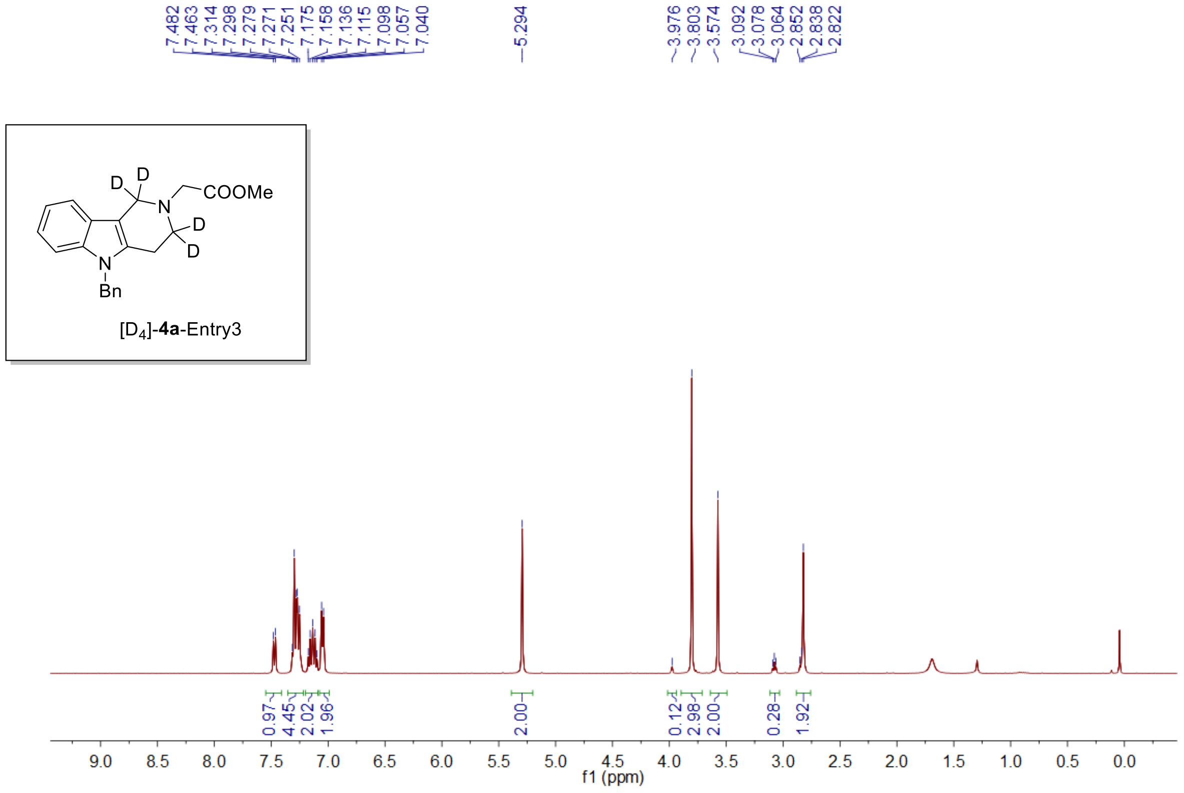

^{13}C-NMR spectrum of compound [D_4]-**4a**-Entry3 (101 MHz, $CDCl_3$)

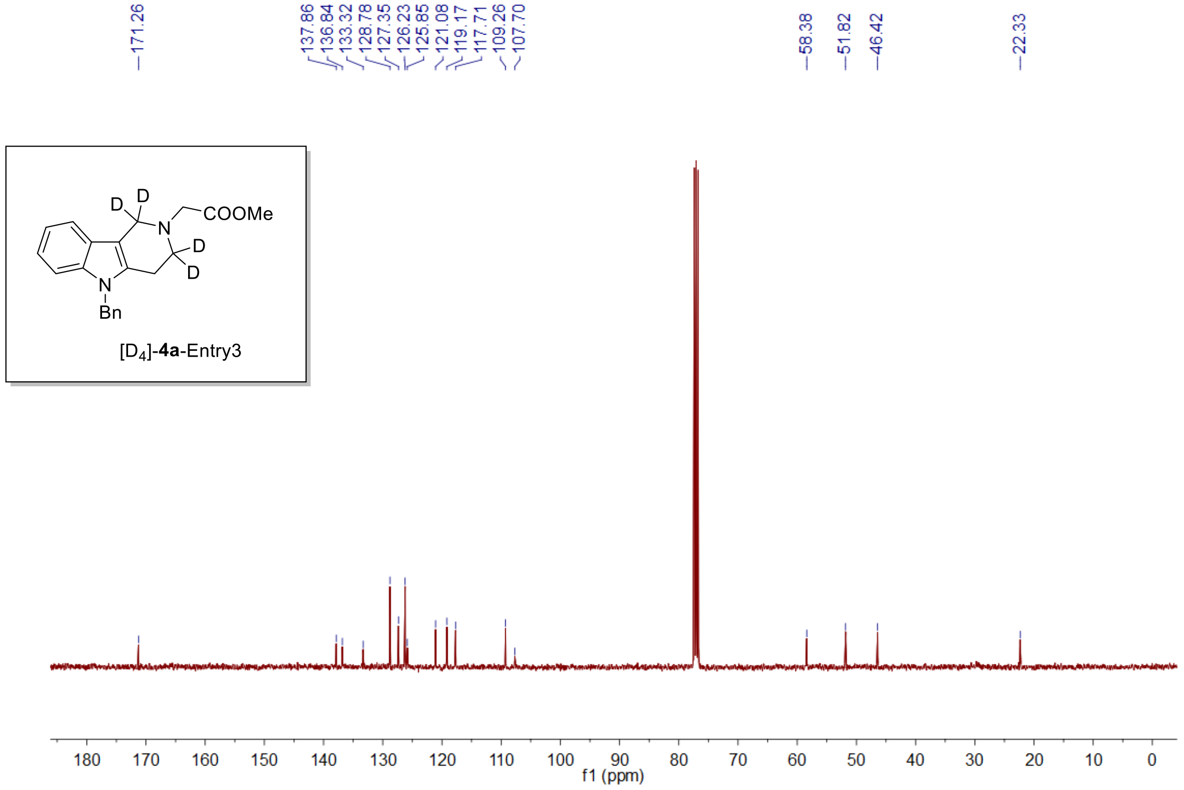

^{1}H-NMR spectrum of compound [D$_4$]-**4a**-Entry4 (400 MHz, $CDCl_3$)

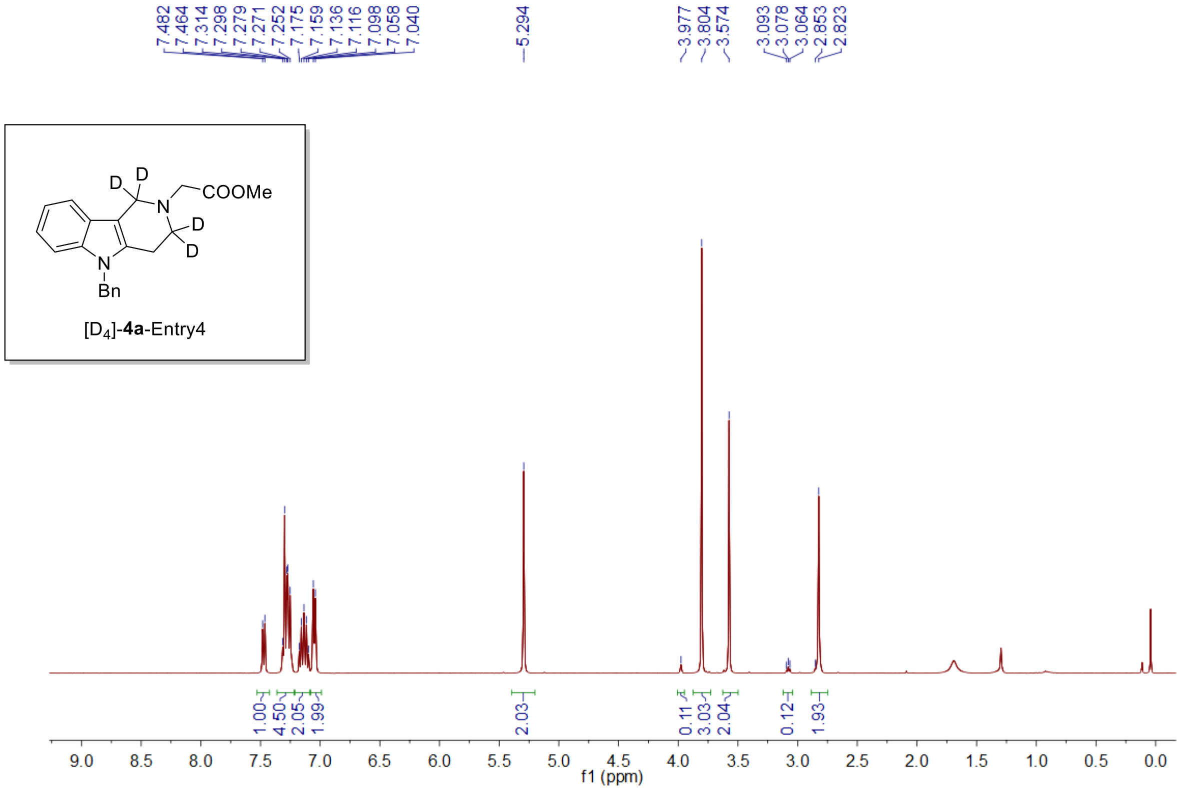

^{13}C-NMR spectrum of compound [D$_4$]-**4a**-Entry4 (101 MHz, $CDCl_3$)

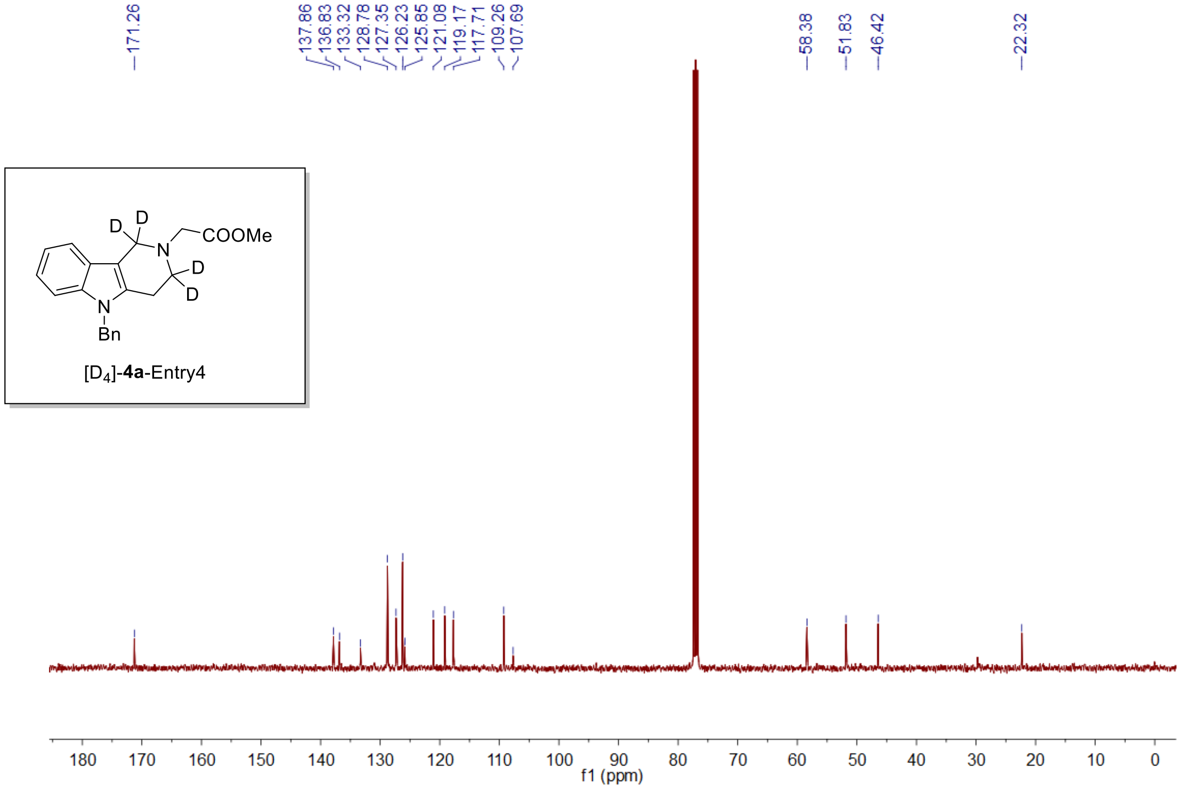

^{1}H-NMR spectrum of compound **21** (400 MHz, $CDCl_3$)

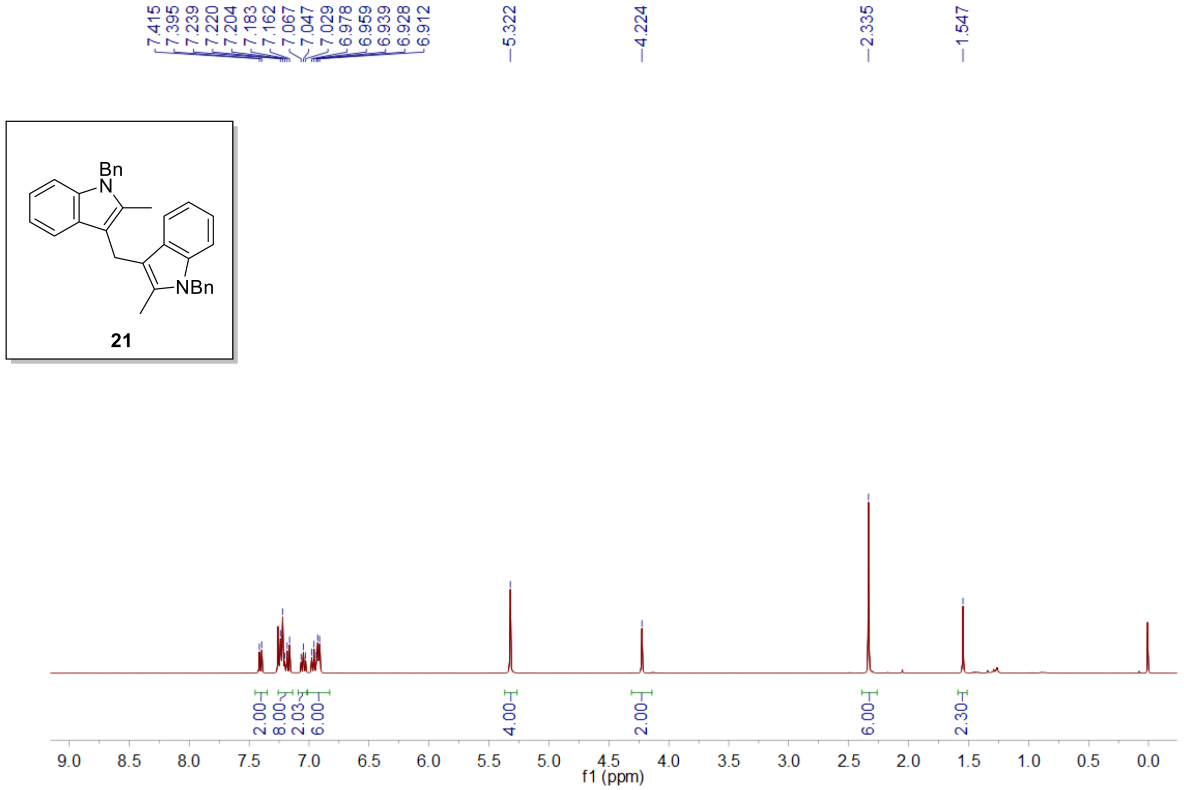

^{13}C-NMR spectrum of compound **21** (101 MHz, $CDCl_3$)

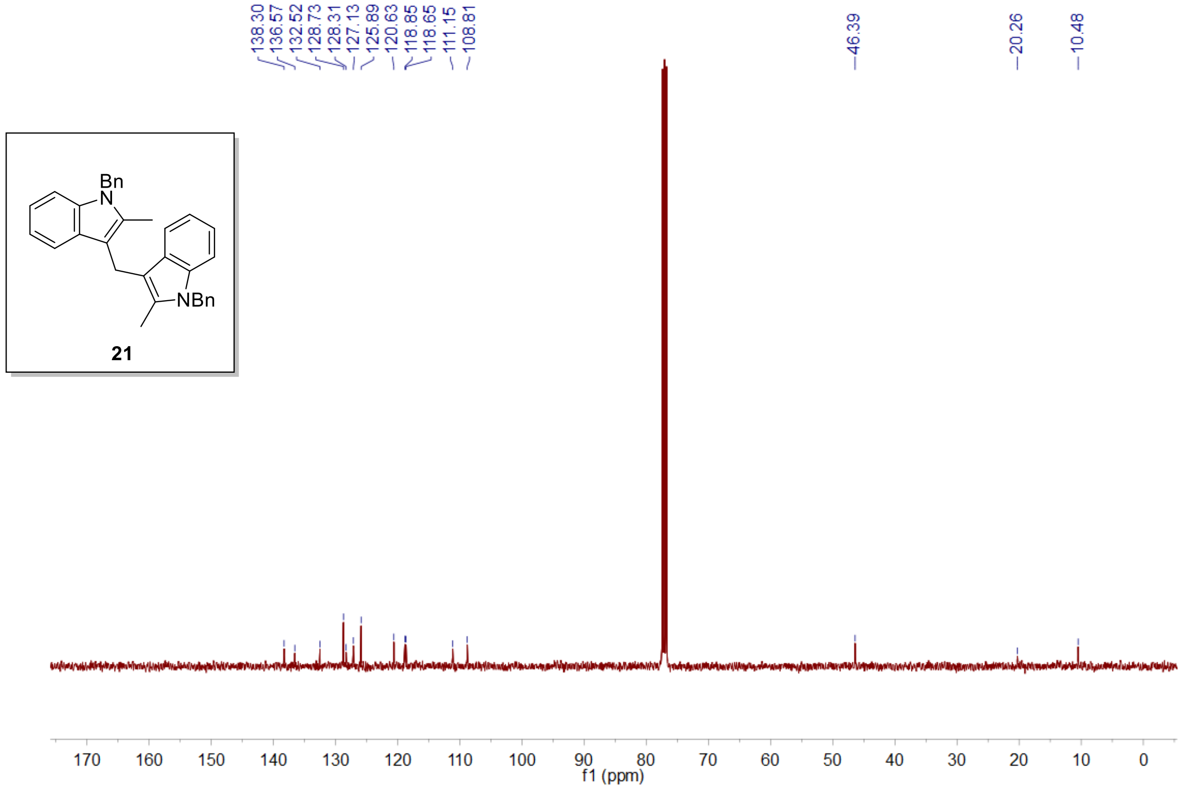

^{1}H-NMR spectrum of compound **22** (400 MHz, $CDCl_3$)

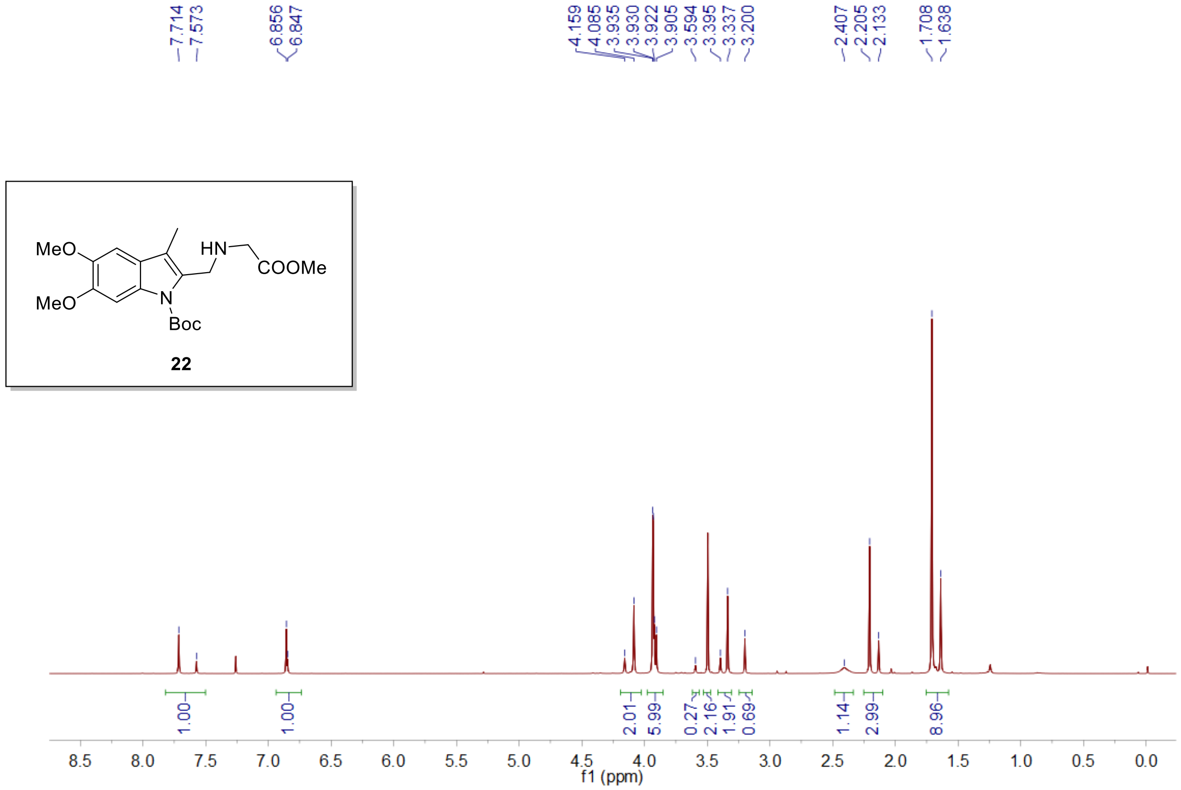

^{13}C-NMR spectrum of compound **22** (101 MHz, $CDCl_3$)

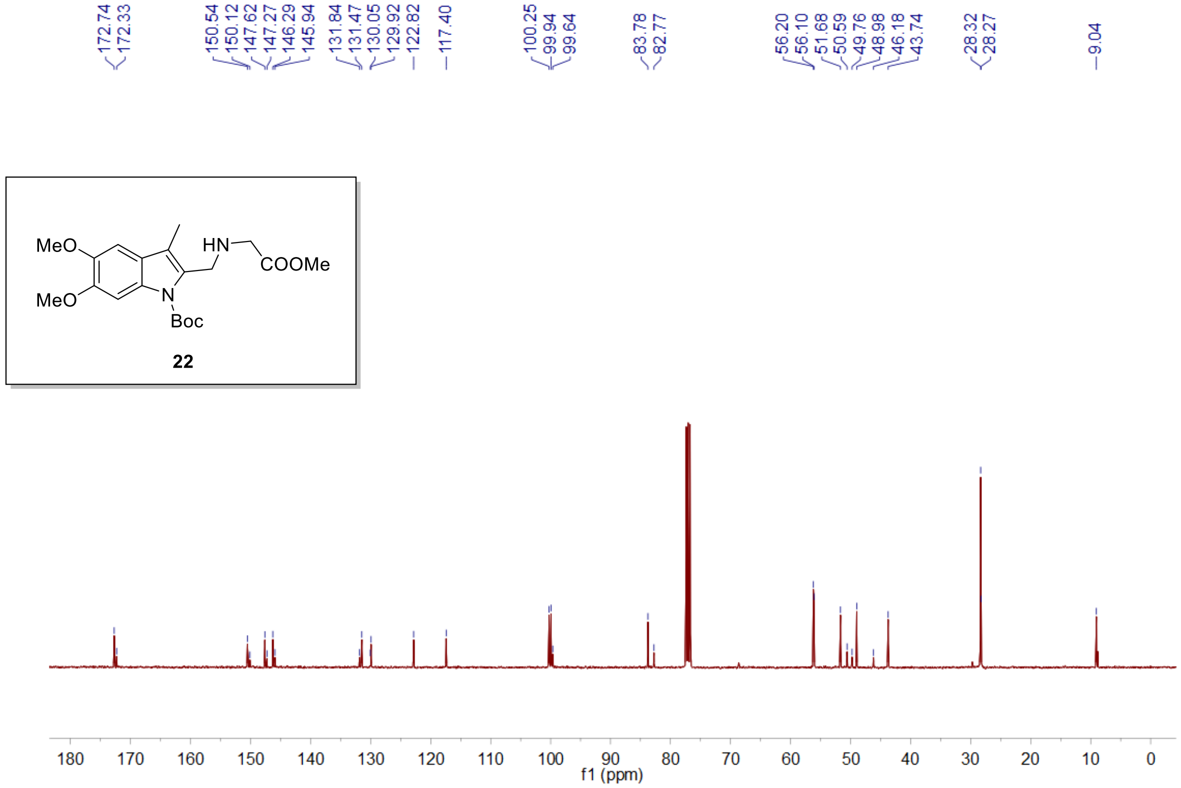

^{1}H-NMR spectrum of compound **23** (400 MHz, $CDCl_3$)

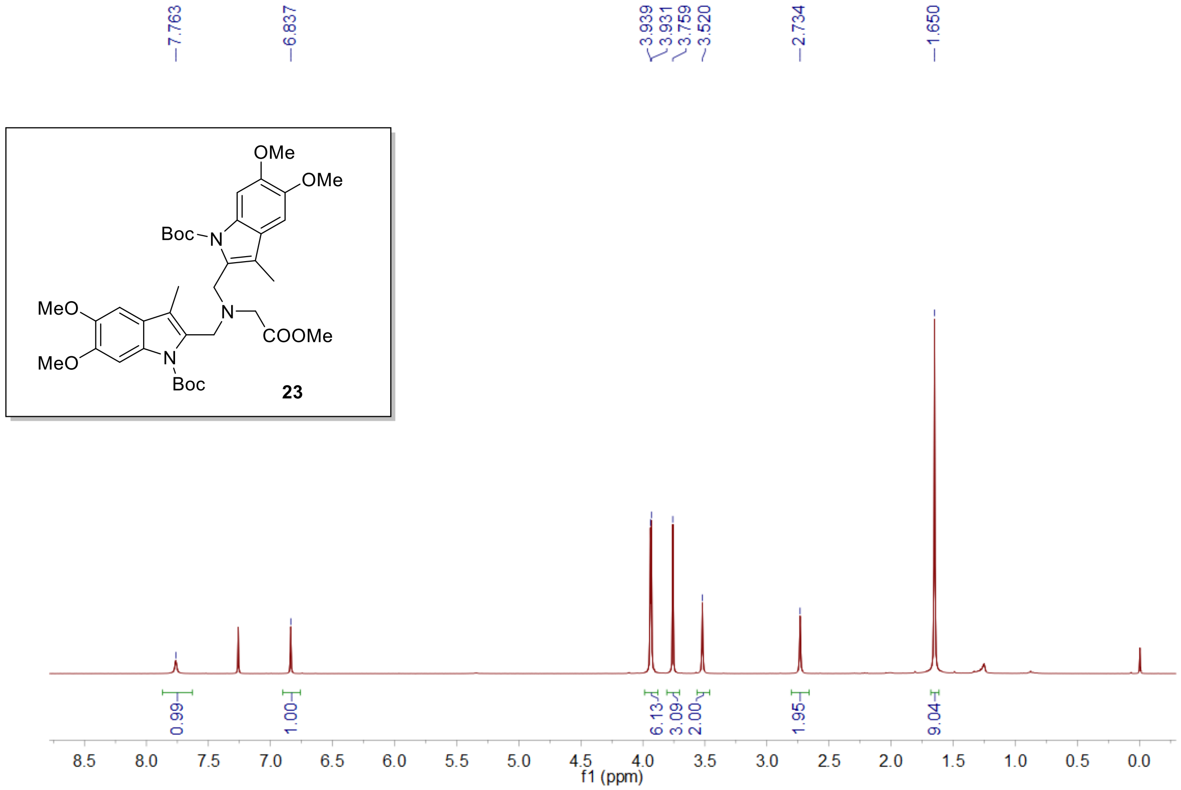

^{13}C-NMR spectrum of compound **23** (101 MHz, $CDCl_3$)

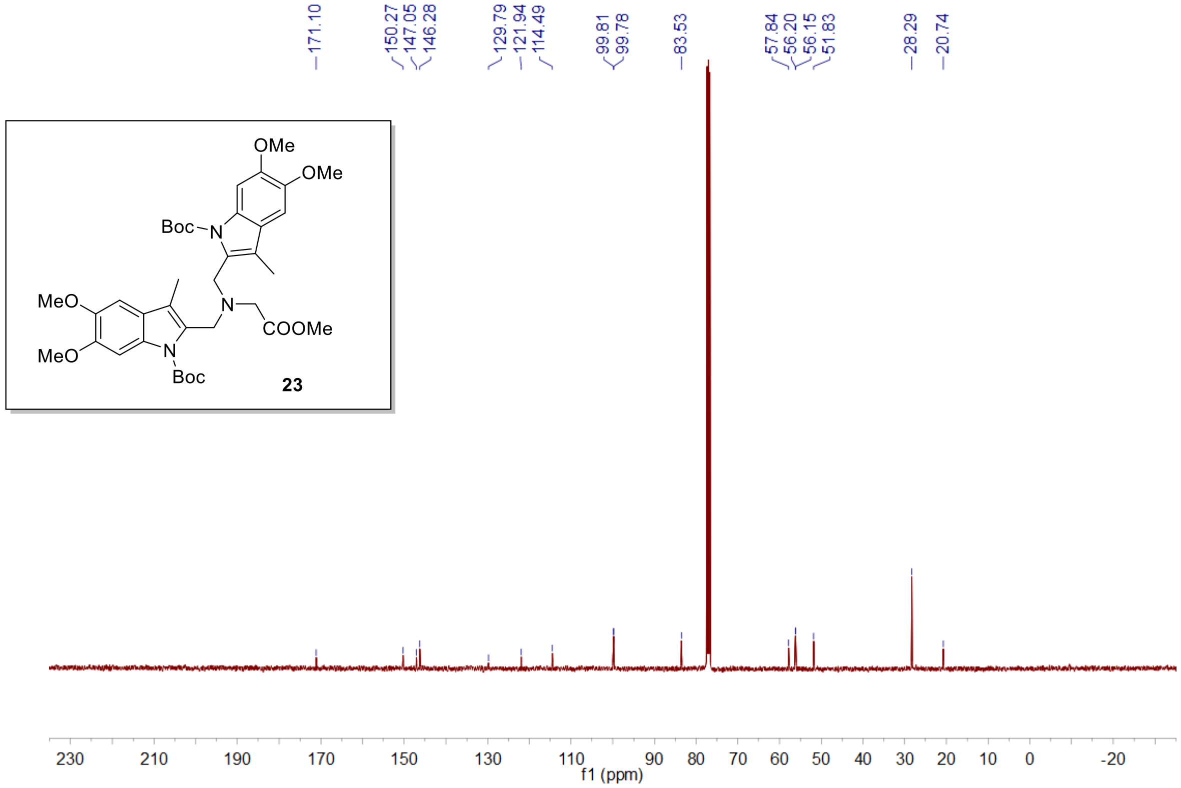

^{1}H-NMR spectrum of compound **22-f** (400 MHz, $CDCl_3$)

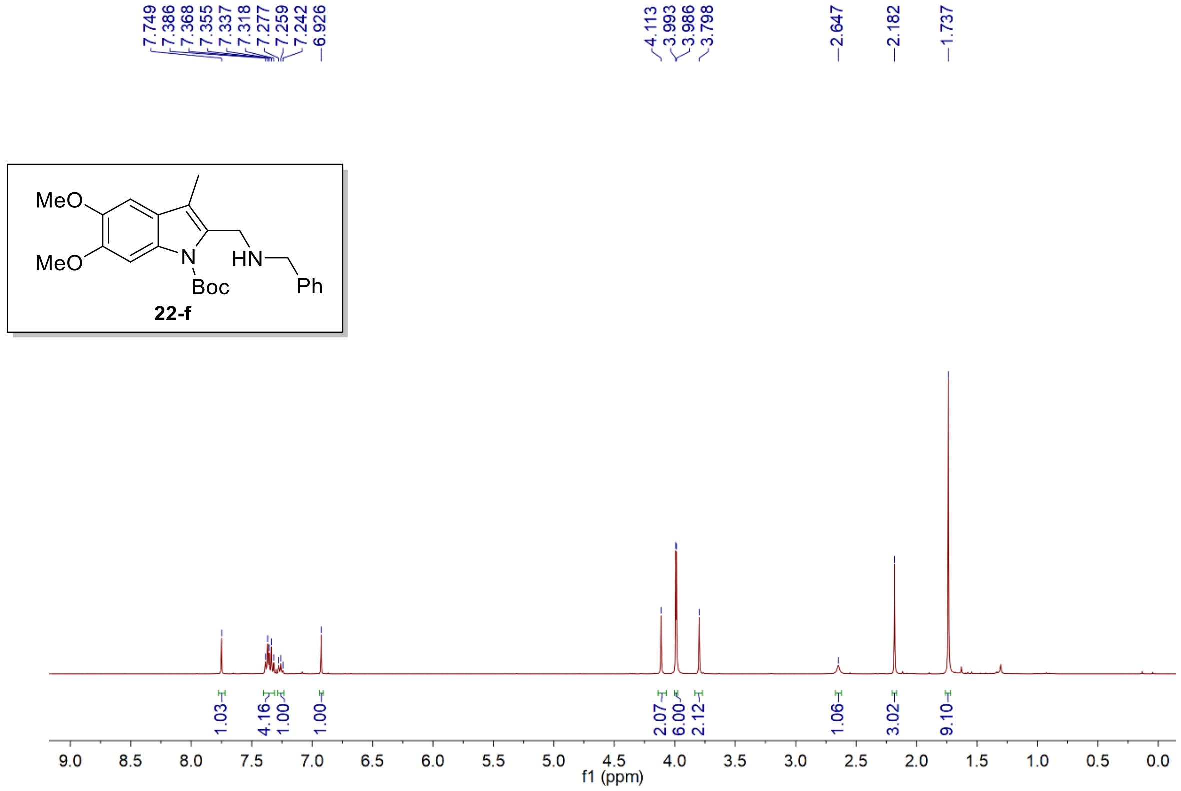

^{13}C-NMR spectrum of compound **22-f** (101 MHz, $CDCl_3$)

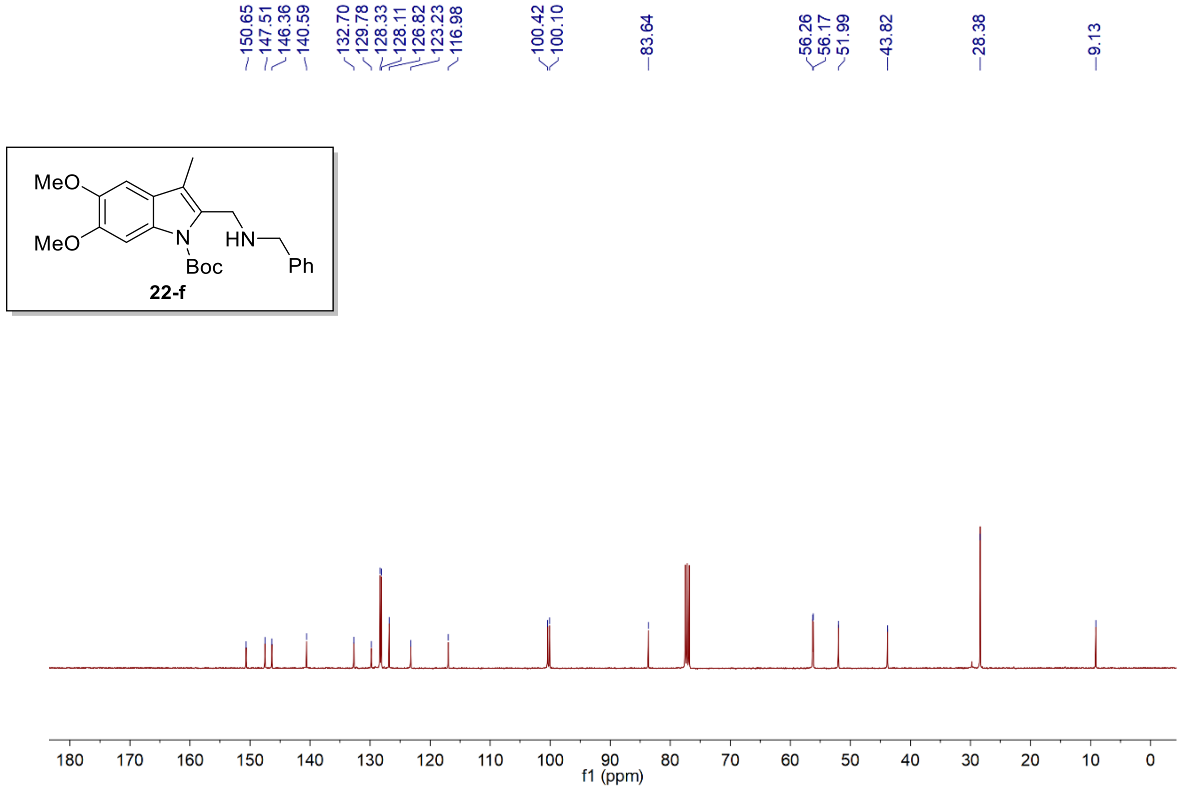

^{1}H-NMR spectrum of compound **24** (400 MHz, $CDCl_3$)

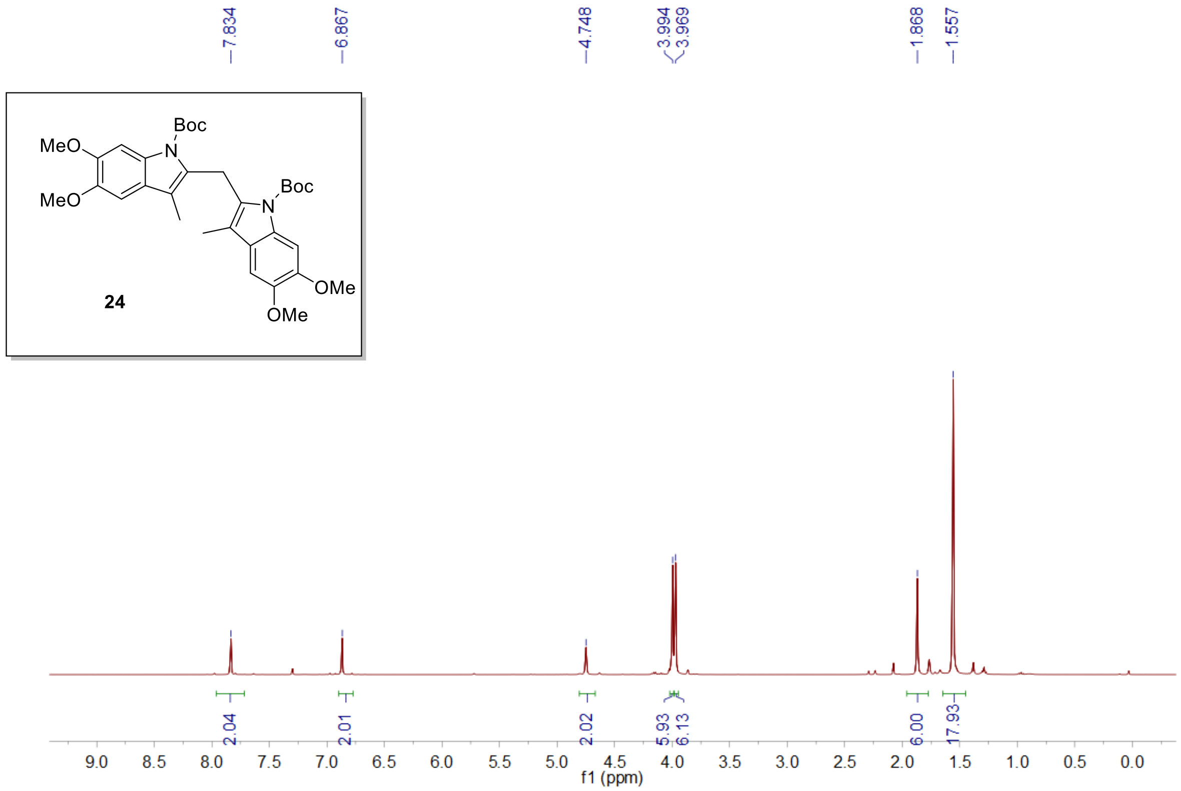

^{13}C-NMR spectrum of compound **24** (101 MHz, $CDCl_3$)

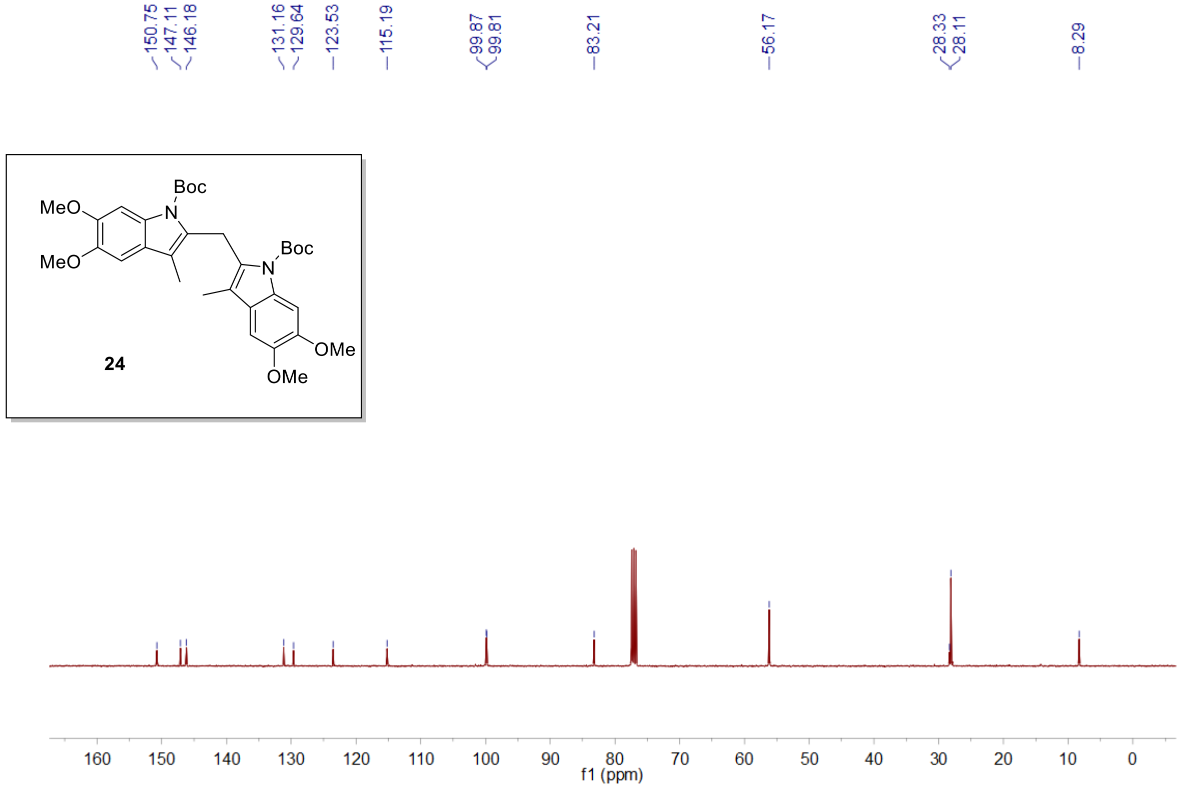

^{1}H-NMR spectrum of compound [D_4]-**6a** (400 MHz, $CDCl_3$)

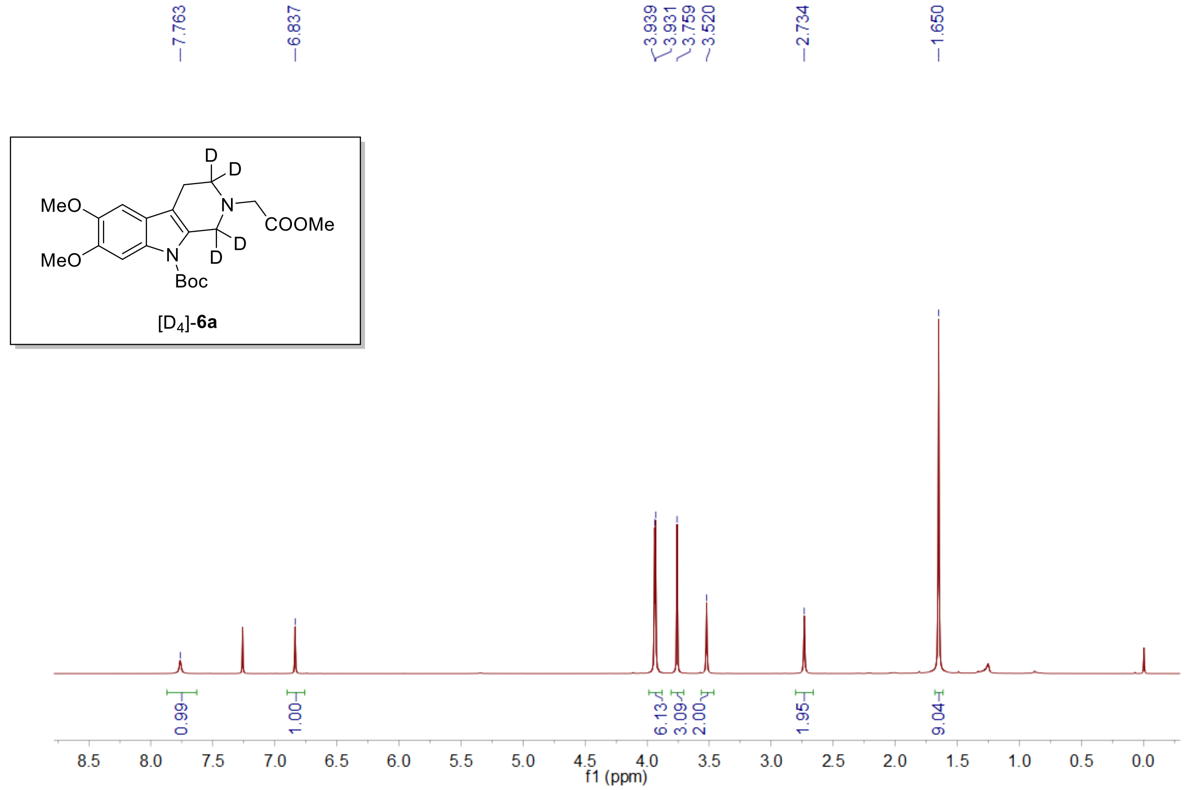

^{13}C-NMR spectrum of compound [D_4]-**6a** (101 MHz, $CDCl_3$)

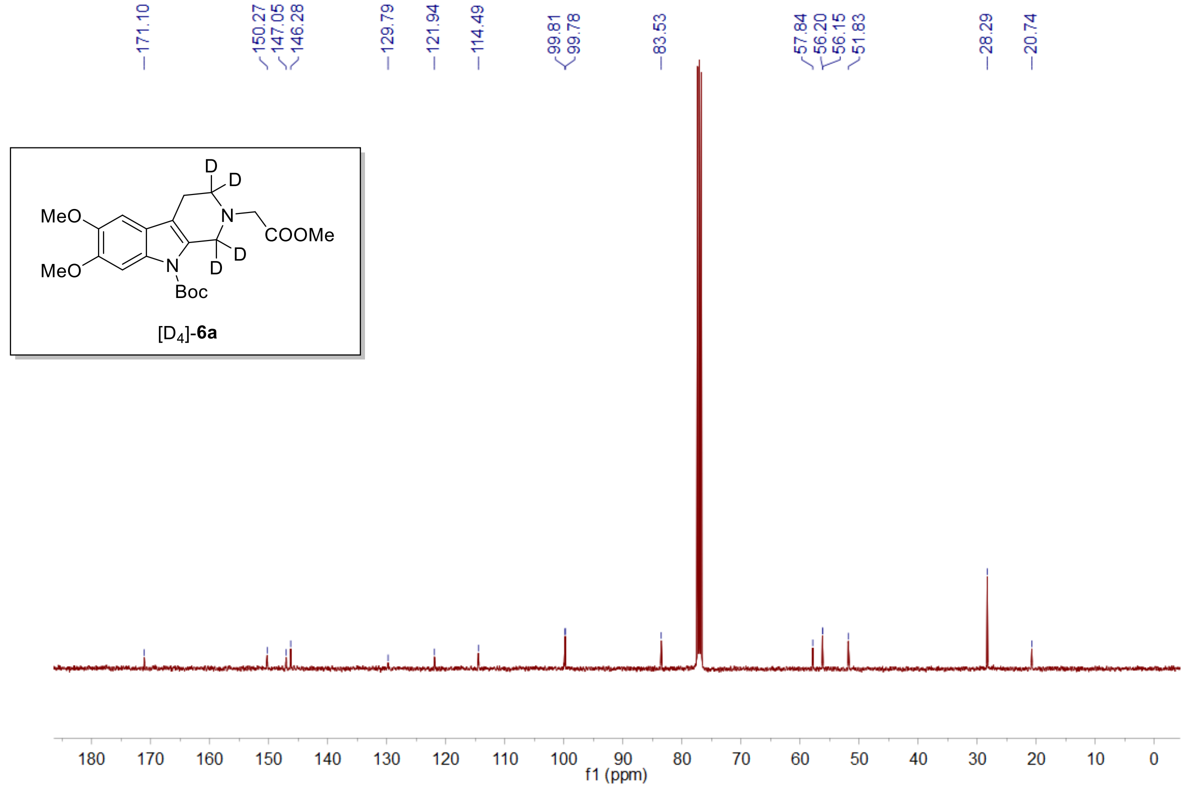

^{1}H-NMR spectrum of compound [D$_2$]-**4a** (400 MHz, $CDCl_3$)

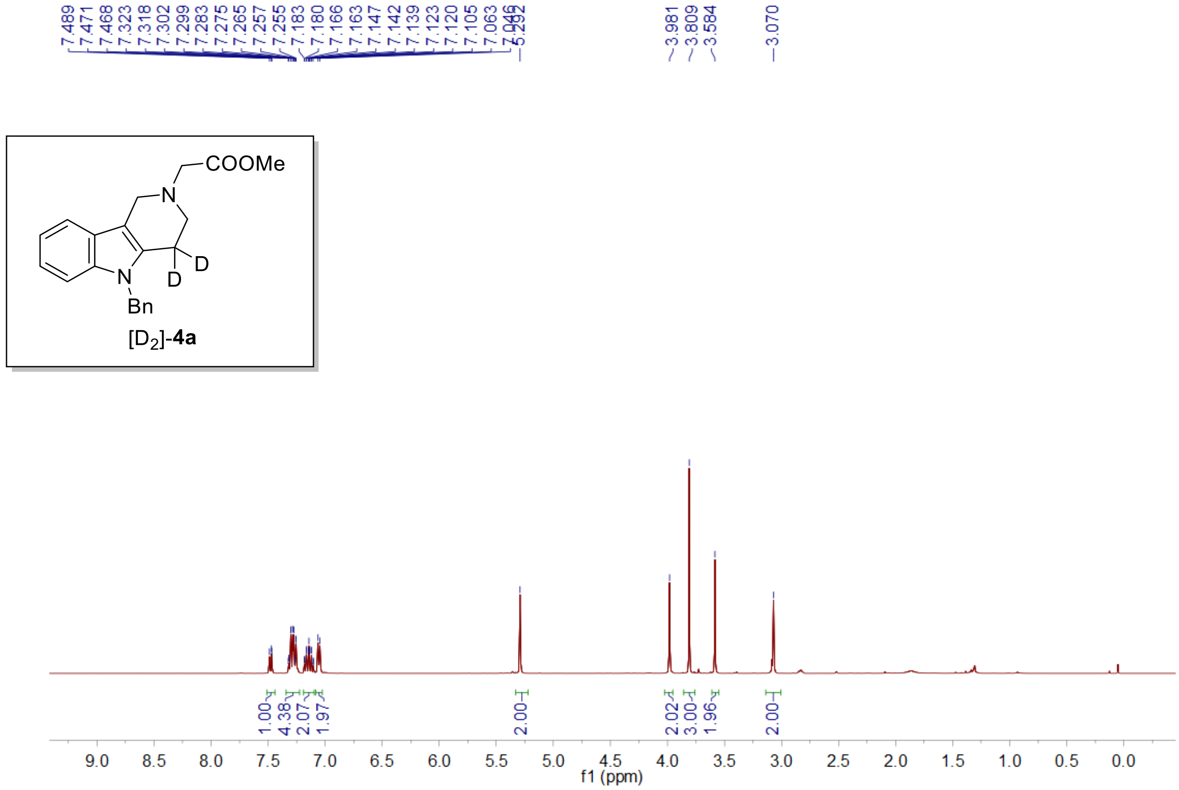

^{13}C-NMR spectrum of compound [D$_2$]-**4a** (101 MHz, $CDCl_3$)

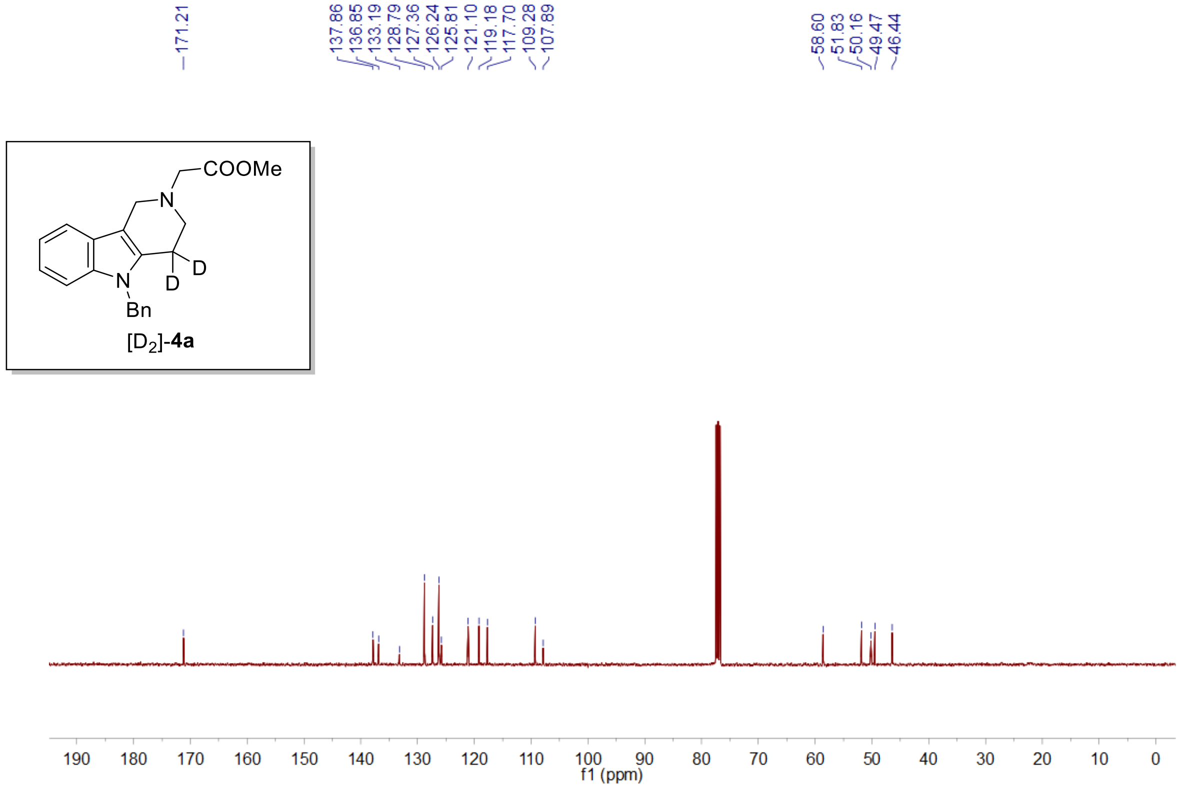

^{1}H-NMR spectrum of compound [D_2]-**6a** (400 MHz, $CDCl_3$)

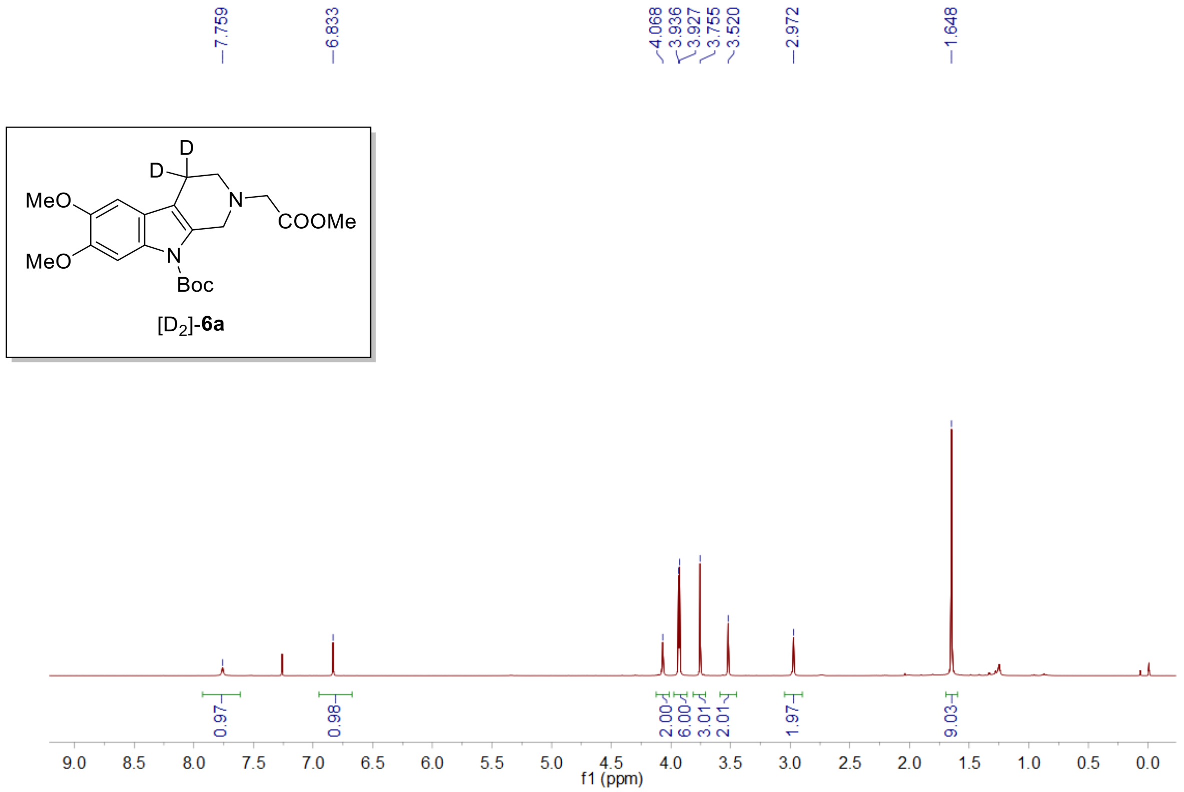

^{13}C-NMR spectrum of compound [D_2]-**6a** (101 MHz, $CDCl_3$)

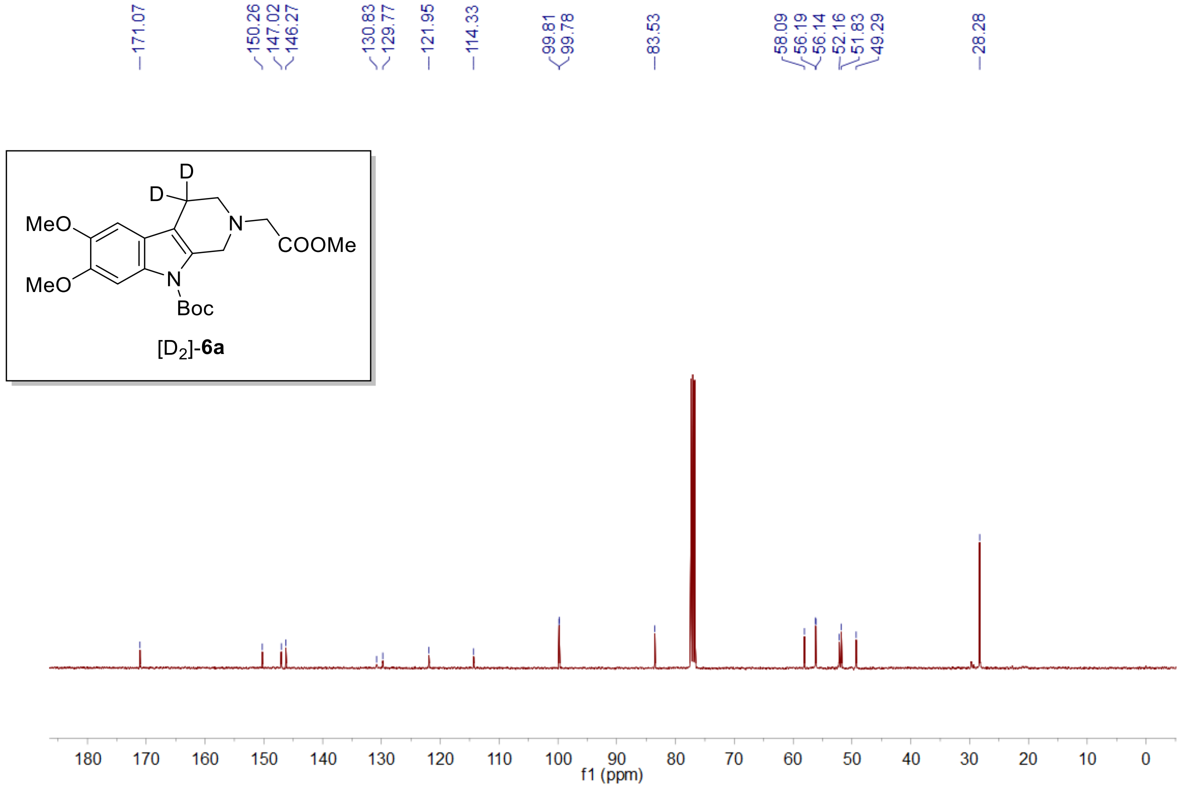

^{1}H-NMR spectrum of compound **1a**/[D]-**1a** ***KIE*** (400 MHz, $CDCl_3$)

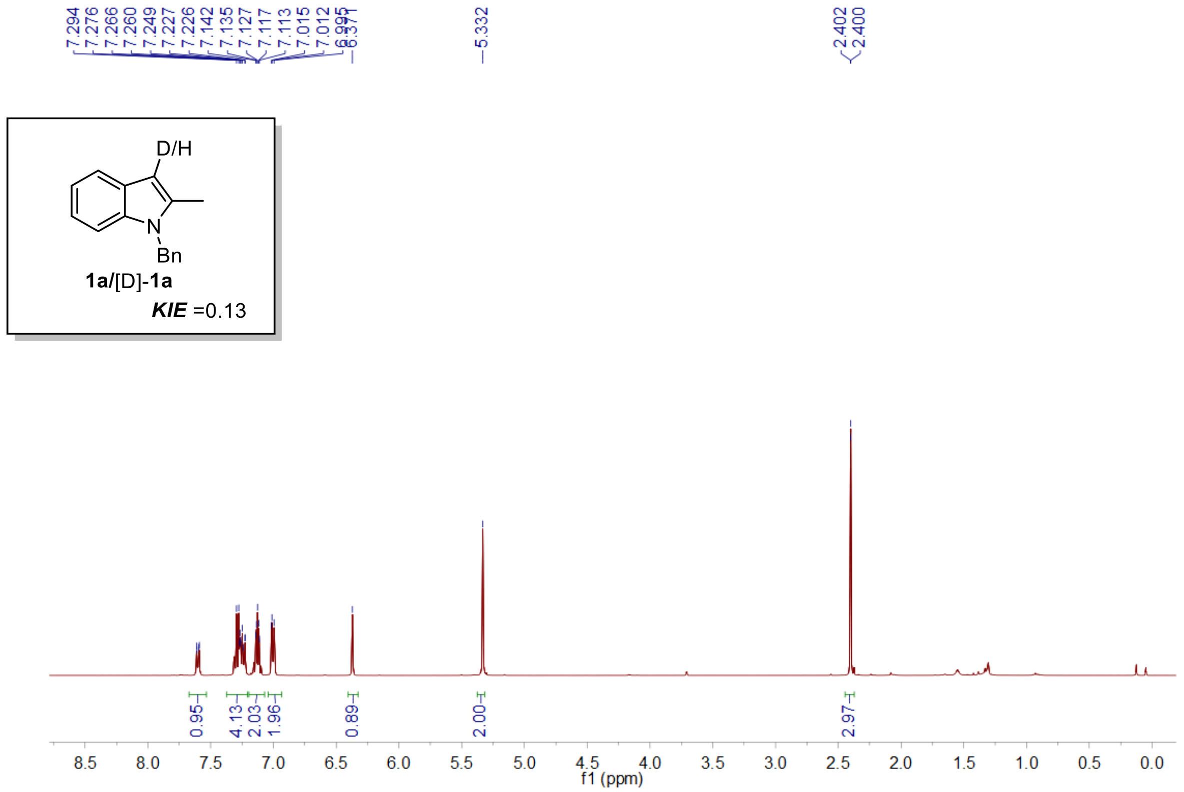

^{1}H-NMR spectrum of compound **4a**/[D_2]-**4a** ***KIE*** (400 MHz, $CDCl_3$)

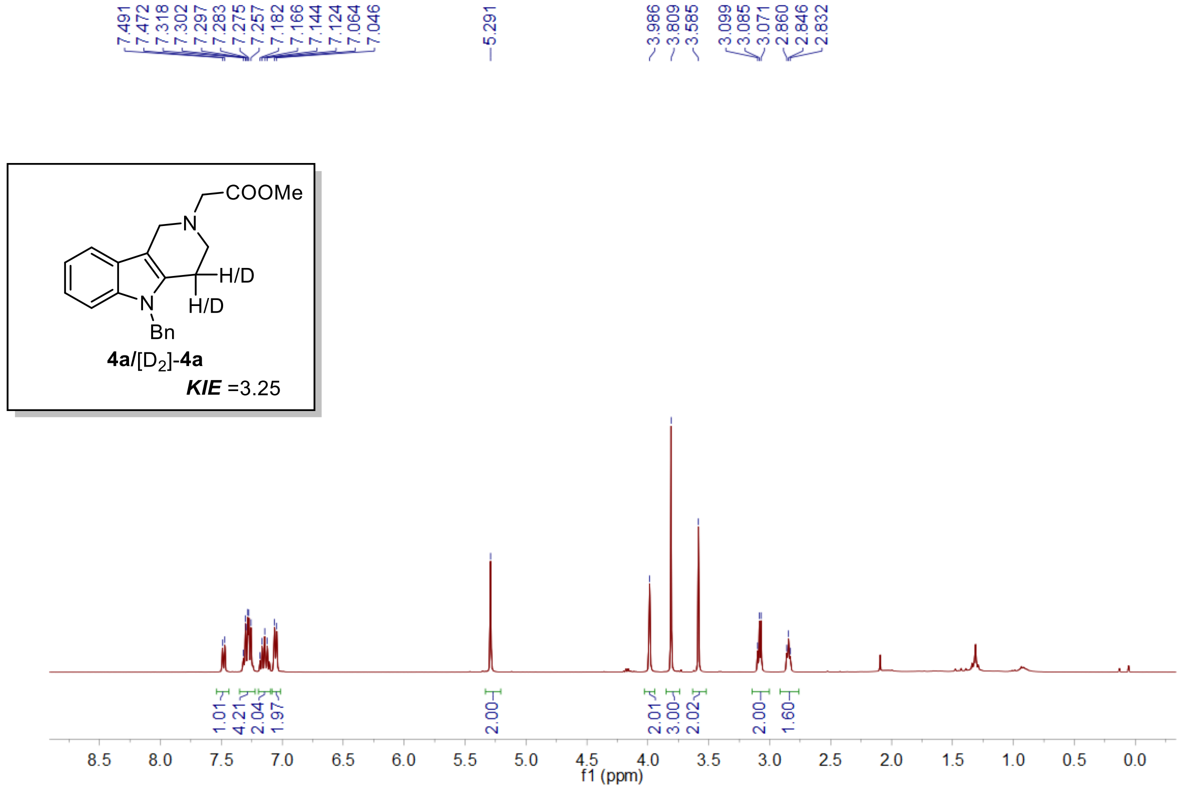

^{1}H-NMR spectrum of compound **5a**/[D]-**5a** ***KIE*** (400 MHz, $CDCl_3$)

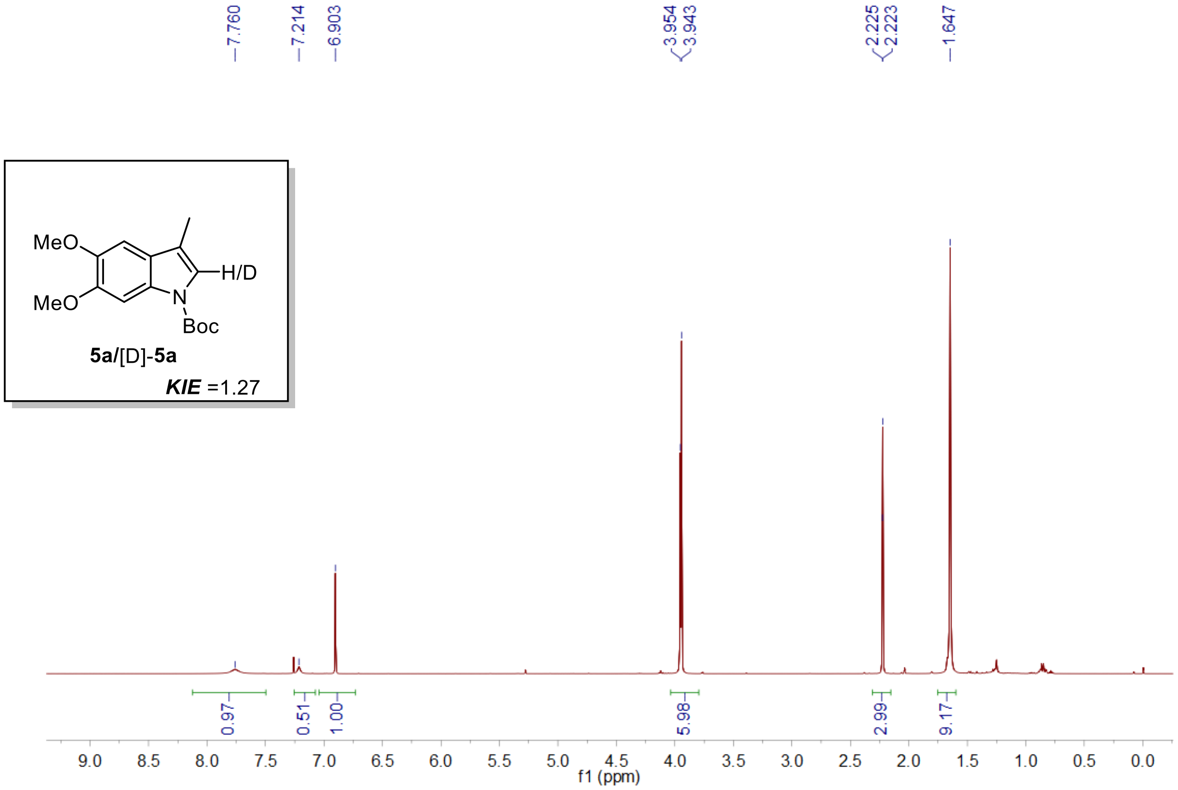

^{1}H-NMR spectrum of compound **6a**/[D_2]-**6a** ***KIE*** (400 MHz, $CDCl_3$)

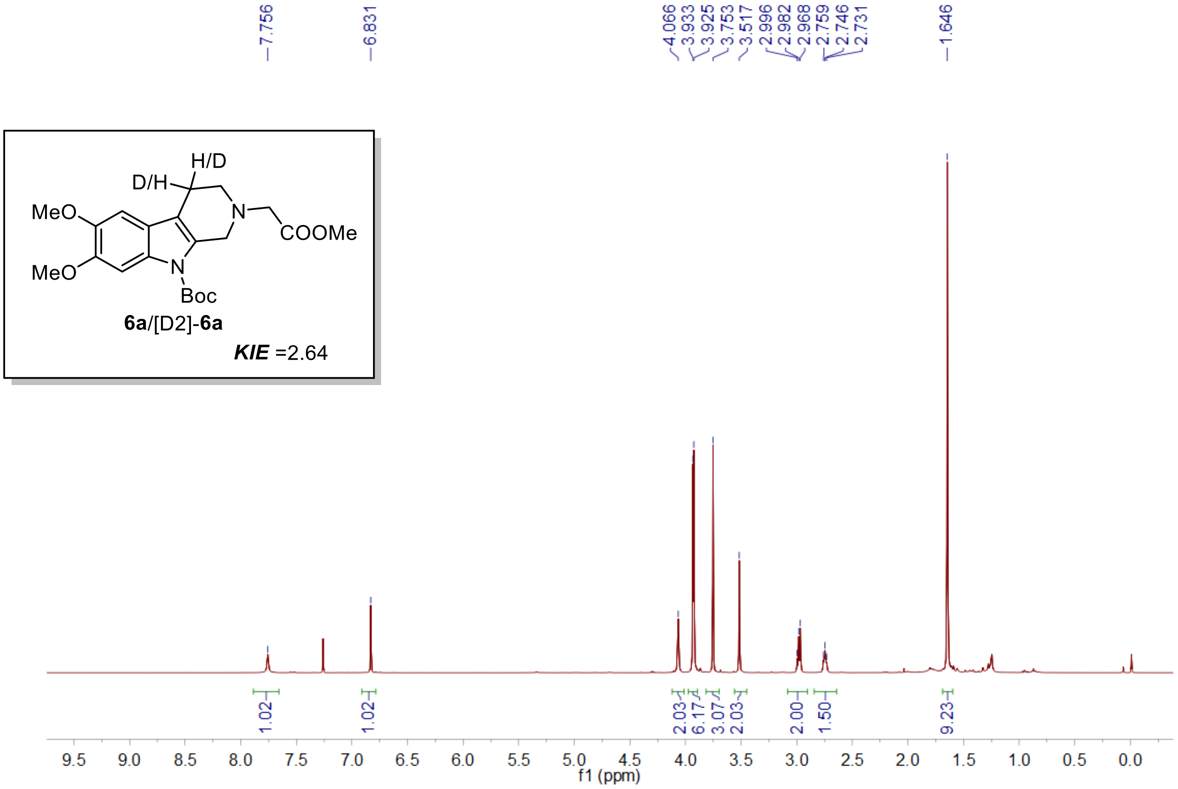

9. References

1. Gottlieb, H. E., Kotlyar, V. & Nudelman, A. NMR Chemical Shifts of Common Laboratory Solvents as Trace Impurities. *J. Org. Chem.* **62**, 7512-7515 (1997).
2. Smith, A. J. et al. Electron-Transfer and Hydride-Transfer Pathways in the Stoltz–Grubbs Reducing System (KOtBu/Et3SiH). *Angew. Chem. Int. Ed.* **56**, 13747-13751 (2017).
3. Sera, M., Mizufune, H., Ueda, T., Mineno, M. & Zanka, A. Integrated Pd-catalyzed cross-coupling strategies for furnishing α-carbolines. *Tetrahedron* **73**, 5946-5958 (2017).
4. Zhao, J.-H. et al. Visible-light-mediated borylation of aryl and alkyl halides with a palladium complex. *Org. Biomol. Chem.* **18**, 4390-4394 (2020).
5. Mondal, D., Pramanik, S. & Chowdhury, C. Palladium(0)-Catalyzed Heteroannulations of Allenamides: General Synthesis of δ-Carbolines and Benzofuro[3,2-b]pyridines. *Org. Lett.* **24**, 8698-8702 (2022).
6. Zhang, J., Torabi Kohlbouni, S. & Borhan, B. Cu-Catalyzed Oxidation of C2 and C3 Alkyl-Substituted Indole via Acyl Nitroso Reagents. *Org. Lett.* **21**, 14-17 (2019).
7. Tomakinian, T., Guillot, R., Kouklovsky, C. & Vincent, G. Direct Oxidative Coupling of N-Acetyl Indoles and Phenols for the Synthesis of Benzofuroindolines Related to Phalarine. *Angew. Chem. Int. Ed.* **53**, 11881-11885 (2014).
8. Yu, L. et al. Diastereoselective Access to Triazolo[1,2-a]indolines via a Bio-Inspired Oxidative Cyclization of NH-Indoles. *J. Org. Chem.* **87**, 15114-15119 (2022).
9. Joshi, M., Patel, M., Tiwari, R. & Verma, A. K. Base-Mediated Selective Synthesis of Diversely Substituted N-Heterocyclic Enamines and Enaminones by the Hydroamination of Alkynes. *J. Org. Chem.* **77**, 5633-5645 (2012).
10. Hadjipavlou-Litina, D., Magoulas, G. E., Krokidis, M. & Papaioannou, D. Syntheses and evaluation of the antioxidant activity of acitretin analogs with amide bond(s) in the polyene spacer. *Eur. J. Med. Chem.* **45**, 298-310 (2010).
11. Anisley S., Fernando M., Samuel S.-P. & Roberto S. PTSA-Catalyzed Reaction of Indoles with 2-Oxoaldehydes: Synthesis of α,α-Bis(indol-3-yl) Ketones. *ChemistrySelect* **2**, 787-790 (2017).
12. Nandwana, N. K., Patel, O. P. S., Srivathsa, M. R. & Kumar, A. Dual Role of Glyoxal in Metal-Free Dicarbonylation Reaction: Synthesis of Symmetrical and Unsymmetrical Dicarbonyl Imidazoheterocycles. *ACS Omega* **4**, 10140-10150 (2019).
13. Wiedemann, M., Altmann, P. J. & Hintermann, L. Mannich *N*-Indolylmethylation of Amino Acids. *Synthesis* **49**, 2257-2265 (2017).
